# Enzyme-mimicking peripheral substituents beyond the second coordination sphere tune single-atom catalysts for periodate activation

**DOI:** 10.1038/s41467-026-77805-5

**Published:** 2026-09-17

**Authors:** Sijia Jin, Xiaofeng Tang, Zhiqiao He, Shuang Song, Yaqi Cai, Haiyan Zhang, Bo Lai, Tao Zeng

**Affiliations:** 1 Zhejiang Key Laboratory of Environment and Health of New Pollutants, School of Environment, Hangzhou Institute for Advanced Study, University of Chinese Academy of Sciences, Hangzhou, 310024 P.R. China; 2 Zhejiang Key Laboratory of Low-carbon Control Technology for Industrial Pollution, College of Environment, Zhejiang University of Technology, Hangzhou, Zhejiang 310032 PR China; 3 State Key Laboratory of Environmental Chemistry and Ecotoxicology, Research Center for Eco-Environmental Sciences, Chinese Academy of Sciences, Beijing, 100085 PR China; 4 State Key Laboratory of Hydraulics and Mountain River Engineering, College of Architecture and Environment, Sichuan University, Chengdu, 610065 PR China

**Keywords:** Pollution remediation, Catalytic mechanisms

## Abstract

Single-atom catalysts (SACs) are promising enzyme-mimetic active centers, yet mimicking long-range regulation by distal binding pockets in natural metalloenzymes remains challenging. Here, we synthesize conjugated organic SACs bearing tunable peripheral substituents beyond the second coordination sphere (denoted as R-FeSAC; R = F, H, or NH_2_) to regulate periodate (PI) activation. F-FeSAC outperforms its H- and NH_2_-substituted counterparts because long-range electronic regulation reconfigures the Fe-site microenvironment and strengthens PI adsorption and activation. In situ Raman spectroscopy, galvanic oxidation assays, and electrochemical analyses show that R-FeSAC activates PI to form high-potential metastable intermediates, enabling a direct electron-transfer pathway (ETP) for selective pollutant removal through polymerization, rather than radical or singlet-oxygen pathways. Fluorine substitution reinforces Fe–O interaction with PI and accelerates electron-transfer kinetics through an electron-withdrawing effect. Consistent relationships among pollutant half-wave potentials (*φ*_1/2_), LUMO_(catalyst/PI)_–HOMO_(pollutant)_ energy gaps, and degradation reactivity further support the ETP and indicate that pollutant electron-donating ability governs selectivity. Moreover, an F-functionalized membrane exhibits enhanced durability during continuous-flow operation, while a pilot-scale ETP reactor achieves efficient pollutant degradation in real wastewater without iodate leakage. These findings establish beyond-second-sphere peripheral functionalization as an enzyme-inspired strategy for PI activation and polymerization-mediated water purification.

## Abstract (web summary)

The study completes the enzyme analogy beyond the second coordination sphere through distal-pocket control of single-atom Fe centers, directing periodate activation toward electron-transfer polymerization and iodate-leakage-free wastewater treatment.

## Introduction

Advanced oxidation processes (AOPs) utilizing solid-phase oxidants such as permanganate, persulfate, and periodate (PI, IO_4_^−^) have garnered significant attention to address pressing environmental challenges^1–3^. In contrast to permanganate (e.g., KMnO_4_) and persulfate (e.g., peroxydisulfate, PDS, or peroxymonosulfate, PMS), PI-based AOPs circumvent chromaticity issues and significant pH fluctuations. Their demonstrated cost-effectiveness under specific operational conditions, coupled with advantages like reduced sludge production in certain cases, as evidenced by multiple studies^4–6^, has consequently spurred considerable interest in these systems for water decontamination applications. Similar to other oxidants, PI itself is relatively stable and exhibits limited oxidative capacity against organic pollutants (E^0^ = +1.60 V vs. NHE)^7^. Structurally, PI is a high-valent iodine (VII) oxyanion with a highly symmetric oxygen coordination environment, which is fundamentally different from peroxide-based oxidants such as PMS and PDS. Accordingly, PI activation does not rely on O–O bond cleavage, but is generally associated with I–O bond activation and subsequent electron-transfer, oxygen-transfer, or reactive-species generation processes. To enhance its oxidative reactivity, diverse activation strategies have been developed, including physical methods such as ultrasonication^8^ and light irradiation^9^, chemical approaches such as alkali activation, reducing agents^10^, and transition metal ions^11^, as well as freezing-induced activation^12^. Despite rapid progress in this field, PI-mediated organic pollutant degradation still suffers from sluggish kinetics, highlighting the need to design highly active and resource-efficient activators.

Recent reports highlight the efficacy of metal-based heterogeneous activators (e.g., FeS, manganese oxides (MnO_2_, Mn_3_O_4_, and Mn_2_O_3_), and iron-based bimetallic nanoparticles)^13^ in activating PI to produce reactive oxygen species (ROS), such as hydroxyl radicals (•OH), iodate radicals (IO_3_^•^), and superoxide radical (O_2_^•−^)^1,14,15^. In these systems, PI activation is commonly initiated by interfacial electron transfer and/or I–O bond cleavage at the catalyst surface, which subsequently gives rise to different oxidizing intermediates and reaction pathways. However, these radical-based pathways are often hindered by scavenging effects from coexisting inorganic ions and natural organic matter. In contrast, nonradical pathways, including electron-transfer processes (ETP), singlet oxygenation, and high-valent metal-induced oxidation, have emerged as promising alternatives^16–19^. ETP, in particular, mediates electron transfer from contaminants to oxidants, enabling selective degradation of organics while maintaining resilience against short lifetimes and diffusion limitations typical of radical species^20^. ETP-based oxidation offers distinct advantages, including high selectivity for electron-rich substrates, stability across complex water matrices, reduced dependence on freely diffusing radical species, and efficient oxidant utilization^21^. Furthermore, strong adsorption of reactants on the catalyst surface enhances PI activation and oxidation efficiency, even at low concentrations. This mechanism effectively mitigates radical quenching, a key limitation of conventional Fenton-like systems where reactive species such as IO_3_^•^ and •OH are characterized by short lifetimes. Lai et al^7^. recently identified an ETP pathway in the functionalized carbon nanotube (CNT)/PI system, in which CNTs were modified through oxidative treatment to introduce oxygen-containing functional groups, defect sites, and edge structures. Carbonyl/ketonic groups and double-vacancy defects were proposed to function as electron-shuttling motifs that mediate direct electron transfer from organic contaminants to PI. This mechanistic insight into ETP is pivotal, as it underscores the critical role of catalyst design in optimizing PI adsorption and interfacial redox potentials. However, in CNT-based systems, ETP can be influenced simultaneously by conductivity, graphitic domains, oxygen-containing groups, vacancy defects, edge sites, and other surface features. As a result, although CNT/PI studies establish carbon-surface-mediated ETP, precise regulation of ETP at a structurally defined catalytic site remains insufficiently explored. Therefore, the rational design of catalysts to optimize PI adsorption and redox potential is essential for developing efficient and tunable ETP-based water treatment technologies.

Single-atom catalysts (SACs), a groundbreaking class of catalysts, have gained prominence in AOPs due to their unparalleled atomic efficiency and superior catalytic performance, offering significant potential to overcome the sluggish reaction kinetics of PI-based AOPs. Among these, metal-nitrogen (M-N_*x*_) coordination structures exhibit exceptional catalytic activity for PI activation, attributed to their distinctive electronic properties and charge states^22^. The coordination environment of M-N_*x*_ sites is a critical determinant in optimizing the performance of M-N-C catalysts by modulating the local electronic structure^23^. Nevertheless, several key challenges persist. First, conventional pyrolysis synthesis lacks the precision required to control the coordination environment of SACs. The inherent structural uncertainties of these uncontrolled high-temperature processes often lead to metal aggregation and ill-defined active sites, which obstruct a precise mechanistic understanding of SAC catalysis. Second, while the first coordination shell (FCS) is recognized as pivotal, research efforts have predominantly focused on its optimization. The influence of the second and higher coordination shells, which are not directly bonded to the metal center but critically modulate the electronic structure and catalytic efficiency, remains a largely unexplored frontier. More importantly, prevailing modulation strategies primarily involve tailoring coordination numbers, heteroatom doping, or creating carbon defects. In contrast, the potential of peripheral organic functional group substitution, a strategy that mirrors the long-range electronic effects in natural metalloenzymes, to regulate the catalytic properties of SACs has rarely been investigated, let alone for PI activation and the elucidation of structure-activity relationships.

Inspired by distal-pocket regulation in natural metalloenzymes, we leverage conjugated organic frameworks as programmable scaffolds to construct precision SACs. This strategy provides a precise route to manipulate the long-range coordination environment through peripheral organic group substitution. By incorporating electron-withdrawing (e.g., -F) or electron-donating (e.g., -NH_2_) groups into the conjugated scaffolds, we synthesized a series of well-defined R-FeSACs (R = F, H, NH_2_), achieving targeted electronic modulation of the Fe single-atom centers. Experimental results demonstrate that F-FeSAC exhibits significantly enhanced activity for PI activation compared to its H- and NH_2_- analogs. Mechanistic studies reveal that this enhancement originates from long-range electronic effects, whereby electron-withdrawing peripheral groups reconfigure the Fe-site electronic microenvironment, strengthen PI binding, and facilitate an efficient ETP between pollutants and the catalyst/PI complex. Theoretical calculations further show that efficient ETP is favored when the pollutant HOMO is energetically well aligned with the LUMO of the catalyst/PI complex. A pilot-scale ETP reactor was constructed, demonstrating potential for treating wastewater without iodate leakage. This work provides atomic-level insight into how peripheral organic substituents regulate long-range electronic interactions in SACs, establishing an enzyme-inspired paradigm for the electronic optimization of high-efficiency, non-radical oxidation catalysts specifically for PI activation, and advancing sustainable strategies for water purification.

## Results

### Synthesis and structure characterization

**Fig. 1:**
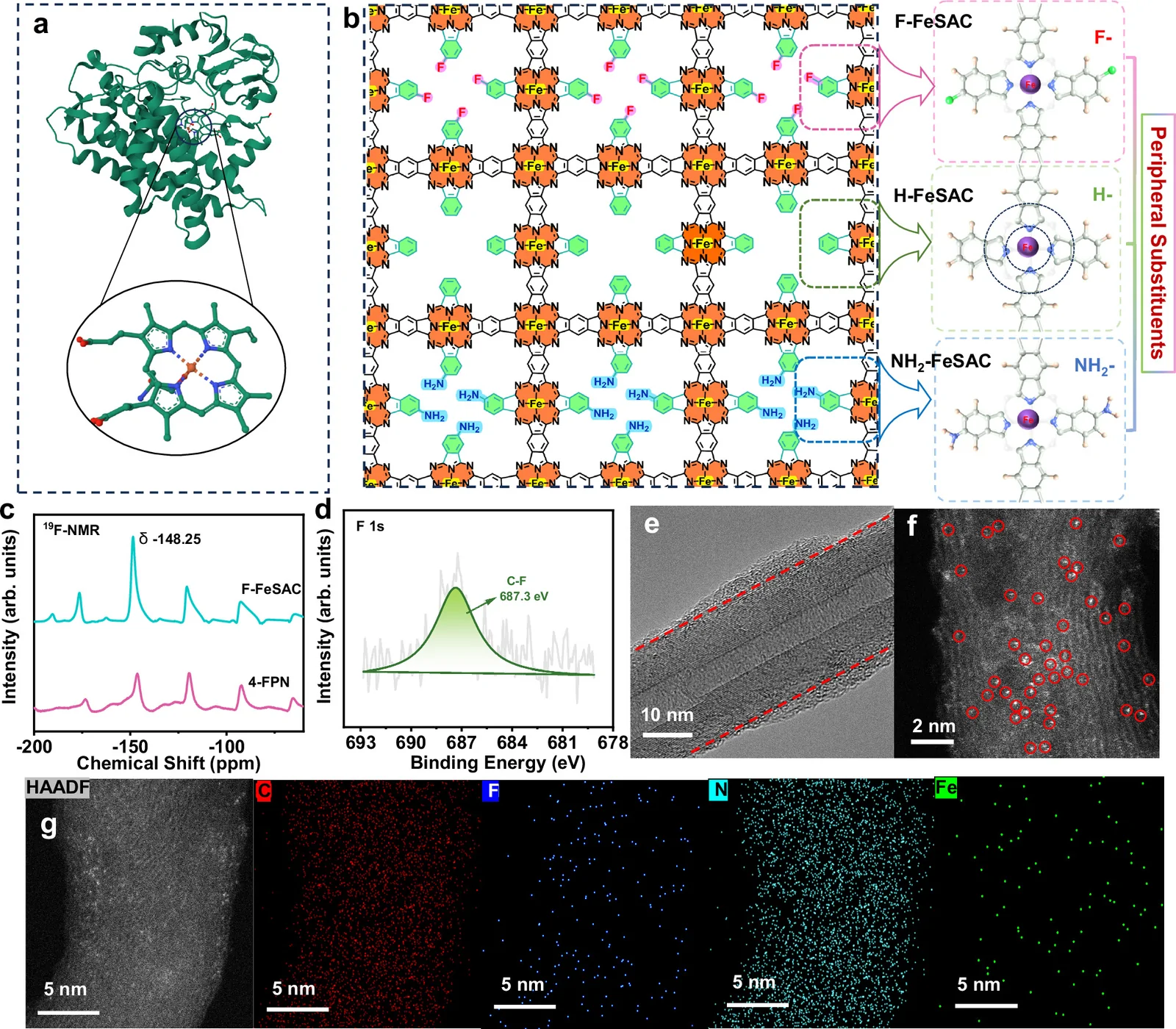
Structural models and characterization of as-prepared FeSACs. **a** Model diagram of the Fe active site in natural metalloenzymes. Fe, C, N and O atoms are shown in orange, green, blue and red, respectively. **b** Structural schematic of R-FeSACs bearing -F, -H or -NH_2_ peripheral substituents, denoted F-FeSAC, H-FeSAC and NH_2_-FeSAC, respectively. In the molecular models, Fe, C, N, F and H atoms are shown in purple, light gray, blue, light green and pale pink, respectively. The inner and outer circles indicate the first and second coordination spheres of Fe, respectively. Crystal structures visualized using VESTA software^48^. **c** ^19^F solid-state NMR spectra of F-FeSAC and 4-FPN. **d** XPS F 1*s* spectra of F-FeSAC. **e** HRTEM image of F-FeSAC. Red dashed lines outline the nanotube boundaries. **f** AC-HAADF-STEM image of F-FeSAC. Red circles highlight isolated Fe atoms. Images in e and f are representative of three independent experiments with similar results. **g** EDS images of F-FeSAC. Source data, including the original, unannotated versions of the images shown in **e** and **f**, are provided as a Source Data file.

Natural enzymes derive their exceptional catalytic efficiency from precisely defined metal centers embedded within well-organized coordination environments, where structural features beyond the immediate coordination sphere exert decisive regulatory effects (Fig. 1a). SACs emulate this enzymatic paradigm by hosting isolated metal sites with uniform coordination structures and maximized atomic utilization. Importantly, the catalytic behavior of SACs can be further tuned through rational modulation of peripheral substituents beyond the second coordination sphere, analogous to remote amino-acid residues in enzyme active pockets. Realizing such enzyme-inspired peripheral regulation, however, requires synthetic strategies capable of decoupling outer-sphere modification from the preservation of intrinsic Fe-N-C coordination.

This synthetic approach diverges from conventional M-N-C catalyst methods by enabling precise control of intrinsic peripheral substituents while maintaining Fe-N-C first coordination shells, eliminating complex pyrolysis (Fig. 1b). The self-polymerization confines transition metals within conjugated organic frameworks cavities, where coordinative stabilization generates well-defined SAC active sites. To engineer the second-beyond coordination environments, we functionalized phthalonitrile (PN) derivatives with tunable substituents (R = -F, -H, -NH_2_). Taking F-FeSAC as a representative example, the 4-fluorophthalonitrile (4-FPN) monomer containing fluorine functional groups serves as an effective F-doping source. Meanwhile, the remaining two cyano groups in the monomer participate in polymerization with benzene-1,2,4,5-tetracarbonitrile (BTC) building blocks, ultimately constructing a network framework through coordinated assembly. FTIR spectroscopy verifies the macrocyclic structure of conjugated organic frameworks, displaying C=C stretching vibrations at 1396 cm^−1^ and a C–H deformation band at 731 cm^−1^ (Supplementary Fig. 1)^24^. These spectral features indicate the successful formation of a phthalocyanine-like conjugated network through polymerization in the R-FeSAC. Notably, the complete disappearance of cyano group signals from both BTC and 4-FPN monomers confirms the effective participation of nitrile groups in the polymerization process. The amorphous nature of the catalyst was verified by XRD characterization (Supplementary Fig. 2), which showed no discernible diffraction peaks corresponding to Fe clusters or crystalline nanostructures. This structural feature primarily originates from the kinetically irreversible reactions occurring during the polymerization process. Successful fluorine incorporation was demonstrated by ^19^F solid-state nuclear magnetic resonance (NMR) spectroscopy, which exhibited a resonance at −148.25 ppm matching the chemical environment of the 4-FPN monomer^25^ (Fig. 1c). Complementary evidence was provided by XPS analysis, where the F 1*s* spectrum displays a characteristic peak at 687.3 eV corresponding to the C–F bond (Fig. 1d), further confirming the effective doping of fluorine functional groups into the conjugated organic frameworks matrix. To probe the coordination environment, solid-state ^13^C NMR spectroscopy was performed on R-FeSAC samples. As depicted in Supplementary Fig. 3, a broad resonance spanning 100–130 ppm is assigned to aromatic carbons within benzene rings, while a distinct signal at ~171 ppm corresponds to C=N groups in the pyrrole ring^24,26,27^.

Scanning electron microscopy (SEM) revealed that F-FeSAC maintains an interwoven fibrous architecture, with CNTs displaying surface roughness attributable to a thin polymeric coating (Supplementary Fig. 4). High-resolution transmission electron microscopy (HRTEM) further confirms the uniform conjugated organic frameworks-based SACs layers on CNT surfaces without apparent aggregation (Fig. 1e and Supplementary Fig. 5). Atomic-resolution Aberration-corrected high-angle annular dark-field scanning transmission electron microscopy (AC-HAADF-STEM) imaging directly visualizes atomically dispersed Fe sites in F-FeSAC, manifesting as bright spots highlighted by red circles (Fig. 1f). Elemental mapping corroborates the homogeneous spatial distribution of C, N, F, and Fe throughout the composite (Fig. 1g). Inductively coupled plasma optical emission spectrometry (ICP-OES) reveals comparable Fe loadings among NH_2_-FeSAC (2.41 wt%), H-FeSAC (2.65 wt%), and F-FeSAC (2.59 wt%), indicating that Fe species are effectively incorporated into the conjugated organic frameworks scaffold. Importantly, the introduction of high-coordination-shell dopants does not compromise metal retention. Brunauer-Emmett-Teller (BET) measurements show a gradual increase in surface area and pore volume relative to FeSAC following peripheral substituents incorporation, while the integrity of the conjugated framework is preserved (Supplementary Table 1). Collectively, these complementary analyses confirm that peripheral organic group substitution has been successfully introduced into the conjugated organic frameworks.

### Local geometry and coordination environment

**Fig. 2:**
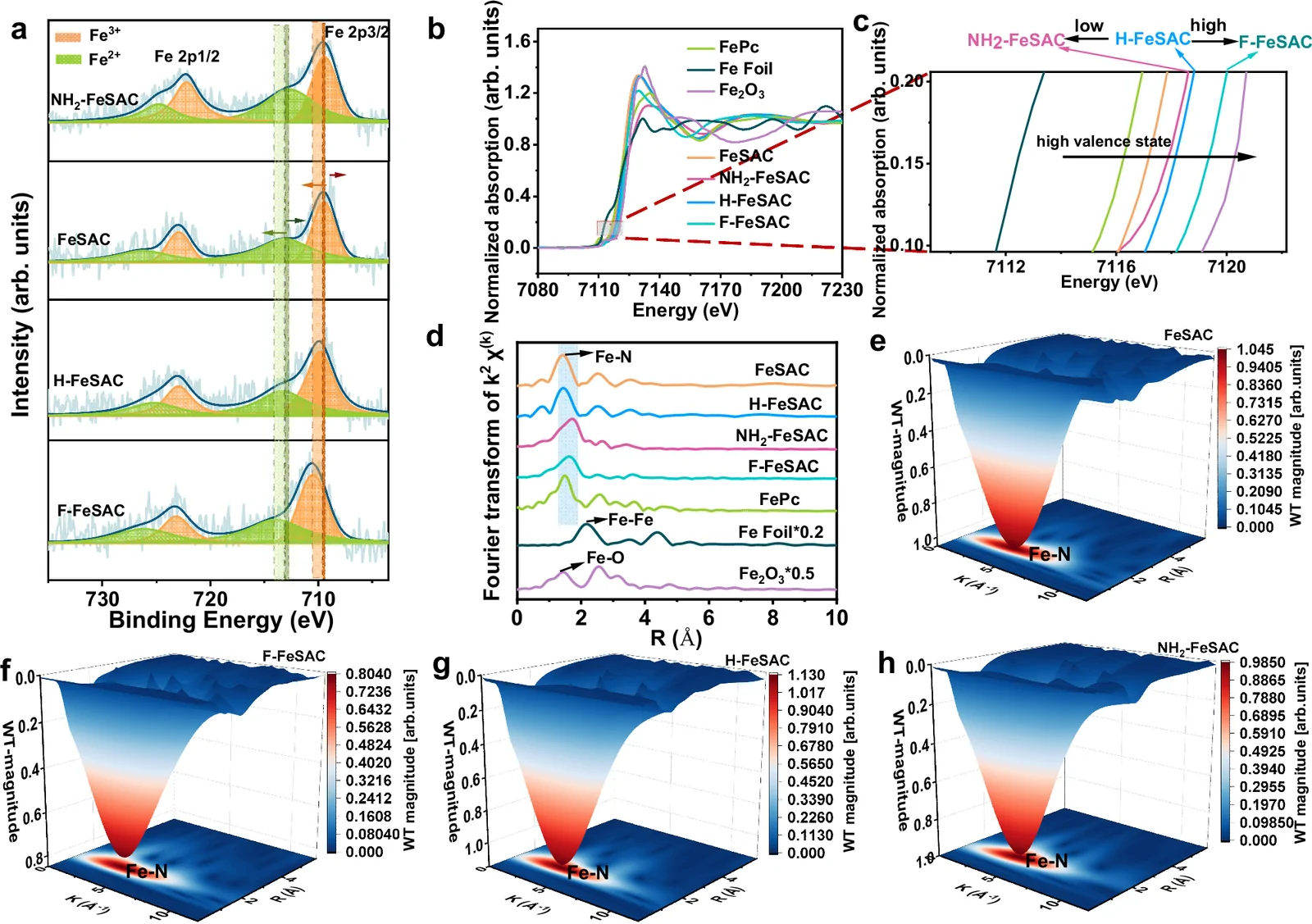
Coordination geometry and chemical states of as-prepared FeSACs. **a** XPS Fe 2*p* spectra of FeSAC and R-FeSACs. The green and orange shaded bars indicate the characteristic binding-energy ranges of the Fe^2+^ and Fe^3+^ components, respectively, while the arrows highlight the corresponding binding-energy shifts among the samples. **b** Fe *K*-edge XANES spectra and (**c**) the enlarged view of samples. **d** Fourier transform EXAFS spectra of samples. Wavelet transform analysis of (**e**) FeSAC, (**f**) F-FeSAC, (**g**) H-FeSAC and (**h**) NH_2_-FeSAC. Source data are provided as a Source Data file.

The chemical states and coordination environments of iron in R-FeSAC samples were systematically investigated using X-ray photoelectron spectroscopy (XPS) and X-ray absorption fine-structure (XAFS) spectroscopy. The Fe 2*p* XPS spectra (Fig. 2a) exhibited characteristic peaks at 709.5 eV (Fe^2+^) and 713.2 eV (Fe^3+^) in the 2*p*3/2 region, with corresponding features at 722.8 eV and 726.2 eV in the 2*p*1/2 region. Notably, the absence of a metallic Fe^0^ signature at 706.7 eV confirmed the atomic dispersion of iron, ruling out the formation of Fe nanoparticles or aggregates. Comparative analysis revealed distinct binding energy shifts across functionalized samples: H-FeSAC and F-FeSAC displayed positive shifts of 0.50-1.1 eV for both Fe^2+^ and Fe^3+^ states relative to pristine FeSAC, whereas NH_2_-FeSAC exhibited negative shifts of 0.44–0.65 eV compared to H-FeSAC. These shifts correlate with the electronegativity of peripheral functional groups, where electron-withdrawing moieties reduce the electron density around Fe centers, thereby increasing the effective valence state of isolated Fe atoms in FeSAC sites^28^. Complementary XAFS studies provided critical insights into the coordination geometry and local atomic environment. All R-FeSAC samples exhibited consistent fingerprint features in XANES spectra, indicating analogous coordination environments. A progressive increase in XANES edge energy was observed following the sequence F-FeSAC>H-FeSAC>NH_2_-FeSAC, consistent with the electronegativity-dependent elevation of Fe valence states (Fig. 2b–c). Fourier-transform EXAFS analysis (Fig. 2d) revealed a prominent Fe–N scattering path at ~1.60 Å without detectable Fe–Fe contributions (~2.2 Å), corroborating the atomic dispersion observed by XRD and AC-HAADF-STEM. Quantitative fitting of EXAFS spectra (Supplementary Fig. 6, Supplementary Table 2) yielded an average Fe–N coordination number of ~4 for all samples. Wavelet transform analysis (Fig. 2e–h and Supplementary Fig. 7) further confirmed Fe–N bonding through a characteristic intensity maximum at 3.60 Å^−1^ in *k*-space. These multimodal findings collectively demonstrate the precise engineering of FeSAC coordination structures with tunable electronic properties through functional group modulation.

### Electronic modulation of Fe centers for catalytic PI activation and mechanism elucidation

**Fig. 3:**
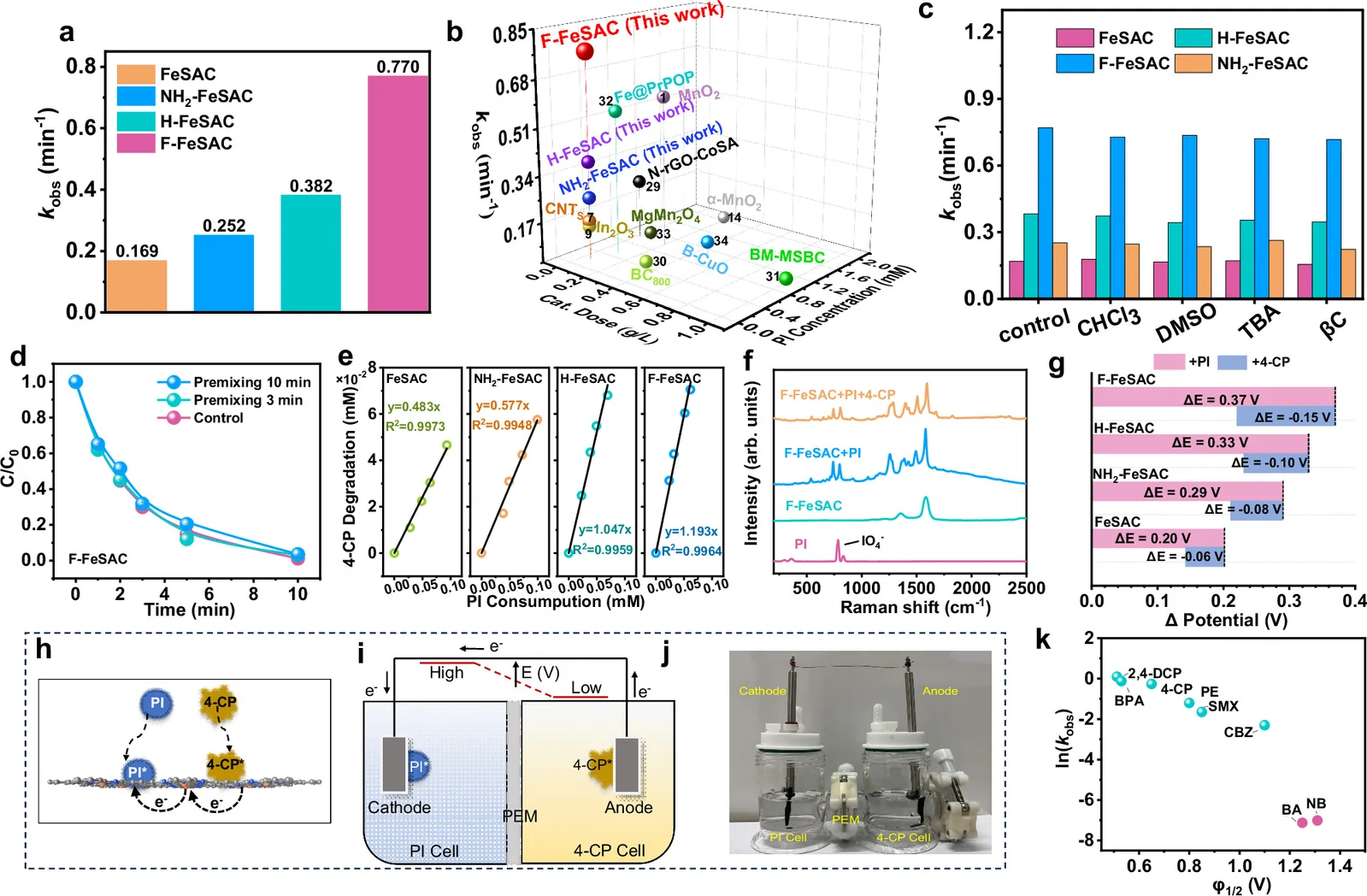
Peripheral substituents enhance FeSACs for periodate activation and mechanistic elucidation. **a** The pseudo-first-order rate constant (*k*_obs_) for 4-chlorophenol (4-CP) degradation in different catalytic systems. **b** Comparison of *k*_obs_ between the R-FeSAC/ periodate (PI) system and reported PI systems. The numbers denote the corresponding references. **c** The *k*_obs_ values of FeSAC/PI and R-FeSAC/PI systems in the presence of various quenchers. **d** Effect of premixing on 4-CP removal in the F-FeSAC/PI system. **e** Correlation between 4-CP degradation and PI consumption in FeSAC/PI and R-FeSAC/PI systems. Solid lines are linear fits through the origin (y$\,={\mbox{a}}x$, with *x* and *y* denoting PI consumption and 4-CP degradation, respectively). **f** In situ Raman spectroscopy of different systems. **g** Open-circuit potential (OCP) changes of different systems with the addition of PI and 4-CP. **h** Proposed electron-transfer pathway: electrons transfer from surface-bound 4-CP through F-FeSAC to surface-bound PI, driving 4-CP oxidation. Fe, C and N atoms are shown in orange, gray and blue, respectively; blue and yellow spheres represent PI and 4-CP, respectively, and asterisks denote surface-bound species. **i** Conceptual design and (**j**) photograph of the double-chamber reactor. The PI cathode and 4-CP anode chambers are separated by a proton-exchange membrane (PEM), with electrons flowing from the anode to the cathode through the external circuit. Each chamber had a working volume of 40 mL, and the graphite-felt electrode measured 3.0 × 2.0 × 0.20 cm. **k** Distribution of ln(*k*_obs_) as a function of half-wave potentials (*φ*_1/2_) for the eight contaminants: 4-chlorophenol (4-CP), bisphenol A (BPA), 2,4-dichlorophenol (2,4-DCP), phenol (PE), carbamazepine (CBZ), sulfamethoxazole (SMX), nitrobenzene (NB), and benzoic acid (BA). Routine conditions: [4-CP] = 80 μM, [catalyst] = 50 mg L^-1^, [PI] = 0.20 mM, temperature = 25 °C, without pH adjustment. Data are presented as mean values ± SD from three experiments (*n* = 3). Source data are provided as a Source Data file.

Systematic investigation of functionalized FeSAC catalysts in 4-chlorophenol (4-CP) degradation revealed critical structure-activity relationships governing PI activation through electronic modulation of iron centers. Control experiments demonstrated negligible 4-CP adsorption by the catalysts (<3% removal, Supplementary Fig. 8) and minimal oxidative capacity in PI-only systems, confirming the essential catalytic nature of the process. Notably, the pristine CNT support showed negligible PI activation under our experimental conditions (Supplementary Fig. 9), whereas all R-FeSAC catalysts markedly outperformed pristine FeSAC (Fig. 3a and Supplementary Fig. 10). This enhancement highlights the crucial role of functional substituents in higher coordination shells for optimizing intrinsic catalytic activity. Among these, F-FeSAC, featuring electron-deficient FeSAC sites due to its electron-withdrawing functional group, achieved a pseudo-first-order rate constant (*k*_obs_) of 0.770 min^-1^—approximately double that of H-FeSAC (0.382 min^−1^). Conversely, NH_2_-FeSAC, with an electron-donating functional group, exhibited a *k*_obs_ of 0.252 min^−1^, indicating slower reaction kinetics compared to H-FeSAC. The performance trends of F-FeSAC and NH_2_-FeSAC highlight the pivotal role of push-pull electronic effects induced by peripheral substituents, underscoring the importance of the Fe site electronic structure in driving catalytic PI activation. Moreover, the R-FeSAC/PI system demonstrated a significantly elevated *k*_obs_ value when considering both the catalyst loading and PI concentration, outperforming most reported PI-based AOPs^1,7,9,14,29–34^ (Fig. 3b and Supplementary Table 3), underscoring the efficiency of FeSACs for PI activation. To distinguish intrinsic site activity from apparent performance gains associated with dosage or oxidant concentration, the turnover frequency (TOF) was further evaluated and compared with previously reported coordination-regulated SAC systems. As shown in Supplementary Fig. 11 and Supplementary Table 4, F-FeSAC delivered a high TOF of 0.345 min^−1^, demonstrating that its superior activity arises from the enhanced reactivity of individual Fe sites.

To elucidate the enhancement mechanism of catalytic efficiency through electronic modulation in Fe sites with functional substituent architectures, we systematically investigated the degradation mechanisms of 4-CP in R-FeSAC/PI systems. Radical scavenger assays coupled with electron paramagnetic resonance (EPR) spectroscopy were employed to identify reactive oxygen species (ROS). As illustrated in Fig. 3c and Supplementary Fig. 12a–d, tert-butanol (TBA) and chloroform (CHCl_3_) were used to probe the possible contributions of •OH and O_2_^•−^, respectively. Their addition caused only minor changes in reaction kinetics, with representative *k*_obs_ values for F-FeSAC/PI remaining comparable to that of the control system (0.72 min^−1^ for TBA, 0.73 min^−1^ for CHCl_3_, and 0.77 min^−1^ for the control). Complementary nitroblue tetrazolium (NBT) assays were conducted to further evaluate O_2_^•−^ involvement. As NBT specifically reacts with O_2_^•−^ to form monoformazan with a characteristic 530 nm absorption band^29^, the absence of this spectral signature in UV-vis analyses post-NBT addition (Supplementary Fig. 13a–d) confirms negligible O_2_^•−^ generation. Additional EPR spin-trapping experiments using DMPO were conducted to examine the possible formation of radical species in the R-FeSAC/PI systems. As shown in Supplementary Figs. 14 and 15, no detectable DMPO-O_2_^•−^ (α_N_ = 14.3 G, α_H_^β^ = 11.4 G, α_H_^γ^ = 1.2 G) or DMPO-IO_3_^•^ (α_H_ = 12.9 G, α_N_ = 14.2 G) signals were observed, excluding O_2_^•−^ and IO_3_^•^ as the dominant radical species in these systems. Although weak DMPO-•OH signals (α_N_ = α_H_ = 14.9 G) were detected, their spectral profiles and intensities remained essentially unchanged after 4-CP addition, suggesting that •OH was not directly involved in 4-CP oxidation (Supplementary Fig. 16). Therefore, these results further support that radical oxidation is not the dominant pathway for 4-CP degradation in these systems. Crucially, prior studies have indicated that IO_3_^•^ is incapable of oxidizing 2,4,6-trichlorophenol (2,4,6-TCP)^13^. As shown in Supplementary Fig. 17a–d, all FeSAC/PI and R-FeSAC/PI systems achieved efficient 2,4,6-TCP degradation, establishing reaction pathways independent of IO_3_^•^ mediation. Collectively, these results conclusively demonstrate that 4-CP elimination proceeds through non-radical mechanisms – potentially involving singlet oxygen (^1^O_2_), high-valent metal-oxo intermediates, or ETP – rather than conventional radical-driven processes.

^1^O_2_ involvement was further examined using β-carotene (βC) quenching and D_2_O solvent substitution. βC caused negligible kinetic inhibition, with the representative *k*_obs_ of F-FeSAC/PI changing from 0.77 to 0.72 min^−1^ (Fig. 3c and Supplementary Fig. 12a–d), while D_2_O substitution did not accelerate the reaction kinetics (0.77 versus 0.76 min^−1^; Supplementary Fig. 18). The potential generation of ^1^O_2_ was further investigated through the oxidation of 9,10-diphenylanthracene (DPA), a probe renowned for its high selectivity and rapid reaction kinetics with ^1^O_2_ (*k* = 1.3 × 10^6 ^M^−1^ s^−1^)^14,35^. As shown in Supplementary Fig. 19a–d, the absorbance at 378 nm, corresponding to the DPA probe, remained virtually unchanged throughout the reaction, indicating negligible participation of ^1^O_2_ in the catalytic process. This conclusion was corroborated by EPR spectral analysis (Supplementary Fig. 20a–d), which revealed no detectable variation in TEMP–^1^O_2_ signals (α_N_ = 16 G) in the catalyst/PI systems, regardless of the presence or absence of 4-CP^36^. These findings collectively confirm that ^1^O_2_ plays an insignificant role in the catalytic degradation reactions.

Dimethyl sulfoxide (DMSO) was employed as a selective scavenger to probe the presence of high-valent iron-oxo species^37^. Remarkably, the addition of DMSO did not compromise the degradation efficiency in any of the four systems (Fig. 3c and Supplementary Fig. 12a–d). To further elucidate the potential involvement of high-valent metal-oxo species, methyl phenyl sulfoxide (PMSO) was utilized as a chemical probe, undergoing specific oxidation to yield methyl phenyl sulfone (PMSO_2_) *via* an oxygen atom transfer (OAT) mechanism^38^. However, quantitative analysis of PMSO and PMSO_2_ revealed no discernible consumption of PMSO or formation of PMSO_2_ across all systems (Supplementary Fig. 21a–d), unequivocally demonstrating the absence of high-valent metal-oxo complex oxidation in these catalytic reactions.

A premixing experiment was subsequently performed to gain deeper insight into the involvement of ETP in both the FeSAC/PI and R-FeSAC/PI systems. In this procedure, the catalysts and PI were premixed in solution, and 4-CP was introduced at predetermined intervals (3 and 10 min). Since the ETP mechanism entails electron flow between the catalysts (serving as the electronic bridge), oxidants (acting as electron acceptors), and pollutants (functioning as electron donors), it was anticipated that prior mixing of the catalysts and PI would exert minimal influence on pollutant degradation^39^. As depicted in Fig. 3d and Supplementary Fig. 22a–c, no discernible change in 4-CP removal was observed across varying premixing durations. Furthermore, the decomposition kinetics of IO_4_^−^ activated by R-FeSAC were quantified both in the presence and absence of 4-CP. As shown in Supplementary Fig. 23, IO_4_^−^ decomposition by R-FeSAC alone was inefficient, with only ~10% conversion. The persistent IO_4_^−^ concentration confirmed a limited direct reaction between IO_4_^−^ and the catalyst. In stark contrast, introducing 4-CP into the R-FeSAC/IO_4_^−^ system triggered rapid IO_4_^−^ consumption. A strong linear correlation between 4-CP removal and IO_4_^−^ depletion was also observed (Fig. 3e), indicating the concerted degradation of the pollutant and decomposition of the oxidant. The corresponding slope was highest for F-FeSAC, indicating the most efficient coupling between IO_4_^−^ consumption and 4-CP oxidation. This behavior is inconsistent with a conventional radical-dominated oxidation route and instead supports an electron-transfer-mediated process in which pollutant oxidation is coupled with oxidant consumption. To probe the interaction between F-FeSAC and PI, in situ Raman spectroscopy was conducted. As shown in Fig. 3f, introducing PI to F-FeSAC generated a new vibrational feature at ~830 cm^−1^, indicative of specific adsorption or coordination. Furthermore, the addition of 4-CP attenuated the PI-related Raman signal, suggesting that surface-associated PI species were consumed during 4-CP oxidation^7^. These findings provide further evidence that electron transfer is the dominant oxidation pathway driving the degradation of 4-CP.

### Electrochemical evidence supporting the electron-transfer pathway

To elucidate the reaction mechanism, electrochemical analyses were conducted employing current-time (*i*-*t*) measurements, open-circuit potential (OCP) measurements, and electrochemical impedance spectroscopy (EIS). The *i*-*t* responses were used to probe the interfacial electrochemical behavior associated with PI activation on different catalysts. A marked alteration in current response was observed for all catalysts upon the sequential introduction of PI and 4-CP (Supplementary Fig. 24), signifying the formation of catalyst-PI* complexes and the subsequent initiation of the ETP reaction. In particular, F-FeSAC exhibited a more pronounced current jump compared to other catalysts, suggesting enhanced electron transfer efficiency between F-FeSAC and PI. The LSV results further strengthen the electrochemical evidence for the ETP mechanism in the R-FeSAC/PI system. As shown in Supplementary Fig. 25, the addition of PI produces a distinct current response on the catalyst-modified electrodes, indicating the formation of a more electrochemically active catalyst/PI interfacial state. After subsequent addition of 4-CP, the current response changes further, suggesting that 4-CP participates in the interfacial charge-transfer process. Notably, F-FeSAC exhibits the strongest electrochemical response among all tested catalysts, indicating more favorable PI activation and enhanced interfacial electron-transfer capability. In contrast, H-FeSAC, NH_2_-FeSAC, and FeSAC display weaker responses, consistent with their lower catalytic activities. Chronopotentiometric measurements revealed shifts in OCP, indicating interfacial charge redistribution associated with electron-transfer behavior in these catalytic systems. As illustrated in Fig. 3g, the potential of electrodes coated with different catalysts increased following the addition of PI, indicating the formation of active catalyst/PI* complexes on the electrode surface, which elevated the redox potential and facilitated contaminant oxidation. In contrast, the introduction of 4-CP led to a potential decline, consistent with electron donation from 4-CP to the surface catalyst/PI complexes, followed by their consumption during the oxidation process (Fig. 3h). Notably, the F-FeSAC catalyst exhibited a more significant potential decrease compared to H-FeSAC and NH_2_-FeSAC upon 4-CP addition (Fig. 3g), suggesting that doping with electron-withdrawing groups enhances electron transfer efficiency. These findings were further corroborated by EIS measurements, where F-FeSAC exhibited lower charge transfer resistance compared to H-FeSAC, NH_2_-FeSAC, and FeSAC (Supplementary Fig. 26), underscoring the superior electron transport capacity of the catalysts, which can be attributed to the long-range modulation facilitated by electron-withdrawing group doping.

To further substantiate the ETP mechanism, a galvanic oxidation process (GOP) was implemented using a dual-chamber electrochemical system featuring catalyst-doped graphite felt (GF) electrodes (Fig. 3i-j). A proton exchange membrane (PEM) effectively isolated the 4-CP containment chamber from the PI chamber, thereby preventing molecular crossover between PI and 4-CP. This configuration facilitated exclusive redox coupling between PI and 4-CP through the external circuit, ensuring 4-CP oxidation occurred strictly *via* the ETP pathway^40^. Real-time monitoring of both current response and 4-CP degradation revealed significant performance variations among catalyst systems. Notably, the F-FeSAC-based GOP system exhibited exceptional catalytic activity, generating an instantaneous current density surpassing those of H-FeSAC, NH_2_-FeSAC, and pristine FeSAC systems by substantial margins (Supplementary Fig. 27). Correspondingly, the F-FeSAC catalyst achieved 52% 4-CP oxidation within 24 h, while control experiments with unmodified GF showed negligible degradation under identical conditions (Supplementary Fig. 28). These results conclusively demonstrate the ETP-mediated oxidation mechanism, with experimental evidence supporting the critical role of catalyst/PI* complex formation in facilitating direct electron transfer from 4-CP to PI.

### Relationship between degradation performances and properties of pollutants

To systematically evaluate the catalytic performance of the F-FeSAC/PI system, degradation experiments were conducted using eight representative contaminants: 4-CP, bisphenol A (BPA), 2,4-dichlorophenol (2,4-DCP), phenol (PE), carbamazepine (CBZ), sulfamethoxazole (SMX), nitrobenzene (NB), and benzoic acid (BA). To assess whether substrate redox accessibility contributes to the observed contaminant-dependent reactivity^40^, the *φ*_1/2_ of these eight contaminants were measured by cyclic voltammetry and used as experimental descriptors of their electron-donating propensity^38,41^. In the F-FeSAC/PI system, pollutants with relatively lower *φ*_1/2_ values, including 2,4-DCP, PE, CBZ, BPA, 4-CP, and SMX, generally exhibited higher degradation rates, whereas BA and NB showed higher *φ*_1/2_ values and much lower ln(*k*_obs_) values (Fig. 3k). This behavior is consistent with the presence of electron-withdrawing –COOH and –NO_2_ groups, which decrease the electron density of the aromatic ring and render oxidation less favorable (Supplementary Fig. 29). These results indicate that pollutant oxidation potential is closely associated with interfacial electron-transfer accessibility in the F-FeSAC/PI system, with electron-rich substrates being more favorable for ETP-mediated oxidation than electron-deficient substrates.

### Mechanistic insights into the electron transfer pathway in R-FeSAC/PI composites

**Fig. 4:**
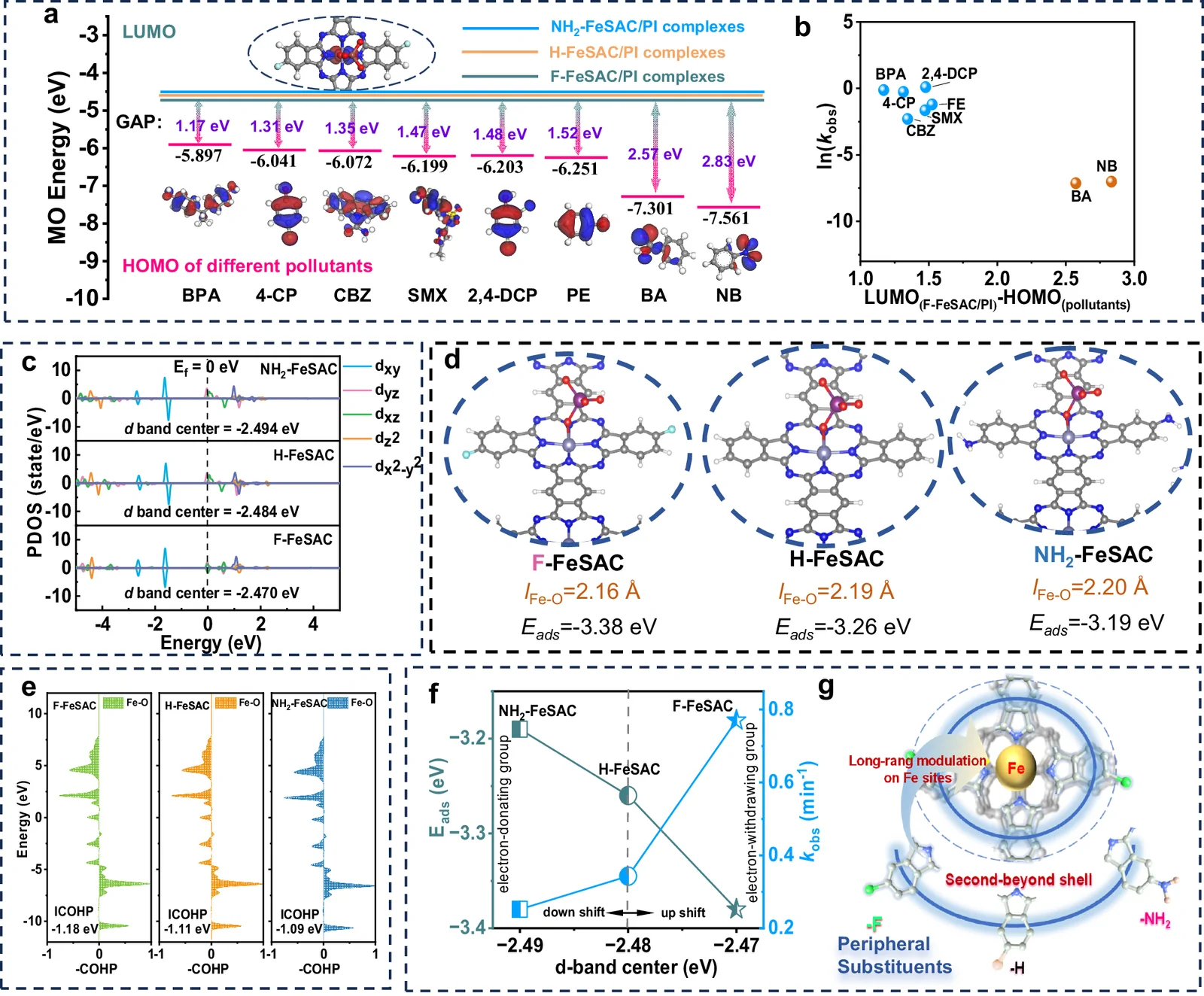
Mechanistic role of peripheral substituents beyond the second coordination sphere in regulating Fe active sites. **a** Energy gap between lowest unoccupied molecular orbital (LUMO) of R-FeSAC/PI and highest occupied molecular orbital (HOMO) of pollutants. C, H, N, O, F, Cl, S, Fe and I atoms are shown in gray, white, blue, red, cyan, green, yellow, lavender and purple, respectively. Red and blue isosurfaces denote opposite phases of the molecular orbitals. Crystal structures visualized using VESTA software^48^. **b** Relationship between LUMO_(R-FeSAC/PI)_-HOMO_(pollutants)_ and ln(*k*_obs_). **c** Projected Density of States (PDOS) of the Fe 3*d* orbitals in F-FeSAC, H-FeSAC, and NH_2_-FeSAC. E_f_ denotes the Fermi level. **d** Optimized adsorption configurations of PI at the Fe site on the F-FeSAC, H-FeSAC, and NH_2_-FeSAC. C, H, N, O, F, Fe and I atoms are shown in gray, white, blue, red, cyan, lavender and purple, respectively. Crystal structures visualized using VESTA software^48^. **e** Crystal orbital Hamilton population (COHP) analysis of Fe–O when PI adsorbs on Fe. **f** Correlations among *k*_obs_, adsorption energy (*E*_ads_), and *d*-band center for F-FeSAC, H-FeSAC, and NH_2_-FeSAC. **g** Schematic illustration of long-range electronic modulation at Fe sites induced by substituent groups beyond the secondary coordination sphere. Fe, C, N, F and H atoms are shown in gold, light gray, blue, green and pale pink, respectively. Crystal structures visualized using VESTA software^48^. Source data are provided as a Source Data file.

To construct Fe centers with beyond-second-coordination-shell substituents, we introduced peripheral substituents around the Fe-N_4_ moiety. Although these substituents are not directly coordinated to the Fe center, they remain electronically coupled to the Fe-N_4_ core through long-range electronic interactions. The optimized density functional theory (DFT) structures show that the substituent groups are positioned beyond the second coordination sphere, with a distance of >7.5 Å from the Fe-N_4_ core (Supplementary Fig. 30). Based on this structurally well-defined model, we further used DFT calculations to elucidate the electron-transfer behavior between diverse pollutants and FeSACs integrated with PI binding sites (denoted as R-FeSAC/PI, where R represents the functional substituents). Specifically, the molecular orbital (MO) energy levels of the R-FeSAC/PI composites and eight representative pollutants were systematically analyzed to clarify how peripheral substituents beyond the second coordination sphere influence interfacial electron-transfer behavior. According to the established ETP oxidation principle, effective electron transfer requires electron donation from the highest occupied molecular orbital (HOMO) of the pollutant to the lowest unoccupied molecular orbital (LUMO) of the R-FeSAC/PI composite^21,42^. As depicted in Fig. 4a, the HOMO energies of the pollutants span −7.56 to −5.90 eV, whereas the LUMO energies of the R-FeSAC/PI composites lie between −4.73 and −4.51 eV. This frontier-orbital energy offset supports the energetic accessibility of electron transfer from the HOMO of the pollutants (HOMO_pollutant_) to the LUMO of the catalyst (LUMO_R-FeSAC/PI_). Notably, the LUMO energy level of the fluorine-functionalized composite (F-FeSAC/PI, −4.73 eV) exhibits a pronounced downward shift relative to those of the hydrogen-functionalized (H-FeSAC/PI, −4.60 eV) and amino-functionalized (NH_2_-FeSAC/PI, −4.51 eV) counterparts. This reduced energy gap between HOMO_pollutant_ and LUMO_R-FeSAC/PI_, particularly for F-FeSAC/PI, facilitates interfacial electron transfer, thereby enhancing the kinetics of the ETP oxidation process.

To further clarify the substrate-dependent ETP reactivity, the LUMO_(R-FeSAC/PI)_–HOMO_(pollutant)_ energy gaps were compared with the observed ln(*k*_obs_) values for the same eight contaminants (Fig. 4b). Pollutants with smaller energy gaps generally showed higher degradation rates, indicating that closer frontier-orbital alignment facilitates electron donation from the pollutant to the R-FeSAC/PI complex^42^. In contrast, BA and NB were located in the large-gap/low-reactivity region, consistent with their high *φ*_1/2_ values and weak electron-donating character. Overall, higher pollutant HOMO levels and smaller catalyst/PI–pollutant energy gaps favor interfacial electron transfer, while electron-deficient pollutants with unfavorable orbital alignment show limited ETP accessibility.

To probe the influence of beyond-second-coordination-shell functional substituents on the underlying electron transfer mechanisms, we investigated the electronic structure regulation of the Fe active sites and its correlation with PI activation performance. Charge density difference analysis shows interfacial electron redistribution between the Fe sites and adsorbed PI species (Supplementary Fig. 31). The number of electrons transferred from F-FeSAC to PI was 0.843 e, slightly higher than that in H-FeSAC (0.841 e) and NH_2_-FeSAC (0.829 e). The calculated Fe *d*-band centers of F-FeSAC, H-FeSAC, and NH_2_-FeSAC are −2.470, −2.484, and −2.494 eV, respectively (Fig. 4c). Although the differences are small, this trend is consistent with a subtle perturbation of the Fe 3*d* electronic structure induced by peripheral substitution, which is further reflected in the orbital-resolved DOS near the Fermi level. In particular, the *d*z² and *d*_xz_/*d*_yz_ components, which are closely related to the subsequent Fe–O interfacial interaction with adsorbed PI, show clear substituent-dependent differences. Compared with H-FeSAC and NH_2_-FeSAC, F-FeSAC exhibits more pronounced Fe 3*d* contributions in the energy region relevant to PI coordination, particularly for orbitals capable of coupling with the O-centered states of PI. This orbital arrangement provides an electronic basis for the stronger Fe–O interaction formed upon PI adsorption.

We then analyzed the PI-adsorbed systems to directly probe the Fe–O interaction. The PDOS results show evident overlap between Fe-*d* and O-*p* states around the Fermi level after PI adsorption, indicating appreciable Fe–O orbital hybridization (Supplementary Fig. 32). This is further supported by the COHP/ICOHP analysis (Fig. 4e). Among the three systems, F-FeSAC-PI exhibits the shortest Fe–O bond length (2.16 Å), compared with 2.19 Å for H-FeSAC-PI and 2.20 Å for NH_2_-FeSAC-PI. Consistently, the ICOHP value of F-FeSAC-PI is the most negative (−1.180 eV), compared with −1.112 eV for H-FeSAC-PI and −1.094 eV for NH_2_-FeSAC-PI, indicating the strongest Fe–O bonding interaction in the F-substituted system (Fig. 4d-e). In addition, the -COHP profile of F-FeSAC-PI shows weaker occupied antibonding contribution near the Fermi level than those of the H- and NH_2_-substituted counterparts, in line with its stronger Fe–O bond. The adsorption energy (*E*_ads_) follows the same trend, with values of –3.38 eV for F-FeSAC, –3.26 eV for H-FeSAC, and –3.19 eV for NH_2_-FeSAC (Fig. 4d and Supplementary Fig. 33), supporting stronger PI binding on F-FeSAC.

Overall, these results demonstrate that beyond-second-coordination-shell substituents remotely regulate the electronic structure of Fe single-atom sites and thereby govern PI adsorption and activation (Fig. 4f–g). Specifically, the electron-withdrawing F substituent reconfigures the Fe-site electronic microenvironment, reshapes the frontier 3*d* orbital distribution near the Fermi level, and strengthens Fe–O orbital hybridization. Consequently, F-FeSAC exhibits the shortest Fe–O bond, the most negative ICOHP value, the weakest occupied antibonding contribution, and the strongest PI adsorption among the three catalysts. This electronically and structurally reinforced Fe–O interaction facilitates catalyst/PI* intermediate formation and promotes interfacial electron transfer, ultimately accounting for the superior PI activation and pollutant oxidation performance of F-FeSAC.

### Polymerization-driven pollutant removal mechanisms and practical application

**Fig. 5:**
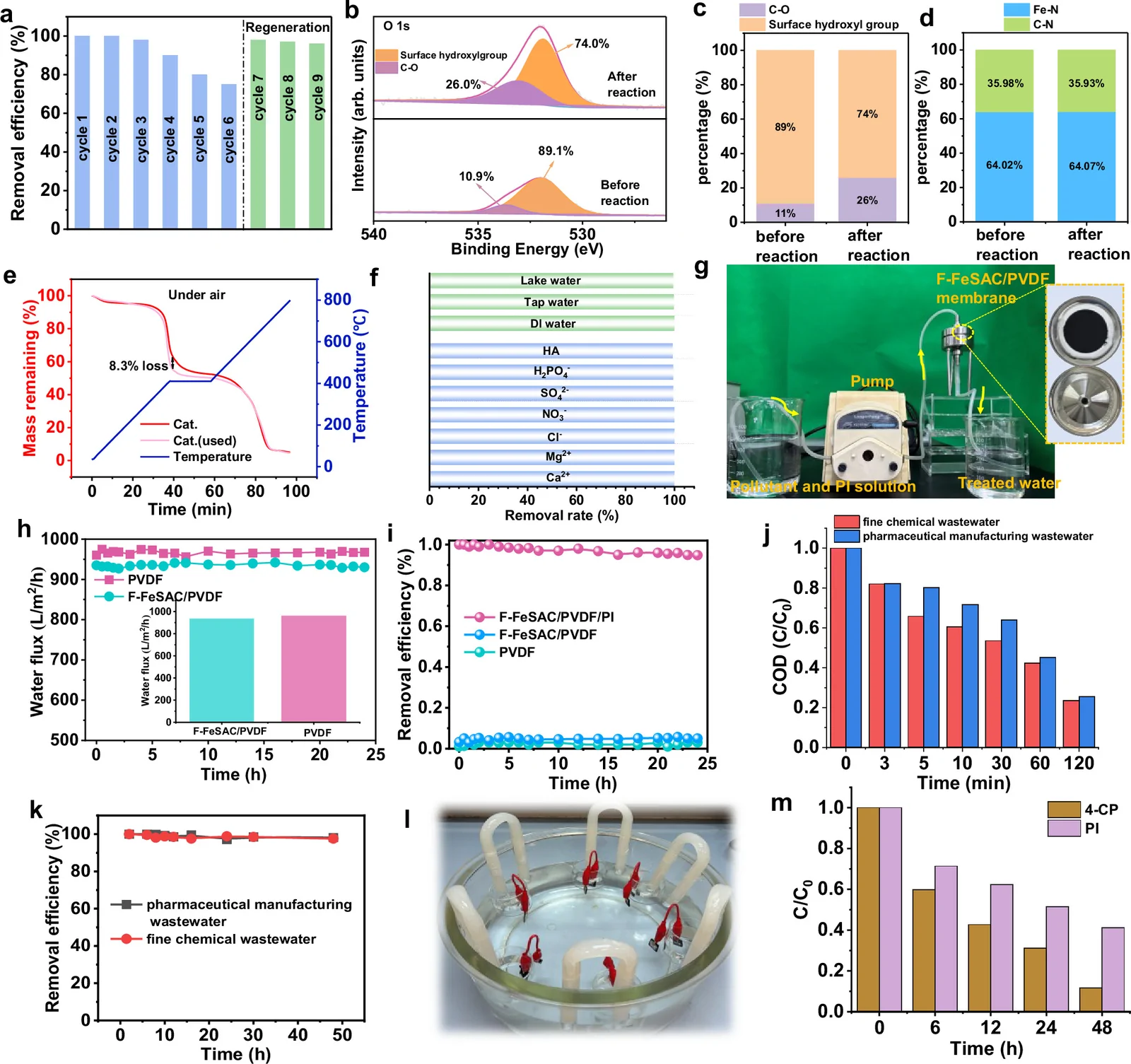
Polymerization-driven pollutant removal mechanisms and practical application. **a** Cycling stability of 4-chlorophenol (4-CP) degradation and active-site regeneration of F-FeSAC after six consecutive cycles. **b** XPS O 1*s* spectra of F-FeSAC before and after reaction. **c** Deconvoluted components of the XPS O 1*s* spectra for F-FeSAC before and after reaction. **d** XPS N 1*s* components of F-FeSAC before and after reaction. **e** TGA curves of F-FeSAC before and after the reaction. **f** Effects of various ionic species and water matrices on 4-CP degradation. **g** Photographs of the continuous-flow experiment in operation. The membrane had an effective filtration area of 13 cm^2^, and the feed reservoir had a working volume of 50 mL. **h** Water flux of different membranes during the continuous-flow process (insets show average flux values). **i** 4-CP removal efficiency of different systems. **j** Chemical oxygen demand (COD) removal in two real wastewater treatment plant (WWTP) scenarios. **k** 4-CP removal in the continuous-flow experiment for two real WWTP scenarios. **l** Photograph of the 3 L pilot-scale electron-transfer reactor comprising six PI chambers; the electrodes were externally connected to enable electron transfer without direct mixing. **m** 4-CP removal efficiency and PI consumption in the scaled-up ETP reactor. Routine conditions: [4-CP] = 80 μM, [catalyst] = 50 mg L^−1^, [PI] = 0.20 mM, temperature = 25 °C, without pH adjustment. Source data are provided as a Source Data file.

To assess the stability and practical applicability of the F-FeSAC/PI system, its catalytic performance was rigorously evaluated under prolonged operation and complex environmental conditions. As demonstrated in Fig. 5a, the F-FeSAC/PI system maintained a high 4-CP degradation efficiency of ~95% over four consecutive cycles. However, a progressive decline in performance was observed thereafter, with efficiency dropping to 78% by the sixth cycle. This attenuation is attributed to the accumulation of polymeric oxidation products on the catalyst surface, which partially blocks active sites and impedes reactant access. To counteract deactivation and demonstrate regenerability, the spent F-FeSAC catalyst was subjected to methanol washing assisted by ultrasonication. This regeneration protocol largely restored the catalytic activity, indicating that the deactivation was mainly associated with surface fouling rather than irreversible catalyst deterioration (Fig. 5a).

Characterization of the accumulated surface species provided mechanistic insight. Liquid chromatography-mass spectrometry (LC-MS) analysis of the solution following desorption identified chlorinated oligomers as the main surface-adsorbed products formed during 4-CP oxidation (Supplementary Figs. 34–35). Their formation supports a polymerization-mediated electron-transfer pathway. Specifically, 4-CP is first oxidized through interfacial electron transfer to generate phenoxy- or phenoxonium-type intermediates. These intermediates are resonance-stabilized over the oxygen atom and the ortho/para positions of the aromatic ring, making them prone to intermolecular C–C and C–O coupling. Repeated coupling of these surface-generated intermediates then leads to chlorinated dimers, trimers, and higher oligomeric products. Owing to their low solubility and strong interfacial affinity, these oligomeric products preferentially accumulate on the catalyst surface, leading to progressive activity attenuation during cycling. Further corroborating evidence for organic deposition was obtained through surface and bulk analysis. XPS analysis of the O 1*s* region revealed a distinct shift in surface oxygen species, transitioning from hydroxyl groups (O–H) to C–O functionalities post-reaction (Fig. 5b–c), indicative of adsorbed carbonaceous matter. Critically, the coordination environment of the single-atom Fe sites remained intact throughout the reaction, as evidenced by negligible alterations in the XPS N 1*s* spectra (Fig. 5d and Supplementary Fig. 36). Post-reaction XPS F 1*s* and solid-state ^19^F NMR analyses further showed that the fluorinated substituents were retained after catalysis (Supplementary Fig. 37). The spent F-FeSAC still exhibited a distinct F 1*s* signal at 687.3 eV, characteristic of covalent C–F species, while the ^19^F NMR spectrum remained consistent with that of the fresh catalyst. These results, together with the unchanged Fe–N coordination environment, indicate that the observed activity attenuation mainly arose from physical site blocking by deposited oligomeric species rather than catalyst degradation. Consistent with this interpretation, thermogravimetric analysis (TGA) quantified substantial organic deposition on the catalyst surface, revealing an 8.3% weight loss during programmed pyrolysis relative to pristine F-FeSAC (Fig. 5e). This mass loss corresponds directly to the thermal decomposition of accumulated chlorinated oligomers^43^.

Considering the complexity of real-world wastewater matrices, we systematically investigated the impact of common ionic constituents (Ca^2+^, Mg^2+^, Cl^−^, NO_3_^−^, SO_4_^2−^, H_2_PO_4_^−^) and natural organic matter (humic acid, HA) on the degradation efficacy. Remarkably, the presence of these potential interferents exerted negligible influence on 4-CP removal by the F-FeSAC/PI system (Fig. 5f). This robust anti-interference capability is likely a hallmark of the system’s dominant non-radical oxidation pathway, which is inherently less susceptible to quenching by background solutes compared to radical-based processes. Furthermore, the system demonstrated exceptional pH adaptability, sustaining high degradation efficiencies across a broad initial pH range from 2.5 to 9.8 (Supplementary Fig. 38). This wide operational window underscores the stability of the active sites under varying protonation conditions. Critically, when challenged with complex, real aqueous samples, the F-FeSAC/PI system consistently delivered superior 4-CP degradation performance (Fig. 5f). These results collectively underscore the robustness and versatility of the F-FeSAC catalyst, highlighting its strong potential for scalable and sustainable deployment in real-world wastewater treatment.

The integration of AOPs with polyvinylidene fluoride (PVDF) membranes represents a promising strategy for simultaneously mitigating organic pollutant permeation and enhancing membrane self-cleaning^44^. To explore this synergy, an F-FeSAC/PVDF composite membrane was meticulously fabricated *via* vacuum-assisted filtration, uniformly depositing an F-FeSAC dispersion onto a PVDF substrate. This engineered membrane was integrated into a continuous-flow wastewater treatment configuration (Fig. 5g). Performance evaluation revealed that the F-FeSAC/PVDF membrane achieved a stable water flux of 935.1 L/m^2^/h and a 4-CP rejection rate of 4.8%, closely comparable to those of pristine PVDF membranes (960.4 L/m^2^/h, 3.1%) (Fig. 5h–i). Crucially, when challenged with a synthetic influent containing 4-CP and PI under continuous operation, the membrane sustained a 4-CP removal efficiency exceeding 95% over 24 h (Fig. 5i). Long-term stability was also evidenced by minimal iron leaching, with Inductively coupled plasma mass spectrometry (ICP-MS) analysis confirming Fe concentrations consistently below 13.99 μg/L during extended operation (Supplementary Fig. 39). Structural integrity of the catalyst was further corroborated by post-reaction characterization (Supplementary Fig. 40). XRD and FTIR analyses of recycled F-FeSAC samples revealed no significant alterations, confirming the Fe single-atom coordination framework, precisely modulated by peripheral functional group substitution, remained intact after catalytic operation. To further strengthen the practical significance of the F-FeSAC/PI system, its performance was evaluated using representative industrial wastewater streams, including pharmaceutical manufacturing wastewater and fine chemical wastewater. These matrices typically exhibit high chemical oxygen demand (COD), complex organic profiles, intense coloration, and substantial inorganic loadings, posing well-recognized challenges to AOPs due to the coexistence of refractory aromatics, surfactants, chelating agents, and radical scavenging ions. Following 0.5 and 2 h of treatment, COD removal reached 50% and 78%, respectively (Fig. 5j). In two real wastewater treatment plant (WWTP) scenarios, the F-FeSAC/PVDF membrane maintained effective removal of 4-CP under continuous flow (Fig. 5k). Collectively, these results demonstrate the operational stability and reusability of the F-FeSAC/PVDF composite membrane and highlight its potential for sustainable deployment in advanced wastewater treatment systems.

To mitigate PI-derived secondary contamination, PI was confined to six independent chambers separated from the main reaction zone, enabling pollutant degradation *via* a salt-bridge-mediated electrotransformation pathway. A pilot-scale, 3 L enlarged electrotransformation reactor featuring electrodes connected to the six PI chambers was constructed (Fig. 5l), ensuring iodate species did not enter the effluent. Control tests showed that a bare electrode alone did not remove 4-CP (Supplementary Fig. 41), whereas the F-FeSAC based-ETP configuration achieved oxidation of >70% and >91% of 4-CP within 24 h and 48 h, respectively (Fig. 5m). Increasing electrode numbers further enhanced degradation (Supplementary Fig. 42). Concurrently, the PI electron acceptor was consumed in the dedicated chambers, with only ~40% remaining (Fig. 5m). Importantly, the enlarged ETP reactor achieved near-complete 4-CP removal in actual WWTP effluents within 60 h, while completely avoiding iodate contamination typical of conventional PI-activation systems (Supplementary Fig. 43).

### Analysis of generated iodine species and toxicity analysis

**Fig. 6:**
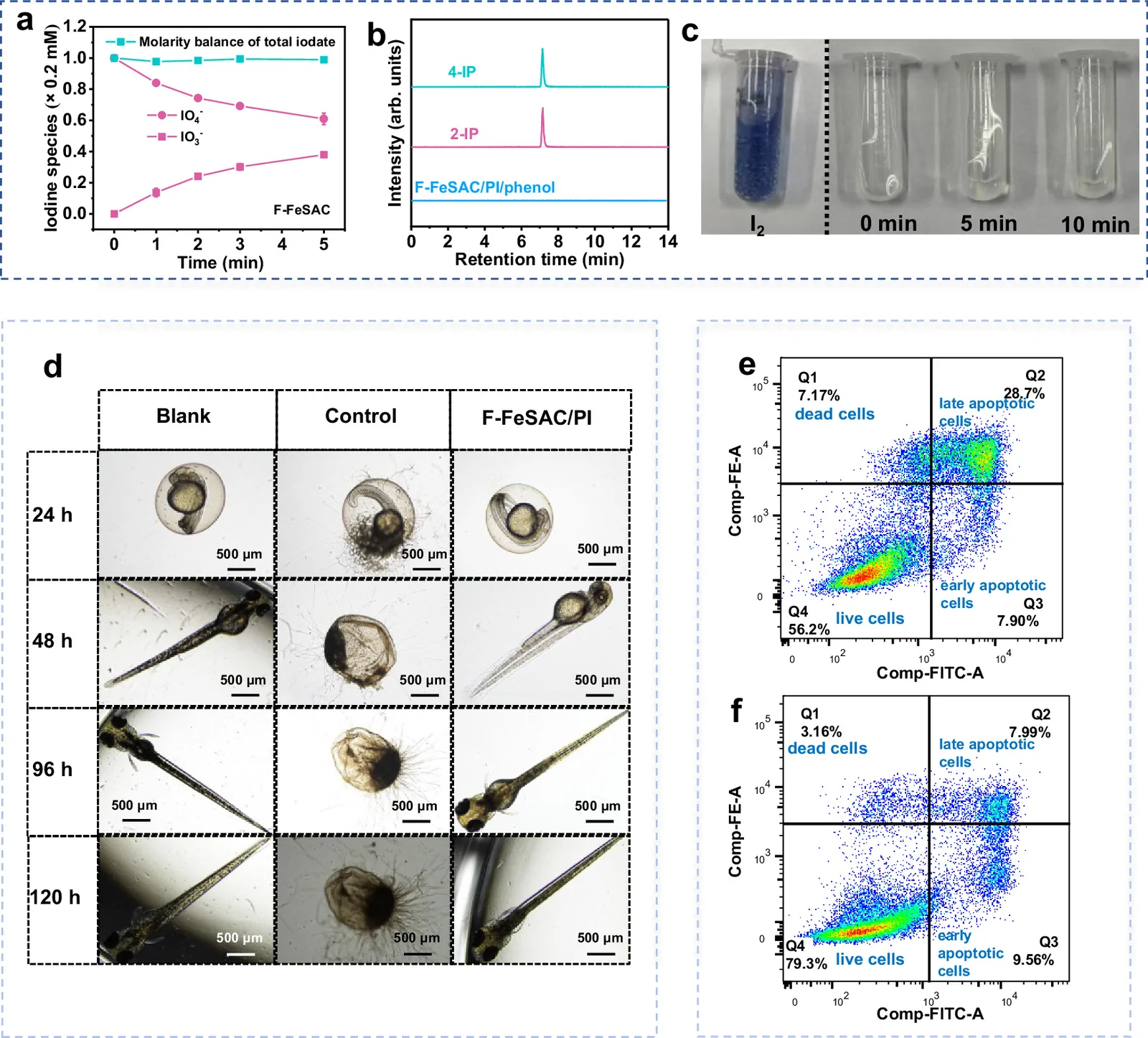
Analysis of generated iodine species and toxicity. **a** Concentration changes of periodate ion (IO_4_^‒^) and iodate ion (IO_3_^–^) in the F-FeSAC/PI/4-CP system. Data are presented as mean values ± SD from three experiments (*n* = 3). **b** Detection of hypoiodous acid (HOI) generated in the F-FeSAC/PI system using phenol as a trapping agent. **c** Detection of molecular iodine (I_2_) and triiodide (I_3_^‒^) in the F-FeSAC/PI system. **d** Images of zebrafish embryos under different treatments: nutrient medium only (Blank), 4-CP solution (Control), and post-reaction 4-CP solution from the F-FeSAC/PI system. Images are representative of three independent experiments with similar results. Flow cytometry analysis of *E. coli* cells exposed to reaction solutions collected at different reaction times: **e** 0 min, **f** 40 min. The four sections in flow cytometry images represent as below: Q1 dead cells; Q2 late apoptotic cells; Q3 early apoptotic cells; Q4 live cells. Source data are provided as a Source Data file.

The possible formation of reactive iodine intermediates, including hypoiodous acid (HOI), iodide (I^–^), and triiodide (I_3_^–^), raises serious environmental concerns in PI-based AOPs. These species may yield toxic iodinated disinfection byproducts that threaten both ecosystems and human health^45^. In contrast, iodate ion (IO_3_^–^) represents a desirable terminal product owing to its chemical stability and low environmental reactivity, rendering it an environmentally benign endpoint for iodine conversion^46^. To evaluate the behavior of IO_4_^–^ in the F-FeSAC catalytic system, its decomposition was systematically examined (Fig. 6a). During 4-CP degradation, IO_4_^–^ was predominantly converted to IO_3_^–^ without the appearance of any reactive iodine intermediates. The combined molar balance of IO_4_^–^ and IO_3_^–^ remained close to unity during the reaction, indicating high iodine recovery and confirming that consumed IO_4_^–^ was recovered mainly as IO_3_^–^. Given that HOI can iodinate phenolic compounds to form iodophenols^15^, such as 4-iodophenol (4-IP) and 2-iodophenol (2-IP), targeted LC-HRMS analysis of 4-IP and 2-IP was further performed. As shown in Fig. 6b, neither compound was detected, indicating no detectable HOI-mediated iodination under the tested conditions. Moreover, I^−^ was not detected by ion chromatography, and starch colorimetry showed no detectable response attributable to I_2_/I_3_^−^ accumulation in the F-FeSAC/PI process (Fig. 6c and Supplementary Fig. 44), corroborating the absence of reduced iodine species.

To further examine possible iodinated organic byproducts, high-resolution LC-MS suspect screening was performed for more than 200 theoretically possible iodinated compounds selected from plausible iodination products of 4-CP and its intermediates. None of the screened candidates met the assignment criteria for iodinated organic byproducts (Supplementary Figs. 45–48 and Supplementary Table 5). Collectively, these findings indicate that F-FeSAC effectively prevents PI reduction into environmentally risky iodine species, establishing the F-FeSAC/PI process as a sustainable and eco-safe AOP for organic contaminant oxidation.

The ecotoxicological safety of the treated effluent was further validated through zebrafish and microbial bioassays. Quantitative survival analysis showed that the untreated 4-CP solution caused clear survival inhibition during 120 h of exposure, whereas zebrafish exposed to the F-FeSAC/PI-treated solution maintained survival rates comparable to those of the blank group and significantly higher than those of the untreated 4-CP group (Supplementary Fig. 49 and Supplementary Table 6). Representative images further showed no obvious morphological abnormalities after exposure to the treated solution (Fig. 6d). These results indicate that F-FeSAC/PI treatment substantially attenuated the acute toxicity of the 4-CP-containing solution. Furthermore, flow cytometry analysis revealed that after 40 min of treatment, the proportion of viable microbial cells increased from 56.2% to 79.3%, while dead cells decreased from 7.17% to 3.16% (Fig. 6e–f), further confirming the strong detoxification efficiency of the F-FeSAC/PI system.

## Discussion

In this study, we engineered conjugated organic SACs (R-FeSAC) with atomically precise long-range coordination environments through peripheral organic group substitution, establishing their efficacy as model systems for PI activation. Crucially, electron-withdrawing fluorine substitution enhances the catalytic performance of F-FeSAC compared with H- and NH_2_-substituted analogs by remotely reconfiguring the Fe-site electronic microenvironment without altering the immediate Fe-N_4_ coordination shell. This remote regulation strengthens PI adsorption and enables a direct ETP for selective pollutant removal. Driven by the catalyst/PI complex LUMO and the pollutant HOMO energy levels, this ETP mechanism enables highly selective contaminant removal *via* polymerization, offering a sustainable alternative to conventional radical-based routes. The consistent trends among pollutant *φ*_1/2_, LUMO_(catalyst/PI)_–HOMO_(pollutant)_ energy alignment, and degradation reactivity indicate that pollutant electron-donating ability and frontier-orbital matching jointly govern electron-transfer accessibility and reaction selectivity. Furthermore, this F-FeSAC/PI system exclusively converts IO_4_^−^ to benign IO_3_^−^, effectively eliminating toxic iodine intermediates and byproducts, thus ensuring ecologically safe effluent as confirmed by zebrafish survival and enhanced microbial viability. The strategic manipulation of electronic properties through peripheral functional group substitution in higher coordination shells represents a transformative design paradigm for advancing SACs in AOPs.

## Methods

### Chemicals and reagents

4-fluorophthalonitrile (4-FPN), 1,2-dicyanobenzene (DCB), 1,2,4,5-tetracyanobenzene (BTC), ferric chloride (FeCl_3_), sodium periodate (NaIO_4_), 4-chlorophenol (4-CP) and the radical or reactive-species probes, were commercially obtained and used without further purification. All chemicals were of at least analytical grade, and deionized water was used throughout the experiments. Pharmaceutical manufacturing wastewater was obtained from Zhejiang Zhebei Pharmaceutical Co., Ltd., and fine chemical wastewater was collected from Shangyu District Water Treatment Development Co., Ltd. A complete list of chemicals and suppliers is provided in the Supplementary Methods.

### Synthetic strategy for F-FeSAC, NH_2_-FeSAC, H-FeSAC and FeSAC

The synthesis of F-FeSAC was performed *via* a single-step approach. Specifically, a combination of BTC (0.98 mmol), 4-FPN (0.42 mmol), and FeCl_3_ (0.085 mmol) was dispersed in a mixed solvent system comprising ethylene glycol and N,N-dimethylformamide (DMF), followed by ultrasonic treatment for 30 min. Subsequently, 1,8-diazabicyclo[5.4.0]undec-7-ene (DBU, 0.15 mmol) was introduced as a catalytic agent, and the reaction was maintained under a nitrogen atmosphere for 30 min. Afterward, 100 mg of carbon nanotubes (CNTs) were added to the solution, which was stirred continuously for 24 h. The mixture was subjected to heating at 180 °C for 72 h and allowed to cool gradually to ambient temperature. The resulting product was collected *via* centrifugation, washed sequentially with 1 M hydrochloric acid (HCl), absolute ethanol, and deionized water, and finally dried under vacuum at 70 °C for 48 h.

For comparative purposes, NH_2_-FeSAC and H-FeSAC were synthesized using the same synthetic procedure as described for F-FeSAC, except that 4-FPN was replaced with an equimolar amount of 4-aminophthalonitrile (4-APN) and phthalonitrile (PN), respectively. For the preparation of FeSAC, only BTC was employed as the monomer while omitting other phthalonitrile derivatives. All subsequent processing steps remained consistent with the aforementioned protocol.

### Characterization

Powder X-ray diffraction (XRD) data were collected on a PANalytical Empyrean using Cu Kα radiation (λ = 0.1541 nm). Fourier-transform infrared (FTIR) spectra were acquired using a Thermo Scientific Nicolet iS20 spectrometer with KBr pellets. Surface chemical states were examined by X-ray photoelectron spectroscopy (XPS) using a PerkinElmer PHI 5000 C instrument equipped with a monochromatic Al Kα X-ray source operated at 200 W. Sample morphology was characterized using a field-emission scanning electron microscope (FE-SEM; HITACHI Regulus 8100), while high-resolution transmission electron microscopy (HRTEM) imaging was performed on an FEI Talos F200S microscope. High-angle annular dark-field scanning transmission electron microscopy (HAADF-STEM) images were acquired using an FEI Titan Themis 60-300 TEM/STEM. The bulk Fe content was determined by inductively coupled plasma optical emission spectroscopy (ICP-OES; Agilent 720ES), whereas leached Fe was quantified by inductively coupled plasma mass spectrometry (ICP-MS; Agilent 7700). Fe *K*-edge X-ray absorption fine structure spectra were measured at beamline BL44B2 of the SPring-8 synchrotron facility in Japan. The storage ring was operated at 8.0 GeV with a maximum current of 250 mA. A Si (111) double-crystal monochromator was used, and the spectra were collected in transmission mode under ambient conditions using ionization chambers. Electron paramagnetic resonance (EPR) spectra were recorded using a Bruker A300 spectrometer. Specific surface areas and pore-size distributions were evaluated from N_2_ adsorption-desorption measurements using the Brunauer-Emmett-Teller method (Micromeritics ASAP 2460).

### Experimental procedure and analytical methods

The degradation of 4-CP, serving as a model pollutant, was investigated under controlled conditions of pH 6.0 and 25 °C, which were employed as standard parameters unless specified otherwise. The experimental setup included a 100 mL beaker containing an 80 μM solution of 4-CP and a catalyst dose of 50 mg L^−1^, stirred at a consistent rate of 700 rpm using a magnetic stirrer to ensure homogeneous mixing. The reaction was initiated by introducing 0.20 mM PI, which acted as the oxidizing agent. At predetermined intervals, aliquots of 1 mL were extracted from the reaction system, filtered through 0.22 μm water-phase membranes to eliminate residual catalyst, and subsequently quenched with sodium thiosulfate (Na_2_S_2_O_3_) to terminate the reaction. To investigate pH-dependent reaction kinetics, the initial solution pH was precisely regulated using 1 M HCl or NaOH prior to experimentation. Data are presented as mean values ± SD from three experiments (*n* = 3).

### Continuous-flow experiment for water treatment

F-FeSAC (50 mg) was dispersed in deionized water by sonication and immobilized on a polyvinylidene fluoride (PVDF) membrane through vacuum-assisted filtration to prepare an F-FeSAC/PVDF composite membrane. The resulting membrane was installed in a flow-through reactor, into which an influent containing 80 μM 4-CP and 0.20 mM PI was continuously supplied using a peristaltic pump. Effluent samples were collected, filtered and analyzed to determine the residual 4-CP concentration.

### Quantification of organic contaminants

Target contaminants were quantified using a Waters e2695 high-performance liquid chromatography (HPLC) system equipped with a UV detector and a C18 column (4.6 × 150 mm, 5 μm). A methanol/water mixture (70:30, *v/v*) was used to separate bisphenol A (BPA), carbamazepine (CBZ), 2,4-dichlorophenol (2,4-DCP) and phenol (PE), which were monitored at 225, 280, 284 and 225 nm, respectively. Sulfamethoxazole (SMX) was detected at 265 nm using methanol/water (50:50, *v/v*). For 4-chlorophenol (4-CP) and benzoic acid (BA), methanol/water (60:40, *v/v*) was used, with detection wavelengths of 280 and 227 nm, respectively.

Contaminant removal efficiency (η) was calculated using Eq. (1):

1 $$\eta=\frac{{\rm{C}}_{0}-{{\rm{C}}}_{{{\rm{t}}}}}{{{{\rm{C}}}}_{0}}\times 100\%$$

Here, *C*_0_ and *C*_t_ denote the contaminant concentrations at the beginning of the reaction and at time t, respectively.

Degradation products were further analysed using an Agilent 1290 liquid chromatograph coupled to an Agilent 6460 triple-quadrupole tandem mass spectrometer. Additional quantification of target contaminants, methyl phenyl sulfoxide (PMSO) and methyl phenyl sulfone (PMSO_2_) was performed using an Agilent 1200 HPLC system equipped with a diode-array detector. Separation was achieved on a ZORBAX Eclipse XDB-C18 column (4.6 × 150 mm, 5 μm; Agilent). The mobile phases consisted of acetonitrile or methanol combined with an aqueous solution containing 0.1% (*v/v*) glacial acetic acid.

### Singlet-oxygen detection

The formation of singlet oxygen (^1^O_2_) was evaluated using 9,10-diphenylanthracene (DPA) as a chemical probe. DPA stock solution (100 μL, 1 g L^−1^ in DMSO) was first diluted with 1.8 mL of deionized water. An aliquot collected at the designated reaction time (1.6 mL) and methanol (0.8 mL) were subsequently added, with methanol used to quench further reactions. DPA consumption was monitored by UV-visible spectroscopy from the decrease in absorbance at 378 nm.

### Electrochemical measurements

Open-circuit potential (OCP), chronoamperometric and electrochemical impedance spectroscopy (EIS) measurements were performed using a CHI 730E electrochemical workstation (Shanghai Chenhua, China) in a three-electrode configuration comprising an Ag/AgCl reference electrode, a Pt counter electrode and an indium tin oxide (ITO) glass working electrode. The working electrode was prepared by sonicating 20 mg of catalyst with 300 μL of isopropanol and 50 μL of Nafion solution, coating the resulting suspension onto pretreated ITO glass and drying it in air at room temperature. For OCP measurements, PI and 4-CP were sequentially introduced into 0.10 M sodium sulfate electrolyte after a stable baseline had been established, and the corresponding potential changes were recorded.

Cyclic voltammetry (CV) was conducted over a potential range of −1.0 to +2.0 V at 10 mV s^-1^ in 20 mM phosphate-buffered solution (PBS) containing 50 μM pollutant. The same three-electrode configuration was used. The half-wave potential (*φ*_1/2_) was determined using Eq. (2):

2 $${\varphi }_{1/2}=1/2({{{\rm{E}}}}_{{{\rm{p}}}}+{{{\rm{E}}}}_{{{\rm{p}}}/2})$$

### Galvanic oxidation processes (GOP)

Commercial graphite felt (GF) was cut into pieces measuring 3.0 × 2.0 × 0.20 cm^3^, washed successively with acetone, ethanol and deionized water, and then dried at 60 °C for 10 h. Catalyst ink was prepared by sonicating 50 mg of catalyst in 5 mL of ethanol, 5 mL of deionized water and 50 μL of 5 wt% Nafion solution for 1 h. The resulting suspension was uniformly drop-cast onto the pretreated GF and dried at 40 °C for 6 h. Before use, the catalyst-coated electrodes were thoroughly rinsed with deionized water.

Galvanic oxidation experiments were performed in a sealed dual-chamber reactor separated by a proton-exchange membrane (PEM). The anodic and cathodic compartments contained 40 mL of 5 μM 4-CP and 40 mL of 2 mM PI, respectively. One R-FeSAC/GF electrode was placed in each compartment, and the two electrodes were externally connected using copper wires to establish a closed circuit. At predetermined time points, 1 mL aliquots were withdrawn from the anodic compartment, passed through a 0.22 μm membrane and analysed for 4-CP by HPLC.

### Iodinated byproducts detection

Hypoiodous acid (HOI) formation was assessed indirectly through phenol iodination, with 4-iodophenol (4-IP) and 2-iodophenol (2-IP) serving as diagnostic products. Phenol was added to the F-FeSAC/PI system at a final concentration of 1 mM before initiating the reaction.

Molecular iodine (I_2_) and triiodide (I_3_^-^) were detected by a starch-based colorimetric method^46^. Reaction aliquots and authentic I_2_ reference solutions were separately combined with freshly prepared starch solution, and formation of the characteristic blue starch-polyiodide complex was used as evidence for I_2_/I_3_^-^. To prepare the starch reagent, 3 g of soluble starch was dispersed in 10 mL of ultrapure water and rapidly transferred into 90 mL of boiling water under vigorous stirring. The mixture was stirred for an additional 3 min and allowed to cool to room temperature before use.

### DFT computational methods

Density functional theory calculations were carried out with the Vienna Ab initio Simulation Package (VASP) using the projector-augmented-wave (PAW) method. Exchange-correlation effects were treated using the Perdew-Burke-Ernzerhof (PBE) functional within the generalized-gradient approximation, while dispersion interactions were incorporated through the DFT-D3 correction. The kinetic-energy cutoff for the plane-wave basis was set to 400 eV. Brillouin-zone integration for the periodic catalyst models employed a 2 × 2 × 1 *k*-point mesh. Individual pollutant molecules were modeled in sufficiently large vacuum cells and sampled only at the Γ point. Structural relaxation was continued until the energy change and residual atomic forces were below 10^-4 ^eV and 0.02 eV Å^-1^, respectively. The highest occupied molecular orbital (HOMO) energies were obtained from the Kohn-Sham eigenvalues of the optimized isolated pollutant molecules. Values calculated under the same computational settings were compared to evaluate the relative electron-donating tendencies of the pollutants. The atomic coordinates of all optimized models are provided in Supplementary Data 1.

### Zebrafish embryo assay

Three healthy adult zebrafish, comprising one female and two males, were maintained together for 12 h to obtain fertilized embryos. The collected embryos were rinsed, examined microscopically and selected at the eight-cell stage for exposure experiments. Three exposure conditions were established: embryo medium alone (blank), untreated 4-CP solution (control) and post-reaction 4-CP solution from the F-FeSAC/PI system (treatment). For each independent experiment, 30 embryos were assigned to each exposure group and evenly distributed across three 24-well plates, with one embryo placed in each well. Each well contained 1 mL of embryo medium and 1 mL of the corresponding exposure solution. Embryonic development and survival were monitored microscopically, and the exposure medium was renewed every 24 h until hatching. Three independent experiments were conducted (*n* = 3).

The zebrafish experiments were approved by the Animal Experimentation Ethics Committee of Zhejiang University of Technology (approval no. 231005016; 15 March 2023), and all procedures complied with the national guideline *Laboratory Animal-Guideline for Ethical Review of Animal Welfare* (GB/T 35892-2018). Sex was considered during breeding but not during embryo allocation or analysis because it cannot be reliably determined at the eight-cell stage.

### Flow cytometry

Flow cytometry was performed on LIVE/DEAD-stained *Escherichia coli* (*E. coli*) to assess toxicity changes during 4-CP degradation in the F-FeSAC/PI system. The *E. coli* strain was obtained from the American Type Culture Collection (ATCC), and beef extract was purchased from Beijing Coolabor Biotechnology Co., Ltd. An aliquot of the bacterial stock suspension (10 μL) was inoculated into 10 mL of culture medium containing 3 g L^-1^ beef extract, 10 g L^-1^ tryptone and 5 g L^−1^ NaCl (pH 7.2), followed by incubation with shaking at 30 °C for 10 h. Cells were collected by centrifugation at 402 × *g* for 5 min and washed three times with PBS (0.5 M, pH 7.4).

The washed bacterial suspension (1 mL) was combined with 10 mL of reaction solution collected at either 0 or 40 min. After incubation for 9 h, the cells were recovered by centrifugation at 402 × *g* for 5 min, washed three times with PBS and adjusted to approximately 1 × 10^6^ cells mL^-1^. SYTO 9 and propidium iodide were added at final concentrations of 3.34 and 20 μM, respectively. Following staining for 15 min at room temperature in the dark, samples were analysed without an additional washing step. A PBS negative control was used to distinguish background signals and establish the primary analysis gate. Single-stained controls were used for compensation between the FL1 and FL3 channels, whereas cells heat-treated at 70 °C for 30 min served as the propidium-iodide-positive control. The same gating criteria were applied to the PBS control and water samples to directly compare bacterial population abundance and viability (Supplementary Fig. 50). Flow cytometric data were acquired and analyzed using a CytoFLEX LX flow cytometer (Beckman Coulter) with CytExpert software (version 2.5).

### Reporting summary

Further information on research design is available in the Nature Portfolio Reporting Summary linked to this article.

## Supplementary information

Supplementary Information

Peer Review File

Description of Additional Supplementary Files

Supplementary Data 1

Reporting Summary

## Source data

Source Data

## Data Availability

The data generated in this study are provided within the article and the Supplementary Information file. Additional data are available from the corresponding authors upon request. Source data are provided with this paper^47^.

## References

[1] Du, J. et al. Periodate activation with manganese oxides for sulfanilamide degradation. *Water Res.* **169**, 115278 (2020). 10.1016/j.watres.2019.115278 31731245

[2] Tang, Q. et al. Electron transfer mediated activation of periodate by contaminants to generate ^1^O_2_ by charge-confined single-atom catalyst. *Nat. Commun.* **15**, 9549 (2024). 10.1038/s41467-024-53941-8 PMC11538331 39500863

[3] Chen, L. et al. Solar-light-activated periodate for degradation and detoxification of highly toxic 6PPD-quinone at environmental levels. *Nat. Water* **2**, 453–463 (2024).

[4] Zong, Y. et al. Enhanced oxidation of organic contaminants by Iron(II)-activated periodate: The significance of high-valent iron–oxo species. *Environ. Sci. Technol.* **55**, 7634–7642 (2021). 10.1021/acs.est.1c00375 33706511

[5] Luo, M. et al. Electrochemical activation of periodate with graphite electrodes for water decontamination: excellent applicability and selective oxidation mechanism. *Water Res.* **240**, 120128 (2023). 10.1016/j.watres.2023.120128 37247436

[6] Yu, Y., Dong, H., Chen, T., Sun, Y. & Guan, X. Unraveling the intrinsic mechanism behind the selective oxidation of sulfonamide antibiotics in the Mn(II)/periodate process: The overlooked surface-mediated electron transfer process. *Water Res.* **244**, 120507 (2023). 10.1016/j.watres.2023.120507 37639991

[7] Peng, J. et al. Removal of phenols by highly active periodate on carbon nanotubes: A mechanistic investigation. *Environ. Sci. Technol.* **57**, 10804–10815 (2023). 10.1021/acs.est.2c08266 37431633

[8] Hamdaoui, O. & Merouani, S. Improvement of sonochemical degradation of Brilliant blue R in water using periodate ions: implication of iodine radicals in the oxidation process. *Ultrason. Sonochem.* **37**, 344–350 (2017). 10.1016/j.ultsonch.2017.01.025 28427642

[9] Yang, T. et al. Enhanced hydroxyl radical generation for micropollutant degradation in the In_2_O_3_/Vis-LED process through the addition of periodate. *Water Res.* **243**, 120401 (2023). 10.1016/j.watres.2023.120401 37536249

[10] Bokare, A. D. & Choi, W. Singlet-oxygen generation in alkaline periodate solution. *Environ. Sci. Technol.* **49**, 14392–14400 (2015). 10.1021/acs.est.5b04119 26594871

[11] Niu, L. et al. Ferrate(VI)/periodate system: synergistic and rapid oxidation of micropollutants via periodate/iodate-modulated Fe(IV)/Fe(V) intermediates. *Environ. Sci. Technol.* **57**, 7051–7062 (2023). 10.1021/acs.est.2c08965 37074844

[12] Choi, Y. et al. Activation of periodate by freezing for the degradation of aqueous organic pollutants. *Environ. Sci. Technol.* **52**, 5378–5385 (2018). 10.1021/acs.est.8b00281 29648451

[13] Lee, H. et al. Oxidizing capacity of periodate activated with iron-based bimetallic nanoparticles. *Environ. Sci. Technol.* **48**, 8086–8093 (2014). 10.1021/es5002902 24896837

[14] Yao, Y. et al. Phase-activity relationship of MnO_2_ nanomaterials in periodate oxidation for organic pollutant degradation. *Water Res.* **264**, 122224 (2024). 10.1016/j.watres.2024.122224 39153314

[15] Ling, C. et al. Sulfide-modified zero-valent iron activated periodate for sulfadiazine removal: Performance and dominant routine of reactive species production. *Water Res.* **220**, 118676 (2022). 10.1016/j.watres.2022.118676 35640509

[16] Chen, Y., Ge, X., Li, J., Zhang, Z. B. & Li, Z. Anchored atomic Ru-O_4_ architecture enables ultra-effective Fe(VI) activation via avoiding Fe(VI) self-decay for water purification. *Nat. Commun.* **16**, 7509 (2025). 10.1038/s41467-025-62930-4 PMC12350647 40804045

[17] Wu, Z. et al. Long-range interactions driving neighboring Fe-N_4_ sites in Fenton-like reactions for sustainable water decontamination. *Nat. Commun.* **15**, 7775 (2024). 10.1038/s41467-024-52074-2 PMC11377441 39237559

[18] Lin, Y. et al. Coordination engineering of heterogeneous high-valent Fe(IV)-oxo for safe removal of pollutants via powerful Fenton-like reactions. *Nat. Commun.* **15**, 10032 (2024). 10.1038/s41467-024-54225-x PMC11576887 39562564

[19] Liu, T. et al. Water decontamination via nonradical process by nanoconfined Fenton-like catalysts. *Nat. Commun.* **14**, 2881 (2023). 10.1038/s41467-023-38677-1 PMC10199022 37208339

[20] Shi, L. J. et al. Dual-substrate synergistic catalysis for highly efficient water purification. *Nat. Water* **3**, 345–353 (2025).

[21] Guo, J. et al. Fenton-like activity and pathway modulation via single-atom sites and pollutants comediates the electron transfer process. *Proc. Natl. Acad. Sci. USA* **121**, e2313387121 (2024). 10.1073/pnas.2313387121 PMC10801885 38190529

[22] Shang, Y., Xu, X., Gao, B., Wang, S. & Duan, X. Single-atom catalysis in advanced oxidation processes for environmental remediation. *Chem. Soc. Rev.* **50**, 5281–5322 (2021). 10.1039/d0cs01032d 33904567

[23] Zhu, Z.-S. et al. Multidimensional engineering of single-atom cobalt catalysts for ultrafast Fenton-like reactions. *Nat. Water* **3**, 211–221 (2025).

[24] Li, X. & Xiang, Z. Identifying the impact of the covalent-bonded carbon matrix to FeN_4_ sites for acidic oxygen reduction. *Nat. Commun.* **13**, 57 (2022). 10.1038/s41467-021-27735-1 PMC8748808 35013260

[25] Li, X. et al. An initial covalent organic polymer with closed-F edges directly for proton-exchange-membrane fuel cells. *Adv. Mater.* **34**, 2204570 (2022). 10.1002/adma.202204570 35863906

[26] Zhang, K. et al. Molecular modulation of sequestered copper sites for efficient electroreduction of carbon dioxide to methane. *Adv. Funct. Mater.* **33**, 2214062 (2023).

[27] Wu, H. et al. π-Conjugated polymeric phthalocyanine for the oxidative coupling of amines. *Chem. Commun.* **56**, 3637–3640 (2020). 10.1039/c9cc09422a 32105284

[28] Li, X., Chen, T., Yang, B. & Xiang, Z. Fundamental understanding of electronic structure in FeN_4_ site on electrocatalytic activity via dz^2^-orbital-driven charge tuning for acidic oxygen reduction. *Angew. Chem.* **135**, e202215441 (2023). 10.1002/anie.202215441 36625776

[29] Long, Y. et al. Atomically dispersed cobalt sites on graphene as efficient periodate activators for selective organic pollutant degradation. *Environ. Sci. Technol.* **55**, 5357–5370 (2021). 10.1021/acs.est.0c07794 33729757

[30] Dai, J. et al. Applying a novel advanced oxidation process of biochar activated periodate for the efficient degradation of bisphenol A: Two nonradical pathways. *Chem. Eng. J.* **453**, 139889 (2023).

[31] He, L. et al. Ball milling-assisted preparation of sludge biochar as a novel periodate activator for nonradical degradation of sulfamethoxazole: Insight into the mechanism of enhanced electron transfer. *Environ. Pollut.* **316**, 120620 (2023). 10.1016/j.envpol.2022.120620 36372368

[32] Shi, Z. et al. Fe confined in Porphyrin-based porous organic polymer as an efficient periodate activator for nonradical pathway removal of contaminants. *Sep. Purif. Technol.* **317**, 123868 (2023).

[33] Yu, J. et al. Periodate activation with stable MgMn_2_O_4_ spinel for bisphenol A removal: Radical and non-radical pathways. *Chem. Eng. J.* **459**, 141574 (2023).

[34] Zhang, X. et al. Activation of periodate with pinewood biochar-CuO composite for the removal of recalcitrant organic pollutants-Mechanisms and degradation products. *Chem. Eng. J.* **465**, 142916 (2023).

[35] Wang, Y., Lin, Y., He, S., Wu, S. & Yang, C. Singlet oxygen: properties, generation, detection, and environmental applications. *J. Hazard. Mater.* **461**, 132538 (2024). 10.1016/j.jhazmat.2023.132538 37734310

[36] Liu, B. et al. Defect-induced electric field effects direct Fenton-like oxidation pathways towards polymerization for sustainable water treatment. *Nat. Commun.* **16**, 10963 (2025). 10.1038/s41467-025-65966-8 PMC12690085 41365849

[37] Zhou, Y. et al. Insights into free radical and non-radical routes regulation for water cleanup. *Nat. Commun.* **16**, 7753 (2025). 10.1038/s41467-025-63235-2 PMC12368032 40835831

[38] Cheng, C. et al. Generation of Fe^IV^=O and its contribution to Fenton-like reactions on a single-atom iron−N−C catalyst. *Angew. Chem. Int. Ed.* **62**, e202218510 (2023). 10.1002/anie.202218510 36625681

[39] Ren, W. et al. Origins of electron-transfer regime in persulfate-based nonradical oxidation processes. *Environ. Sci. Technol.* **56**, 78–97 (2022). 10.1021/acs.est.1c05374 34932343

[40] Yu, Z. et al. Decoupled oxidation process enabled by atomically dispersed copper electrodes for in-situ chemical water treatment. *Nat. Commun.* **15**, 1186 (2024). 10.1038/s41467-024-45481-y PMC10853265 38332033

[41] Ren, W. et al. Activation of peroxydisulfate on carbon nanotubes: electron-transfer mechanism. *Environ. Sci. Technol.* **53**, 14595–14603 (2019). 10.1021/acs.est.9b05475 31721570

[42] Yin, K. et al. Pore modulation of single atomic Fe sites for ultrafast Fenton-like chemistry with amplified electron migration oxidation. *Water Res.* **268**, 122545 (2025). 10.1016/j.watres.2024.122545 39378749

[43] Liu, H. Z. et al. Tailoring *d*-band center of high-valent metal-oxo species for pollutant removal via complete polymerization. *Nat. Commun.* **15**, 2327 (2024). 10.1038/s41467-024-46739-1 PMC10940690 38485966

[44] Yang, Y. et al. Large-scale deployment of single-atom catalysts via cross-scale confinement in ceramic membranes for advanced water treatment. *Nat. Water* **3**, 1281–1290 (2025).

[45] Zong, Y. et al. Surface-mediated periodate activation by nano zero-valent iron for the enhanced abatement of organic contaminants. *J. Hazard. Mater.* **423**, 126991 (2022). 10.1016/j.jhazmat.2021.126991 34482081

[46] Sun, H., He, F. & Choi, W. Production of reactive oxygen species by the reaction of periodate and hydroxylamine for rapid removal of organic pollutants and waterborne bacteria. *Environ. Sci. Technol.* **54**, 6427–6437 (2020). 10.1021/acs.est.0c00817 32298086

[47] Jin, S. et al. Enzyme-mimicking peripheral substituents beyond the second coordination sphere tune single-atom catalysts for periodate activation. *Figshare*10.6084/m9.figshare.33033551 (2026). PMC13680503 42897163

[48] Momma, K. & Izumi, F. VESTA 3 for three-dimensional visualization of crystal, volumetric and morphology data. *J. Appl. Crystallogr.* **44**, 1272–1276 (2011).

