# Supplementary material for: Enzyme-mimicking peripheral substituents beyond the second coordination sphere tune single-atom catalysts for periodate activation
Source: Nat Commun. 2026 Sep 17;17:10926. doi: 10.1038/s41467-026-77805-5 (PMC13680503; doi:10.1038/s41467-026-77805-5)
Supplement: Supplementary file 1 [file 41467_2026_77805_MOESM1_ESM.pdf]

Supplementary Information

Enzyme-Mimicking Peripheral Substituents beyond the Second Coordination Sphere Tune Single-Atom Catalysts for Periodate Activation

Sijia Jin[1,2], Xiaofeng Tang[1,2], Zhiqiao He[2], Shuang Song[2], Yaqi Cai[1,3], Haiyan Zhang[1], Bo Lai*[4] and Tao Zeng*[1,2]*

[1]Zhejiang Key Laboratory of Environment and Health of New Pollutants, School of Environment, Hangzhou Institute for Advanced Study, University of Chinese Academy of Sciences, Hangzhou, 310024, P.R. China.

[2]Zhejiang Key Laboratory of Low-carbon Control Technology for Industrial Pollution, College of Environment, Zhejiang University of Technology, Hangzhou, Zhejiang, 310032, P.R. China.

[3]State Key Laboratory of Environmental Chemistry and Ecotoxicology, Research Center for Eco-Environmental Sciences, Chinese Academy of Sciences, Beijing, 100085, P.R. China.

[4]State Key Laboratory of Hydraulics and Mountain River Engineering, College of Architecture and Environment, Sichuan University, Chengdu, 610065, P.R. China.

*** Corresponding Authors**

Tao Zeng (email: zengtao@ucas.ac.cn);

Haiyan Zhang (email: zhanghaiyan@ucas.ac.cn);

Bo Lai (email: laibo@scu.edu.cn)

Supplementary Methods

Chemicals and reagents

4-fluorophthalonitrile (4-FP), 1,2-dicyanobenzene (DCB), 1,2,4,5-tetracyanobenzene (BTC), ethylene glycol, N, N-dimethylformamide (DMF), carbon nanotubes (CNTs) and β-carotene (βC) were purchased from Shanghai Macklin Biochemical Co., Ltd. Ferric chloride ($FeCl_3$), potassium thiocyanate (KSCN), 4-chlorophenol (4-CP), 9,10-diphenylanthracene (DPA) and nitroblue tetrazolium chloride (NBT) were supplied by Aladdin Industrial Corporation. Sodium periodate ($NaIO_4$), 5,5-dimethyl-1-pyrroline-N-oxide (DMPO), and 2,2,6,6-tetramethylpiperidine (TEMP) were purchased from Sigma-Aldrich Chemical Co., Ltd. Potassium Iodide (KI), 1,8-diazabicyclo[5.4.0]undec-7-ene (DBU) and 1,4-benzoquinone (*p*-BQ) were obtained from J&K Chemical Co., Ltd. *Tert*-butyl alcohol (TBA), methanol (MeOH), ethanol (EtOH), hydrochloric acid (HCl), furfuryl alcohol (FFA) and dimethyl sulfoxide (DMSO) were obtained from Sinopharm Chemical Reagent Co., Ltd. All chemicals used in this study were at least in analytical grade without any further purification. The water used in the whole research was deionized water. Pharmaceutical manufacturing wastewater was obtained from Zhejiang Zhebei Pharmaceutical Co. Ltd., and fine chemical wastewater was collected from Shangyu District Water Treatment Development Co. Ltd.

Supplementary Figures

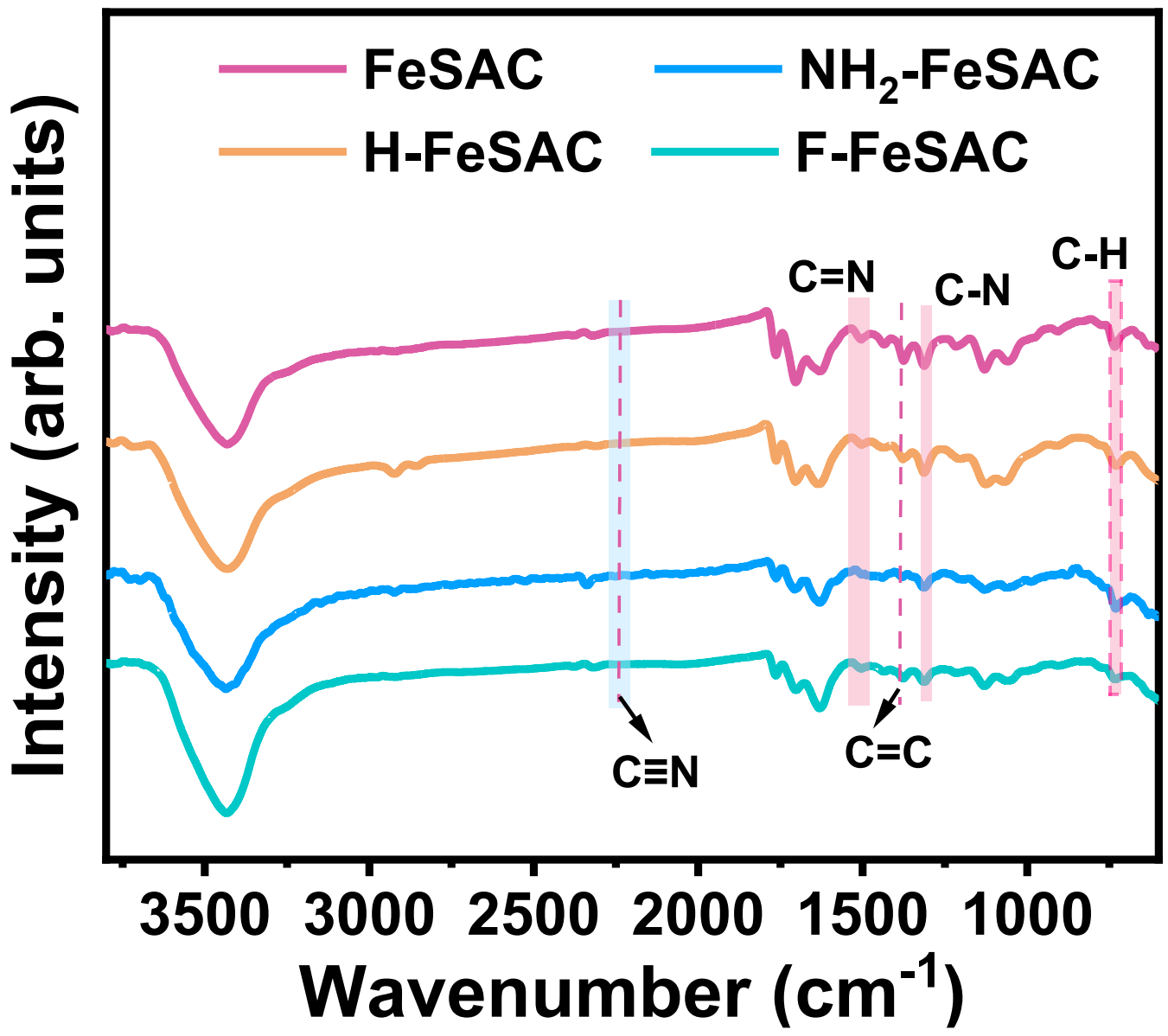

Supplementary Fig. 1 | FTIR characterization results. FTIR spectra of FeSAC, H-FeSAC, NH_2-FeSAC and F-FeSAC. Source data are provided as a Source Data file.

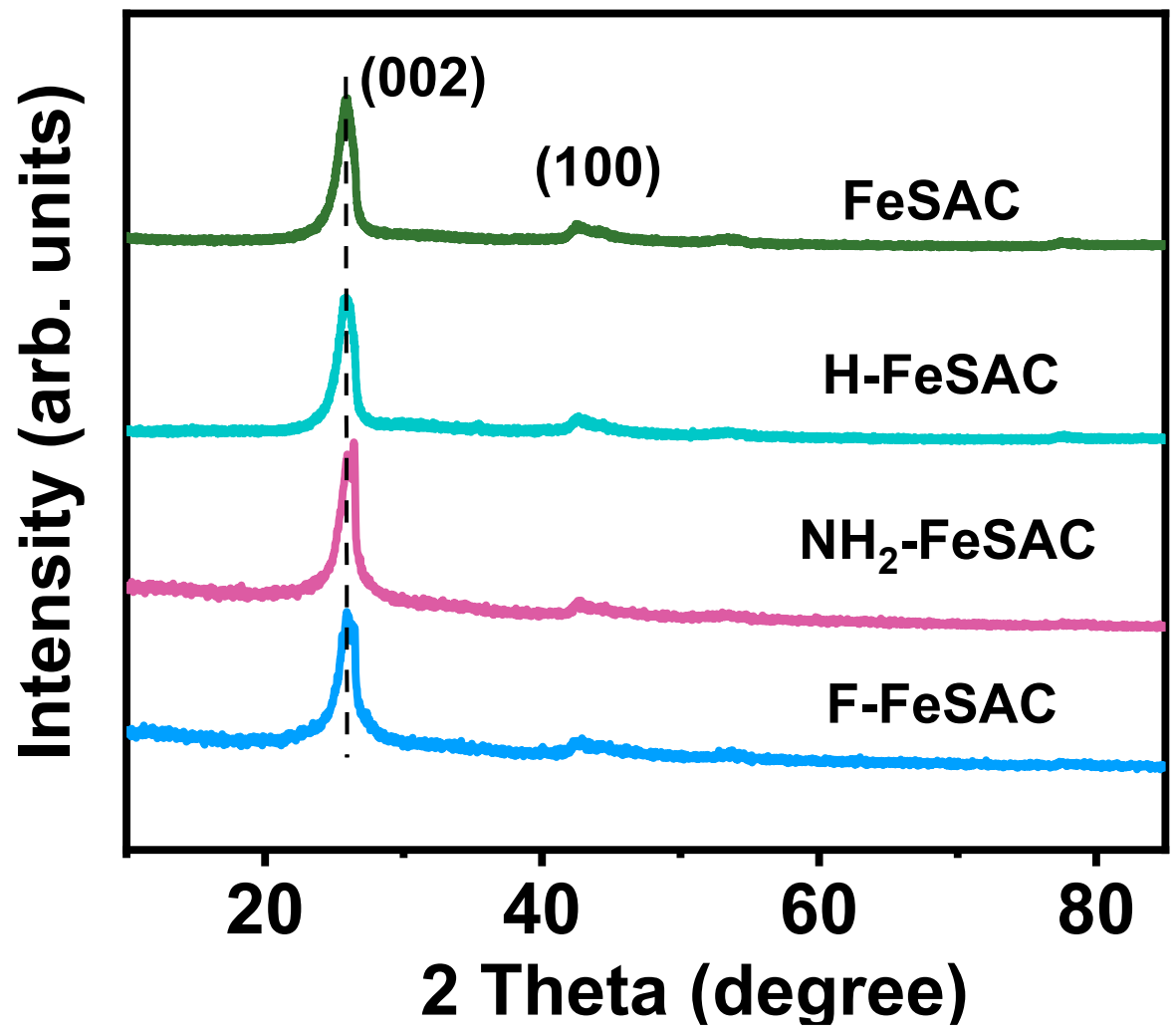

Supplementary Fig. 2 | XRD characterization results. XRD patterns of FeSAC, H-FeSAC, NH_2-FeSAC and F-FeSAC. Source data are provided as a Source Data file.

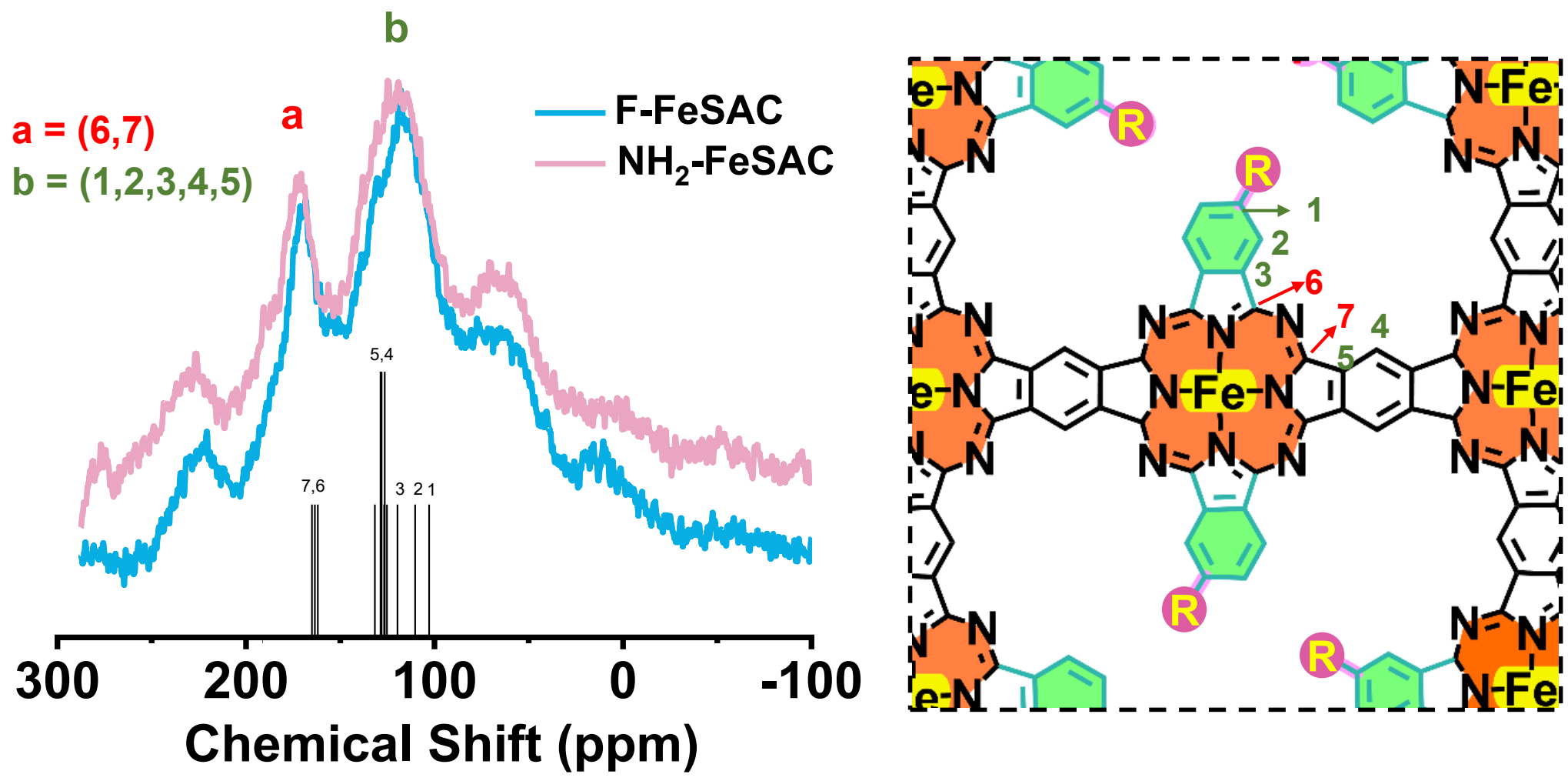

Supplementary Fig. 3 | Experimental and predicted ^{13}C NMR spectra. Solid-state ^{13}C NMR spectra and ChemDraw 20.0-predicted spectra of F-FeSAC and NH_2-FeSAC. Source data are provided as a Source Data file.

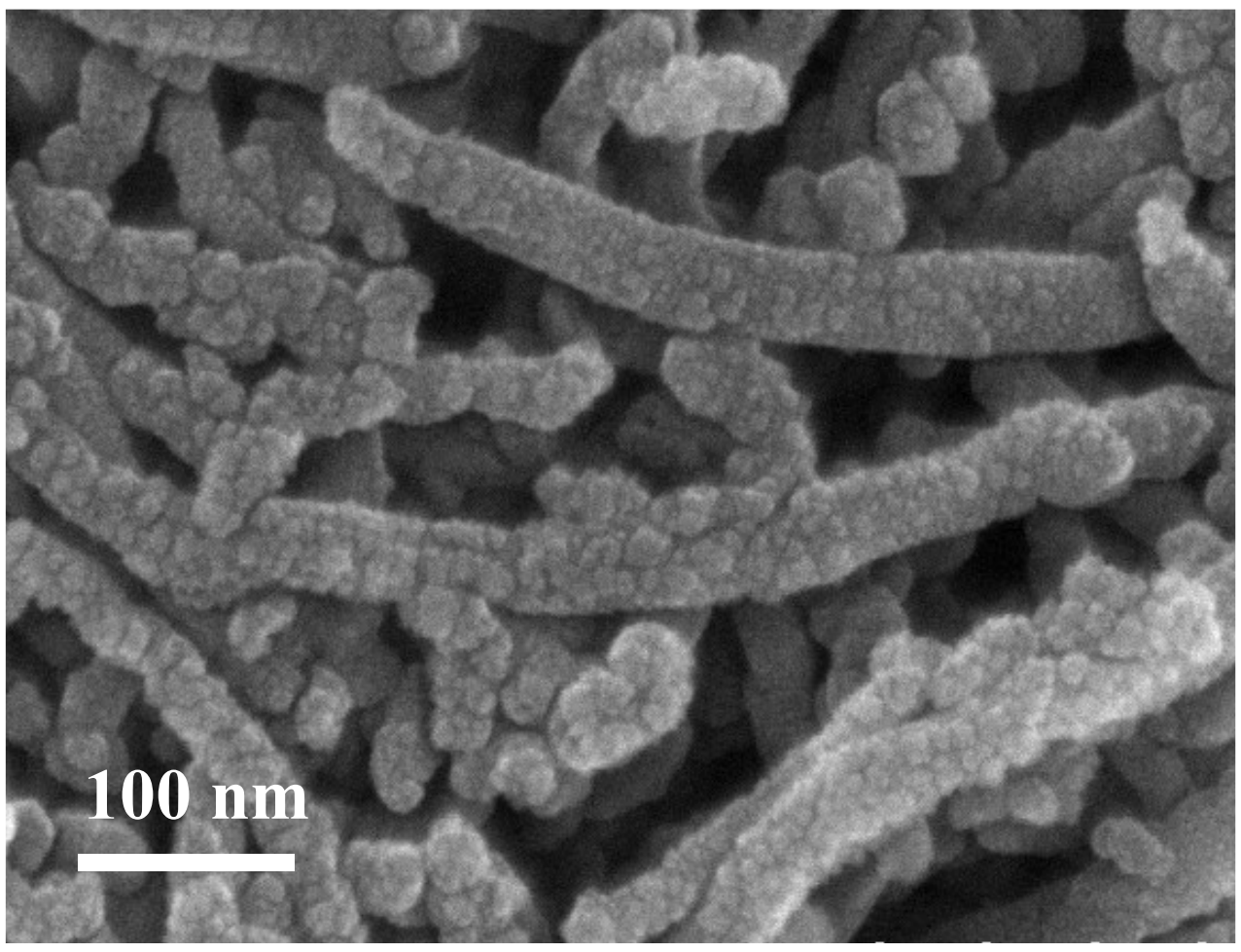

Supplementary Fig. 4 | Morphology of F-FeSAC. SEM image of F-FeSAC. Images are representative of three independent experiments with similar results. Source data are provided as a Source Data file.

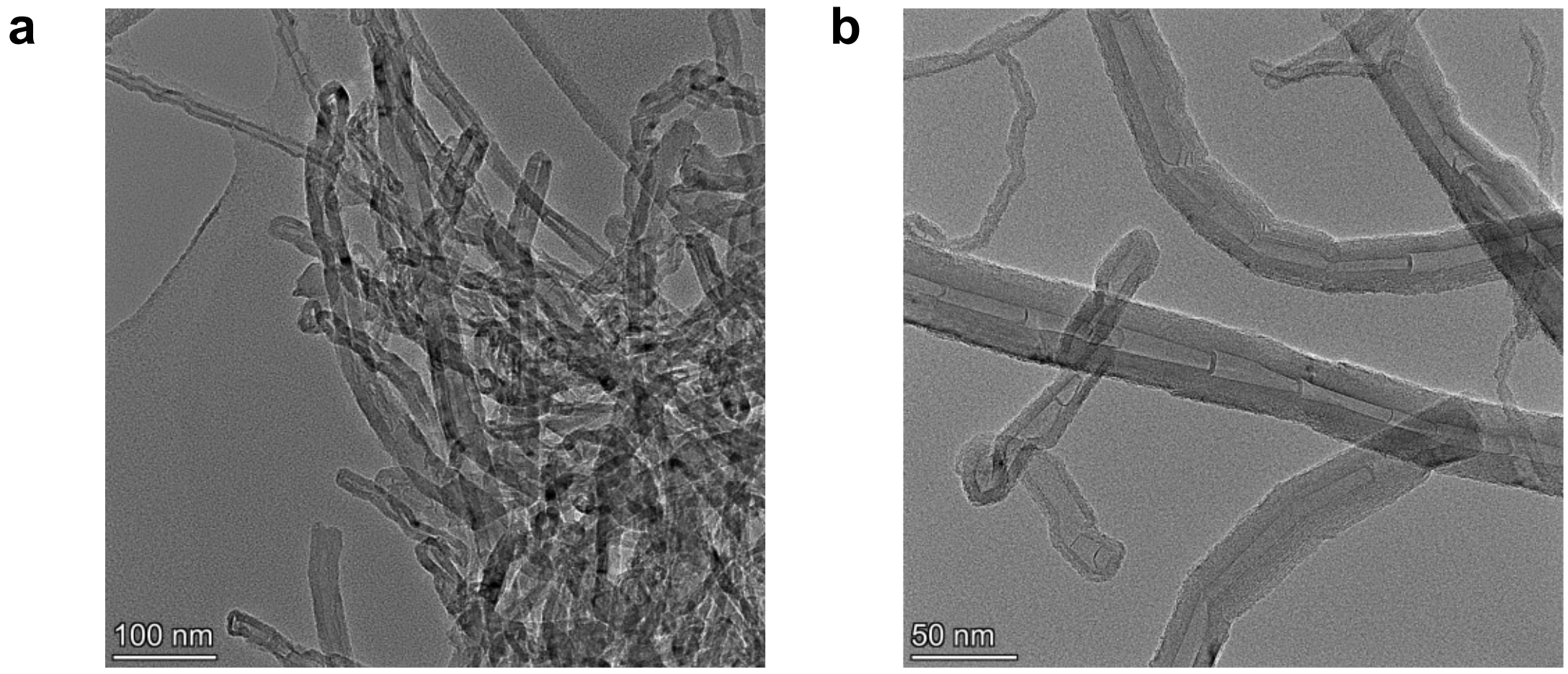

Supplementary Fig. 5 | Nanostructure of F-FeSAC. a-b HRTEM images of F-FeSAC. Images are representative of three independent experiments with similar results.

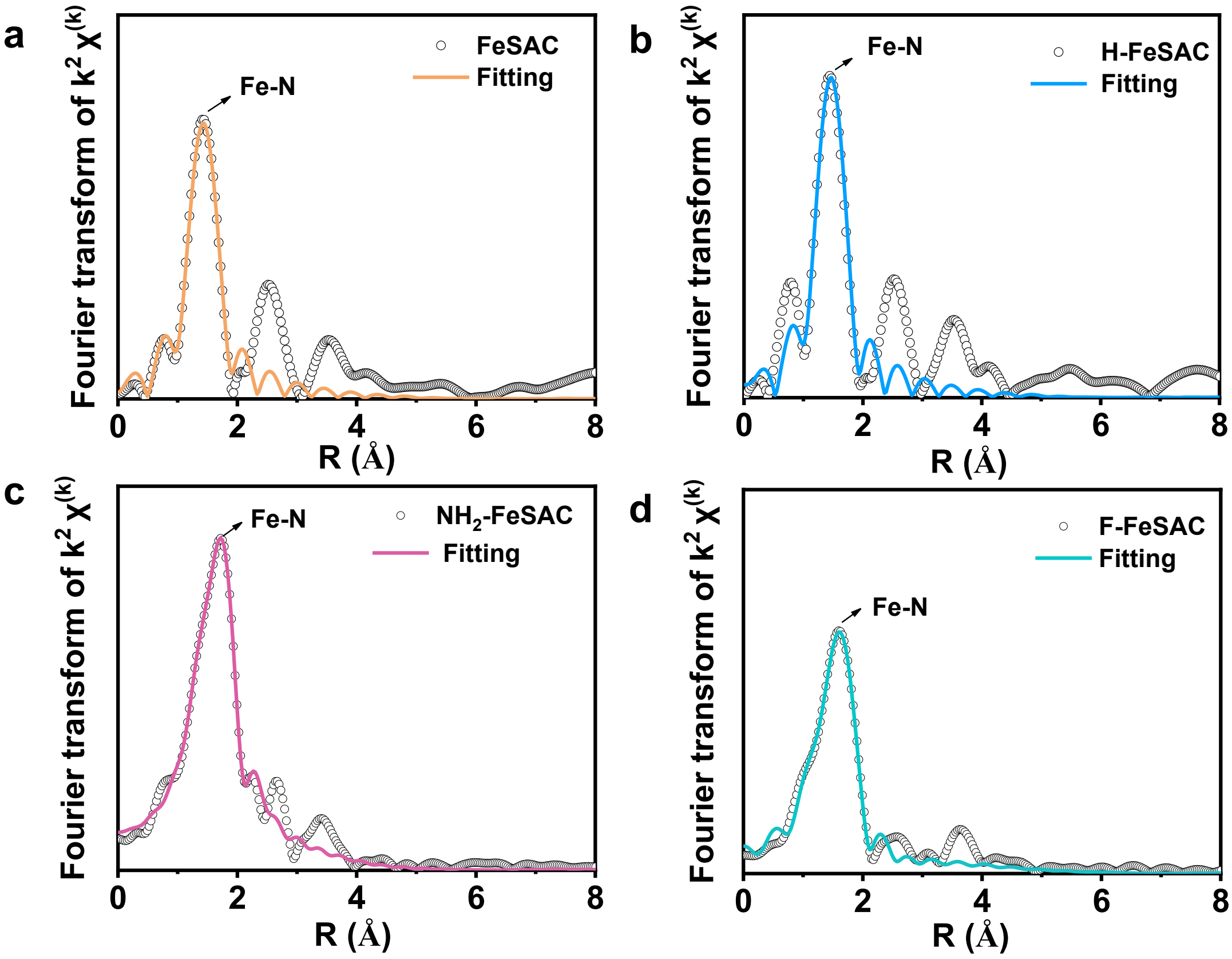

Supplementary Fig. 6 | EXAFS fitting results for FeSACs. The corresponding EXAFS fitting curves for (a) FeSAC, (b) H-FeSAC, (c) NH_2-FeSAC and (d) F-FeSAC. Source data are provided as a Source Data file.

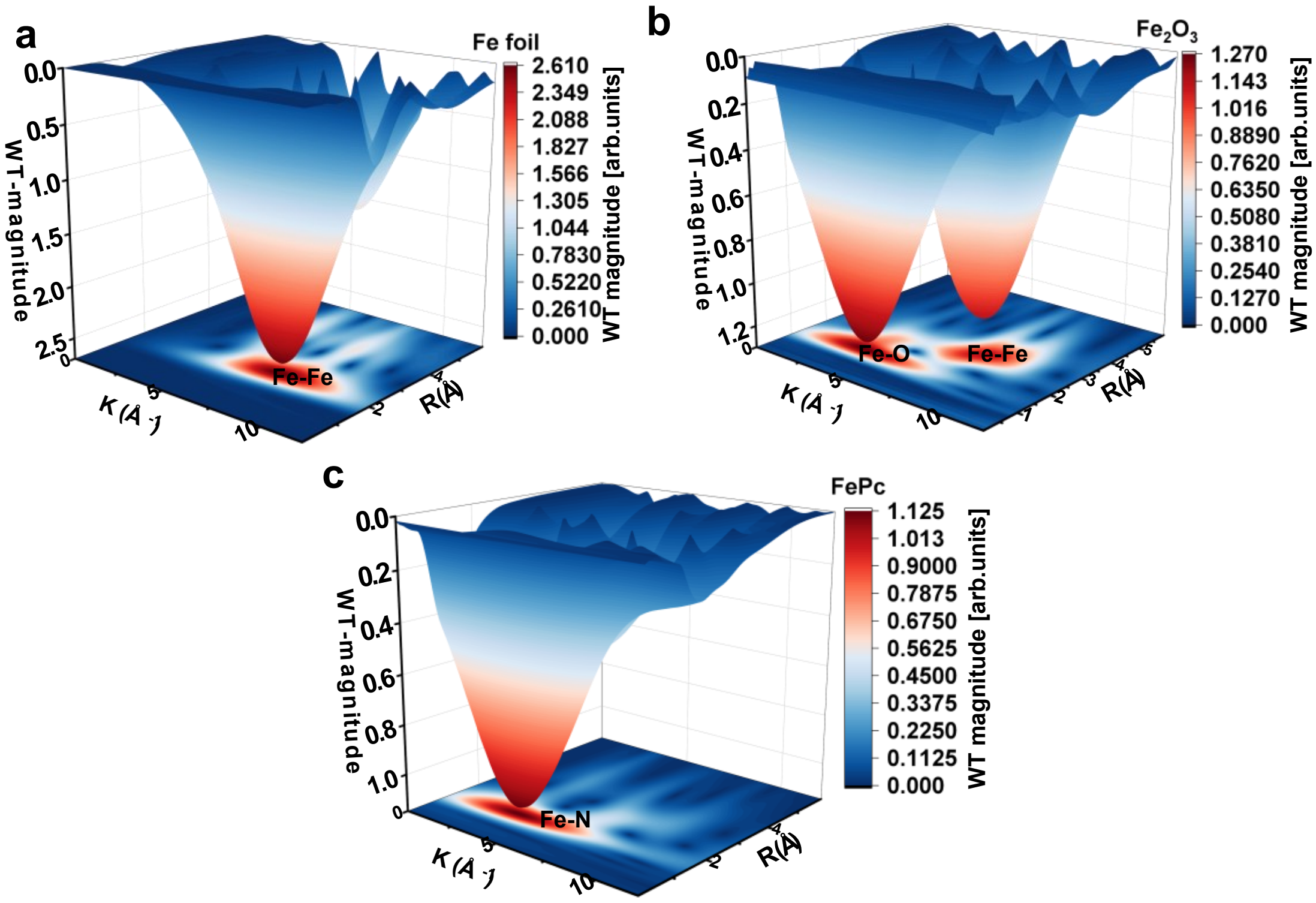

Supplementary Fig. 7 | Wavelet-transform analysis of Fe reference materials.

Wavelet transform analysis of (a) Fe foil, showing the Fe-Fe contribution; (b) Fe_2O_3, showing the Fe-O and Fe-Fe contributions; and (c) FePc, showing the Fe-N contribution.

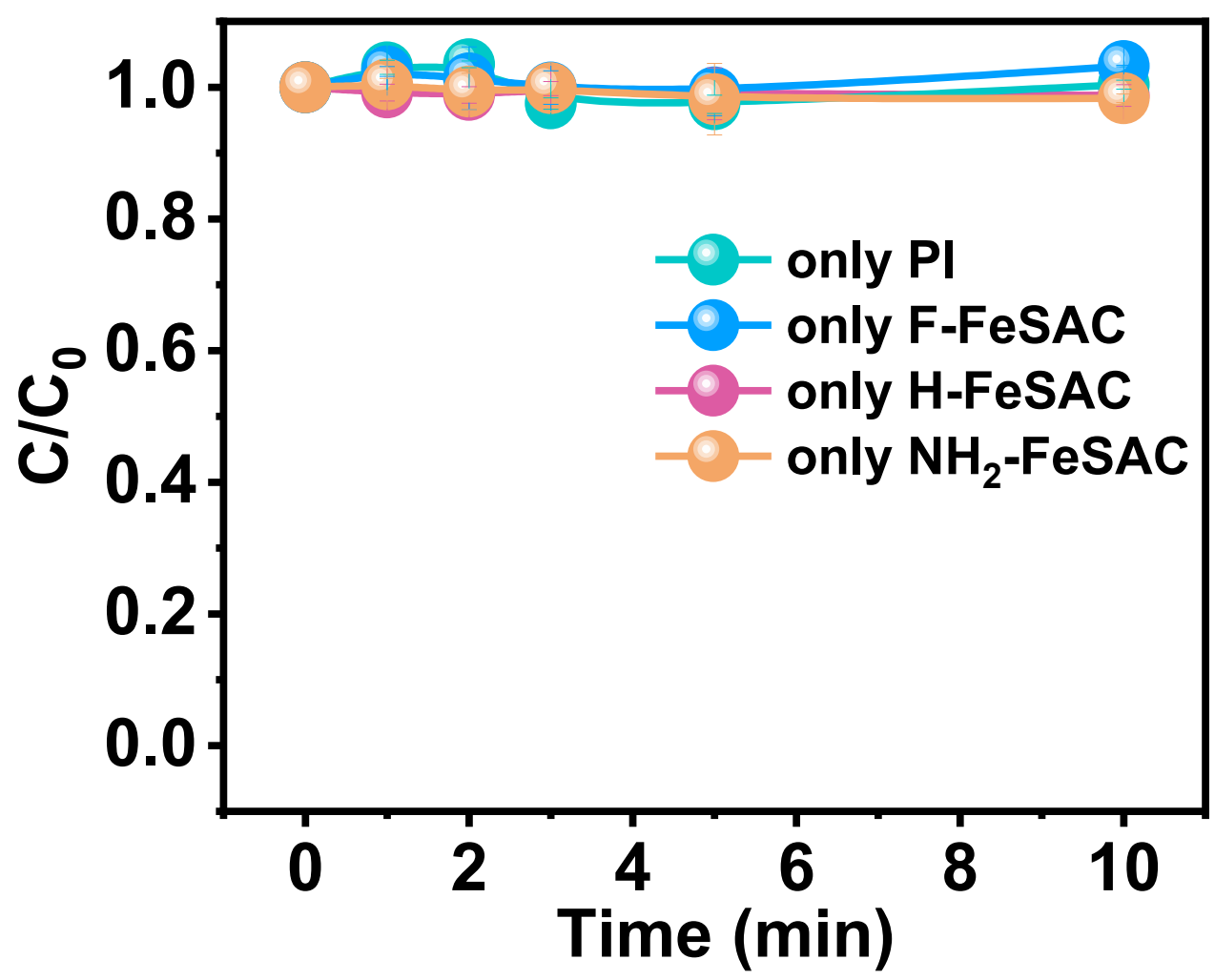

Supplementary Fig. 8 | Catalytic performance of different systems. Effects of different catalytic systems on the degradation of 4-CP. Data are presented as mean values ± SD from three experiments (n = 3). Source data are provided as a Source Data file.

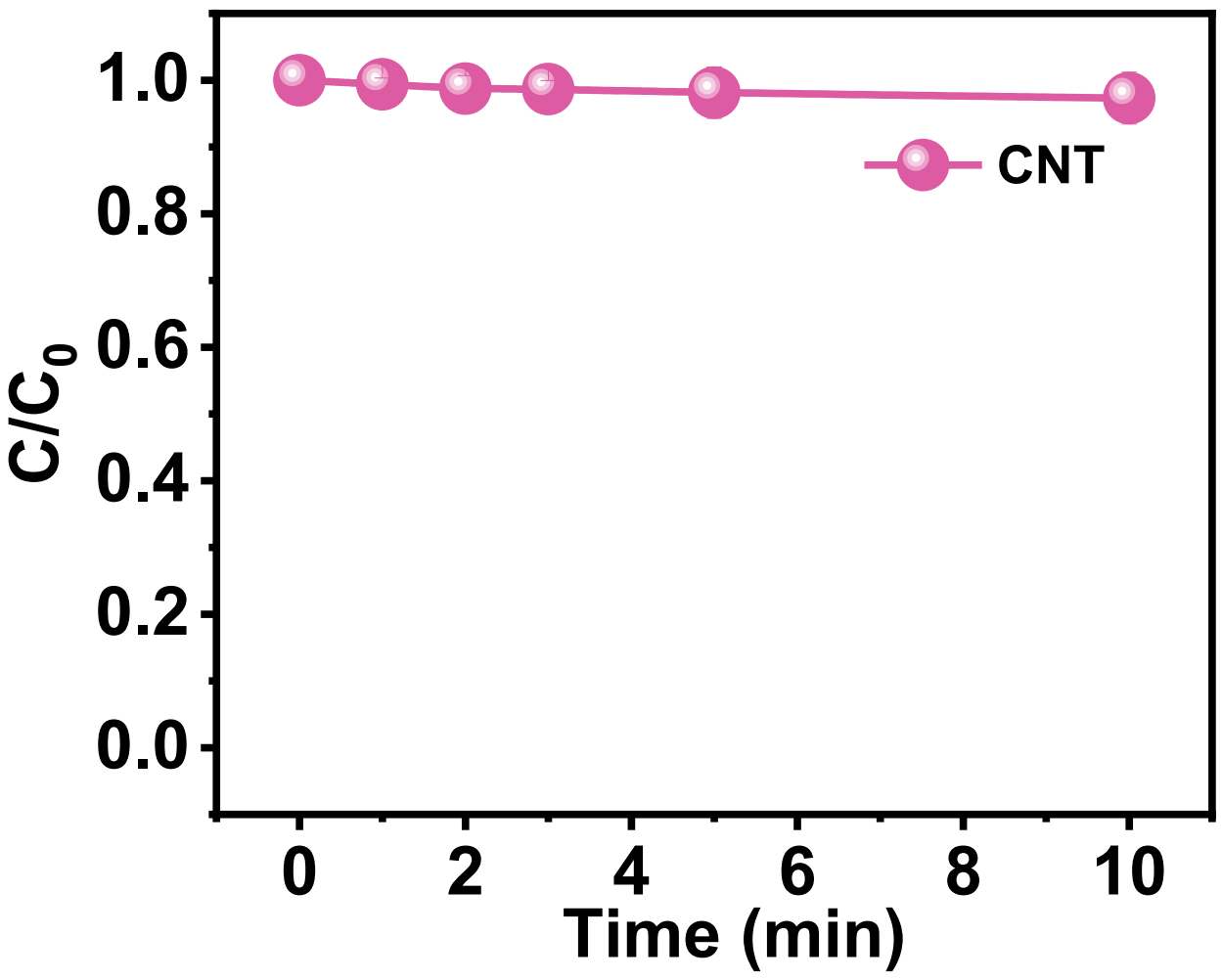

Supplementary Fig. 9 | Catalytic performance of CNT/PI systems. Effects of CNT/PI systems on the degradation of 4-CP. Data are presented as mean values ± SD from three experiments (n = 3). Source data are provided as a Source Data file.

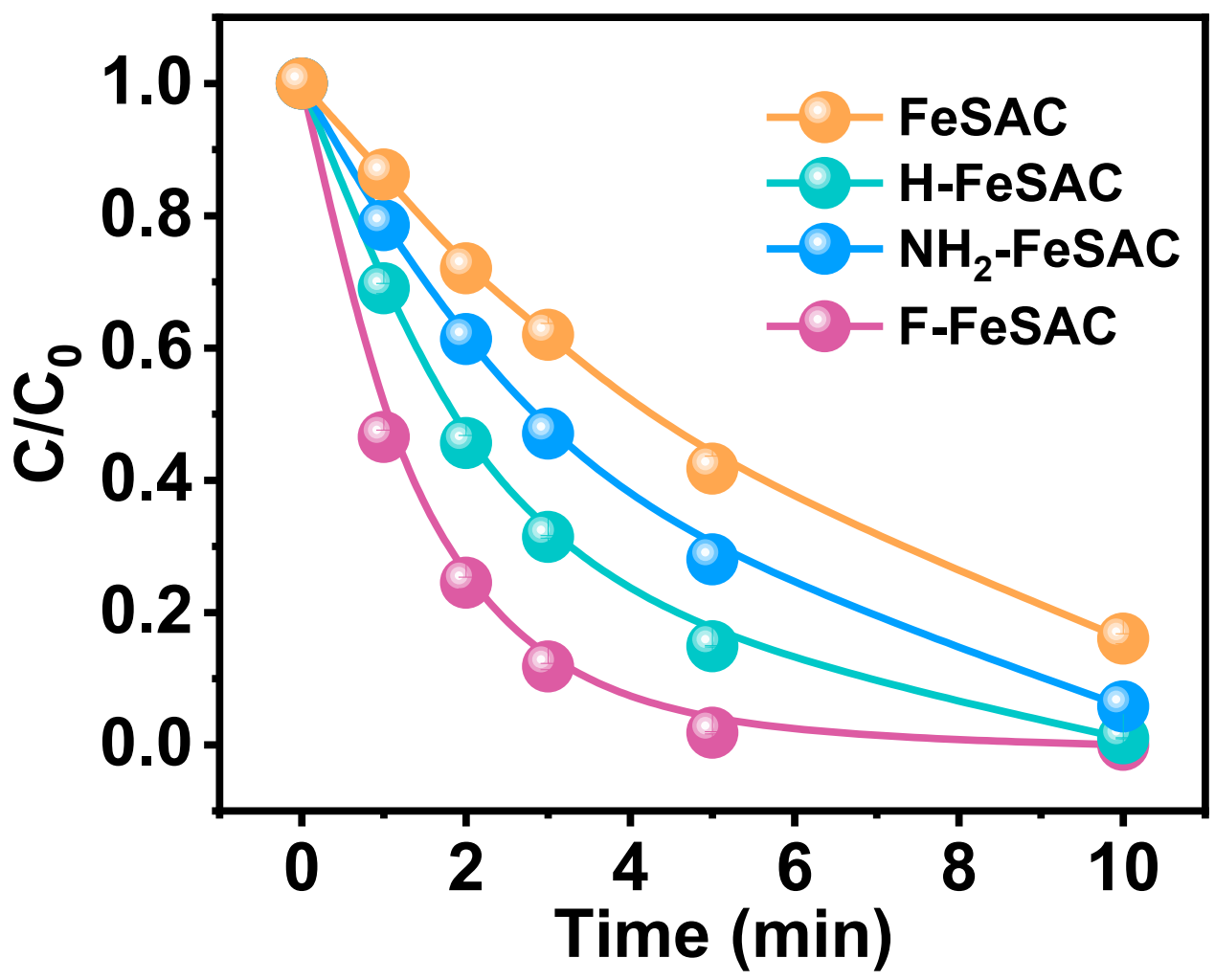

Supplementary Fig. 10 | Catalytic performance of different systems. Effects of different catalytic systems on the degradation of 4-CP. Data are presented as mean values ± SD from three experiments (n = 3). Source data are provided as a Source Data file.

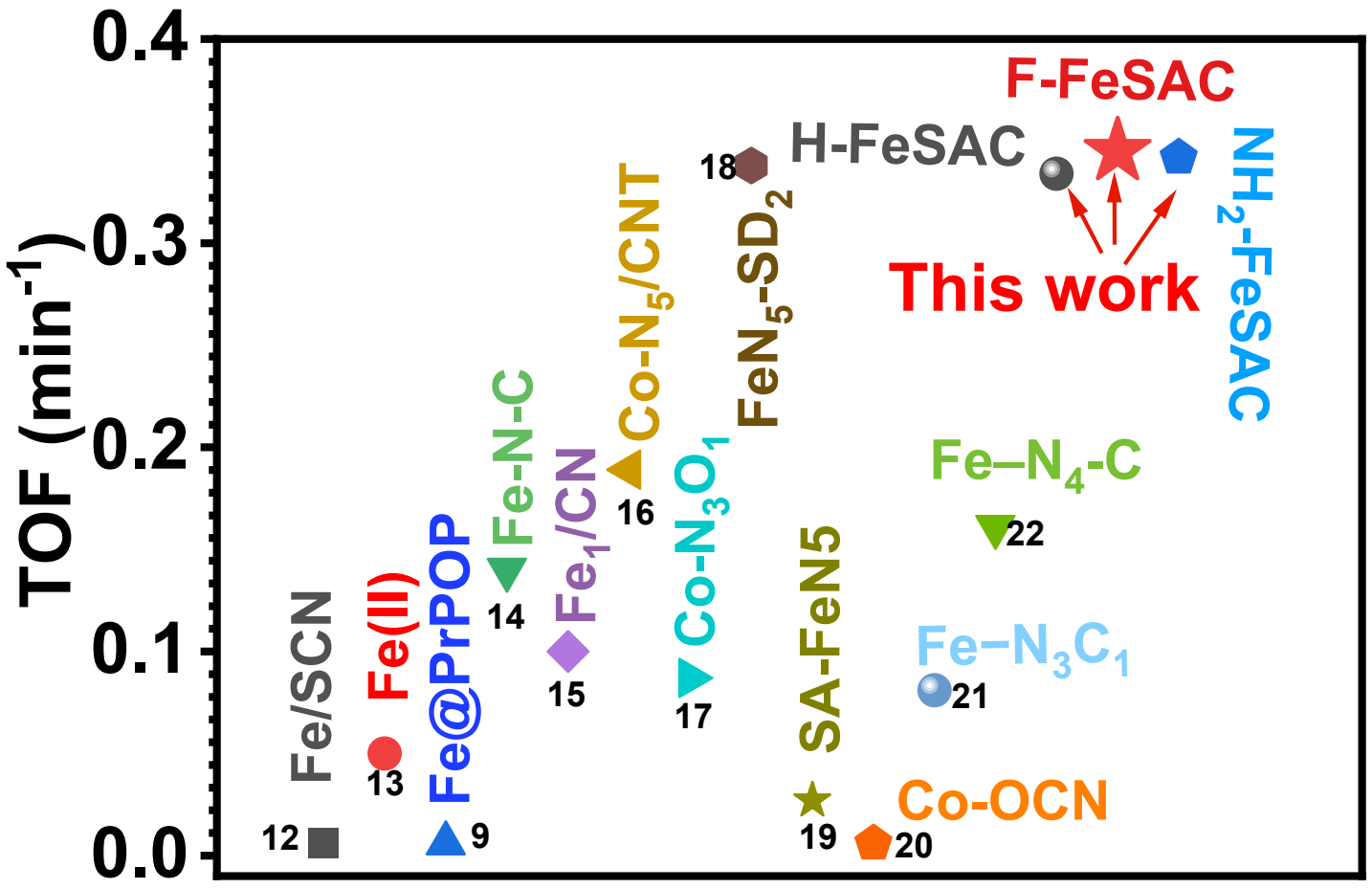

Supplementary Fig. 11 | TOF comparison among coordination-regulated SAC systems. Comparison of TOF values for pollutant degradation in SAC systems featuring coordination environment regulation. Numbers in the figure indicate the corresponding references. Source data are provided as a Source Data file.

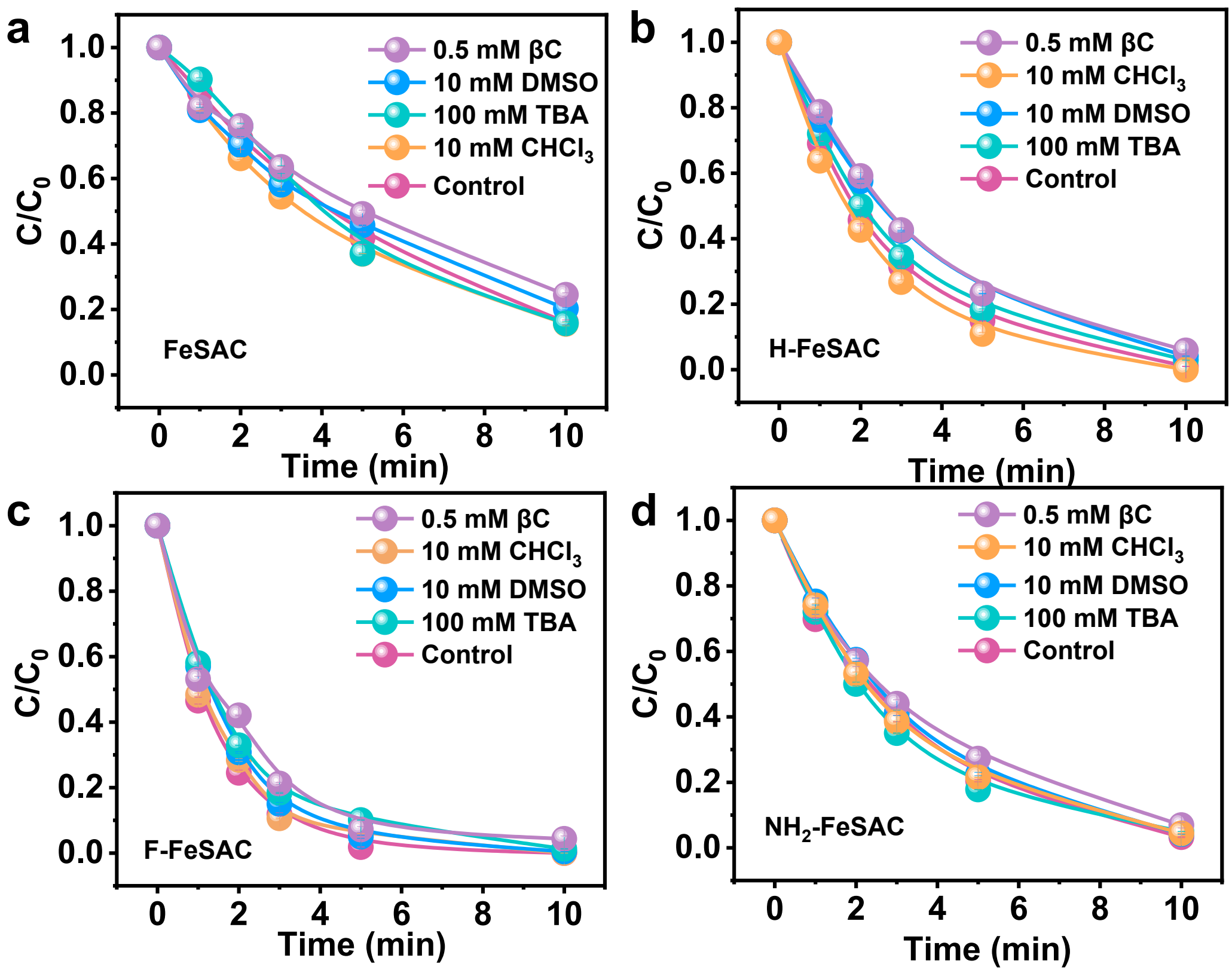

Supplementary Fig. 12 | Quenching effects on 4-CP degradation. Degradation of 4-CP in (a) FeSAC/PI, (b) H-FeSAC/PI, (c) F-FeSAC/PI and (d) NH_2-FeSAC/PI systems under different quenchers. Routine conditions: [4-CP] = 80 μM, [catalyst] = 50 mg L^{-1}, [PI] = 0.20 mM, temperature = 25 °C, without pH adjustment. Data are presented as mean values ± SD from three experiments (n = 3). Source data are provided as a Source Data file.

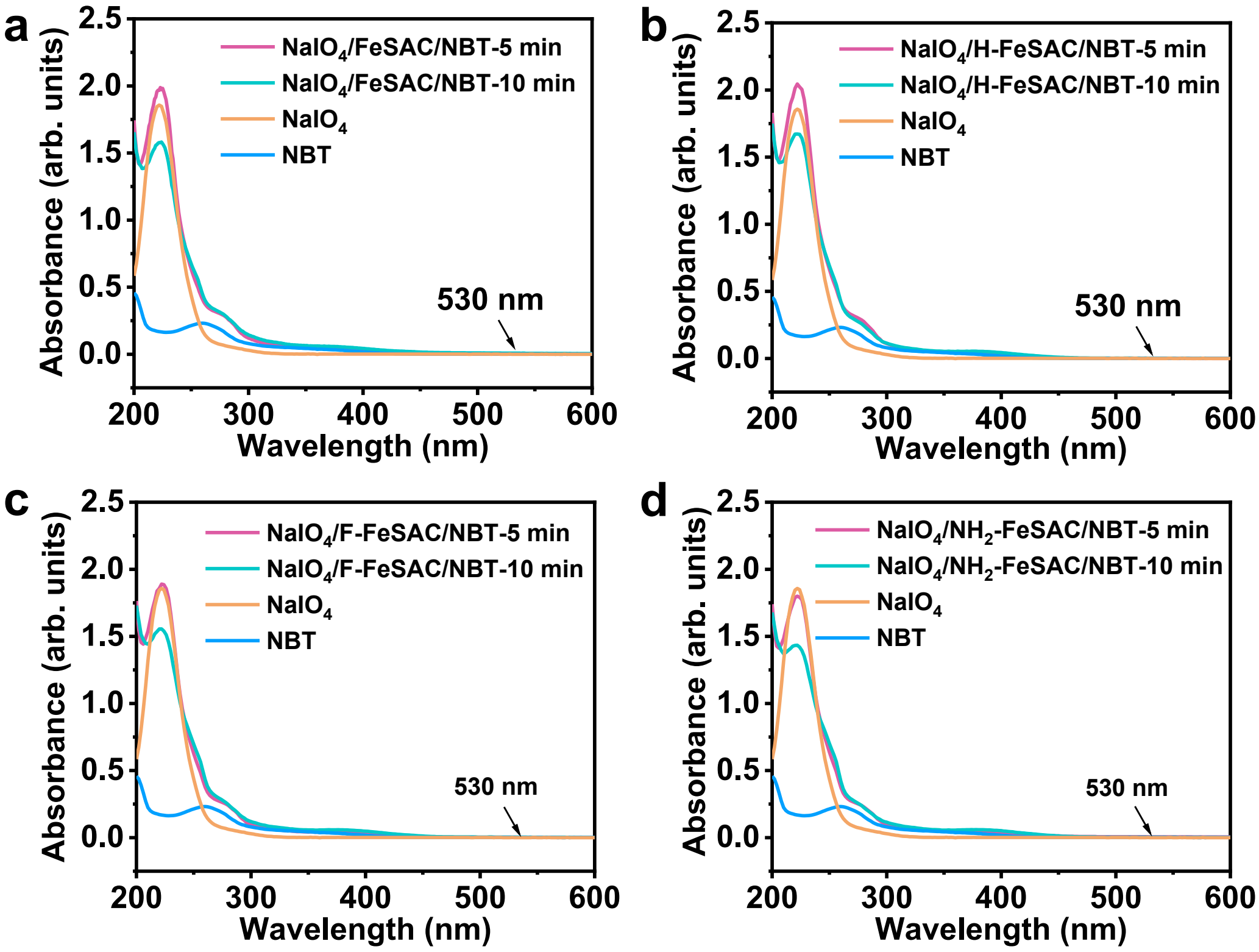

Supplementary Fig. 13 | NBT detection in FeSACs/PI systems. UV-vis absorption spectra of NBT in the (a) FeSAC/PI, (b) H-FeSAC/PI, (c) F-FeSAC/PI and (d) NH_2-FeSAC/PI systems. Source data are provided as a Source Data file.

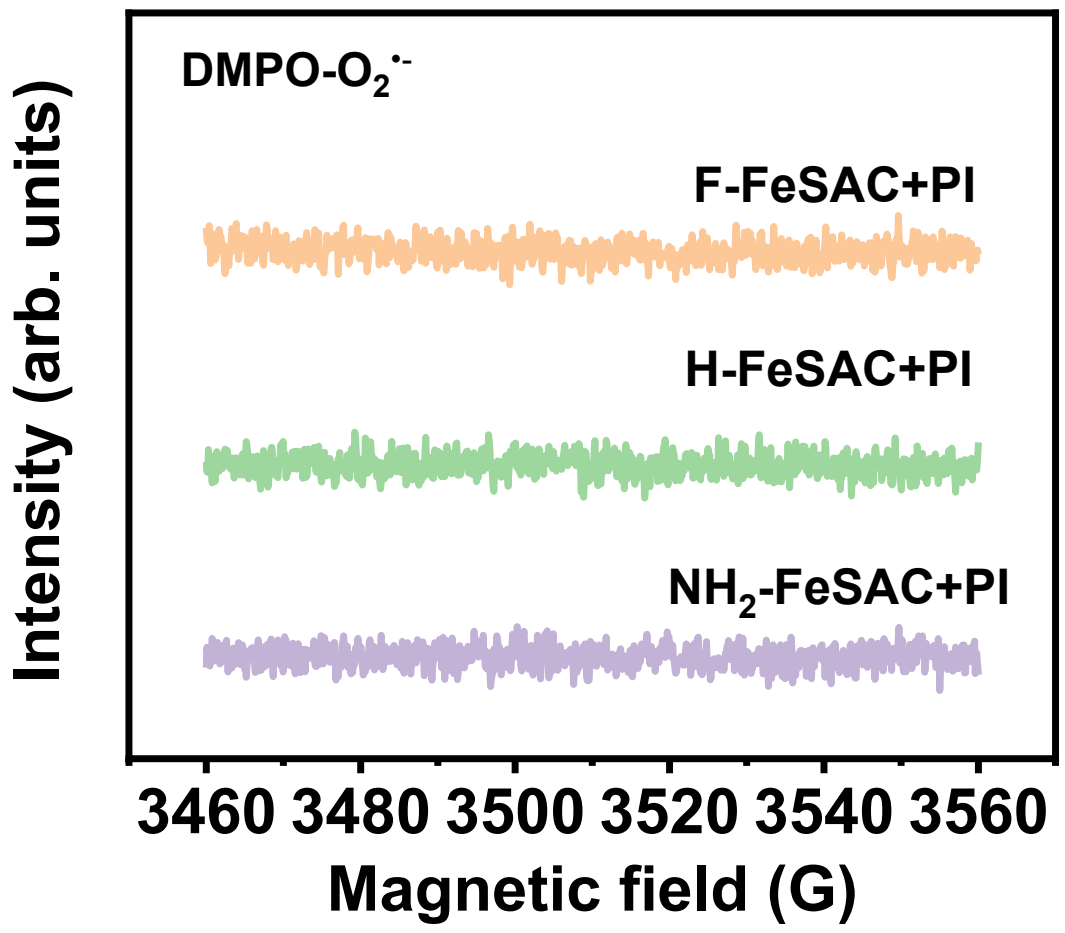

Supplementary Fig. 14 | EPR detection of superoxide radicals. EPR spectra of $O_2^{\bullet-}$ trapped by DMPO in F-FeSAC/PI, H-FeSAC/PI, and NH_2-FeSAC/PI systems. Source data are provided as a Source Data file.

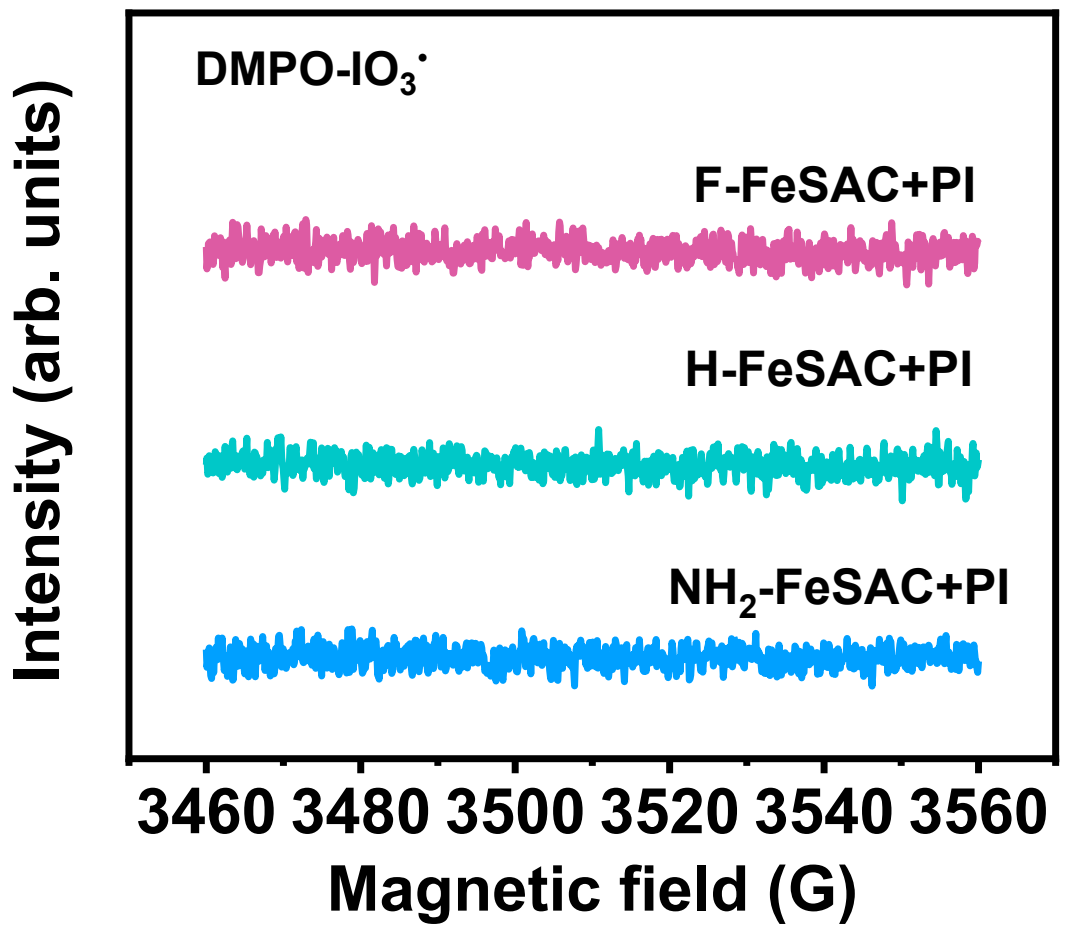

Supplementary Fig. 15 | EPR detection of iodate radicals. EPR spectra of $IO_3^{\bullet}$ trapped by DMPO in F-FeSAC/PI, H-FeSAC/PI, and NH_2-FeSAC/PI systems. Source data are provided as a Source Data file.

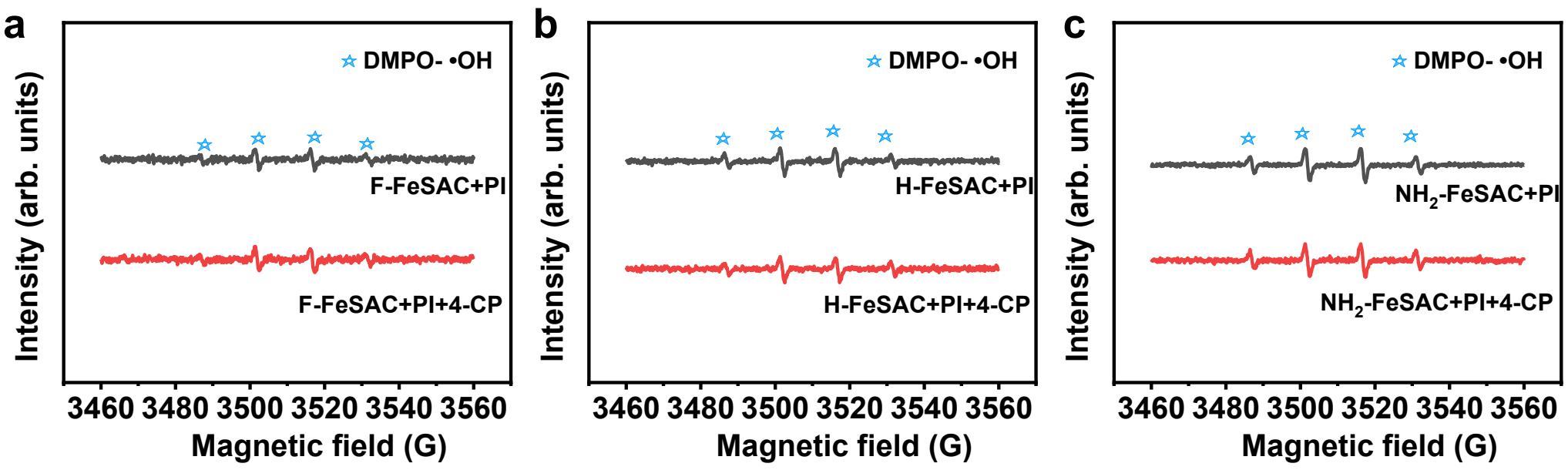

Supplementary Fig. 16 | EPR detection of hydroxyl radicals. EPR spectra of •OH trapped by DMPO in (a) F-FeSAC/PI, (b) H-FeSAC/PI, and (c) NH_2-FeSAC/PI systems. Source data are provided as a Source Data file.

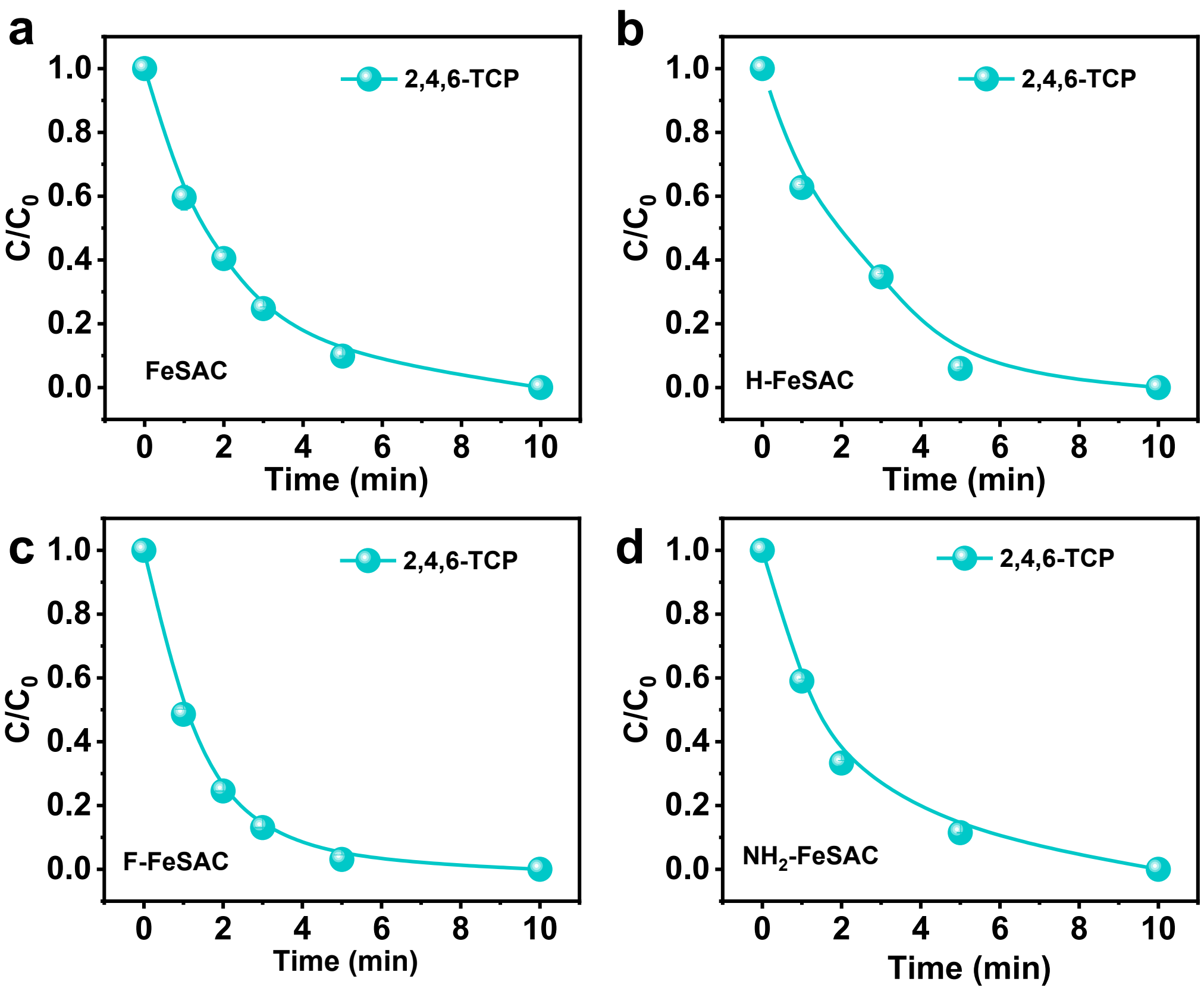

Supplementary Fig. 17 | Degradation of 2,4,6-TCP by FeSACs/PI systems. Degradation of 2,4,6-TCP in the (a) FeSAC/PI, (b) H-FeSAC/PI, (c) F-FeSAC/PI and (d) NH_2-FeSAC/PI systems. Data are presented as mean values ± SD from three experiments (n = 3). Source data are provided as a Source Data file.

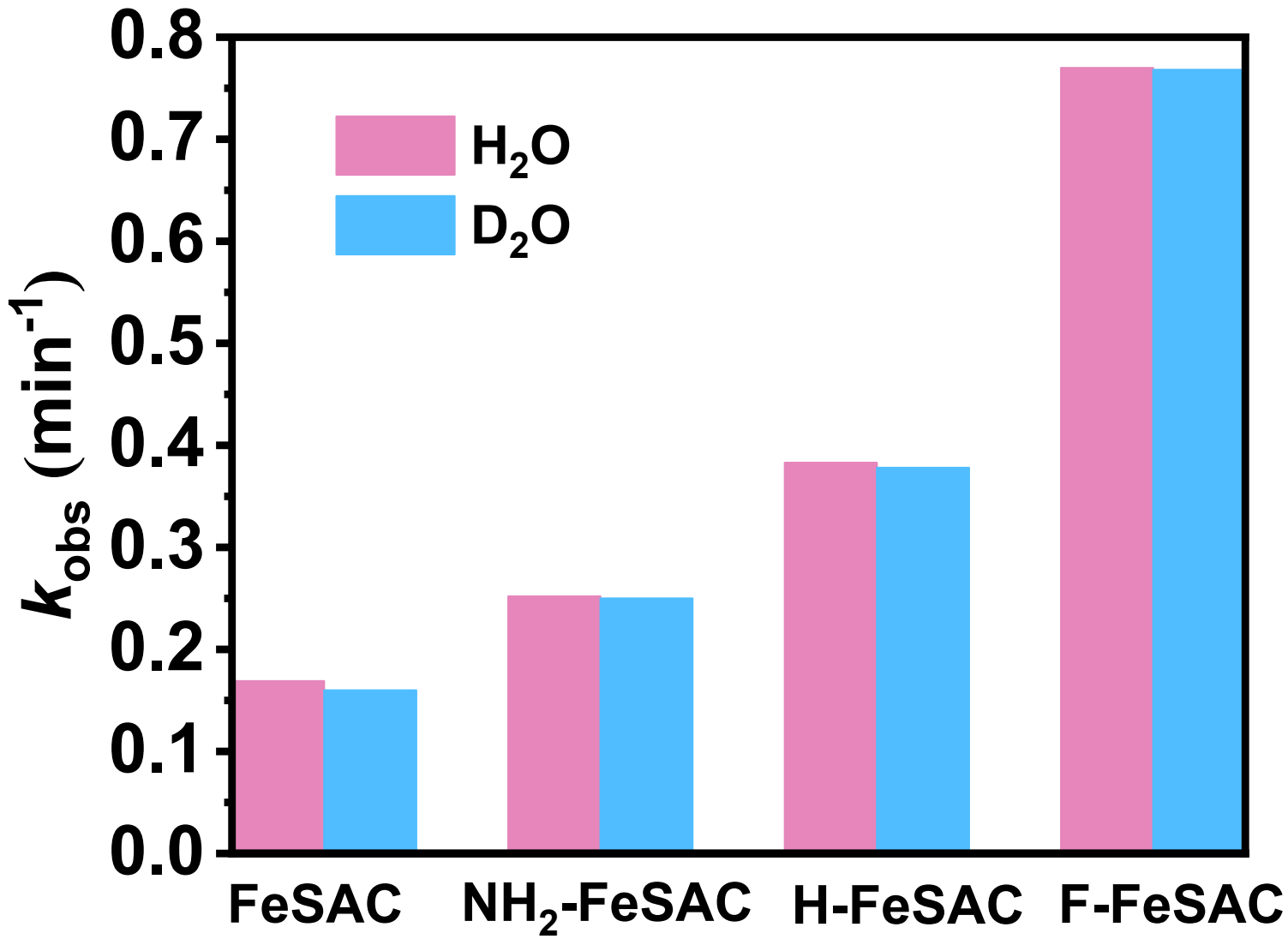

Supplementary Fig. 18 | Solvent isotope effects on catalytic activity. Effect of reaction solvents (H_2O and D_2O) on k_{obs} in different systems. Source data are provided as a Source Data file.

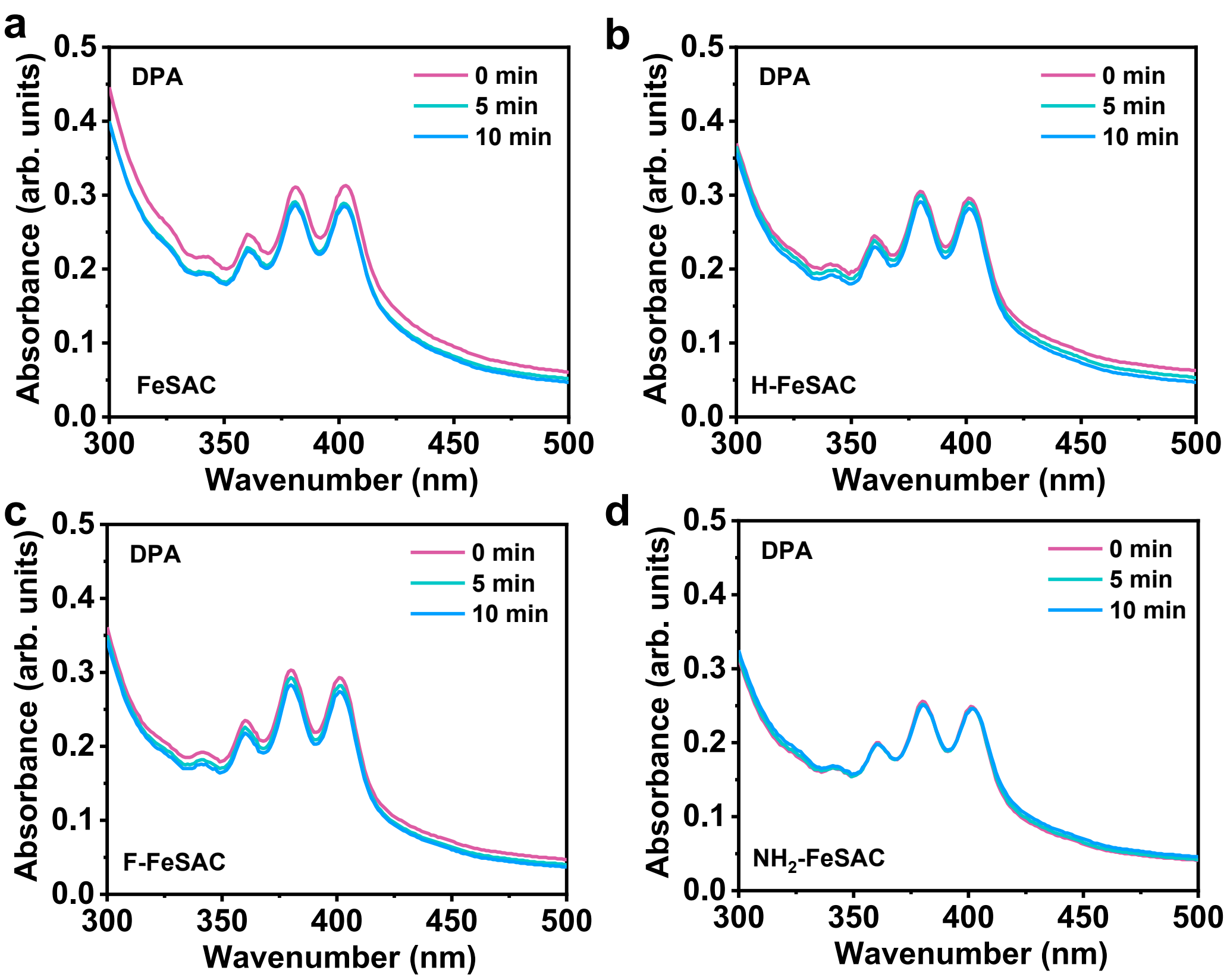

Supplementary Fig. 19 | DPA signals in FeSACs/PI systems. DPA signals in (a) FeSAC/PI, (b) H-FeSAC/PI, (c) F-FeSAC/PI and (d) NH_2-FeSAC/PI systems. Routine conditions: [4-CP] = 80 μM, [catalyst] = 50 mg L^{-1}, [PI] = 0.20 mM, temperature = 25 °C, without pH adjustment. Source data are provided as a Source Data file.

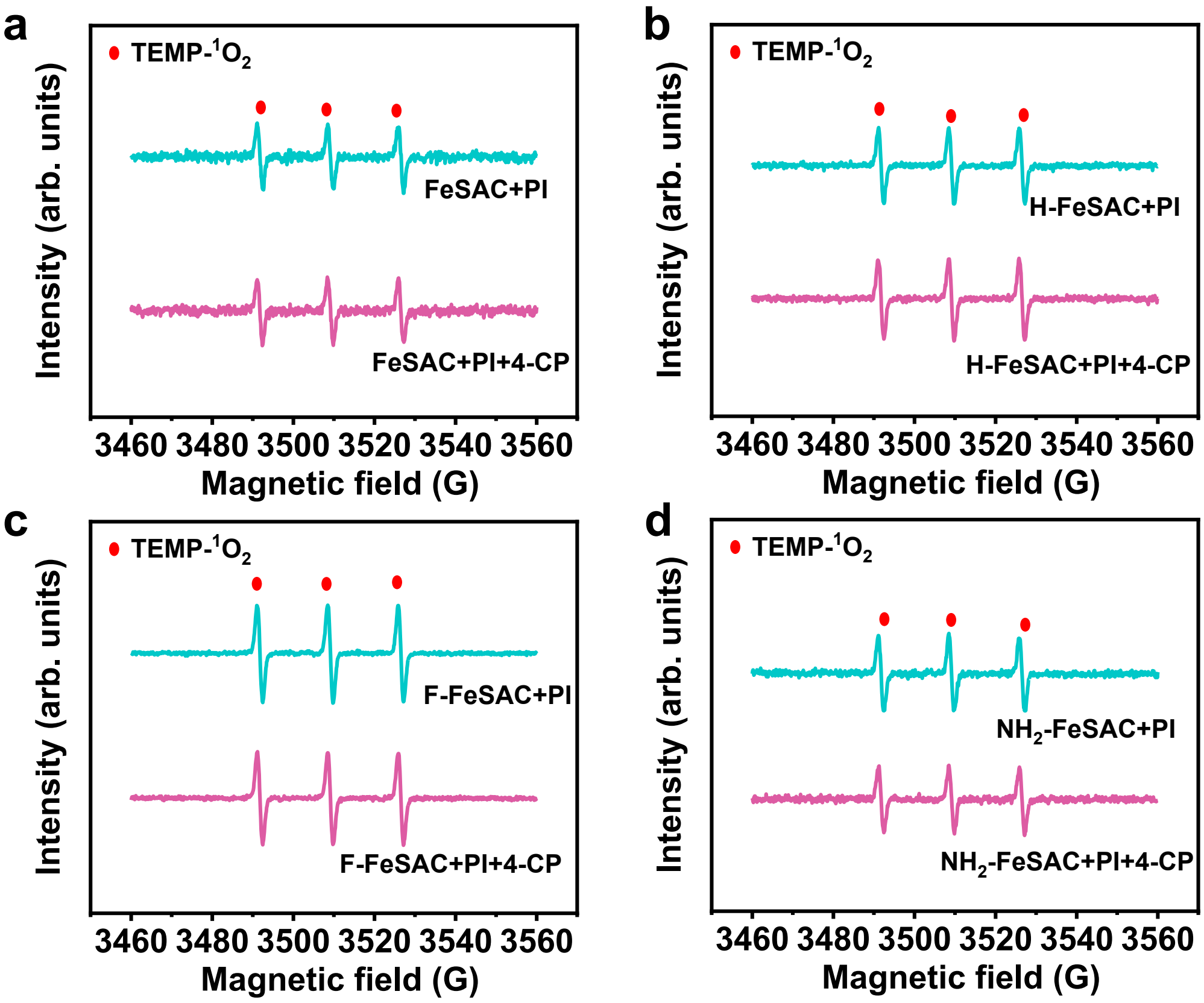

Supplementary Fig. 20 | EPR detection of singlet oxygen. EPR spectra of $^1O_2$ trapped by TEMP in (a) FeSAC/PI, (b) H-FeSAC/PI, (c) F-FeSAC/PI and (d) NH_2-FeSAC/PI systems. Source data are provided as a Source Data file.

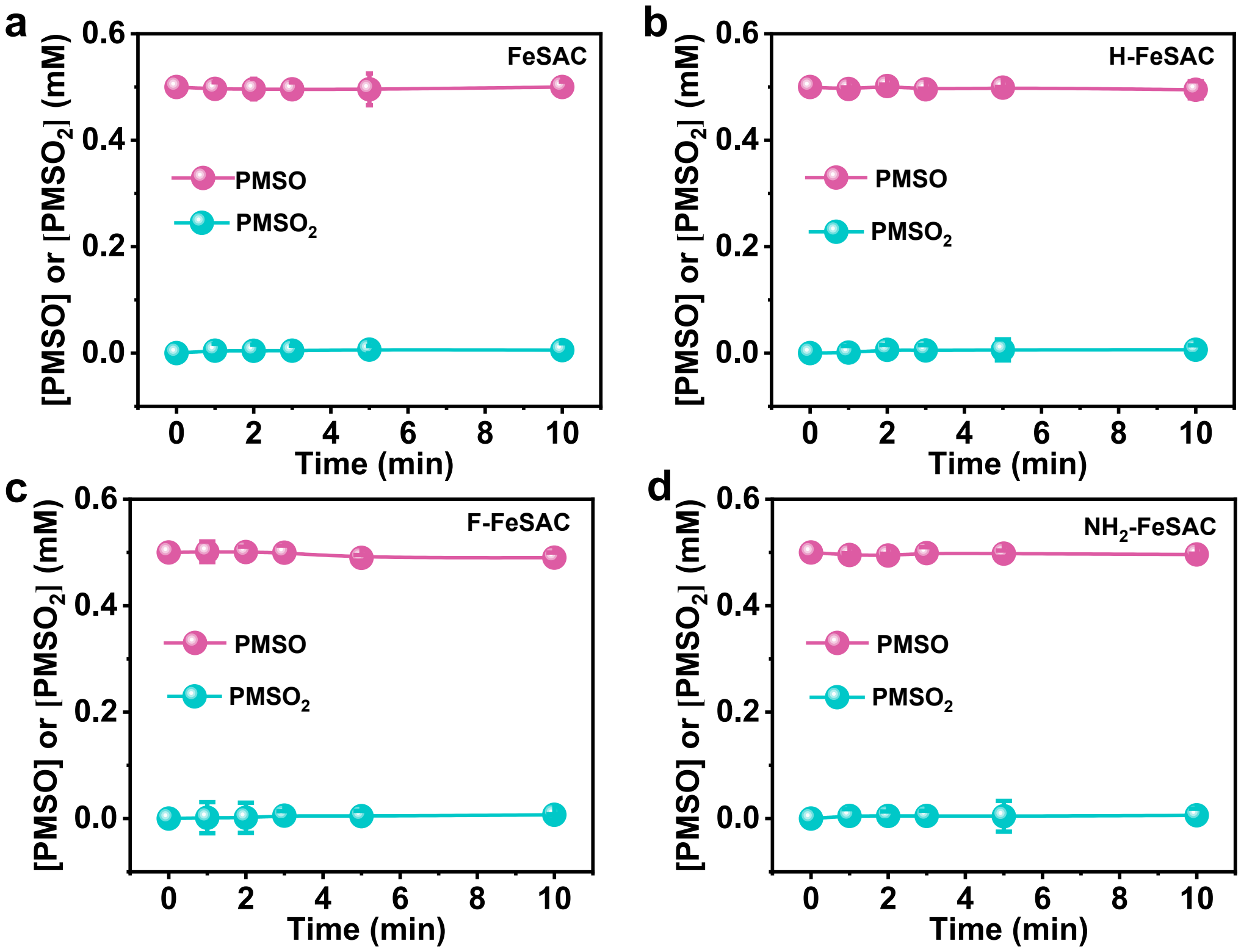

Supplementary Fig. 21 | PMSO oxidation in FeSACs/PI systems. Concentration changes of PMSO and $PMSO_2$ in (a) FeSAC/PI, (b) H-FeSAC/PI, (c) F-FeSAC/PI and (d) NH_2-FeSAC/PI systems. Data are presented as mean values ± SD from three experiments (n = 3). Source data are provided as a Source Data file.

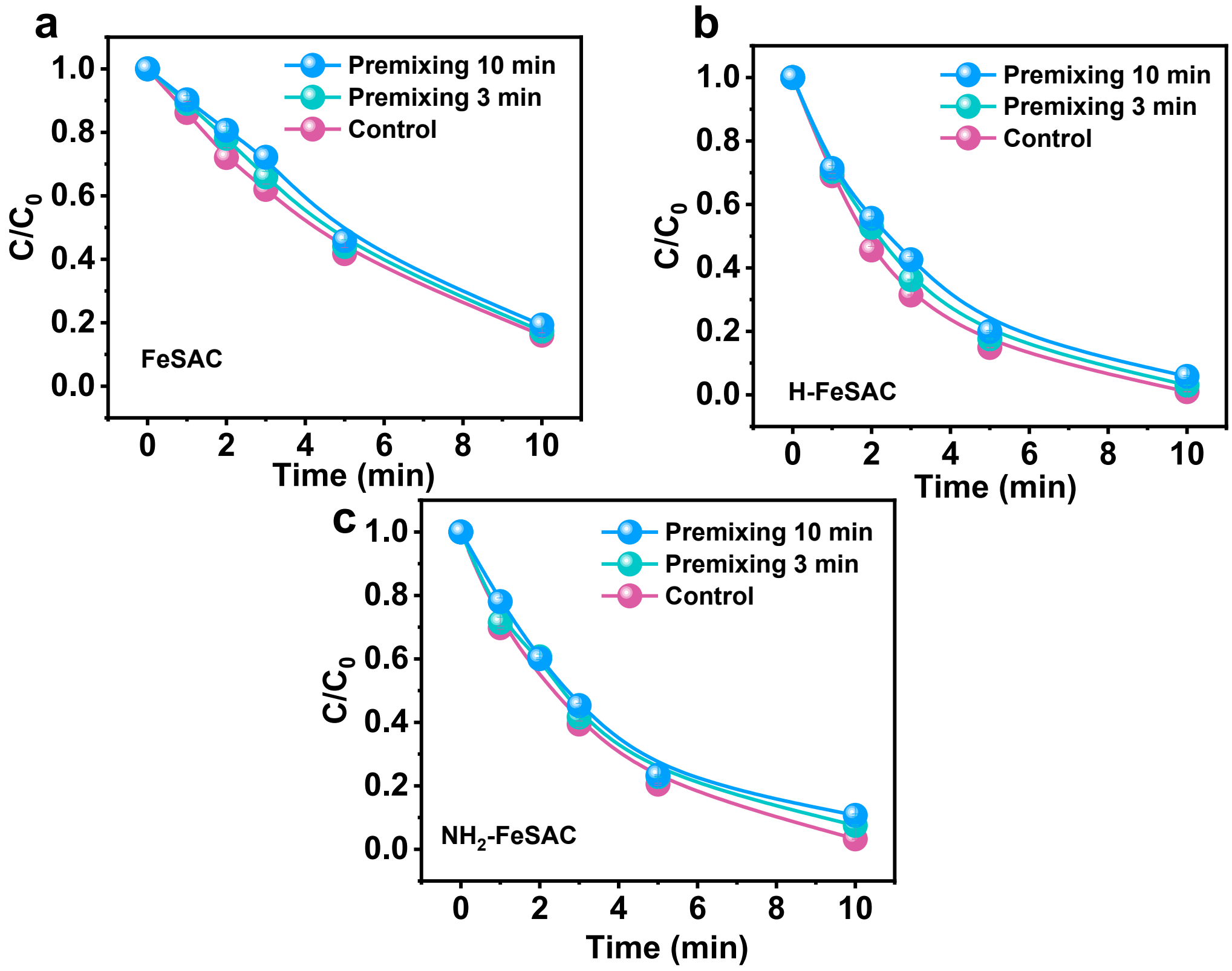

Supplementary Fig. 22 | Premixing effects on 4-CP removal. Effect of premixing in the (a) FeSAC/PI, (b) H-FeSAC/PI, (c) NH_2-FeSAC/PI systems on 4-CP removal. Data are presented as mean values ± SD from three experiments (n = 3). Source data are provided as a Source Data file.

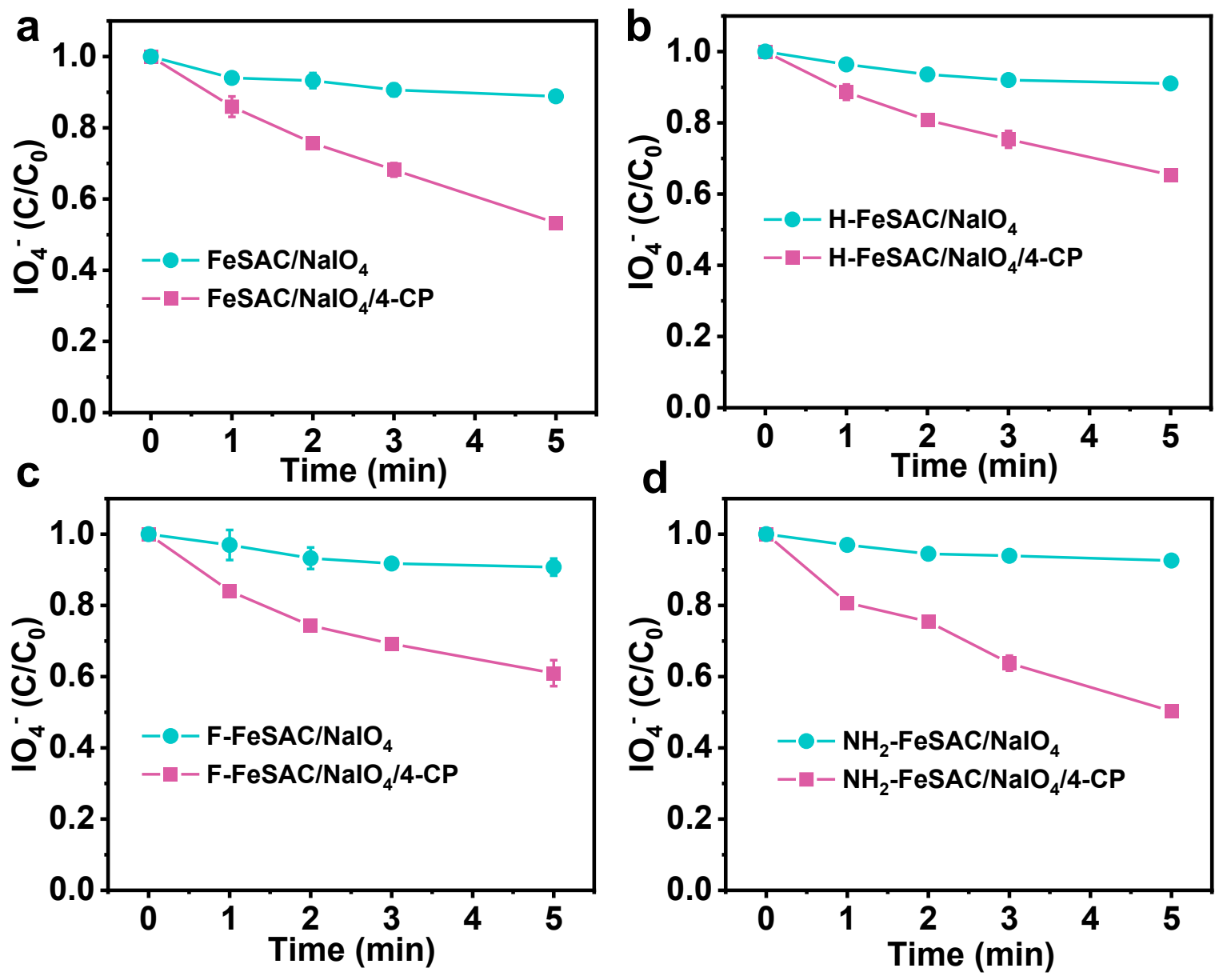

Supplementary Fig. 23 | PI decomposition in FeSACs/PI systems. Decomposition rate of PI in (a) FeSAC/PI, (b) H-FeSAC/PI, (c) F-FeSAC/PI and (d) NH_2-FeSAC/PI systems. Data are presented as mean values ± SD from three experiments (n = 3). Source data are provided as a Source Data file.

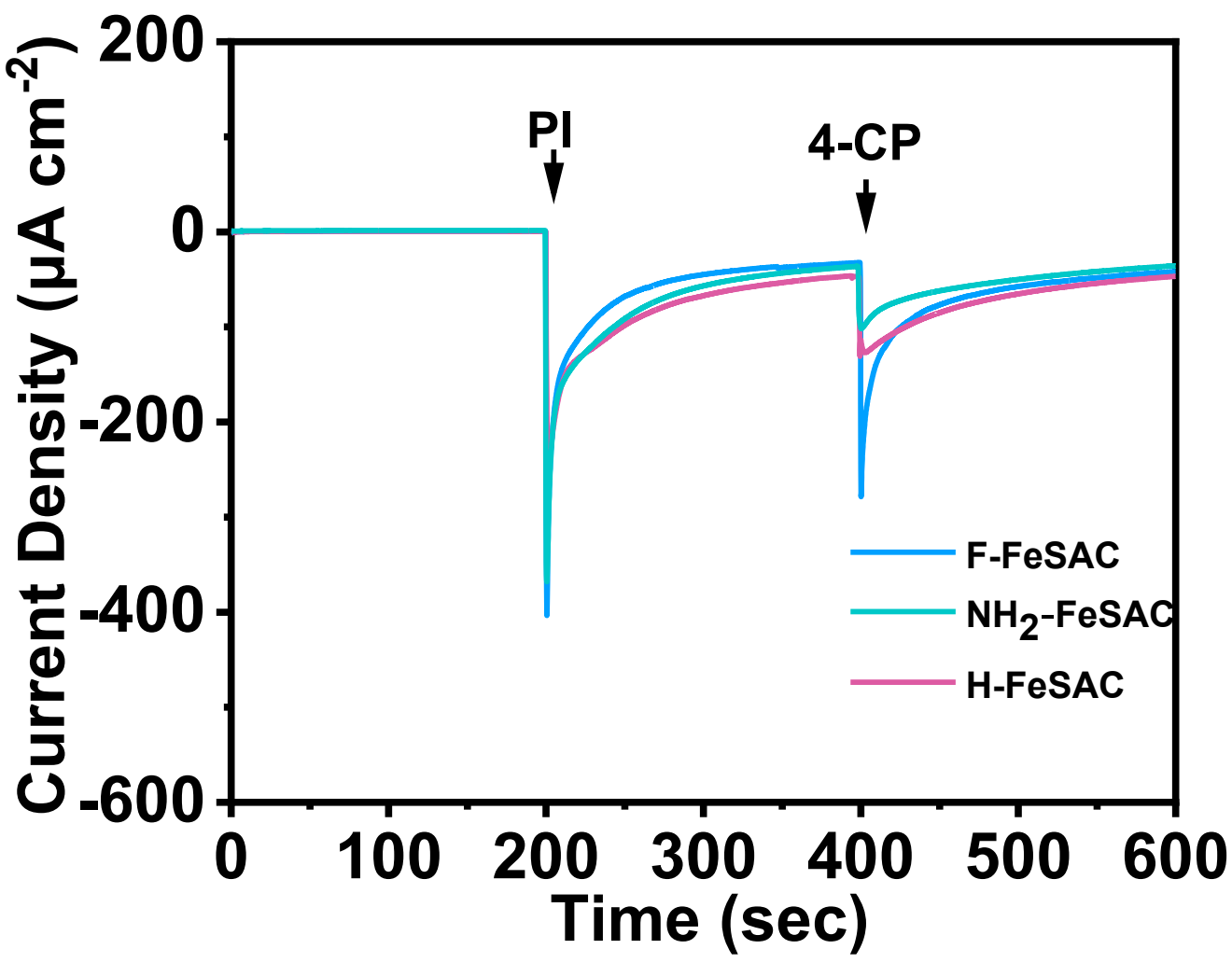

Supplementary Fig. 24 | Current-time responses to sequential reactant addition.

Current-time (i-t) curve of F-FeSAC, H-FeSAC and NH_2-FeSAC after sequential addition of PI and 4-CP. Source data are provided as a Source Data file.

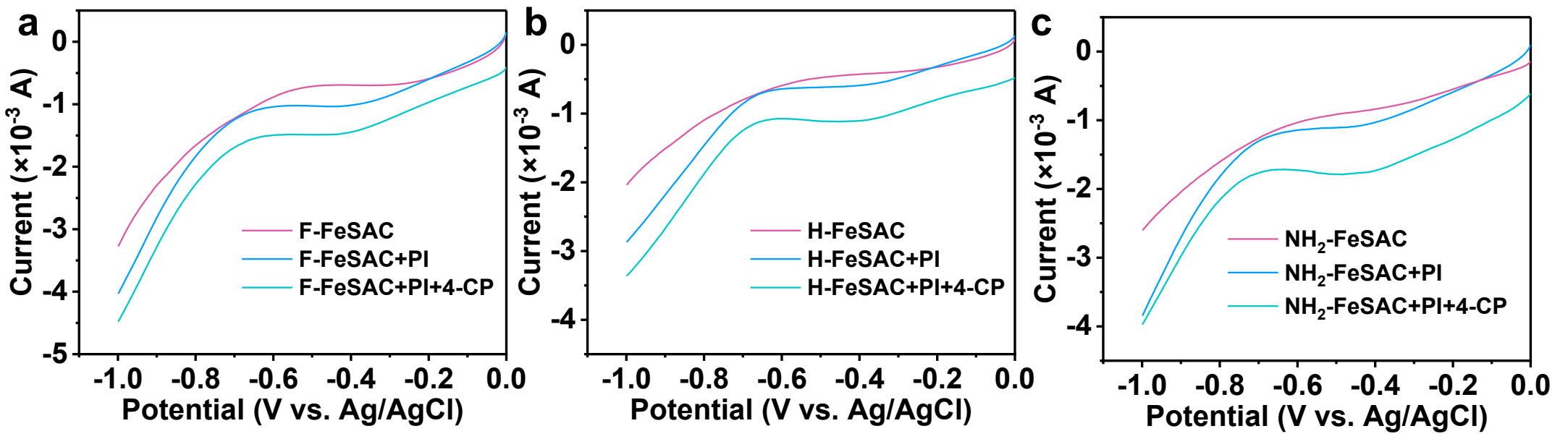

Supplementary Fig. 25 | Linear sweep voltammetry responses to sequential reactant addition. Linear sweep voltammetry (LSV) curve of (a) F-FeSAC, (b) H-FeSAC and (c) NH_2-FeSAC after sequential addition of PI and 4-CP. Source data are provided as a Source Data file.

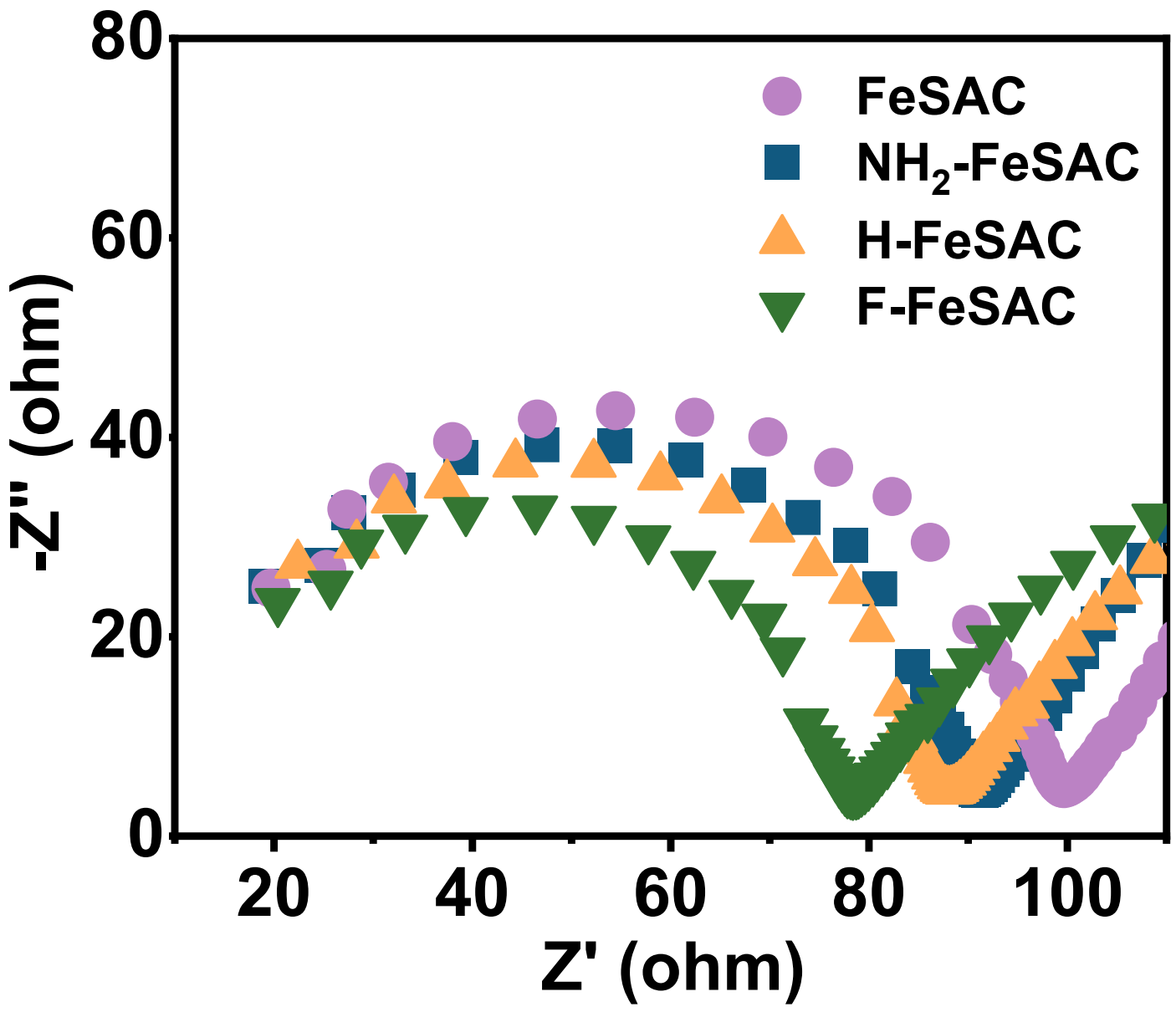

Supplementary Fig. 26 | Electrochemical impedance characteristics of FeSACs. Electrochemical impedance spectroscopy (EIS) spectra of FeSAC, F-FeSAC, H-FeSAC and NH_2-FeSAC. Source data are provided as a Source Data file.

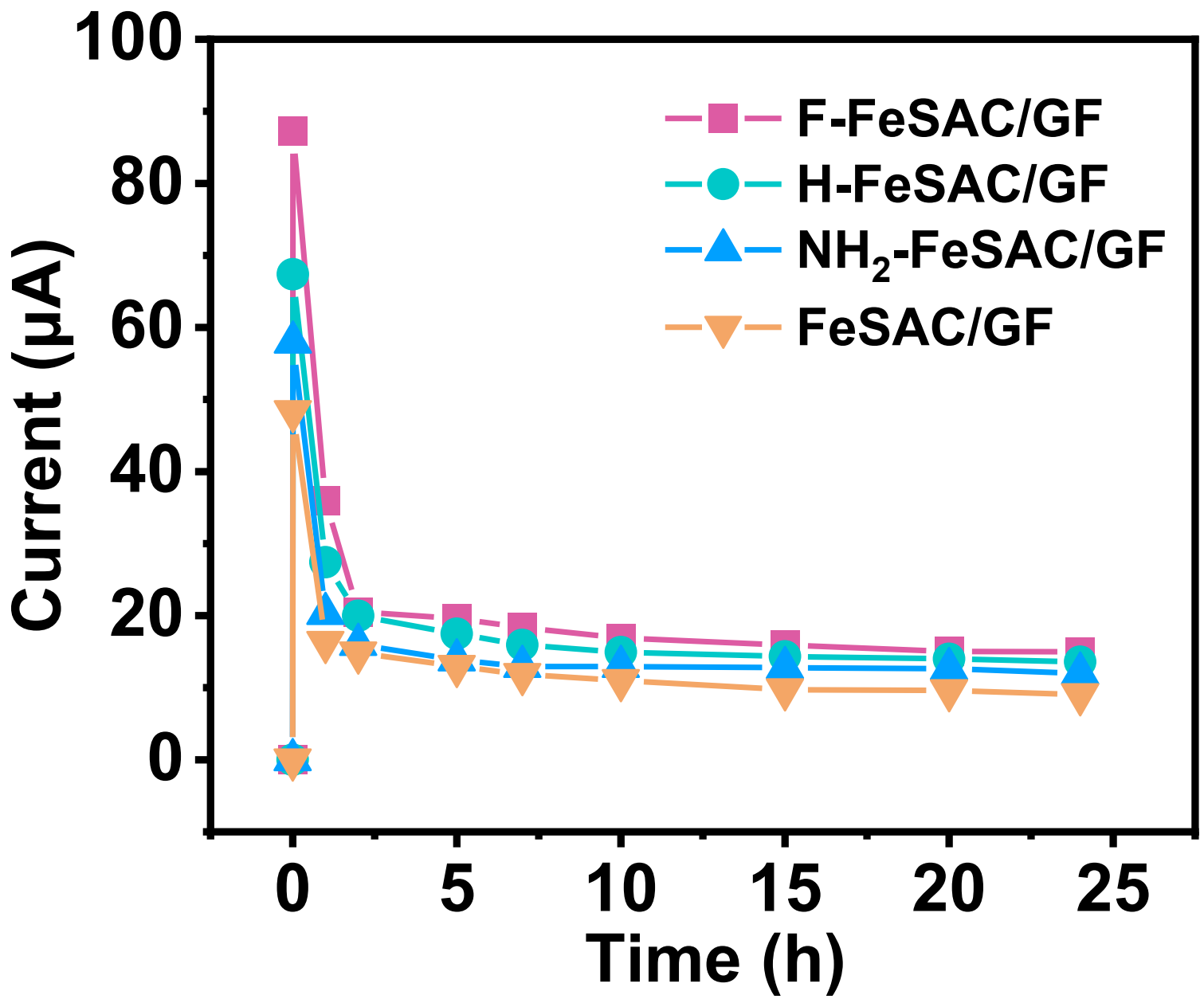

Supplementary Fig. 27 | Current-time responses of FeSAC/GF electrodes. Current-time curves of FeSAC/GF, F-FeSAC/GF, H-FeSAC/GF and NH_2-FeSAC/GF electrodes, where GF denotes graphite felt. Source data are provided as a Source Data file.

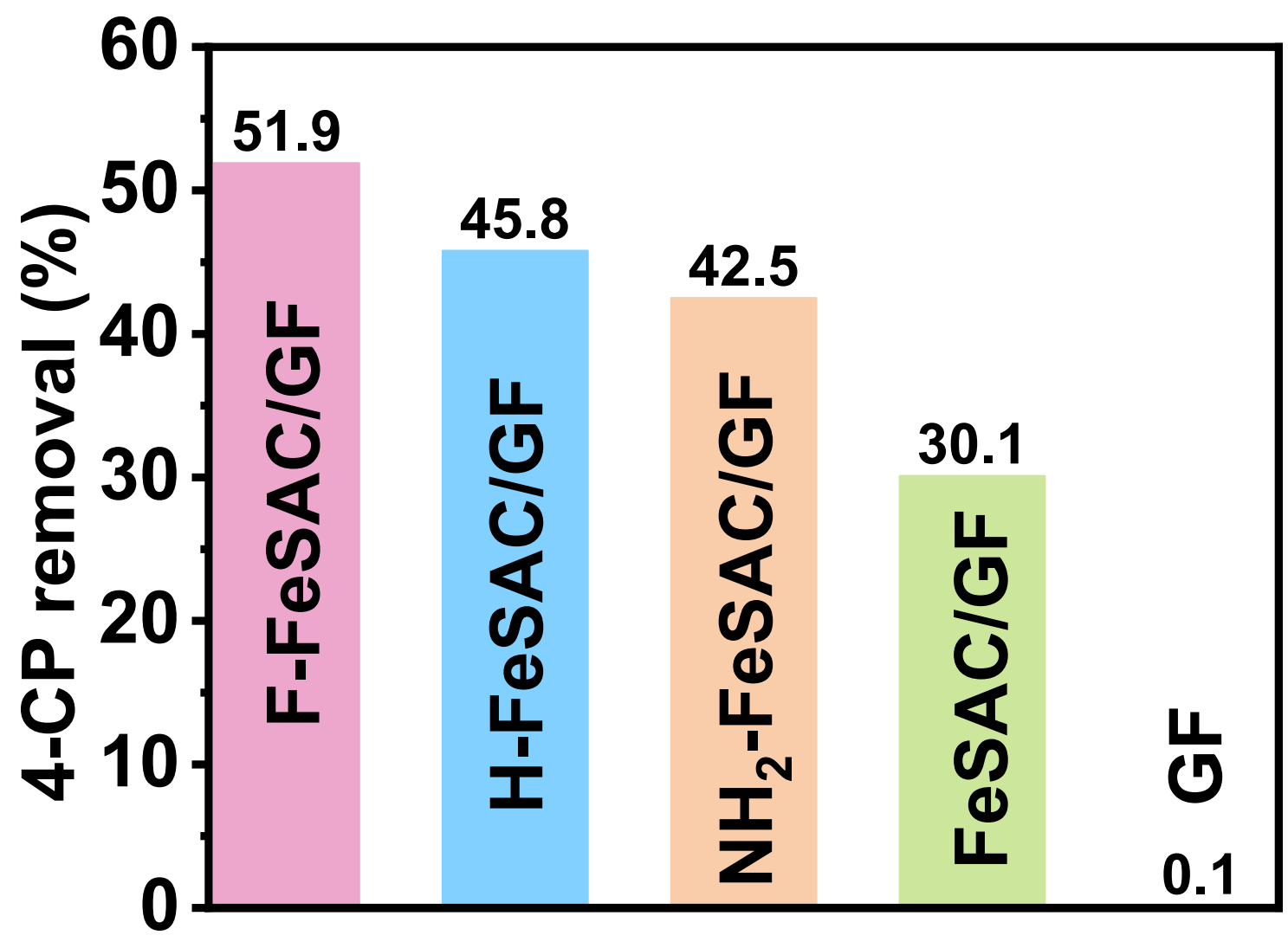

Supplementary Fig. 28 | 4-CP removal in different GOP systems. Removal efficiencies of 4-CP in different galvanic oxidation process (GOP) systems. Source data are provided as a Source Data file.

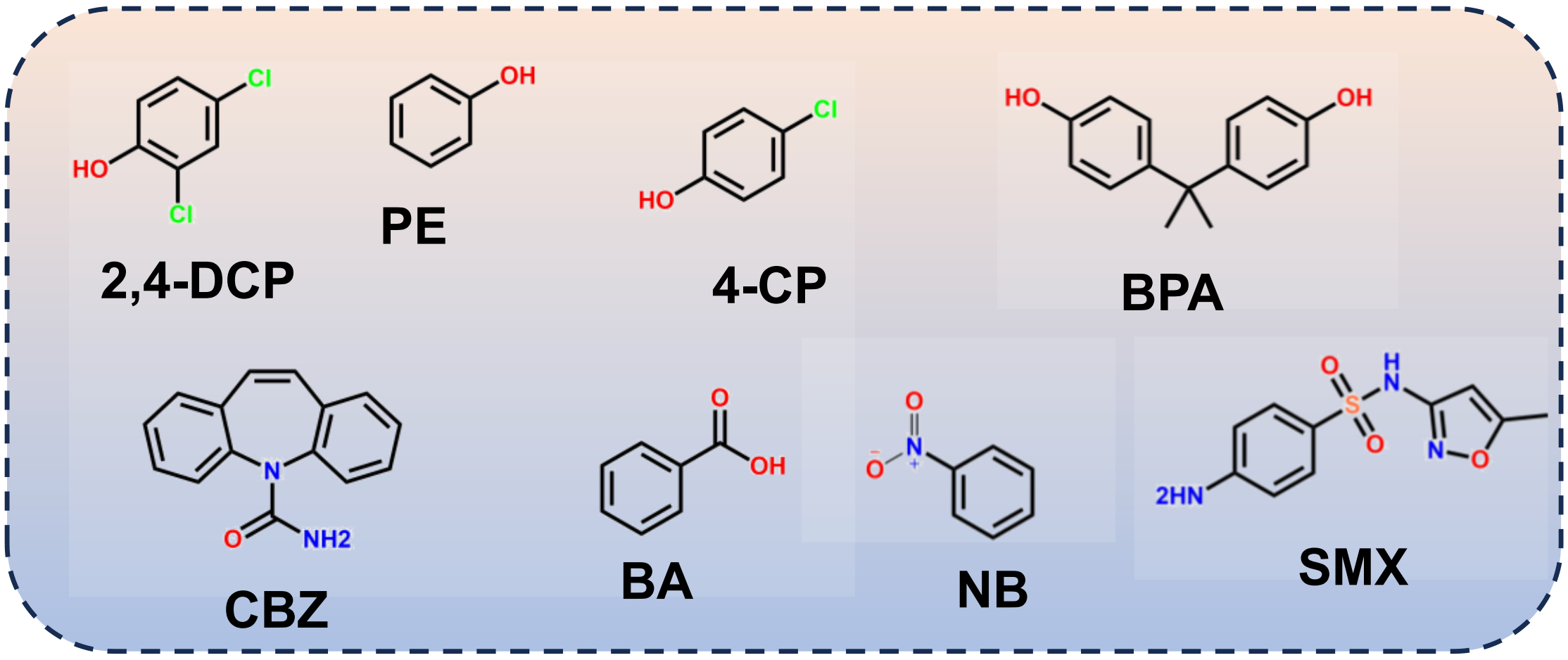

Supplementary Fig. 29 | Molecular structures of target pollutants. Molecular structure diagrams of target pollutants.

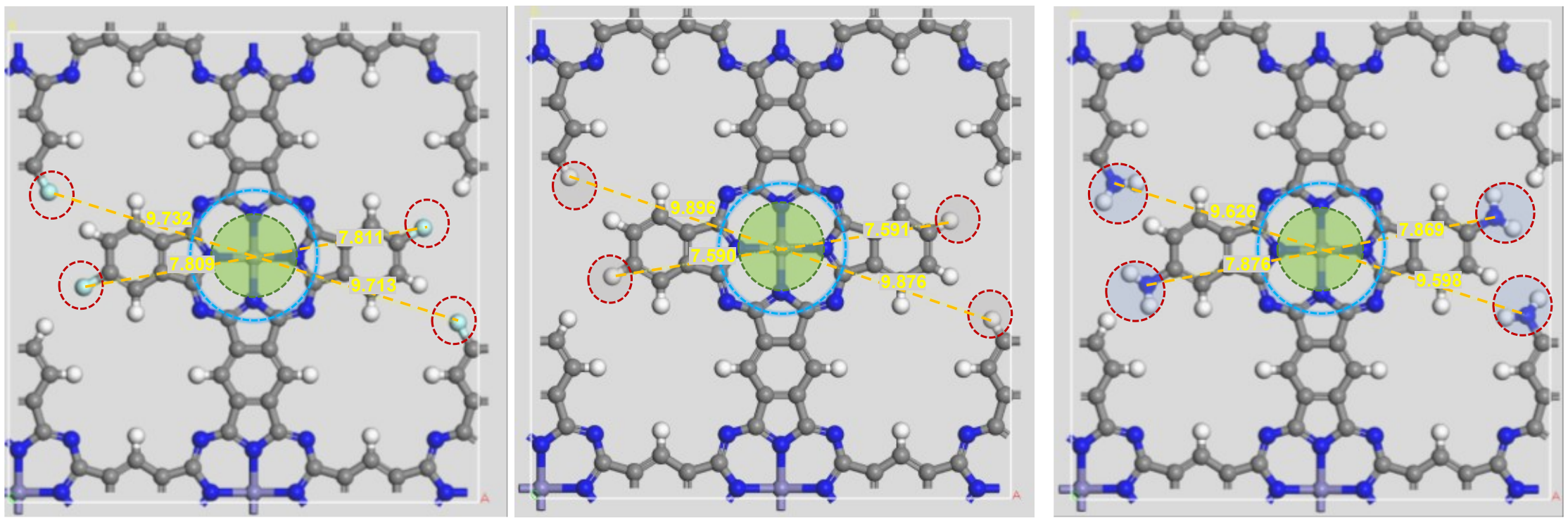

F-FeSAC H-FeSAC NH_2-FeSAC

Supplementary Fig. 30 | Spatial separation of Fe-N_4 cores and peripheral substituents. Structural models illustrating the spatial separation between the Fe–N_4 core and substituent groups. C, H, N, F and Fe atoms are shown in grey, white, blue, cyan and lavender, respectively. Red dashed circles highlight the peripheral substituents, and yellow dashed lines indicate the distances between the Fe centre and the substituents. The green shaded circle indicates the region within the first coordination sphere of Fe, whereas the blue dashed circle denotes the second coordination sphere. Crystal structures visualized using VESTA software[1].

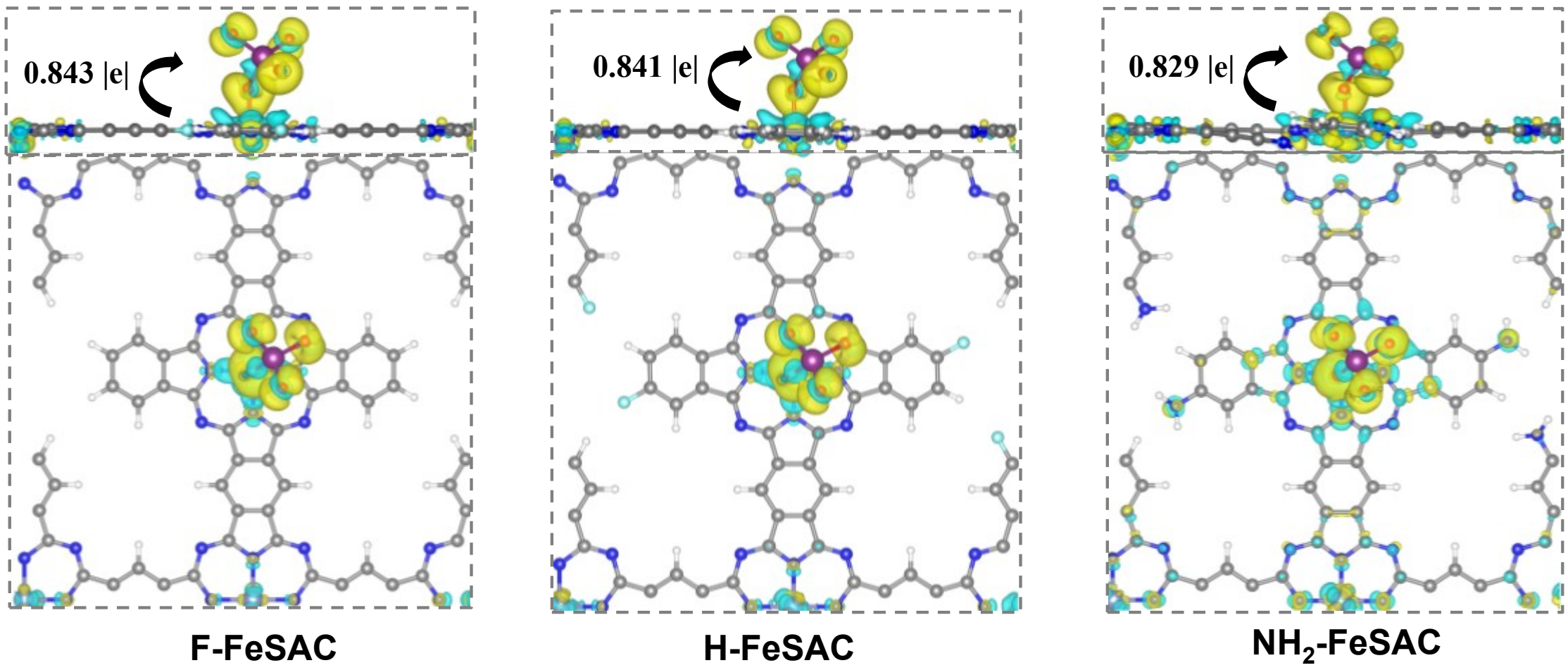

Supplementary Fig. 31 | Charge redistribution in catalyst-PI complexes. Differential charge density analysis of catalyst–PI complexes for F-FeSAC, H-FeSAC, and NH_2-FeSAC. C, H, N, O, F, Fe and I atoms are shown in grey, white, blue, red, cyan, lavender and purple, respectively. Yellow and cyan isosurfaces indicate electron accumulation and depletion, respectively, and the black arrows indicate the direction of electron transfer. Crystal structures visualized using VESTA software[1].

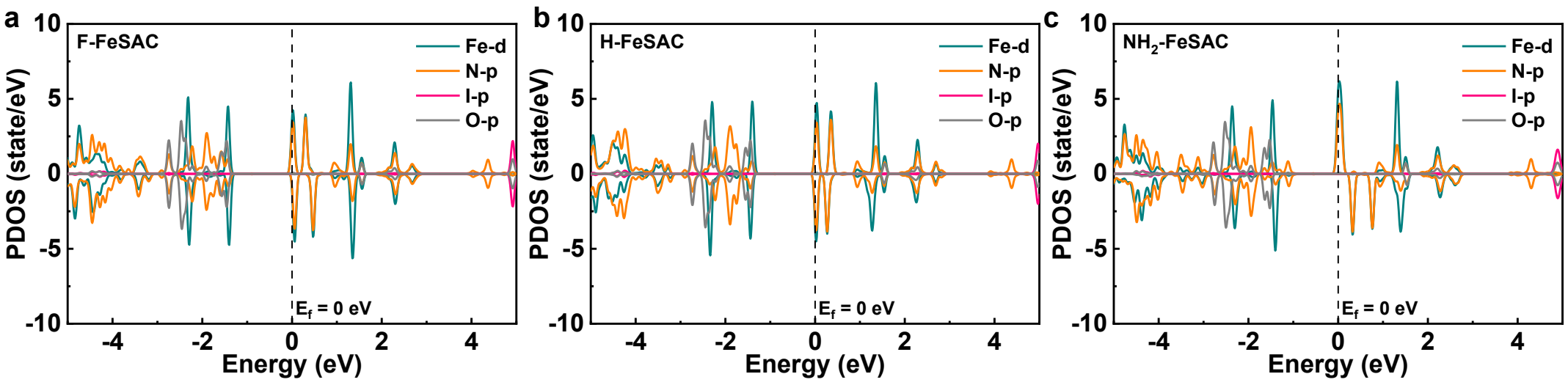

Supplementary Fig. 32 | Electronic structures of PI-adsorbed FeSACs. Projected density of states (PDOS) of PI-adsorbed (a) F-FeSAC, (b) H-FeSAC, and (c) NH_2-FeSAC. Source data are provided as a Source Data file.

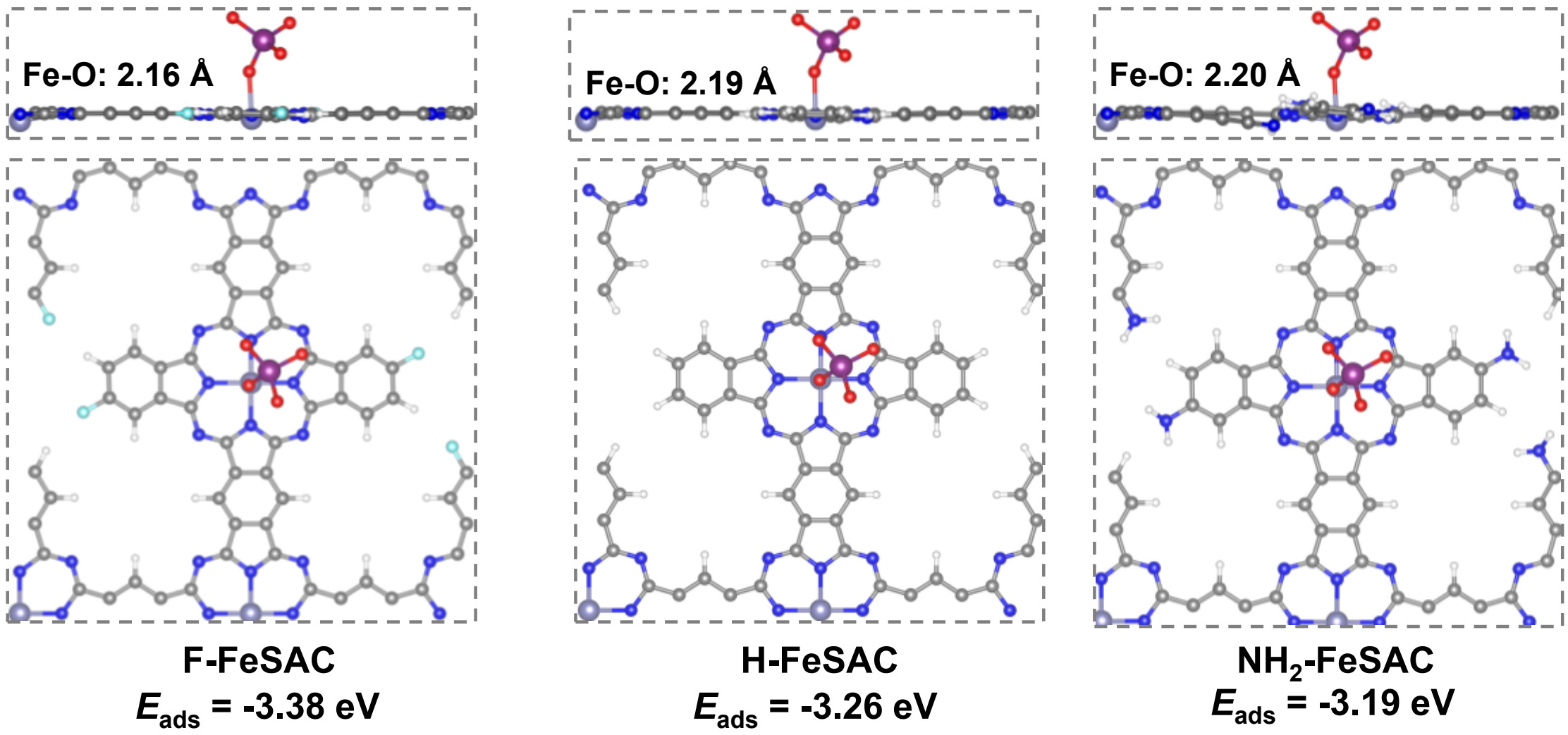

Supplementary Fig. 33 | PI adsorption configurations on substituted FeSACs. Optimized adsorption configurations of PI on F-FeSAC, H-FeSAC, and NH_2-FeSAC. C, H, N, O, F, Fe and I atoms are shown in grey, white, blue, red, cyan, lavender and purple, respectively. Crystal structures visualized using VESTA software[1].

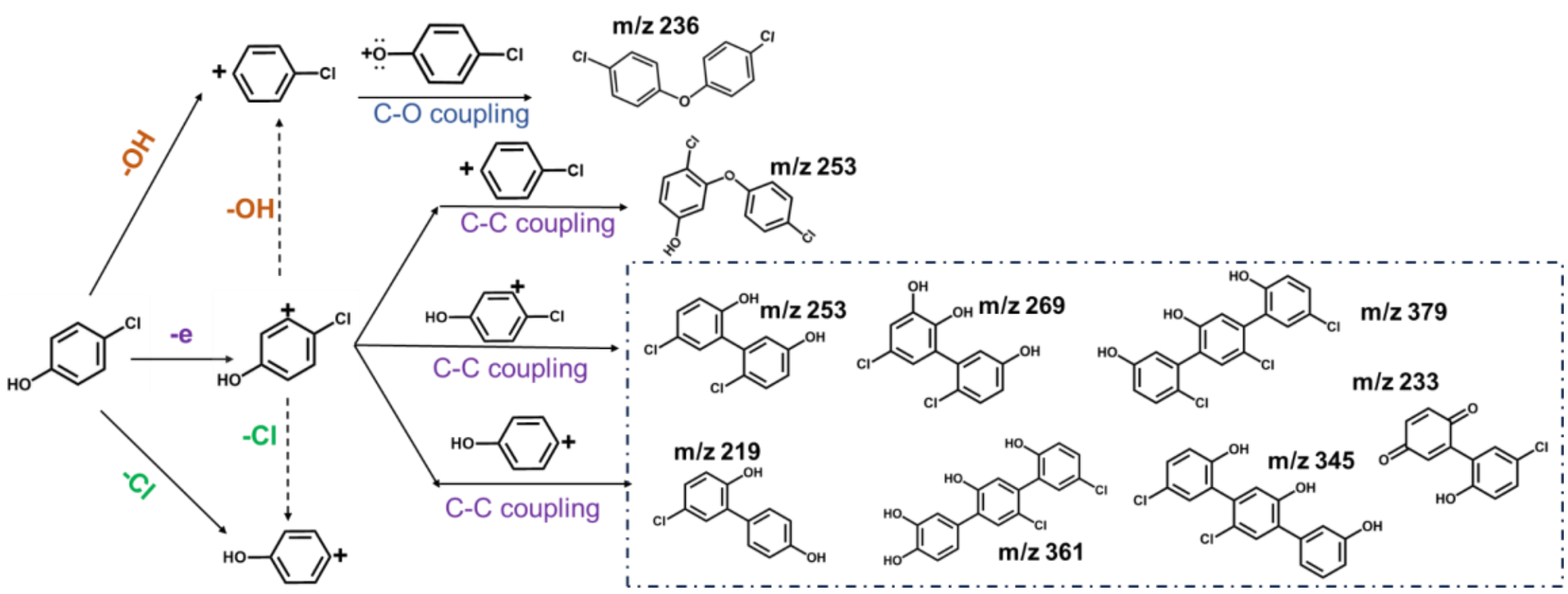

Supplementary Fig. 34 | Transformation pathways of 4-CP. Possible transformation pathways of 4-CP in the F-FeSAC/PI system.

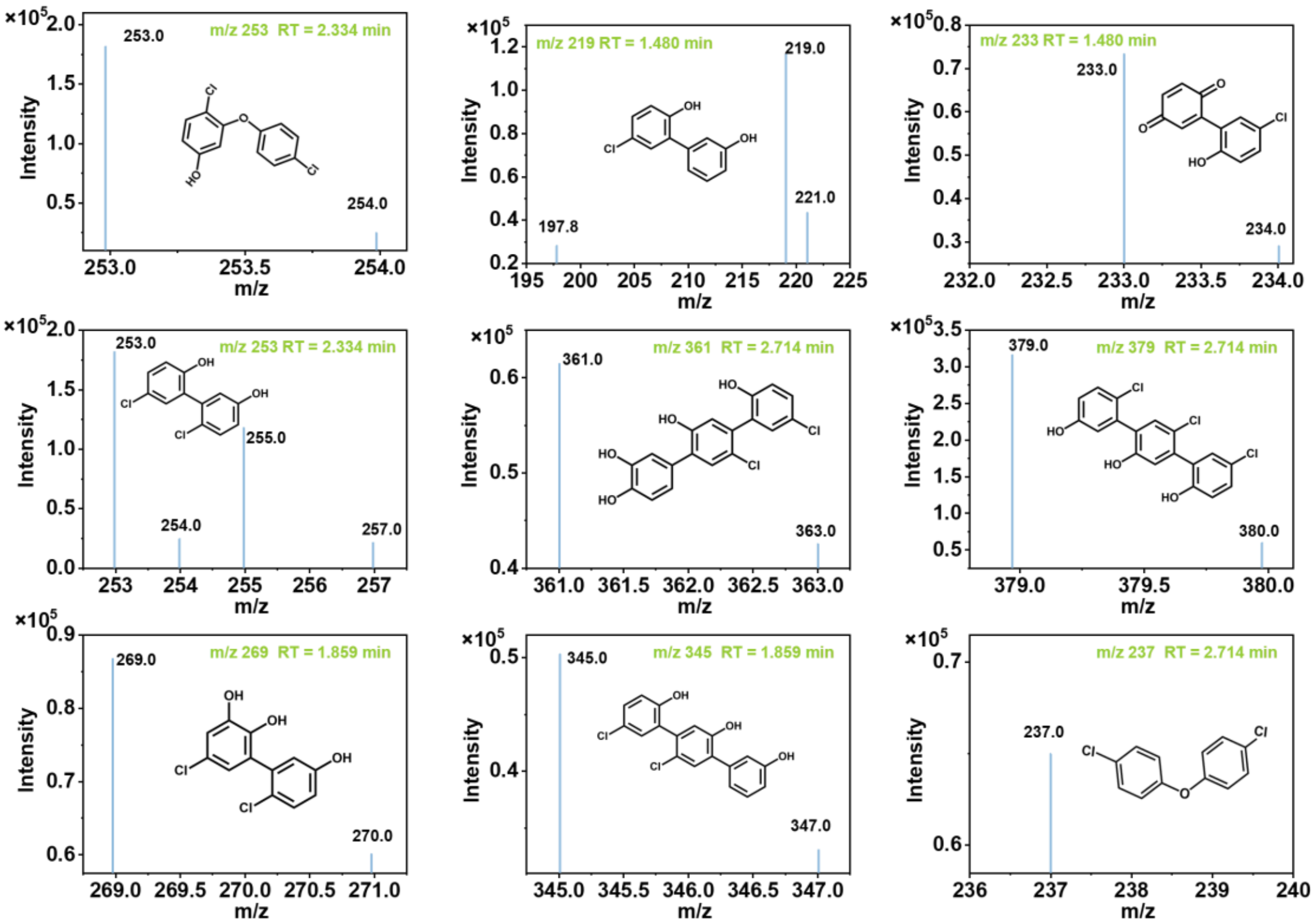

Supplementary Fig. 35 | Mass-spectrometric detection of 4-CP transformation products. Electrospray ionization (ESI) mass spectra of 4-CP during reaction in the F-FeSAC/PI system. Source data are provided as a Source Data file.

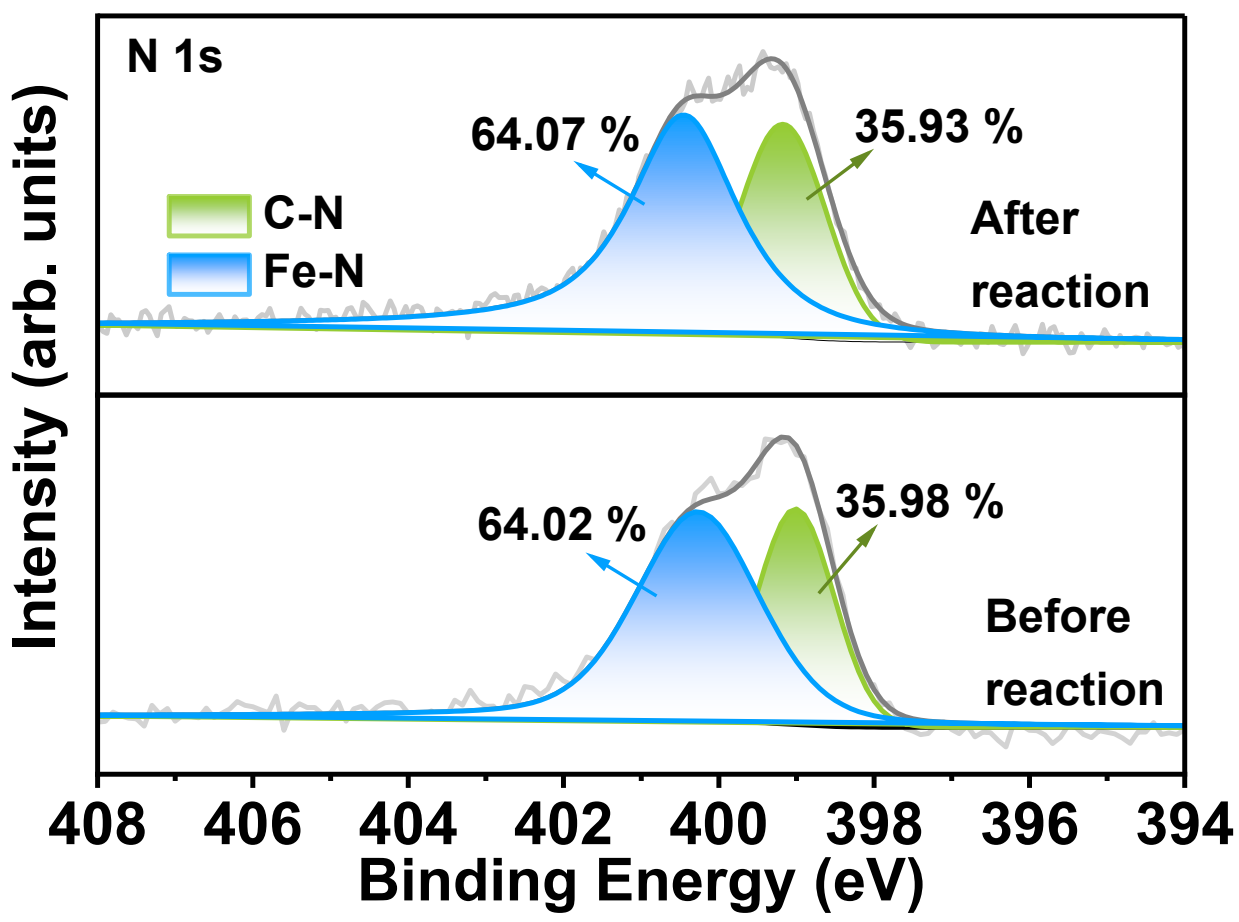

Supplementary Fig. 36 | N 1s chemical states of F-FeSAC before and after reaction.

XPS N 1*s* spectra of F-FeSAC before and after reaction. Routine conditions: [4-CP] = 80 μM, [catalyst] = 50 mg L^{-1}, [PI] = 0.20 mM, temperature = 25 °C. Source data are provided as a Source Data file.

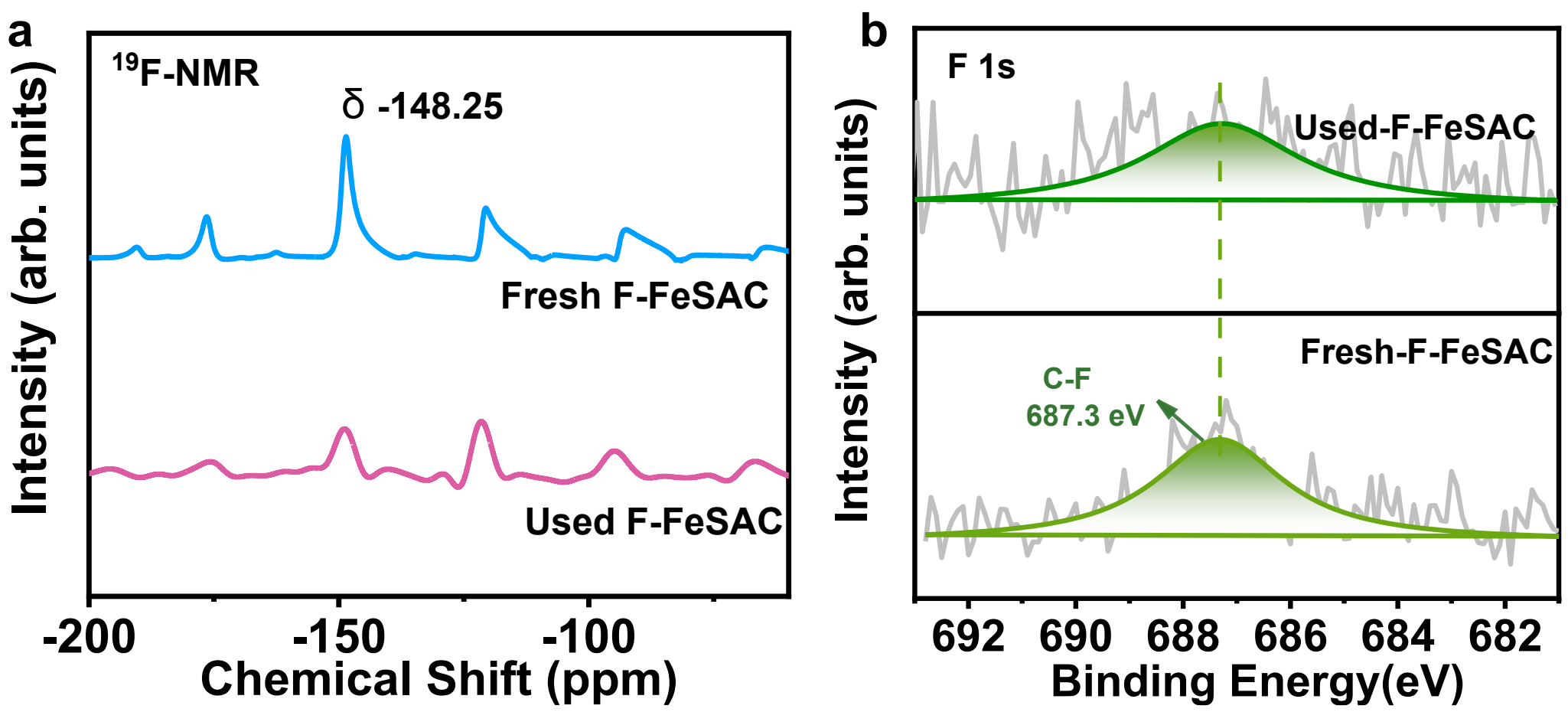

Supplementary Fig. 37 | Surface chemistry of fresh and used F-FeSAC. (a) ^{19}F solid-state NMR spectroscopy and (b) XPS F 1*s* spectra of fresh and used F-FeSAC. Routine conditions: [4-CP] = 80 μM, [catalyst] = 50 mg L^{-1}, [PI] = 0.20 mM, temperature = 25 °C. Source data are provided as a Source Data file.

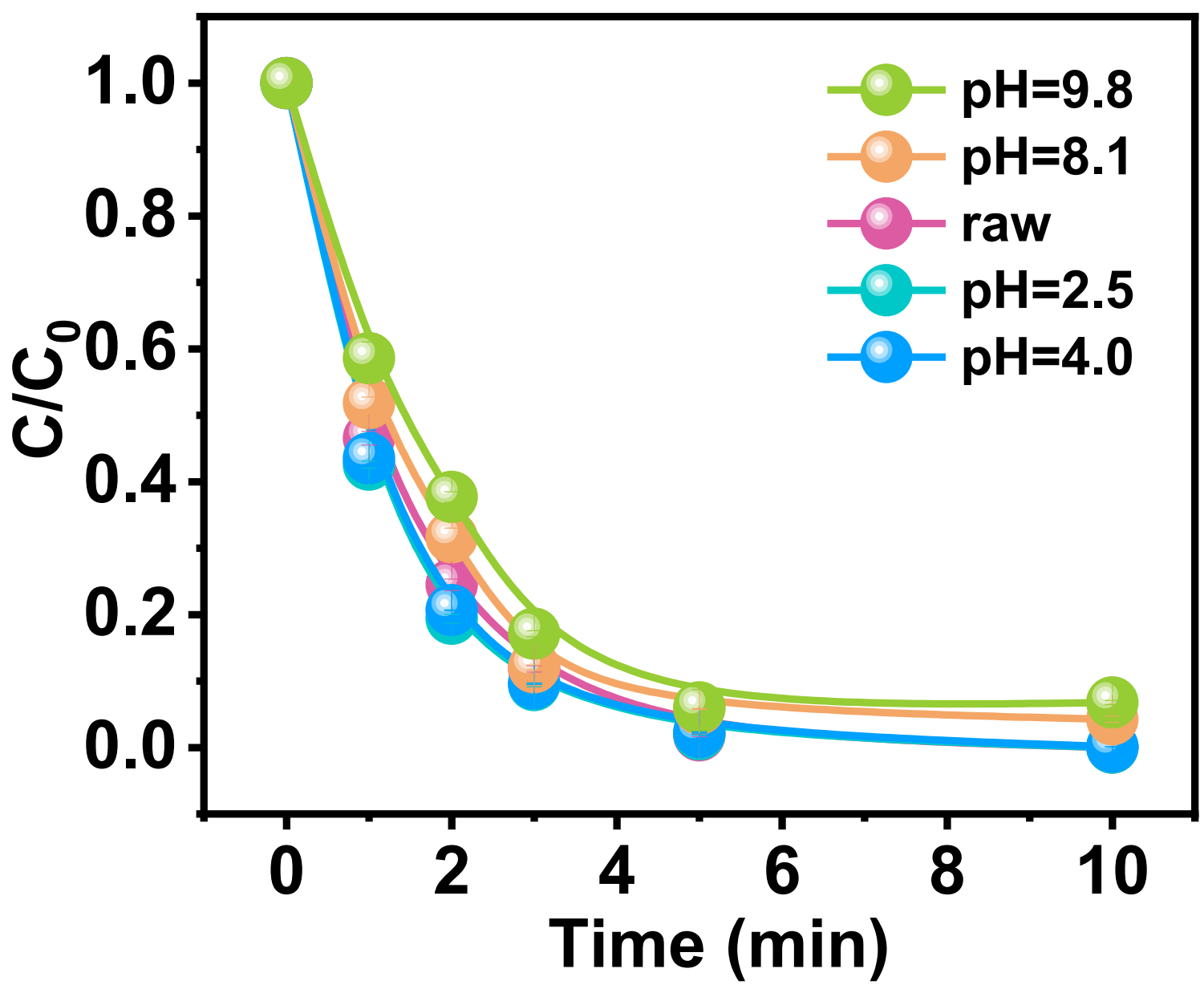

Supplementary Fig. 38 | Effect of pH on 4-CP removal. Effects of pH on the removal of 4-CP in F-FeSAC/PI system. Routine conditions: [4-CP] = 80 μM, [catalyst] = 50 mg L^{-1}, [PI] = 0.20 mM, temperature = 25 °C. Data are presented as mean values ± SD from three experiments (n = 3). Source data are provided as a Source Data file.

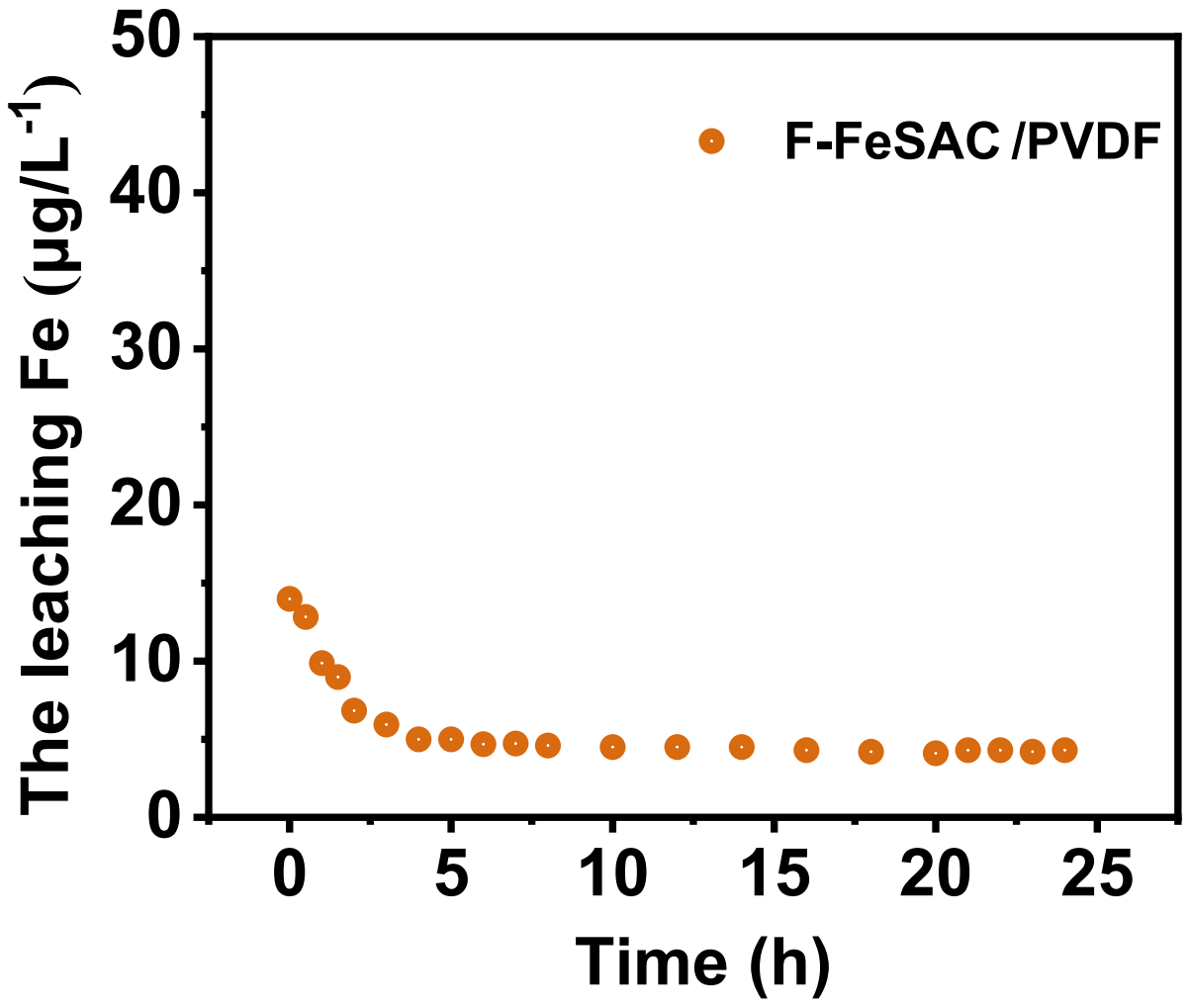

Supplementary Fig. 39 | Fe leaching from the F-FeSAC/PVDF membrane. Fe leaching from the F-FeSAC/PVDF membrane system. Routine conditions: [4-CP] = 80 μM, [catalyst] = 0.5 g L^{-1}, [PI] = 0.20 mM, temperature = 25 °C, without pH adjustment. Source data are provided as a Source Data file.

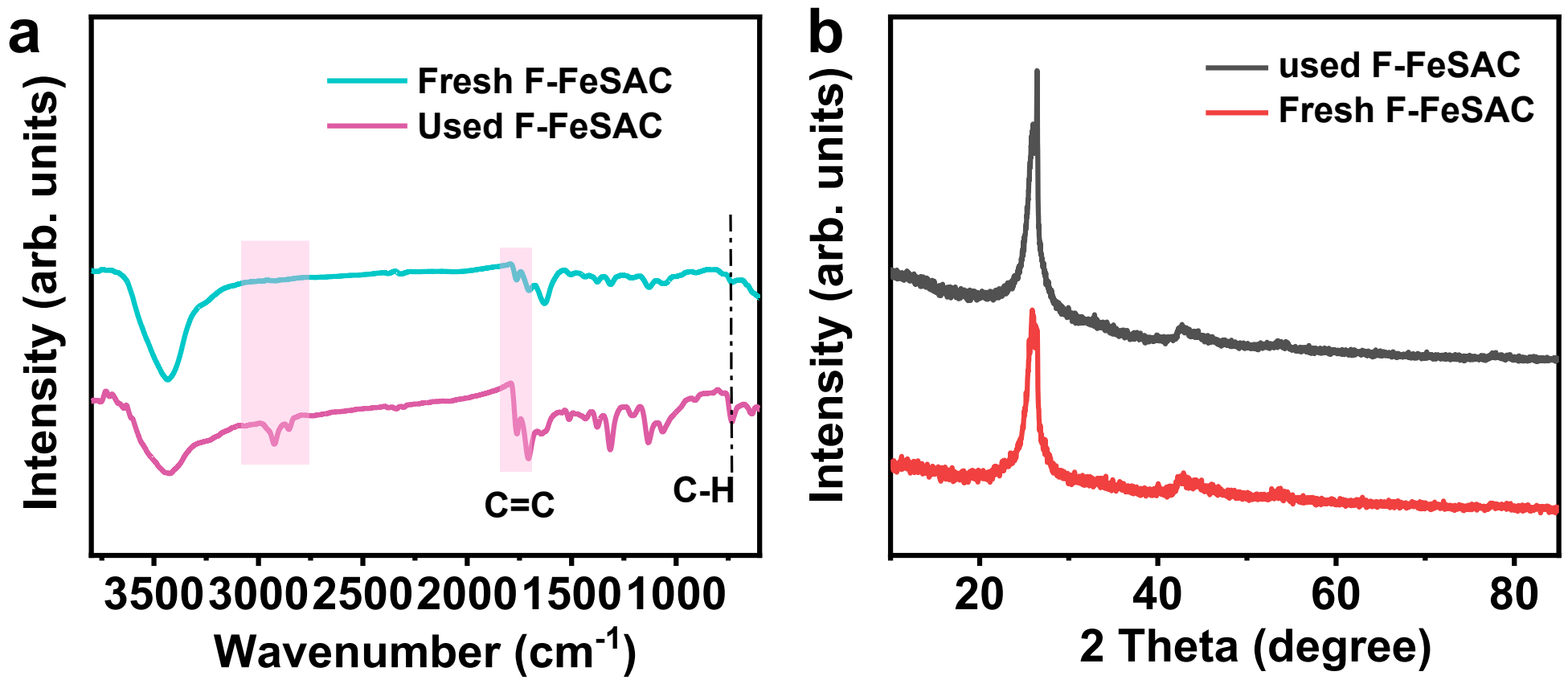

Supplementary Fig. 40 | Structural characterization of F-FeSAC before and after use. (a) FTIR and (b) XRD spectra of F-FeSAC before and after use. The red shaded region indicates the position of the C=C band. Routine conditions: [4-CP] = 80 μM, [catalyst] = 0.5 g L^{-1}, [PI] = 0.20 mM, temperature = 25 °C. Source data are provided as a Source Data file.

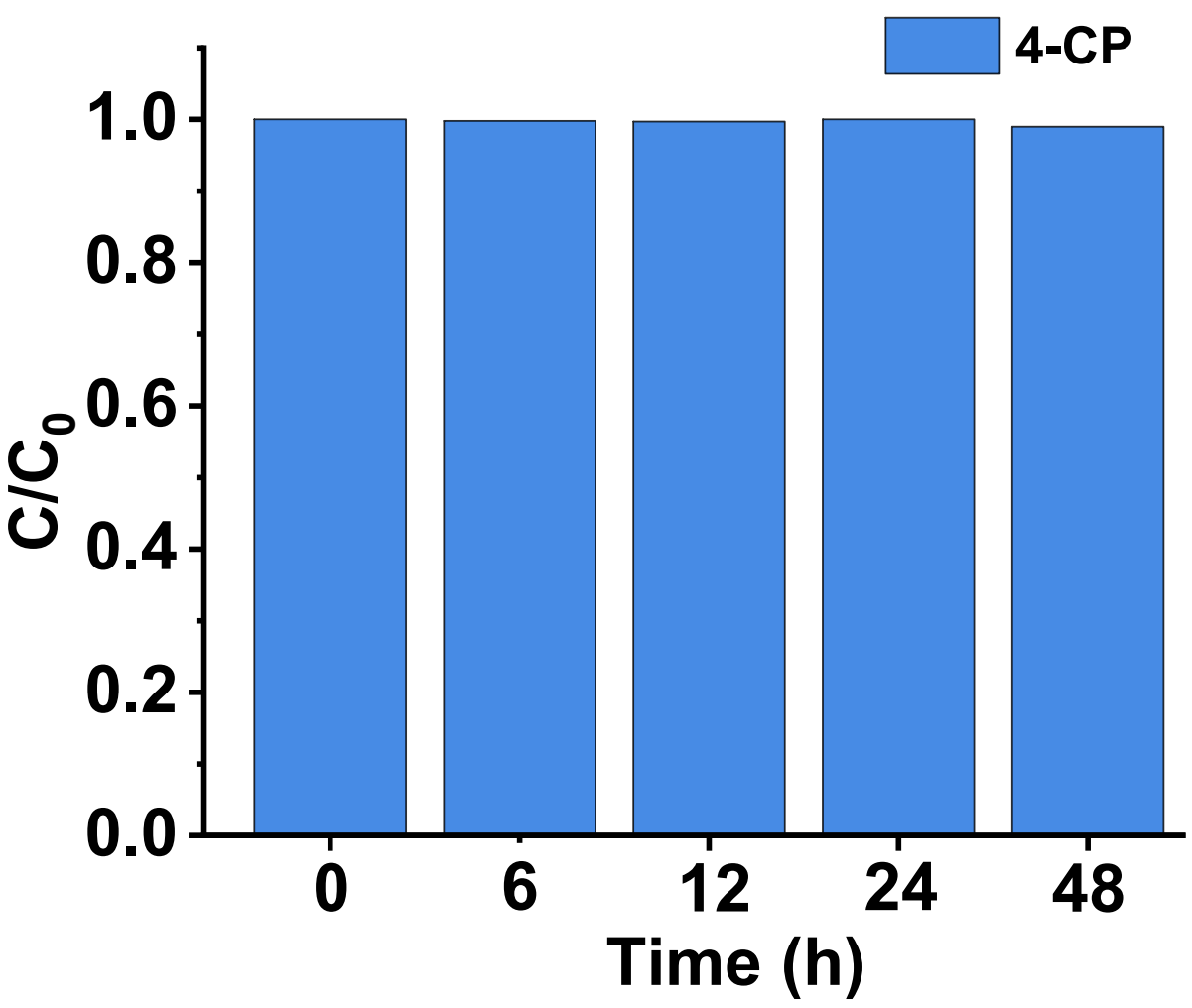

Supplementary Fig. 41 | 4-CP removal using bare electrodes in the pilot-scale reactor. 4-CP removal in the pilot-scale electron-transfer reactor under bare-electrode conditions. Routine conditions: [4-CP] = 80 μM, [PI] = 1.0 mM, temperature = 25 °C, without pH adjustment. Source data are provided as a Source Data file.

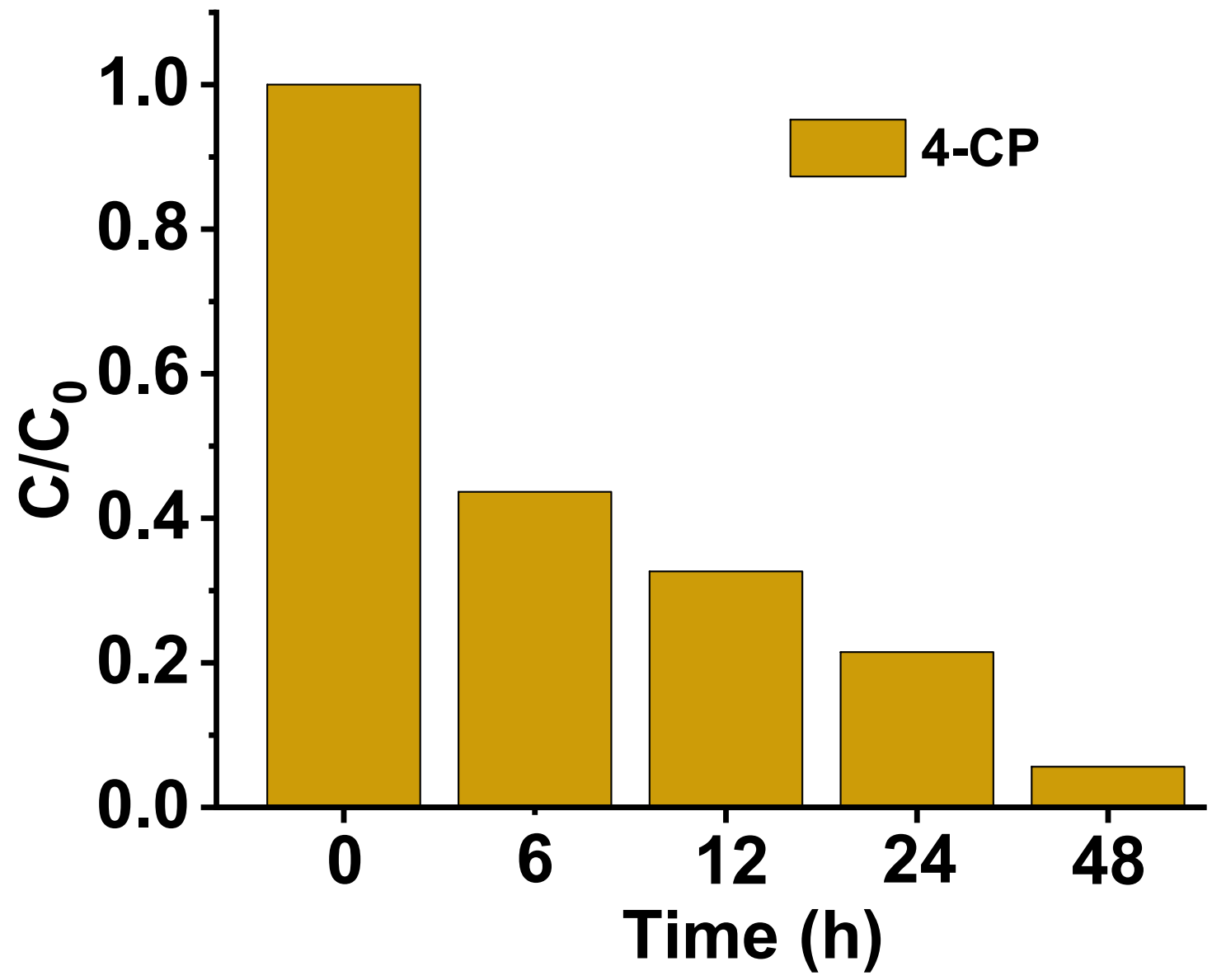

Supplementary Fig. 42 | 4-CP removal using eight electrodes in the pilot-scale reactor. 4-CP removal efficiency in the pilot-scale electron-transfer reactor with eight electrodes. Routine conditions: [4-CP] = 80 μM, [PI] = 1.0 mM, temperature = 25 °C, without pH adjustment. Source data are provided as a Source Data file.

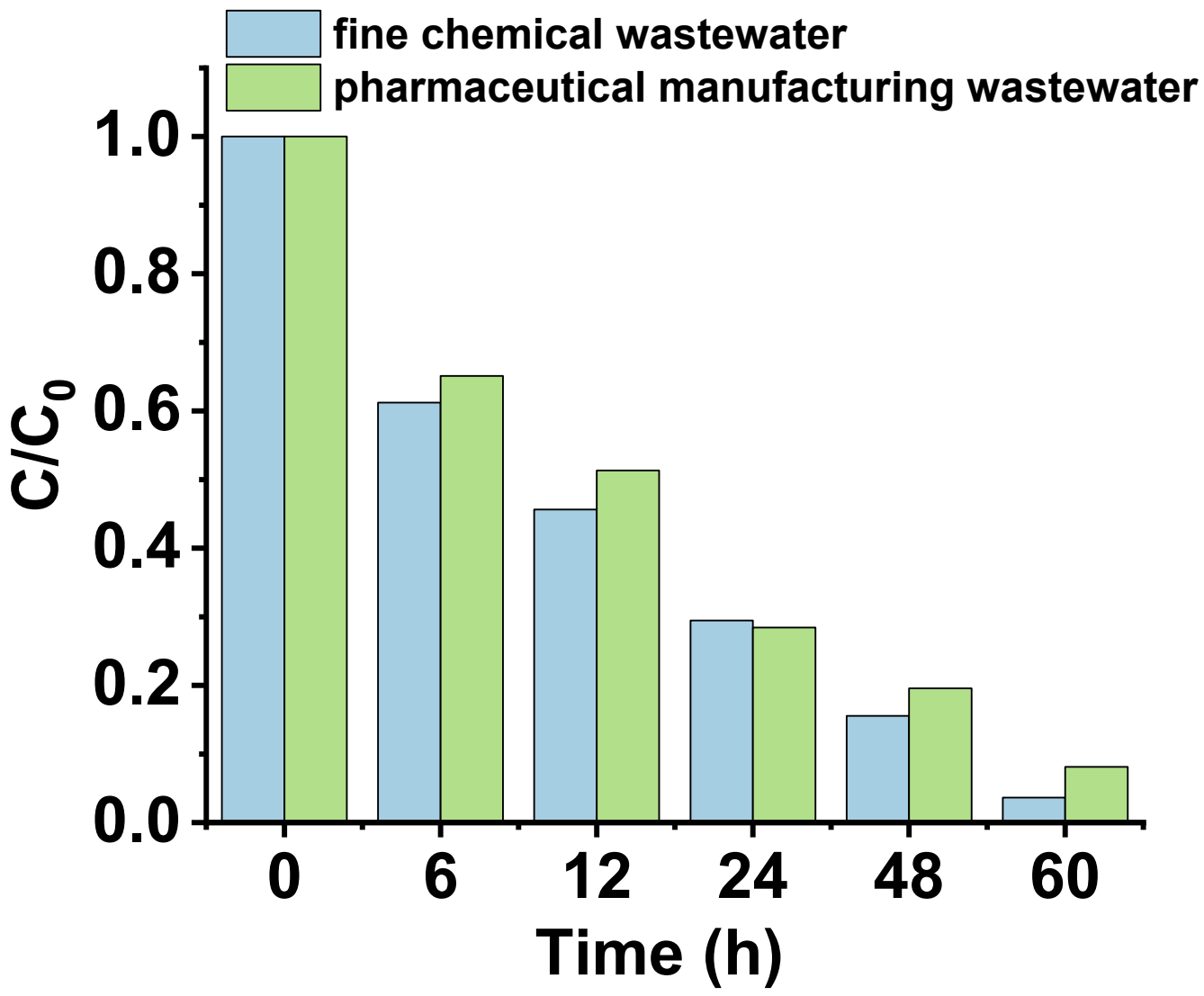

Supplementary Fig. 43 | Treatment of real WWTP effluents in the pilot-scale reactor. 4-CP removal in the pilot-scale electron-transfer reactor using effluents from two wastewater treatment plants (WWTPs). Routine conditions: [4-CP] = 80 μM, [PI] = 1.0 mM, temperature = 25 °C, without pH adjustment. Source data are provided as a Source Data file.

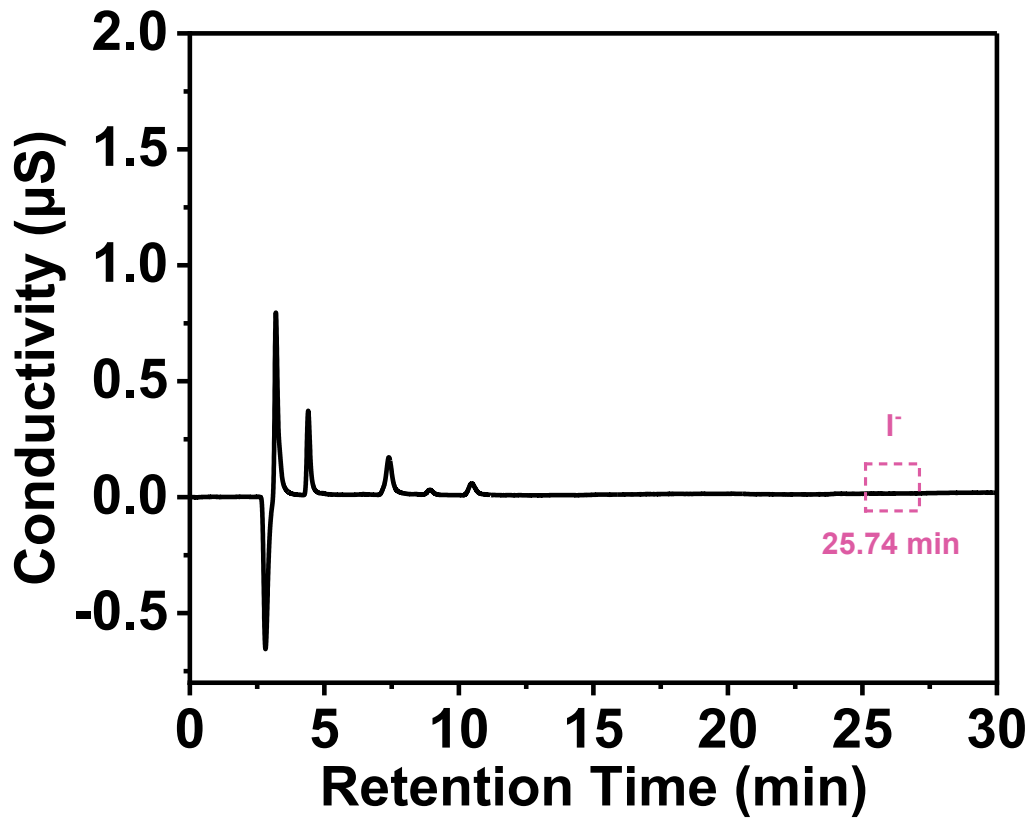

Supplementary Fig. 44 | Ion-chromatographic detection of iodide. Ion chromatography (IC) chromatogram for iodide detection. The red dashed box indicates the peak position of iodide ions. Source data are provided as a Source Data file.

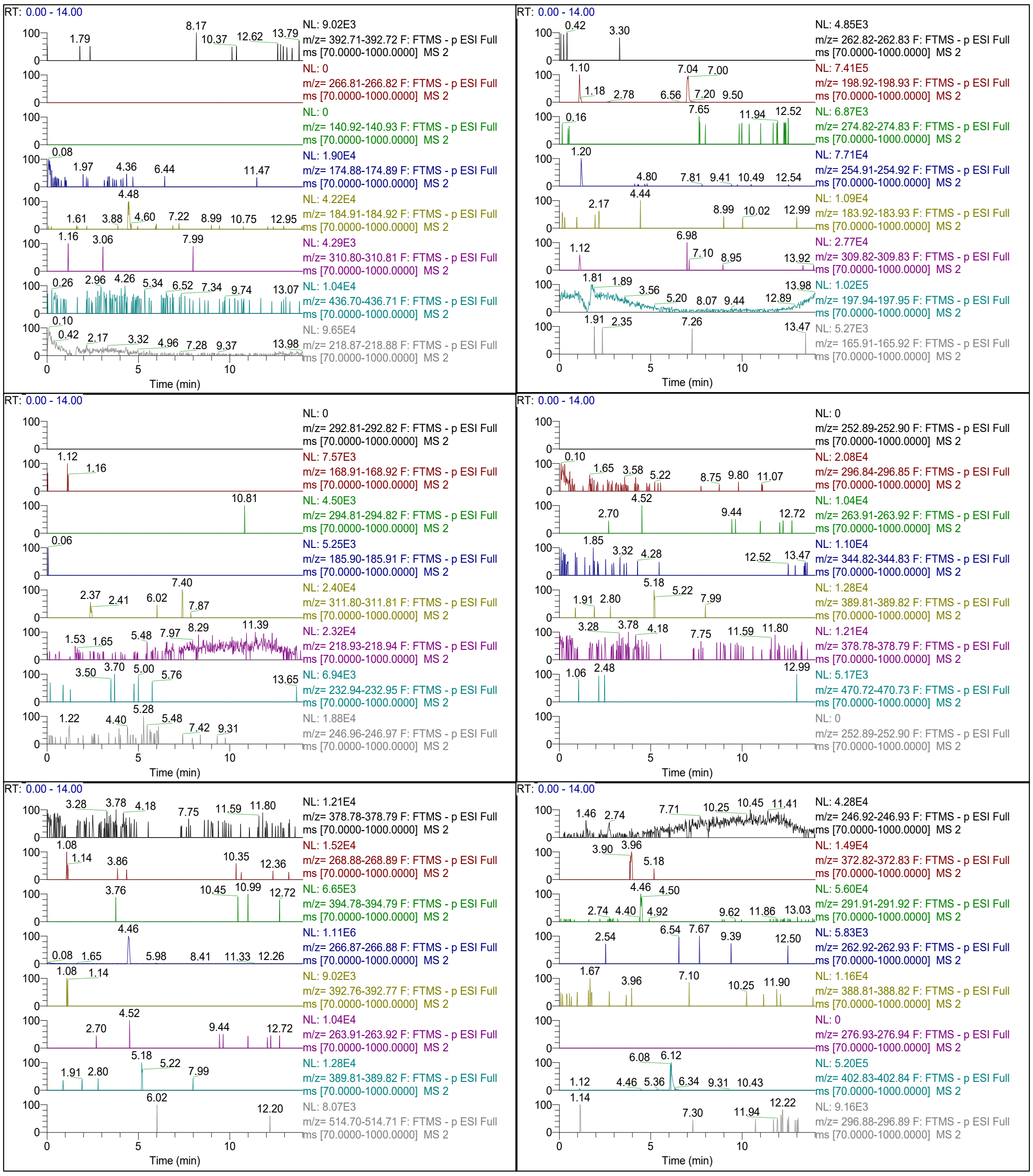

Supplementary Fig. 45 | Extracted-ion chromatograms of suspected iodinated byproducts (Part I). Representative extracted ion chromatograms (EICs) of suspected iodinated byproduct candidates (Part I). The molecular formula and mass-to-charge ratio (*m/z*) value of each candidate are indicated above the corresponding chromatogram. Source data are provided as a Source Data file.

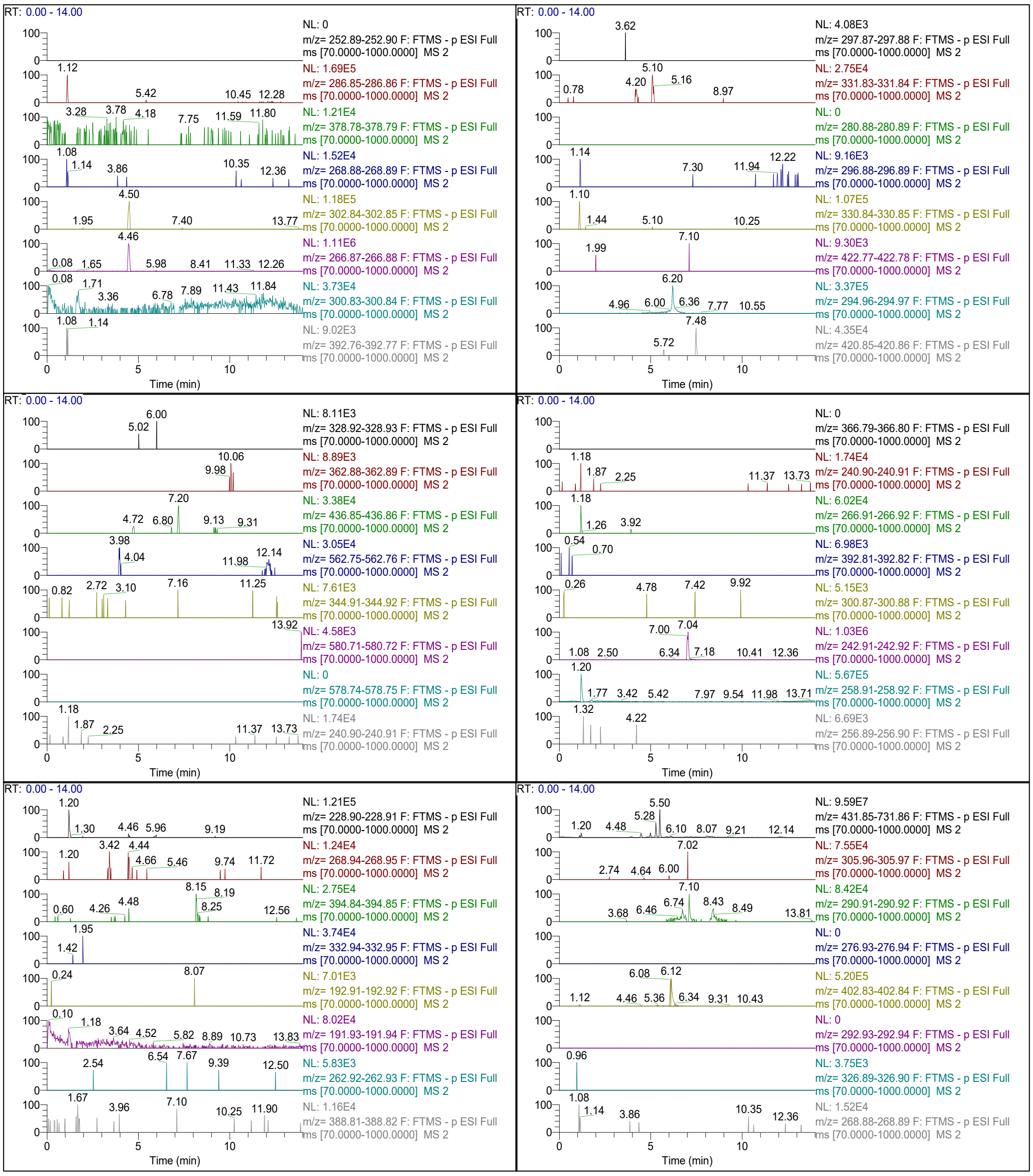

Supplementary Fig. 46 | Extracted-ion chromatograms of suspected iodinated byproducts (Part II). Representative EICs of suspected iodinated byproduct candidates (Part II). The molecular formula and *m/z* value of each candidate are indicated above the corresponding chromatogram. Source data are provided as a Source Data file.

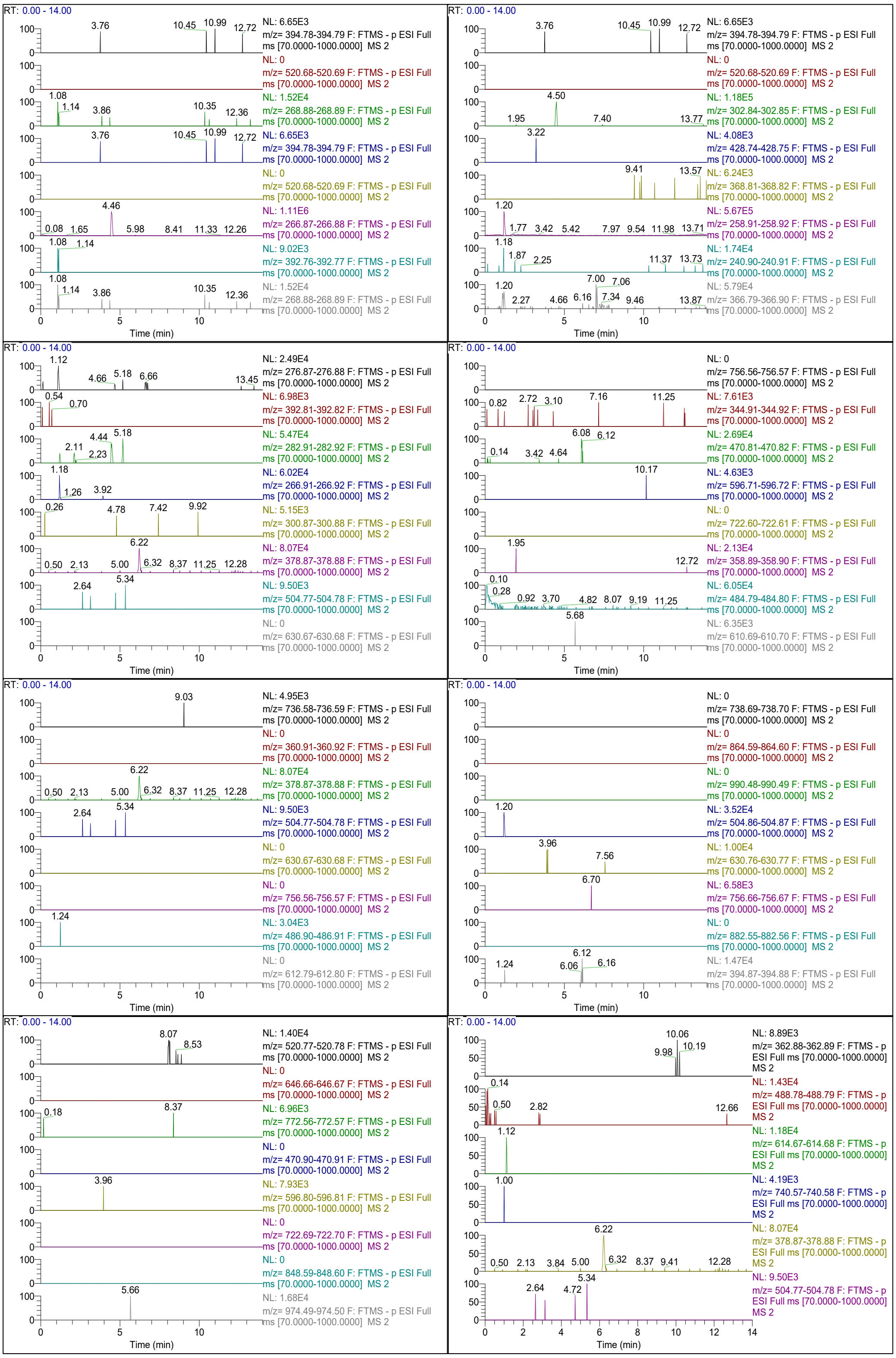

Supplementary Fig. 47 | Extracted-ion chromatograms of suspected iodinated byproducts (Part III). Representative EICs of suspected iodinated byproduct candidates (Part III). The molecular formula and *m/z* value of each candidate are indicated above the corresponding chromatogram. Source data are provided as a Source Data file.

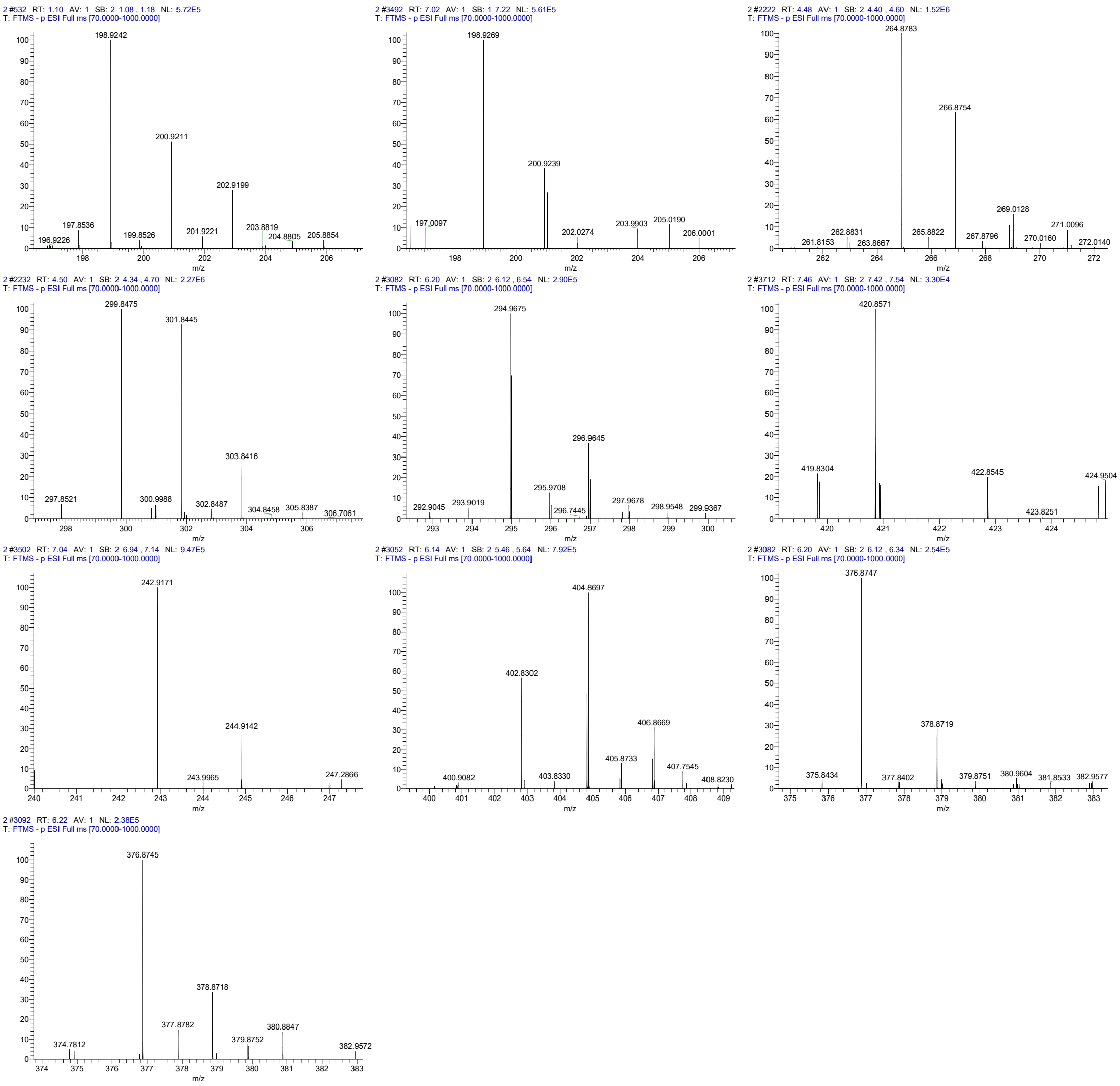

Supplementary Fig. 48 | Mass spectra of suspected iodinated byproducts. Mass spectra of suspected iodinated byproduct candidates. Source data are provided as a Source Data file.

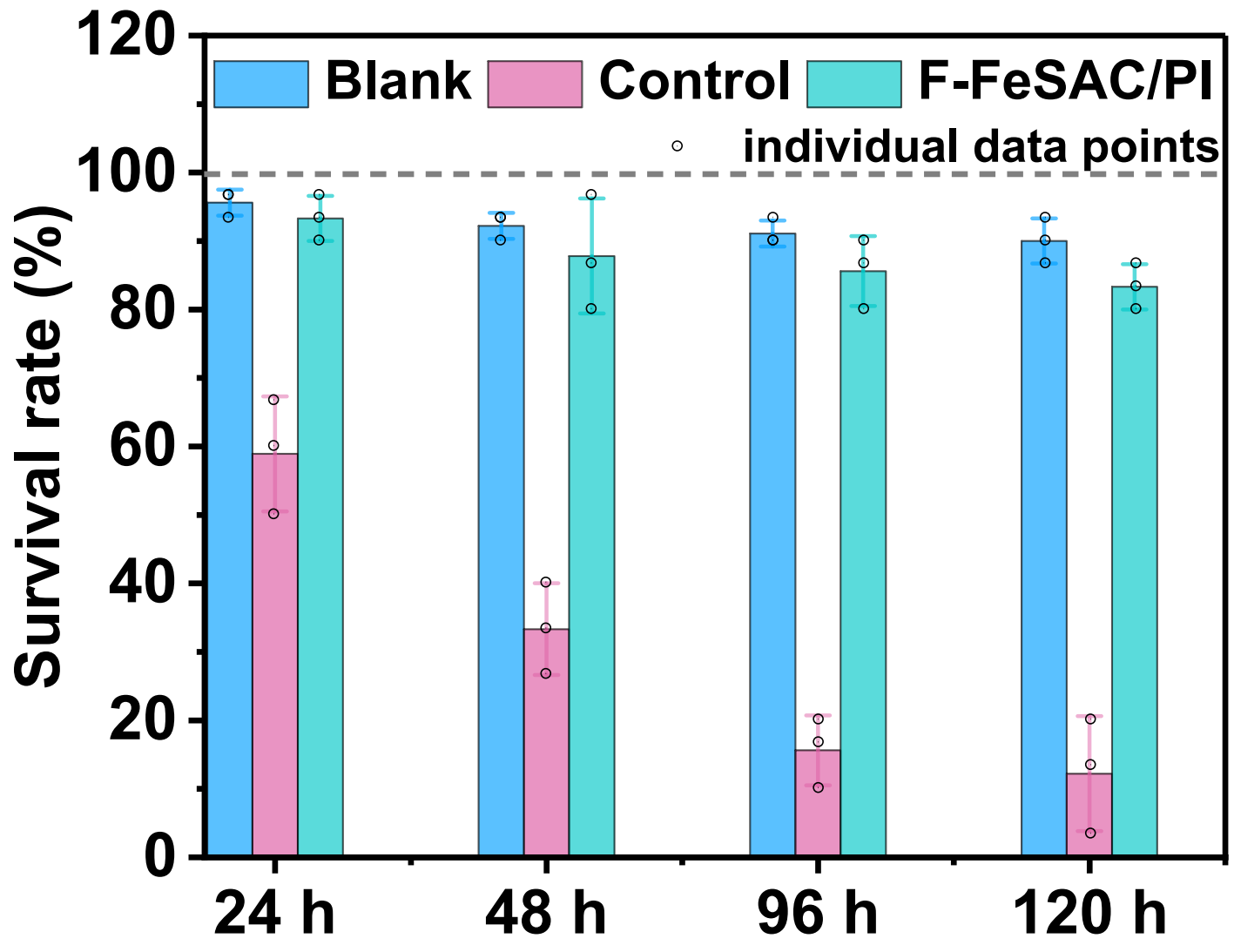

Supplementary Fig. 49 | Zebrafish survival following exposure to treated and untreated 4-CP solutions. Quantitative survival rates of zebrafish exposed to the control, untreated 4-CP solution, and treated solution for 24, 48, 96, and 120 h. Survival rates were calculated as the number of surviving zebrafish divided by the total number of exposed zebrafish in each replicate. Data are presented as mean ± SD from three independent experiments (n = 3), with 30 zebrafish per group in each experiment. Statistical analysis was performed using one-way ANOVA followed by Tukey's multiple comparisons test. Detailed statistical results are provided in Supplementary Table 6. Source data are provided as a Source Data file.

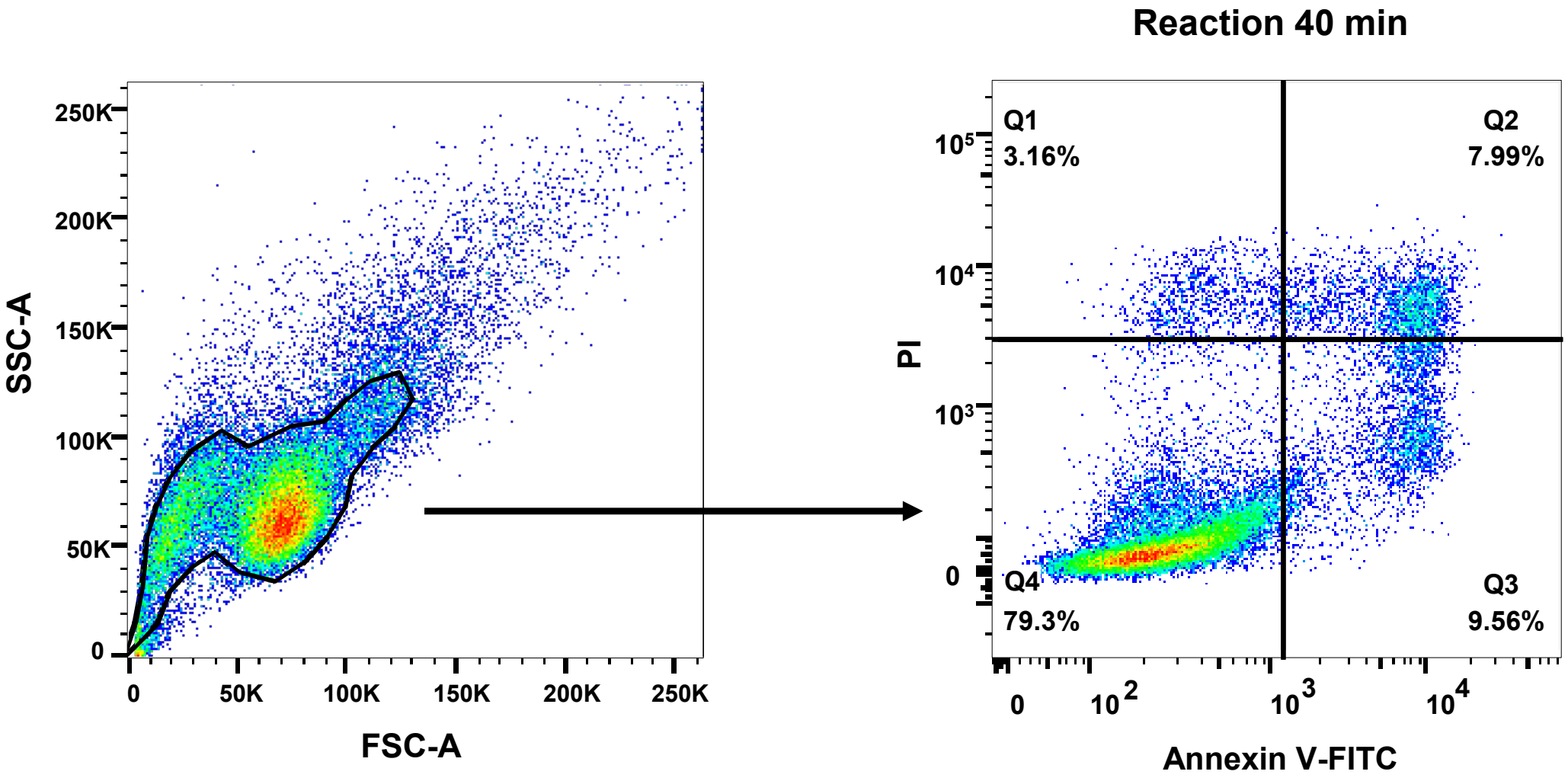

Supplementary Fig. 50 | Gating strategy for flow cytometry. Figure exemplifying the gating strategy for *Escherichia coli* (*E. coli*) for flow cytometry analysis. The four sections in flow cytometry images represent as below: Q1 dead cells; Q2 late apoptotic cells; Q3 early apoptotic cells; Q4 live cells.

Supplementary Tables

Supplementary Table 1. BET surface area and pore volume of FeSAC and R-FeSAC.

Catalysts	BET specific surface area (m^2 g^{-1})	Pore volume (cm^3 g^{-1})
FeSAC	29.8588	0.222440
H-FeSAC	43.7142	0.282100
F-FeSAC	36.6166	0.256884
NH_2-FeSAC	46.6077	0.302192

Supplementary Table 2. EXAFS fitting parameters at the Fe *K*-edge for various samples.

**Sample**	**Shell**	***CN***[a]	***R*(Å)**[b]	**σ2 (Å²·10⁻³)**[c]	**Δ*E*₀(eV)**[d]	**R factor (%)**
Fe foil	Fe-Fe_1	8*	2.45±0.02	0.0058	4.40±1.72	0.5
	Fe-Fe_2	6*	2.85±0.03	0.0073	5.19±0.68	
Fe_2O_3	Fe-O_1^*	3*	1.95±0.01	0.0068	3.43±0.53	1.2
	Fe-O_2^*	3*	2.05±0.01	0.0040	5.23±1.51	
FePc	Fe-N	4*	1.93±0.01	0.0096	8.93±1.72	1.0
FeSAC	Fe-N	4.3±0.2	1.91±0.01	0.0032	9.06±1.61	0.4
H-FeSAC	Fe-N	4.0±0.2	1.93±0.01	0.0033	5.71±0.98	0.5
NH_2-FeSAC	Fe-N	4.2±0.1	2.04±0.005	0.0034	2.40±0.26	0.2
F-FeSAC	Fe-N	3.8±0.2	1.95±0.017	0.0057	4.51±0.55	0.2

[a]*CN*, coordination number; [b]*R*, distance between absorber and backscatter atoms; [c]σ^2, Debye-Waller factor to account for both thermal and structural disorders; [d]ΔE_0, inner potential correction; *R* factor indicates the goodness of the fit. S_0^2 was set as 0.84 for Fe data, which was obtained from the experimental EXAFS fit of reference by fixing CN as the known crystallographic value and was fixed to all the samples.

Supplementary Table 3. Comparison of catalytic performance among R-FeSACs and other PI-based systems.

**Catalysts**	**Catalyst dosage (g L^{-1})**	**IO_4^- (mM)**	**Contaminant (μM)**	**k_{obs} (min^{-1})**	**Ref.**
N-rGO-CoSA	0.02	1.0	4-CP (100 μM)	0.241	[2]
MnO_2	0.2	1.0	SMD (58 μM)	0.581	[3]
CNTs	0.1	0.1	phenol (20 μM)	0.183	[4]
α-MnO_2	0.2	2.0	Orange II (57 μM)	0.037	[5]
In_2O_3	0.1	0.1	SMX (5 μM)	0.165	[6]
BC_{800}	0.3	0.5	BPA (20 μM)	0.027	[7]
BM-MSBC	1.0	1.0	SMX (20 μM)	0.040	[8]
Fe@PrPOP	0.1	0.5	BPA (5 μM)	0.55	[9]
$MgMn_2O_4$	0.1	1.0	BPA (10 μM)	0.054	[10]
B-CuO	0.5	1.0	MB (62 μM)	0.085	[11]
NH_2-FeSAC	0.05	0.2	4-CP (80 μM)	0.252	This work
H-FeSAC	0.05	0.2	4-CP (80 μM)	0.382	This work
F-FeSAC	0.05	0.2	4-CP (80 μM)	0.770	This work

Supplementary Table 4. Catalytic performances of SAC-based systems regulated *via* coordination environment engineering.

**Catalysts**	**Δn(pollutants) ($\times 10^{-6}$ mol)**	**ω_{metal} (%)**	**Reaction time (min)**	**TOF (min^{-1})**	**Ref.**
Fe/SCN (0.5 g L^{-1})	4-CP (100)	6.20	30	0.006	12
Fe(II) (0.25 mM)	SMX (24.9)	100	2	0.050	13
Fe@PrPOP (0.1 g L^{-1})	BPA (5)	3.62	12	0.006	9
Fe–N–C (0.10 g L^{-1})	BPA (100)	2.00	20	0.140	14
Fe_1/CN (0.05 g L^{-1})	4-CP (100)	11.2	10	0.100	15
Co-N_5/CNT (0.03 g L^{-1})	SMZ (40)	1.39	30	0.189	16
CoN_3O_1 (0.10 g L^{-1})	CIP (15)	0.51	20	0.087	17
FeN_5-SD_2 (0.03 g L^{-1})	BPA (80)	2.21	20	0.338	18
SA-FeN_5 (0.20 g L^{-1})	APAP (30)	2.63	12	0.027	19
Co-OCN (0.03 g L^{-1})	APAP (13)	7.25	60	0.006	20
Fe−N_3C_1 (0.06 g L^{-1})	SIZ (19.6)	0.75	30	0.081	21
Fe-N_4-C (0.10 g L^{-1})	NPX (43)	1.50	10	0.161	22
H-FeSAC (0.05 g L^{-1})	4-CP (79.2)	2.65	10	0.334	This work
F-FeSAC (0.05 g L^{-1})	4-CP (80)	2.59	10	0.345	This work
NH_2-FeSAC (0.05 g L^{-1})	4-CP (73.7)	2.41	10	0.342	This work

Supplementary Table 5. HRMS-based suspect screening of theoretically possible iodinated organic byproducts in the treated solution.

No	Compound name	Molecular formula	Exact mass [M-H]- (individually verified)	Screening result
**1**	Iodoform	CHI_3	392.7140	ND
**2**	Diiodomethane	CH_2I_2	266.8173	ND
**3**	Iodomethane	CH_3I	140.9207	ND
**4**	Chloroiodomethane	CH_2ClI	174.8817	ND
**5**	Iodoacetic acid	$C_2H_3IO_2$	184.9105	ND
**6**	Diiodoacetic acid	$C_2H_2I_2O_2$	310.8066	ND
**7**	Triiodoacetic acid	$C_2HI_3O_2$	436.7038	ND
**8**	Chloroiodoacetic acid	$C_2H_2ClIO_2$	218.8715	ND
**9**	Bromoiodoacetic acid	$C_2H_2BrIO_2$	262.8210	ND
**10**	3-Iodopropionic acid	$C_3H_5IO_2$	198.9261	ND (MIC response observed; excluded based on isotope-pattern distribution)
**11**	3-Bromo-3-iodoacrylic acid	$C_3H_2BrIO_2$	274.8210	ND
**12**	2-Iodo-3-methylbutenedioic acid	$C_5H_5IO_4$	254.9160	ND
**13**	Iodoacetamide	C_2H_4INO	183.9265	ND
**14**	Diiodoacetamide	$C_2H_3I_2NO$	309.8231	ND
**15**	Iodopropionamide	C_3H_6INO	197.9421	ND
**16**	Iodoacetonitrile	C_2H_2IN	165.9154	ND
**17**	Diiodoacetonitrile	C_2HI_2N	292.8198	ND
**18**	Iodoacetaldehyde	C_2H_3IO	168.9156	ND
**19**	Diiodoacetaldehyde	$C_2H_2I_2O$	294.8122	ND
**20**	Iodonitromethane	CH_2INO_2	185.9057	ND
**21**	Diiodonitromethane	CHI_2NO_2	311.8024	ND
**22**	2-Iodophenol	C_6H_5IO	218.9313	
**23**	3-Iodophenol	C_6H_5IO	218.9313	ND
**24**	4-Iodophenol	C_6H_5IO	218.9313	
**25**	4-Iodo-2-methylphenol	C_7H_7IO	232.9469	ND
**26**	2-Iodo-4-methylphenol	C_7H_7IO	232.9469	

No	Compound name	Molecular formula	Exact mass [M-H]- (individually verified)	Screening result
**27**	4-Iodo-2,6-dimethylphenol	C_8H_9IO	246.9625	ND
**28**	2-Iodo-4-chlorophenol	C_6H_4ClIO	252.8923	
**29**	2-Iodo-6-chlorophenol	C_6H_4ClIO	252.8923	ND
**30**	4-Iodo-2-chlorophenol	C_6H_4ClIO	252.8923	
**31**	2-Iodo-4-bromophenol	C_6H_4BrIO	296.8417	ND
**32**	4-Iodo-2-nitrophenol	$C_6H_4INO_3$	263.9163	ND
**33**	2-Iodo-4-nitrophenol	$C_6H_4INO_3$	263.9163	
**34**	2,4-Diiodophenol	$C_6H_4I_2O$	344.8279	
**35**	2,6-Diiodophenol	$C_6H_4I_2O$	344.8279	ND
**36**	2,5-Diiodophenol	$C_6H_4I_2O$	344.8279	
**37**	2,6-Diiodo-4-nitrophenol	$C_6H_3I_2NO_3$	389.8130	ND
**38**	2,4-Diiodo-6-nitrophenol	$C_6H_3I_2NO_3$	389.8130	
**39**	2,6-Diiodo-4-chlorophenol	$C_6H_3ClI_2O$	378.7889	ND
**40**	2,4-Diiodo-6-chlorophenol	$C_6H_3ClI_2O$	378.7889	
**41**	2,4,6-Triiodophenol	$C_6H_3I_3O$	470.7245	ND
**42**	2,3,5-Triiodophenol	$C_6H_3I_3O$	470.7245	
**43**	4-Chloro-2-iodophenol	C_6H_4ClIO	252.8923	ND
**44**	4-Chloro-2,6-diiodophenol	$C_6H_3ClI_2O$	378.7889	ND
**45**	2-Chloro-4-iodophenol	C_6H_4ClIO	252.8923	ND
**46**	2-Chloro-4,6-diiodophenol	$C_6H_3ClI_2O$	378.7889	ND
**47**	4-Chloro-6-iodobenzene-1,2-diol	$C_6H_4ClIO_2$	268.8872	
**48**	4-Chloro-6-iodobenzene-1,3-diol	$C_6H_4ClIO_2$	268.8872	ND
**49**	2-Iodo-5-chlorohydroquinone	$C_6H_4ClIO_2$	268.8872	
**50**	2,6-Diiodo-5-chlorohydroquinone	$C_6H_3ClI_2O_2$	394.7838	ND
**51**	2-Iodo-5-chloro-p-benzoquinone	$C_6H_2ClIO_2$	266.8715	ND
**52**	2,6-Diiodo-5-chloro-p-benzoquinone	$C_6HClI_2O_2$	392.7682	ND
**53**	2-Iodo-4-nitrophenol	$C_6H_4INO_3$	263.9163	
**54**	4-Iodo-2-nitrophenol	$C_6H_4INO_3$	263.9163	ND
**55**	2-Iodo-5-nitrophenol	$C_6H_4INO_3$	263.9163	
**56**	2,4-Diiodo-6-nitrophenol	$C_6H_3I_2NO_3$	389.8130	ND
**57**	2,6-Diiodo-4-nitrophenol	$C_6H_3I_2NO_3$	389.8130	
**58**	Triiodo nitrophenol	$C_6HI_3NO_3$	514.7018	ND

No	Compound name	Molecular formula	Exact mass [M-H]- (individually verified)	Screening result
**59**	3-Iodo-4-hydroxybenzaldehyde	$C_7H_5IO_2$	246.9262	
**60**	2-Iodo-4-hydroxybenzaldehyde	$C_7H_5IO_2$	246.9262	ND
**61**	4-Iodo-3-hydroxybenzaldehyde	$C_7H_5IO_2$	246.9262	
**62**	3,5-Diiodo-4-hydroxybenzaldehyde	$C_7H_4I_2O_2$	372.8228	ND
**63**	2,6-Diiodo-4-hydroxybenzaldehyde	$C_7H_4I_2O_2$	372.8228	
**64**	3-Iodo-4-hydroxy-5-nitrobenzaldehyde	$C_7H_4INO_4$	291.9112	ND
**65**	3-Iodo-4-hydroxybenzoic acid	$C_7H_5IO_3$	262.9211	ND
**66**	2-Iodo-4-hydroxybenzoic acid	$C_7H_5IO_3$	262.9211	
**67**	3,5-Diiodo-4-hydroxybenzoic acid	$C_7H_4I_2O_3$	388.8177	ND
**68**	2,6-Diiodo-4-hydroxybenzoic acid	$C_7H_4I_2O_3$	388.8177	ND
**69**	3-Iodo-4-hydroxy-5-methylbenzoic acid	$C_8H_7IO_3$	276.9367	ND
**70**	3,5-Diiodo-4-hydroxy-methylbenzoic acid	$C_8H_6I_2O_3$	402.8334	ND
**71**	2-Iodo-5-chloro-4-hydroxybenzoic acid	$C_7H_4ClIO_3$	296.8821	ND
**72**	Monochloro-monoiodophenol (all isomers)	C_6H_4ClIO	252.8923	ND
**73**	Dichloro-monoiodophenol	$C_6H_3Cl_2IO$	286.8533	ND
**74**	Monochloro-diiodophenol	$C_6H_3ClI_2O$	378.7889	ND
**75**	Monochloro-monoiodohydroquinone	$C_6H_4ClIO_2$	268.8872	ND
**76**	Dichloro-monoiodohydroquinone	$C_6H_3Cl_2IO_2$	302.8482	ND (MIC response observed; excluded based on isotope-pattern distribution)
**77**	Monochloro-monoiodo-p-benzoquinone	$C_6H_2ClIO_2$	266.8715	ND (MIC response observed; excluded based on isotope-pattern distribution)
**78**	Dichloro-monoiodo-p-benzoquinone	$C_6HCl_2IO_2$	300.8326	ND
**79**	Monochloro-diiodo-p-benzoquinone	$C_6HClI_2O_2$	392.7682	ND
**80**	Monochloro-monoiodonitrophenol	$C_6H_3ClINO_3$	297.8773	ND
**81**	Dichloro-monoiodonitrophenol	$C_6H_2Cl_2INO_3$	331.8384	ND

No	Compound name	Molecular formula	Exact mass [M-H]- (individually verified)	Screening result
**82**	Monochloro-monoiodohydroxybenzaldehyde	$C_7H_4ClIO_2$	280.8872	ND
**83**	Monochloro-monoiodohydroxybenzoic acid	$C_7H_4ClIO_3$	296.8821	ND
**84**	Dichloro-iodohydroxybenzoic acid	$C_7H_3Cl_2IO_3$	330.8431	ND
**85**	Monochloro-diiodohydroxybenzoic acid	$C_7H_3ClI_2O_3$	422.7787	ND
**86**	2-Iodo-[1,1'-biphenyl]-2-ol	$C_{12}H_9IO$	294.9625	ND (MIC response observed; excluded based on isotope-pattern distribution)
**87**	4-Iodo-[1,1'-biphenyl]-4-ol	$C_{12}H_9IO$	294.9625	
**88**	Diiodohydroxybiphenyl	$C_{12}H_8I_2O$	420.8592	ND (MIC response observed; excluded based on isotope-pattern distribution)
**89**	Chloroiodohydroxybiphenyl	$C_{12}H_8ClIO$	328.9236	ND
**90**	Dichloroiodohydroxybiphenyl	$C_{12}H_7Cl_2IO$	362.8846	ND
**91**	Diiodo biphenol	$C_{12}H_8I_2O_2$	436.8541	ND
**92**	Triiodo biphenol	$C_{12}H_7I_3O_2$	562.7507	ND
**93**	Chloroiodobiphenol	$C_{12}H_8ClIO_2$	344.9185	ND
**94**	Polychloro-polyiododiphenyl ether	$C_{12}H_{(7-x-y)}Cl_xI_yO_2$	various isomers	ND
**95**	2,4,6-Triiodo-4-chlorohydroxybiphenyl	$C_{12}H_6ClI_3O$	580.7169	ND
**96**	Triiodohydroxydiphenyl ether	$C_{12}H_7I_3O_3$	578.7457	ND
**97**	Iodomaleic acid	$C_4H_3IO_4$	240.9003	ND
**98**	Diiodomaleic acid	$C_4H_2I_2O_4$	366.7970	ND
**99**	Iodofumaric acid	$C_4H_3IO_4$	240.9003	ND
**100**	Iodomuconic acid	$C_6H_5IO_4$	266.9160	ND
**101**	Diiodomuconic acid	$C_6H_4I_2O_4$	392.8126	ND
**102**	Chloroiodomuconic acid	$C_6H_4ClIO_4$	300.8770	ND
**103**	Iodosuccinic acid	$C_4H_5IO_4$	242.9160	ND (MIC response observed; excluded based on isotope-pattern distribution)

No	Compound name	Molecular formula	Exact mass [M-H]- (individually verified)	Screening result
**104**	Iodomalic acid	$C_4H_5IO_5$	258.9109	ND
**105**	Iodooxaloacetic acid	$C_4H_3IO_5$	256.8952	ND
**106**	Iodomalonic acid	$C_3H_3IO_4$	228.9003	ND
**107**	Iodinated naphthols	$C_{10}H_7IO$	268.9469	ND
**108**	Diiodonaphthol	$C_{10}H_6I_2O$	394.8435	ND
**109**	Iodoanthraquinone	$C_{14}H_7IO_2$	332.9418	ND
**110**	Iodinated furans	C_4H_3IO	192.9156	ND
**111**	Iodinated pyrroles	C_4H_4IN	191.9316	ND
**112**	(E)-3-(5-Iodofuran-2-yl)acrylic acid	$C_7H_5IO_3$	262.9211	ND
**113**	3,5-Diiodosalicylic acid	$C_7H_4I_2O_3$	388.8177	ND
**114**	3,5-Diiodo-L-tyrosine	$C_9H_9I_2NO_3$	431.8599	ND
**115**	3-Iodo-L-tyrosine	$C_9H_{10}INO_3$	305.9633	ND
**116**	3-Iodophthalic acid	$C_8H_5IO_4$	290.9160	ND
**117**	3-Iodo-4-hydroxyphenylacetic acid	$C_8H_7IO_3$	276.9367	ND
**118**	3,5-Diiodo-4-hydroxyphenylacetic acid	$C_8H_6I_2O_3$	402.8334	ND (MIC response observed; excluded based on isotope-pattern distribution)
**119**	methyl iododihydroxybenzoate	$C_8H_7IO_4$	292.9316	ND
**120**	methyl chloro-iododihydroxybenzoate	$C_8H_6ClIO_4$	326.8927	ND
**121**	3-Iodo-4-chlorocatechol	$C_6H_4ClIO_2$	268.8872	
**122**	5-Iodo-4-chlorocatechol	$C_6H_4ClIO_2$	268.8872	ND
**123**	6-Iodo-4-chlorocatechol	$C_6H_4ClIO_2$	268.8872	
**124**	3,5-Diiodo-4-chlorocatechol	$C_6H_3ClI_2O_2$	394.7838	
**125**	3,6-Diiodo-4-chlorocatechol	$C_6H_3ClI_2O_2$	394.7838	ND
**126**	5,6-Diiodo-4-chlorocatechol	$C_6H_3ClI_2O_2$	394.7838	
**127**	Triiodo-4-chlorocatechol	$C_6H_2ClI_3O_2$	520.6805	ND
**128**	2-Iodo-4-chlororesorcinol	$C_6H_4ClIO_2$	268.8872	
**129**	5-Iodo-4-chlororesorcinol	$C_6H_4ClIO_2$	268.8872	ND
**130**	6-Iodo-4-chlororesorcinol	$C_6H_4ClIO_2$	268.8872	
**131**	2,5-Diiodo-4-chlororesorcinol	$C_6H_3ClI_2O_2$	394.7838	ND

No	Compound name	Molecular formula	Exact mass [M-H]- (individually verified)	Screening result
**132**	2,6-Diiodo-4-chlororesorcinol	$C_6H_3ClI_2O_2$	394.7838	
**133**	5,6-Diiodo-4-chlororesorcinol	$C_6H_3ClI_2O_2$	394.7838	
**134**	Triiodo-4-chlororesorcinol	$C_6H_2ClI_3O_2$	520.6805	ND
**135**	2-Iodo-5-chloro-p-benzoquinone	$C_6H_2ClIO_2$	266.8715	ND
**136**	2,6-Diiodo-5-chloro-p-benzoquinone	$C_6HClI_2O_2$	392.7682	ND
**137**	2,3-Diiodo-5-chloro-p-benzoquinone	$C_6HClI_2O_2$	392.7682	
**138**	Triiodo-5-chloro-p-benzoquinone	$C_6ClI_3O_2$	/	ND
**139**	3-Iodo-2-chlorohydroquinone	$C_6H_4ClIO_2$	268.8872	
**140**	5-Iodo-2-chlorohydroquinone	$C_6H_4ClIO_2$	268.8872	ND
**141**	6-Iodo-2-chlorohydroquinone	$C_6H_4ClIO_2$	268.8872	
**142**	3,5-Diiodo-2-chlorohydroquinone	$C_6H_3ClI_2O_2$	394.7838	
**143**	3,6-Diiodo-2-chlorohydroquinone	$C_6H_3ClI_2O_2$	394.7838	ND
**144**	5,6-Diiodo-2-chlorohydroquinone	$C_6H_3ClI_2O_2$	394.7838	
**145**	Triiodo-2-chlorohydroquinone	$C_6H_2ClI_3O_2$	520.6805	ND
**146**	3-Iodo-2,5-dichlorohydroquinone	$C_6H_3Cl_2IO_2$	302.8482	ND
**147**	3,6-Diiodo-2,5-dichlorohydroquinone	$C_6H_2Cl_2I_2O_2$	428.7448	ND
**148**	2,3-Diiodosuccinic acid	$C_4H_4I_2O_4$	368.8126	ND
**149**	2-Iodo-3-hydroxysuccinic acid	$C_4H_5IO_5$	258.9109	ND
**150**	Iodomaleic acid	$C_4H_3IO_4$	240.9003	ND
**151**	Iodofumaric acid	$C_4H_3IO_4$	240.9003	
**152**	Diiodomaleic acid	$C_4H_2I_2O_4$	366.7970	ND
**153**	Diiodofumaric acid	$C_4H_2I_2O_4$	366.7970	
**154**	Chloroiodosuccinic acid	$C_4H_4ClIO_4$	276.8770	ND
**155**	2,3-Diiodomuconic acid	$C_6H_4I_2O_4$	392.8126	ND
**156**	2,5-Diiodomuconic acid	$C_6H_4I_2O_4$	392.8126	
**157**	2-Iodo-3-hydroxymuconic acid	$C_6H_5IO_5$	282.9109	ND
**158**	2-Iodo-5-hydroxymuconic acid	$C_6H_5IO_5$	282.9109	
**159**	Monoiodomuconic acid (all isomers)	$C_6H_5IO_4$	266.9160	ND (MIC response observed; excluded based on isotope-pattern distribution)

No	Compound name	Molecular formula	Exact mass [M-H]- (individually verified)	Screening result
**160**	Chloroiodomuconic acid	$C_6H_4ClIO_4$	300.8770	ND
**161**	Hydroxy-dichloro-monoiododiphenyl ether	$C_{12}H_7Cl_2IO_2$	378.8795	ND (MIC response observed; excluded based on isotope-pattern distribution)
**162**	Hydroxy-dichloro-diiododiphenyl ether	$C_{12}H_6Cl_2I_2O_2$	504.7761	ND
**163**	Hydroxy-dichloro-triiododiphenyl ether	$C_{12}H_5Cl_2I_3O_2$	630.6728	ND
**164**	Hydroxy-dichloro-tetraiododiphenyl ether	$C_{12}H_4Cl_2I_4O_2$	756.5694	ND
**165**	Chloro-dihydroxy-monoiodobiphenyl	$C_{12}H_8ClIO_2$	344.9185	ND
**166**	Chloro-dihydroxy-diiodobiphenyl	$C_{12}H_7ClI_2O_2$	470.8151	ND
**167**	Chloro-dihydroxy-triiodobiphenyl	$C_{12}H_6ClI_3O_2$	596.7118	ND
**168**	Chloro-dihydroxy-tetraiodobiphenyl	$C_{12}H_5ClI_4O_2$	722.6084	ND
**169**	Chloro-hydroxy-monoiodobiphenylquinone	$C_{12}H_6ClIO_3$	358.8977	ND
**170**	Chloro-hydroxy-diiodobiphenylquinone	$C_{12}H_5ClI_2O_3$	484.7944	ND
**171**	Chloro-hydroxy-triiodobiphenylquinone	$C_{12}H_4ClI_3O_3$	610.6910	ND
**172**	Chloro-hydroxy-tetraiodobiphenylquinone	$C_{12}H_3ClI_4O_3$	736.5877	ND
**173**	Chloro-hydroxy-monoiodobiphenylhydroquinone	$C_{12}H_8ClIO_3$	360.9134	ND
**174**	Dichloro-dihydroxy-monoiodobiphenyl	$C_{12}H_7Cl_2IO_2$	378.8795	ND (MIC response observed; excluded based on isotope-pattern distribution)
**175**	Dichloro-dihydroxy-diiodobiphenyl	$C_{12}H_6Cl_2I_2O_2$	504.7761	ND
**176**	Dichloro-dihydroxy-triiodobiphenyl	$C_{12}H_5Cl_2I_3O_2$	630.6728	ND
**177**	Dichloro-dihydroxy-tetraiodobiphenyl	$C_{12}H_4Cl_2I_4O_2$	756.5694	ND
**178**	Dichloro-tetrahydroxy-monoiodoterphenyl	$C_{18}H_{11}Cl_2IO_4$	486.9006	ND
**179**	Dichloro-tetrahydroxy-diiodoterphenyl	$C_{18}H_{10}Cl_2I_2O_4$	612.7973	ND
**180**	Dichloro-tetrahydroxy-triiodoterphenyl	$C_{18}H_9Cl_2I_3O_4$	738.6939	ND
**181**	Dichloro-tetrahydroxy-tetraiodoterphenyl	$C_{18}H_8Cl_2I_4O_4$	864.5906	ND

No	Compound name	Molecular formula	Exact mass [M-H]- (individually verified)	Screening result
**182**	Dichloro-tetrahydroxy-pentaiodoterphenyl	$C_{18}H_7Cl_2I_5O_4$	990.4872	ND
**183**	Trichloro-trihydroxy-monoiodoterphenyl	$C_{18}H_{10}Cl_3IO_3$	504.8667	ND
**184**	Trichloro-trihydroxy-diiodoterphenyl	$C_{18}H_9Cl_3I_2O_3$	630.7634	ND
**185**	Trichloro-trihydroxy-triiodoterphenyl	$C_{18}H_8Cl_3I_3O_3$	756.6600	ND
**186**	Trichloro-trihydroxy-tetraiodoterphenyl	$C_{18}H_7Cl_3I_4O_3$	882.5567	ND
**187**	Dichloro-trihydroxy-monoiodobiphenyl	$C_{12}H_7Cl_2IO_3$	394.8744	ND
**188**	Dichloro-trihydroxy-diiodobiphenyl	$C_{12}H_6Cl_2I_2O_3$	520.7711	ND
**189**	Dichloro-trihydroxy-triiodobiphenyl	$C_{12}H_5Cl_2I_3O_3$	646.6677	ND
**190**	Dichloro-trihydroxy-tetraiodobiphenyl	$C_{12}H_4Cl_2I_4O_3$	772.5643	ND
**191**	Dichloro-trihydroxy-monoiodoterphenyl	$C_{18}H_{11}Cl_2IO_3$	470.9057	ND
**192**	Dichloro-trihydroxy-diiodoterphenyl	$C_{18}H_{10}Cl_2I_2O_3$	596.8024	ND
**193**	Dichloro-trihydroxy-triiodoterphenyl	$C_{18}H_9Cl_2I_3O_3$	722.6990	ND
**194**	Dichloro-trihydroxy-tetraiodoterphenyl	$C_{18}H_8Cl_2I_4O_3$	848.5957	ND
**195**	Dichloro-trihydroxy-pentaiodoterphenyl	$C_{18}H_7Cl_2I_5O_3$	974.4923	ND
**196**	Dichloro-monoiododiphenyl ether	$C_{12}H_7Cl_2IO$	362.8846	ND
**197**	Dichloro-diiododiphenyl ether	$C_{12}H_6Cl_2I_2O$	488.7812	ND
**198**	Dichloro-triiododiphenyl ether	$C_{12}H_5Cl_2I_3O$	614.6779	ND
**199**	Dichloro-tetraiododiphenyl ether	$C_{12}H_4Cl_2I_4O$	740.5745	ND
**200**	Hydroxylated dichloro-monoiododiphenyl ether	$C_{12}H_7Cl_2IO_2$	378.8795	ND
**201**	Hydroxylated dichloro-diiododiphenyl ether	$C_{12}H_6Cl_2I_2O_2$	504.7761	ND

301

Supplementary Table 6. Statistical comparison of zebrafish survival rates between the post-reaction 4-CP solution, blank, and untreated 4-CP solution.

Time	Comparison	Mean difference (%)	95% CI (%)	Adjusted P value
24 h	post-reaction 4-CP solution (F-FeSAC/PI) vs Blank	-2.22	-15.57 to 11.13	0.869
	post-reaction 4-CP solution (F-FeSAC/PI) vs 4-CP solution (Control)	34.44	21.09 to 47.79	5.28×10^{-4}
48 h	post-reaction 4-CP solution (F-FeSAC/PI) vs Blank	-4.44	-20.19 to 11.30	0.679
	post-reaction 4-CP solution (F-FeSAC/PI) vs 4-CP solution (Control)	54.44	38.70 to 70.19	1.02×10^{-4}
96 h	post-reaction 4-CP solution (F-FeSAC/PI) vs Blank	-5.56	-16.34 to 5.23	0.323
	post-reaction 4-CP solution (F-FeSAC/PI) vs 4-CP solution (Control)	70.00	59.22 to 80.78	2.58×10^{-6}
120 h	post-reaction 4-CP solution (F-FeSAC/PI) vs Blank	-6.67	-20.58 to 7.25	0.368
	post-reaction 4-CP solution (F-FeSAC/PI) vs 4-CP solution (Control)	71.11	57.19 to 85.03	1.06×10^{-5}

Note: Survival rates were calculated separately for each replicate as the number of surviving zebrafish divided by the corresponding number of exposed zebrafish. Data are presented as mean ± SD from three independent experiments (n = 3), with 30 zebrafish per group in each experiment. Statistical analysis was performed using one-way ANOVA followed by Tukey's multiple comparisons test. The one-way ANOVA results were F(2,6) = 44.65, 81.66, 287.27 and 180.64 at 24, 48, 96 and 120 h, respectively, with $P = 2.50 \times 10^{-4}$, 4.45×10^{-5}, 1.10×10^{-6} *and* 4.36×10^{-6}*, respectively. Effect sizes (*η^2*) were 0.937, 0.965, 0.990 and 0.984, respectively. Tukey's comparisons were two-sided. Tukey-adjusted P values and simultaneous 95% confidence intervals are reported for pairwise comparisons. CI, confidence interval.*

Supplementary References

1. Momma, K. & Izumi, F. VESTA 3 for three-dimensional visualization of crystal, volumetric and morphology data. *J. Appl. Crystallogr.* **44**, 1272-1276 (2011).
2. Long Y, Dai J, Zhao S, Su Y, Wang Z, Zhang Z. Atomically dispersed cobalt sites on graphene as efficient periodate activators for selective organic pollutant degradation. *Environ. Sci. Technol* **55**, 5357-5370 (2021).
3. Du J, Xiao G, Xi Y, Zhu X, Su F, Kim SH. Periodate activation with manganese oxides for sulfanilamide degradation. *Water Res.* **169**, 115278 (2020).
4. Peng J*, et al.* Removal of phenols by highly active periodate on carbon nanotubes: A mechanistic investigation. *Environ. Sci. Technol* **57**, 10804-10815 (2023).
5. Yao Y, Zhang L, Qiu Y, Li Z, Ma Z, Wang S. Phase–activity relationship of MnO2 nanomaterials in periodate oxidation for organic pollutant degradation. *Water Res.* **264**, 122224 (2024).
6. Yang T*, et al.* Enhanced hydroxyl radical generation for micropollutant degradation in the In_2O_3/Vis-LED process through the addition of periodate. *Water Res.* **243**, 120401 (2023).
7. Dai J, Wang Z, Chen K, Ding D, Yang S, Cai T. Applying a novel advanced oxidation process of biochar activated periodate for the efficient degradation of bisphenol A: Two nonradical pathways. *Chem. Eng. J* **453**, 139889 (2023).
8. He L*, et al.* Ball milling-assisted preparation of sludge biochar as a novel periodate activator for nonradical degradation of sulfamethoxazole: Insight into the mechanism of enhanced electron transfer. *Environ. Pollut.* **316**, 120620 (2023).
9. Shi Z*, et al.* Fe confined in Porphyrin-based porous organic polymer as an efficient periodate activator for nonradical pathway removal of contaminants. *Sep. Purif. Technol.* **317**, 123868 (2023).
10. Yu J*, et al.* Periodate activation with stable $MgMn_2O_4$ spinel for bisphenol A removal: Radical and non-radical pathways. *Chem. Eng. J* **459**, 141574 (2023).

11. Zhang X, *et al.* Activation of periodate with pinewood biochar-CuO composite for the removal of recalcitrant organic pollutants-Mechanisms and degradation products. *Chem. Eng. J* **465**, 142916 (2023).
12. Tang Q, *et al.* Electron transfer mediated activation of periodate by contaminants to generate $^1O_2$ by charge-confined single-atom catalyst. *Nat. Commun.* **15**, 9549 (2024).
13. Zong Y, *et al.* Enhanced oxidation of organic contaminants by iron(ii)-activated periodate: The significance of high-valent iron–oxo species. *Environ. Sci. Technol.* **55**, 7634-7642 (2021).
14. Cheng C, Ren W, Miao F, Chen X, Chen X, Zhang H. Generation of Fe^{IV}=O and its contribution to Fenton-like reactions on a single-atom iron−N−C catalyst. *Angew. Chem.* **135**, e202218510 (2023).
15. Zhang L S, *et al.* Carbon nitride supported high-loading Fe single-atom catalyst for activation of peroxymonosulfate to generate $^1O_2$ with 100 % selectivity. *Angew. Chem. Int. Ed.* **60**, 21751-21755 (2021).
16. Xie M, *et al.* Single-atom Co-N_5 catalytic sites on carbon nanotubes as peroxymonosulfate activator for sulfamerazine degradation via enhanced electron transfer pathway. *Sep. Purif. Technol.* **304**, 122398 (2023).
17. Wang Z, *et al.* Cobalt single atoms anchored on oxygen-doped tubular carbon nitride for efficient peroxymonosulfate activation: Simultaneous coordination structure and morphology modulation. *Angew. Chem.* **134**, e202202338 (2022).
18. Jin S, *et al.* Remote tuning of single-atom Fe-N_5 sites via high-coordination defects for enhanced Fenton-like water decontamination. *Nat. Commun.* **16**, 10455 (2025).
19. Liu C, Li J, He X, Yue J, Chen M, Chen JP. The "4 + 1" strategy fabrication of iron single-atom catalysts with selective high-valent iron-oxo species generation. *Proc. Natl. Acad. Sci.* **121**, e2322283121 (2024).
20. Wu Q-Y, Yang Z-W, Wang Z-W, Wang W-L. Oxygen doping of cobalt-single-atom coordination enhances peroxymonosulfate activation and high-valent cobalt-oxo

species formation. *Proc. Natl. Acad. Sci.* **120**, e2219923120 (2023).

21. Wu Z*, et al.* Facilely tuning the first-shell coordination microenvironment in iron single-atom for Fenton-like chemistry toward highly efficient wastewater purification. *Environ. Sci. Technol.* **57**, 14046-14057 (2023).

22. Yin K*, et al.* High-loading of well dispersed single-atom catalysts derived from Fe-rich marine algae for boosting Fenton-like reaction: Role identification of iron center and catalytic mechanisms. *Appl. Catal. B* **336**, 122951 (2023).
