# Supplementary material for: Enzyme-mimicking peripheral substituents beyond the second coordination sphere tune single-atom catalysts for periodate activation
Source: Nat Commun. 2026 Sep 17;17:10926. doi: 10.1038/s41467-026-77805-5 (PMC13680503; doi:10.1038/s41467-026-77805-5)
Supplement: Supplementary file 2 [file 41467_2026_77805_MOESM2_ESM.pdf]

nature portfolio

Peer Review File

Enzyme-Mimicking Peripheral Substituents beyond the Second Coordination Sphere Tune Single-Atom Catalysts for Periodate Activation

Corresponding Author: Professor Tao Zeng

Parts of this Peer Review File have been redacted as indicated to remove third-party material.

This file contains all reviewer reports in order by version, followed by all author rebuttals in order by version.

Version 1:

Reviewer comments:

Reviewer #1

(Remarks to the Author)
In this manuscript, the authors report a strategy to regulate the catalytic behavior of Fe single-atom catalysts (Fe-SACs) through peripheral substituent engineering. By introducing tunable functional groups (-F, -H, -NH2) into a conjugated organic framework, the electronic structure of Fe-N-C active sites is modulated while preserving the first coordination shell. The optimized F-FeSAC activates periodate (PI) via a non-radical electron-transfer pathway (ETP), achieving selective pollutant removal without generating iodinated byproducts. However, the manuscript currently lacks sufficiently rigorous mechanistic evidence, and fails to clearly distinguish itself from recent reports on PI activation by single-atom catalysts. Some experimental designs require clarification.
1.The charge-confined single-atom catalysts for PI activation via a non-radical electron-transfer pathway has already been reported (Nat. Commun. 2024. 15, 9549). The authors should clarify the novelty of this work.
2.The Abstract should be reorganized. A considerable portion of the abstract is devoted to describing catalyst design, which leaves limited space for AOPs (application of catalyst), other key findings and mechanistic insights.
3.While the Introduction section provides some relevant background, the connection to the study's objectives needs to be strengthened, such as the advantages of periodate activation compared with other advanced oxidation processes, the scientific rationale, and innovation of their catalyst design strategy specifically in the field of periodate activation
4.The manuscript emphasizes regulation “beyond the second coordination sphere”, but the geometric definition of the second sphere is not provided. Moreover, the DFT models do not clearly demonstrate spatial separation between Fe-N4 core and substituents.
5.ETP mechanism is primarily supported by exclusion-based evidence. Changes in open-circuit potential (OCP) reflect surface charge redistribution. It can’t demonstrate electron transfer from pollutant to PI. Solid evidences are needed for the direct electron-transfer pathway.
6.The classical d-band theory does not directly reveal d-band upshift and reduced antibonding occupation without detailed electronic structure analysis. The authors should consider COHP (Crystal Orbital Hamilton Population) analysis and Orbital-resolved DOS discussion.
7.The reference to “PMS electron acceptor” in line 579 contradicts with the use of PI in Fig. 5m.
8.The performance of the proposed system is evaluated by pollutant removal efficiency. The authors should provide TOC measurements to assess mineralization efficiency and conduct intermediate product analysis to elucidate degradation pathways. Without such data, it remains unclear whether it generates persistent or toxic intermediates.
9.Given that periodate activation may generate multiple inorganic and organic iodine species, non-target screening of iodinated organic byproducts is essential, which is necessary to exclude secondary environmental risks. Moreover, a quantitative iodine mass balance should be provided to assess iodine recovery efficiency and long-term sustainability of the system.

Reviewer #2

(Remarks to the Author)
This work establishes peripheral functionalization beyond the second coordination sphere as an enzyme-inspired design

principle, enabling efficient pollutant removal via electron-transfer processes. The characterization techniques employed, including HRTEM, XAFS, electrochemical analysis, and DFT calculations, are adequate and consistent with typical studies in the field of single-atom catalysis, yet the overall approach appears somewhat homogenized. Electron-transfer processes (ETP) represent a currently active research topic, with various catalysts reported to exhibit promising performance. These include catalysts based on finely modulated steric positioning and tailoring of the d-band center in high-valent metal–oxo species. Against this context, what distinct advantages does the R-FeSAC catalyst offer relative to these systems? Several related studies focusing on the modulation of coordination environments have already been reported, and the novelty of the present work appears to lie mainly in the introduction of PI. I cannot recommend this work for publication in NC. Furthermore, the current dataset still allows for alternative mechanistic interpretations, suggesting that several key conclusions require substantially more robust and quantitative validation.

1. The authors have not fully decoupled the observed kinetics from morphology- and transport-related effects. This is because (i) PI activation may be governed by PI adsorption/partitioning and interfacial charge transfer, and (ii) differences in porosity and wettability can modulate local oxidant concentrations and effective collision frequencies.
2. Turnover frequency is suggested to calculate for comparison.
3. Line 248: Notably, CNTs exhibited no PI activation capability. Line 82: Lai et al. recently identified a ETP pathway in the carbon nanotube (CNT)/PI system, wherein CNTs function as an electron shuttle to mediate direct electron transfer from organic contaminants to PI. What is the reason for this discrepancy?
4. The DFT parameters to calculate HOMO values of pollutants should be provided.
5. Clarify terminology: describing the process as “Fenton-like” may be misleading if the dominant mechanism is non-radical ETP.
6. I note that the degradation performance remains efficient even in a pilot-scale ETP reactor. Given this, what factors currently hinder its practical application?
7. When HOI concentrations fall below the detection limit of HPLC, the use of HPLC for the detection of 4-iodophenol (4-IP) and 2-iodophenol (2-IP) is not suitable. Instead, LC-MS is recommended.
8. Fig. S20. The authors are requested to calculate the utilization efficiency of $IO4^-$ (i.e., the moles of 4-CP degradable per mole of $IO4^-$), and explain whether there are differences in the utilization efficiency of $IO4^-$ among FeSAC modified with different substituents, and whether fluorine substituents can improve the effective activation ratio of $IO4^-$? It appears that H-FeSAC exhibits the highest PI utilization efficiency. Please quantify the corresponding difference and provide a detailed discussion.
9. Only the signal of 1O2 was detected in the EPR experiments, and no signals of free radicals such as ·OH and $O2\cdot^-$ were detected. The authors are requested to supplement EPR data (e.g., DMPO trapping $\cdot OH/O2\cdot^-$) to further confirm that the system is dominated by non-radicals
10. Fig S26. Dichlorination products of 4-CP were observed. The ETP system promotes electron loss from 4-CP, corresponding to the oxidation of 4-CP. In this context, what is the mechanism responsible for the reductive dichlorination? In addition, -OH addition products were detected, implying that ·OH contributes to 4-CP degradation. However, this observation appears inconsistent with the quenching experimental results.

Reviewer #3

(Remarks to the Author)
This manuscript presents a strategic mechanism for the simultaneous utilization of 4-CP and periodate (PI) in wastewater treatment using Fe-N-C SAC catalysts. The study provides a comprehensive evaluation, including zebrafish-based toxicity assessments, compatibility tests with various pollutants, and DFT-level orbital analysis. However, the mechanistic discussion relies on indirect negative evidence, and more robust empirical support is required to substantiate the proposed conclusions.

1. While studies on the second and third coordination shells exist in various catalytic fields, the authors also emphasized these long-range interactions. Please clarify why long-range interactions were prioritized over first-shell modifications, which typically exert stronger electronic control. Was first-shell modulation feasible with the chosen synthetic route? Please provide more explicit reasons for why these reagents were chosen to target long-range effects.
2. The stability discussion focuses on the F-FeSAC catalyst, but the structural integrity of the F-C bond is not directly verified post-reaction. Please provide XPS F 1s spectra or 19F solid-state NMR of the spent catalyst to confirm that F substituents remain intact after catalytic cycles.
3. While similar 2% Fe loading is stated for effective Fe incorporation into the scaffold, what is the theoretical maximum for metal substitution in this scaffold? A quantitative analysis or estimation of the fraction of available sites occupied by Fe would help assess the site utilization efficiency.
4. The logic for comparing this system to CNT-based systems needs improvement. Given that earlier studies (e.g., CNT/PI) have already identified the electron transfer pathway, please clarify the more specific novelty of this work that justifies its publication.
5. Is the observed ETP mechanism unique to Fe? If the interaction with PI is significant, would other transition metals (e.g., Co or Ni) exhibit similar performance within this scaffold? A brief comparison or discussion on metal specificity would strengthen the manuscript.
6. The manuscript attributes 4-CP removal to a polymerization-mediated ETP. The LC-MS identification of chlorinated oligomeric species on the used catalyst is consistent with this. However, to substantiate the claim that polymerization is the dominant pathway, a clearer mechanistic description of how ETP leads to oligomer formation would be necessary.
7. The claim of a non-radical mechanism relies on negative evidence. A direct comparison between IO3 radicals and IO3- ions would be more convincing than noting the absence of a radical peak. Additionally, please provide positive reference

data to prove the detection is sensitive enough.
8. Regarding the PEM analysis, did the authors verify that no species crossover occurred after the measurement?
9. What was the scientific basis for selecting 3 min and 10 min intervals for 4-CP addition in the premixing experiments?
10. For the zebrafish toxicity assay (Fig. 6d), please report quantitative survival rates and include statistical analysis. The current presentation with representative images alone is insufficient for drawing conclusions about biocompatibility.
11. The correlation between ln(kobs) and φ1/2 (Fig. 3k) excludes BA and NB based on a φ1/2 < 1.2 V threshold (line 387), yet the two pollutants are included in the linear regressions in Fig. 4b,c. This inconsistency raises questions whether this categorization has a mechanistic basis or is introduced as a post-hoc threshold to optimize linearity.
12. In the discussion of the pilot-scale ETP reactor (lines 571-583), the authors refer to "the PMS electron acceptor", whereas the rest of the section and Fig. 5m describe a PI-based system. The authors should clarify whether PMS was indeed used in the pilot-scale experiment.
13. Minor comments:
- Typo, 0.8 in Figure 3g should be corrected to 0.08.
- The bar graph in Figure 3c is currently difficult to interpret and compare.
- In Fig. 3k, the R2 value (0.962) differs from the R2 = 0.90 stated in the main text (line 388). The values should be corrected.
- Including specific values such as g-values for EPR and directly stating kobs values would significantly improve the clarity and impact for the readership.

Version 2:

Reviewer comments:

Reviewer #1

(Remarks to the Author)
The authors have revised the manuscript according to my comments, and the paper can be accepted for publication

Reviewer #2

(Remarks to the Author)
After revision, the authors have addressed all my concerns, I agree to accept this manuscript under current version.

Reviewer #3

(Remarks to the Author)
Although the authors addressed some of my previous concerns, I have further questions that should be addressed.

1. The differential treatment of BA and NB across the two analyses is acknowledged. However, the 1.2 V threshold underlying Fig. 3k is not independently substantiated and appears to have been defined a posteriori by the data points it segregates. Whereas Fig. 4a-c incorporate BA and NB into the φ1/2 and frontier-orbital correlations, Fig. 3k excludes these same points. The adoption of inconsistent inclusion criteria across a single contaminant set raises the concern that the classification was introduced to maximize the coefficient of determination rather than on a defensible mechanistic basis. The authors are requested to substantiate the 1.2 V threshold on physicochemical grounds. 2. In Fig. 4b, CBZ appears to be excluded from the dataset despite its inclusion in Fig. 4a and 4c. Clarification as to whether this exclusion is intentional is requested. 3. The difference in the Fe d-band center among the F-, H-, and NH2-FeSAC variants is on the order of 0.02 eV. While a difference of this magnitude is consistent with the modification of solely peripheral substituents upon an otherwise invariant Fe-N-C framework, it resides within the characteristic uncertainty of DFT and is therefore of limited reliability as a quantitative descriptor. Consequently, the deterministic phrasing invoked in the abstract and discussion ("optimizes" or "upshifts" the Fe d-band center) is arguably overstated. The authors are requested either to establish the robustness of this ordering or to attenuate the causal language accordingly. 4. In Fig. 4e, the reported adsorption energies and Fe-O bond lengths deviate substantially from those presented in the original manuscript (F-FeSAC/PI: Fe-O 1.64 to 2.16 Å; Eads -2.02 to -3.38 eV, with similar shifts for H- and NH2-FeSAC). Shifts of this magnitude imply a completely different optimized adsorption geometry, yet the molecular structures in Fig. 4e remain unchanged between versions. Please explain the origin of these modified values and provide the actual optimized structures that correspond to the reported Eads and bond distances.

Version 3:

Reviewer comments:

Reviewer #3

(Remarks to the Author)
The authors address well my comments, so I would like to recommend this manuscript to publish in Nat. Commun.

Open Access This Peer Review File is licensed under a Creative Commons Attribution 4.0 International License, which permits use, sharing, adaptation, distribution and reproduction in any medium or format, as long as you give appropriate credit to the original author(s) and the source, provide a link to the Creative Commons license, and indicate if changes were made.
In cases where reviewers are anonymous, credit should be given to 'Anonymous Referee' and the source.
The images or other third party material in this Peer Review File are included in the article's Creative Commons license, unless indicated otherwise in a credit line to the material. If material is not included in the article's Creative Commons license and your intended use is not permitted by statutory regulation or exceeds the permitted use, you will need to obtain permission directly from the copyright holder.
To view a copy of this license, visit https://creativecommons.org/licenses/by/4.0/

REVIEWER COMMENTS

Reviewer #1 (Remarks to the Author):

In this manuscript, the authors report a strategy to regulate the catalytic behavior of Fe single-atom catalysts (Fe-SACs) through peripheral substituent engineering. By introducing tunable functional groups (-F, -H, $-NH_2$) into a conjugated organic framework, the electronic structure of Fe–N–C active sites is modulated while preserving the first coordination shell. The optimized F-FeSAC activates periodate (PI) via a non-radical electron-transfer pathway (ETP), achieving selective pollutant removal without generating iodinated byproducts. However, the manuscript currently lacks sufficiently rigorous mechanistic evidence, and fails to clearly distinguish itself from recent reports on PI activation by single-atom catalysts. Some experimental designs require clarification.

1.The charge-confined single-atom catalysts for PI activation via a non-radical electron-transfer pathway has already been reported (Nat. Commun. 2024. 15, 9549). The authors should clarify the novelty of this work.

Response 1:

We thank the reviewer for pointing out this important study. The reported charge-confined Fe single-atom catalyst for PI activation (*Nature Communications 2024, 15, 9549*) indeed represents an important precedent in the development of non-radical PI activation systems. In that work, an Fe/SCN catalyst with an $Fe–N_3S_1$ coordination configuration was designed to confine charge transfer at the Fe single-atom site. The confined electron-transfer process enabled contaminant-derived electrons to activate PI and generate singlet oxygen ($^1O_2$), thereby coupling electron-transfer-mediated dechlorination with $^1O_2$-assisted ring opening and mineralization.

We agree that this prior work should be clearly distinguished from our study. We have therefore revised the Introduction section to better clarify the novelty of our work. Although both studies involve PI activation over single-atom catalysts, they address different scientific questions, employ different active-site regulation strategies, and lead to different pollutant-removal mechanisms.

First, **the central scientific question of our work is different.** The previous Fe/SCN study focused on engineering the first coordination shell of the Fe single-atom center through N/S coordination to confine electron transfer and promote $^1O_2$ generation. In contrast, our work asks whether peripheral substituents located beyond the second

coordination sphere can remotely regulate the electronic structure of an atomically dispersed Fe center and thereby establish a new structure-property relationship for PI activation. This question has rarely been addressed in artificial SAC systems, mainly because conventional pyrolysis-derived M–N–C catalysts make it difficult to precisely modify remote functional groups without simultaneously perturbing the primary metal coordination structure.

Second, **the active-site regulation strategy is fundamentally different.** The reported Fe/SCN catalyst relies on first-coordination-shell engineering, namely the construction of an $Fe–N_3S_1$ site. By contrast, our R-FeSAC platform maintains a similar primary $Fe–N_4$ coordination environment while introducing different peripheral substituents (R = F, H, NH_2) beyond the second coordination sphere. This structural design allows us to decouple remote substituent effects from the first coordination shell and to examine long-range electronic regulation of the Fe center. As demonstrated by XPS, XANES, EXAFS, electrochemical measurements, and DFT calculations, the electron-withdrawing F substituent remotely decreases the electron density of Fe, shifts the Fe *d*-band center upward, strengthens PI adsorption, and accelerates interfacial electron transfer.

Third, **the PI activation and pollutant-removal mechanisms are different.** In the reported Fe/SCN system, charge-confined electron transfer promotes the generation of $^1O_2$, and the degradation process proceeds through an ETP-coupled $^1O_2$ oxidation route that contributes to dechlorination, ring opening, and mineralization. In our system, multiple lines of evidence, including scavenging experiments, EPR, DPA oxidation, D_2O substitution, PMSO probing, premixing tests, in situ Raman spectroscopy, OCP/i-t/EIS measurements, and galvanic oxidation experiments, exclude dominant contributions from radicals, $^1O_2$, or high-valent Fe–oxo species. Instead, the data support a direct ETP pathway mediated by the catalyst/PI interfacial complex. Importantly, this ETP process induces pollutant coupling and polymerization, leading to the formation of surface-deposited oligomers rather than following a $^1O_2$-dominated mineralization route. Therefore, our study does not simply report another non-radical PI activation system; it identifies a peripheral-substituent-governed ETP polymerization mechanism in SAC-catalyzed PI activation.

Finally, **the conceptual novelty of our work lies in introducing an enzyme-mimetic distal regulation principle into SAC design.** Natural metalloenzymes achieve high activity and selectivity not only through the metal center and its immediate

ligands, but also through distal binding pockets that remotely modulate the electronic and steric environment of the active site. However, such distal-pocket-like regulation remains challenging in conventional pyrolysis-based SAC synthesis (including the mentioned *Nature Communications 2024, 15, 9549*). Our R-FeSAC model system demonstrates that peripheral substituents beyond the second coordination sphere can transmit long-range electronic effects to an Fe–N_4 center, thereby regulating charge redistribution, Fe electronic structure, PI adsorption/activation, and electron-transfer kinetics. Thus, the novelty of this work lies not merely in using PI activation as a model reaction, but in establishing a distal-substituent-regulated, enzyme-inspired SAC design strategy that expands the design space beyond conventional first-shell M–N_x coordination engineering.

Action 1: To make this distinction clearer, we have revised the manuscript as follows:

Revised text in the Introduction:

"More importantly, prevailing modulation strategies primarily involve tailoring coordination numbers, heteroatom doping, or creating carbon defects. In contrast, the potential of peripheral organic functional group substitution, a strategy that mirrors the long-range electronic effects in natural metalloenzymes, to regulate the catalytic properties of SACs has rarely been investigated, let alone for PI activation and the elucidation of structure-activity relationships.

Inspired by distal-pocket regulation in natural metalloenzymes, we leverage conjugated organic frameworks as programmable scaffolds to construct precision SACs. This strategy provides a precise route to manipulate the long-range coordination environment through peripheral organic group substitution. By incorporating electron-withdrawing (e.g., -F) or electron-donating (e.g., -NH_2) groups into the conjugated scaffolds, we synthesized a series of well-defined R-FeSACs (R = F, H, NH_2), achieving targeted electronic modulation of the Fe single-atom centers."

2.The Abstract should be reorganized. A considerable portion of the abstract is devoted to describing catalyst design, which leaves limited space for AOPs (application of catalyst), other key findings and mechanistic insights.

Response 2:

We thank the reviewer for this helpful suggestion. We have revised the abstract by condensing the design-related description and giving greater emphasis to the PI-based AOP application, key findings, and mechanistic insights. The revised version now

provides a more balanced summary of the catalyst design, oxidation performance, mechanistic understanding, and practical significance of this work. The revised version is provided below.

Action 2:

Revised Abstract:

Single-atom catalysts (SACs) have attracted extensive interest as enzyme-mimetic active centers; however, faithfully reproducing the long-range regulation exerted by distal binding pockets in natural metalloenzymes remains a formidable challenge. *To investigate how such underexplored long-range effects influence SA center for advanced oxidation reactions, we precisely synthesized a series of conjugated organic SACs with tunable peripheral substituents beyond the second coordination sphere (denoted as R-FeSAC, where R = F, H, NH_2). These catalysts reveal that functional groups in second-beyond coordination shells can remotely regulate periodate (PI) activation and advanced oxidation performance. Among them, F-FeSAC exhibits superior pollutant degradation performance over its H- and NH_2-substituted counterparts, owing to long-range electronic modulation that optimizes the Fe d-band configuration. Mechanistic studies combining in situ Raman spectroscopy, galvanic oxidation assays, and electrochemical analysis reveal that R-FeSAC activates PI to* generate high-potential metastable intermediates, thereby facilitating a direct electron-transfer pathway (ETP) for selective pollutant removal *via* polymerization, rather than radical or singlet-oxygen pathways. Fluorine substitution further tunes the Fe *d*-band center through electron withdrawal and accelerates direct electron-transfer kinetics. The feasibility of the ETP pathway is further supported by a strong correlation between pollutant half-wave potentials($\varphi_{1/2}$) and the LUMO $_{(catalyst/PI)}$–HOMO $_{(pollutant)}$ energy gap, reflecting reaction selectivity dictated by pollutant electronics. Notably, the F-functionalized membrane exhibits outstanding durability under continuous-flow operation, and a pilot-scale ETP reactor achieves efficient degradation of real wastewater without iodate leakage. This work establishes peripheral functionalization beyond the second coordination sphere as an enzyme-inspired design principle *for PI-based advanced oxidation and provides a sustainable, polymerization-mediated paradigm for advanced water purification.*

3.While the Introduction section provides some relevant background, the connection to the study's objectives needs to be strengthened, such as the advantages of periodate activation compared with other advanced oxidation processes, the scientific rationale,

and innovation of their catalyst design strategy specifically in the field of periodate activation.

Response 3:

We thank the reviewer for this valuable comment regarding the logical connection between the background discussion and the objectives of this study. In response, we have refined the Introduction to more clearly establish why periodate activation represents a distinct and promising AOP platform, how this rationale motivates the present study, and how our catalyst design strategy advances the field of PI activation.

Action 3: The revised version is provided below.

Introduction:

Advanced oxidation processes (AOPs) utilizing solid-phase oxidants such as permanganate, persulfate, and periodate (PI, IO_4^-) have garnered significant attention to address pressing environmental challenges[1, 2, 3]. In contrast to permanganate (e.g., $KMnO_4$) and persulfate (e.g., peroxydisulfate, PDS, or peroxymonosulfate, PMS), PI-based AOPs circumvent chromaticity issues and significant pH fluctuations. Their demonstrated cost-effectiveness under specific operational conditions, coupled with advantages like reduced sludge production in certain cases, as evidenced by multiple studies[4, 5, 6], has consequently spurred considerable interest in these systems for water decontamination applications. *Similar to other oxidants, PI itself is relatively stable and exhibits limited oxidative capacity against organic pollutants (E^0= +1.60 V vs. NHE)[7], Structurally, PI is a high-valent iodine(VII) oxyanion with a highly symmetric oxygen coordination environment, which is fundamentally different from peroxide-based oxidants such as PMS and PDS. Accordingly, PI activation does not rely on O–O bond cleavage, but is generally associated with I–O bond activation and subsequent electron-transfer, oxygen-transfer, or reactive-species generation processes. To enhance its oxidative reactivity,* diverse activation strategies have been developed, including physical methods such as ultrasonication[8] and light irradiation[9], chemical approaches such as alkali activation, reducing agents,[10] and transition metal ions[11], as well as freezing-induced activation[12]. *Despite rapid progress in this field, PI-mediated organic pollutant degradation still suffers from sluggish kinetics, highlighting the need to design highly active and resource-efficient activators.*

Recent reports highlight the efficacy of metal-based heterogeneous activators (e.g., FeS, manganese oxides (MnO_2, Mn_3O_4, and Mn_2O_3), and iron-based bimetallic nanoparticles)[13] in activating PI to produce reactive oxygen species (ROS), such as

hydroxyl radicals (•OH), iodate radicals ($IO_3^•$), and superoxide radical($O_2^{•−}$)[1, 14, 15]. *In these systems, PI activation is commonly initiated by interfacial electron transfer or I–O bond cleavage at the catalyst surface, which subsequently gives rise to different oxidizing intermediates and reaction pathways.* However, these radical-based pathways are often hindered by scavenging effects from coexisting inorganic ions and natural organic matter. In contrast, nonradical pathways, including electron-transfer processes (ETP), singlet oxygenation, and high-valent metal-induced oxidation, have emerged as promising alternatives[16, 17, 18, 19]. ETP, in particular, mediates electron transfer from contaminants to oxidants, enabling selective degradation of organics while maintaining resilience against short lifetimes and diffusion limitations typical of radical species[20]. ETP-based oxidation offers distinct advantages, including high selectivity for electron-rich substrates, stability across complex water matrices, avoidance of toxic halogenated byproducts, and efficient oxidant utilization[21]. Furthermore, strong adsorption of reactants on the catalyst surface enhances PI activation and oxidation efficiency, even at low concentrations. This mechanism effectively mitigates radical quenching, a key limitation of conventional Fenton-like systems where reactive species such as $IO_3^•$ and •OH are characterized by short lifetimes. Lai et al.[7] recently identified an ETP pathway in the functionalized carbon nanotube (CNT)/PI system, in which CNTs were modified through oxidative treatment to introduce oxygen-containing functional groups, defect sites, and edge structures. Carbonyl/ketonic groups and double-vacancy defects were proposed to function as electron-shuttling motifs that mediate direct electron transfer from organic contaminants to PI. This mechanistic insight into ETP is pivotal, as it underscores the critical role of catalyst design in optimizing PI adsorption and interfacial redox potentials. *However, in CNT-based systems, ETP can be influenced simultaneously by conductivity, graphitic domains, oxygen-containing groups, vacancy defects, edge sites, and other surface features. As a result, although CNT/PI studies establish carbon-surface-mediated ETP, precise regulation of ETP at a structurally defined catalytic site remains insufficiently explored.* Therefore, the rational design of catalysts to optimize PI adsorption and redox potential is essential for developing efficient and tunable ETP-based water treatment technologies.

Single-atom catalysts (SACs), a groundbreaking class of catalysts, have gained prominence in AOPs due to their unparalleled atomic efficiency and superior catalytic performance, offering significant potential to overcome the sluggish reaction kinetics of PI-based AOPs. Among these, metal-nitrogen (M-N_x) coordination structures exhibit exceptional catalytic activity for PI activation, attributed to their distinctive electronic

properties and charge states[22]. The coordination environment of M-N_x sites is a critical determinant in optimizing the performance of M-N-C catalysts by modulating the local electronic structure[23]. Nevertheless, several key challenges persist. First, conventional pyrolysis synthesis lacks the precision required to control the coordination environment of SACs. The inherent structural uncertainties of these uncontrolled high-temperature processes often lead to metal aggregation and ill-defined active sites, which obstruct a precise mechanistic understanding of SAC catalysis. Second, while the first coordination shell (FCS) is recognized as pivotal, research efforts have predominantly focused on its optimization. The influence of the secondary and second-beyond coordination shells, which are not directly bonded to the metal center but critically modulate the electronic structure and catalytic efficiency, remains a largely unexplored frontier. *More importantly, prevailing modulation strategies primarily involve tailoring coordination numbers, heteroatom doping, or creating carbon defects. In contrast, the potential of peripheral organic functional group substitution, a strategy that mirrors the long-range electronic effects in natural metalloenzymes, to regulate the catalytic properties of SACs has rarely been investigated, let alone for PI activation and the elucidation of structure-activity relationships.*

Inspired by distal-pocket regulation in natural metalloenzymes, we leverage conjugated organic frameworks as programmable scaffolds to construct precision SACs. This strategy provides a precise route to manipulate the long-range coordination environment through peripheral organic group substitution. By incorporating electron-withdrawing (e.g., -F) or electron-donating (e.g., -NH_2) groups into the conjugated scaffolds, we synthesized a series of well-defined R-FeSACs (R = F, H, NH_2), achieving targeted electronic modulation of the Fe single-atom centers. Experimental results demonstrate that F-FeSAC exhibits significantly enhanced activity for PI activation compared to its H- and NH_2- analogues. Mechanistic studies reveal that this enhancement originates from long-range electronic effects, whereby the electron-withdrawing groups optimize the Fe *d*-band configuration, thereby facilitating an efficient ETP between pollutants and the catalyst/PI complex. Theoretical calculations confirm that ETP feasibility is governed by energy alignment between the pollutant HOMO and the catalyst/PI LUMO. A strong correlation between half-wave potentials ($\varphi_{1/2}$) of pollutant and the LUMO $_{(catalyst/PI)}$–HOMO $_{(pollutant)}$ energy gap elucidates reaction selectivity dictated by the electronic properties of pollutants. A pilot-scale ETP reactor was constructed, demonstrating potential for treating wastewater without iodate leakage. This work provides atomic-level insight into how peripheral organic

substituents regulate long-range electronic interactions in SACs, establishing an enzyme-inspired paradigm for the electronic optimization of high-efficiency, non-radical oxidation catalysts specifically for PI activation, and advancing sustainable strategies for water purification.

4.The manuscript emphasizes regulation “beyond the second coordination sphere”, but the geometric definition of the second sphere is not provided. Moreover, the DFT models do not clearly demonstrate spatial separation between Fe-N4 core and substituents.

Response 4:

We sincerely thank the reviewer for raising this important point. To clarify the concept of “beyond the second coordination sphere”, we have carefully reviewed the literature regarding the classification of coordination shells and provide the following definitions.

As defined in previous studies, the first coordination shell refers to atoms that are directly bonded to the single metal atom[24, 25]. The second coordination shell consists of atoms or groups that are not directly bonded to the metal center but are directly connected to, or immediately surround, the first-shell ligands and can still influence the active site through local electronic or interfacial interactions[26]. Structural elements beyond this immediate local environment are therefore regarded as higher coordination shells, or as being beyond the second coordination sphere[27, 28]. In this context, the second coordination sphere is geometrically defined as the local environment immediately surrounding the first-shell ligands, whereas substituent groups located outside this local ligand environment are considered to be beyond the second coordination sphere. (**Fig. R1a-d**)

[Figure Redacted]

Fig. R1 Coordination environment of single-atom dispersed metal sites. Literature reports:(a) *Nano Converg.* ***9**, 34 (2022)*[29], (b) *J. Am. Chem. Soc.* ***147**, 1884-1892 (2025)*[30], (c) *J. Mater. Chem. A* ***10**, 17948-17967 (2022)*[31] and (d) *Nat. Commun.* ***16**, 10455 (2025)*[32]. (e) Our study.

In our study, "beyond the second coordination sphere" specifically refers to the substituent groups that are not directly bonded to the Fe center and are also not part of the immediate second-shell environment surrounding the $Fe–N_4$ moiety. These substituents are spatially separated from the $Fe–N_4$ core, but can still affect its electronic configuration through long-range electronic interactions. This interpretation is consistent with previous studies showing that second- and higher-shell environments can regulate catalytic active sites without direct coordination to the metal center[29, 30]. In particular, Chen et al.[31] reported that both the second and higher coordination shells can influence active sites through long-range electronic delocalization, especially in systems involving exotic atoms or carbon-supported substrates. This type of modulation is similar to that observed in our study, where the substituent groups alter the properties of the $Fe–N_4$ active site without direct coordination to the metal center.

To address the reviewer's concern more directly, we have now explicitly distinguished the $Fe–N_4$ core, the second-shell region, and the substituent groups in the revised structural models. The DFT results show that the substituents are located away from the $Fe–N_4$ core and do not directly coordinate with Fe, thereby demonstrating clear spatial separation between the $Fe–N_4$ core and the substituents (>7.5 Å). These results

confirm that the observed modulation does not originate from direct coordination, but from structural effects beyond the second coordination sphere (**Fig. R2**).

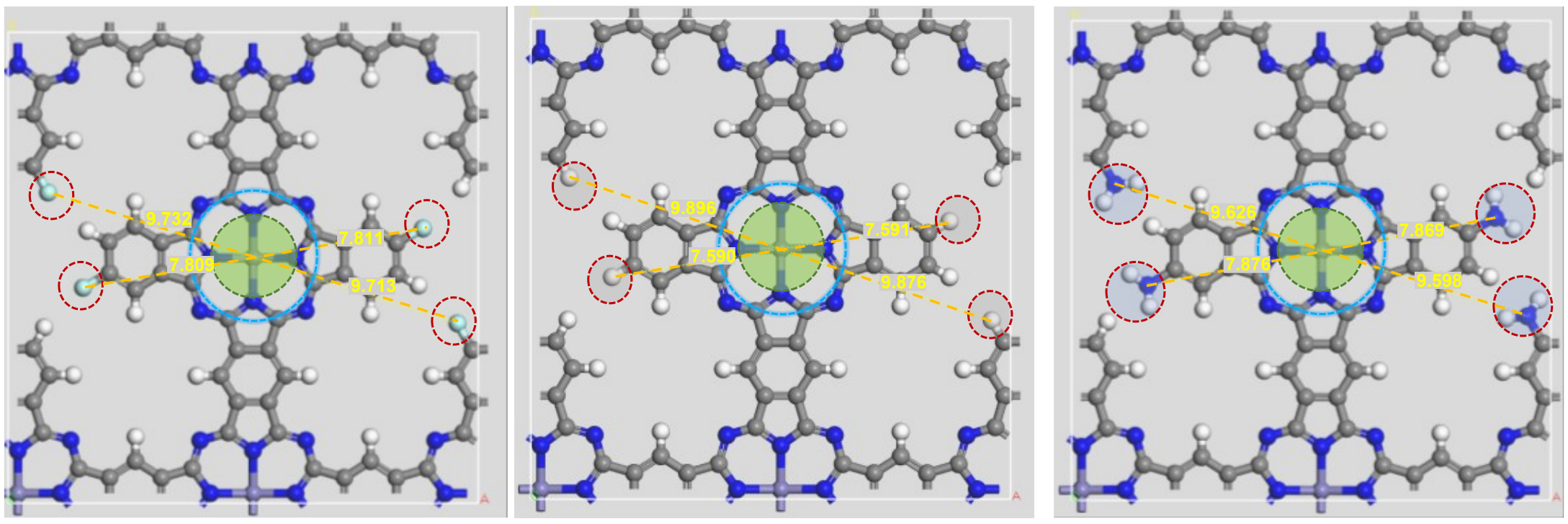

Fig. R2 Structural models illustrating the spatial separation between the Fe–N_4 core and substituent groups.

Action 4: We have added the relevant definitions in the Introduction section of the revised manuscript and further clarified them in the DFT section and the Supporting Information (**Supplementary Fig. 30**).

Introduction: *"The influence of the second and higher coordination shells, which are not directly bonded to the metal center but critically modulate the electronic structure and catalytic efficiency, remains a largely unexplored frontier."*

DFT section: *"To construct Fe centers with beyond-second-coordination-shell substituents, we introduced peripheral substituents around the Fe–N_4 moiety. Although these substituents are not directly coordinated to the Fe center, they remain electronically coupled to the Fe–N_4 core through long-range electronic interactions. The optimized density functional theory (DFT) structures show that the substituent groups are positioned beyond the second coordination sphere, with a distance of >7.5 Å from the Fe–N_4 core (**Supplementary Fig. 30**)."*

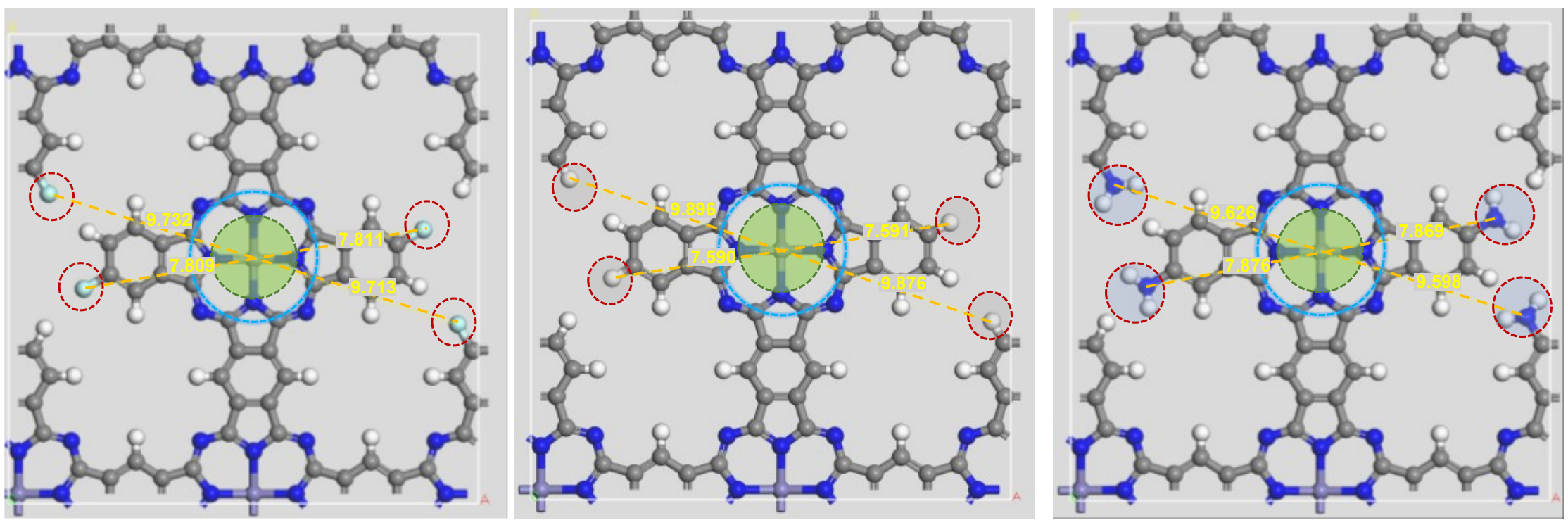

Supplementary Fig. 30 Structural models illustrating the spatial separation between the Fe–N_4 core and substituent groups.

5.ETP mechanism is primarily supported by exclusion-based evidence. Changes in open-circuit potential (OCP) reflect surface charge redistribution. It can't demonstrate electron transfer from pollutant to PI. Solid evidences are needed for the direct electron-transfer pathway.

Response 5:

We sincerely thank the reviewer for this insightful and important comment. We agree that exclusion-based evidence alone is insufficient to rigorously establish a direct electron-transfer pathway, and we also agree that the open-circuit potential (OCP) response mainly reflects interfacial charge redistribution rather than, by itself, proving directional electron transfer from the pollutant to PI. In the revised manuscript, we have therefore carefully moderated the interpretation of the OCP data and reorganized the mechanistic discussion so that the ETP assignment is supported by multiple converging lines of evidence, rather than by OCP or exclusion experiments alone.

First, our mechanistic analysis excludes the major alternative oxidation pathways. Radical scavenging experiments, EPR measurements, NBT assays, β-carotene quenching, D_2O substitution, DPA probing, DMSO quenching, PMSO oxidation, and iodine-speciation analyses collectively show that •OH, $O_2^{\bullet-}$, $^1O_2$, high-valent metal-oxo species, and reactive iodine intermediates do not make significant contributions in the R-FeSAC/PI system (**Fig. 3c; Supplementary Figs. 12-21**). These results narrow the plausible oxidation mechanism to a non-radical pathway and establish the basis for evaluating the electron-transfer process more specifically.

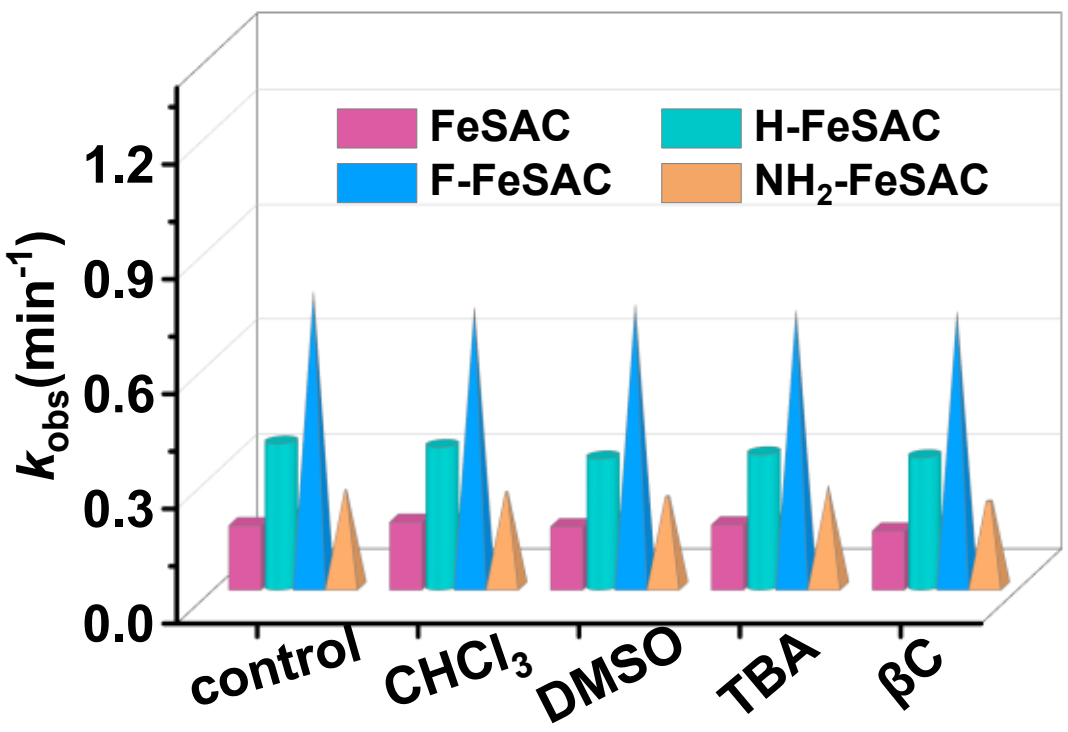

Fig. 3 (c) The k_{obs} values of FeSAC/PI and R-FeSAC/PI systems in the presence of various quenchers.

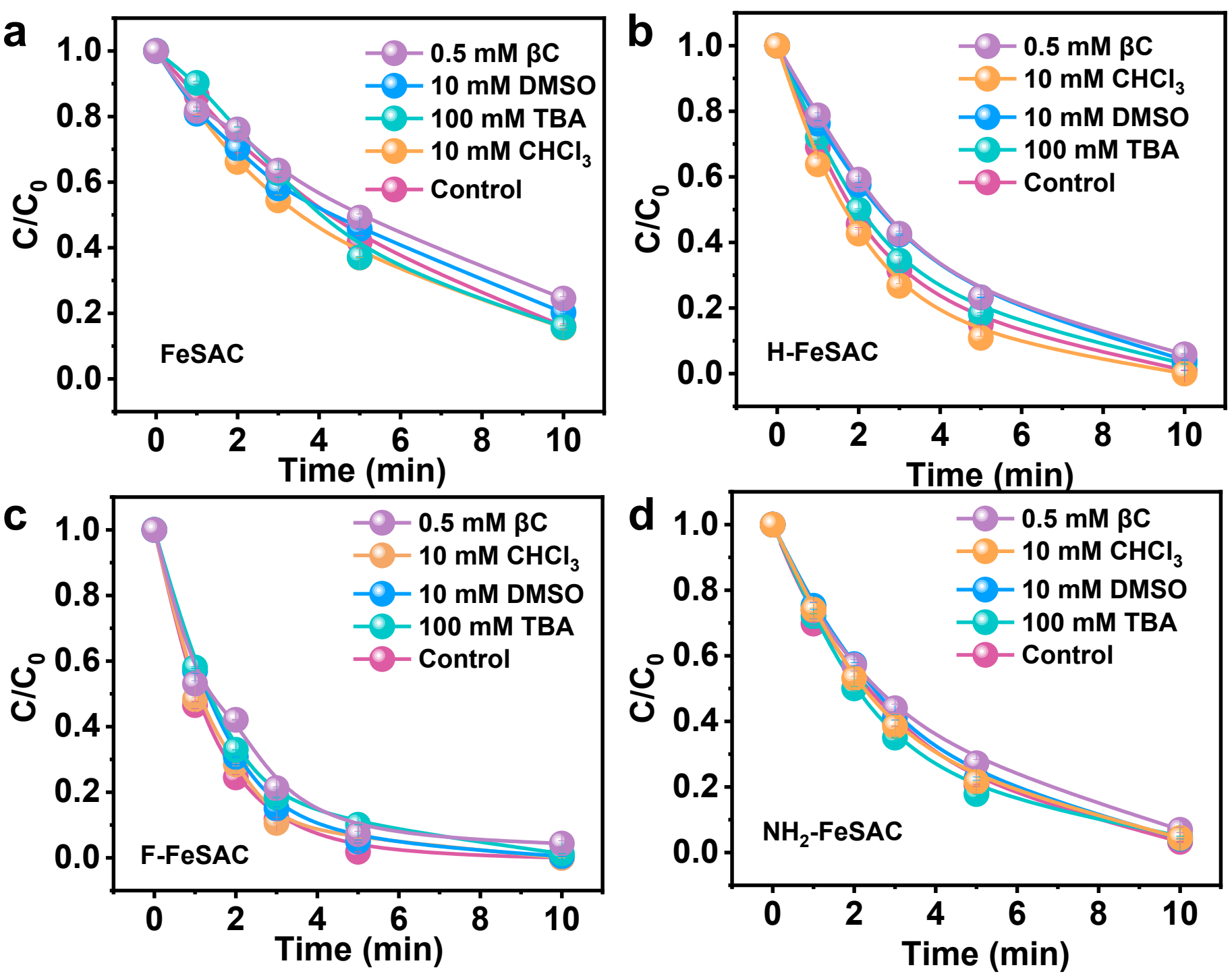

Supplementary Fig. 12 Degradation of 4-CP in (a) FeSAC/PI, (b) H-FeSAC/PI, (c) F-FeSAC/PI and (d) NH_2-FeSAC/PI system under different quenchers. Routine conditions: [4-CP] = 80 μM, [catalyst] = 50 mg/L, [PI] = 0.20 mM, temperature = 25 °C, without pH adjustment.

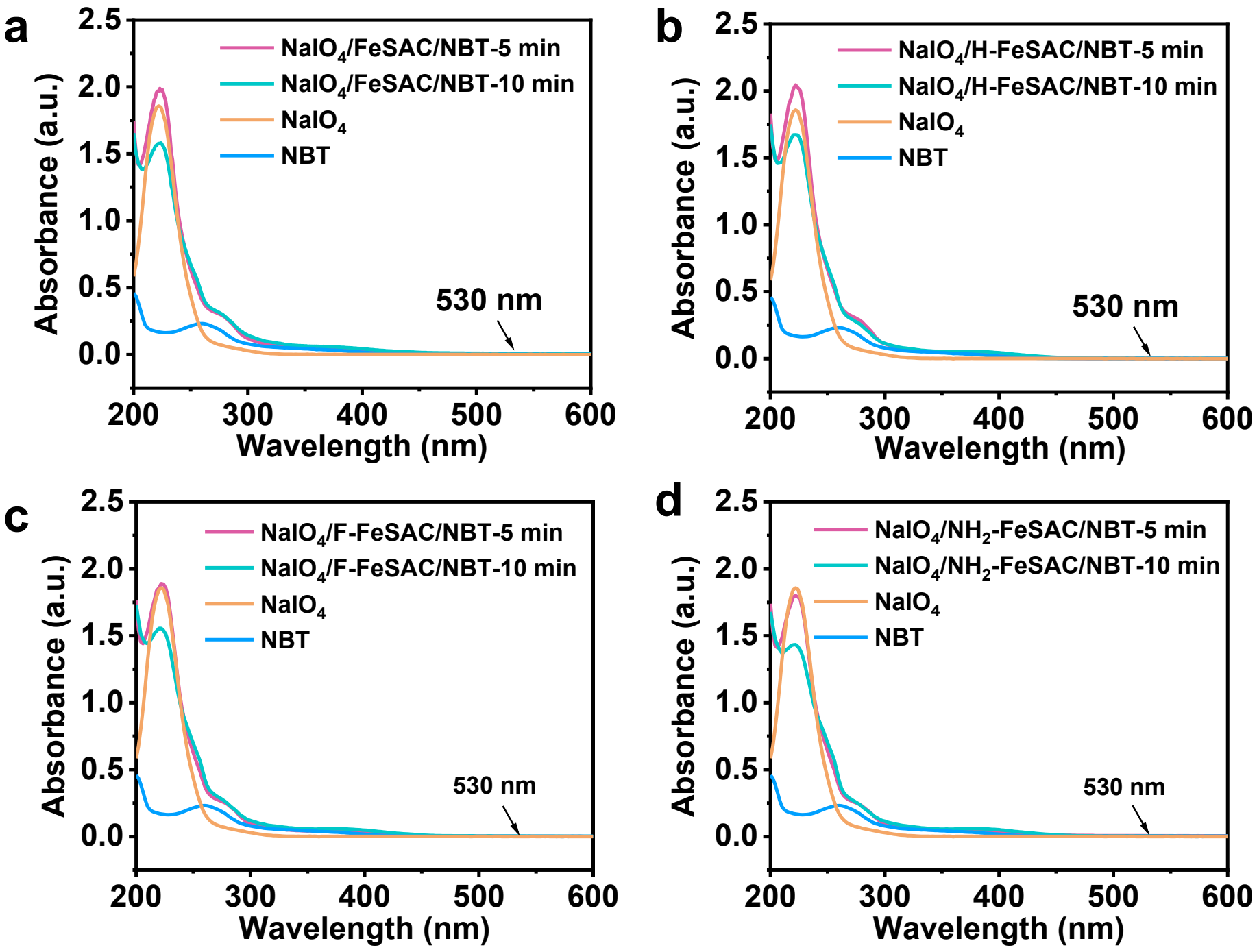

Supplementary Fig. 13 UV-vis absorption spectra of NBT in the (a) FeSAC/PI, (b) H-FeSAC/PI, (c) F-FeSAC/PI and (d) NH_2-FeSAC/PI system.

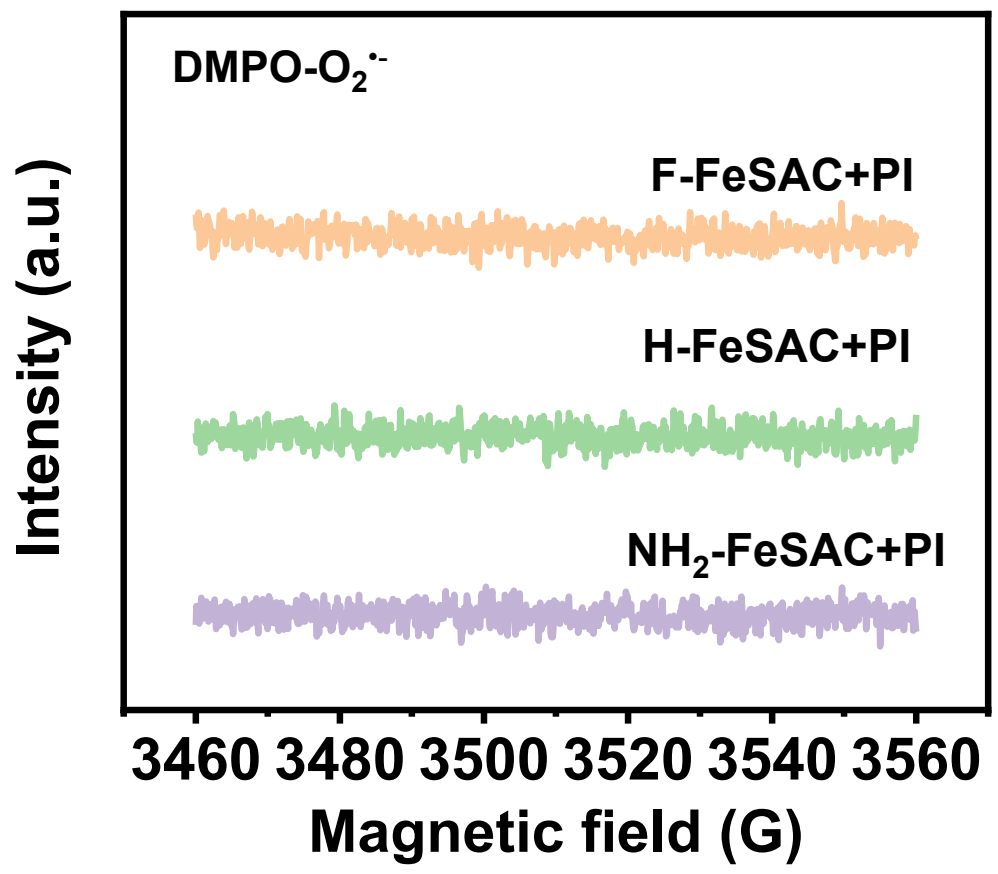

Supplementary Fig. 14 EPR spectra of $O_2^{\bullet-}$ trapped by DMPO in F-FeSAC/PI, H-FeSAC/PI, and NH_2-FeSAC/PI system.

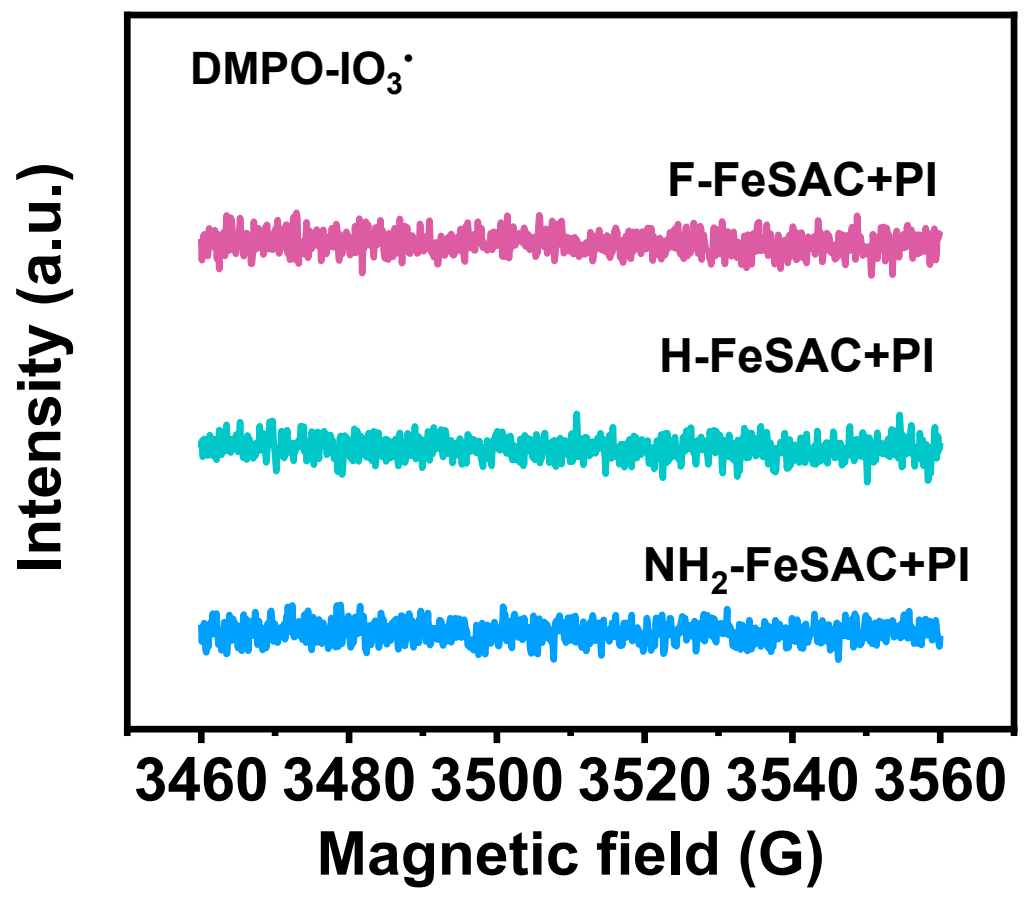

Supplementary Fig. 15 EPR spectra of $IO_3^{\bullet}$ trapped by DMPO in F-FeSAC/PI, H-FeSAC/PI, and NH_2-FeSAC/PI system.

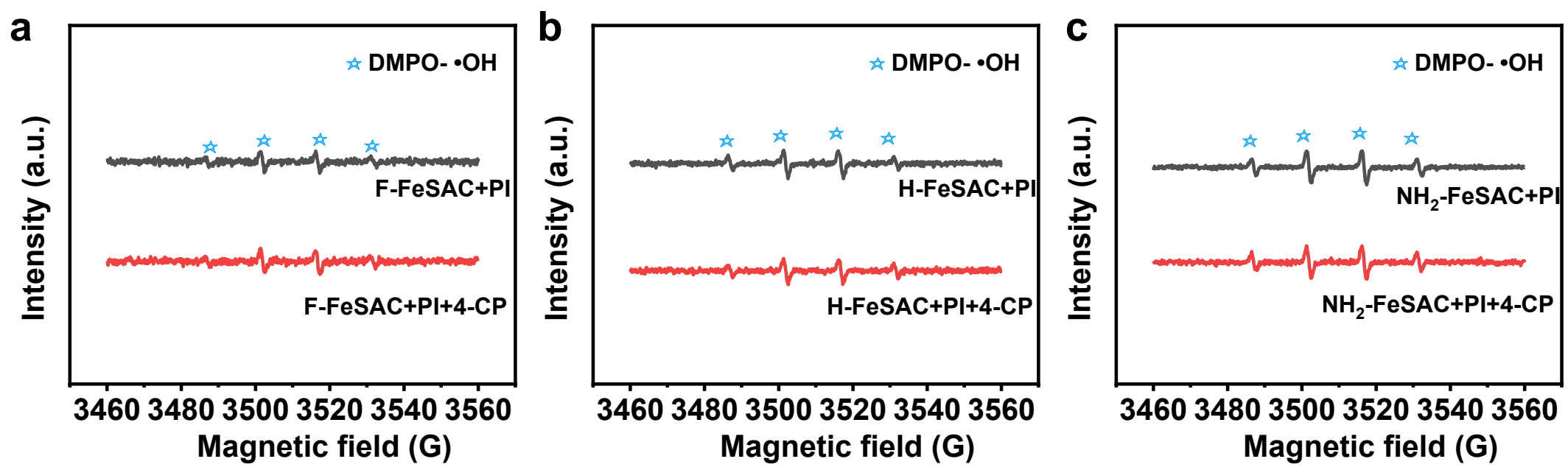

Supplementary Fig. 16 EPR spectra of •OH trapped by DMPO in (a) F-FeSAC/PI, (b) H-FeSAC/PI, and (c) NH_2-FeSAC/PI system.

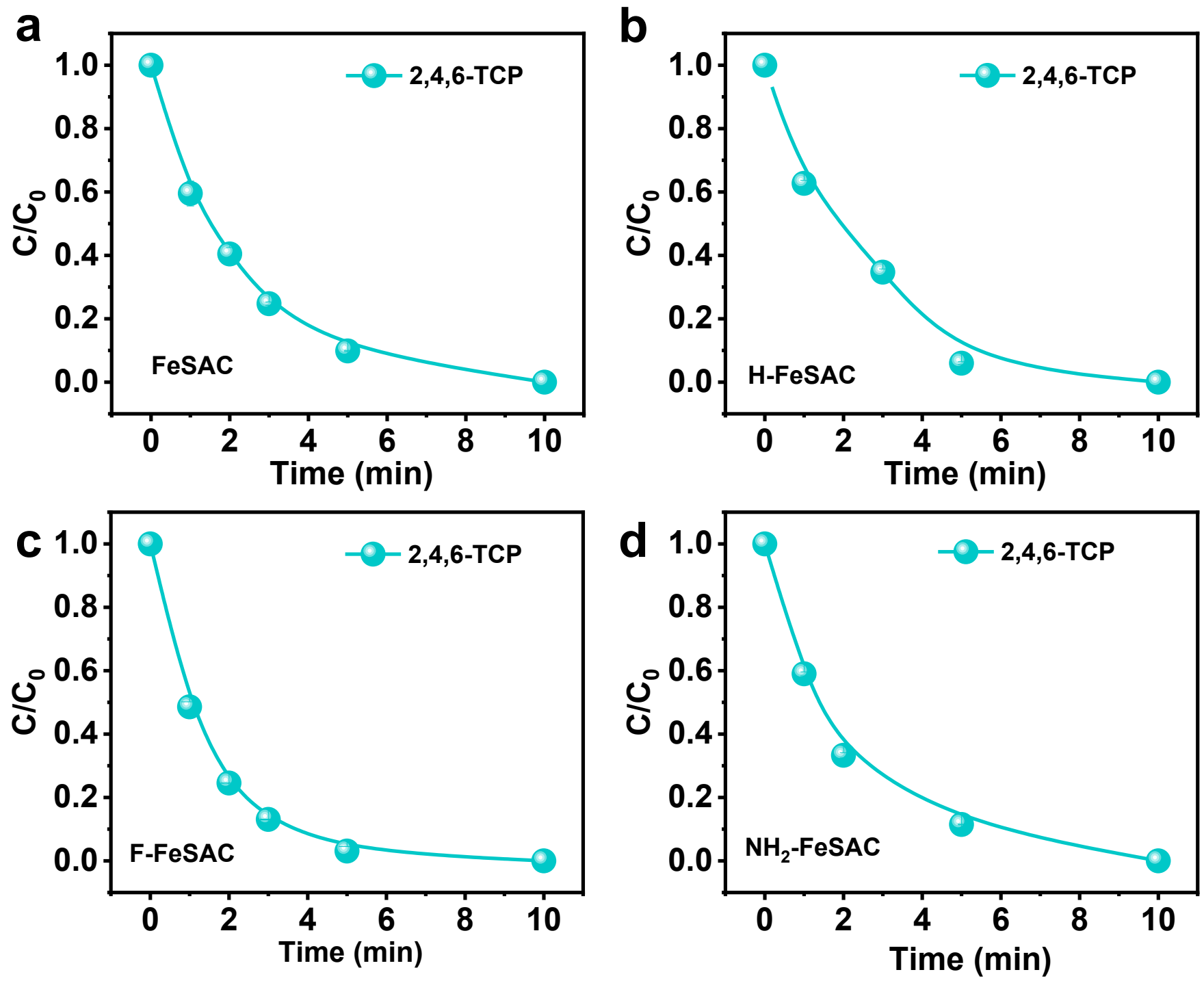

Supplementary Fig. 17 Degradation of 2,4,6-TCP in the (a) FeSAC/PI, (b) H-FeSAC/PI, (c) F-FeSAC/PI and (d) NH_2-FeSAC/PI system.

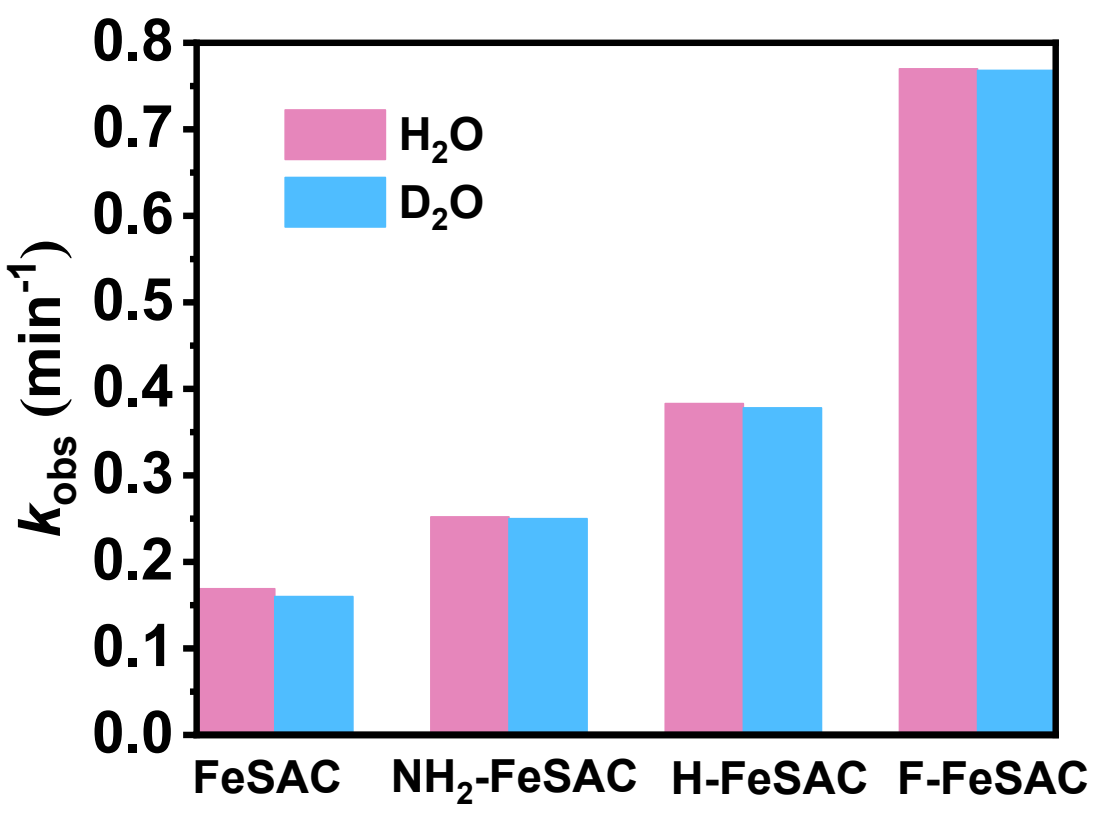

Supplementary Fig. 18 Effect of reaction solvents (H_2O and D_2O) on k_{obs} in different systems.

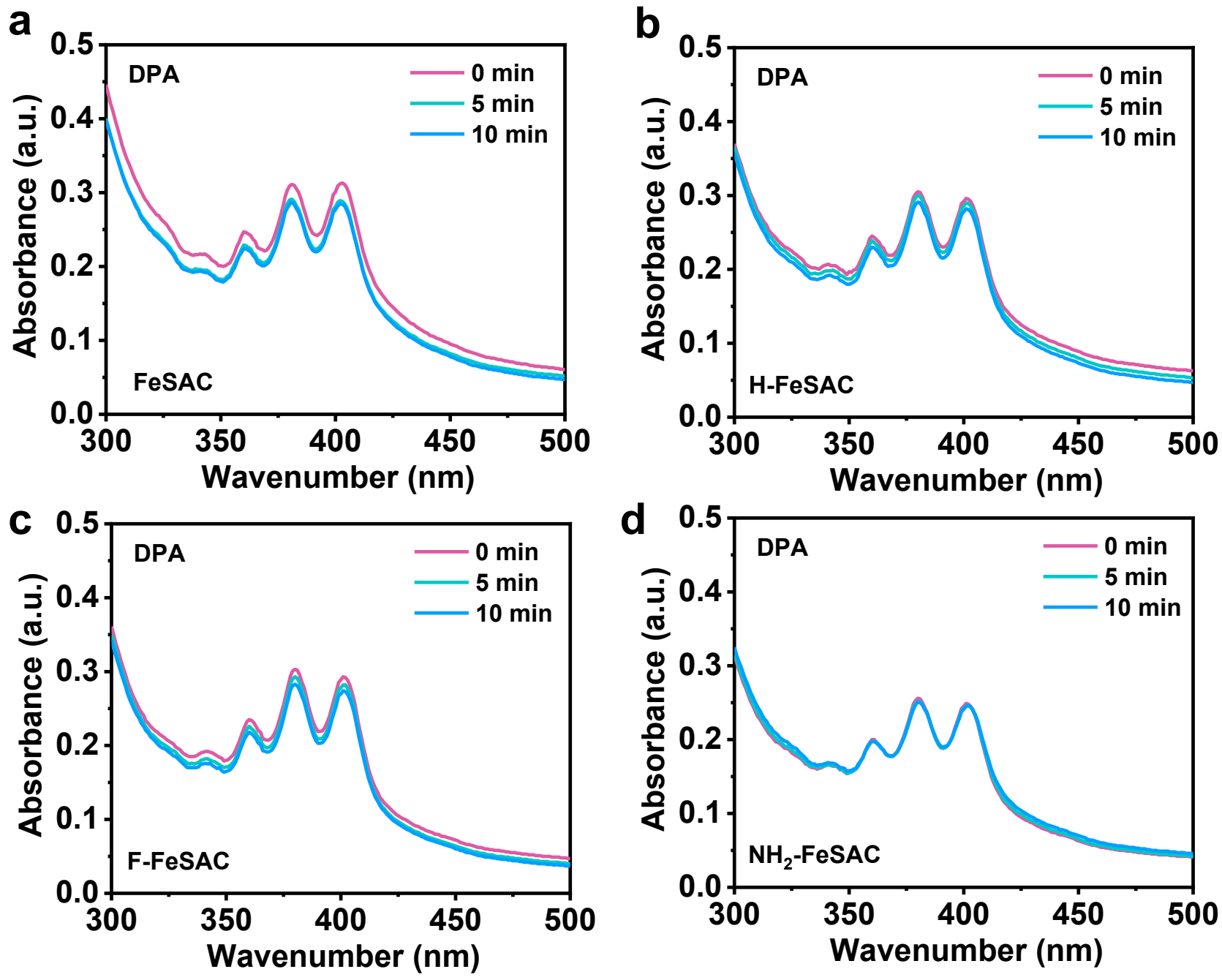

Supplementary Fig. 19 DPA signals in (a) FeSAC/PI, (b) H-FeSAC/PI, (c) F-FeSAC/PI and (d) NH_2-FeSAC/PI systems, Routine conditions: [4-CP] = 80 μM, [catalyst] = 50 mg/L, [PI] = 0.20 mM, temperature = 25 °C, without pH adjustment.

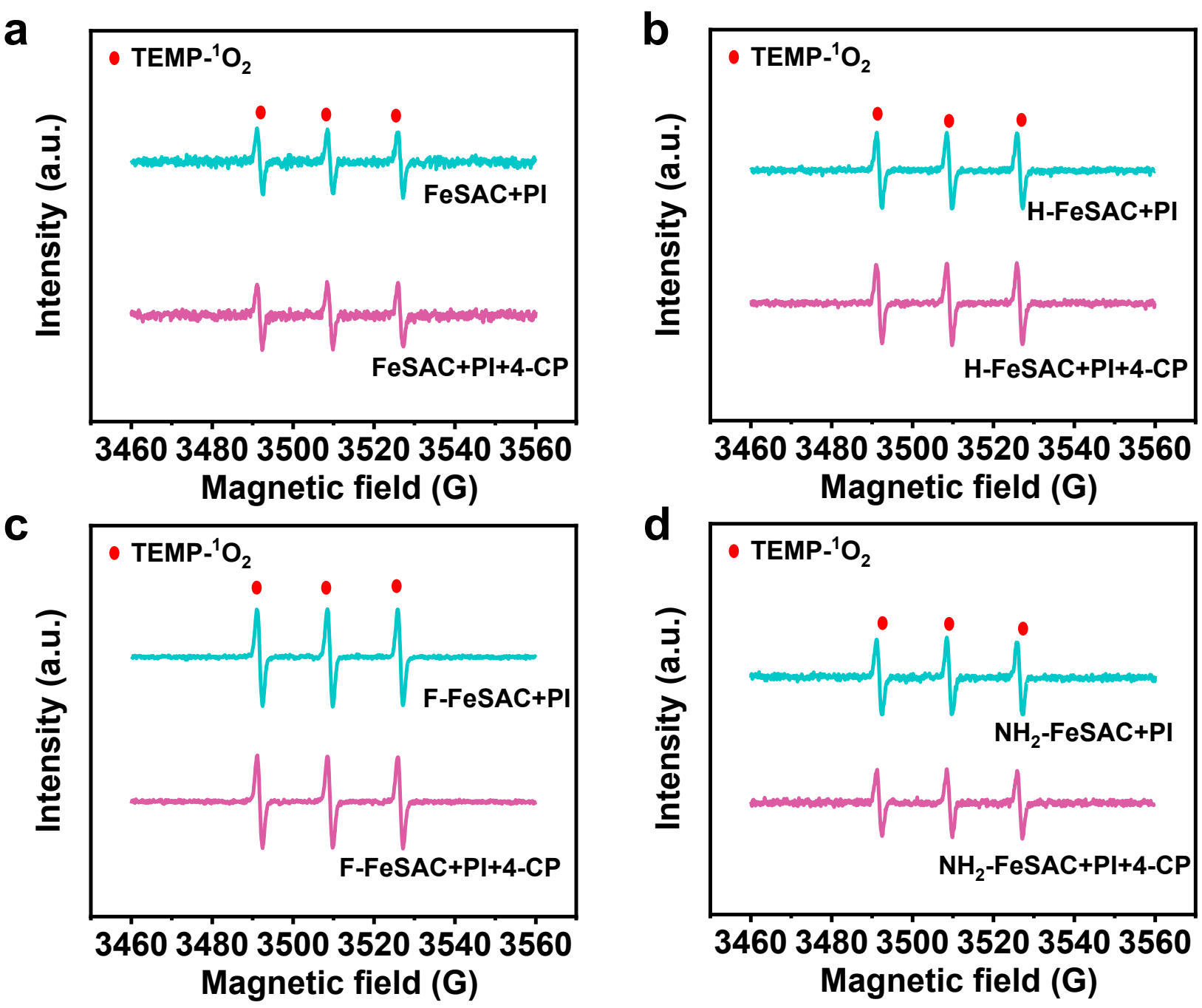

Supplementary Fig. 20 EPR spectra of $^1O_2$ trapped by TEMP in (a) FeSAC/PI, (b) H-FeSAC/PI, (c) F-FeSAC/PI and (d) NH_2-FeSAC/PI system.

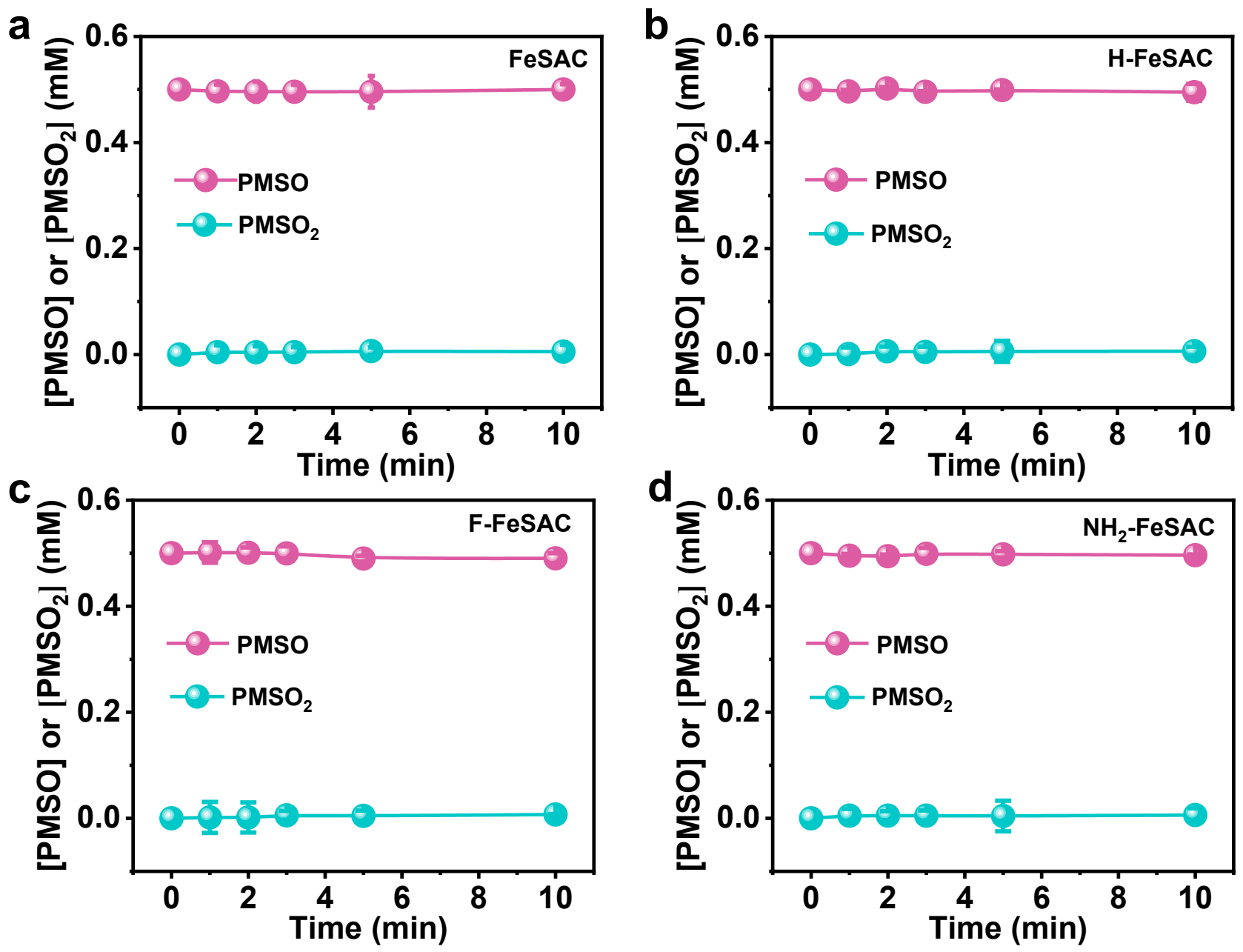

Supplementary Fig. 21 Concentration changes of PMSO and $PMSO_2$ in (a) FeSAC/PI, (b) H-FeSAC/PI, (c) F-FeSAC/PI and (d) NH_2-FeSAC/PI system.

Second, the premixing experiment provides additional mechanistic support for the ETP pathway. When the catalysts and PI were premixed for different periods before the introduction of 4-CP, no discernible change in 4-CP removal was observed (**Fig. 3d; Supplementary Figs. 22a–c**). This result suggests that the oxidation process does not rely on the prior accumulation of freely diffusing reactive oxidizing species. Instead, it is more consistent with a surface-mediated process that is triggered when the pollutant interacts with the catalyst/PI system.

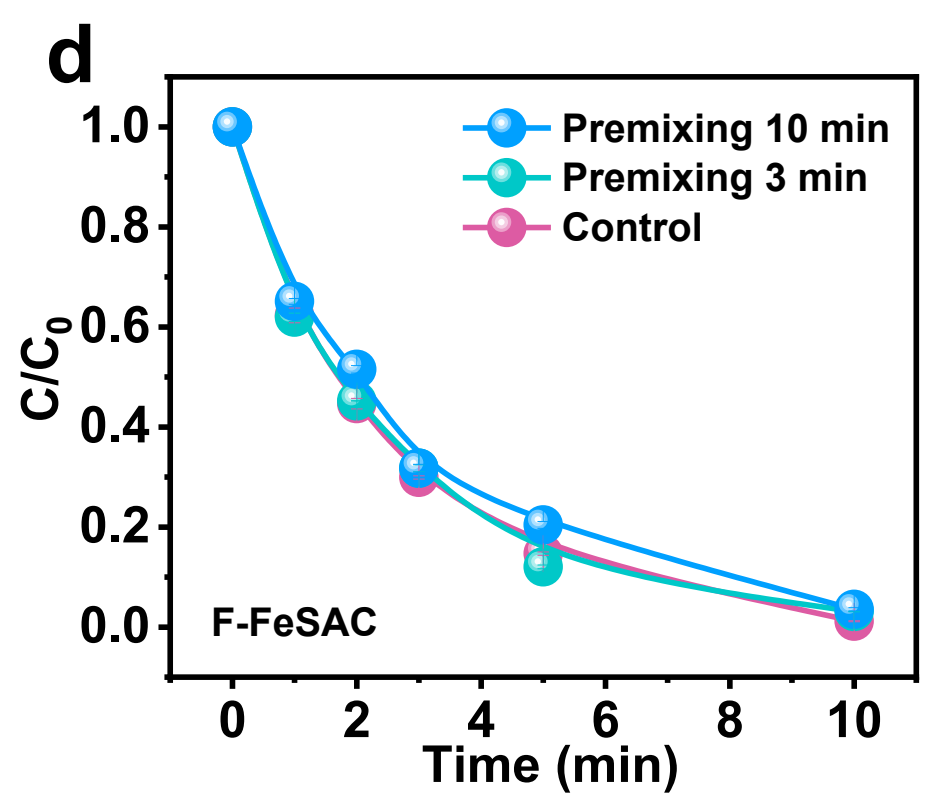

Fig. 3 (d) Effect of premixing on 4-CP removal in the F-FeSAC/PI system.

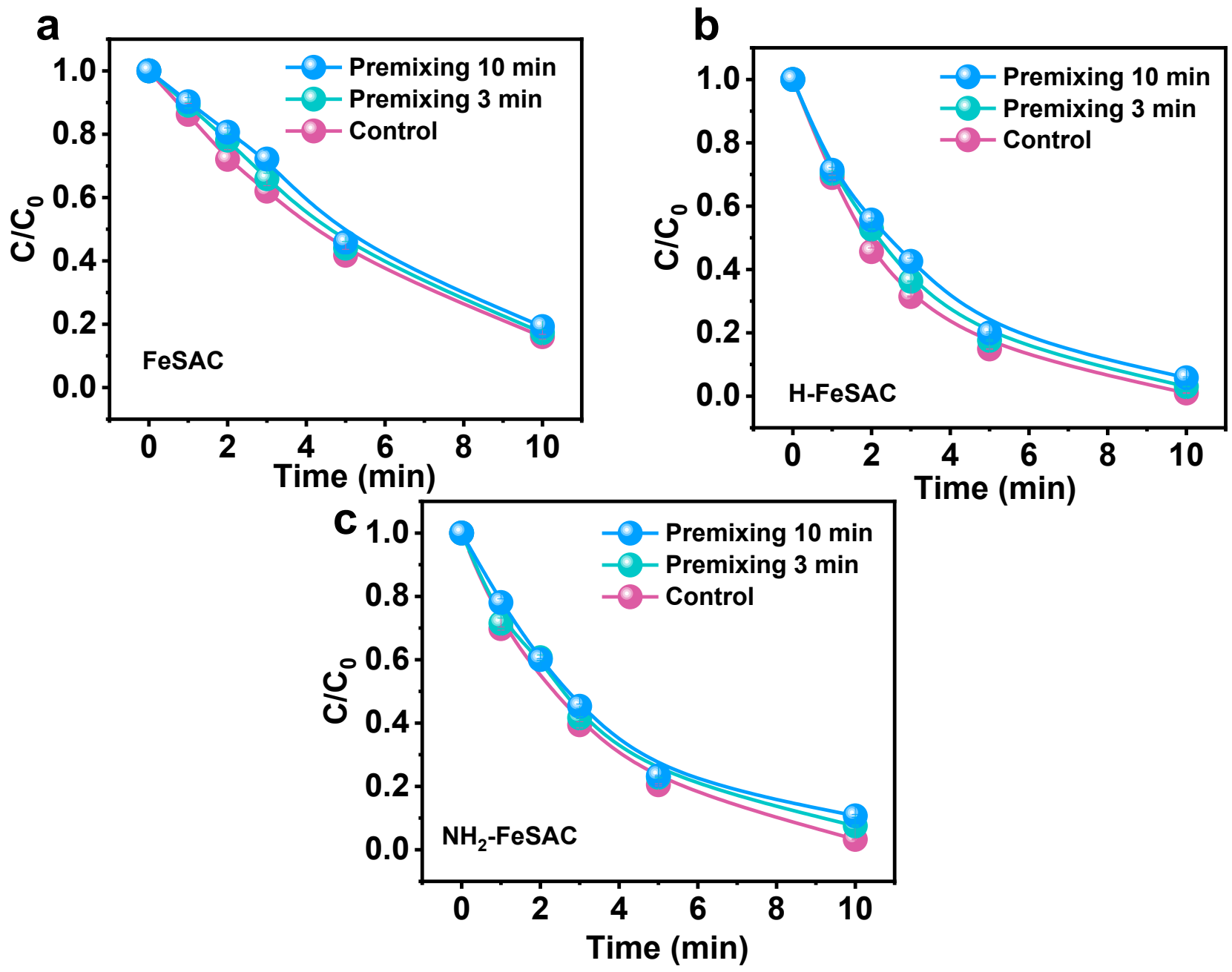

Supplementary Fig. 22 Effect of premixing in the (a) FeSAC/PI, (b) H-FeSAC/PI, (c) NH_2-FeSAC/PI system on 4-CP removal.

Third, we have already provided strong evidence that oxidant consumption is tightly coupled with pollutant oxidation. In the absence of 4-CP, IO_4^- decomposition by R-FeSAC is very limited, whereas the introduction of 4-CP immediately triggers rapid IO_4^- depletion (**Supplementary Fig. 23)**. A strong linear correlation between 4-CP removal and IO_4^- consumption was observed, indicating that pollutant oxidation and oxidant consumption proceed synchronously (**Fig. 3e**). In addition, iodine-speciation analysis shows that IO_4^- is quantitatively converted to IO_3^- without detectable formation of HOI, I^-, or I_3^- (**Fig. 6a-c**). Together, these results indicate that PI functions as the terminal electron-accepting oxidant in the reaction process, rather than serving as a source of reactive iodine intermediates.

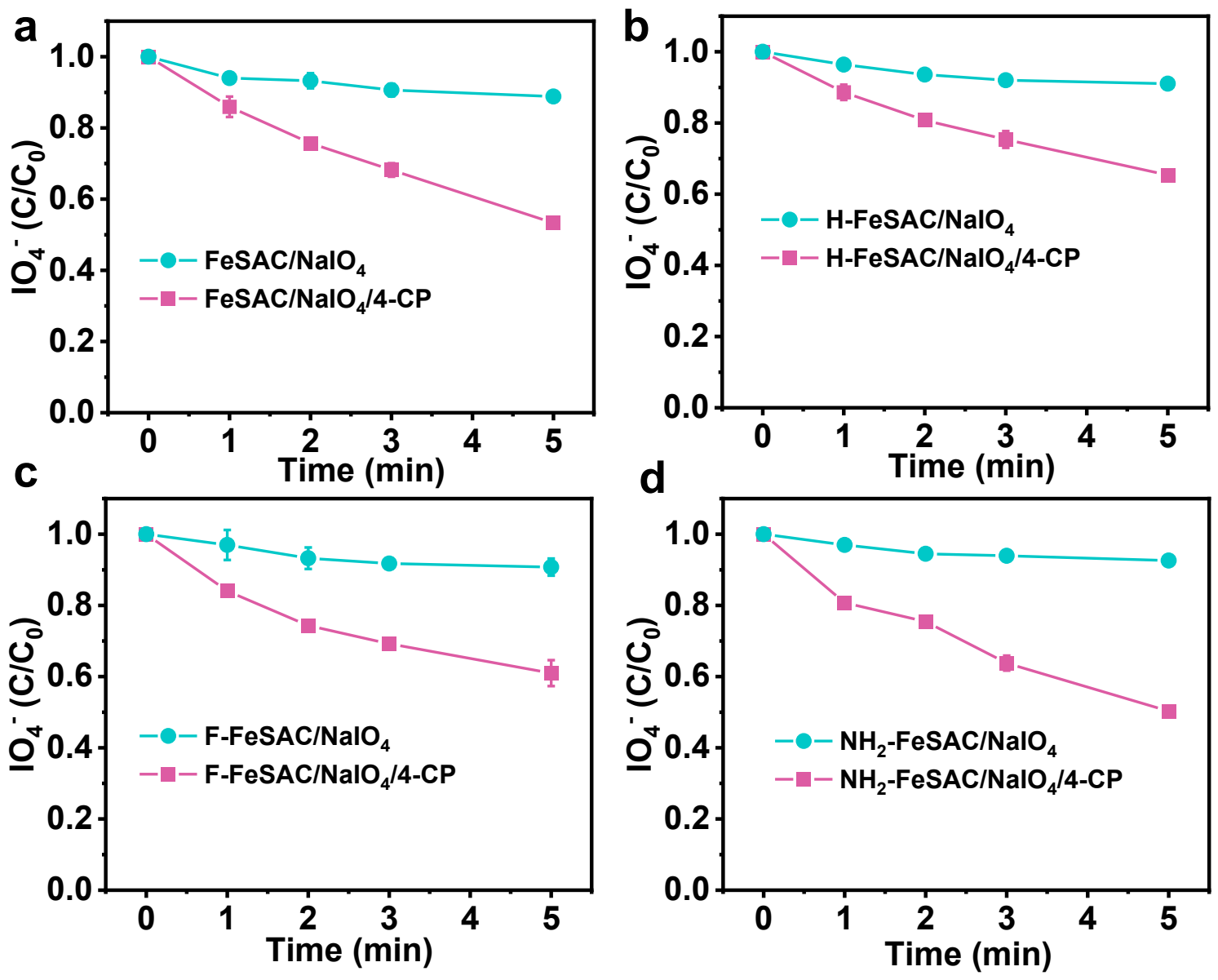

Supplementary Fig. 23 Decomposition rate of PI in (a) FeSAC/PI, (b) H-FeSAC/PI, (c) F-FeSAC/PI and (d) NH_2-FeSAC/PI system.

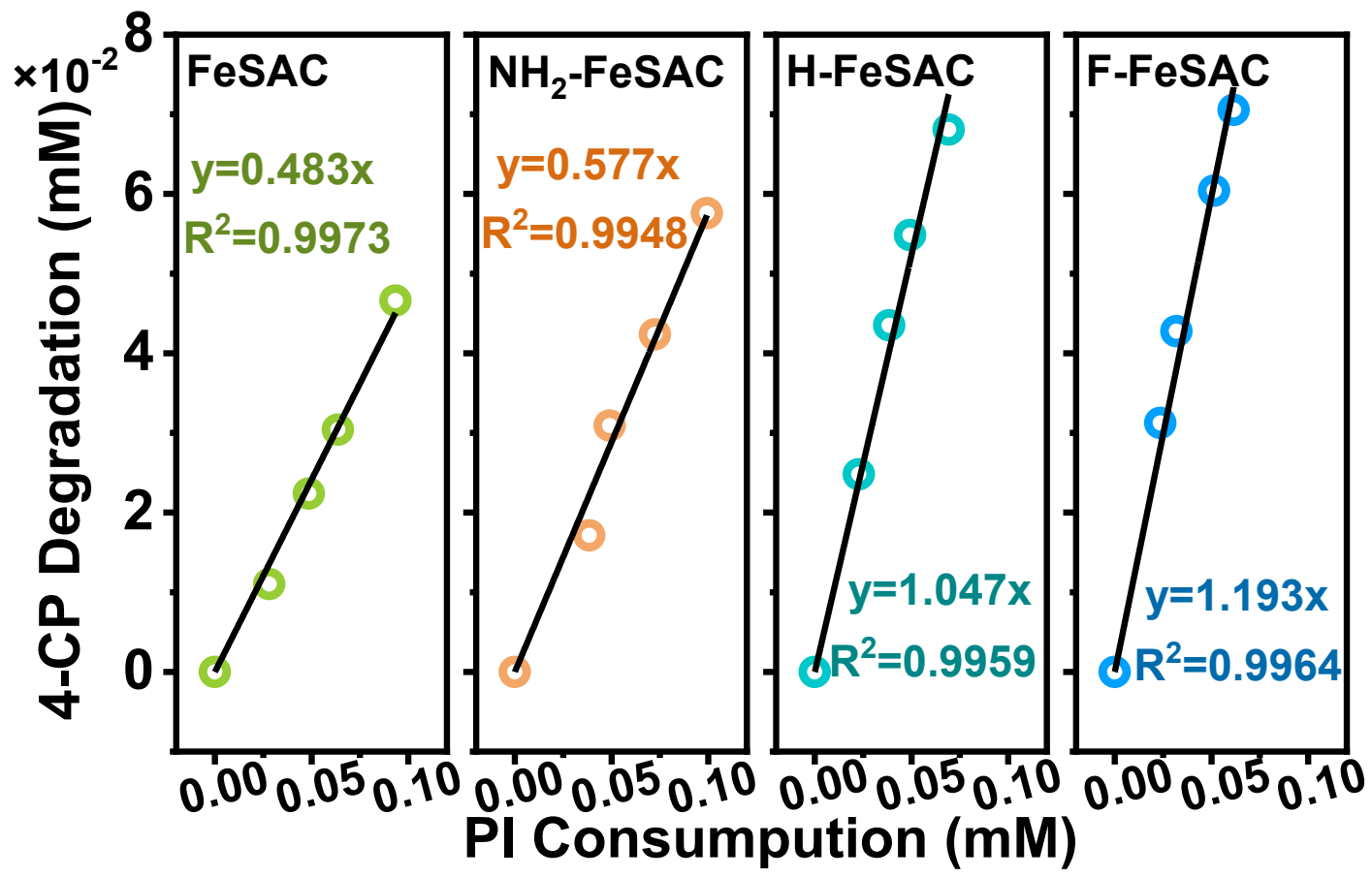

Fig. 3 (e) Correlation between 4-CP degradation and PI consumption in FeSAC/PI, NH_2-FeSAC/PI, H-FeSAC/PI and F-FeSAC/PI systems.

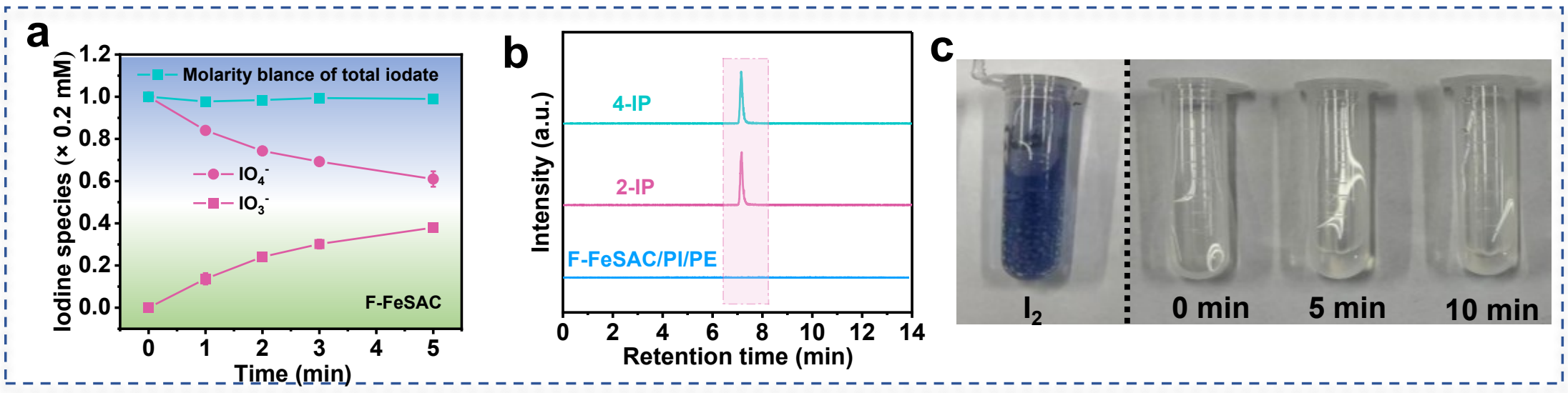

Fig. 6 (a) Concentration changes of IO_4^- and IO_3^- in the F-FeSAC/PI/4-CP system. (b) Detection of HOI generated in the F-FeSAC/PI system using phenol as a trapping agent. (c) Detection of I_2 and I_3^- in the F-FeSAC/PI system.

Fourth, in situ Raman spectroscopy supports the formation and consumption of surface catalyst/PI* complexes. Upon addition of PI to F-FeSAC, a new vibrational feature appears at ~830 cm^{-1}, indicating specific adsorption or coordination of PI on the catalyst surface. After introduction of 4-CP, the corresponding PI-related Raman signal decreases, suggesting the consumption of the surface-associated catalyst/PI* species during pollutant oxidation (**Fig. 3f**). These observations are fully consistent with a surface-mediated electron-transfer process.

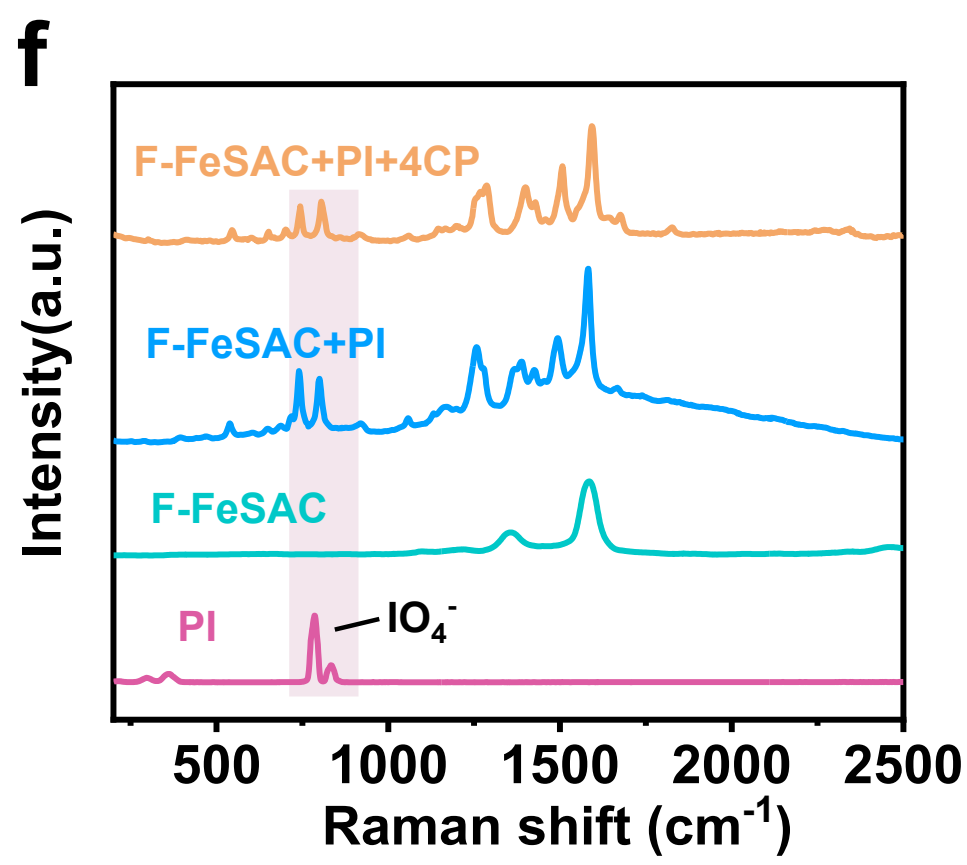

Fig. 3 (f) In situ Raman spectroscopy of different systems.

Fifth, we further strengthened the electrochemical evidence for the proposed ETP mechanism in the R-FeSAC/PI system. In addition to the previously reported i-t, OCP, and EIS results (**Fig. 3g; Supplementary Fig 26),** we additionally performed sequential-addition chronoamperometry (**Fig. R3**) and LSV measurements (**Fig. R4**). The chronoamperometric responses show that PI addition induces the formation of an electroactive catalyst/PI interfacial state, whereas subsequent addition of 4-CP triggers a further current response associated with pollutant-involved interfacial charge-transfer behavior. Among the tested catalysts, F-FeSAC exhibits the most pronounced response, indicating its more favorable PI activation and enhanced interfacial charge-transfer capability relative to H-FeSAC and NH_2-FeSAC. Consistently, the LSV curves show that successive addition of PI and 4-CP significantly alters the electrochemical response of the catalyst-modified electrodes, with F-FeSAC again displaying the strongest electrochemical response. These results suggest that the electron-withdrawing substituent facilitates the formation of a more electrochemically active catalyst/PI interface and promotes interfacial charge transfer during pollutant oxidation. In the revised manuscript, OCP is treated as supportive evidence of interfacial charge-transfer behavior, whereas the electrochemical support for the ETP mechanism is derived from the combined chronoamperometric, OCP, LSV, and EIS results.

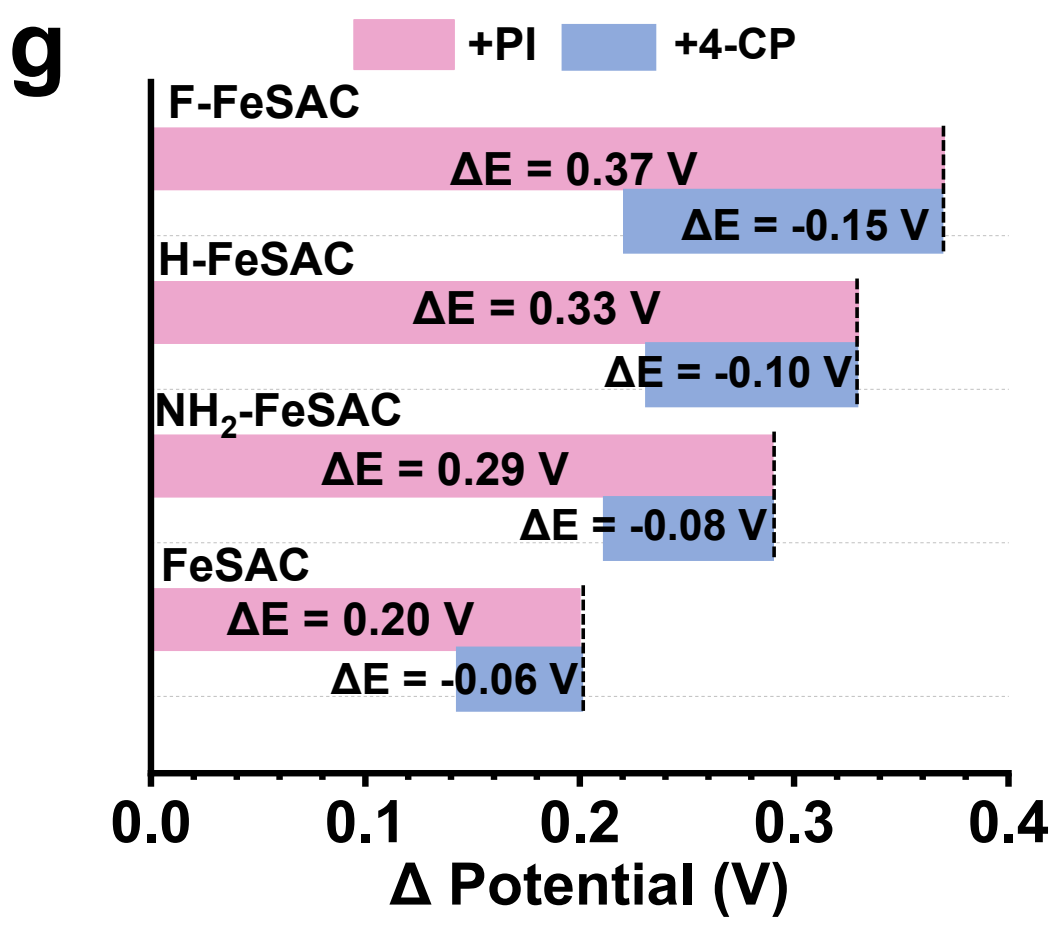

Fig. 3 (g) OCP changes of different systems with the addition of PI and 4-CP.

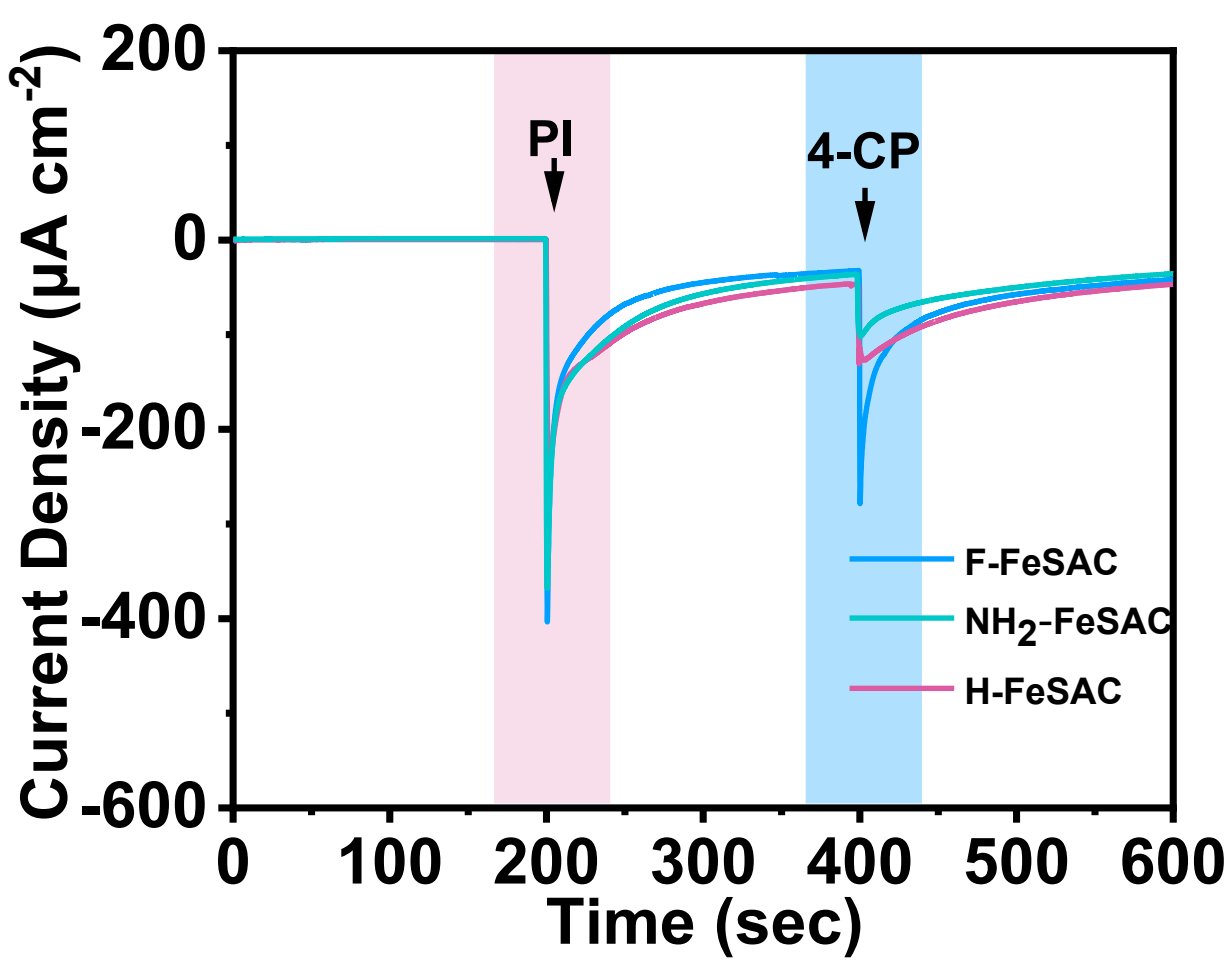

Fig. R3 i-t curve of F-FeSAC, H-FeSAC and NH_2-FeSAC after adding PI and 4-CP successively.

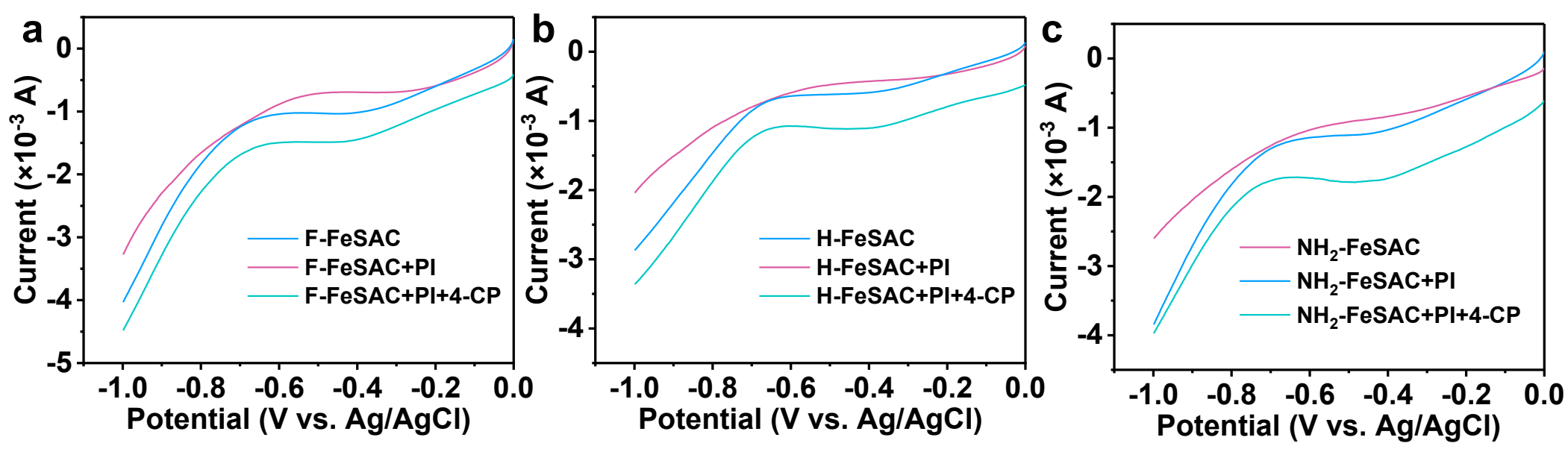

Fig. R4 Linear voltammetry curves (LSV) curve of (a) F-FeSAC, (b) H-FeSAC and (c) NH_2-FeSAC after adding PI and 4-CP successively.

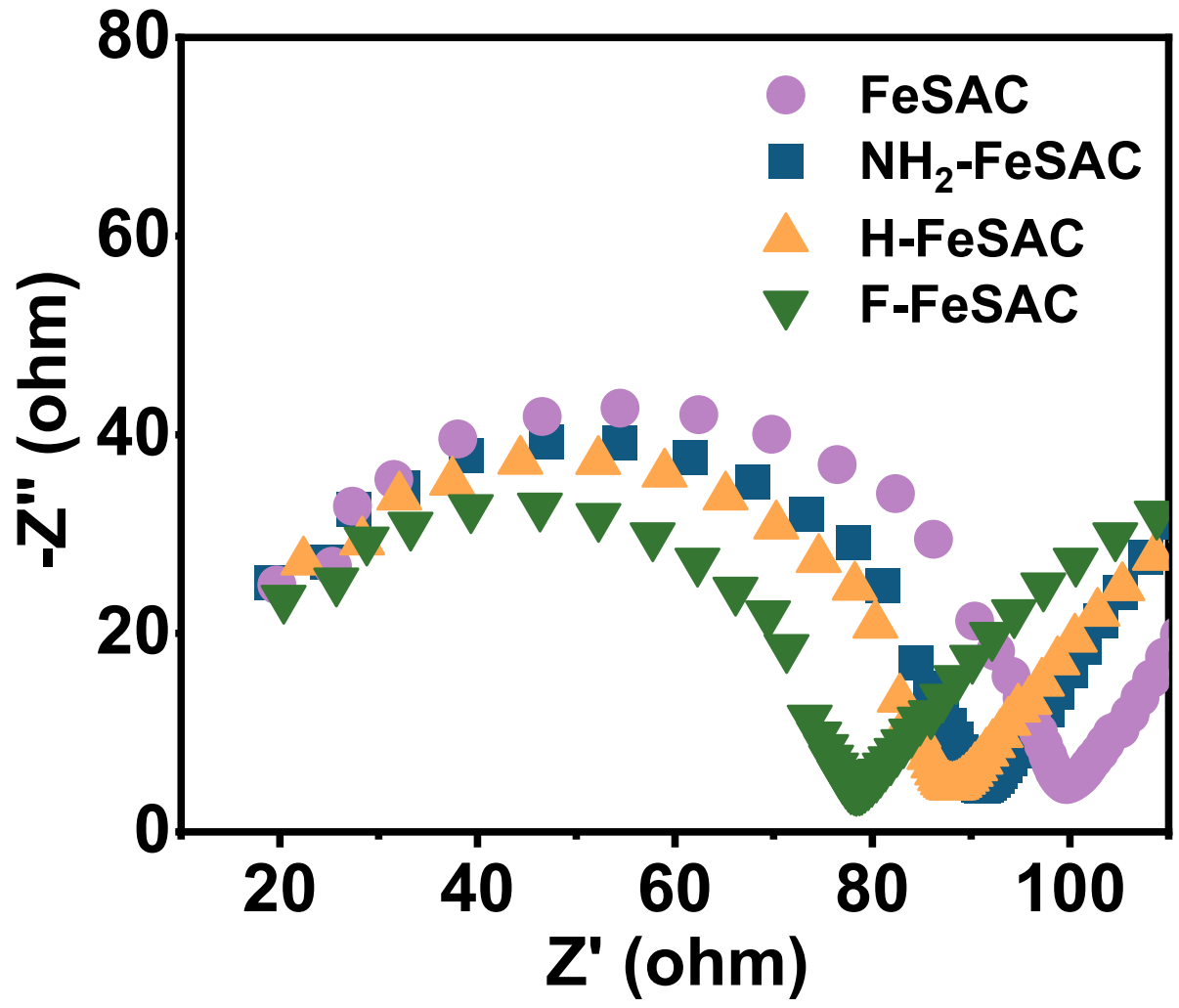

Supplementary Fig. 26 EIS analyses spectra of FeSAC, F-FeSAC, H-FeSAC and NH_2-FeSAC.

Sixth, the galvanic oxidation process (GOP) experiment provides particularly strong mechanistic support. In the dual-chamber system separated by a proton exchange membrane, PI and 4-CP are physically isolated and can only communicate through the external circuit. Under these conditions, the F-FeSAC-based system still generates a substantial current response and achieves significant 4-CP oxidation, whereas the unmodified graphite felt shows negligible activity (**Fig. 3h–j; Supplementary Figs. 27–28**). This dual-chamber reactor design eliminates direct molecular contact between PI and 4-CP and thereby provides strong evidence for an electron-transfer-mediated oxidation pathway.

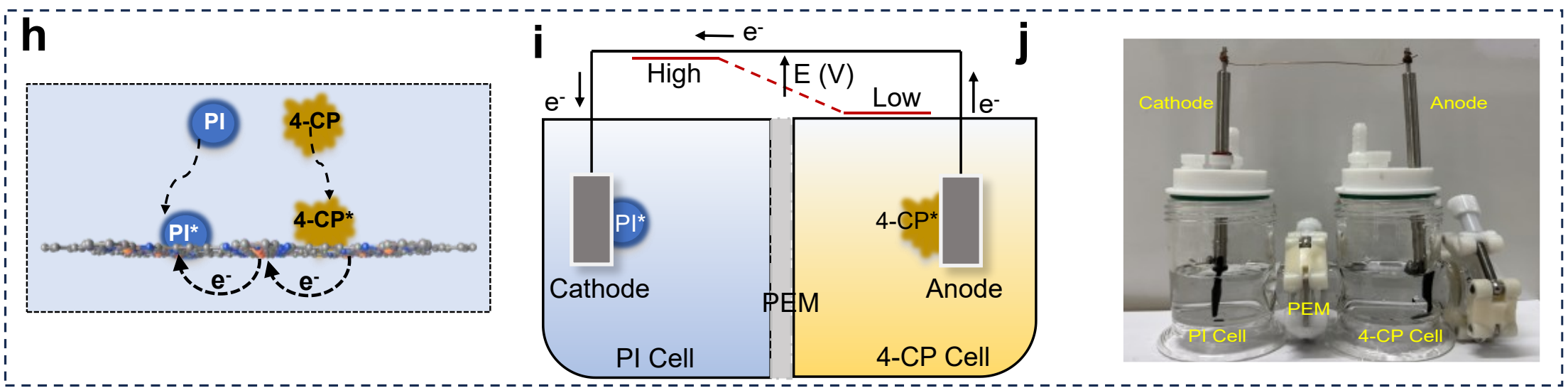

Fig. 3 (h) Schematic illustration of the electron transfer process. (i) Schematic diagram of the concept design and (j) photograph of the double-chamber reactor developed for R-FeSAC/GF performance evaluation.

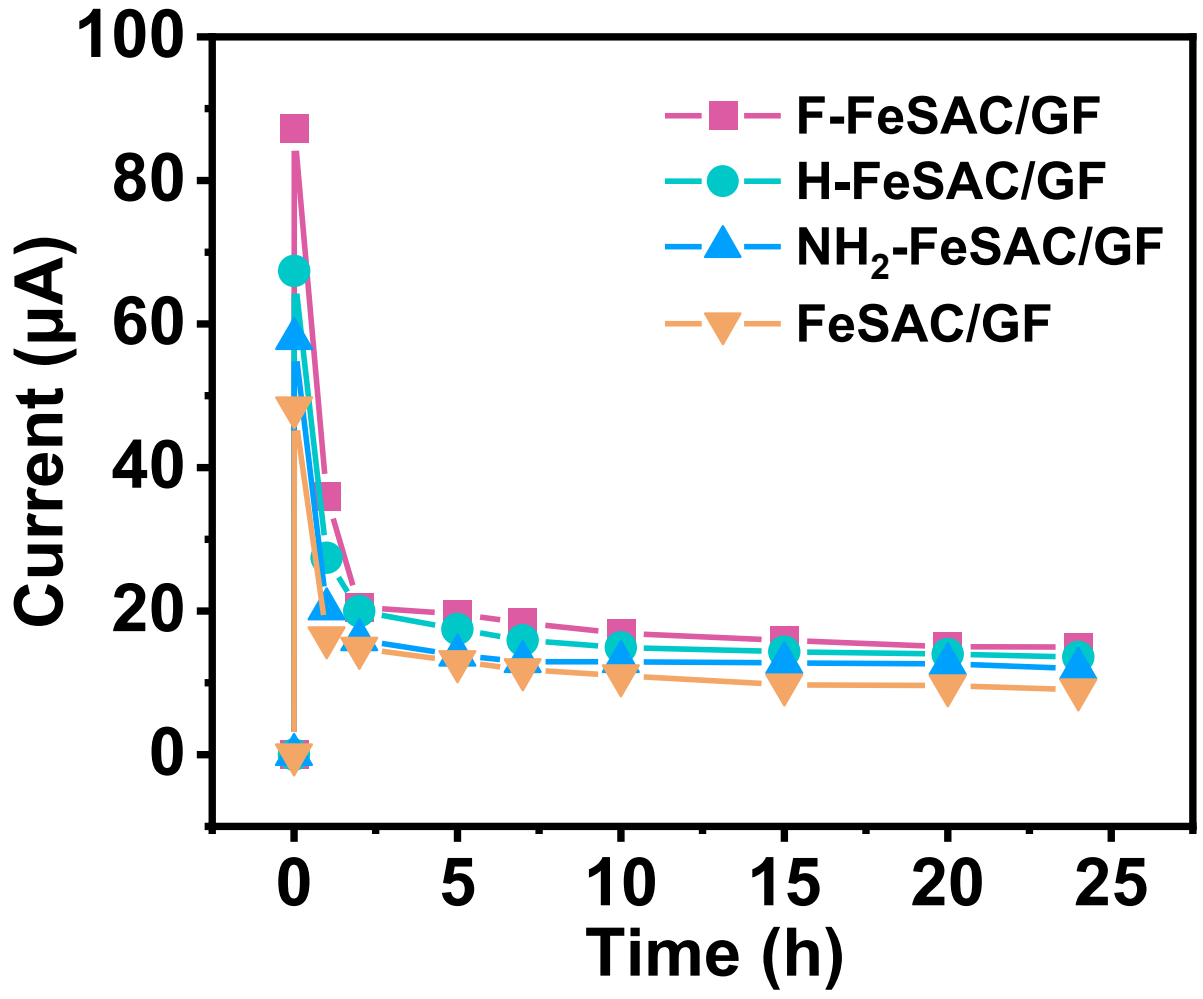

Supplementary Fig. 27 Measurement of current-time curves on the FeSAC/GF, F-FeSAC/GF, H-FeSAC/GF and NH_2-FeSAC/GF electrode.

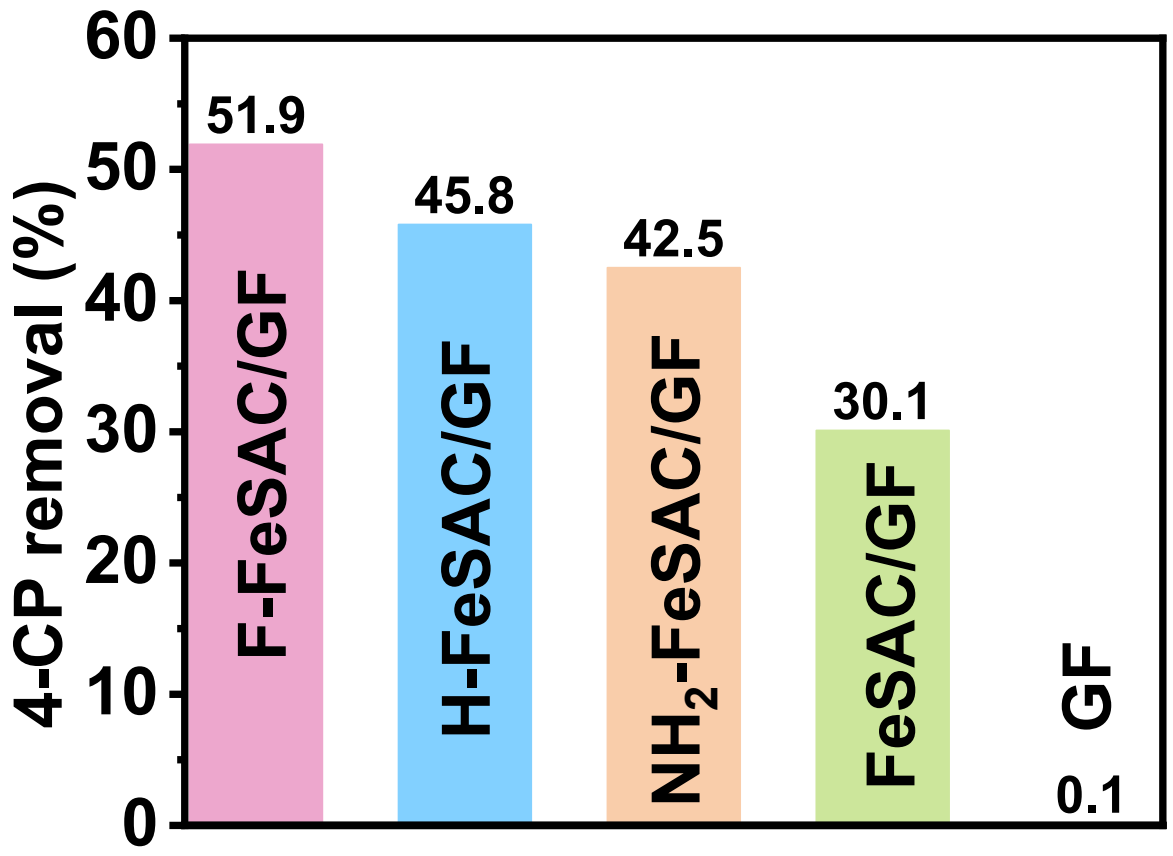

Supplementary Fig. 28 Removal rates of 4-CP in different GOP systems.

Seventh, the pollutant-dependent reactivity is also consistent with the ETP mechanism. The degradation rates of eight representative pollutants correlate strongly with their half-wave potentials ($\varphi_{1/2}$), and electron-rich pollutants exhibit much faster degradation than electron-poor substrates such as nitrobenzene and benzoic acid (**Fig. 3k**). This behavior is inconsistent with a nonspecific radical-driven oxidation pathway and instead shows that pollutant degradation is strongly governed by electron-donating ability, consistent with an electron-transfer-controlled process.

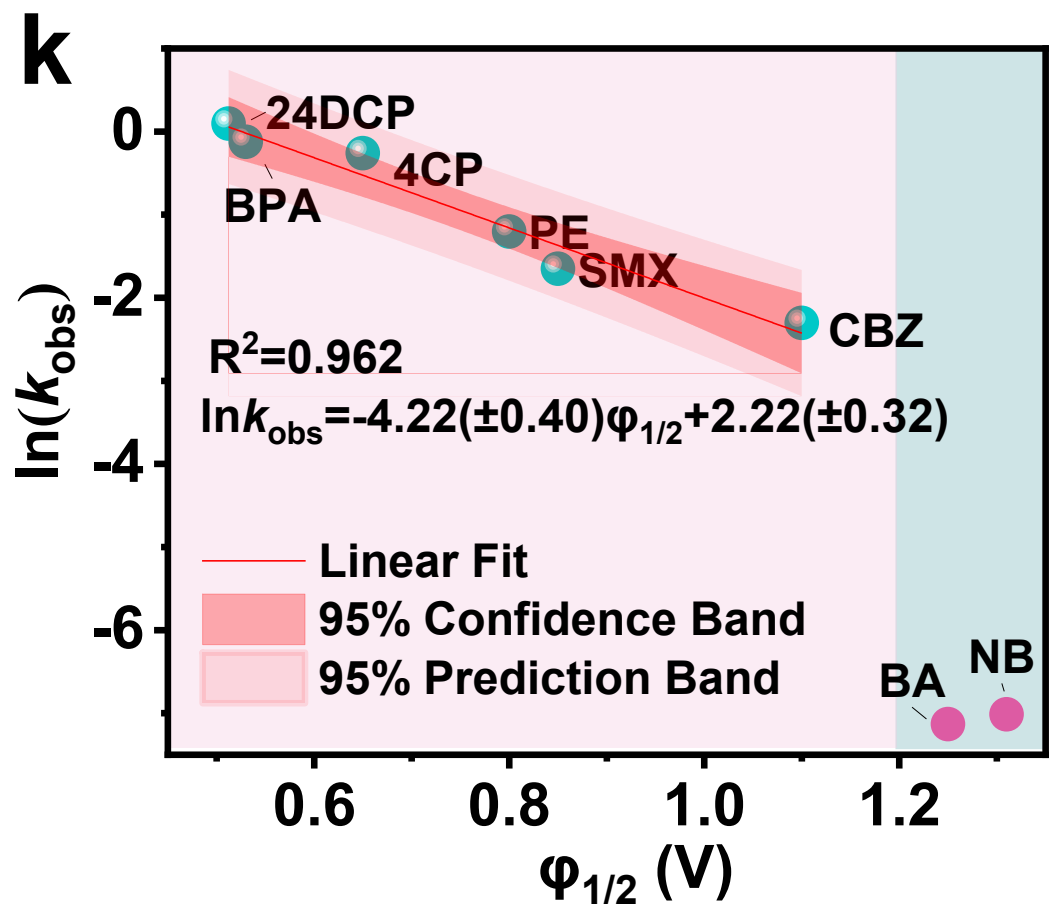

Fig. 3 (k) Correlation between the $\varphi_{1/2}$ values of eight contaminants and their ln(k_{obs}).

Finally, we have further strengthened the theoretical analysis to address the reviewer's concern regarding directional electron transfer. Together with the HOMO/LUMO, PDOS, charge-density-difference, adsorption energy, and Fe–O bond-length analyses (**Fig. 4a–h**), the charge-transfer results reveal the most pronounced interfacial electron redistribution in F-FeSAC/PI. This is consistent with its lower LUMO level, stronger PI adsorption, reduced electron-transfer barrier, and highest catalytic activity.

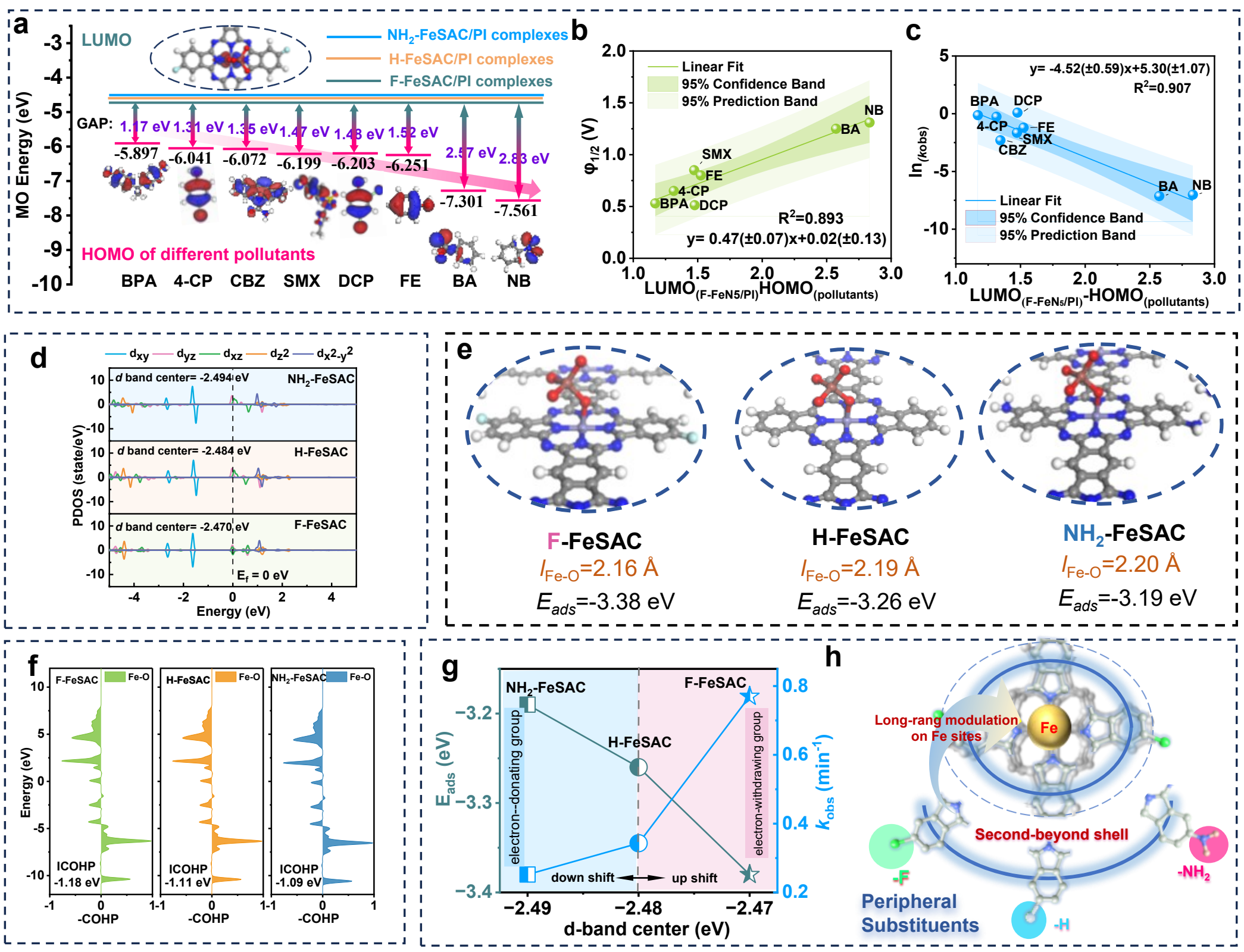

Fig. 4 | The mechanistic of substituents beyond the second coordination sphere in

regulating Fe active sites. (a) Energy gap between $LUMO_{(R\text{-}FeSAC/PI)}$ and $HOMO_{pollutants}$. (b) Linear correlation between $LUMO_{(R\text{-}FeSAC/PI)}$-$HOMO_{pollutants}$ and $\varphi_{1/2}$ values of pollutants. (c) Linear correlation between $LUMO_{(R\text{-}FeSAC/PI)}$-$HOMO_{pollutants}$ and ln(k_{obs}). (d) Projected Density of States of the Fe 3*d* orbitals in F-FeSAC, H-FeSAC, and NH_2-FeSAC. (e) Optimized adsorption configurations of PI at Fe site on the F-FeSAC, H-FeSAC, and NH_2-FeSAC. (f) Crystal orbital Hamilton population (COHP) analysis of Fe-O when PI adsorbs on Fe. (g) Correlations among k_{obs}, E_{ads}, and *d*-band center for F-FeSAC, H-FeSAC, and NH_2-FeSAC. (h) Schematic illustration of long-range electronic modulation at Fe sites induced by substituent groups beyond the secondary coordination sphere.

Therefore, in the revised manuscript, the ETP mechanism is no longer assigned on the basis of exclusion experiments or OCP alone. Instead, it is supported collectively by: (i) exclusion of radical, $^1O_2$, high-valent metal-oxo, and reactive iodine intermediate pathways; (ii) the premixing experiment excluding a mechanism dominated by pre-accumulated freely diffusing oxidizing species; (iii) synchronous pollutant oxidation and PI consumption, together with quantitative conversion of IO_4^- to IO_3^-; (iv) in situ Raman evidence for catalyst/PI* surface complexes; (v) combined electrochemical evidence from chronoamperometry, OCP, LSV, and EIS; (vi) the galvanic oxidation experiment in a spatially separated dual-chamber system; (vii) pollutant structure-reactivity correlations based on $\varphi_{1/2}$; and (viii) DFT-based charge-density-difference and Bader charge analyses. We believe that this revised evidence chain addresses the reviewer's concern and provides a substantially stronger basis for the electron-transfer pathway proposed in this work.

6.The classical d-band theory does not directly reveal d-band upshift and reduced antibonding occupation without detailed electronic structure analysis. The authors should consider COHP (Crystal Orbital Hamilton Population) analysis and Orbital-resolved DOS discussion.

Response 6:

We sincerely thank the reviewer for this important and constructive comment. We agree that the classical *d*-band model alone is insufficient to support the discussion of d-band upshift and antibonding-state occupation. In the revised manuscript, we therefore strengthened this part by combining *d*-band-center calculations with orbital-resolved DOS of the catalysts, together with PDOS and COHP/ICOHP analyses of the PI-adsorbed systems.

The calculated Fe *d*-band centers of F-FeSAC, H-FeSAC, and NH_2-FeSAC are −2.470, −2.484, and −2.494 eV, respectively (**Fig. R5**). This order shows that F-FeSAC has the Fe 3*d* states nearest to the Fermi level among the three catalysts, providing the most favorable electronic basis for subsequent interaction with PI. More importantly, the orbital-resolved DOS reveals that the peripheral substituents distinctly regulate the distribution of the Fe frontier 3*d* orbitals near the Fermi level. In particular, the dz^2 and d_{xz}/d_{yz} components, which are closely related to the subsequent Fe–O interfacial interaction with adsorbed PI, show clear substituent-dependent differences. Compared with H-FeSAC and NH_2-FeSAC, F-FeSAC exhibits the most favorable frontier-orbital distribution around the Fermi level, indicating that the F substituent most effectively tunes the Fe center for interaction with PI.

We then analyzed the PI-adsorbed systems to directly probe the Fe–O interaction. The PDOS results show evident overlap between Fe-*d* and O-*p* states around the Fermi level after PI adsorption, indicating appreciable Fe–O orbital hybridization (**Fig. R6**). This is further supported by the COHP/ICOHP analysis (**Fig. R7**). Among the three systems, F-FeSAC-PI exhibits the shortest Fe–O bond length (2.16 Å), compared with 2.19 Å for H-FeSAC-PI and 2.20 Å for NH_2-FeSAC-PI. Consistently, the ICOHP value of F-FeSAC-PI is the most negative (−1.18 eV), compared with −1.11 eV for H-FeSAC-PI and −1.09 eV for NH_2-FeSAC-PI, indicating the strongest Fe–O bonding interaction in the F-substituted system. In addition, the -COHP profile of F-FeSAC-PI shows weaker occupied antibonding contribution near the Fermi level than those of the H- and NH_2-substituted counterparts, in line with its stronger Fe–O bond.

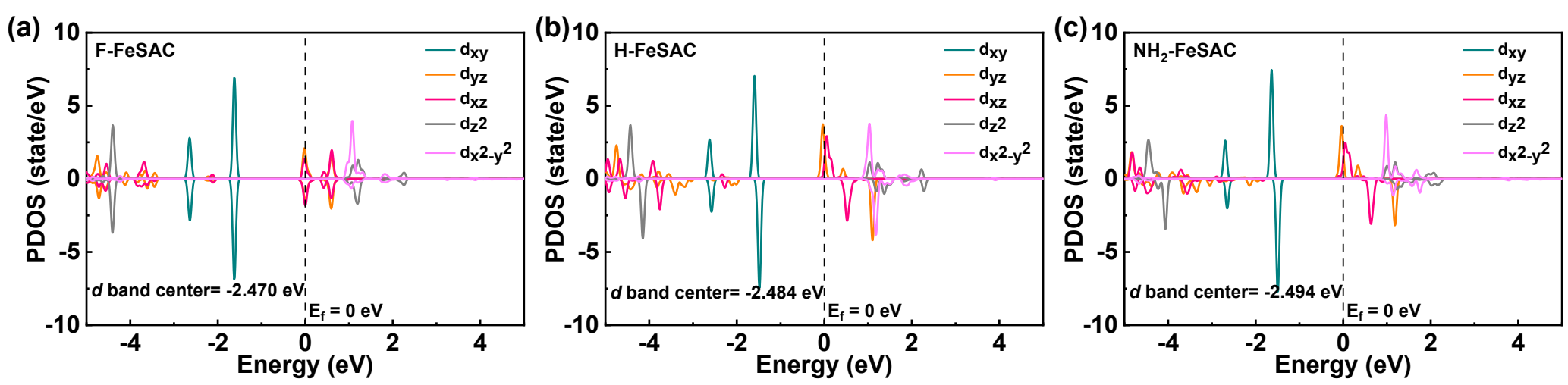

Fig. R5 Projected Density of States of the Fe 3*d* orbitals in (a) F-FeSAC, (b)H-FeSAC and (c)NH_2-FeSAC.

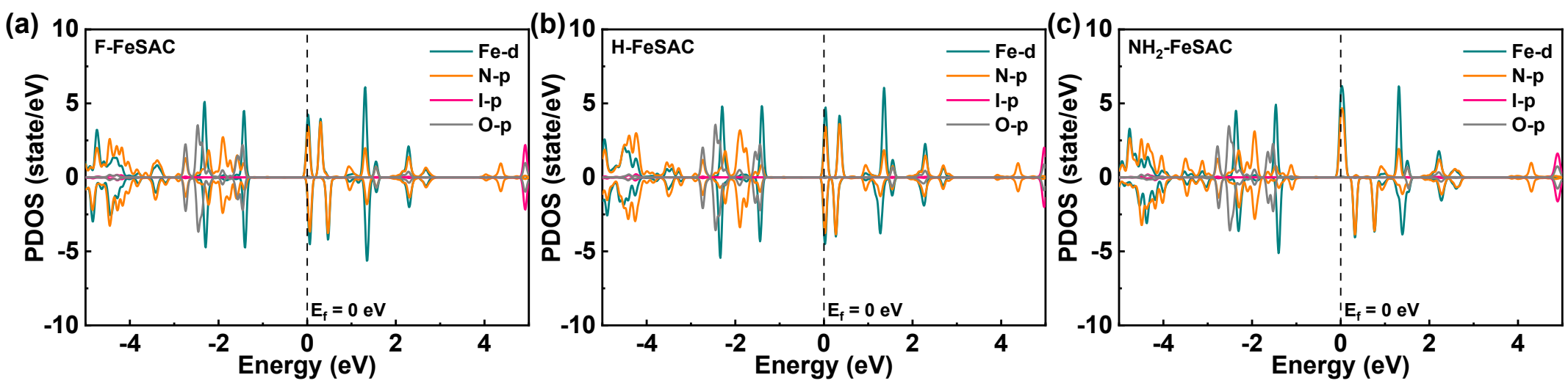

Fig. R6 Projected density of states (PDOS) of PI-adsorbed (a) F-FeSAC, (b) H-FeSAC, and (c) NH_2-FeSAC.

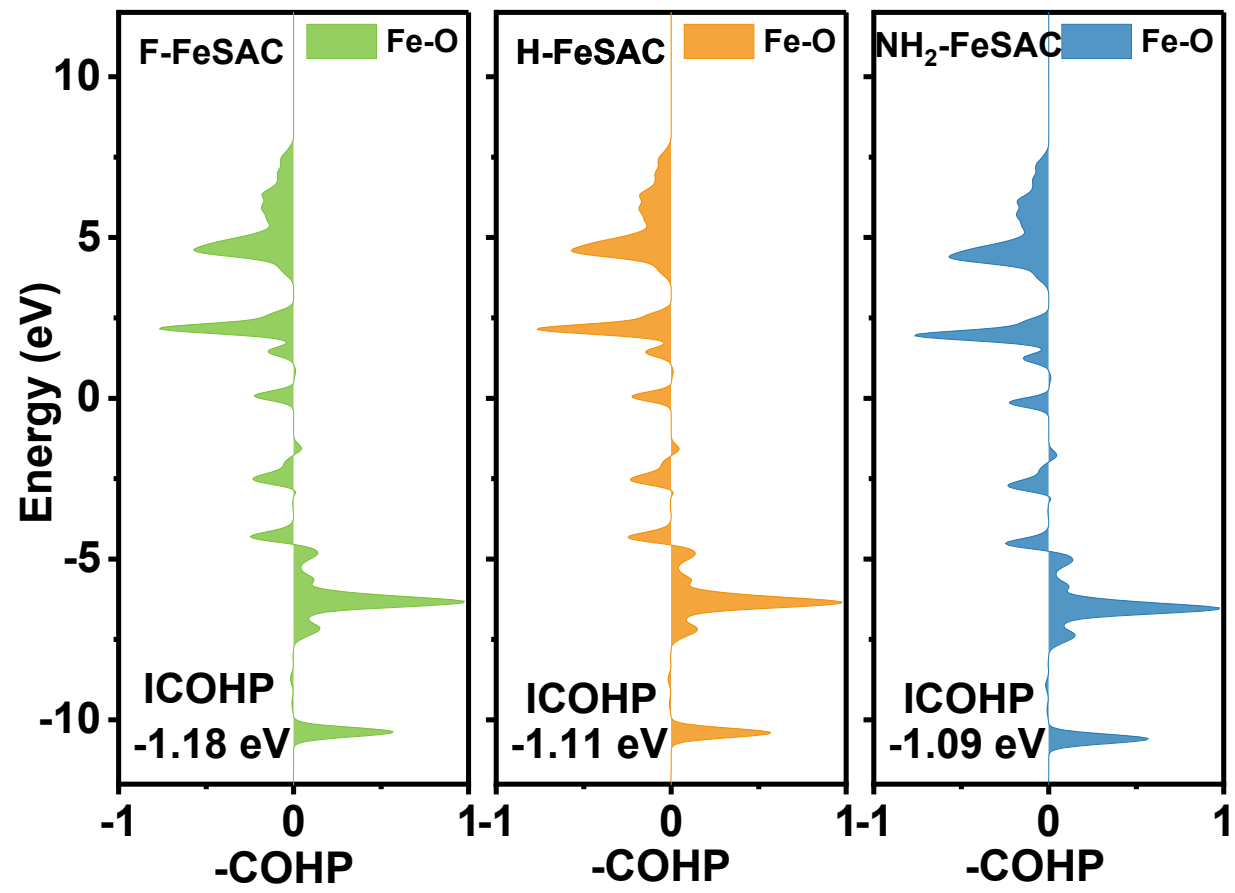

Fig. R7 Crystal orbital Hamilton population (COHP) analysis of Fe-O when PI adsorbs on Fe.

Action 6: We have added the relevant discussion in the revised manuscript (Fig. 4d–f) and Supporting Information (*Supplementary Fig. 32*).

*"The calculated Fe d-band centers of F-FeSAC, H-FeSAC, and NH_2-FeSAC are −2.470, −2.484, and −2.494 eV, respectively (**Fig. 4d**). This order shows that F-FeSAC has the Fe 3d states nearest to the Fermi level among the three catalysts, providing a more favorable electronic configuration for subsequent interaction with PI. The orbital-resolved DOS further reveals substituent-dependent regulation of the Fe frontier 3d orbitals near the Fermi level. In particular, the dz^2 and d_{xz}/d_{yz} components, which are closely related to the subsequent Fe–O interfacial interaction with adsorbed PI, show clear substituent-dependent differences. Compared with H-FeSAC and NH_2-FeSAC, F-FeSAC exhibits a most favorable frontier-orbital distribution around the Fermi level, indicating that the F substituent more effectively tunes the Fe center for interaction with PI.*

We then analyzed the PI-adsorbed systems to directly probe the Fe–O interaction. The PDOS results show evident overlap between Fe-d and O-p states around the Fermi

*level after PI adsorption, indicating appreciable Fe–O orbital hybridization (**Supplementary Fig. 32**). This is further supported by the COHP/ICOHP analysis (**Fig. 4f**). Among the three systems, F-FeSAC-PI exhibits the shortest Fe–O bond length (2.16 Å), compared with 2.19 Å for H-FeSAC-PI and 2.20 Å for NH_2-FeSAC-PI. Consistently, the ICOHP value of F-FeSAC-PI is the most negative (−1.180 eV), compared with −1.112 eV for H-FeSAC-PI and −1.094 eV for NH_2-FeSAC-PI, indicating the strongest Fe–O bonding interaction in the F-substituted system (**Fig. 4e-f**). In addition, the -COHP profile of F-FeSAC-PI shows weaker occupied antibonding contribution near the Fermi level than those of the H- and NH_2-substituted counterparts, in line with its stronger Fe–O bond."*

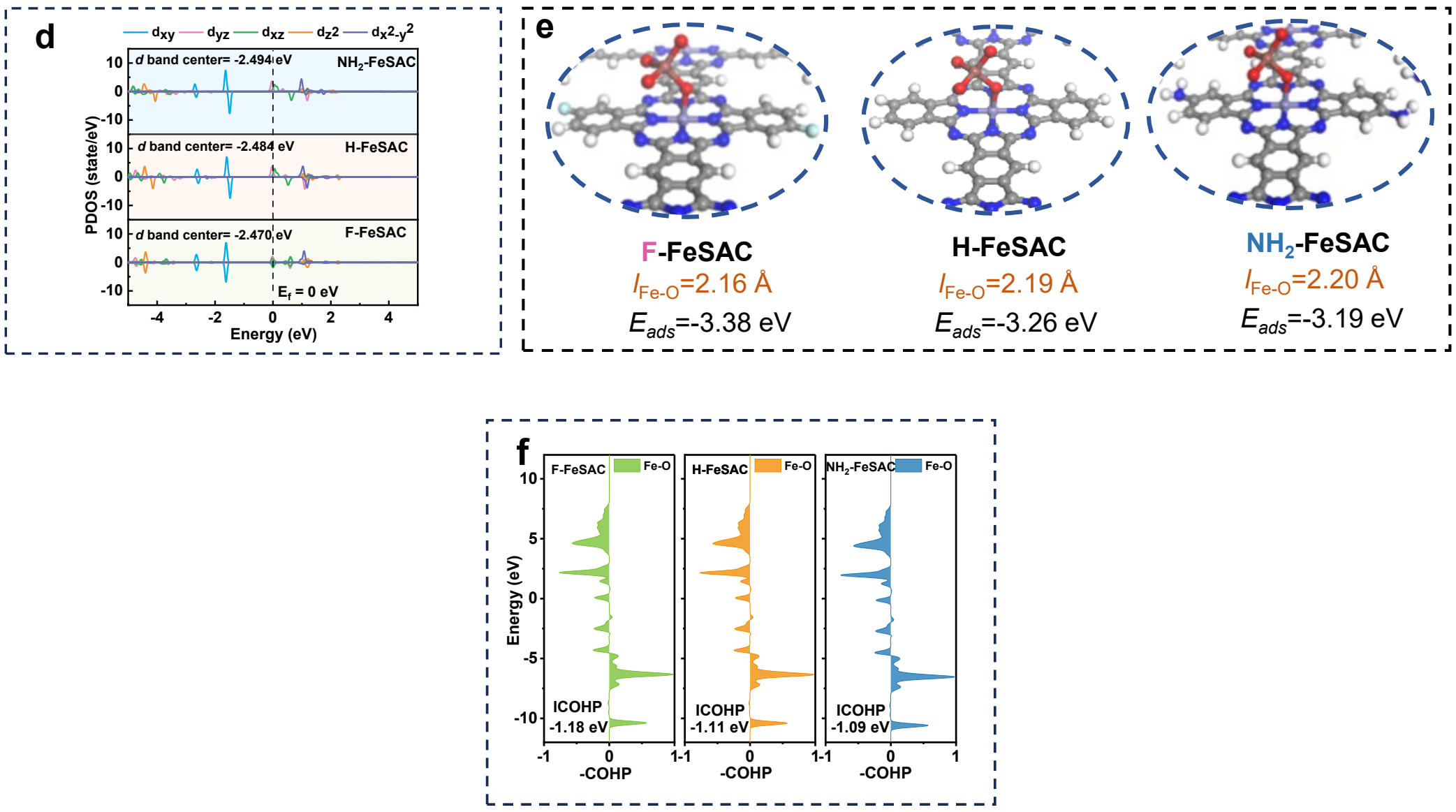

Fig. 4 (d) Projected Density of States of the Fe 3*d* orbitals in F-FeSAC, H-FeSAC, and NH_2-FeSAC. (e) Optimized adsorption configurations of PI at Fe site on the F-FeSAC, H-FeSAC, and NH_2-FeSAC. (f) Crystal orbital Hamilton population (COHP) analysis of Fe-O when PI adsorbs on Fe.

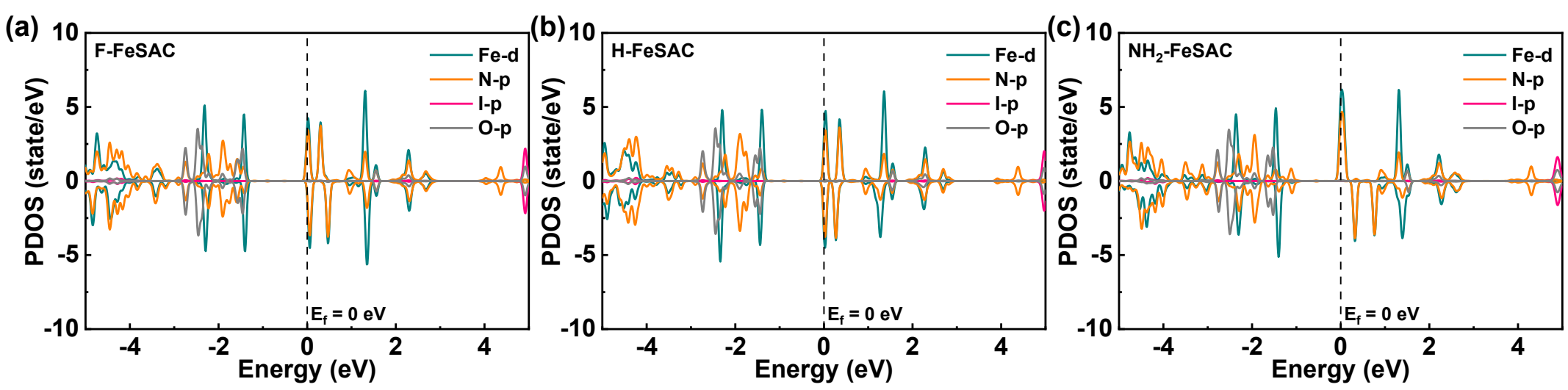

Supplementary Fig. 32 Projected density of states (PDOS) of PI-adsorbed (a) F-FeSAC, (b) H-FeSAC, and (c) NH_2-FeSAC.

7.The reference to “PMS electron acceptor” in line 579 contradicts with the use of PI in Fig. 5m.

Response 7:

We thank the reviewer for this careful comment. We sincerely apologize for this oversight. This error in line 579 was incorrect and inconsistent with the use of PI in Fig. 5m.

Action 7: We have the added relevant statements in the revised manuscript.

“Concurrently, the PI electron acceptor was consumed in the dedicated chambers, with only ~40% remaining (**Fig. 5m**).”

m

Fig. 5 (m) 4-CP removal efficiency and PI consumption in the amplifying ETP reactor.

8.The performance of the proposed system is evaluated by pollutant removal efficiency. The authors should provide TOC measurements to assess mineralization efficiency and conduct intermediate product analysis to elucidate degradation pathways. Without such data, it remains unclear whether it generates persistent or toxic intermediates.

Response 8:

We thank the reviewer for this important and constructive comment. We fully agree that pollutant removal efficiency alone is not sufficient to comprehensively evaluate the performance of the proposed system. In the original manuscript, although several transformation products were identified, we did not provide TOC data, the degradation pathway was not clearly established based on these products, and the possible formation of persistent or toxic intermediates was not discussed in sufficient depth. We appreciate this comment, which helped us improve both the completeness and the environmental relevance of the study.

Accordingly, we have now supplemented the manuscript with TOC measurements and further clarified the transformation pathway on the basis of the identified products. The TOC removal reached 81.2%, indicating substantial elimination of organic carbon from the aqueous phase during the reaction. At the same time, we would like to clarify that, for polymerization-mediated advanced oxidation systems, TOC should not be

interpreted simply as a direct measure of complete mineralization. In such systems, part of the dissolved organic carbon may be converted into oligomeric or polymeric products, including insoluble or separable carbonaceous species, rather than being fully oxidized to CO_2. Therefore, TOC abatement in our system indicates substantial removal of aqueous-phase organic carbon, but should not be directly interpreted as complete mineralization without considering the identified transformation products.

Based on the identified transformation products, the transformation of 4-CP in our system was dominated by a coupling pathway rather than a purely stepwise ring-opening route. The mechanistic link between ETP and oligomer formation can be described as follows: ETP first initiates one-electron oxidation of 4-CP, producing phenoxy/phenoxonium-type intermediates; these intermediates then undergo intermolecular C–C/C–O coupling at the catalyst interface; repeated coupling finally leads to chlorinated dimers, trimers, and higher oligomeric products (**Fig. R8**). Therefore, under the present reaction conditions, polymerization represented a major transformation route.

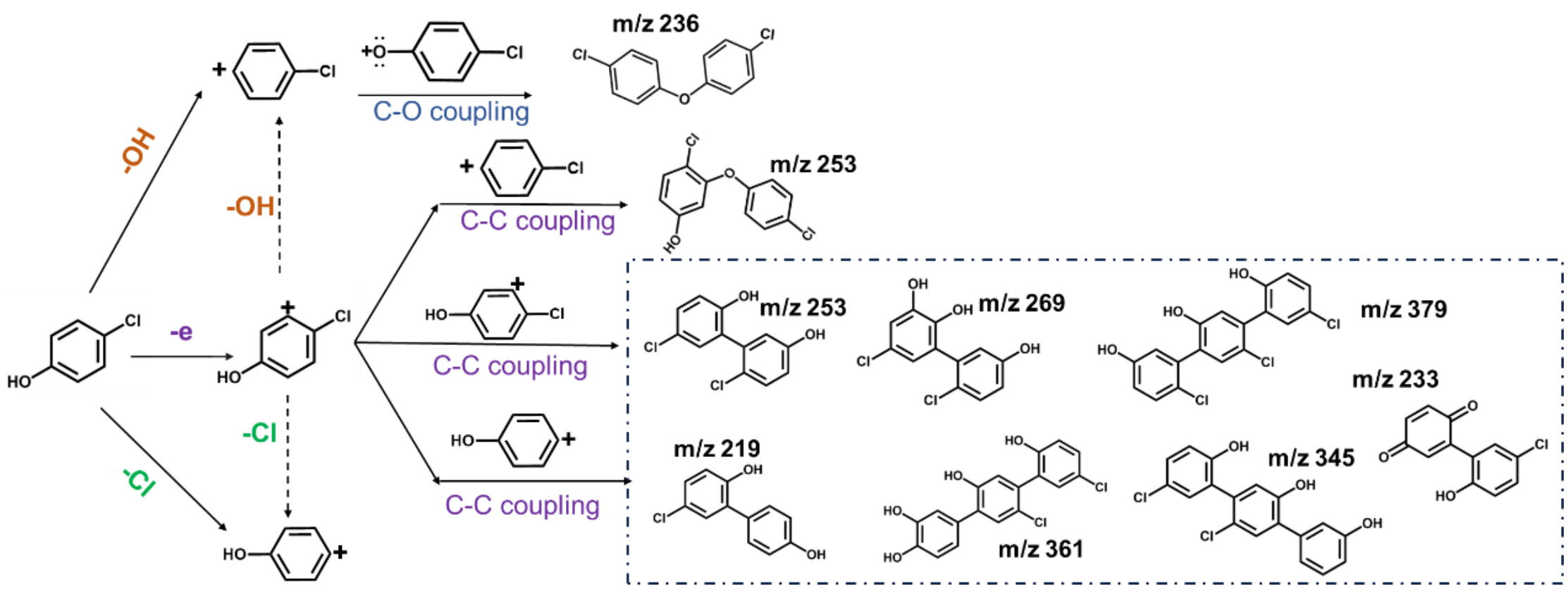

Fig. R8 Possible transformation pathways of 4-CP in F-FeSAC/PI.

We agree that these oligomeric products cannot be simply regarded as harmless, because they still retain aromatic structures and, in some cases, quinone-like oxidation motifs, which may imply potential intrinsic hazard. However, for polymerization-mediated transformation pathways, the environmental implication should not be judged solely by the intrinsic structure of the products, but also by their phase behavior and exposure form. Compared with small dissolved intermediates, oligomeric products are generally less soluble and are more likely to deposit on catalyst surfaces or be removed by solid-liquid separation. This interpretation is also supported by both recent literature and our experimental evidence. Recent high-impact studies on polymerization-

mediated electron-transfer oxidation have shown that phenolic pollutants can be selectively converted into insoluble polymeric products, which tend to accumulate on catalyst surfaces or be removed as recoverable solids rather than persist as dissolved intermediates[33, 34]. In particular, Wang et. al[35] recently reported that most 4-CP was transformed into insoluble polymers accumulated on the catalyst surface.

Consistent with this pattern, our results provide direct evidence for surface-associated organic deposition. The oligomeric products exhibited strong adsorption affinity toward the catalyst substrate, likely due to the combined effects of hydrophobic interactions, specific interfacial binding, and their low aqueous solubility. XPS analysis of the O 1s region showed a clear shift in surface oxygen species from O–H to C–O functionalities after reaction, indicating the accumulation of adsorbed carbonaceous matter on the catalyst surface **(Fig. 5b-c)**. Furthermore, TGA revealed an additional 8.3% weight loss for the used catalyst relative to pristine F-FeSAC **(Fig. 5e)**, which can be attributed to the thermal decomposition of deposited chlorinated oligomeric species[36]. These results collectively support that, compared with small dissolved intermediates, the oligomeric products formed in our system were more prone to surface accumulation and could be removed from the aqueous phase through solid-associated deposition rather than remaining as persistent dissolved byproducts.

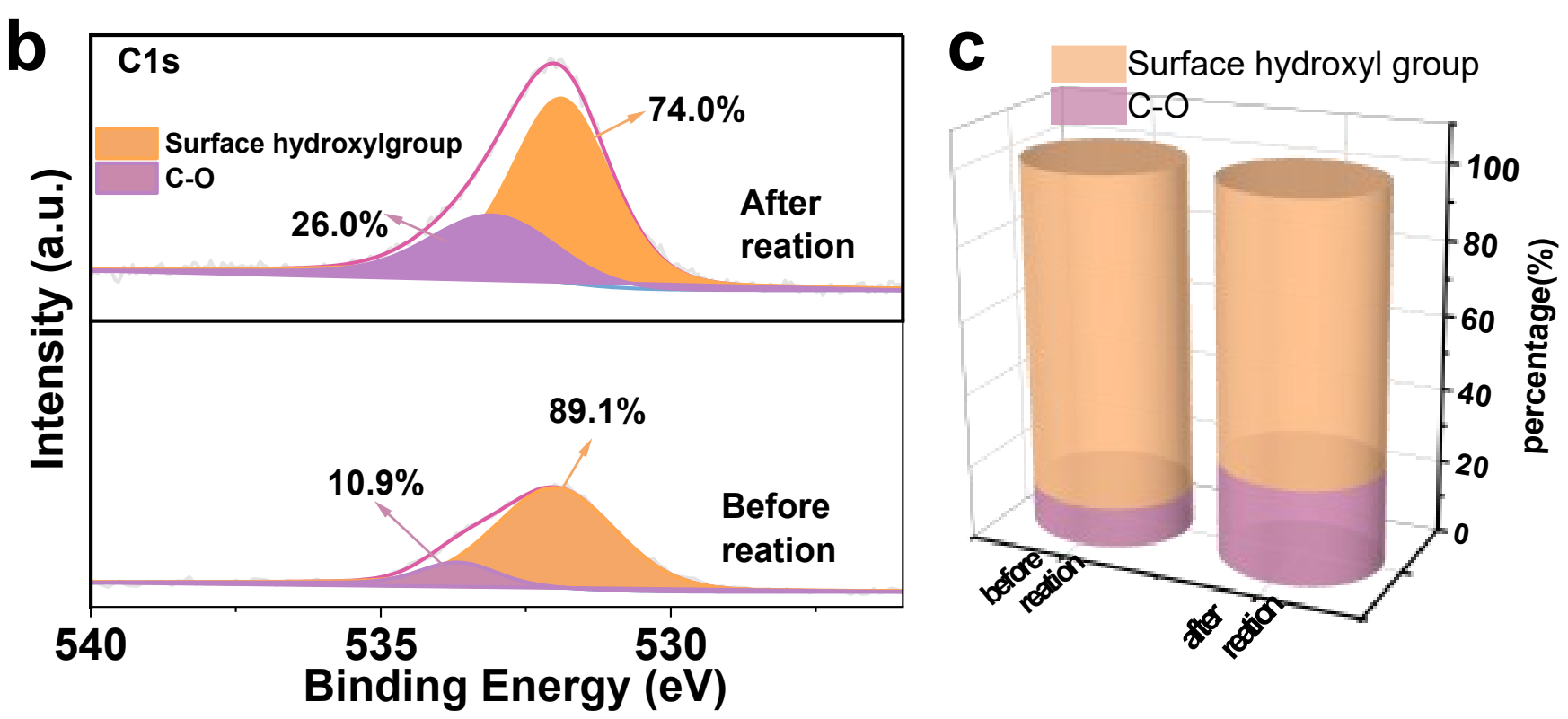

Fig. 5 (b) XPS C 1s spectra of F-FeSAC before and after reaction. (c) Deconvoluted components of the XPS C 1s spectra for F-FeSAC before and after reaction.

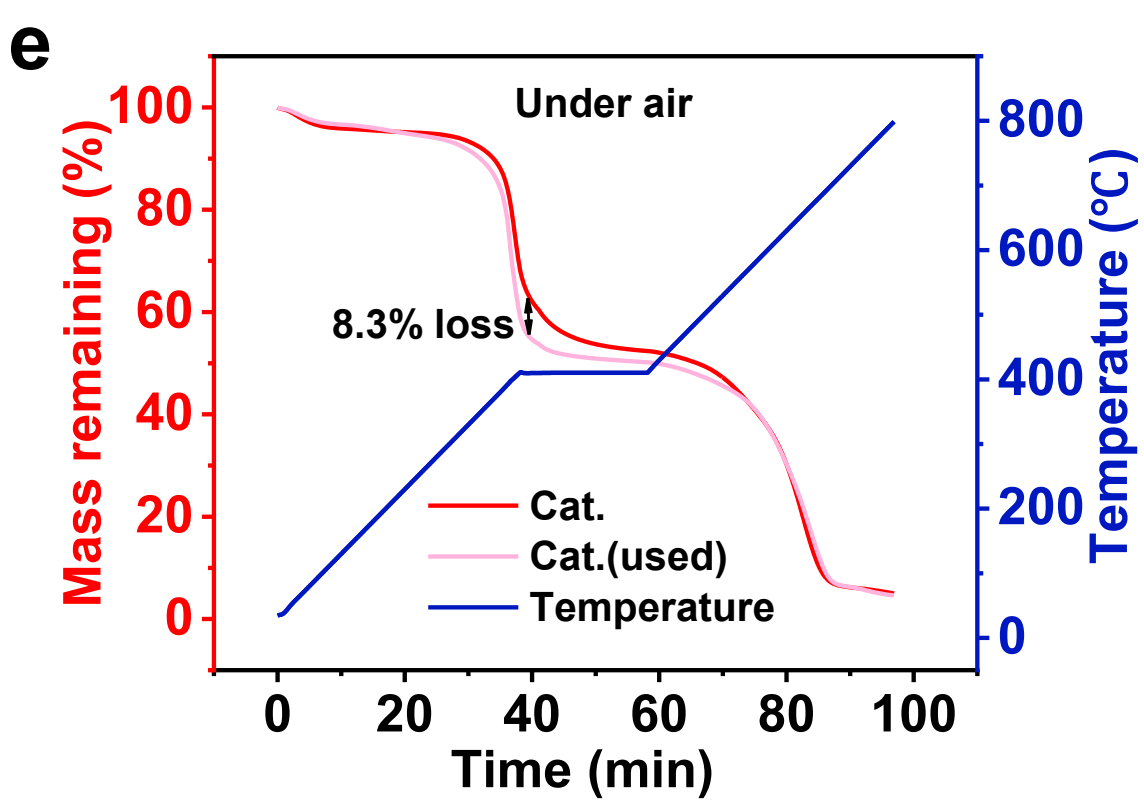

Fig. 5 (e) TGA curves of F-FeSAC before and after the reaction.

To further evaluate the environmental safety of the treated system, we additionally examined the possible formation of reactive iodine species and conducted ecotoxicological validation experiments. Because PI-based AOPs may generate environmentally concerning iodine intermediates, such as HOI, I^-, and I_3^-, which can further lead to toxic iodinated byproducts, we systematically investigated the iodine-speciation behavior in the F-FeSAC/PI system. The results showed that IO_4^- was predominantly converted to IO_3^- without the appearance of any reactive iodine intermediates **(Fig. 6a)**. Since HOI is known to react with phenolic compounds to produce iodinated phenolic byproducts such as 4-iodophenol and 2-iodophenol, we further analyzed the reaction solution by LC-HRMS. Neither 4-IP nor 2-IP was detected, confirming that HOI was not formed in the reaction system **(Fig. 6b)**. In addition, starch colorimetry showed no detectable I_2 or I_3^- **(Fig. 6c)**, further corroborating the absence of reduced iodine species. These results demonstrate that the F-FeSAC/PI process effectively suppresses the formation of environmentally risky iodine-derived byproducts.

More importantly, the ecotoxicological safety of the treated effluent was further assessed by zebrafish and microbial bioassays. After 120 h of exposure, untreated 4-CP caused severe mortality and behavioral abnormalities, whereas zebrafish exposed to the F-FeSAC/PI-treated solution maintained normal morphology and survival throughout the observation period **(Fig. 6d)**. In parallel, flow-cytometry analysis showed that after 40 min of treatment, the proportion of viable microbial cells increased from 56.2% to 79.3%, while the proportion of dead cells decreased from 7.17% to 3.16% **(Fig. 6e-f)**. These toxicity results provide direct biological evidence that the treatment process significantly reduced the ecotoxicological effects of the original pollutant solution.

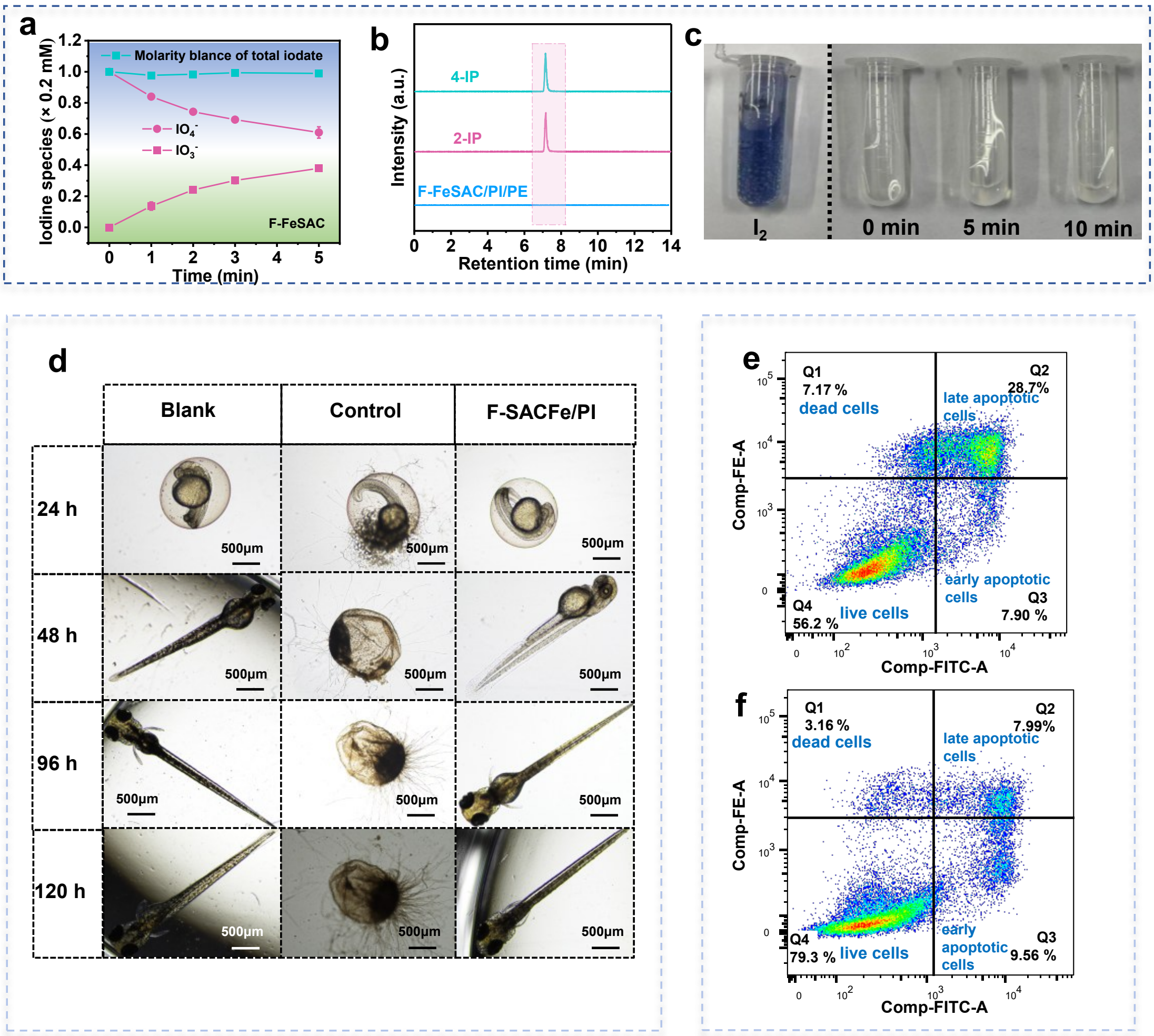

Fig. 6 | Analysis of generated iodine species and toxicity analysis. (a) Concentration changes of IO_4^- and IO_3^- in the F-FeSAC/PI/4-CP system. (b) Detection of HOI generated in the F-FeSAC/PI system using phenol as a trapping agent. (c) Detection of I_2 and I_3^- in the F-FeSAC/PI system. (d) Images of zebrafish embryos under different treatments: nutrient medium only (Blank), 4-CP solution (Control), and post-reaction 4-CP solution from the F-FeSAC/PI system. Flow cytometry analysis of *E. coli* cells during 4-CP degradation in F-FeSAC/PI system at different reaction time: (e) 0 min, (f) 40 min. The four sections in flow cytometry images represent as below: Q1 dead cells; Q2 late apoptotic cells; Q3 early apoptotic cells; Q4 live cells.

Taken together, the revised results show that the proposed system does not merely achieve high 4-CP removal efficiency, but also substantially removes aqueous-phase organic carbon, converts 4-CP mainly through coupling-mediated transformation pathways, suppresses the formation of hazardous reactive iodine species, and significantly reduces the biological toxicity of the treated effluent. Accordingly, we have revised the manuscript to present the mineralization behavior, transformation

pathway, and environmental implications of the system in a more complete manner.

Action 8: We have the added relevant statements in the revised manuscript and Supplementary Figs. 34-35 have been added in the supporting information.

*"Characterization of the accumulated surface species provided mechanistic insight. Liquid chromatography-mass spectrometry (LC-MS) analysis of the solution following desorption identified chlorinated oligomers as the main surface-adsorbed products formed during 4-CP oxidation (**Supplementary Figs. 34-35**). Their formation supports a polymerization-mediated electron-transfer pathway. Specifically, 4-CP is first oxidized through interfacial electron transfer to generate phenoxy- or phenoxonium-type intermediates. These intermediates are resonance-stabilized over the oxygen atom and the ortho/para positions of the aromatic ring, making them prone to intermolecular C–C and C–O coupling. Repeated coupling of these surface-generated intermediates then leads to chlorinated dimers, trimers, and higher oligomeric products. Owing to their low solubility and strong interfacial affinity, these oligomeric products preferentially accumulate on the catalyst surface, leading to progressive activity attenuation during cycling."*

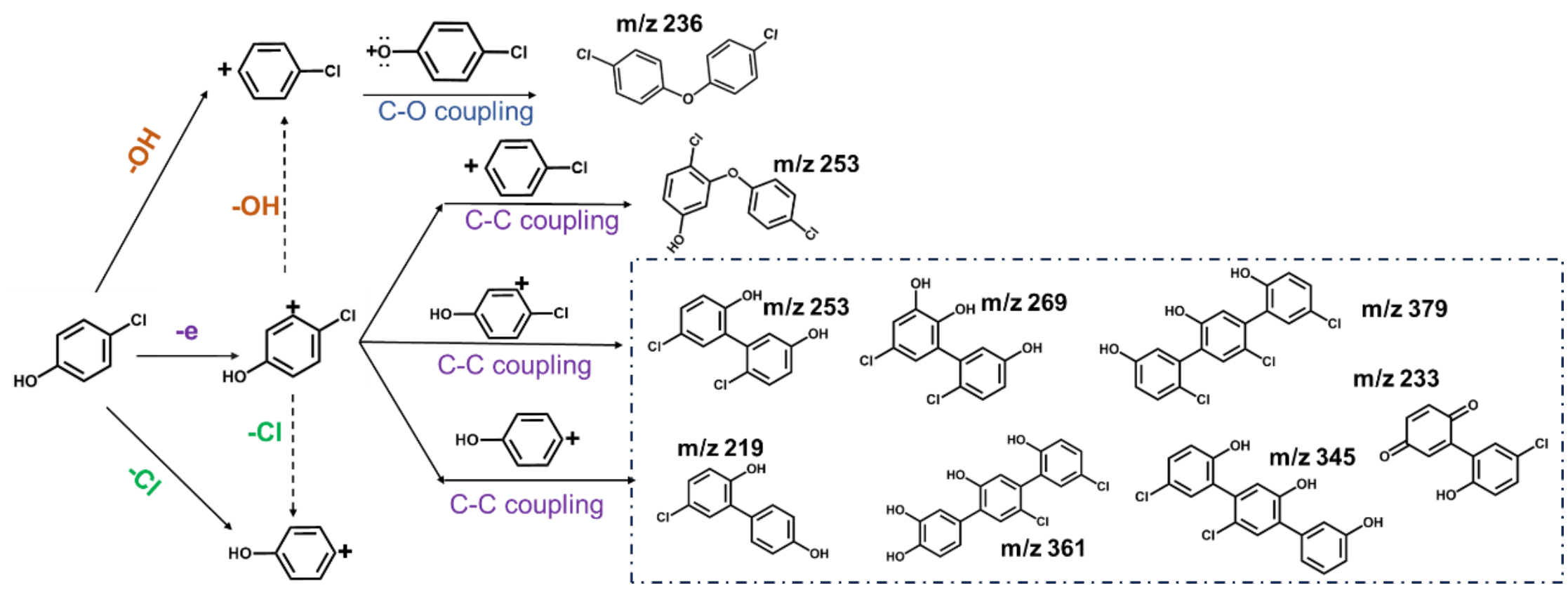

Supplementary Fig. 34 Possible transformation pathways of 4-CP in F-FeSAC/PI.

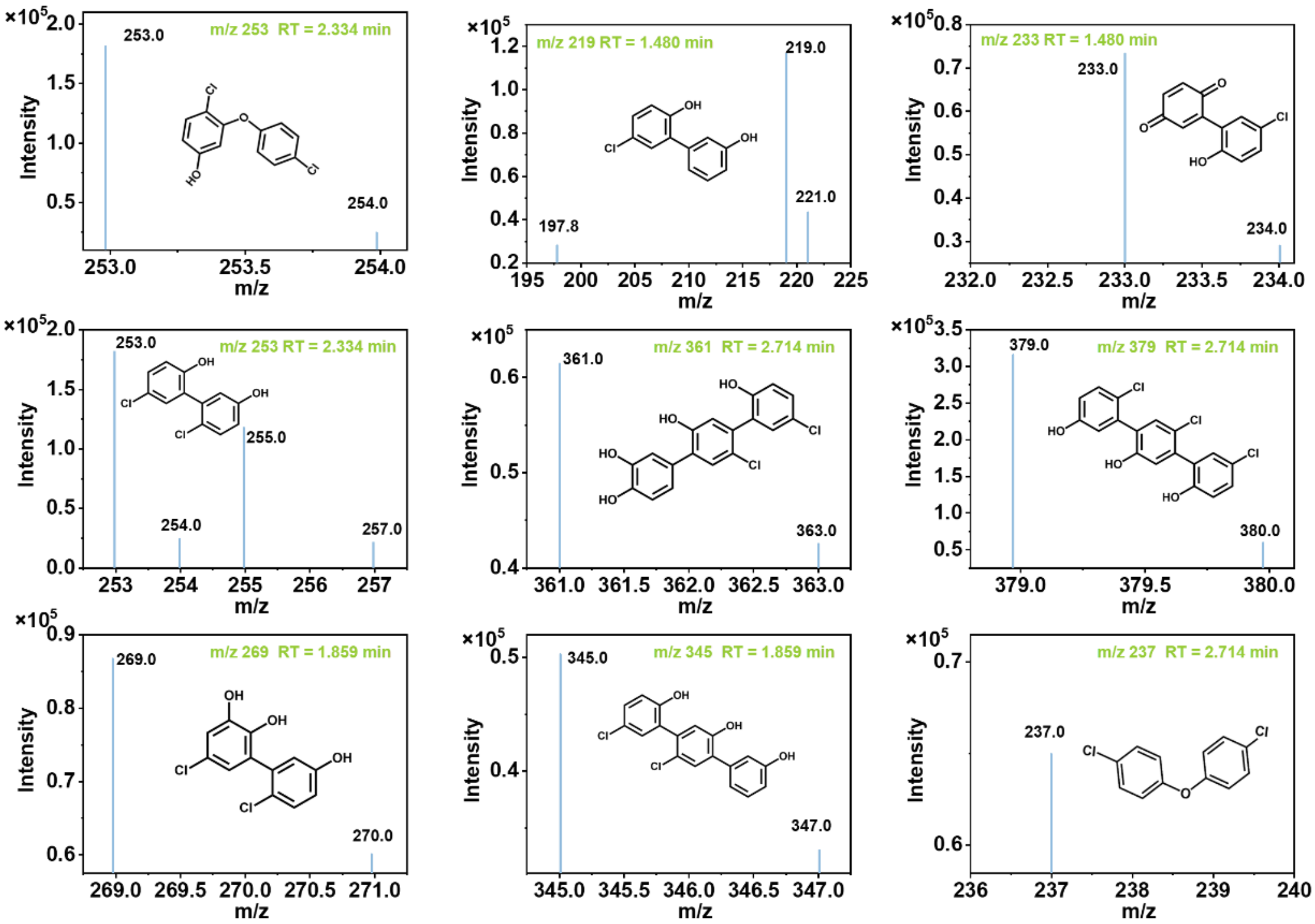

Supplementary Fig. 35 ESI mass spectra of 4-CP during reaction in F-FeSAC/PI system.

9.Given that periodate activation may generate multiple inorganic and organic iodine species, non-target screening of iodinated organic byproducts is essential, which is necessary to exclude secondary environmental risks. Moreover, a quantitative iodine mass balance should be provided to assess iodine recovery efficiency and long-term sustainability of the system.

Response 9:

We thank the reviewer for raising this important comment. We agree that possible iodinated organic byproducts and iodine recovery should be carefully evaluated when PI is used as the oxidant, because these species may introduce secondary environmental risks.

Screening of iodinated organic byproducts

To address this concern, we performed additional high-resolution LC-MS analysis of the treated solution. HRMS-based non-target and suspect-screening workflows are widely used for identifying transformation products and halogenated disinfection byproducts in aquatic systems, because they allow accurate-mass extraction, molecular-formula assignment, isotopic evaluation, and MS/MS-based structural assessment[37]. Similar LC-HRMS suspect-screening strategies have also been applied to iodinated

compounds and their transformation products in surface-water systems, supporting the suitability of this approach for iodine-containing organic species[38, 39].

Initial non-target screening of the full-scan HRMS data did not identify reliable iodinated organic features. This may be related to the low molecular weights, weak ionization responses, and background interference of plausible iodinated phenolic intermediates. Therefore, to increase the analytical coverage and reduce false-positive assignments, **we further conducted suspect screening for more than 200 theoretically possible iodinated organic compounds.** These candidates were constructed based on the detected transformation products, plausible iodination products of 4-CP and its intermediates, and structurally related iodine-containing compounds expected in oxidative iodine chemistry.

For each candidate, the molecular formula and theoretical exact mass were calculated, and extracted-ion chromatograms were generated within a ±5 ppm mass tolerance. Candidate signals were further evaluated according to chromatographic peak shape, signal-to-noise ratio, formula plausibility, isotopic simulation, and MS/MS fragmentation information when available. None of the more than 200 screened candidates met the assignment criteria for iodinated organic byproducts. Representative EICs and mass spectra are provided in **Figs. R9-10**, and the screening results are summarized in **Table R1.** These results indicate that no credible iodinated organic byproducts were detected under the present analytical conditions.

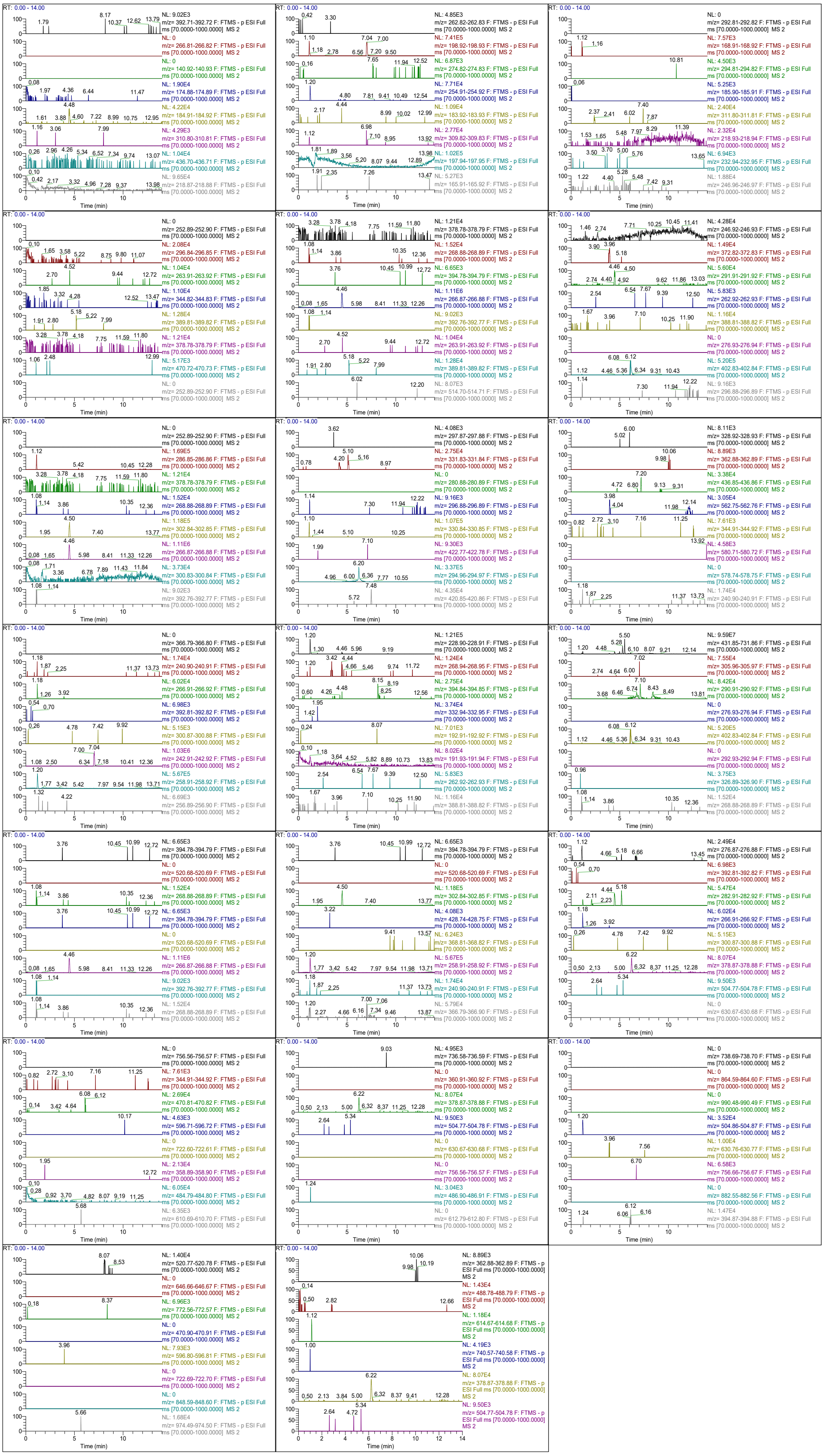

Fig. R9 Representative EICs of iodinated byproduct candidates

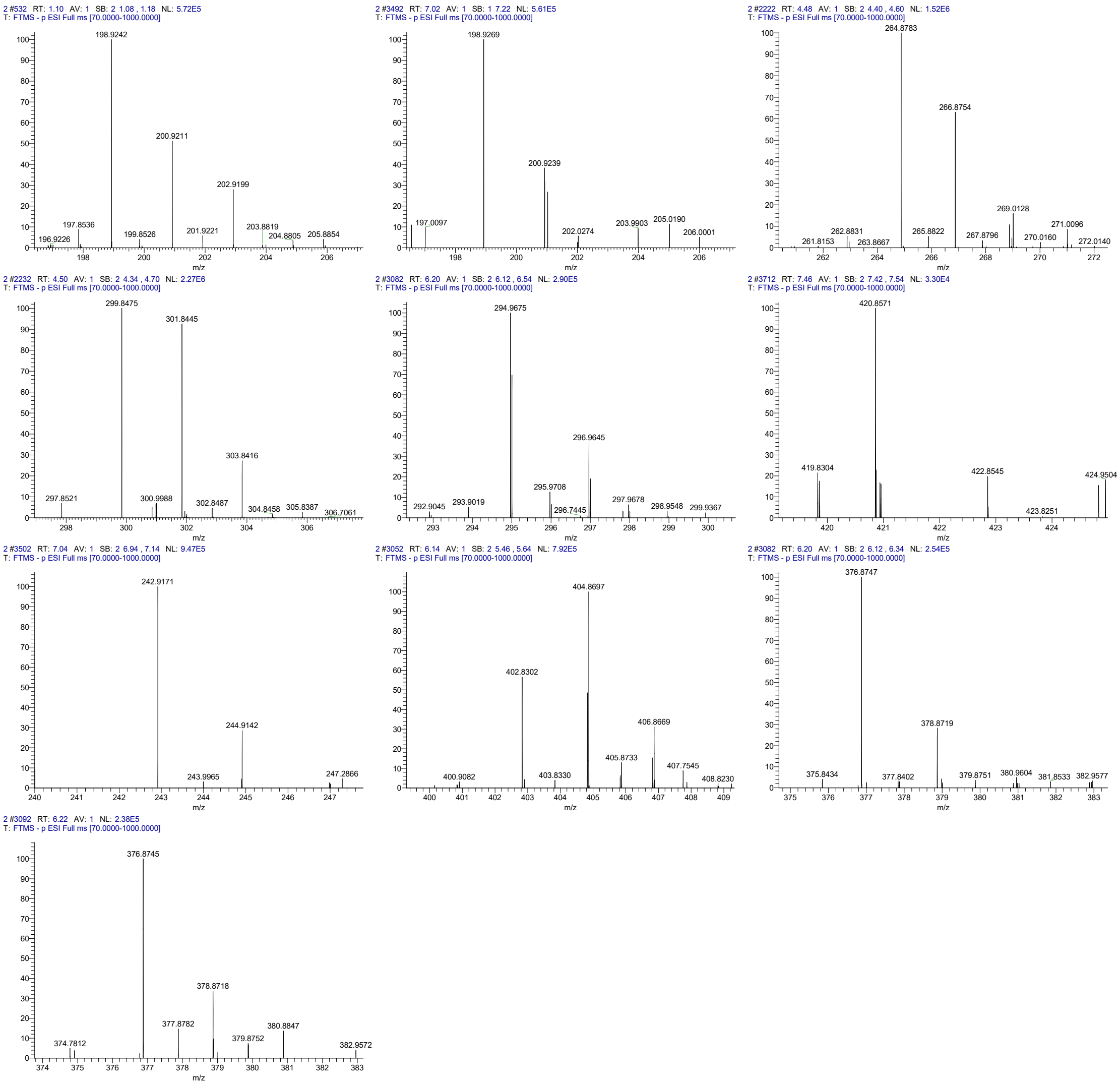

Fig. R10 Mass spectra of suspected iodinated byproduct candidates.

Table R1. HRMS-based suspect screening of theoretically possible iodinated organic byproducts in the treated solution.

No	Compound name	Molecular formula	Exact mass [M-H]- (individually verified)	Screening result
**1**	Iodoform	CHI_3	392.7140	ND
**2**	Diiodomethane	CH_2I_2	266.8173	ND
**3**	Iodomethane	CH_3I	140.9207	ND
**4**	Chloroiodomethane	CH_2ClI	174.8817	ND
**5**	Iodoacetic acid	$C_2H_3IO_2$	184.9105	ND
**6**	Diiodoacetic acid	$C_2H_2I_2O_2$	310.8066	ND
**7**	Triiodoacetic acid	$C_2HI_3O_2$	436.7038	ND

No	Compound name	Molecular formula	Exact mass [M-H]- (individually verified)	Screening result
**8**	Chloroiodoacetic acid	$C_2H_2ClIO_2$	218.8715	ND
**9**	Bromoiodoacetic acid	$C_2H_2BrIO_2$	262.8210	ND
**10**	3-Iodopropionic acid	$C_3H_5IO_2$	198.9261	ND (MIC response observed; excluded based on isotope-pattern distribution)
**11**	3-Bromo-3-iodoacrylic acid	$C_3H_2BrIO_2$	274.8210	ND
**12**	2-Iodo-3-methylbutenedioic acid	$C_5H_5IO_4$	254.9160	ND
**13**	Iodoacetamide	C_2H_4INO	183.9265	ND
**14**	Diiodoacetamide	$C_2H_3I_2NO$	309.8231	ND
**15**	Iodopropionamide	C_3H_6INO	197.9421	ND
**16**	Iodoacetonitrile	C_2H_2IN	165.9154	ND
**17**	Diiodoacetonitrile	C_2HI_2N	292.8198	ND
**18**	Iodoacetaldehyde	C_2H_3IO	168.9156	ND
**19**	Diiodoacetaldehyde	$C_2H_2I_2O$	294.8122	ND
**20**	Iodonitromethane	CH_2INO_2	185.9057	ND
**21**	Diiodonitromethane	CHI_2NO_2	311.8024	ND
**22**	2-Iodophenol	C_6H_5IO	218.9313	
**23**	3-Iodophenol	C_6H_5IO	218.9313	ND
**24**	4-Iodophenol	C_6H_5IO	218.9313	
**25**	4-Iodo-2-methylphenol	C_7H_7IO	232.9469	ND
**26**	2-Iodo-4-methylphenol	C_7H_7IO	232.9469	
**27**	4-Iodo-2,6-dimethylphenol	C_8H_9IO	246.9625	ND
**28**	2-Iodo-4-chlorophenol	C_6H_4ClIO	252.8923	
**29**	2-Iodo-6-chlorophenol	C_6H_4ClIO	252.8923	ND
**30**	4-Iodo-2-chlorophenol	C_6H_4ClIO	252.8923	
**31**	2-Iodo-4-bromophenol	C_6H_4BrIO	296.8417	ND
**32**	4-Iodo-2-nitrophenol	$C_6H_4INO_3$	263.9163	ND
**33**	2-Iodo-4-nitrophenol	$C_6H_4INO_3$	263.9163	
**34**	2,4-Diiodophenol	$C_6H_4I_2O$	344.8279	ND

No	Compound name	Molecular formula	Exact mass [M-H]- (individually verified)	Screening result
**35**	2,6-Diiodophenol	$C_6H_4I_2O$	344.8279	
**36**	2,5-Diiodophenol	$C_6H_4I_2O$	344.8279	
**37**	2,6-Diiodo-4-nitrophenol	$C_6H_3I_2NO_3$	389.8130	ND
**38**	2,4-Diiodo-6-nitrophenol	$C_6H_3I_2NO_3$	389.8130	
**39**	2,6-Diiodo-4-chlorophenol	$C_6H_3ClI_2O$	378.7889	ND
**40**	2,4-Diiodo-6-chlorophenol	$C_6H_3ClI_2O$	378.7889	
**41**	2,4,6-Triiodophenol	$C_6H_3I_3O$	470.7245	ND
**42**	2,3,5-Triiodophenol	$C_6H_3I_3O$	470.7245	
**43**	4-Chloro-2-iodophenol	C_6H_4ClIO	252.8923	ND
**44**	4-Chloro-2,6-diiodophenol	$C_6H_3ClI_2O$	378.7889	ND
**45**	2-Chloro-4-iodophenol	C_6H_4ClIO	252.8923	ND
**46**	2-Chloro-4,6-diiodophenol	$C_6H_3ClI_2O$	378.7889	ND
**47**	4-Chloro-6-iodobenzene-1,2-diol	$C_6H_4ClIO_2$	268.8872	
**48**	4-Chloro-6-iodobenzene-1,3-diol	$C_6H_4ClIO_2$	268.8872	ND
**49**	2-Iodo-5-chlorohydroquinone	$C_6H_4ClIO_2$	268.8872	
**50**	2,6-Diiodo-5-chlorohydroquinone	$C_6H_3ClI_2O_2$	394.7838	ND
**51**	2-Iodo-5-chloro-p-benzoquinone	$C_6H_2ClIO_2$	266.8715	ND
**52**	2,6-Diiodo-5-chloro-p-benzoquinone	$C_6HClI_2O_2$	392.7682	ND
**53**	2-Iodo-4-nitrophenol	$C_6H_4INO_3$	263.9163	
**54**	4-Iodo-2-nitrophenol	$C_6H_4INO_3$	263.9163	ND
**55**	2-Iodo-5-nitrophenol	$C_6H_4INO_3$	263.9163	
**56**	2,4-Diiodo-6-nitrophenol	$C_6H_3I_2NO_3$	389.8130	ND
**57**	2,6-Diiodo-4-nitrophenol	$C_6H_3I_2NO_3$	389.8130	
**58**	Triiodo nitrophenol	$C_6HI_3NO_3$	514.7018	ND
**59**	3-Iodo-4-hydroxybenzaldehyde	$C_7H_5IO_2$	246.9262	
**60**	2-Iodo-4-hydroxybenzaldehyde	$C_7H_5IO_2$	246.9262	ND
**61**	4-Iodo-3-hydroxybenzaldehyde	$C_7H_5IO_2$	246.9262	
**62**	3,5-Diiodo-4-hydroxybenzaldehyde	$C_7H_4I_2O_2$	372.8228	ND
**63**	2,6-Diiodo-4-hydroxybenzaldehyde	$C_7H_4I_2O_2$	372.8228	
**64**	3-Iodo-4-hydroxy-5-	$C_7H_4INO_4$	291.9112	ND

No	Compound name	Molecular formula	Exact mass [M-H]- (individually verified)	Screening result
	nitrobenzaldehyde			
**65**	3-Iodo-4-hydroxybenzoic acid	$C_7H_5IO_3$	262.9211	ND
**66**	2-Iodo-4-hydroxybenzoic acid	$C_7H_5IO_3$	262.9211	
**67**	3,5-Diiodo-4-hydroxybenzoic acid	$C_7H_4I_2O_3$	388.8177	ND
**68**	2,6-Diiodo-4-hydroxybenzoic acid	$C_7H_4I_2O_3$	388.8177	ND
**69**	3-Iodo-4-hydroxy-5-methylbenzoic acid	$C_8H_7IO_3$	276.9367	ND
**70**	3,5-Diiodo-4-hydroxy-methylbenzoic acid	$C_8H_6I_2O_3$	402.8334	ND
**71**	2-Iodo-5-chloro-4-hydroxybenzoic acid	$C_7H_4ClIO_3$	296.8821	ND
**72**	Monochloro-monoiodophenol (all isomers)	C_6H_4ClIO	252.8923	ND
**73**	Dichloro-monoiodophenol	$C_6H_3Cl_2IO$	286.8533	ND
**74**	Monochloro-diiodophenol	$C_6H_3ClI_2O$	378.7889	ND
**75**	Monochloro-monoiodohydroquinone	$C_6H_4ClIO_2$	268.8872	ND
**76**	Dichloro-monoiodohydroquinone	$C_6H_3Cl_2IO_2$	302.8482	ND (MIC response observed; excluded based on isotope-pattern distribution)
**77**	Monochloro-monoiodo-p-benzoquinone	$C_6H_2ClIO_2$	266.8715	ND (MIC response observed; excluded based on isotope-pattern distribution)
**78**	Dichloro-monoiodo-p-benzoquinone	$C_6HCl_2IO_2$	300.8326	ND
**79**	Monochloro-diiodo-p-benzoquinone	$C_6HClI_2O_2$	392.7682	ND
**80**	Monochloro-monoiodonitrophenol	$C_6H_3ClINO_3$	297.8773	ND
**81**	Dichloro-monoiodonitrophenol	$C_6H_2Cl_2INO_3$	331.8384	ND

No	Compound name	Molecular formula	Exact mass [M-H]- (individually verified)	Screening result
**82**	Monochloro-monoiodohydroxybenzaldehyde	$C_7H_4ClIO_2$	280.8872	ND
**83**	Monochloro-monoiodohydroxybenzoic acid	$C_7H_4ClIO_3$	296.8821	ND
**84**	Dichloro-iodohydroxybenzoic acid	$C_7H_3Cl_2IO_3$	330.8431	ND
**85**	Monochloro-diiodohydroxybenzoic acid	$C_7H_3ClI_2O_3$	422.7787	ND
**86**	2-Iodo-[1,1'-biphenyl]-2-ol	$C_{12}H_9IO$	294.9625	ND (MIC response observed; excluded based on isotope-pattern distribution)
**87**	4-Iodo-[1,1'-biphenyl]-4-ol	$C_{12}H_9IO$	294.9625	
**88**	Diiodohydroxybiphenyl	$C_{12}H_8I_2O$	420.8592	ND (MIC response observed; excluded based on isotope-pattern distribution)
**89**	Chloroiodohydroxybiphenyl	$C_{12}H_8ClIO$	328.9236	ND
**90**	Dichloroiodohydroxybiphenyl	$C_{12}H_7Cl_2IO$	362.8846	ND
**91**	Diiodo biphenol	$C_{12}H_8I_2O_2$	436.8541	ND
**92**	Triiodo biphenol	$C_{12}H_7I_3O_2$	562.7507	ND
**93**	Chloroiodobiphenol	$C_{12}H_8ClIO_2$	344.9185	ND
**94**	Polychloro-polyiododiphenyl ether	$C_{12}H_{(7-x-y)}Cl_xI_yO_2$	various isomers	ND
**95**	2,4,6-Triiodo-4-chlorohydroxybiphenyl	$C_{12}H_6ClI_3O$	580.7169	ND
**96**	Triiodohydroxydiphenyl ether	$C_{12}H_7I_3O_3$	578.7457	ND
**97**	Iodomaleic acid	$C_4H_3IO_4$	240.9003	ND
**98**	Diiodomaleic acid	$C_4H_2I_2O_4$	366.7970	ND
**99**	Iodofumaric acid	$C_4H_3IO_4$	240.9003	ND
**100**	Iodomuconic acid	$C_6H_5IO_4$	266.9160	ND
**101**	Diiodomuconic acid	$C_6H_4I_2O_4$	392.8126	ND
**102**	Chloroiodomuconic acid	$C_6H_4ClIO_4$	300.8770	ND

No	Compound name	Molecular formula	Exact mass [M-H]- (individually verified)	Screening result
**103**	Iodosuccinic acid	$C_4H_5IO_4$	242.9160	ND (MIC response observed; excluded based on isotope-pattern distribution)
**104**	Iodomalic acid	$C_4H_5IO_5$	258.9109	ND
**105**	Iodooxaloacetic acid	$C_4H_3IO_5$	256.8952	ND
**106**	Iodomalonic acid	$C_3H_3IO_4$	228.9003	ND
**107**	Iodinated naphthols	$C_{10}H_7IO$	268.9469	ND
**108**	Diiodonaphthol	$C_{10}H_6I_2O$	394.8435	ND
**109**	Iodoanthraquinone	$C_{14}H_7IO_2$	332.9418	ND
**110**	Iodinated furans	C_4H_3IO	192.9156	ND
**111**	Iodinated pyrroles	C_4H_4IN	191.9316	ND
**112**	(E)-3-(5-Iodofuran-2-yl)acrylic acid	$C_7H_5IO_3$	262.9211	ND
**113**	3,5-Diiodosalicylic acid	$C_7H_4I_2O_3$	388.8177	ND
**114**	3,5-Diiodo-L-tyrosine	$C_9H_9I_2NO_3$	431.8599	ND
**115**	3-Iodo-L-tyrosine	$C_9H_{10}INO_3$	305.9633	ND
**116**	3-Iodophthalic acid	$C_8H_5IO_4$	290.9160	ND
**117**	3-Iodo-4-hydroxyphenylacetic acid	$C_8H_7IO_3$	276.9367	ND
**118**	3,5-Diiodo-4-hydroxyphenylacetic acid	$C_8H_6I_2O_3$	402.8334	ND (MIC response observed; excluded based on isotope-pattern distribution)
**119**	methyl iododihydroxybenzoate	$C_8H_7IO_4$	292.9316	ND
**120**	methyl chloro-iododihydroxybenzoate	$C_8H_6ClIO_4$	326.8927	ND
**121**	3-Iodo-4-chlorocatechol	$C_6H_4ClIO_2$	268.8872	
**122**	5-Iodo-4-chlorocatechol	$C_6H_4ClIO_2$	268.8872	ND
**123**	6-Iodo-4-chlorocatechol	$C_6H_4ClIO_2$	268.8872	

No	Compound name	Molecular formula	Exact mass [M-H]- (individually verified)	Screening result
**124**	3,5-Diiodo-4-chlorocatechol	$C_6H_3ClI_2O_2$	394.7838	
**125**	3,6-Diiodo-4-chlorocatechol	$C_6H_3ClI_2O_2$	394.7838	ND
**126**	5,6-Diiodo-4-chlorocatechol	$C_6H_3ClI_2O_2$	394.7838	
**127**	Triiodo-4-chlorocatechol	$C_6H_2ClI_3O_2$	520.6805	ND
**128**	2-Iodo-4-chlororesorcinol	$C_6H_4ClIO_2$	268.8872	
**129**	5-Iodo-4-chlororesorcinol	$C_6H_4ClIO_2$	268.8872	ND
**130**	6-Iodo-4-chlororesorcinol	$C_6H_4ClIO_2$	268.8872	
**131**	2,5-Diiodo-4-chlororesorcinol	$C_6H_3ClI_2O_2$	394.7838	
**132**	2,6-Diiodo-4-chlororesorcinol	$C_6H_3ClI_2O_2$	394.7838	ND
**133**	5,6-Diiodo-4-chlororesorcinol	$C_6H_3ClI_2O_2$	394.7838	
**134**	Triiodo-4-chlororesorcinol	$C_6H_2ClI_3O_2$	520.6805	ND
**135**	2-Iodo-5-chloro-p-benzoquinone	$C_6H_2ClIO_2$	266.8715	ND
**136**	2,6-Diiodo-5-chloro-p-benzoquinone	$C_6HClI_2O_2$	392.7682	ND
**137**	2,3-Diiodo-5-chloro-p-benzoquinone	$C_6HClI_2O_2$	392.7682	
**138**	Triiodo-5-chloro-p-benzoquinone	$C_6ClI_3O_2$	/	ND
**139**	3-Iodo-2-chlorohydroquinone	$C_6H_4ClIO_2$	268.8872	
**140**	5-Iodo-2-chlorohydroquinone	$C_6H_4ClIO_2$	268.8872	ND
**141**	6-Iodo-2-chlorohydroquinone	$C_6H_4ClIO_2$	268.8872	
**142**	3,5-Diiodo-2-chlorohydroquinone	$C_6H_3ClI_2O_2$	394.7838	
**143**	3,6-Diiodo-2-chlorohydroquinone	$C_6H_3ClI_2O_2$	394.7838	ND
**144**	5,6-Diiodo-2-chlorohydroquinone	$C_6H_3ClI_2O_2$	394.7838	
**145**	Triiodo-2-chlorohydroquinone	$C_6H_2ClI_3O_2$	520.6805	ND
**146**	3-Iodo-2,5-dichlorohydroquinone	$C_6H_3Cl_2IO_2$	302.8482	ND
**147**	3,6-Diiodo-2,5-dichlorohydroquinone	$C_6H_2Cl_2I_2O_2$	428.7448	ND
**148**	2,3-Diiodosuccinic acid	$C_4H_4I_2O_4$	368.8126	ND
**149**	2-Iodo-3-hydroxysuccinic acid	$C_4H_5IO_5$	258.9109	ND
**150**	Iodomaleic acid	$C_4H_3IO_4$	240.9003	ND
**151**	Iodofumaric acid	$C_4H_3IO_4$	240.9003	
**152**	Diiodomaleic acid	$C_4H_2I_2O_4$	366.7970	ND
**153**	Diiodofumaric acid	$C_4H_2I_2O_4$	366.7970	

No	Compound name	Molecular formula	Exact mass [M-H]- (individually verified)	Screening result
**154**	Chloroiodosuccinic acid	$C_4H_4ClIO_4$	276.8770	ND
**155**	2,3-Diiodomuconic acid	$C_6H_4I_2O_4$	392.8126	ND
**156**	2,5-Diiodomuconic acid	$C_6H_4I_2O_4$	392.8126	
**157**	2-Iodo-3-hydroxymuconic acid	$C_6H_5IO_5$	282.9109	ND
**158**	2-Iodo-5-hydroxymuconic acid	$C_6H_5IO_5$	282.9109	
**159**	Monoiodomuconic acid (all isomers)	$C_6H_5IO_4$	266.9160	ND (MIC response observed; excluded based on isotope-pattern distribution)
**160**	Chloroiodomuconic acid	$C_6H_4ClIO_4$	300.8770	ND
**161**	Hydroxy-dichloro-monoiododiphenyl ether	$C_{12}H_7Cl_2IO_2$	378.8795	ND (MIC response observed; excluded based on isotope-pattern distribution)
**162**	Hydroxy-dichloro-diiododiphenyl ether	$C_{12}H_6Cl_2I_2O_2$	504.7761	ND
**163**	Hydroxy-dichloro-triiododiphenyl ether	$C_{12}H_5Cl_2I_3O_2$	630.6728	ND
**164**	Hydroxy-dichloro-tetraiododiphenyl ether	$C_{12}H_4Cl_2I_4O_2$	756.5694	ND
**165**	Chloro-dihydroxy-monoiodobiphenyl	$C_{12}H_8ClIO_2$	344.9185	ND
**166**	Chloro-dihydroxy-diiodobiphenyl	$C_{12}H_7ClI_2O_2$	470.8151	ND
**167**	Chloro-dihydroxy-triiodobiphenyl	$C_{12}H_6ClI_3O_2$	596.7118	ND
**168**	Chloro-dihydroxy-tetraiodobiphenyl	$C_{12}H_5ClI_4O_2$	722.6084	ND
**169**	Chloro-hydroxy-monoiodobiphenylquinone	$C_{12}H_6ClIO_3$	358.8977	ND
**170**	Chloro-hydroxy-diiodobiphenylquinone	$C_{12}H_5ClI_2O_3$	484.7944	ND
**171**	Chloro-hydroxy-triiodobiphenylquinone	$C_{12}H_4ClI_3O_3$	610.6910	ND

No	Compound name	Molecular formula	Exact mass [M-H]- (individually verified)	Screening result
**172**	Chloro-hydroxy-tetraiodobiphenylquinone	$C_{12}H_3ClI_4O_3$	736.5877	ND
**173**	Chloro-hydroxy-monoiodobiphenylhydroquinone	$C_{12}H_8ClIO_3$	360.9134	ND
**174**	Dichloro-dihydroxy-monoiodobiphenyl	$C_{12}H_7Cl_2IO_2$	378.8795	ND (MIC response observed; excluded based on isotope-pattern distribution)
**175**	Dichloro-dihydroxy-diiodobiphenyl	$C_{12}H_6Cl_2I_2O_2$	504.7761	ND
**176**	Dichloro-dihydroxy-triiodobiphenyl	$C_{12}H_5Cl_2I_3O_2$	630.6728	ND
**177**	Dichloro-dihydroxy-tetraiodobiphenyl	$C_{12}H_4Cl_2I_4O_2$	756.5694	ND
**178**	Dichloro-tetrahydroxy-monoiodoterphenyl	$C_{18}H_{11}Cl_2IO_4$	486.9006	ND
**179**	Dichloro-tetrahydroxy-diiodoterphenyl	$C_{18}H_{10}Cl_2I_2O_4$	612.7973	ND
**180**	Dichloro-tetrahydroxy-triiodoterphenyl	$C_{18}H_9Cl_2I_3O_4$	738.6939	ND
**181**	Dichloro-tetrahydroxy-tetraiodoterphenyl	$C_{18}H_8Cl_2I_4O_4$	864.5906	ND
**182**	Dichloro-tetrahydroxy-pentaiodoterphenyl	$C_{18}H_7Cl_2I_5O_4$	990.4872	ND
**183**	Trichloro-trihydroxy-monoiodoterphenyl	$C_{18}H_{10}Cl_3IO_3$	504.8667	ND
**184**	Trichloro-trihydroxy-diiodoterphenyl	$C_{18}H_9Cl_3I_2O_3$	630.7634	ND
**185**	Trichloro-trihydroxy-triiodoterphenyl	$C_{18}H_8Cl_3I_3O_3$	756.6600	ND
**186**	Trichloro-trihydroxy-tetraiodoterphenyl	$C_{18}H_7Cl_3I_4O_3$	882.5567	ND
**187**	Dichloro-trihydroxy-monoiodobiphenyl	$C_{12}H_7Cl_2IO_3$	394.8744	ND
**188**	Dichloro-trihydroxy-diiodobiphenyl	$C_{12}H_6Cl_2I_2O_3$	520.7711	ND
**189**	Dichloro-trihydroxy-	$C_{12}H_5Cl_2I_3O_3$	646.6677	ND

No	Compound name	Molecular formula	Exact mass [M-H]- (individually verified)	Screening result
	triiodobiphenyl			
**190**	Dichloro-trihydroxy-tetraiodobiphenyl	$C_{12}H_4Cl_2I_4O_3$	772.5643	ND
**191**	Dichloro-trihydroxy-monoiodoterphenyl	$C_{18}H_{11}Cl_2IO_3$	470.9057	ND
**192**	Dichloro-trihydroxy-diiodoterphenyl	$C_{18}H_{10}Cl_2I_2O_3$	596.8024	ND
**193**	Dichloro-trihydroxy-triiodoterphenyl	$C_{18}H_9Cl_2I_3O_3$	722.6990	ND
**194**	Dichloro-trihydroxy-tetraiodoterphenyl	$C_{18}H_8Cl_2I_4O_3$	848.5957	ND
**195**	Dichloro-trihydroxy-pentaiodoterphenyl	$C_{18}H_7Cl_2I_5O_3$	974.4923	ND
**196**	Dichloro-monoiododiphenyl ether	$C_{12}H_7Cl_2IO$	362.8846	ND
**197**	Dichloro-diiododiphenyl ether	$C_{12}H_6Cl_2I_2O$	488.7812	ND
**198**	Dichloro-triiododiphenyl ether	$C_{12}H_5Cl_2I_3O$	614.6779	ND
**199**	Dichloro-tetraiododiphenyl ether	$C_{12}H_4Cl_2I_4O$	740.5745	ND
**200**	Hydroxylated dichloro-monoiododiphenyl ether	$C_{12}H_7Cl_2IO_2$	378.8795	ND
**201**	Hydroxylated dichloro-diiododiphenyl ether	$C_{12}H_6Cl_2I_2O_2$	504.7761	ND

Iodide and reactive iodine analysis

We further examined possible inorganic and reactive iodine species. I^- was quantified by ion chromatography and was not detected under the tested conditions, excluding iodide as a relevant iodine species in the present system (**Fig. R11**). In addition, starch colorimetry showed no detectable response attributable to I_2/I_3^- accumulation during the F-FeSAC/PI process (**Fig. 6c**). These results do not support significant accumulation of reactive iodine intermediates.

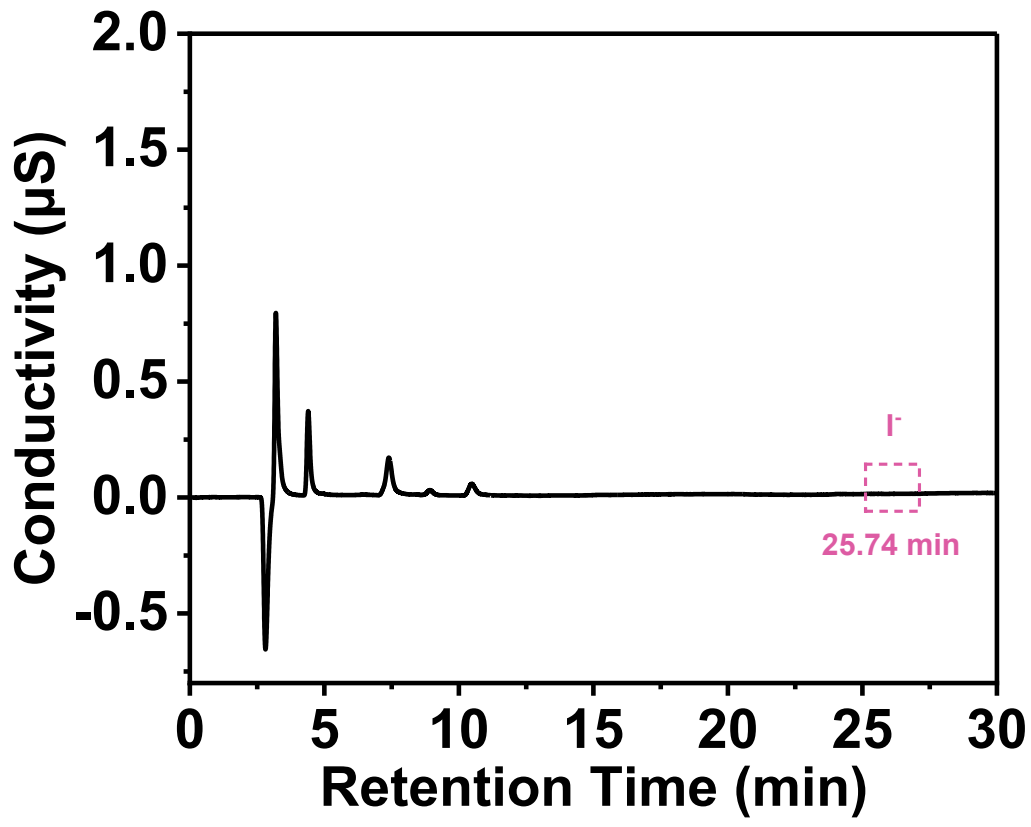

Fig. R11 IC chromatogram of iodide detection.

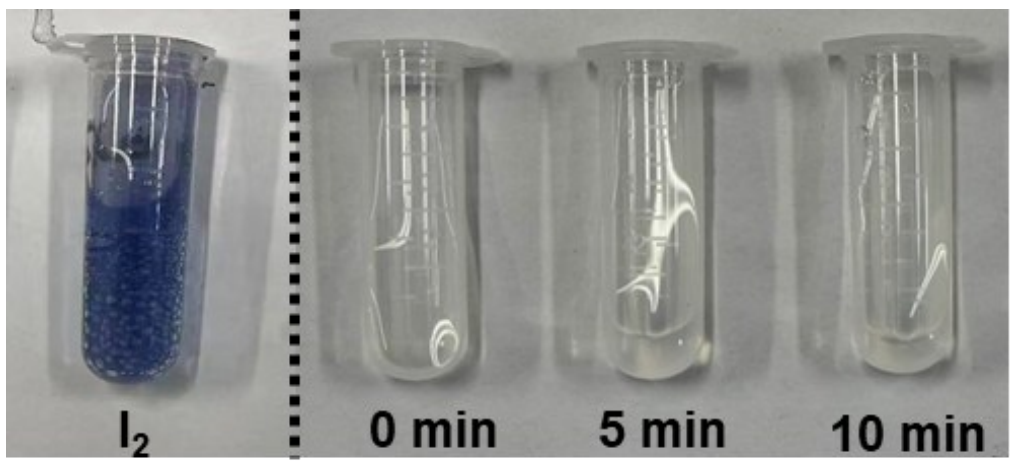

Fig. 6 (c) Detection of I_2 and I_3^- in the F-FeSAC/PI system.

Iodine mass balance and biological safety

A quantitative iodine mass balance was also established by monitoring IO_4^- and IO_3^- throughout the reaction. IO_4^- decreased continuously, while IO_3^- increased correspondingly (**Fig. 6a**). More importantly, the combined molar balance of IO_4^- and IO_3^- remained close to unity during the reaction, indicating high iodine recovery and confirming that the consumed IO_4^- was recovered predominantly as IO_3^-. Together with the absence of detectable I^-, I_2/I_3^-, iodophenols, and HRMS-screened iodinated organic byproducts, these results indicate that iodine was mainly retained as inorganic oxyiodine species rather than being incorporated into organic byproducts.

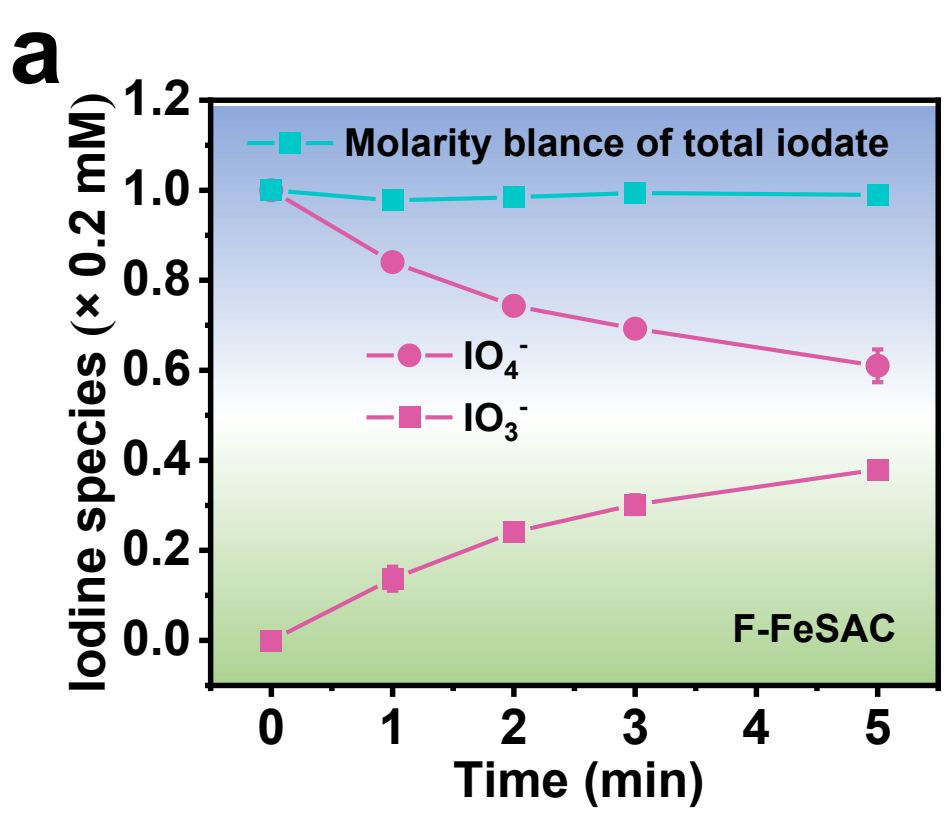

Fig. 6 (a) Concentration changes of IO_4^- and IO_3^- in the F-FeSAC/PI/4-CP system.

Finally, the biological safety of the treated effluent was further evaluated using

zebrafish and microbial bioassays. The untreated 4-CP solution caused severe toxicity, whereas the F-FeSAC/PI-treated solution showed markedly reduced biological toxicity, as evidenced by normal zebrafish morphology and high survival after 120 h of exposure (**Fig. 6c**). Flow-cytometry analysis further showed that the proportion of viable microbial cells increased from 56.2% to 79.3% after treatment, while dead cells decreased from 7.17% to 3.16% (**Fig. 6d-e**). These results further support that the treatment process reduced the overall ecotoxicity of the original pollutant solution.

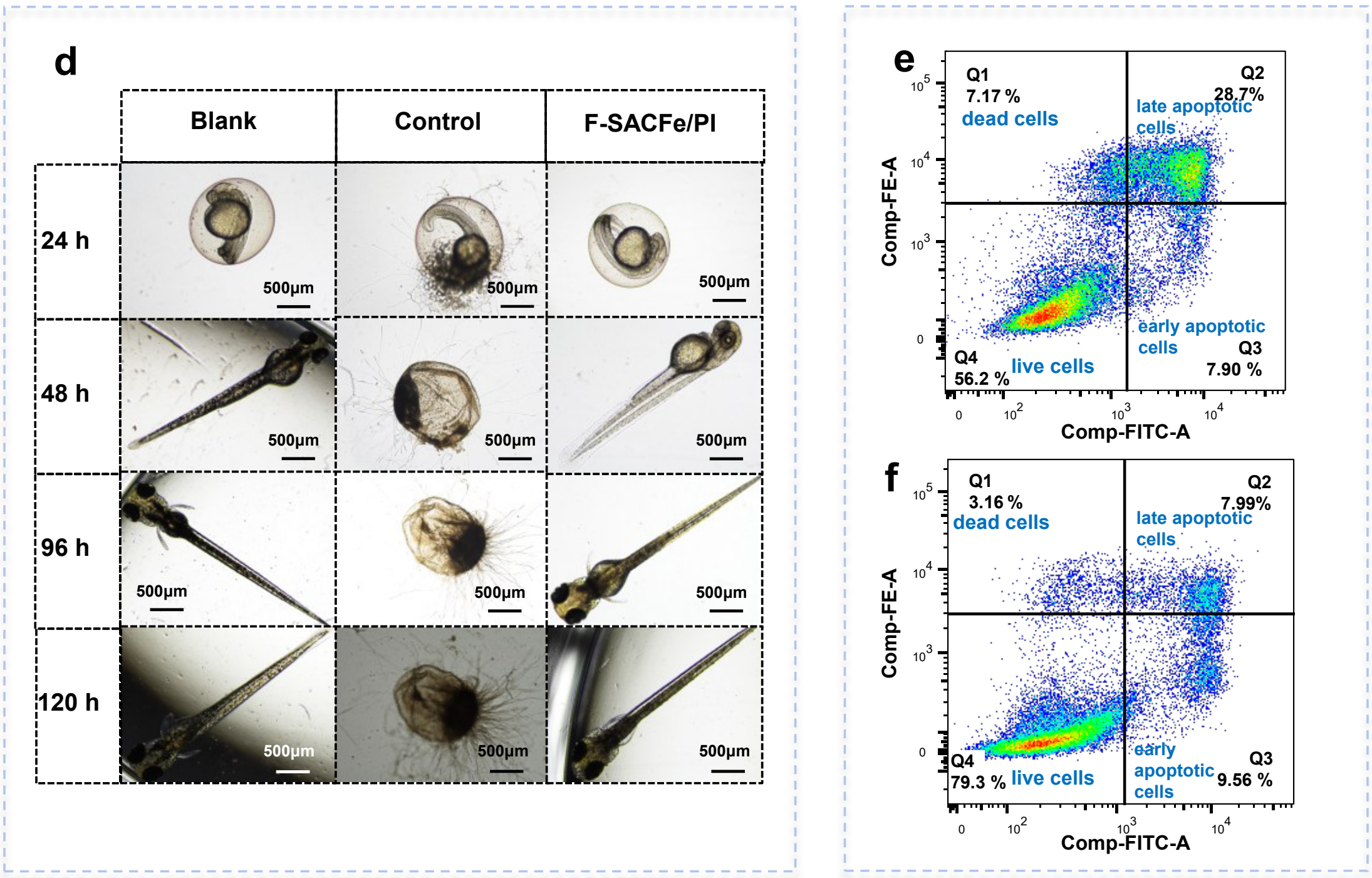

Fig. 6 (d) Images of zebrafish embryos under different treatments: nutrient medium only (Blank), 4-CP solution (Control), and post-reaction 4-CP solution from the F-FeSAC/PI system. Flow cytometry analysis of *E. coli* cells during 4-CP degradation in F-FeSAC/PI system at different reaction time: (e) 0 min, (f) 40 min. The four sections in flow cytometry images represent as below: Q1 dead cells; Q2 late apoptotic cells; Q3 early apoptotic cells; Q4 live cells.

Action 9: We have revised the iodine-species and toxicity analysis section. The representative EICs and mass spectra shown as Fig. R9-R10 in this response have been added to the revised Supporting Information as Supplementary Figs. 45-46, and the summary table of more than 200 theoretically possible iodinated candidates has been added as Supplementary Table 5. The revised version is provided below.

"The combined molar balance of IO_4^- and IO_3^- remained close to unity during the reaction, indicating high iodine recovery and confirming that consumed IO_4^- was

recovered mainly as IO_3^-. Given that HOI can iodinate phenolic compounds to form iodophenols, such as 4-iodophenol (4-IP) and 2-iodophenol (2-IP), targeted LC-HRMS analysis of 4-IP and 2-IP was further performed. As shown in ***Fig. 6b****, neither compound was detected, indicating no detectable HOI-mediated iodination under the tested conditions. Moreover, I^- was not detected by ion chromatography, and starch colorimetry showed no detectable response attributable to I_2/I_3^- accumulation in the F-FeSAC/PI process (****Fig. 6c and Supplementary Fig. 44****), corroborating the absence of reduced iodine species.*

*To further examine possible iodinated organic byproducts, high-resolution LC-MS suspect screening was performed for more than 200 theoretically possible iodinated compounds selected from plausible iodination products of 4-CP and its intermediates. None of the screened candidates met the assignment criteria for iodinated organic byproducts (****Supplementary Figs. 45-46 and Supplementary Table 5****).”*

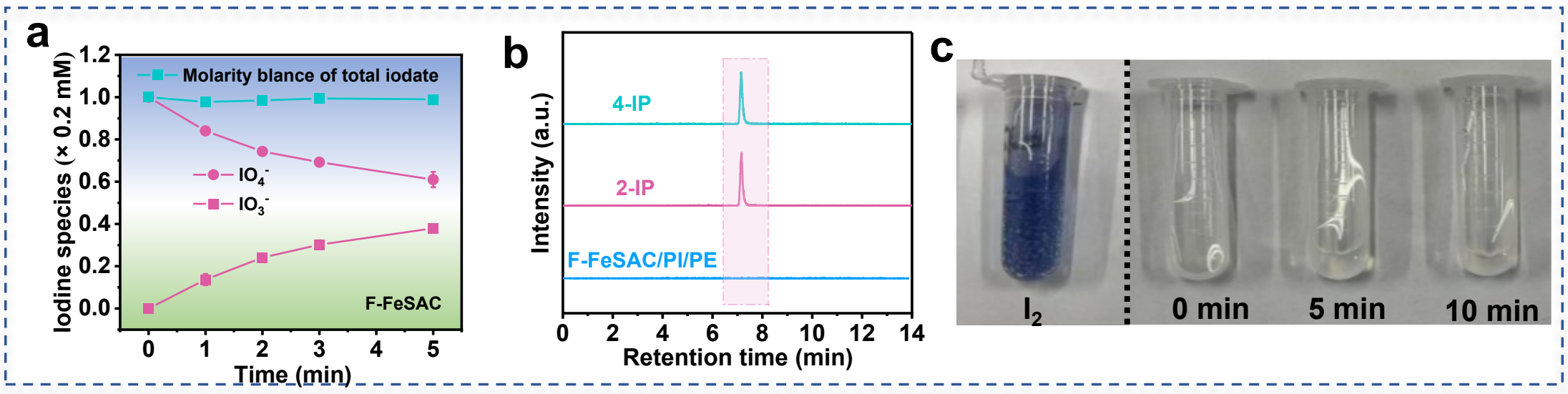

Fig. 6 (a) Concentration changes of IO_4^- and IO_3^- in the F-FeSAC/PI/4-CP system. (b) Detection of HOI generated in the F-FeSAC/PI system using phenol as a trapping agent. (c) Detection of I_2 and I_3^- in the F-FeSAC/PI system.

Reviewer #2 (Remarks to the Author):

This work establishes peripheral functionalization beyond the second coordination sphere as an enzyme-inspired design principle, enabling efficient pollutant removal via electron-transfer processes. The characterization techniques employed, including HRTEM, XAFS, electrochemical analysis, and DFT calculations, are adequate and consistent with typical studies in the field of single-atom catalysis, yet the overall approach appears somewhat homogenized. Electron-transfer processes (ETP) represent a currently active research topic, with various catalysts reported to exhibit promising performance. These include catalysts based on finely modulated steric positioning and tailoring of the d-band center in high-valent metal-oxo species. Against this context, what distinct advantages does the R-FeSAC catalyst offer relative to these systems? Several related studies focusing on the modulation of coordination environments have

already been reported, and the novelty of the present work appears to lie mainly in the introduction of PI. I cannot recommend this work for publication in NC. Furthermore, the current dataset still allows for alternative mechanistic interpretations, suggesting that several key conclusions require substantially more robust and quantitative validation.

Response:

We thank the reviewer for this thoughtful and important comment. We fully agree that ETP-related oxidation has become an active research area and that several elegant catalyst-design strategies, including charge-confined single-atom catalysts[2], axial-ligand engineering[40], steric regulation, and *d*-band tuning of high-valent metal-oxo species[36], have already been reported. We recognize that the previous version did not sufficiently distinguish the conceptual advance of this work from the broader field of ETP-related oxidation, which may have led to the reviewer's impression that our novelty primarily stems from introducing PI as the oxidant.

We would like to clarify that PI activation is used here as a model reaction to probe the catalytic consequence of distal electronic regulation, rather than being the sole source of novelty. Similarly, ETP is not presented merely as a newly discovered reaction route, but as the mechanistic manifestation of how remote peripheral substituents regulate the Fe single-atom center and promote the resulting oxidation pathway. The central advance of our work is the establishment of **peripheral substituent engineering beyond the second coordination sphere** as an enzyme-inspired design principle for single-atom catalysis, and in defining a structure-activity relationship governed by long-range electronic effects during PI activation.

Most reported SAC systems regulate catalytic behavior by directly modifying the metal center or its close coordination environment[41], including first-shell M–N_x coordination, heteroatom substitution near the metal site, axial ligation, neighboring defects, steric positioning, or the electronic structure of metal-oxo intermediates. These strategies are powerful but generally perturb the immediate coordination environment of the active center. In contrast, our R-FeSAC platform preserves a similar primary Fe–N_4 coordination structure while introducing tunable peripheral substituents (R = F, H, NH_2) beyond the second coordination sphere. This design allows us to decouple remote substituent effects from first-shell coordination changes and to examine whether long-range electronic perturbation alone can tune the Fe center, PI adsorption, and interfacial electron-transfer kinetics.

This distinction is important from an enzyme-mimetic perspective. Natural metalloenzymes achieve high activity and selectivity not only through the metal center and its directly bound ligands, but also through distal binding pockets that remotely modulate the electronic and steric environment of the active site[42]. However, this distal-pocket-like principle has been difficult to implement in synthetic SACs, particularly in conventional pyrolysis-derived M–N–C materials, where remote functional groups, coordination structure, defects, and local carbon environments are often uncontrollable under high-temperature conditions. Our non-pyrolytic conjugated organic SAC platform provides a structurally defined model to introduce peripheral organic substituents without substantially altering the primary $Fe–N_4$ coordination environment. Thus, the novelty of this work lies not simply in PI activation, but in translating enzyme-like distal regulation into an artificial SAC system and linking it to an observable structure-property-mechanism relationship.

Compared with representative ETP-related catalysts, R-FeSAC therefore offers several distinct advantages. First, it provides a more precise platform for separating remote electronic effects from first-shell coordination effects. Second, it uses simple peripheral substituents to tune the Fe electronic structure through long-range electronic communication across the conjugated scaffold. Third, it establishes a direct correlation between substituent electronic character, electronic configuration, PI adsorption, charge-transfer resistance, and pollutant-removal kinetics. Specifically, XPS and XANES reveal that the electron-withdrawing F substituent increases the effective valence state of Fe; EXAFS confirms that the Fe–N coordination structure remains similar across the R-FeSAC series; electrochemical measurements show accelerated interfacial electron transfer for F-FeSAC; and DFT calculations indicate that F substitution shifts the Fe *d*-band center upward, strengthens Fe–O interaction with PI, narrows the $LUMO_{(catalyst/PI)}–HOMO_{(pollutant)}$ energy gap, and thereby promotes ETP-driven oxidation. These results support a remote-substituent-governed catalytic mechanism rather than a simple oxidant replacement effect.

In terms of reaction pathway, our system also differs from previously reported non-radical ETP systems. For example, charge-confined SACs for PI activation couple electron transfer with singlet-oxygen-mediated oxidation[2], while other systems focus on axial coordination or high-valent metal-oxo chemistry to tune oxidative pathways[36]. In our R-FeSAC/PI system, multiple control experiments exclude dominant contributions from radicals, singlet oxygen, and high-valent Fe-oxo species. Instead, the catalyst/PI interfacial complex mediates direct electron transfer from electron-rich

pollutants, leading to coupling and polymerization products that accumulate on the catalyst surface. This ETP-induced polymerization pathway is further supported by the premixing experiment, PI consumption-pollutant removal correlation, in situ Raman spectroscopy, OCP/i-t/EIS measurements, galvanic oxidation experiments, LC-MS product analysis, and TGA/XPS evidence of surface oligomer deposition. Therefore, the present work identifies a distinct distal-substituent-regulated ETP polymerization mechanism rather than simply reporting another PI-based non-radical oxidation system.

Action: We have revised the Introduction to sharpen the rationale and novelty of our catalyst design.

Revised text in the Introduction:

"Nevertheless, several key challenges persist. First, conventional pyrolysis synthesis lacks the precision required to control the coordination environment of SACs. The inherent structural uncertainties of these uncontrolled high-temperature processes often lead to metal aggregation and ill-defined active sites, which obstruct a precise mechanistic understanding of SAC catalysis. Second, while the first coordination shell (FCS) is recognized as pivotal, research efforts have predominantly focused on its optimization. The influence of the second and higher coordination shells, which are not directly bonded to the metal center but critically modulate the electronic structure and catalytic efficiency, remains a largely unexplored frontier. More importantly, prevailing modulation strategies primarily involve tailoring coordination numbers, heteroatom doping, or creating carbon defects. In contrast, the potential of peripheral organic functional group substitution, a strategy that mirrors the long-range electronic effects in natural metalloenzymes, to regulate the catalytic properties of SACs has rarely been investigated, let alone for PI activation and the elucidation of structure-activity relationships.

Inspired by distal-pocket regulation in natural metalloenzymes, we leverage conjugated organic frameworks as programmable scaffolds to construct precision SACs. This strategy provides a precise route to manipulate the long-range coordination environment through peripheral organic group substitution. By incorporating electron-withdrawing (e.g., -F) or electron-donating (e.g., $-NH_2$) groups into the conjugated scaffolds, we synthesized a series of well-defined R-FeSACs (R = F, H, NH_2), achieving targeted electronic modulation of the Fe single-atom centers."

1. The authors have not fully decoupled the observed kinetics from morphology- and transport-related effects. This is because (i) PI activation may be governed by PI adsorption/partitioning and interfacial charge transfer, and (ii) differences in porosity and wettability can modulate local oxidant concentrations and effective collision frequencies.

Response 1:

We thank the reviewer for this insightful comment. We agree that the observed kinetic differences should be interpreted carefully, because PI adsorption/partitioning, interfacial charge-transfer behavior, and morphology-related factors such as porosity and wettability may all influence the apparent reaction rate.

First, to evaluate whether PI adsorption/partitioning plays a dominant role, we monitored IO_4^- depletion in the absence and presence of 4-CP (**Supplementary Fig. 23 and Fig. R12**). In the catalyst/$NaIO_4$ systems without 4-CP, IO_4^- showed only limited depletion within 5 min, with approximately 89-92% remaining in solution. This limited decrease may be associated with PI adsorption. By contrast, substantially stronger IO_4^- consumption was observed after the addition of 4-CP. These results indicate that IO_4^- is not extensively removed by simple adsorption consumption on surface consumption alone; instead, its pronounced depletion mainly occurs when pollutant oxidation is involved.

To further evaluate whether the consumed PI was effectively coupled to pollutant oxidation, we additionally calculated the IO_4^- utilization efficiency, defined as the moles of 4-CP degraded per mole of IO_4^- consumed. This parameter provides a more direct measure of how efficiently IO_4^- consumption is translated into pollutant removal. As shown in **Fig. R13**, F-FeSAC exhibited the steepest relationship between 4-CP degradation and IO_4^- consumption, indicating that more 4-CP molecules were degraded per mole of IO_4^- consumed. In other words, F-FeSAC achieved the highest PI utilization efficiency among the tested catalysts. This result suggests that the superior activity of F-FeSAC is more closely associated with PI activation driven by pollutant-involved interfacial electron transfer at the catalyst interface.

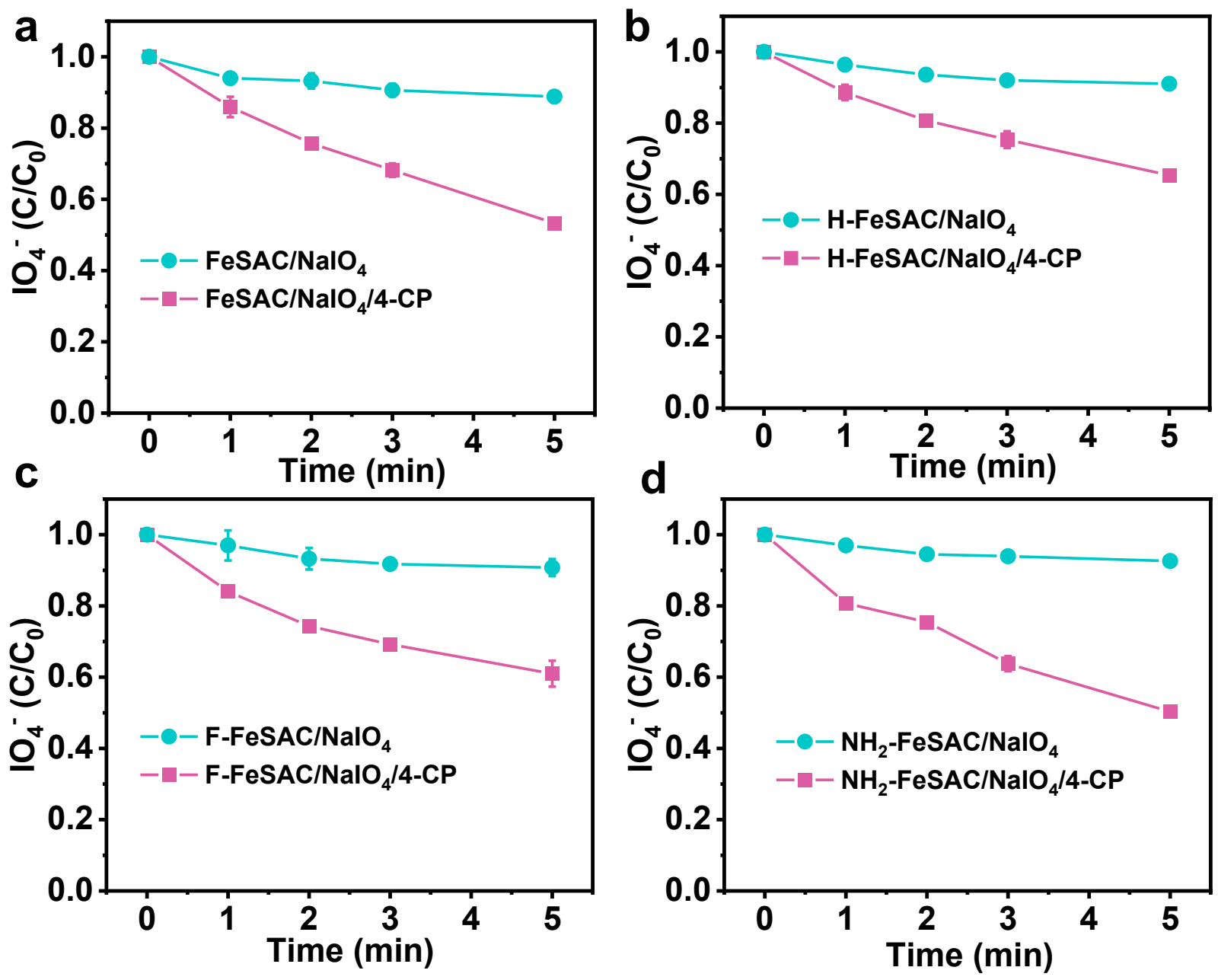

Supplementary Fig. 23 Adsorption and decomposition rate of PI in (a) FeSAC/PI, (b) H-FeSAC/PI, (c) F-FeSAC/PI and (d) NH_2-FeSAC/PI system.

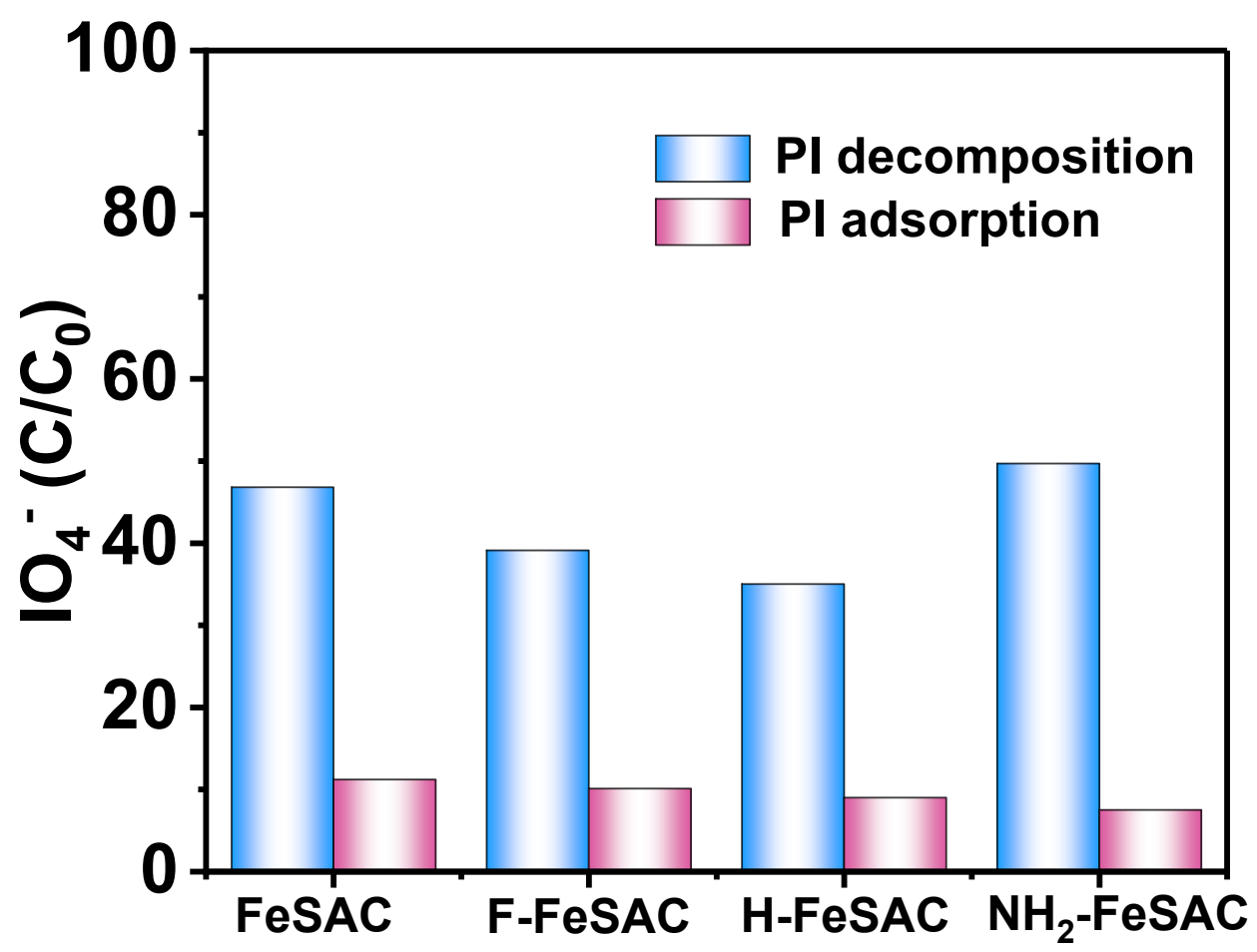

Fig. R12 Adsorption and decomposition rate of PI inFeSAC/PI, H-FeSAC/PI, F-FeSAC/PI and NH_2-FeSAC/PI system.

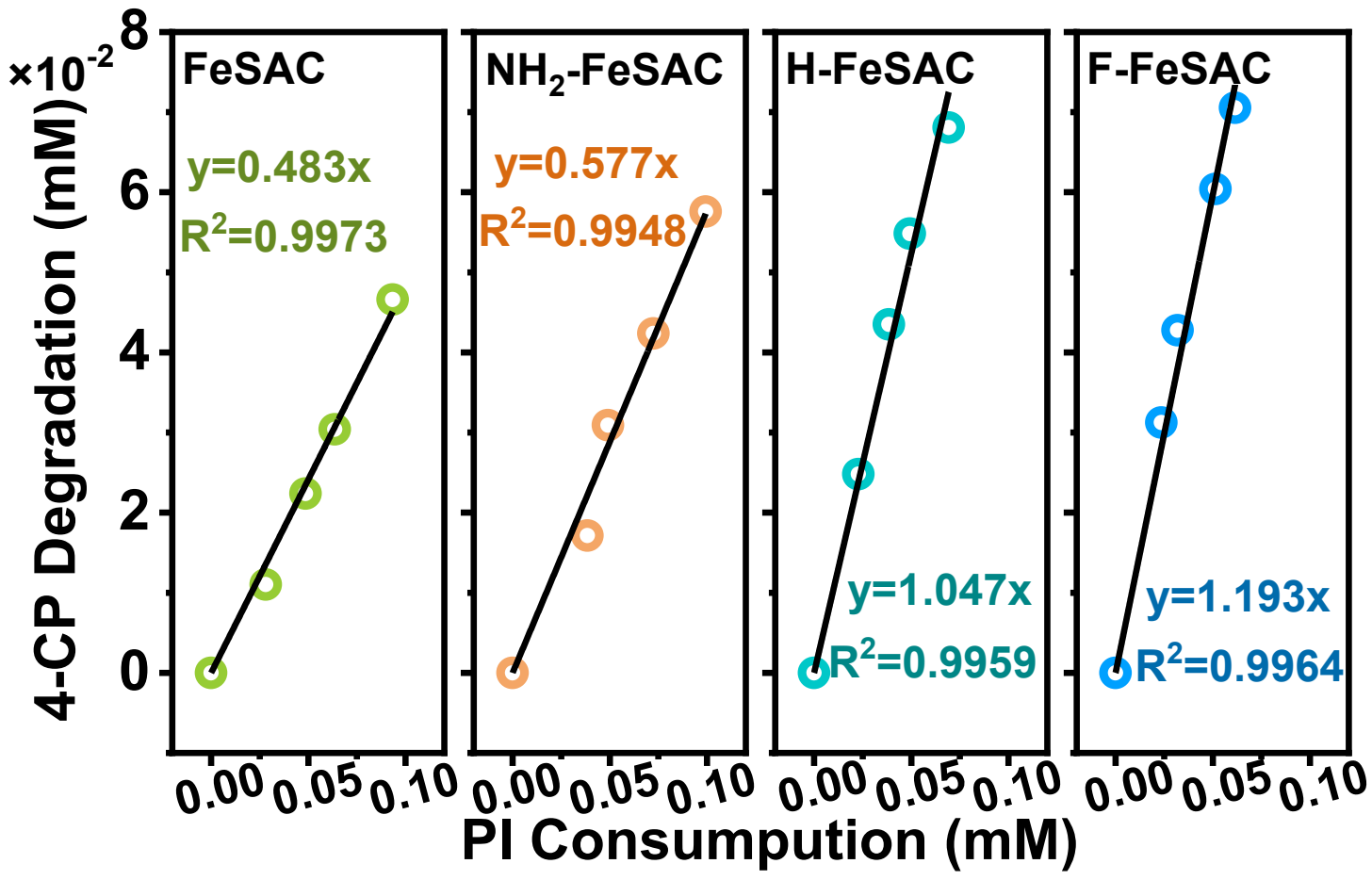

Fig. R13 Correlation between 4-CP degradation and PI consumption in FeSAC/PI, NH_2-FeSAC/PI, H-FeSAC/PI and F-FeSAC/PI systems.

Second, we examined the interfacial electronic process by sequential-addition chronoamperometry. Upon PI addition, all catalysts showed an immediate cathodic current response, indicating rapid formation of electroactive catalyst/PI interfacial states (**Fig. R3**). More importantly, subsequent addition of 4-CP triggered a second distinct current response, showing that the pollutant further participates in the interfacial electronic process. Among the tested catalysts, F-FeSAC displayed the strongest current response, especially after 4-CP injection, consistent with stronger interfacial electronic coupling and more efficient pollutant-involved charge-transfer behavior. This interpretation is further supported by the EIS results, in which F-FeSAC exhibits the smallest semicircle diameter among the tested catalysts (**Supplementary Fig. 26**), indicating the lowest interfacial charge-transfer resistance. Together with the highest IO_4^- utilization efficiency, these electrochemical results suggest that the catalyst-dependent kinetic differences are not solely determined by oxidant accessibility, but are closely associated with the efficiency of interfacial electron transfer.

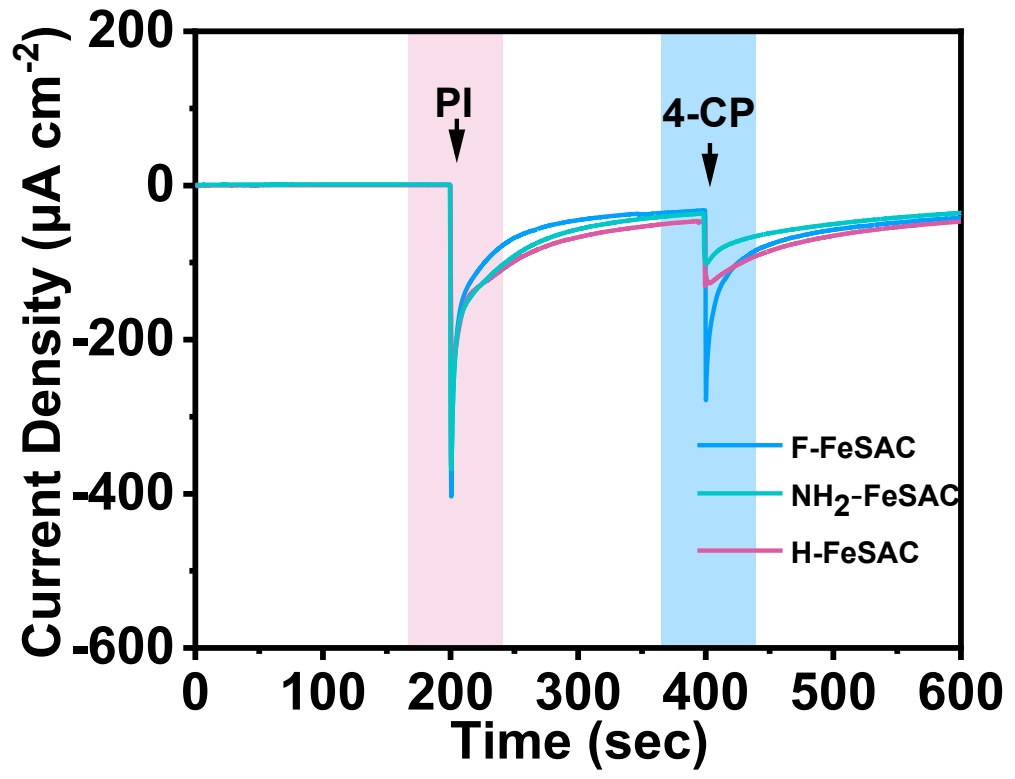

Fig. R3 i-t curve of F-FeSAC, H-FeSAC and NH_2-FeSAC.

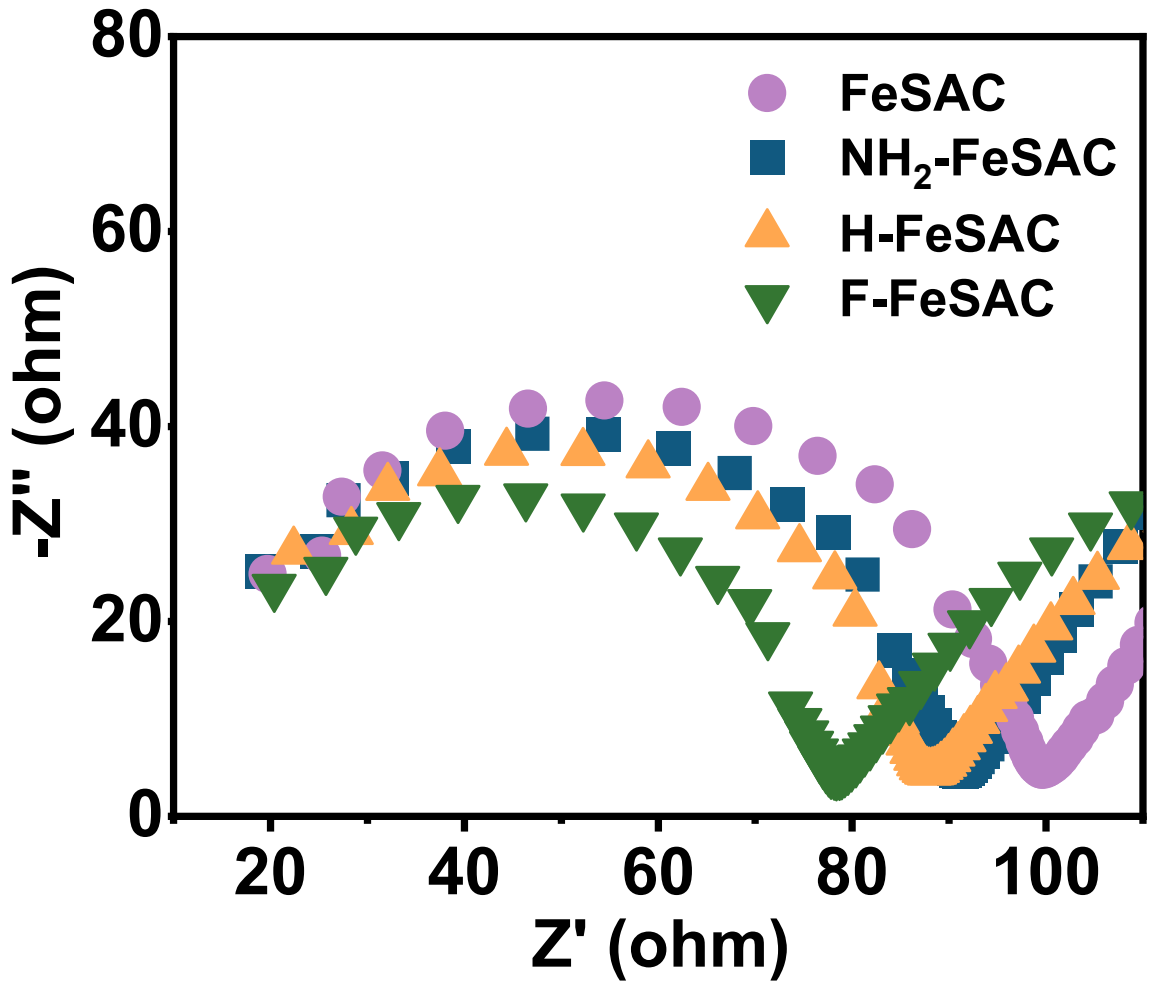

Supplementary Fig. 26 EIS analyses spectra of FeSAC, F-FeSAC, H-FeSAC and NH_2-FeSAC.

Third, we evaluated whether porosity and wettability could account for the activity trend. N_2 adsorption-desorption measurements show that the BET surface areas of FeSAC, H-FeSAC, F-FeSAC, and NH_2-FeSAC are 29.86, 43.71, 36.62, and 46.61 m^2 g^{-1}, respectively, and the corresponding pore volumes are 0.222, 0.282, 0.257, and 0.302 cm^3 g^{-1} (**Supplementary Table 1**). In particular, F-FeSAC does not have the largest BET surface area or pore volume, although it exhibits the highest activity (**Fig. 3a**). We therefore further normalized the catalytic activity by the BET specific surface area (**Table R2**). The surface-area-normalized rate constants of F-FeSAC, H-FeSAC, and NH_2-FeSAC are 0.0210, 0.0087, and 0.0054 $min^{-1}/(m^2 \cdot g^{-1})$, respectively. F-FeSAC remains the most active catalyst after normalization.

Supplementary Table 1. BET surface area, pore volume and average pore diameter of FeSAC and R-FeSAC.

Catalysts	BET specific surface area (m^2 g^{-1})	Pore volume (cm^3 g^{-1})
FeSAC	29.8588	0.222440
H-FeSAC	43.7142	0.282100
F-FeSAC	36.6166	0.256884
NH_2-FeSAC	46.6077	0.302192

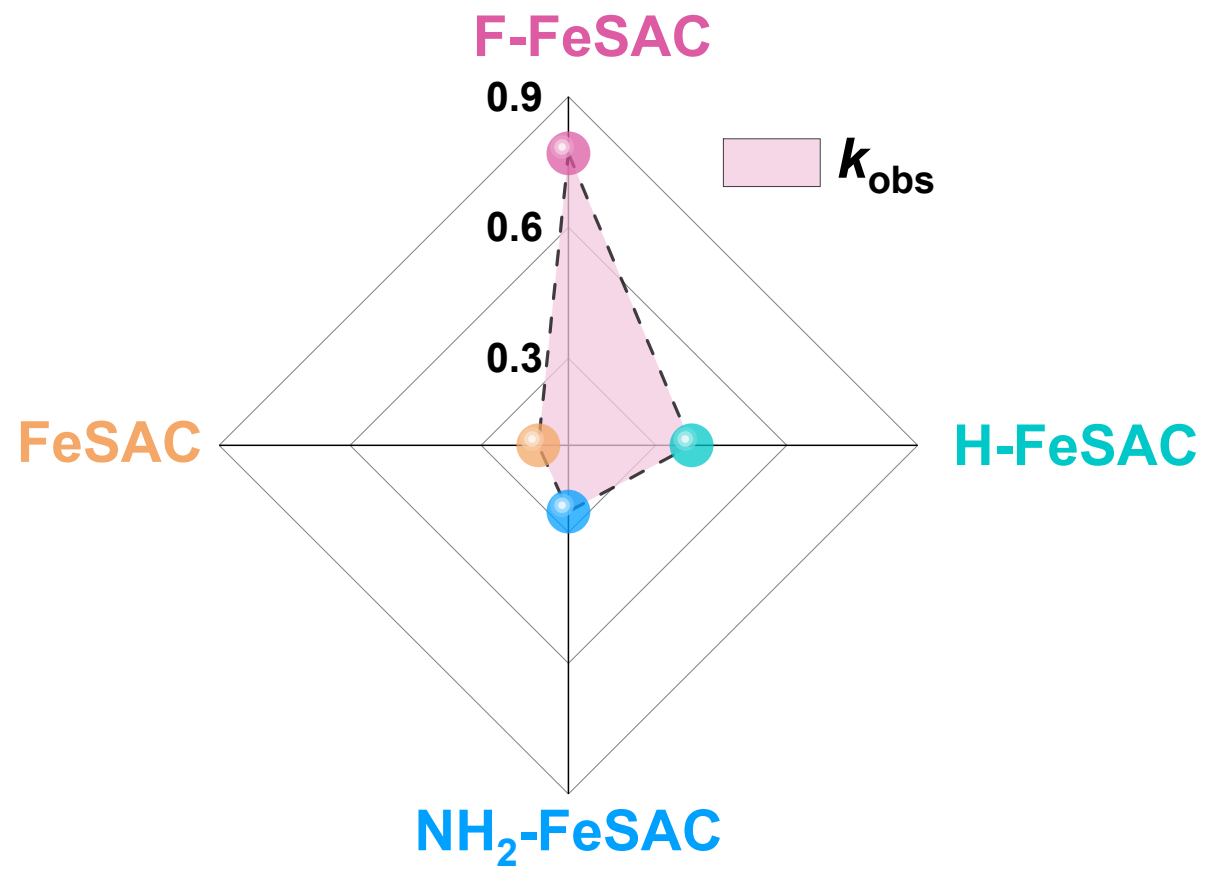

Fig. 3 (a) The k_{obs} of different catalytic systems on the degradation of 4-CP.

Table R2. Comparison of area-normalized rate constants ($K_{per\text{-}area}$) for FeSAC-based PI systems with varying functional group.

**Catalysts**	**BET (m^2/g)**	**k_{obs} (min^{-1})**	**$K_{per\text{-}area}$ ($min^{-1}/(m^2 \cdot g^{-1})$)**
**F-FeSAC**	**36.6166**	**0.770**	**0.0210**
H-FeSAC	43.7142	0.382	0.0087
NH_2-FeSAC	46.6077	0.252	0.0054
FeSAC	29.8588	0.169	0.0057

We also compared the surface wettability of the catalysts by contact-angle measurements. The average contact angles of H-FeSAC, F-FeSAC, and NH_2-FeSAC are 45.13°, 43.67°, and 41.14°, respectively (**Fig. R14**). The relatively small differences among these values indicate comparable wettability across the three catalysts. Moreover, the contact-angle trend does not follow the catalytic activity order, as NH_2-FeSAC is slightly more hydrophilic than F-FeSAC but exhibits lower catalytic activity. Therefore, wettability may affect local oxidant distribution to some extent, but it is unlikely to be the dominant factor governing the observed kinetic differences.

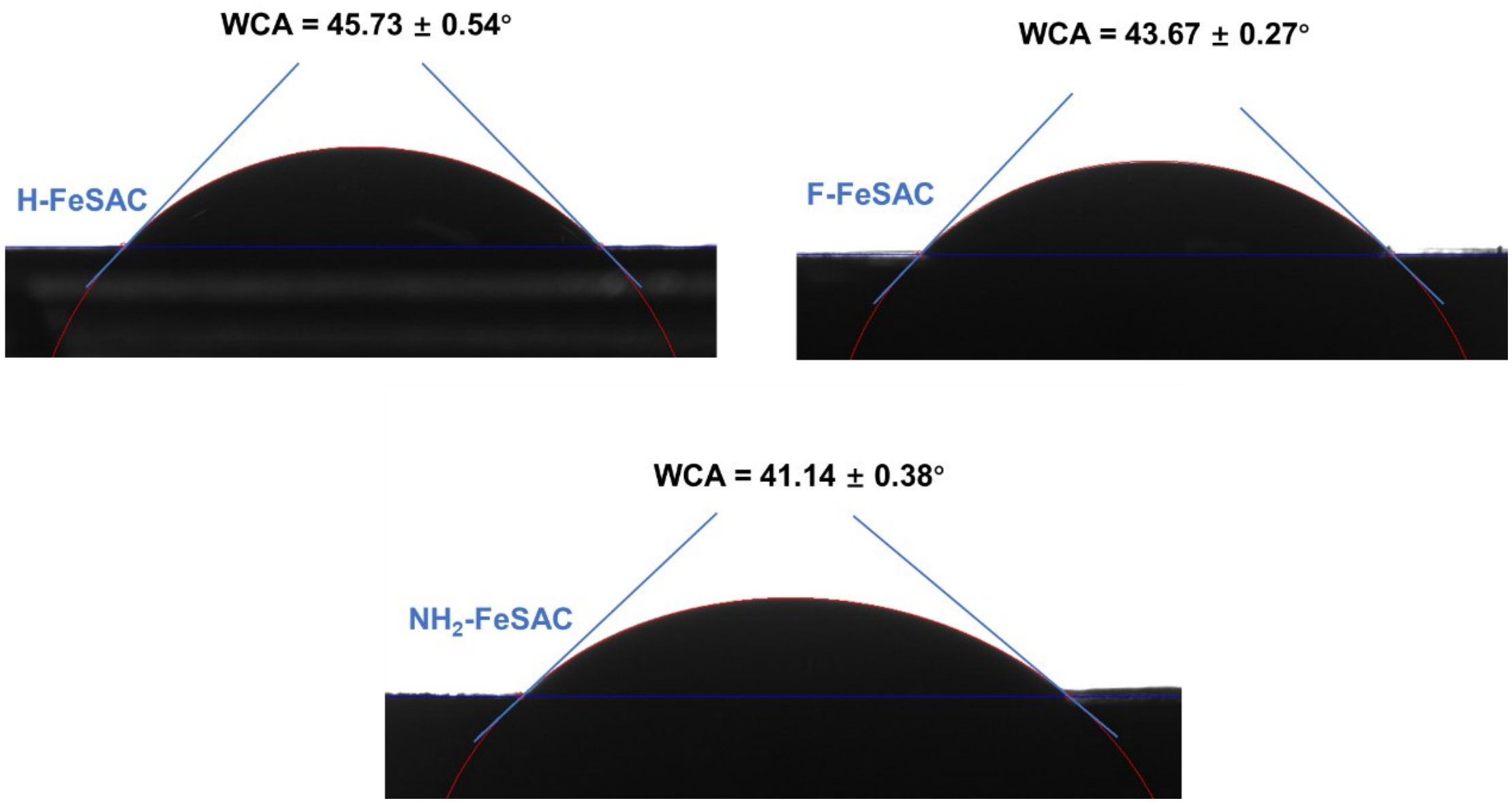

Fig. R14 Water contact angle measurements of H-FeSAC, F-FeSAC, and NH_2-FeSAC.

Overall, these complementary analyses provide a more complete evaluation of the apparent kinetics. Although PI adsorption/partitioning, porosity, and wettability may affect local oxidant availability and interfacial contact, the higher IO_4^- utilization efficiency of F-FeSAC, together with its stronger chronoamperometric response and lower charge-transfer resistance, indicates that more productive PI activation and faster pollutant-involved interfacial electron transfer are key contributors to its enhanced catalytic performance.

2. Turnover frequency is suggested to calculate for comparison.

Response 2:

We thank the reviewer for this valuable suggestion. We agree that turnover frequency (TOF) is a more appropriate metric for evaluating and comparing the intrinsic activity of catalytic sites than pollutant removal efficiency or apparent rate constant alone. In heterogeneous single-atom catalytic systems, the overall degradation performance can be affected by catalyst dosage, metal loading, and active-site density. Therefore, TOF provides a more meaningful basis for comparison because it normalizes the reaction rate to the amount of catalytically active sites.

Following the reviewer's suggestion, we have calculated the TOF values of our catalysts and included the corresponding results in the revised manuscript. The TOF was calculated according to the amount of pollutant converted per mole of Fe sites per unit time, using the following expression[43, 44, 45, 46]:

$$\text{TOF [min}^{-1}] = \frac{\text{moles of reactant converted}}{\text{moles of active sites} \times \text{reaction time}} = \frac{\Delta n(\text{pollutant}) \times M_{\text{metal}}}{m_0 \times \omega_{\text{metal}} \times t}$$

Where Δn (pollutant) is moles converted, M_{metal} is metal atomic weight, m_0 is catalyst mass, ω_{metal} is metal mass fraction, and t is reaction time. All parameters were derived from our kinetic experiments and site quantification analyses.

The calculated TOF values have now been added to **Supplementary Fig. 11 and Table 4**. The F-FeSAC catalyst exhibits a notably high TOF value of 0.345 min^{-1}, outperforming many recently reported coordination-regulated SAC systems. The results further confirm that our catalyst exhibits superior intrinsic site activity, rather than merely showing an apparent advantage arising from dosage or composition differences. We believe that this addition makes the performance comparison more rigorous and convincing.

Action 2: We have the added relevant statements in the Supplementary Fig. 11 and Table 4 has been added in the supporting information.

*"To distinguish intrinsic site activity from apparent performance gains associated with dosage or oxidant concentration, the turnover frequency (TOF) was further evaluated and compared with previously reported coordination-regulated SAC systems. As shown in **Supplementary Fig. 11 and Supplementary Table 4**, F-FeSAC delivered a high TOF of 0.345 min^{-1}, demonstrating that its superior activity arises from the enhanced reactivity of individual Fe sites."*

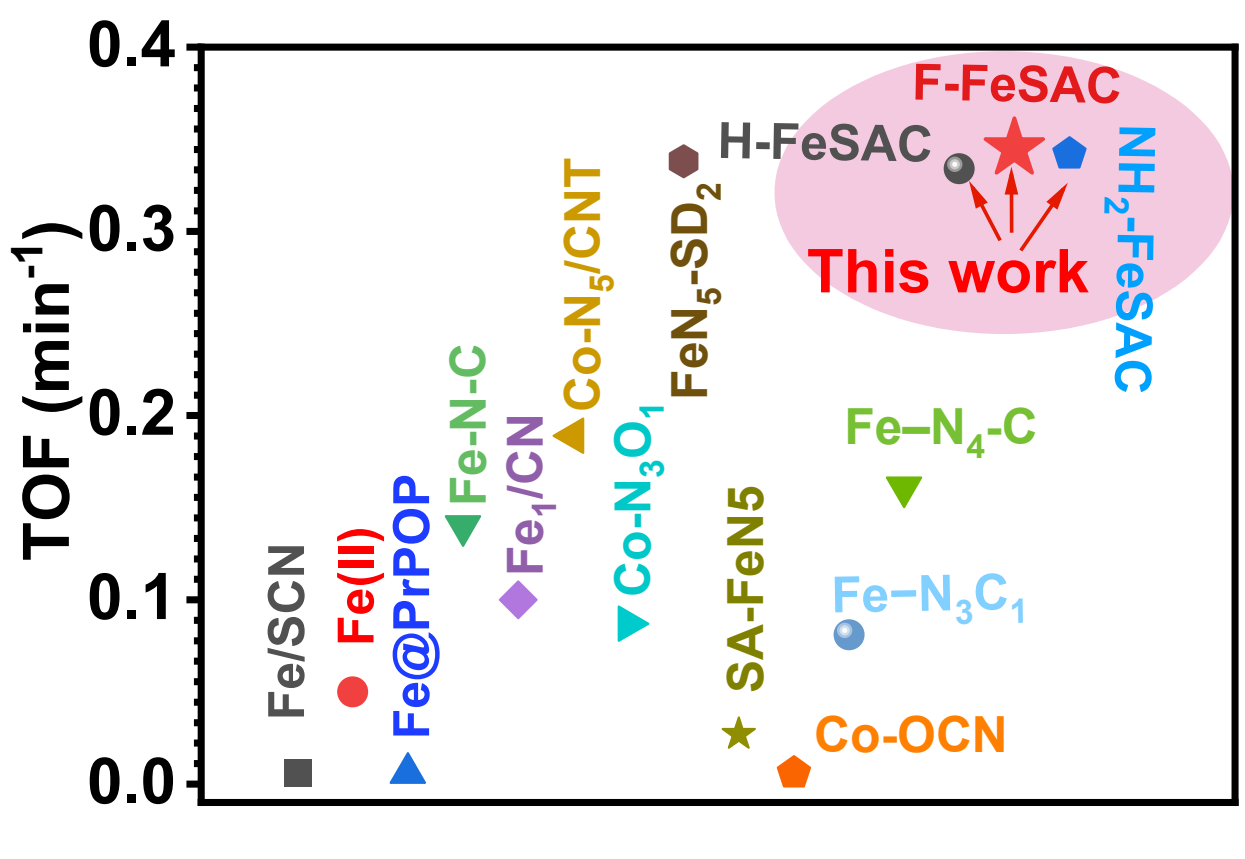

Supplementary Fig. 11 Comparison of TOF values for pollutant degradation in SAC systems featuring coordination environment regulation.

Supplementary Table 4. Catalytic performances of SAC-based systems regulated *via* coordination environment engineering.

Catalysts	Δn(pollutants) ($\times 10^{-6}$ mol)	ω_{metal} (%)	Reaction time (min)	TOF (min^{-1})	Ref.
Fe/SCN (0.5 g L^{-1})	4-CP (100)	6.20	30	0.006	[2]
Fe(II) (0.25 mM)	SMX (24.9)	100	2	0.050	[4]
Fe@PrPOP (0.1 g L^{-1})	BPA (5)	3.62	12	0.006	[47]
Fe–N–C (0.10 g L^{-1})	BPA (100)	2.00	20	0.140	[48]
Fe_1/CN (0.05 g L^{-1})	4-CP (100)	11.2	10	0.100	[49]
Co-N_5/CNT (0.03 g L^{-1})	SMZ (40)	1.39	30	0.189	[50]
CoN_3O_1 (0.10 g L^{-1})	CIP (15)	0.51	20	0.087	[51]
FeN_5-SD_2 (0.03 g L^{-1})	BPA (80)	2.21	20	0.338	[32]
SA-FeN_5 (0.20 g L^{-1})	APAP (30)	2.63	12	0.027	[52]
Co-OCN (0.03 g L^{-1})	APAP (13)	7.25	60	0.006	[53]
Fe–N_3C_1 (0.06 g L^{-1})	SIZ (19.6)	0.75	30	0.081	[54]
Fe-N_4-C (0.10 g L^{-1})	NPX (43)	1.50	10	0.161	[55]
**H-FeSAC (0.05 g L^{-1})**	**4-CP (79.2)**	**2.65**	**10**	**0.334**	**This work**
**F-FeSAC (0.05 g L^{-1})**	**4-CP (80)**	**2.59**	**10**	**0.345**	**This work**
**NH_2-FeSAC (0.05 g L^{-1})**	**4-CP (73.7)**	**2.41**	**10**	**0.342**	**This work**

3. Line 248: Notably, CNTs exhibited no PI activation capability. Line 82: Lai et al. recently identified a ETP pathway in the carbon nanotube (CNT)/PI system, wherein CNTs function as an electron shuttle to mediate direct electron transfer from organic contaminants to PI. What is the reason for this discrepancy?

Response 3:

We thank the reviewer for pointing out this apparent discrepancy. We apologize that our original wording may have caused confusion. The CNTs referred to in these two contexts are not equivalent. Specifically, Lai et al. investigated CNTs with catalytically relevant surface structures for PI activation, whereas the CNTs used in our study were pristine, untreated CNTs introduced only as a conductive support.

In the work of Lai et al., the CNTs were surface-modified through oxidative treatment, oxygen-containing functional-group enrichment, and defect/edge-site generation, and were systematically characterized as reactive carbon materials for PI activation. In particular, carbonyl/ketonic groups and double-vacancy defects were identified as the key active motifs responsible for forming high-potential CNT-PI* intermediates. Therefore, their CNTs functioned as catalytically active, defect- and oxygen-functionalized carbon materials, rather than as a simple conductive support.

By contrast, the CNTs used in our work were not subjected to oxidative activation, defect engineering, or deliberate surface functionalization. They were introduced only as a conductive support to anchor the Fe single-atom sites. This difference in surface chemistry and defect structure explains the apparent discrepancy. Under our routine reaction conditions ([4-CP] = 80 μM, [CNT] = 50 mg/L, [PI] = 0.20 mM, 25 °C, without pH adjustment), the pristine CNT support alone showed negligible PI activation when used as the control group, whereas the Fe-containing catalyst exhibited pronounced activity. This result indicates that, in our catalyst design, PI activation is dominated by the Fe single-atom centers rather than by the bare CNT support.

Thus, the apparent discrepancy reflects the different surface chemistry and defect structures of the CNTs used in the two studies, rather than a contradiction in the role of CNTs in PI activation. Lai et al. demonstrated PI activation over defect- and oxygen-functionalized CNTs bearing catalytically active surface motifs, whereas the pristine CNT support used as the control group in our study showed negligible PI activation under the present conditions.

Action 3: We have corrected this statement in the revised manuscript.

"Lai et al.[7] *recently identified an ETP pathway in the functionalized carbon nanotube*

(CNT)/PI system, in which CNTs were modified through oxidative treatment to introduce oxygen-containing functional groups, defect sites, and edge structures. Carbonyl/ketonic groups and double-vacancy defects were proposed to function as electron-shuttling motifs that mediate direct electron transfer from organic contaminants to PI."

"Notably, the pristine CNT support showed negligible PI activation under our experimental conditions (Supplementary Fig. 9), whereas all R-FeSAC catalysts markedly outperformed pristine FeSAC (Fig. 3a and Supplementary Fig. 10)."

4. The DFT parameters to calculate HOMO values of pollutants should be provided.

Response 4:

We thank the reviewer for this helpful comment. We have now clarified the computational parameters used to obtain the HOMO values of the pollutant molecules in the revised manuscript.

Action 4: We have the added relevant statements in the supporting information.

"All calculations were performed using the Vienna Ab initio Simulation Package (VASP) within the projector augmented wave (PAW) framework and the Perdew-Burke-Ernzerhof (PBE) generalized gradient approximation. A plane-wave cutoff energy of 400 eV was used. For the catalyst slab models, the Brillouin zone was sampled using a 2 × 2 × 1 k-point mesh. For the pollutant molecules, isolated-molecule calculations were carried out in a sufficiently large vacuum box, and only the Γ point was used for Brillouin-zone sampling. The geometries of the molecules were fully optimized until the energy and force converged to 10^{-4} eV and 0.02 eV $Å^{-1}$, respectively. van der Waals interactions were described using the DFT-D3 method. The HOMO values reported in this work were extracted from the Kohn-Sham eigenvalues of the optimized isolated pollutant molecules. These values were used to compare the relative electron-donating ability of different pollutants under the same computational protocol."

5. Clarify terminology: describing the process as "Fenton-like" may be misleading if the dominant mechanism is non-radical ETP.

Response 5:

We thank the reviewer for this important comment. We agree that the term "Fenton-like" may be misleading in our case because the dominant pathway is a non-radical electron-transfer process rather than conventional radical oxidation. To avoid ambiguity, we have revised the terminology throughout the manuscript. The process is now

described as **"periodate activation"**, and the use of "Fenton-like" has been removed where it could imply a radical-based mechanism. These changes make the mechanistic description more precise and fully consistent with our experimental evidence.

6. I note that the degradation performance remains efficient even in a pilot-scale ETP reactor. Given this, what factors currently hinder its practical application?

Response 6:

Thank you for this insightful comment. We agree that the consistently high performance observed in both the small-scale tests and the pilot-scale ETP reactor demonstrates the strong practical potential of F-FeSAC/PI system. In our study, the catalyst showed good stability and strong tolerance to matrix interference over different scales (**Fig. 5f-m**), indicating that catalytic performance itself is not the primary barrier at the current stage.

[Figure Redacted]

Fig. 5 (f) Effects of various ionic species and water matrices on 4-CP degradation. (g) Photographs of the continuous-flow experiment in operation. (h) Water flux of differen t membranes during the continuous-flow process (insets show average flux values). (i) 4-CP removal efficiency of different systems. (j) COD removal in two real WWTP sc enarios. (k) 4-CP removal in the continuous-flow experiment for two real WWTP scen arios. (l) Image of the amplifying ETP reactor. (m) 4-CP removal efficiency and PI co nsumption in the amplifying ETP reactor.

That said, some distance still remains between promising laboratory or pilot-scale result and real-world implementation. At present, the more critical limitations are related to practical cost, including reagent input and other operational consumables. In addition, as with many SACs systems, the synthesis conditions, and cost of large-scale production remain important factors that may hinder broader application.

We therefore view the present work as a meaningful step forward for advanced oxidation, rather than a complete solution for immediate full-scale deployment. The main objective of this study is to clarify the structure-effect relationship and the underlying microscopic mechanism of the catalyst in the ETP process. Although we have made an initial attempt toward practical application and obtained encouraging results, further efforts in process optimization, cost control, and scalable catalyst production will still be required before real implementation can be fully realized.

7. When HOI concentrations fall below the detection limit of HPLC, the use of HPLC for the detection of 4-iodophenol (4-IP) and 2-iodophenol (2-IP) is not suitable. Instead, LC-MS is recommended.

Response 7:

We sincerely thank the reviewer for this valuable and technically important suggestion. We agree that HPLC alone may not provide sufficient sensitivity for assessing trace iodinated phenolic products when HOI, if formed, is present below the HPLC detection limit. Following the reviewer's recommendation, we re-analyzed the reaction samples using liquid chromatography-high-resolution mass spectrometry (LC-HRMS), which provides higher sensitivity, mass accuracy, and molecular selectivity for trace-level detection of 2-iodophenol (2-IP) and 4-iodophenol (4-IP).

Specifically, the treated solutions were analyzed using an LC system coupled to a SCIEX X500B QTOF high-resolution mass spectrometer. The extracted ion chromatograms (EICs) of 2-IP and 4-IP were monitored using their accurate mass signals within a mass tolerance of ±5 ppm, together with retention-time comparison against authentic standards when available. Under these LC-HRMS conditions, neither 2-IP nor 4-IP was detected in the reaction solution after F-FeSAC/PI treatment (**Fig. 6b**).

This additional LC-HRMS analysis excludes insufficient HPLC sensitivity as the reason for the previous non-detection of iodophenols. More importantly, it further supports that HOI-derived iodination products were not observed in the present system

under the tested conditions.

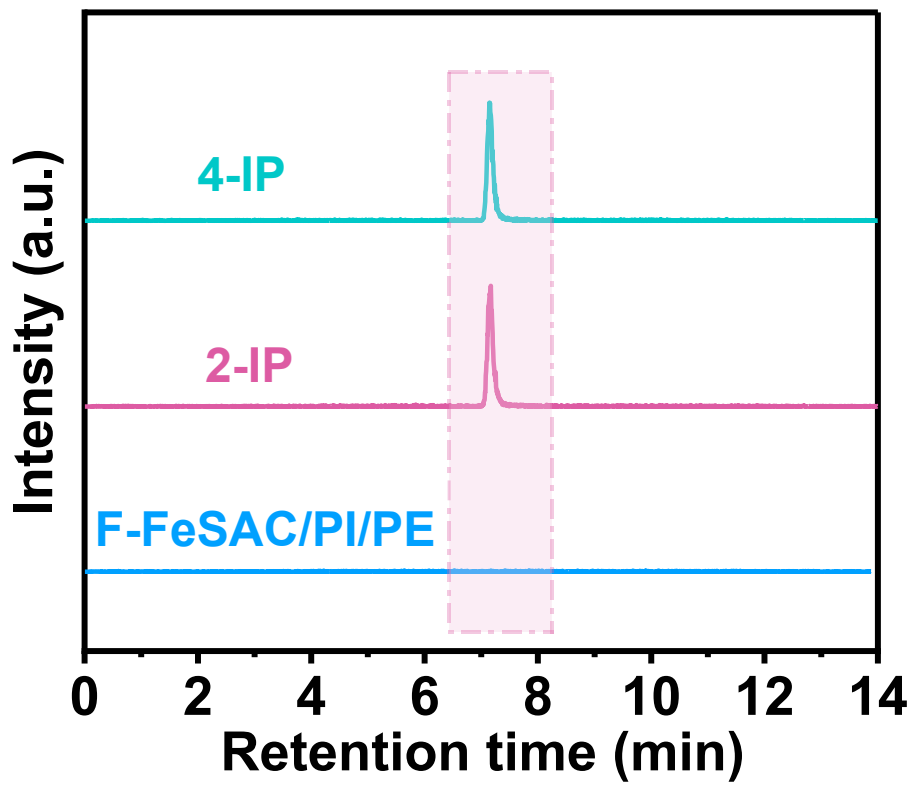

Fig. 6 (b) Detection of HOI generated in the F-FeSAC/PI system using phenol as a trapping agent.

Action 7: We have revised the manuscript accordingly to clarify that LC-HRMS was used to verify the absence of 2-IP and 4-IP. The revised version is provided below.

*"Given that HOI can iodinate phenolic compounds to form iodophenols, such as 4-iodophenol (4-IP) and 2-iodophenol (2-IP), targeted LC-HRMS analysis of 4-IP and 2-IP was further performed. As shown in **Fig. 6b**, neither compound was detected, indicating no detectable HOI-mediated iodination under the tested conditions."*

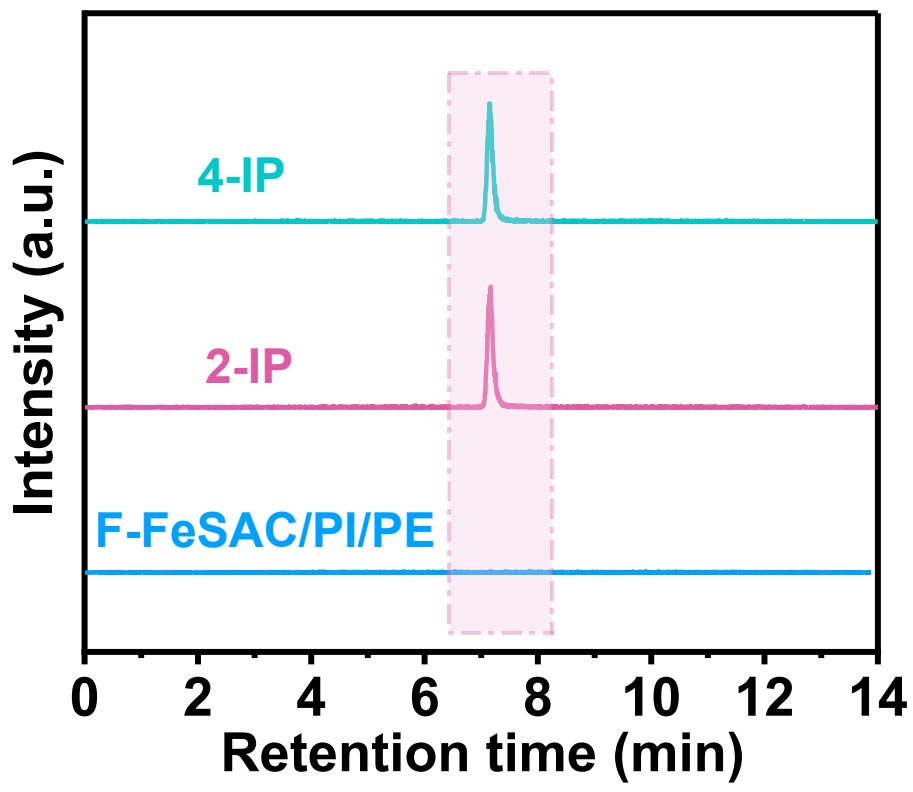

Fig. 6 (b) Detection of HOI generated in the F-FeSAC/PI system using phenol as a trapping agent.

8. Fig. S20. The authors are requested to calculate the utilization efficiency of $IO4^-$ (i.e., the moles of 4-CP degradable per mole of $IO4^-$), and explain whether there are differences in the utilization efficiency of $IO4^-$ among FeSAC modified with different substituents, and whether fluorine substituents can improve the effective activation ratio of $IO4^-$? It appears that H-FeSAC exhibits the highest PI utilization efficiency. Please quantify the corresponding difference and provide a detailed discussion.

Response 8:

We thank the reviewer for this important and constructive suggestion. Following this comment, we re-examined the 4-CP degradation data together with the corresponding IO_4^- consumption data and calculated the utilization efficiency of IO_4^-, defined here as the apparent moles of 4-CP removed per mole of IO_4^- consumed.

To ensure a meaningful comparison among the different catalysts, we calculated this efficiency using the initial fast-reaction period rather than the entire reaction duration because the reaction over F-FeSAC proceeds much faster than those over the other catalysts. As shown in the degradation profiles, 4-CP is almost completely removed within the first 5 min in the F-FeSAC/PI system, after which the degradation curve reaches a plateau (**Supplementary Fig. 10**). In contrast, 4-CP degradation in the FeSAC, H-FeSAC, and NH_2-FeSAC systems is still ongoing during this period. For this reason, we used the first 5 min, during which both 4-CP degradation and IO_4^- consumption showed a reliable kinetic correlation, to evaluate the intrinsic coupling between pollutant oxidation and IO_4^- consumption.

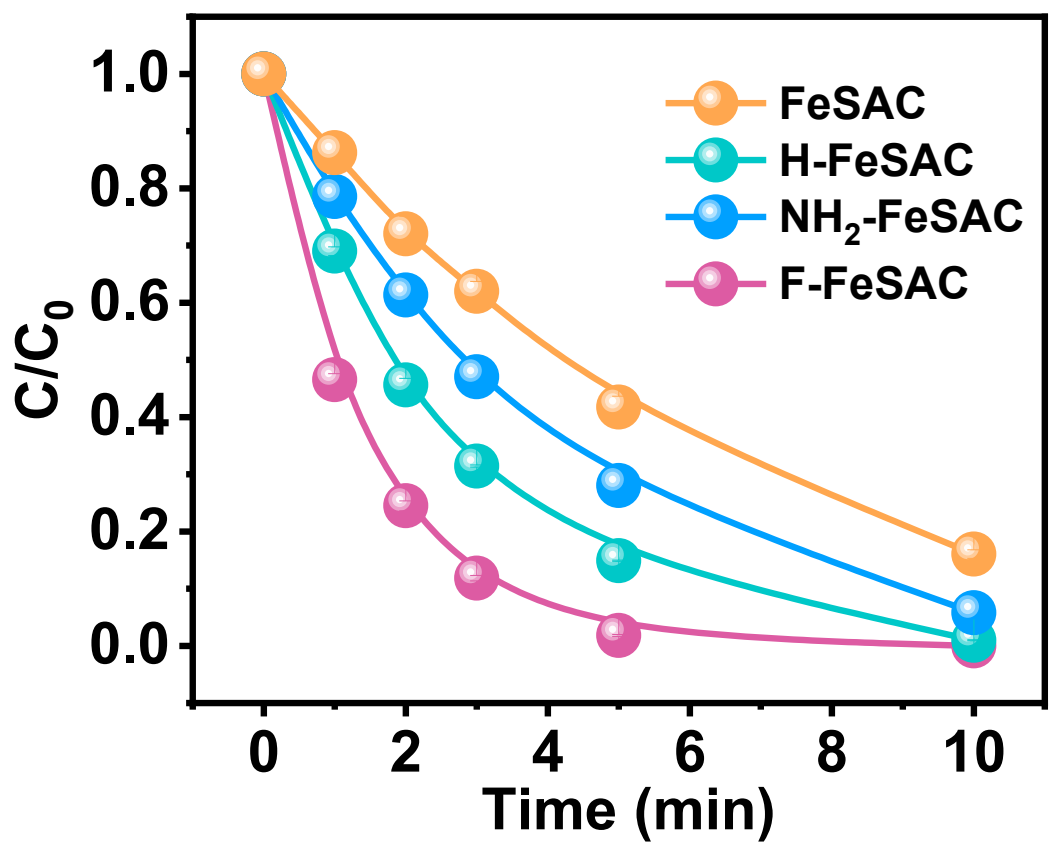

Supplementary Fig. 10 Effects of different catalytic systems on the degradation of 4-CP.

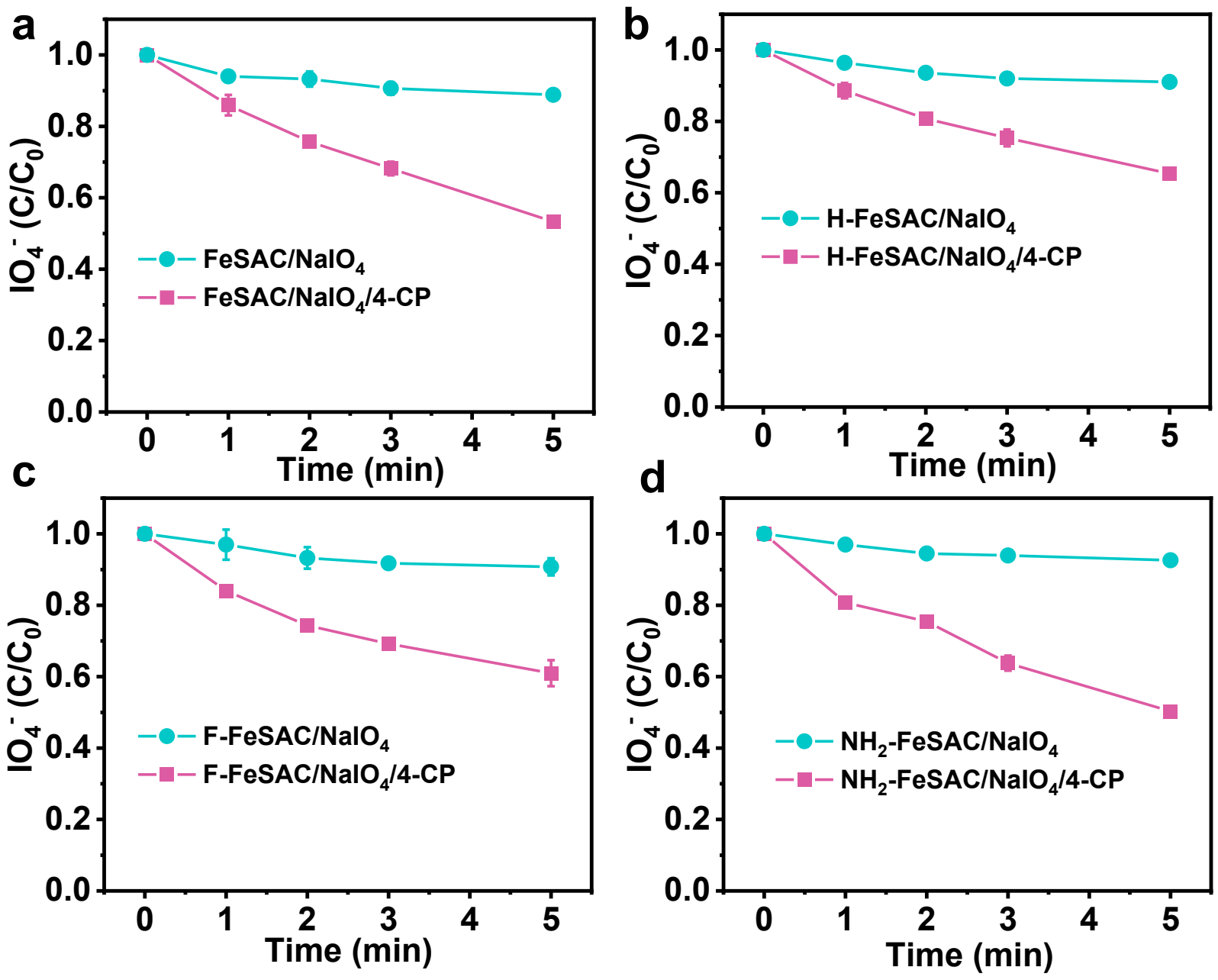

Supplementary Fig. 23 Decomposition rate of PI in (a) FeSAC/PI, (b) H-FeSAC/PI, (c) F-FeSAC/PI and (d) NH_2-FeSAC/PI system.

These results show clear differences in IO_4^- utilization efficiency among FeSACs with different peripheral substituents (**Fig. R15**). The calculated slopes are 1.193, 1.047, and 0.577 (mol 4-CP removal per mol IO_4^- consumed) for F-FeSAC, H-FeSAC, and NH_2-FeSAC, respectively. Compared with the pristine FeSAC (0.483), the introduction of peripheral substituents improves the effective utilization of IO_4^-, confirming that beyond-second-coordination-shell regulation can promote the coupling between PI activation and pollutant oxidation. Moreover, the different utilization efficiencies of F-FeSAC and NH_2-FeSAC indicate that the electronic nature of the peripheral substituent plays a critical role in governing IO_4^- activation efficiency. In particular, the electron-withdrawing F substituent gives the highest IO_4^- utilization efficiency, whereas the electron-donating NH_2 substituent shows a relatively lower value. This behavior can be rationalized by the remote electronic modulation of the Fe single-atom sites by the peripheral substituents. As shown by the DFT results (**Fig. 4d-h**), the electron-withdrawing F substituent upshifts the Fe *d*-band center, optimizes the frontier Fe 3*d* orbital distribution near the Fermi level, and strengthens the interaction between the Fe site and adsorbed PI. These electronic effects facilitate the formation of catalytically active FeSAC/PI* intermediates and promote interfacial electron transfer, thereby accounting for the superior IO_4^- utilization efficiency and 4-CP oxidation performance of F-FeSAC.

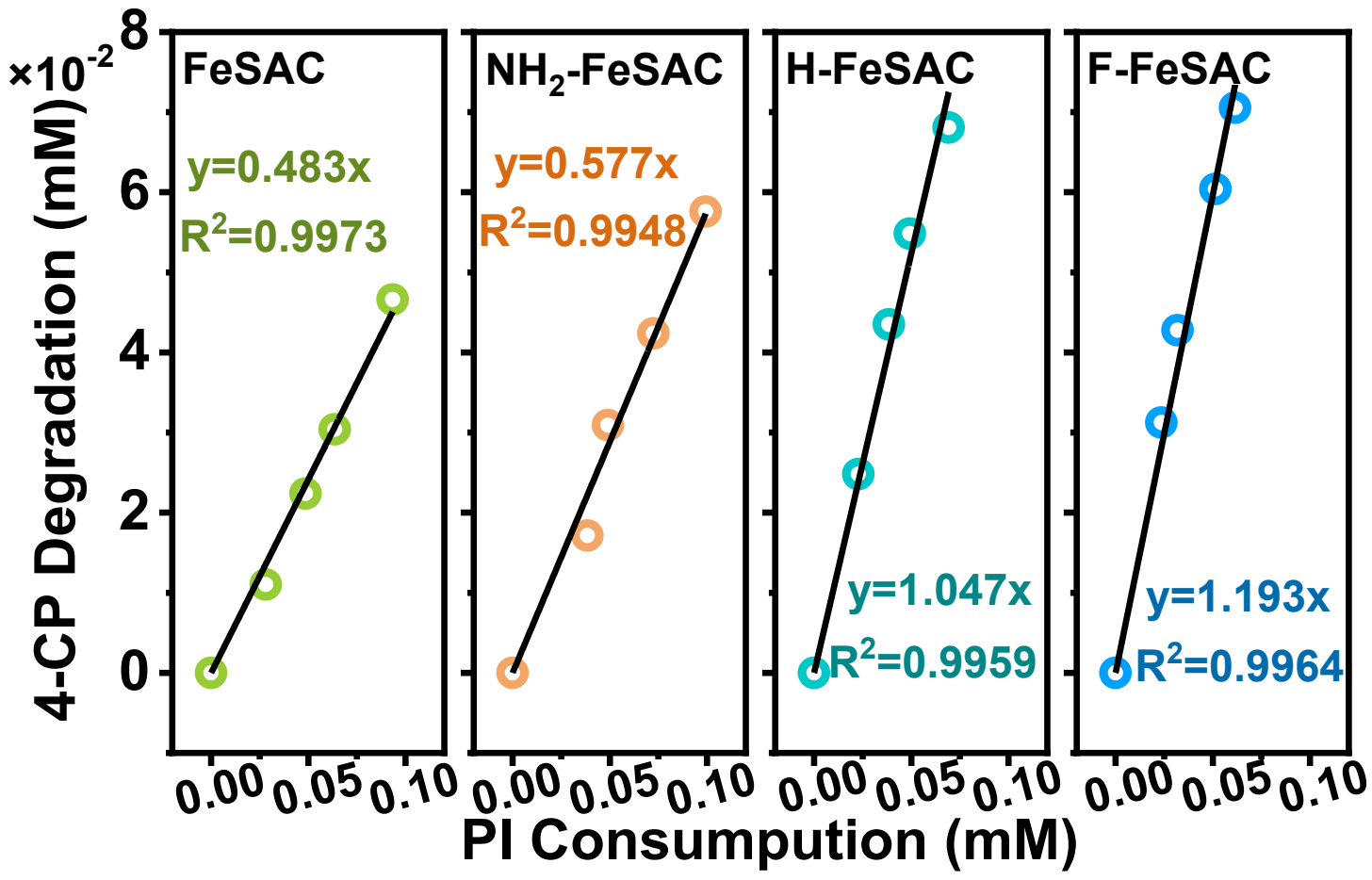

Fig. R15 Correlation between 4-CP degradation and PI consumption in FeSAC/PI, NH_2-FeSAC/PI, H-FeSAC/PI and F-FeSAC/PI systems.

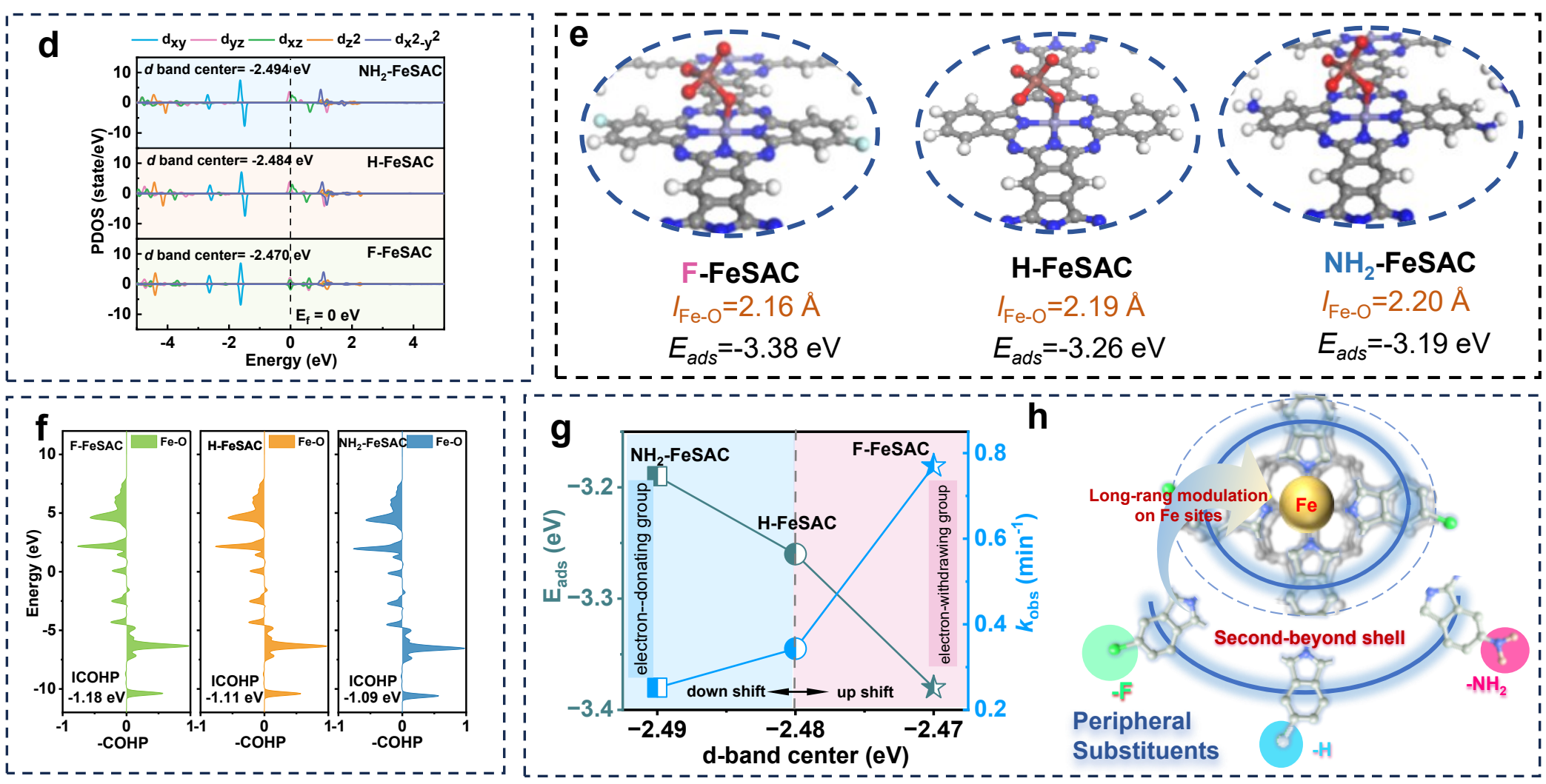

Fig. 4 (d) Projected Density of States of the Fe 3*d* orbitals in F-FeSAC, H-FeSAC, and NH_2-FeSAC. (e) Optimized adsorption configurations of PI at Fe site on the F-FeSAC, H-FeSAC, and NH_2-FeSAC. (f) Crystal orbital Hamilton population (COHP) analysis of Fe-O when PI adsorbs on Fe. (g) Correlations among k_{obs}, E_{ads}, and *d*-band center for F-FeSAC, H-FeSAC, and NH_2-FeSAC. (h) Schematic illustration of long-range electronic modulation at Fe sites induced by substituent groups beyond the secondary coordination sphere.

Action 8: We have added the calculated results of IO_4^- utilization efficiency in Fig. 3e. And the corresponding discussion have been added to the revised manuscript.

*"A strong linear correlation between 4-CP removal and IO_4^- depletion was also observed (**Fig. 3e**), indicating the concerted degradation of the pollutant and*

decomposition of the oxidant. The corresponding slope was highest for F-FeSAC, indicating the most efficient coupling between IO_4^- consumption and 4-CP oxidation."

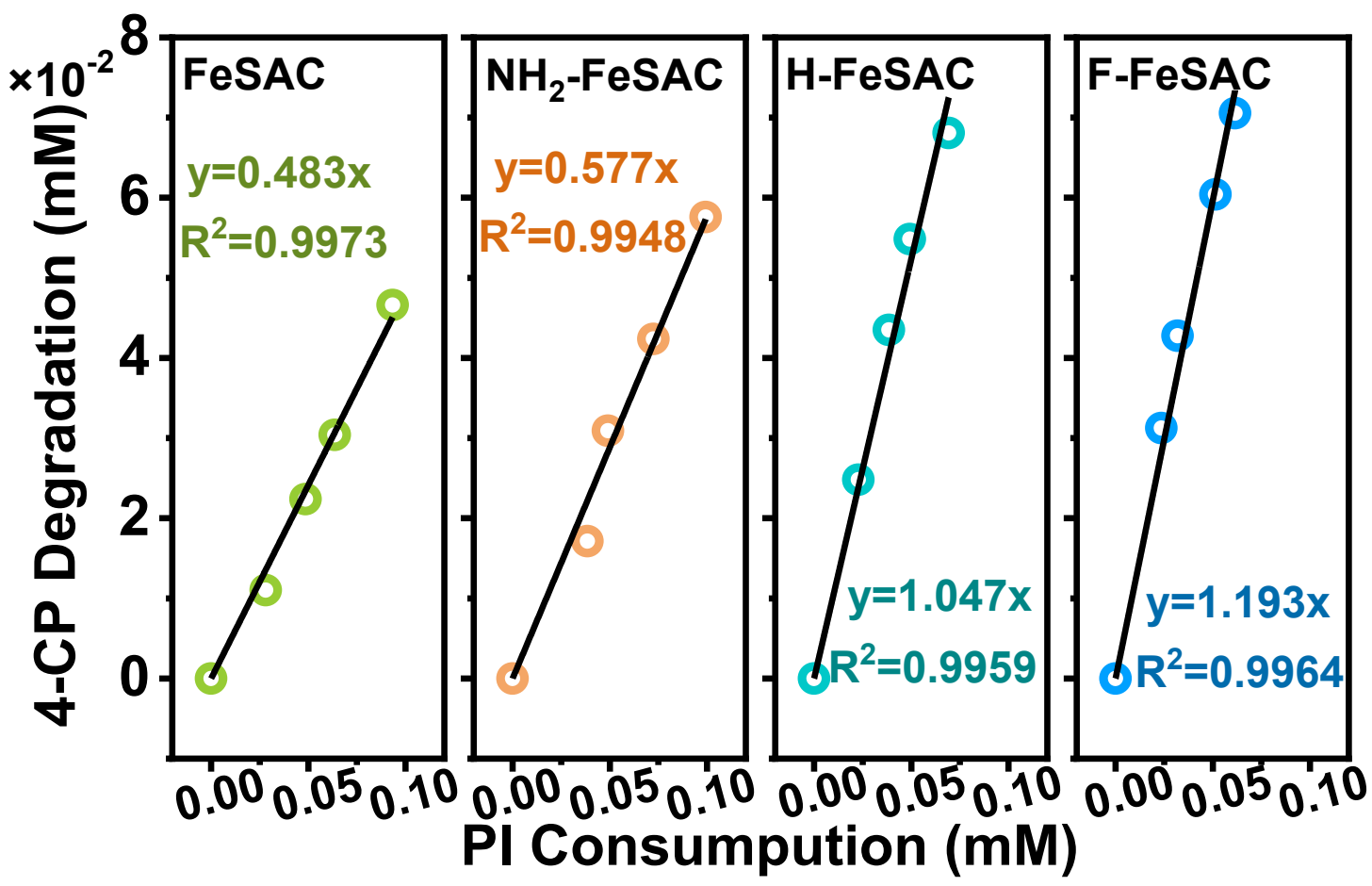

Fig. 3 (e) Correlation between 4-CP degradation and PI consumption in FeSAC/PI, NH_2-FeSAC/PI, H-FeSAC/PI and F-FeSAC/PI systems.

9. Only the signal of $^1O_2$ was detected in the EPR experiments, and no signals of free radicals such as ·OH and O2·⁻ were detected. The authors are requested to supplement EPR data (e.g., DMPO trapping ·OH/O2·⁻) to further confirm that the system is dominated by non-radicals.

Response 9:

We thank the reviewer for this valuable comment. To further clarify whether common free radicals are directly involved in 4-CP degradation, we supplemented the EPR measurements using DMPO as the spin-trapping agent.

Following the reviewer's suggestion, we have now supplemented the EPR experiments using DMPO as the spin-trapping agent to probe possible $IO_3^•$, •OH and $O_2^{•-}$ generation in the R-FeSAC/PI systems. As shown in the newly added EPR data, no characteristic signal of DMPO-$O_2^{•-}$ or DMPO-$IO_3^•$ were detected (**Fig. R16-R17**), whereas weak signals assignable to DMPO-•OH were observed in the R-FeSAC/PI systems. More importantly, the addition of 4-CP did not induce any obvious change in these signals (**Fig. R18**). This result suggests that, although these radical species may be present in the reaction system, they are not directly involved in the oxidation of 4-CP.

This interpretation is consistent with the scavenger experiments and the NBT results (**Supplementary Figs. 12-13**). TBA and $CHCl_3$ caused only limited inhibition, and the NBT assay did not support a meaningful involvement of $O_2^{•-}$. We also examined the

possible role of $IO_3^{\bullet}$ by using 2,4,6-TCP as a probe substrate. Since all FeSAC/PI and R-FeSAC/PI systems still showed efficient degradation of 2,4,6-TCP, a dominant contribution from $IO_3^{\bullet}$ is also unlikely (**Supplementary Fig. 17**).

Taken together, these results suggest that conventional radical species, including •OH, $O_2^{\bullet-}$, and $IO_3^{\bullet}$, are not the key factors governing 4-CP degradation in the present system. The oxidation process is therefore more reasonably attributed to a non-radical pathway.

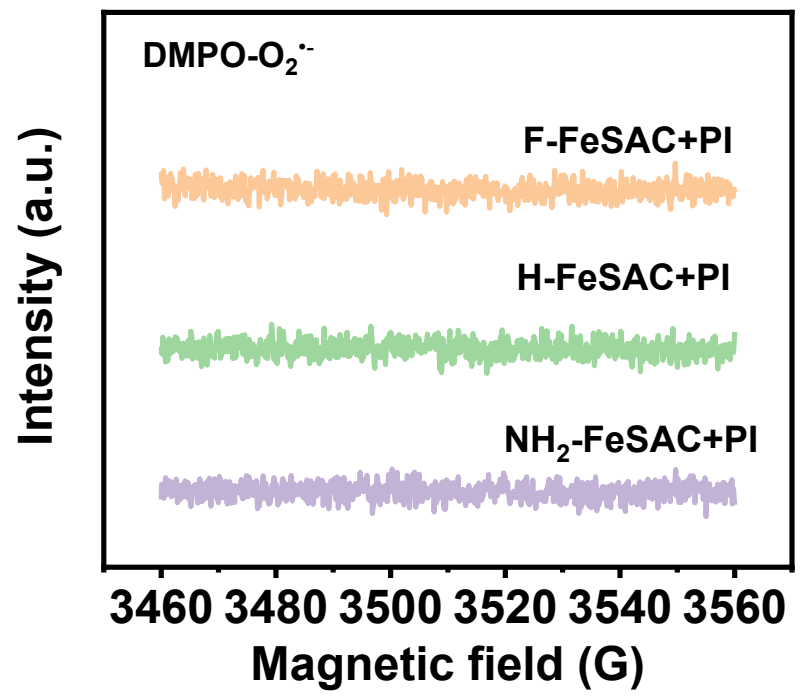

Fig. R16 EPR spectra of $O_2^{\bullet-}$ trapped by DMPO in F-FeSAC/PI, H-FeSAC/PI, and NH_2-FeSAC/PI system.

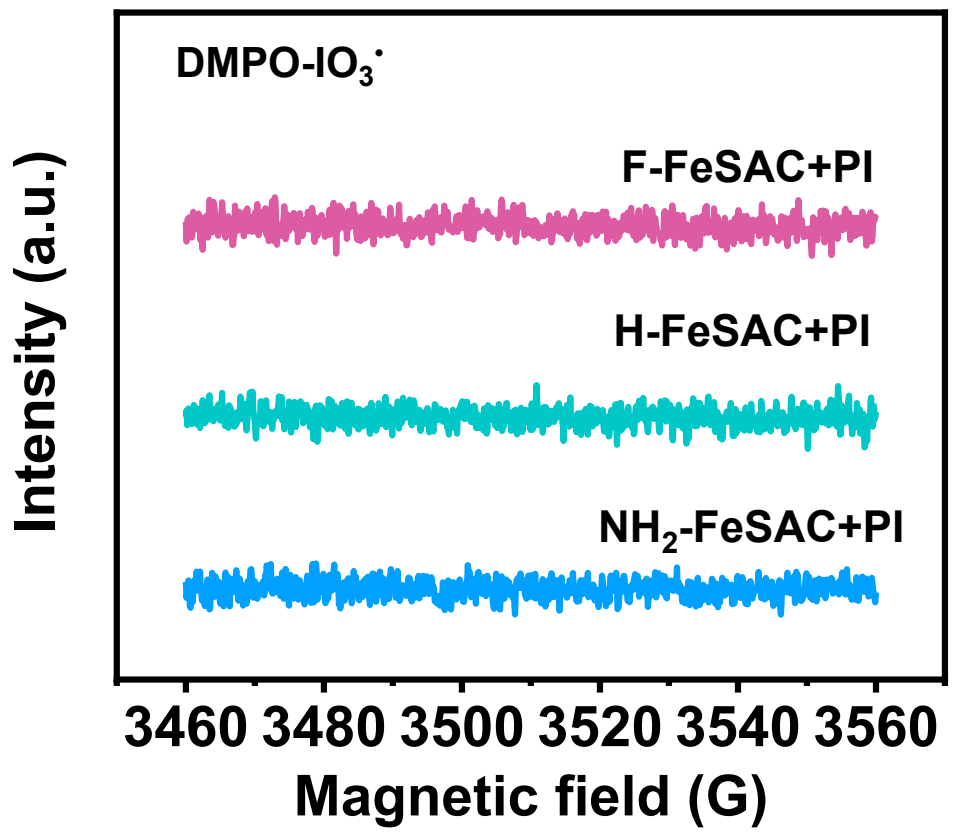

Fig. R17 EPR spectra of $IO_3^{\bullet}$ trapped by DMPO in F-FeSAC/PI, H-FeSAC/PI, and NH_2-FeSAC/PI system.

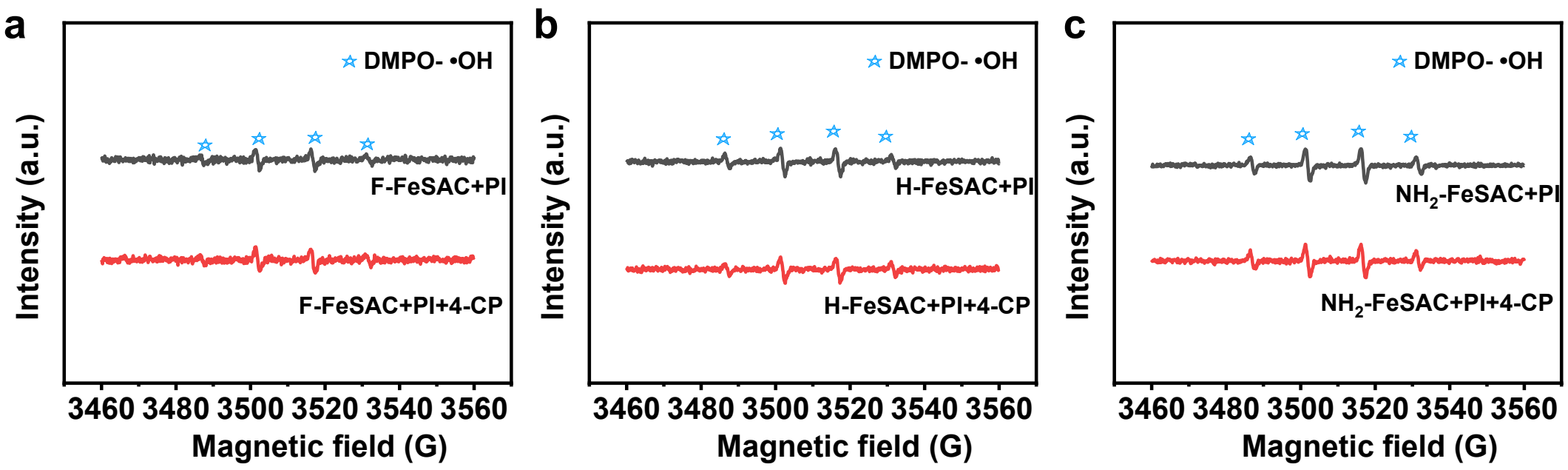

Fig. R18 EPR spectra of •OH trapped by DMPO in (a) F-FeSAC/PI, (b) H-FeSAC/PI, and (c) NH_2-FeSAC/PI system.

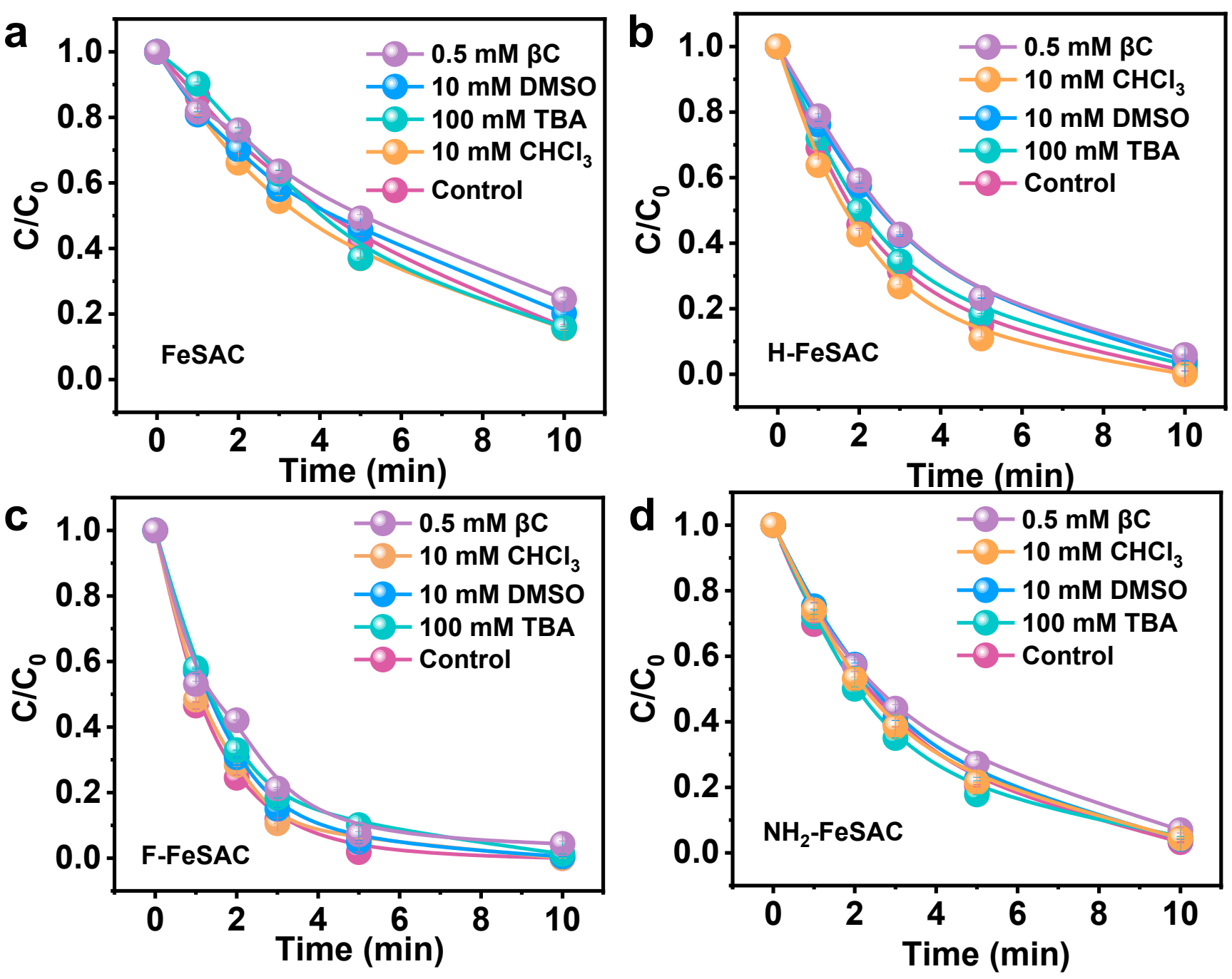

Supplementary Fig. 12 Degradation of 4-CP in (a) FeSAC/PI, (b) H-FeSAC/PI, (c) F-FeSAC/PI and (d) NH_2-FeSAC/PI system under different quenchers. Routine conditions: [4-CP] = 80 μM, [catalyst] = 50 mg/L, [PI] = 0.20 mM, temperature = 25 °C, without pH adjustment.

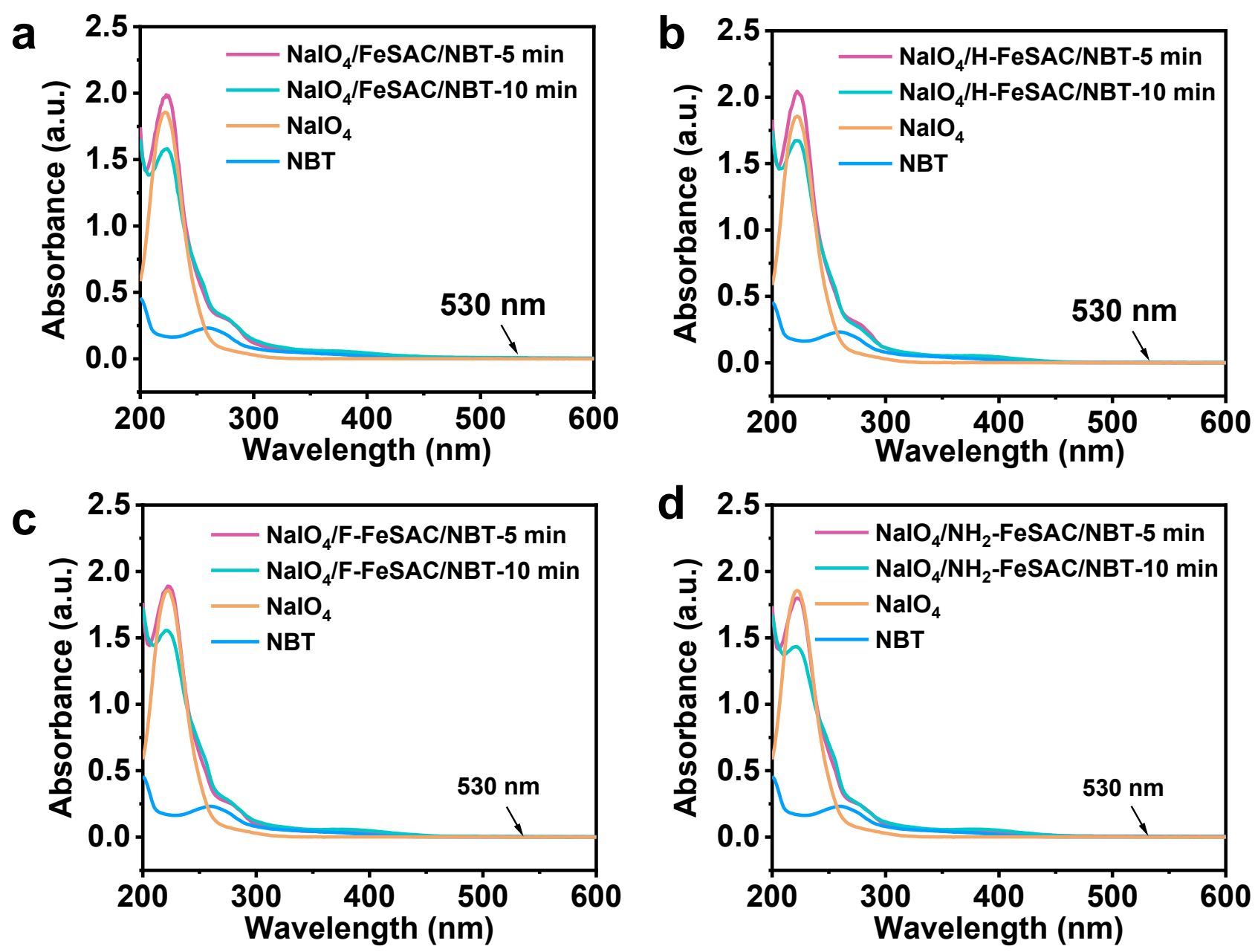

Supplementary Fig. 13 UV-vis absorption spectra of NBT in the (a) FeSAC/PI, (b) H-FeSAC/PI, (c) F-FeSAC/PI and (d) NH_2-FeSAC/PI system.

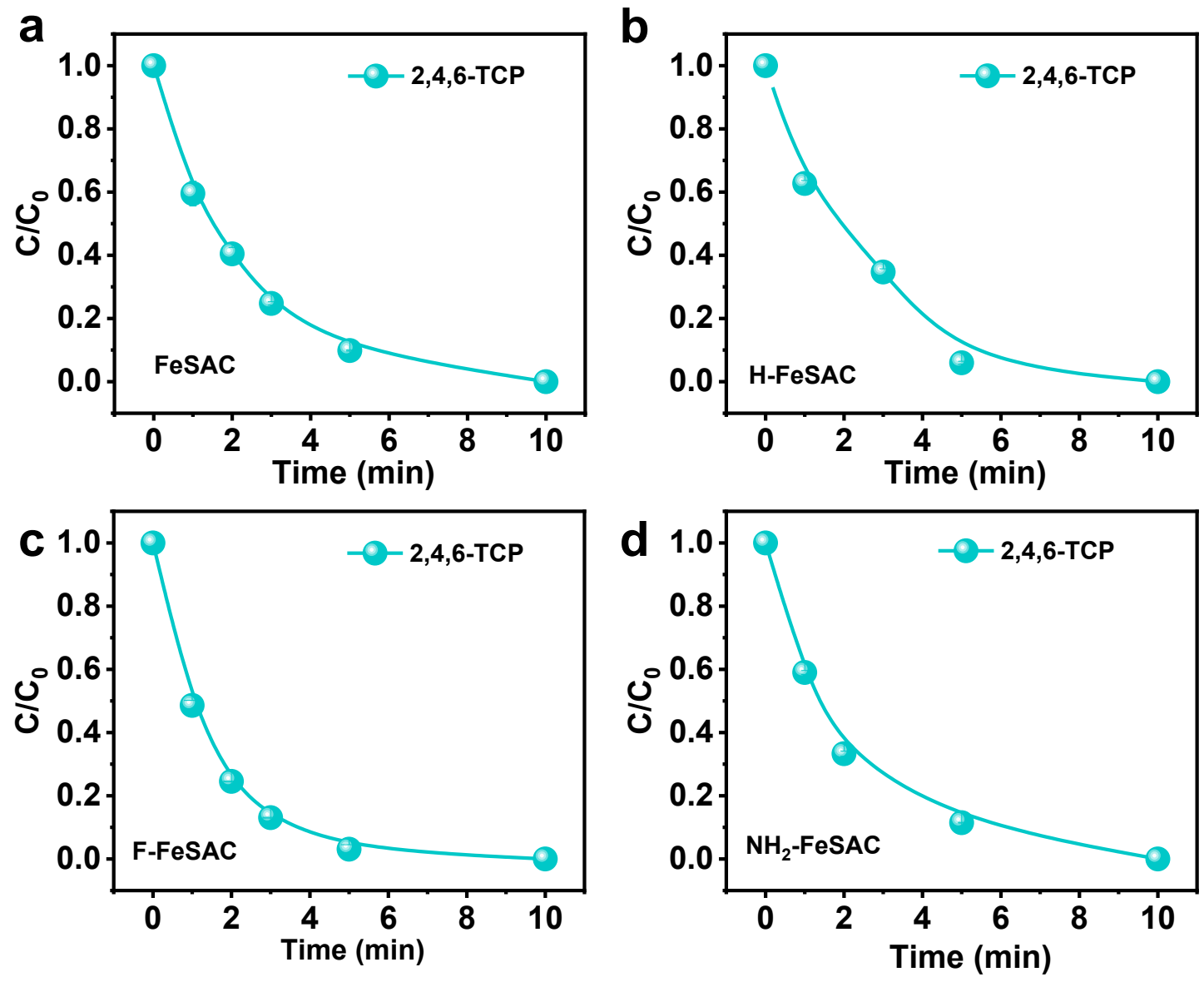

Supplementary Fig. 17 Degradation of 2,4,6-TCP in the (a) FeSAC/PI, (b) H-FeSAC/PI, (c) F-FeSAC/PI and (d) NH_2-FeSAC/PI system.

Action 9: The DMPO spin-trapping EPR data for $IO_3^{\bullet}$, •OH and $O_2^{\bullet-}$ have now been added to the revised Supplementary Information as **Supplementary Fig. 14-16**, and the relevant discussion has been updated accordingly.

"Additional EPR spin-trapping experiments using DMPO were conducted to examine the possible formation of radical species in the R-FeSAC/PI systems. As shown in ***Supplementary Fig. 14 and 15****, no detectable DMPO-$O_2^{\bullet-}$ (α_N = 14.3 G, α_H^{β} = 11.4 G, α_H^{γ} = 1.2 G) or DMPO-$IO_3^{\bullet}$ (α_H = 12.9 G, α_N = 14.2 G) signals were observed, excluding $O_2^{\bullet-}$ and $IO_3^{\bullet}$ as the dominant radical species in these systems. Although weak DMPO-•OH signals ($\alpha_N = \alpha_H$ = 14.9 G) were detected, their spectral profiles and intensities remained essentially unchanged after 4-CP addition, suggesting that •OH was not directly involved in 4-CP oxidation (****Supplementary Fig. 16****). Therefore, these results further support that radical oxidation is not the dominant pathway for 4-CP degradation in these systems."*

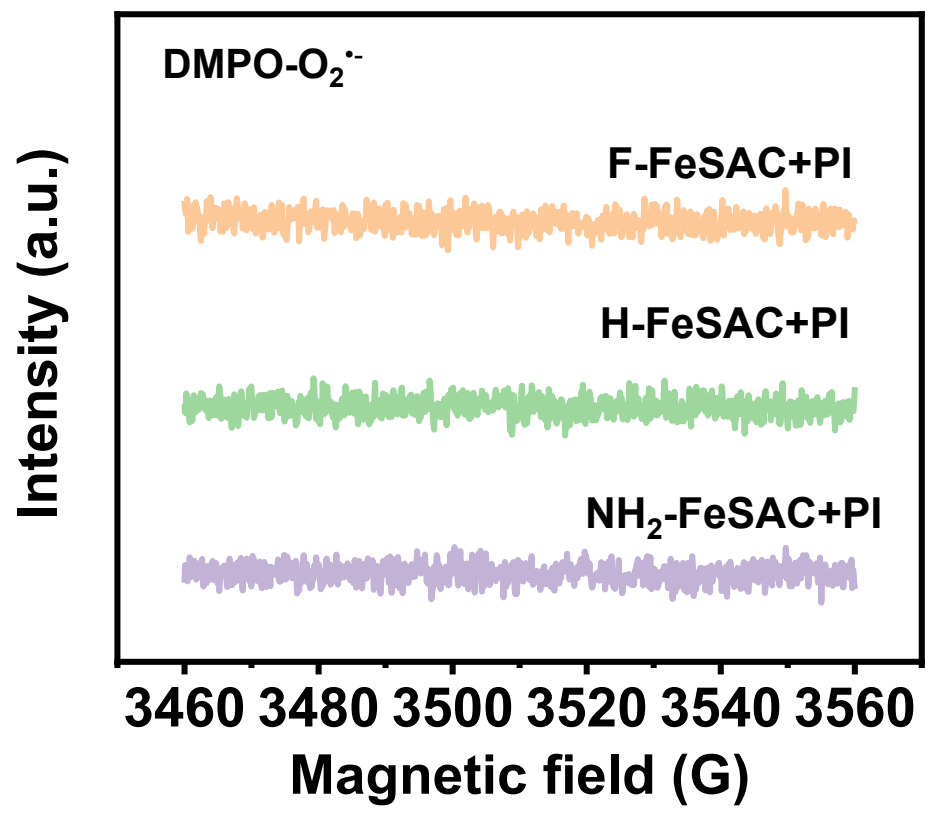

Supplementary Fig. 14 EPR spectra of $O_2^{•-}$ trapped by DMPO in F-FeSAC/PI, H-FeSAC/PI, and NH_2-FeSAC/PI system.

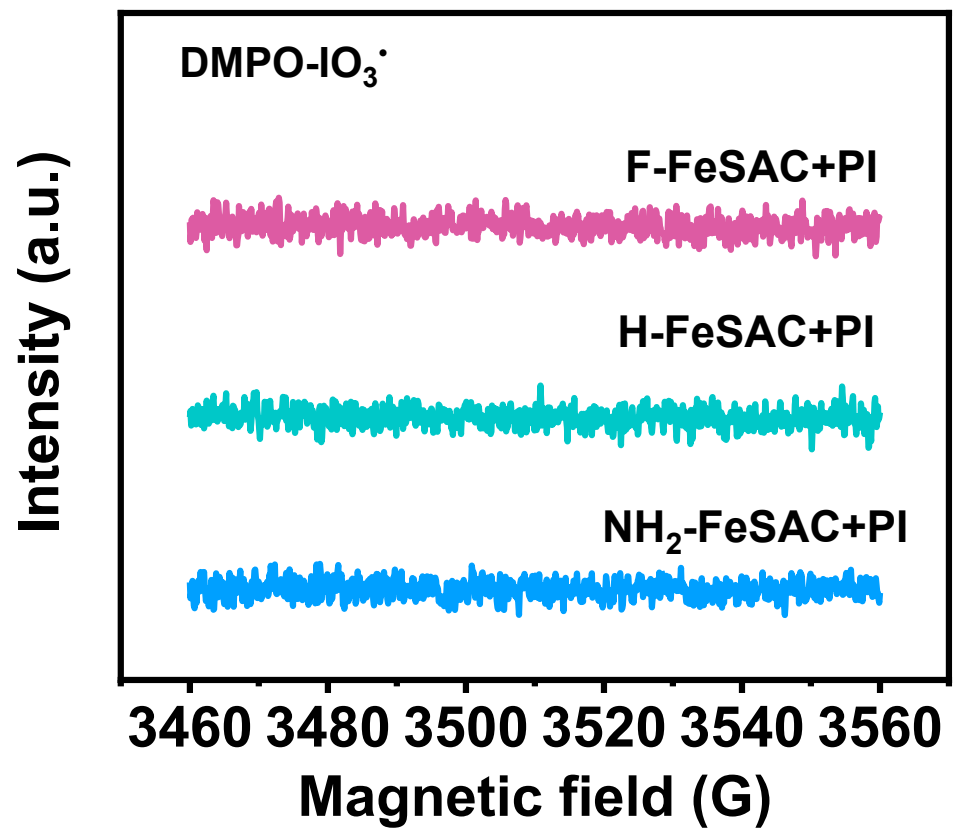

Supplementary Fig. 15 EPR spectra of $IO_3^•$ trapped by DMPO in F-FeSAC/PI, H-FeSAC/PI, and NH_2-FeSAC/PI system.

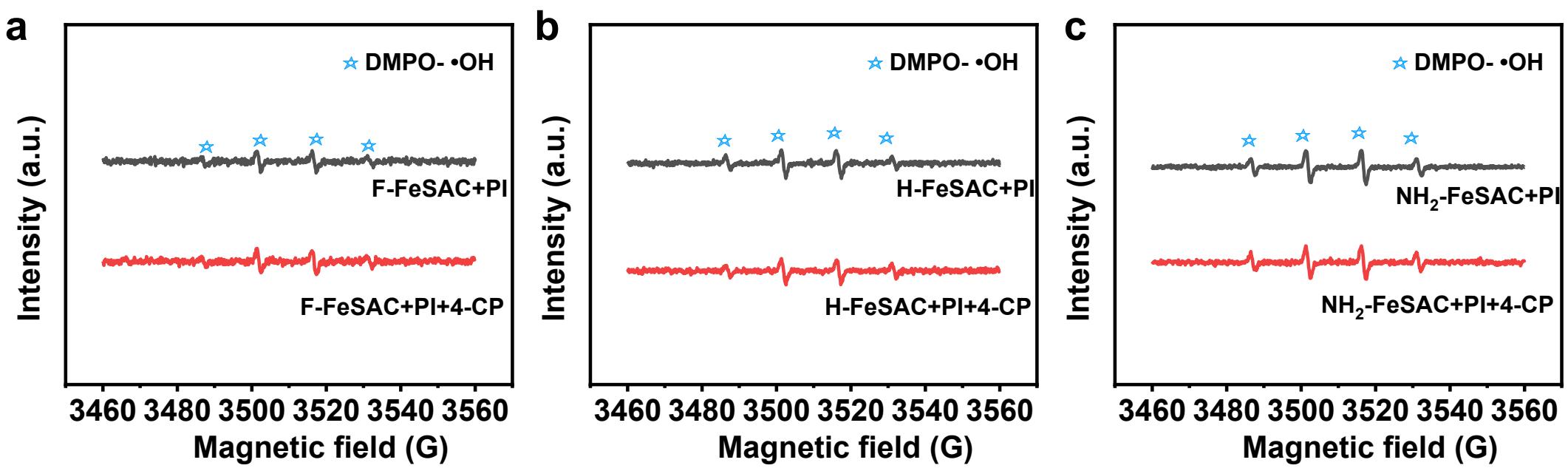

Supplementary Fig. 16 EPR spectra of •OH trapped by DMPO in (a) F-FeSAC/PI, (b) H-FeSAC/PI, and (c) NH_2-FeSAC/PI system.

10. Fig S26. Dichlorination products of 4-CP were observed. The ETP system promotes electron loss from 4-CP, corresponding to the oxidation of 4-CP. In this context, what is the mechanism responsible for the reductive dichlorination? In addition, -OH addition products were detected, implying that ·OH contributes to 4-CP degradation.

However, this observation appears inconsistent with the quenching experimental results.

Response 10:

We thank the reviewer for raising this important mechanistic question. We agree that the relationship between the detected products and the proposed electron-transfer pathway should be clarified. The reviewer used the term “dichlorination”, which literally refers to the introduction of two chlorine atoms. However, based on the products shown in Fig. S26, we are not fully certain whether the reviewer intended to refer to “dichlorination” or “dechlorination”. To avoid ambiguity, we have clarified both issues.

First, regarding the dichlorinated products, we would like to clarify that their detection does not necessarily indicate a reductive transformation process. These products are more reasonably assigned to oxidative coupling products derived from multiple 4-CP units, in which the original chlorine substituent on each aromatic unit is retained. This interpretation is consistent with previous studies on halophenol/phenolic oxidation. For example, Cui et al.[56] reported in *Water Research* that phenolic pollutants can undergo periodate-mediated oxidative oligomerization through the coupling of phenoxy-type intermediates. Similarly, Xie et al.[35] showed in *Water Research* that chlorophenols can be transformed through a surface-complex-mediated ETP pathway into coupled/polymeric products rather than through reductive transformation.

Second, if the reviewer intended to refer to dechlorination-related products, we would further clarify that their formation also does not necessarily indicate reductive dechlorination. Although dechlorination is often discussed in the context of reductive processes, oxidative dechlorination of chlorophenols can also occur under oxidative conditions. Reductive dechlorination generally requires reductive species or electron-donating environments, such as hydrated electrons, hydrogen atoms, or cathodic electrons. In the present F-FeSAC/PI system, we did not obtain evidence for such reductive species. Instead, the overall reaction environment is oxidative. Specifically, ETP promotes electron loss from 4-CP, generating phenoxy-radical- or radical-cation-like intermediates. Owing to the p–π conjugation of phenolic compounds, the electron-deficient character can be delocalized over the aromatic ring. When this electron-deficient character is localized at or near the carbon atom bearing the Cl substituent, the C–Cl bond may become more susceptible to activation and subsequent cleavage, followed by chloride release and further oxidative transformation (**Fig. R8**). This interpretation is supported by previous studies. For example, Xie et al.[35] showed in *Water Research* that chlorophenols can undergo oxidative dechlorination through the

formation of quinone-/semiquinone-type intermediates or hydroxylated products, accompanied by C–Cl bond cleavage and chloride release. Therefore, the dechlorination-related products detected in our system are more reasonably attributed to secondary transformations of ETP-generated oxidized intermediates rather than to reductive dechlorination.

Regarding the hydroxylated products, we agree with the reviewer that such products are commonly associated with ·OH attack in radical-dominated advanced oxidation processes. However, the detection of hydroxylated products alone does not necessarily demonstrate that free ·OH makes a major contribution to 4-CP degradation. In our system, both quenching and EPR results suggest that free ·OH is unlikely to be the dominant oxidizing species. More importantly, hydroxyl incorporation may also occur through pathways other than direct free-·OH attack, especially in heterogeneous oxidation systems. Previous heterogeneous oxidation studies reported in *Water Research*[56] have shown that hydroxyl substitution and coupling reactions are common transformation routes for chlorophenols under ETP-mediated oxidative conditions. In our F-FeSAC/PI system, ETP-generated electron-deficient intermediates may undergo secondary reactions with H_2O /HO^- or oxygen-containing oxidizing species, leading to hydroxyl substitution, and the formation of hydroxylated products. Thus, the detection of hydroxylated products does not necessarily indicate a dominant free-·OH pathway, but can also be rationalized as secondary transformation of ETP-generated oxidized intermediates.

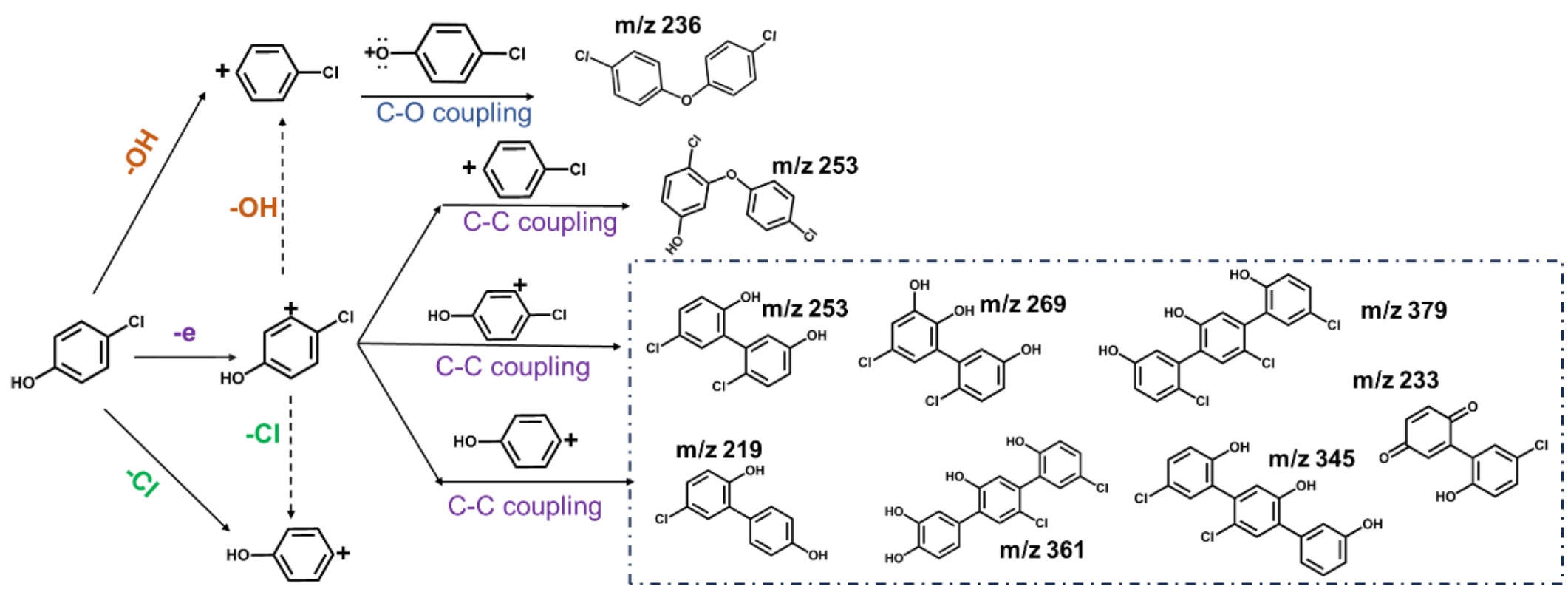

Fig. R8 Possible transformation pathways of 4-CP in F-FeSAC/PI.

Action 10: We have the added relevant statements in the revised manuscript and Supplementary Figs. 34-35 have been added in the supporting information.

"Characterization of the accumulated surface species provided mechanistic insight. Liquid chromatography-mass spectrometry (LC-MS) analysis of the solution following desorption identified chlorinated oligomers as the main surface-adsorbed products

*formed during 4-CP oxidation (**Supplementary Figs. 34-35**). Their formation supports a polymerization-mediated electron-transfer pathway. Specifically, 4-CP is first oxidized through interfacial electron transfer to generate phenoxy- or phenoxonium-type intermediates. These intermediates are resonance-stabilized over the oxygen atom and the ortho/para positions of the aromatic ring, making them prone to intermolecular C–C and C–O coupling. Repeated coupling of these surface-generated intermediates then leads to chlorinated dimers, trimers, and higher oligomeric products. Owing to their low solubility and strong interfacial affinity, these oligomeric products preferentially accumulate on the catalyst surface, leading to progressive activity attenuation during cycling."*

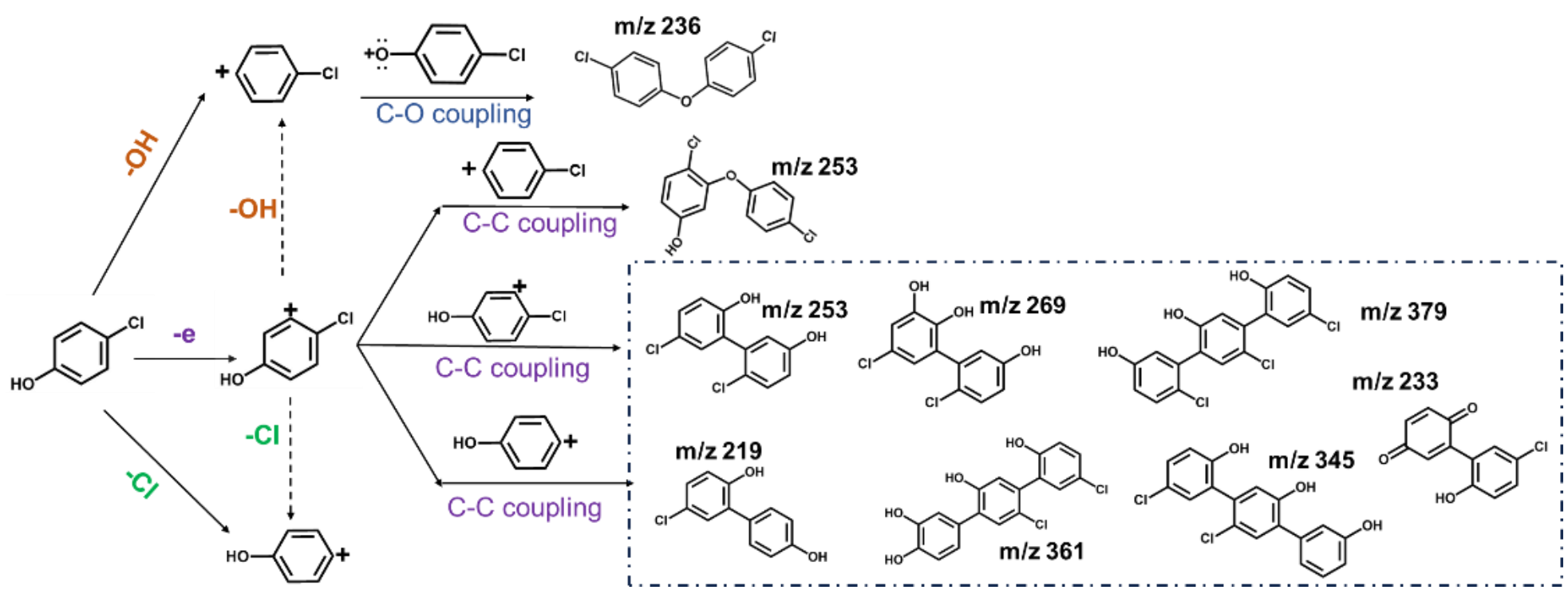

Supplementary Fig. 34 Possible transformation pathways of 4-CP in F-FeSAC/PI.

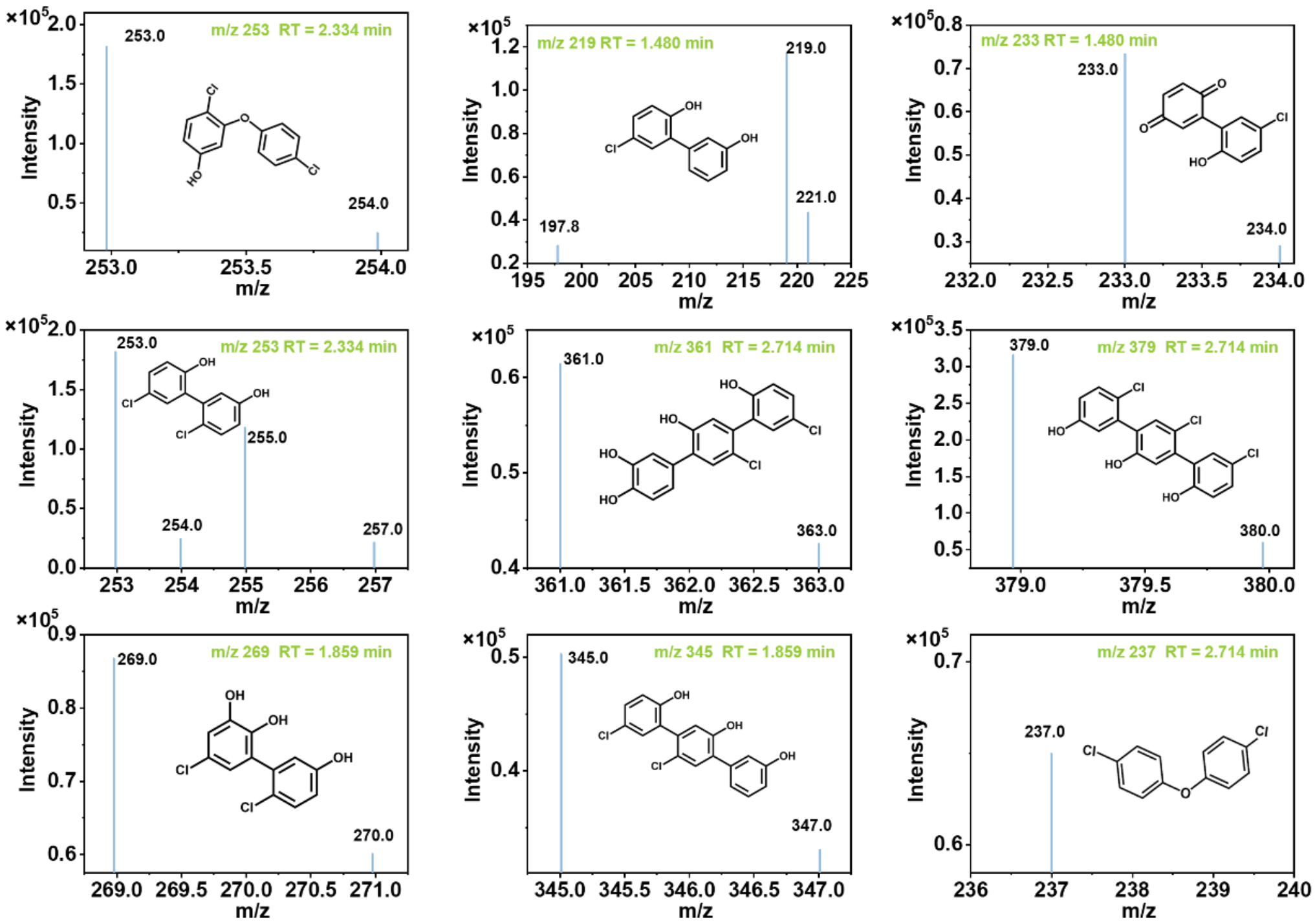

Supplementary Fig. 35 ESI mass spectra of 4-CP during reaction in F-FeSAC/PI system.

Reviewer #3 (Remarks to the Author):

This manuscript presents a strategic mechanism for the simultaneous utilization of 4-CP and periodate (PI) in wastewater treatment using Fe-N-C SAC catalysts. The study provides a comprehensive evaluation, including zebrafish-based toxicity assessments, compatibility tests with various pollutants, and DFT-level orbital analysis. However, the mechanistic discussion relies on indirect negative evidence, and more robust empirical support is required to substantiate the proposed conclusions.

1. While studies on the second and third coordination shells exist in various catalytic fields, the authors also emphasized these long-range interactions. Please clarify why long-range interactions were prioritized over first-shell modifications, which typically exert stronger electronic control. Was first-shell modulation feasible with the chosen synthetic route? Please provide more explicit reasons for why these reagents were chosen to target long-range effects.

Response 1:

We thank the reviewer for this insightful comment. We agree that first-shell coordination generally exerts the most direct electronic influence on single-atom catalytic centers. Our intention was not to argue that long-range interactions are more important than first-shell coordination. Rather, we aimed to address a less explored but mechanistically important question: whether peripheral substituents beyond the second coordination sphere can remotely modulate an otherwise preserved Fe–N_4 single-atom site and thereby influence PI adsorption, interfacial electron transfer, and catalytic performance.

In single-atom catalysis, the activity of an atomically dispersed metal center is collectively determined by multiple coordination layers, including the first coordination shell, the second coordination shell, and more distal coordination environments beyond the second shell. First-shell modulation directly changes the metal-ligand bonding configuration and therefore often produces strong electronic effects. This is why most previous studies have focused on first-shell M–N_x coordination, heteroatom substitution close to the metal center, or second-shell neighboring atoms/defects. However, precise regulation becomes increasingly difficult as the targeted coordination environment moves farther away from the metal center. For higher-shell or distal environments, it is challenging to introduce well-defined structural variations while keeping the primary metal coordination motif unchanged. Consequently, clear structure-effect relationships associated with long-range interactions remain much less

established in artificial SAC systems.

Nevertheless, long-range interactions are important for catalytic regulation. Natural metalloenzymes provide a representative example: their high activity and selectivity are governed not only by the metal center and its directly coordinated ligands, but also by distal residues and binding pockets that remotely tune the electronic and steric environment of the active site. This distal-pocket-like regulation has long been recognized as an important principle in enzyme catalysis, but it remains difficult to implement in synthetic SACs, particularly in conventional pyrolysis-derived M–N–C materials, where remote functional groups, coordination structures, defects, and local carbon environments are often uncontrollable under high-temperature conditions.

Our non-pyrolytic conjugated organic SAC platform was designed to overcome this limitation. The R-FeSAC system preserves a similar primary Fe–N_4 coordination structure while introducing tunable peripheral substituents (R = F, H, NH_2) beyond the second coordination sphere (**Fig. 1a-b**). This design allows us to decouple remote substituent effects from first-shell coordination changes and to examine whether long-range electronic perturbation alone can tune the Fe center, PI adsorption, and interfacial electron-transfer kinetics. Therefore, the emphasis on long-range interactions reflects a controlled mechanistic design rather than a preference over first-shell modification.

To the best of our knowledge, this is the first report to identify a peripheral-substituent-governed PI activation mechanism in which distal electronic tuning modulates and enhances an electron-transfer-driven polymerization pathway in SAC catalysis. This work expands the design space of SACs beyond the widely studied M–N_4 motif and establishes a new structure-property relationship relevant to PI activation.

Whether first-shell modulation was feasible with this route

Regarding the feasibility of first-shell modulation, the metal center can in principle be changed in this type of framework. However, within the specific synthetic design used in this study, the Fe–N_4 coordination template is predetermined by the polyphthalocyanine-like macrocyclic structure (**Fig. R19**). Directly modifying the first coordination shell would require changing the metal-binding motif, introducing different coordinating atoms into the macrocycle, or applying post-synthetic treatments. These approaches would inevitably introduce additional structural variables, such as altered Fe coordination number, Fe-ligand bond length, local defects, or framework disorder. Such changes would make it difficult to unambiguously attribute the observed catalytic differences to peripheral substituent effects. Therefore, we deliberately fixed

the Fe–N_4 first coordination shell and used the R-FeSAC series as a model platform to isolate long-range substituent effects.

Why these reagents were chosen to target long-range effects

The F-, H-, and NH_2-substituted phthalonitrile monomers were chosen for this purpose because they provide a controlled set of peripheral electronic perturbations while maintaining comparable framework and Fe–N_4 coordination structures. The three substituents differ clearly in electronic character: F is electron-withdrawing, H serves as a neutral reference, and NH_2 is electron-donating. Since these groups are located on the peripheral benzene ring rather than directly bound to Fe, they allow us to examine how distal electronic effects are transmitted through the conjugated scaffold to regulate the Fe center. Consistent with this design, XPS and XANES show systematic changes in the Fe electronic state, EXAFS confirms similar Fe–N coordination environments across the R-FeSAC series, and electrochemical/DFT analyses demonstrate that the F substituent remotely shifts the Fe *d*-band center, strengthens PI adsorption, and accelerates interfacial electron transfer.

Therefore, our emphasis on long-range interactions reflects a controlled mechanistic design rather than a preference over first-shell modification. By fixing the Fe–N_4 coordination template and varying only distal peripheral substituents, this work provides a clearer platform to evaluate enzyme-like long-range electronic regulation in artificial single-atom catalysts.

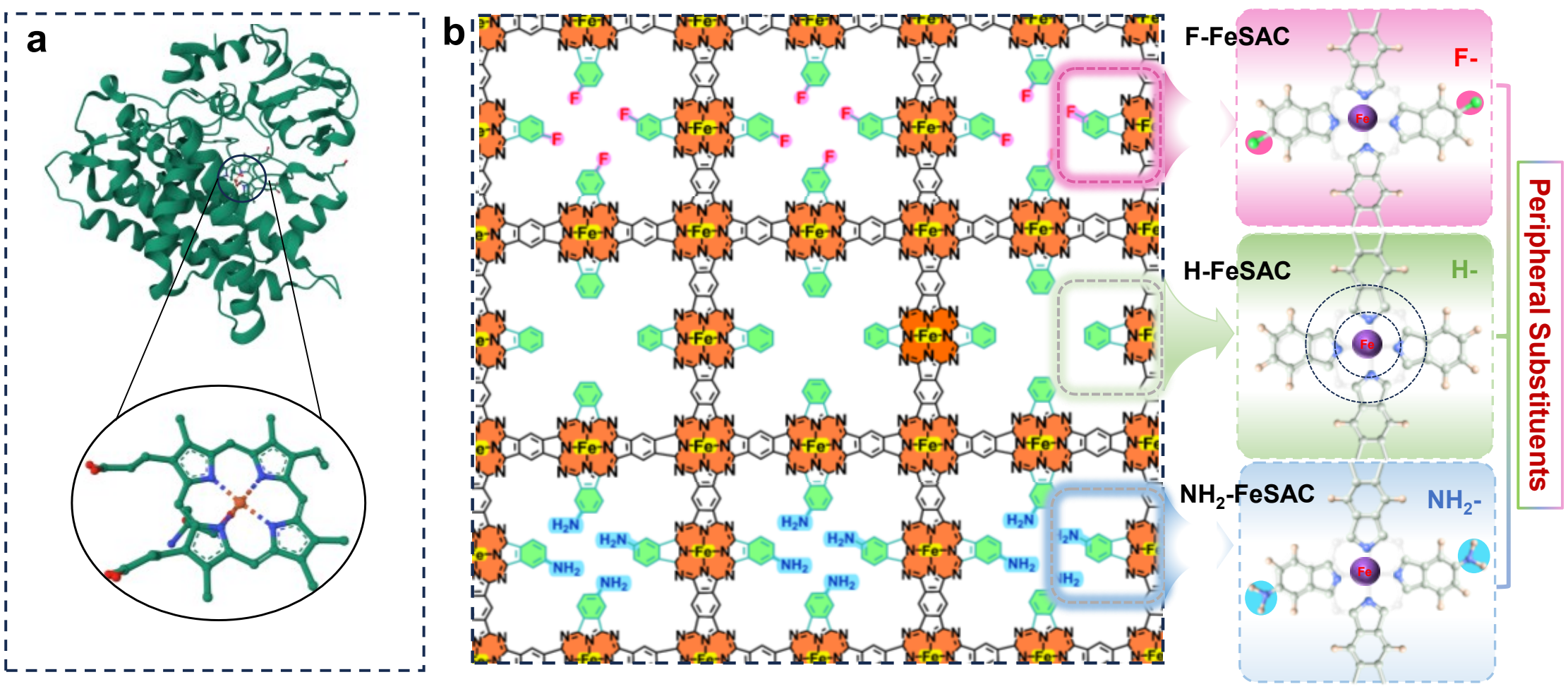

Fig. 1 (a) Model diagram of the Fe active site in the natural metalloenzymes. (b) Schematic of the preparation strategy for R-FeSAC.

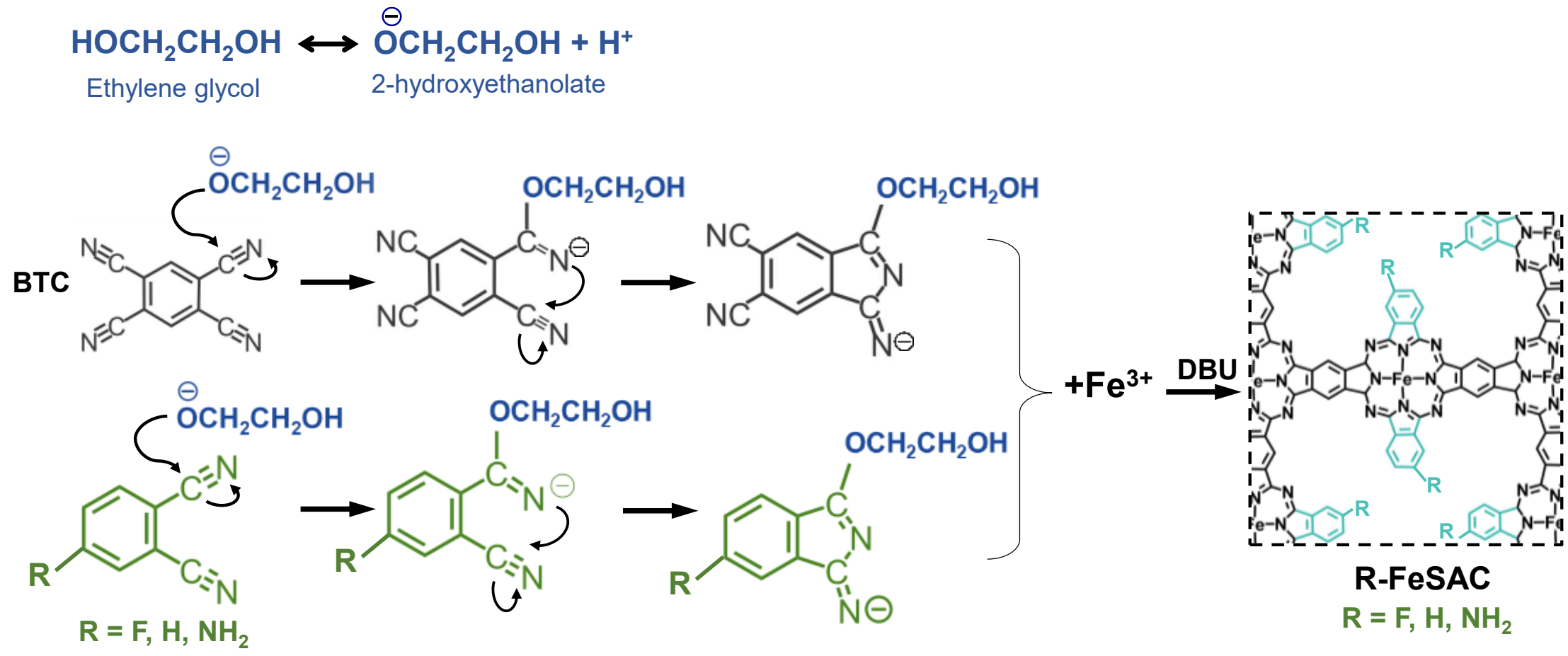

Fig. R19 Formation procedure of R-FeSAC.

2. The stability discussion focuses on the F-FeSAC catalyst, but the structural integrity of the F-C bond is not directly verified post-reaction. Please provide XPS F 1s spectra or ^{19}F solid-state NMR of the spent catalyst to confirm that F substituents remain intact after catalytic cycles.

Response 2:

We thank the reviewer for this important suggestion. To directly verify the structural integrity of the fluorinated substituents after catalysis, we analyzed the spent F-FeSAC using both XPS F 1s and solid-state ^{19}F NMR (**Fig. R20**). The spent catalyst still shows a distinct F 1s signal centered at 687.3 eV, which is characteristic of covalent C–F species. In addition, the solid-state ^{19}F NMR spectrum of the spent catalyst retains the characteristic signal of the fluorinated moiety, consistent with the chemical environment observed for the fresh F-FeSAC. These results together show that the F-containing substituents remained intact during reaction, rather than being lost or transformed under the reaction conditions.

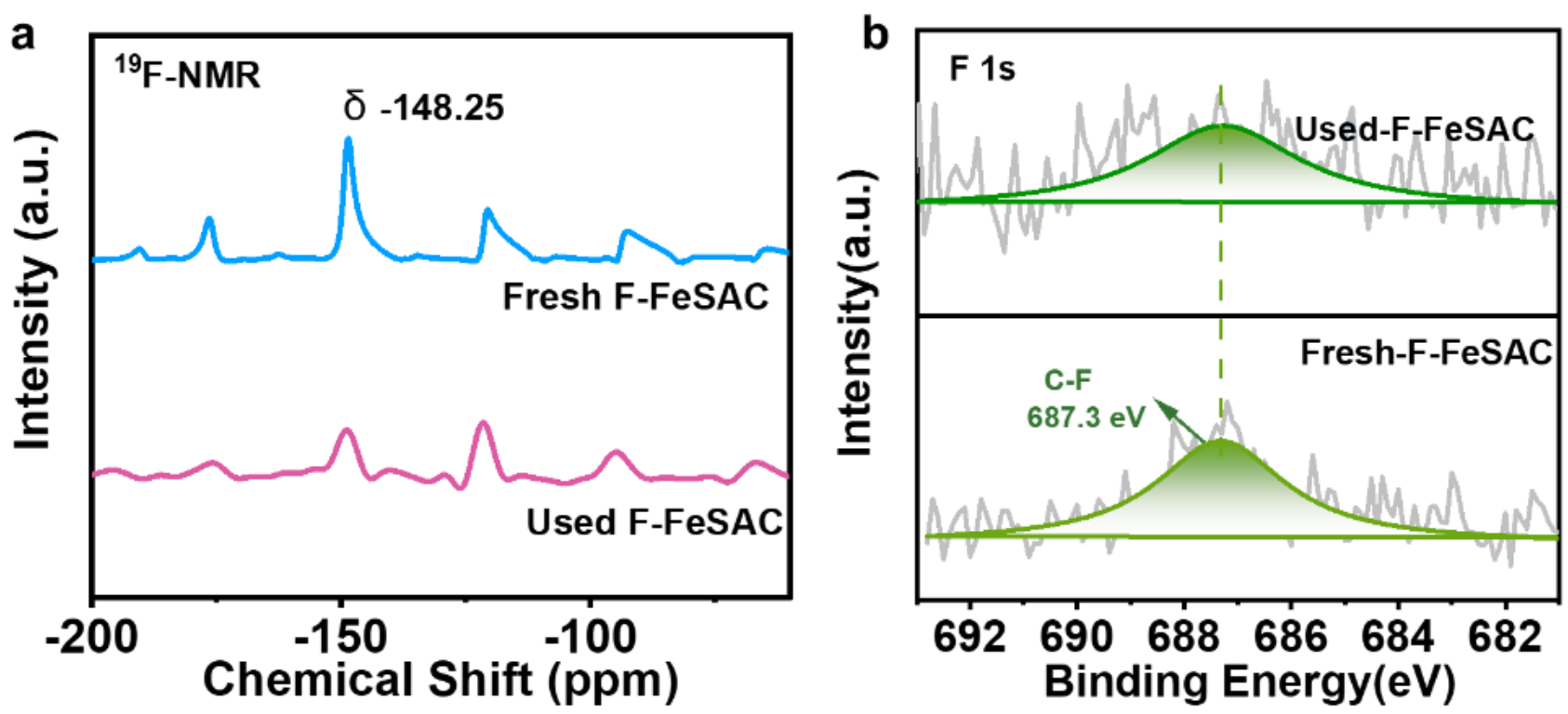

Fig. R20 (a) ^{19}F solid-state NMR spectroscopy and (b) XPS F 1s spectra of Fresh-F-FeSAC and Used-F-FeSAC.

Action 2: The XPS F 1s spectra and solid-state ^{19}F NMR spectra of the fresh and spent F-FeSAC catalysts have now been added to the revised Supplementary Information (Supplementary Fig. 37), and the corresponding discussion has been included in the stability section.

*"Post-reaction XPS F 1s and solid-state ^{19}F NMR analyses further showed that the fluorinated substituents were retained after catalysis (**Supplementary Fig. 37**). The spent F-FeSAC still exhibited a distinct F 1s signal at 687.3 eV, characteristic of covalent C-F species, while the ^{19}F NMR spectrum remained consistent with that of the fresh catalyst. These results, together with the unchanged Fe-N coordination environment, indicate that the observed activity attenuation mainly arose from physical site blocking by deposited oligomeric species rather than catalyst degradation."*

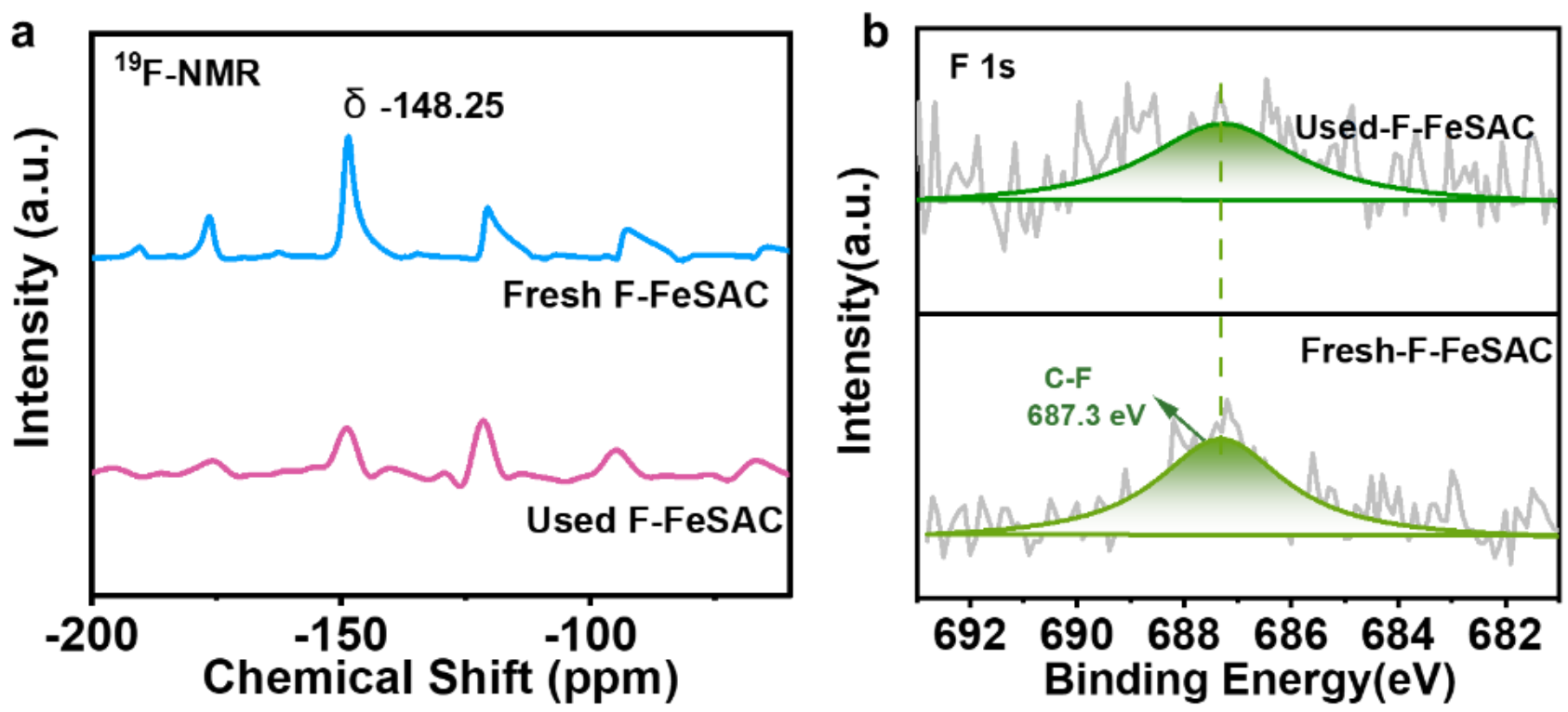

Supplementary Fig. 37 (a) ^{19}F solid-state NMR spectroscopy and (b) XPS F 1s spectra of Fresh-F-FeSAC and Used-F-FeSAC.

3. While similar 2% Fe loading is stated for effective Fe incorporation into the scaffold, what is the theoretical maximum for metal substitution in this scaffold? A quantitative analysis or estimation of the fraction of available sites occupied by Fe would help assess the site utilization efficiency.

Response 3:

We thank the reviewer for this valuable comment. We agree that the theoretical maximum metal substitution and the fraction of occupied sites should be clarified more quantitatively.

The measured Fe contents of the final CNT-containing materials were 2.59 wt.% for F-FeSAC, 2.65 wt.% for H-FeSAC, and 2.41 wt.% for NH_2-FeSAC. However, these values represent the apparent Fe loadings of the hybrid materials as a whole and are diluted by the CNT component. They therefore do not directly reflect the Fe occupation level within the conjugated organic SAC scaffold.

To better estimate the site occupation efficiency of the SAC scaffold itself, we additionally measured the Fe contents of the corresponding CNT-free materials. The Fe contents were 5.29 wt.% for CNT-free F-FeSAC, 5.39 wt.% for CNT-free H-FeSAC, and 5.13 wt.% for CNT-free NH_2-FeSAC. Assuming full Fe occupation of the idealized structural units $C_{72}N_{24}H_{20}Fe_3F_4$, $C_{72}N_{24}H_{24}Fe_3$, and $C_{72}N_{28}H_{28}Fe_3$, the theoretical maximum Fe loadings were calculated to be 11.44 wt.%, 12.03 wt.%, and 11.53 wt.%, respectively. The corresponding estimated site occupation fractions are 46.2% for CNT-free F-FeSAC, 44.8% for CNT-free H-FeSAC, and 44.5% for CNT-free NH_2-FeSAC. The idealized structural units are shown in Fig. R21, and the calculated results are summarized in Table R3.

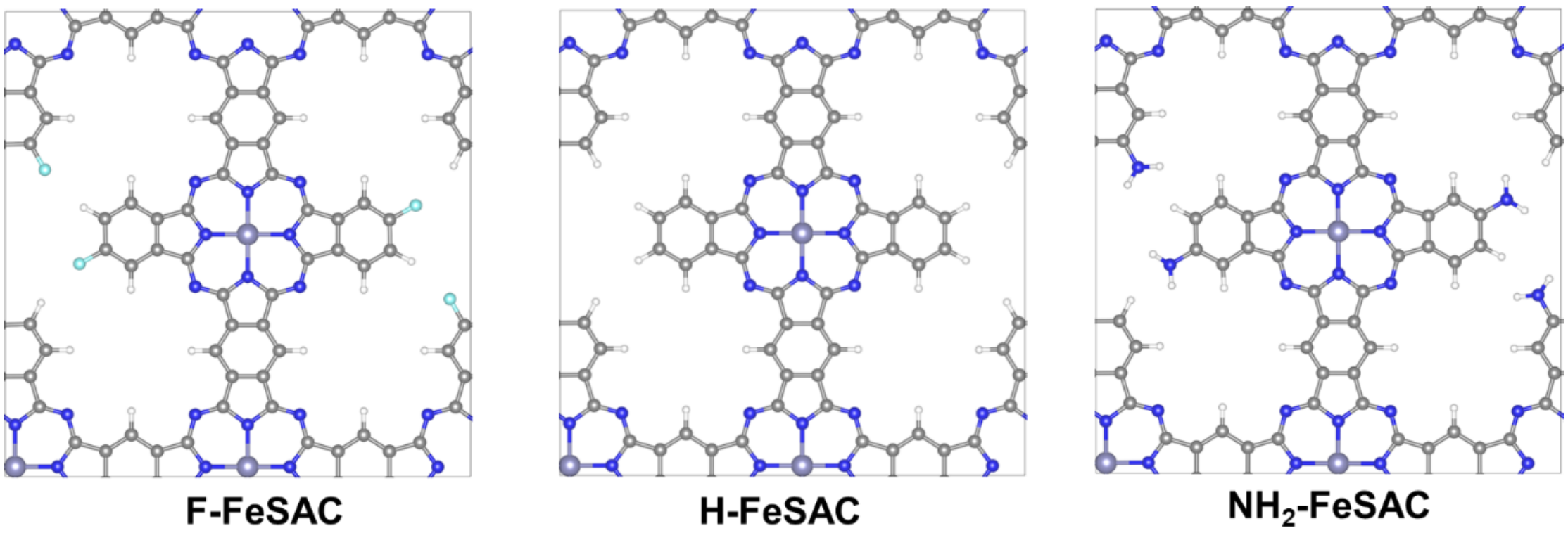

Fig. R21 Idealized structural units of CNT-free F-FeSAC, H-FeSAC, and NH_2-FeSAC.

Table R3 Theoretical maximum Fe loading and estimated Fe site occupation fractions of CNT-free R-FeSAC materials.

Sample	Structural unit	Theoretical Fe loading (wt%)	Fe loading in CNT-free sample (wt%)	**Site occupation (%)**
CNT-free F-FeSAC	$C_{72}N_{24}H_{20}Fe_3F_4$	11.44 wt%	5.29 wt%	**46.2%**
CNT-free H-FeSAC	$C_{72}N_{24}H_{24}Fe_3$	12.03 wt%	5.39 wt%	**44.8%**
CNT-free NH_2-FeSAC	$C_{72}N_{28}H_{28}Fe_3$	11.53 wt%	5.13 wt%	**44.5%**

Note: *The theoretical maximum Fe loading was calculated assuming full Fe occupation of the idealized structural unit. The Fe site occupation fraction was then estimated by dividing the measured Fe loading of the corresponding CNT-free material by this theoretical maximum.*

4. The logic for comparing this system to CNT-based systems needs improvement. Given that earlier studies (e.g., CNT/PI) have already identified the electron transfer pathway, please clarify the more specific novelty of this work that justifies its publication.

Response 4:

We thank the reviewer for highlighting the need to better define the novelty of this work relative to CNT/PI systems. We agree that CNT/PI studies have already shown that PI can mediate pollutant oxidation through an electron-transfer pathway[7]. Therefore, our comparison with CNT/PI is not intended to claim that ETP is unique to our system. Instead, we use PI activation as a model reaction to examine how peripheral substituents beyond the second coordination sphere remotely modulate the electronic structure of a defined Fe–N_4 single-atom site, thereby influencing PI adsorption, interfacial electron transfer, and pollutant oxidation kinetics.

This question differs from that addressed in CNT/PI systems because different active sites can give rise to distinct structure-activity relationships in PI activation. In the work of Lai et al.[7], CNTs were surface-modified through oxidative treatment, enrichment of oxygen-containing functional groups, and generation of defect/edge sites. Carbonyl/ketonic groups and double-vacancy defects were identified as the key motifs responsible for forming high-potential CNT-PI* intermediates. Thus, the active interface in that system is a modified carbon surface, and the central mechanistic issue is how carbon defects and oxygenated groups enable PI-mediated electron transfer. By contrast, our system is not designed around CNT activity. It uses atomically dispersed Fe–N_4 sites as the catalytic units, and PI activation occurs at a Fe–N_4/PI interfacial complex rather than at a heterogeneous carbon/PI surface.

The two systems also differ in the degree of structural control over the active interface. In CNT-based systems, ETP can be influenced simultaneously by conductivity, graphitic domains, oxygen-containing groups, vacancy defects, edge sites, and other surface features. These factors are all relevant to carbon-mediated PI activation, but they make it difficult to isolate one well-defined structural variable that controls ETP efficiency. In our R-FeSAC platform, the primary Fe–N_4 coordination structure is largely preserved, while the peripheral substituents are varied as F, H, and NH_2 (**Fig. 1a-b and Fig. R19**). This design allows us to separate remote substituent effects from first-shell coordination changes and to test whether long-range electronic communication through the conjugated framework can tune the Fe center.

The activity trend and mechanistic analyses support this site-specific remote regulation. F-FeSAC shows the highest catalytic activity (**Fig. 3a**), consistent with its more favorable electronic structure for PI activation. The OCP, i-t, and EIS results indicate more efficient Fe–N_4/PI complex formation and faster interfacial electron transfer on F-FeSAC (**Fig. 3g and Supplementary Figs. 24 and 26**). DFT calculations further show that the electron-withdrawing F substituent modulates the Fe electronic structure, strengthens PI adsorption, promotes interfacial charge redistribution, and improves the electronic alignment between the R-FeSAC/PI complex and organic pollutants (**Fig. 4d-h and Supplementary Figs. 31-33**). These results establish a direct relationship among peripheral substituent electronics, Fe-site electronic structure, PI adsorption/activation, charge-transfer resistance, and pollutant-removal kinetics.

Accordingly, the main advance of this work is the establishment of a peripheral-substituent-regulated Fe–N_4 single-atom platform for PI activation. CNT/PI studies demonstrate that modified carbon surfaces can mediate ETP, whereas our work shows that ETP efficiency can be tuned at a defined Fe–N_4 single-atom site through substituents beyond the second coordination sphere. This provides a more precise structure-electronic structure-reactivity relationship and extends ETP-based non-radical oxidation from carbon-surface chemistry to enzyme-inspired distal regulation of single-atom catalysts.

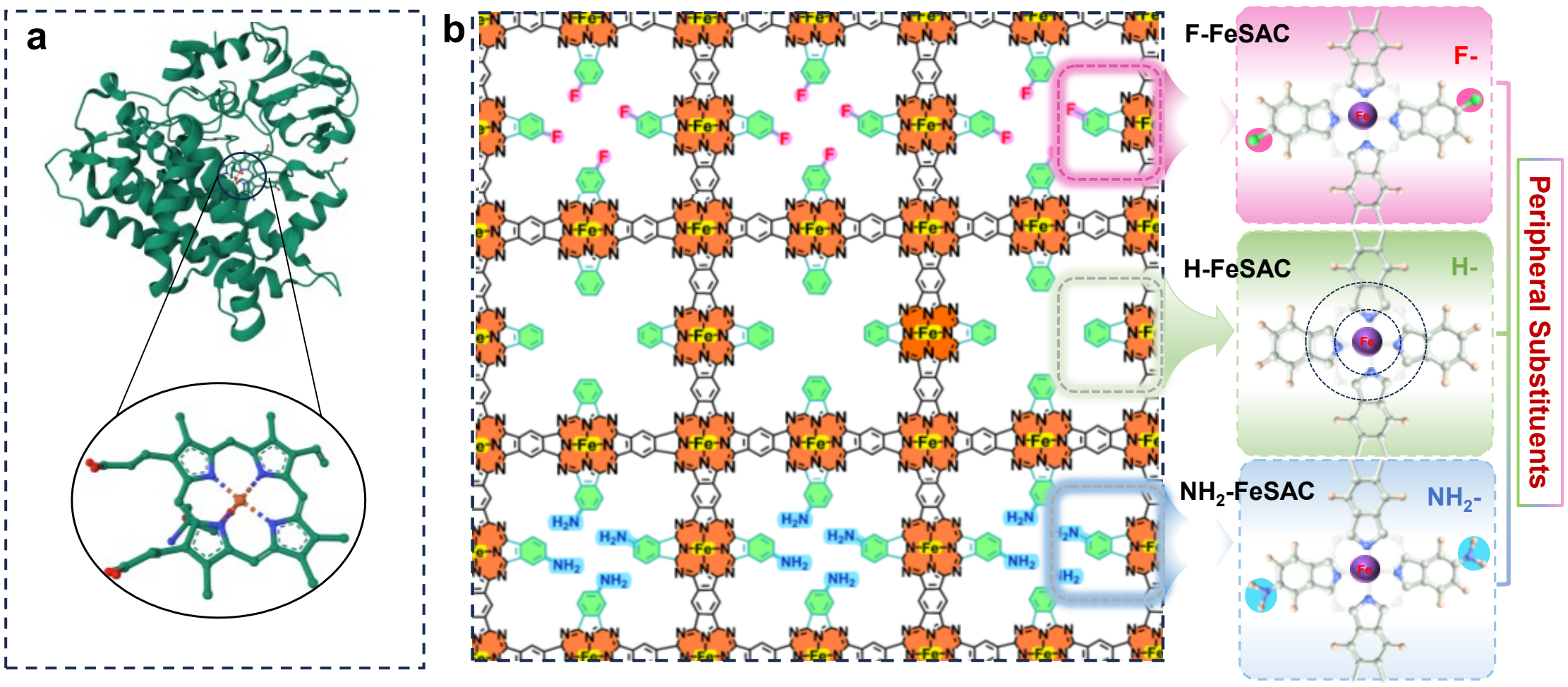

Fig. 1 (a) Model diagram of the Fe active site in the natural metalloenzymes. (b) Schematic of the preparation strategy for R-FeSAC.

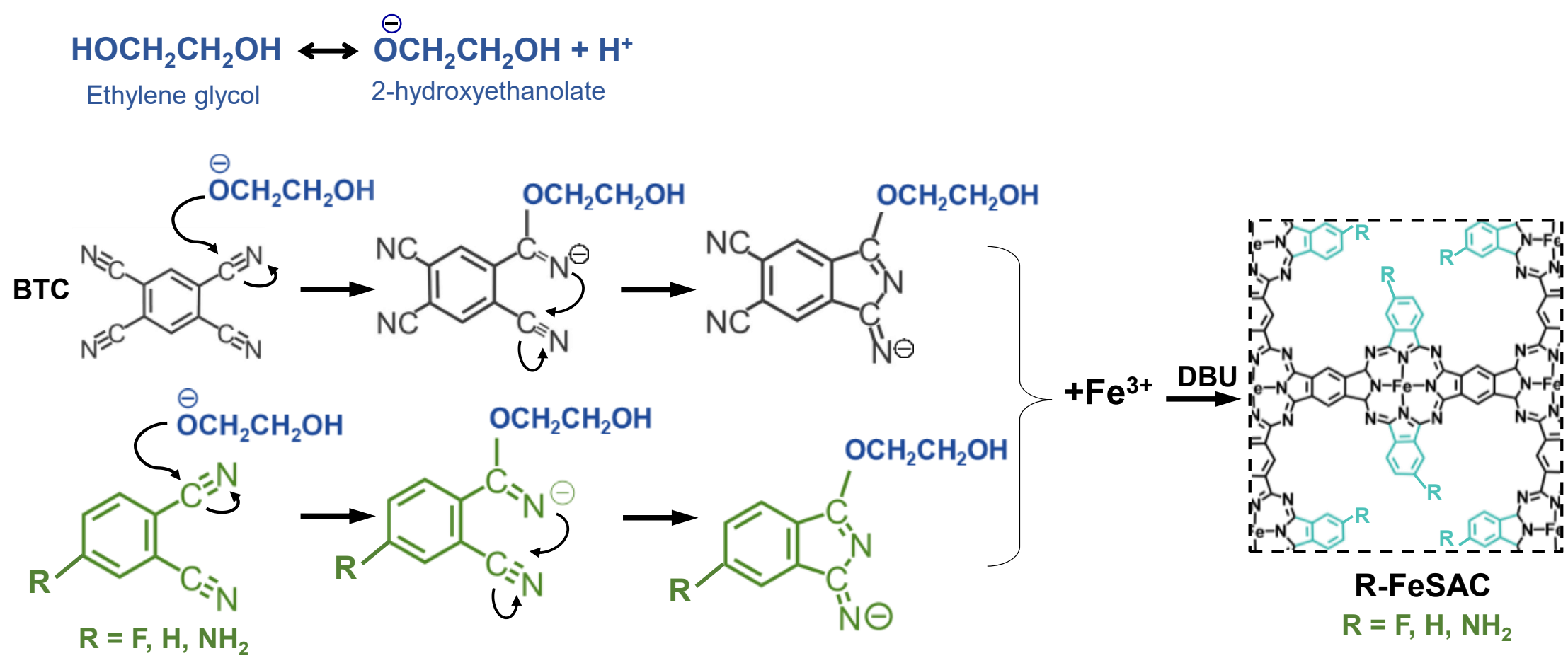

Fig. R19 Formation procedure of R-FeSAC.

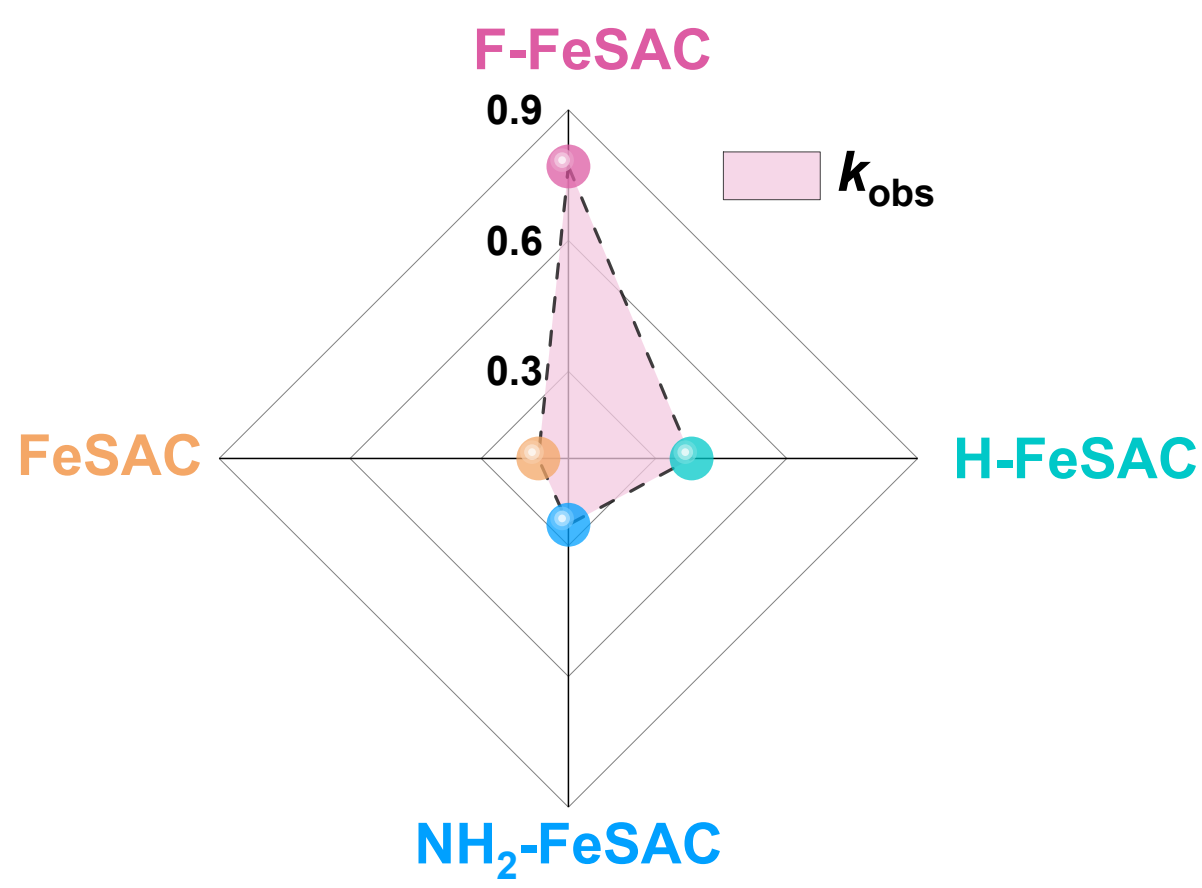

Fig. 3 (a) The k_{obs} of different catalytic systems on the degradation of 4-CP.

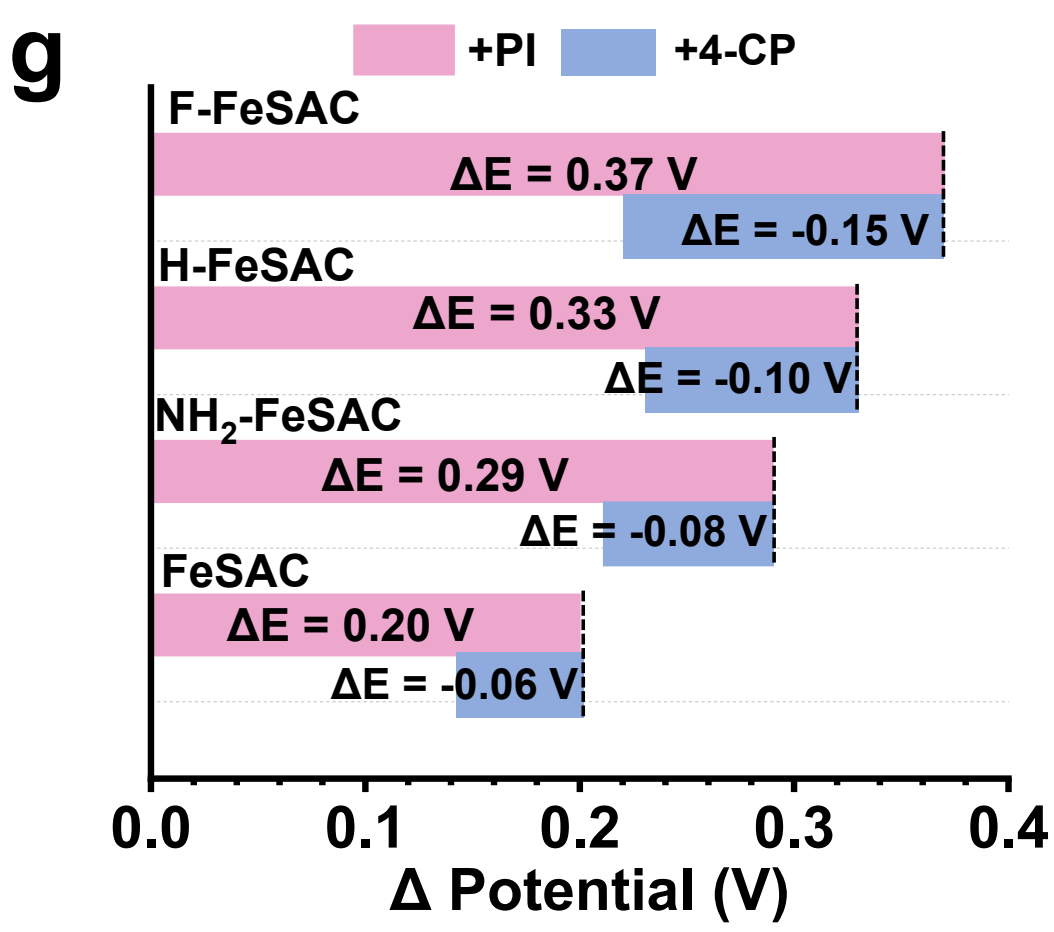

Fig. 3 (g) OCP changes of different systems with the addition of PI and 4-CP.

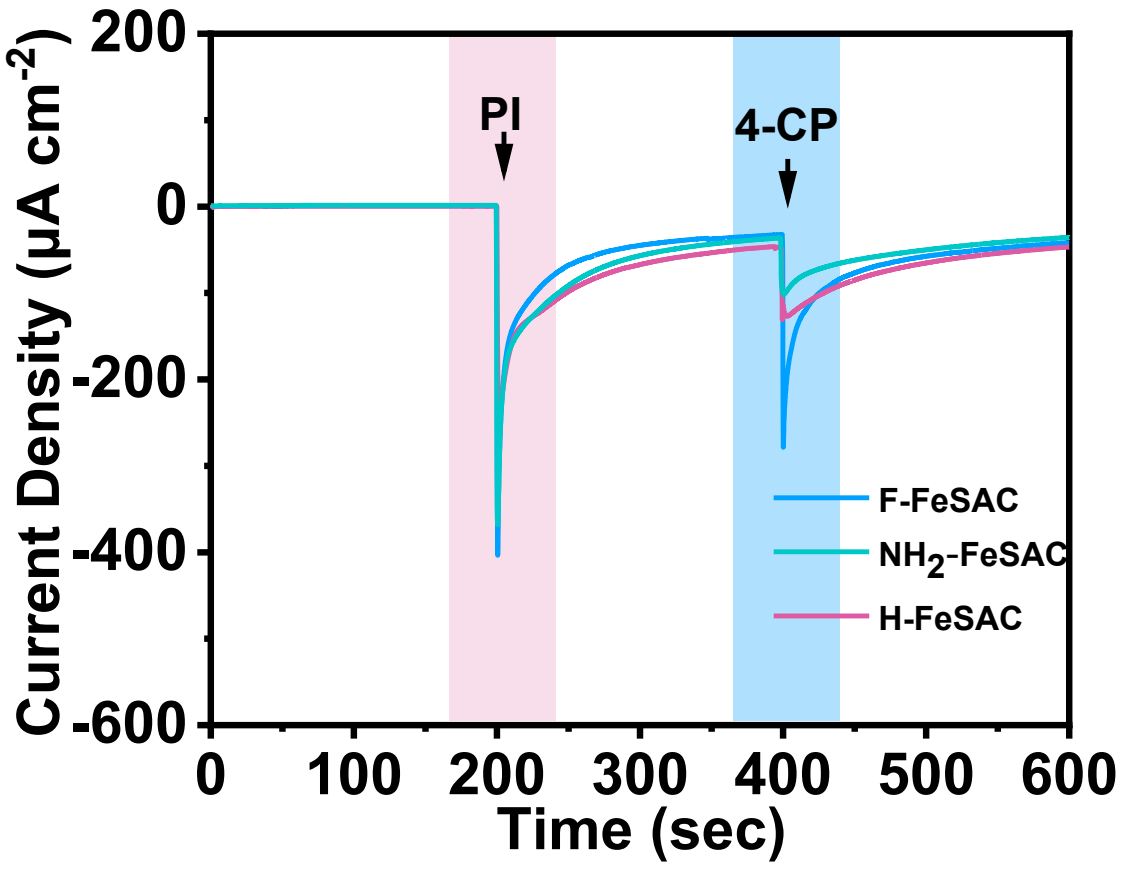

Supplementary Fig. 24 i-t curve of F-FeSAC, H-FeSAC and NH_2-FeSAC after adding PI and 4-CP successively.

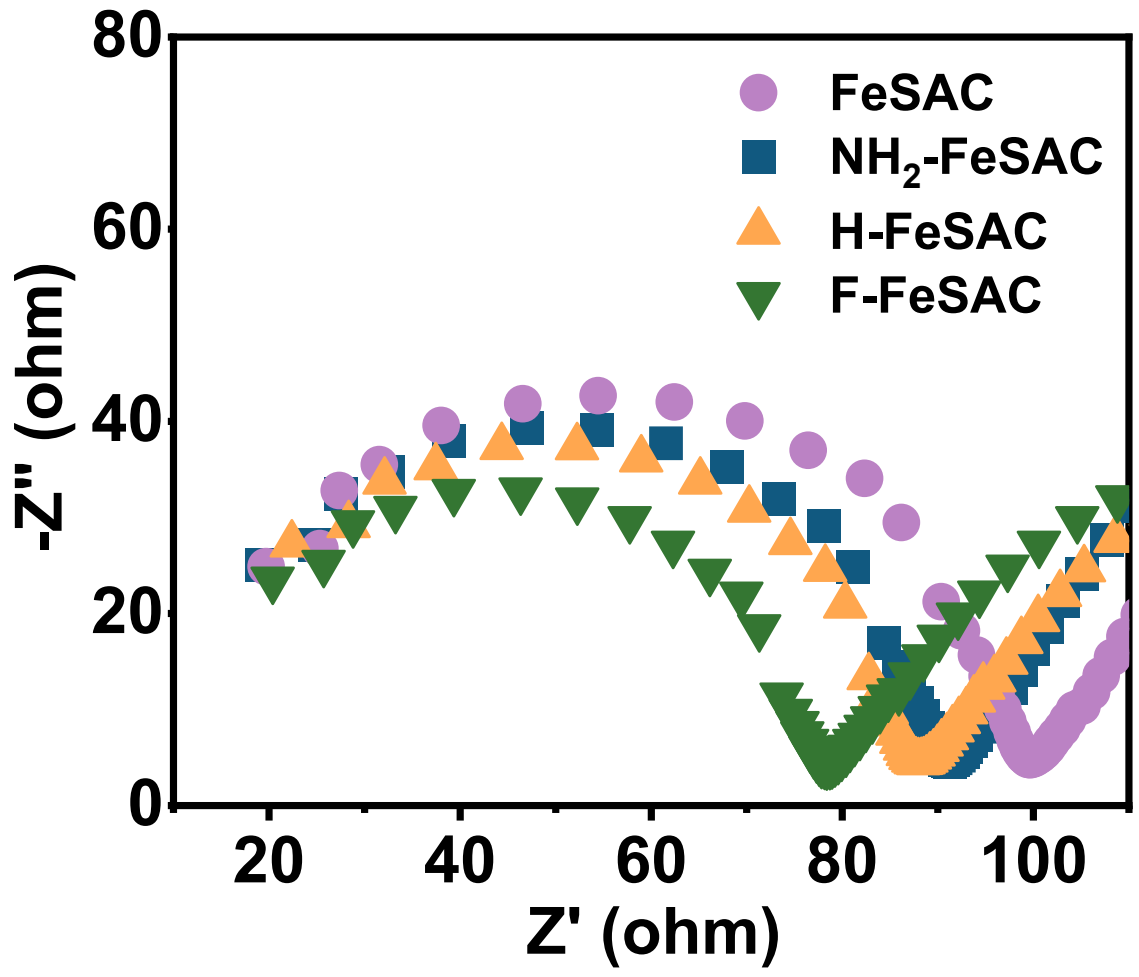

Supplementary Fig. 26 EIS analyses spectra of FeSAC, F-FeSAC, H-FeSAC and NH_2-FeSAC.

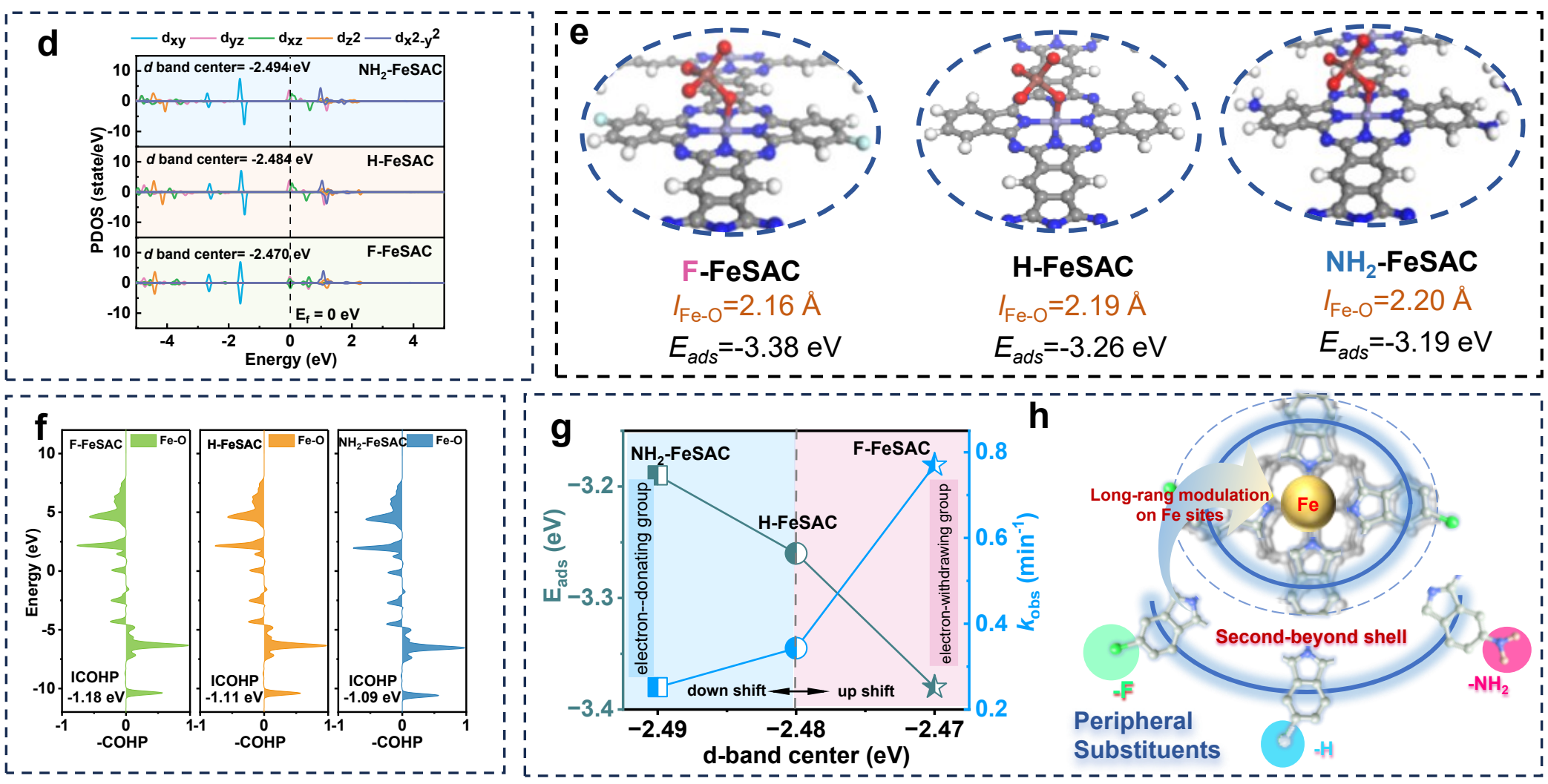

Fig. 4 (d) Projected Density of States of the Fe 3*d* orbitals in F-FeSAC, H-FeSAC, and NH_2-FeSAC. (e) Optimized adsorption configurations of PI at Fe site on the F-FeSAC, H-FeSAC, and NH_2-FeSAC. (f) Crystal orbital Hamilton population (COHP) analysis of Fe-O when PI adsorbs on Fe. (g) Correlations among k_{obs}, E_{ads}, and *d*-band center for F-FeSAC, H-FeSAC, and NH_2-FeSAC. (h) Schematic illustration of long-range electronic modulation at Fe sites induced by substituent groups beyond the secondary coordination sphere.

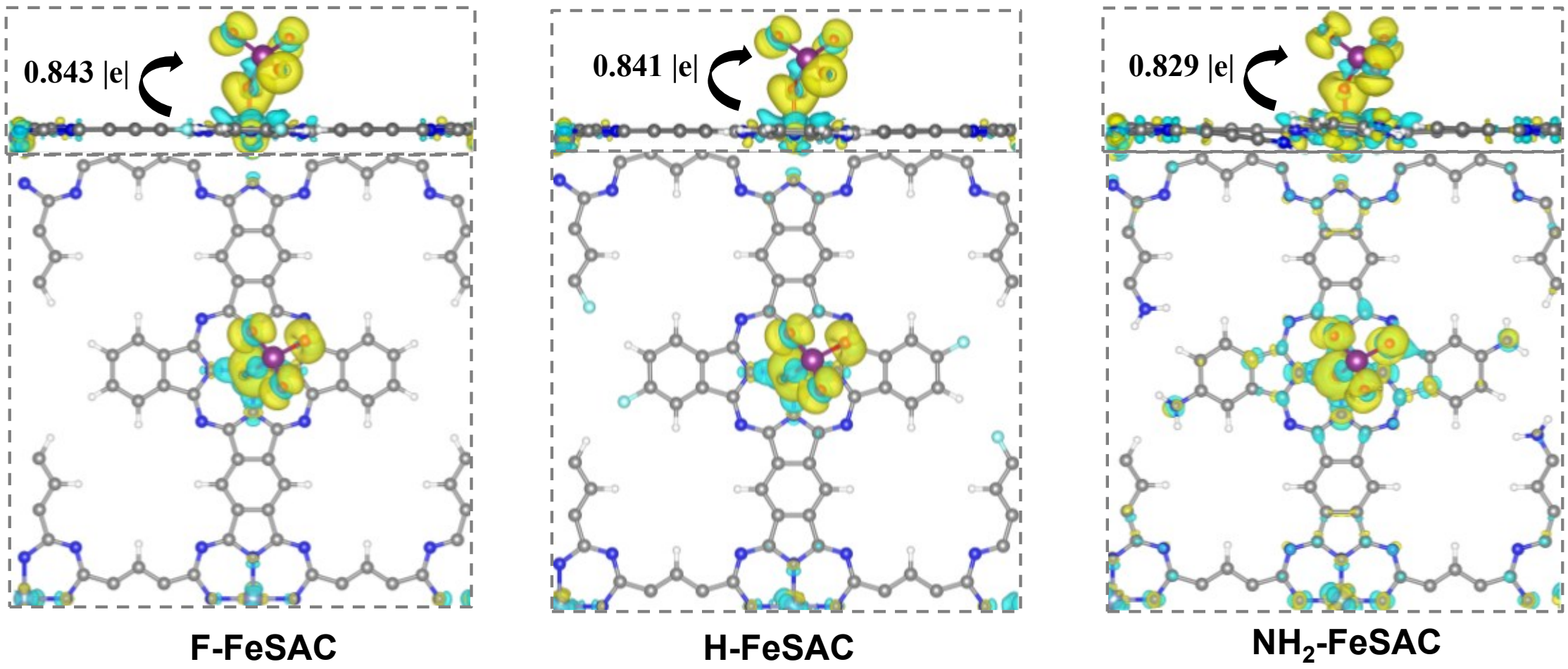

Supplementary Fig. 31 Differential charge density analysis of catalyst-PI complexes over F-FeSAC, H-FeSAC, and NH_2-FeSAC.

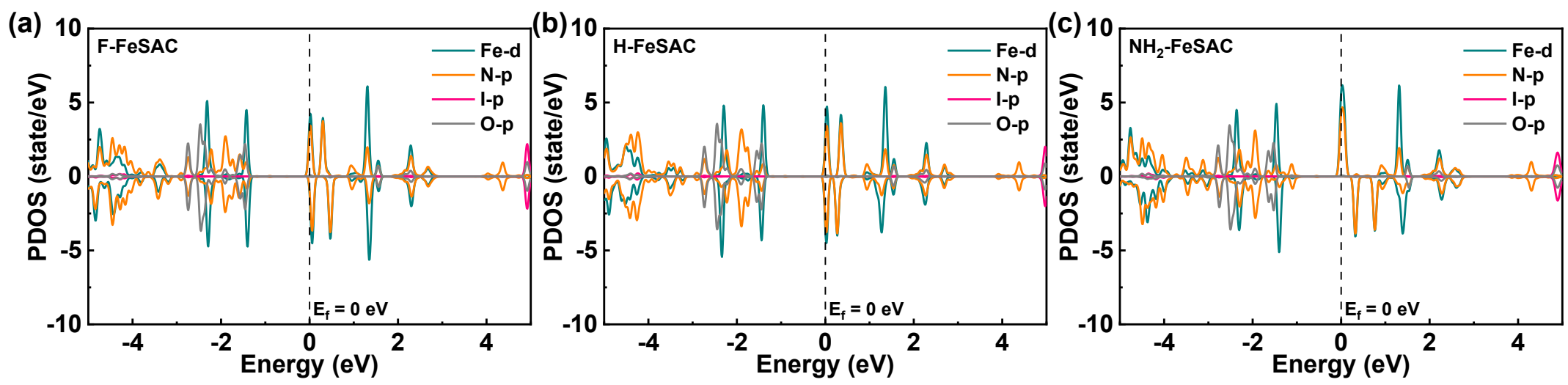

Supplementary Fig. 32 Projected density of states (PDOS) of PI-adsorbed (a) F-FeSAC, (b) H-FeSAC, and (c) NH_2-FeSAC.

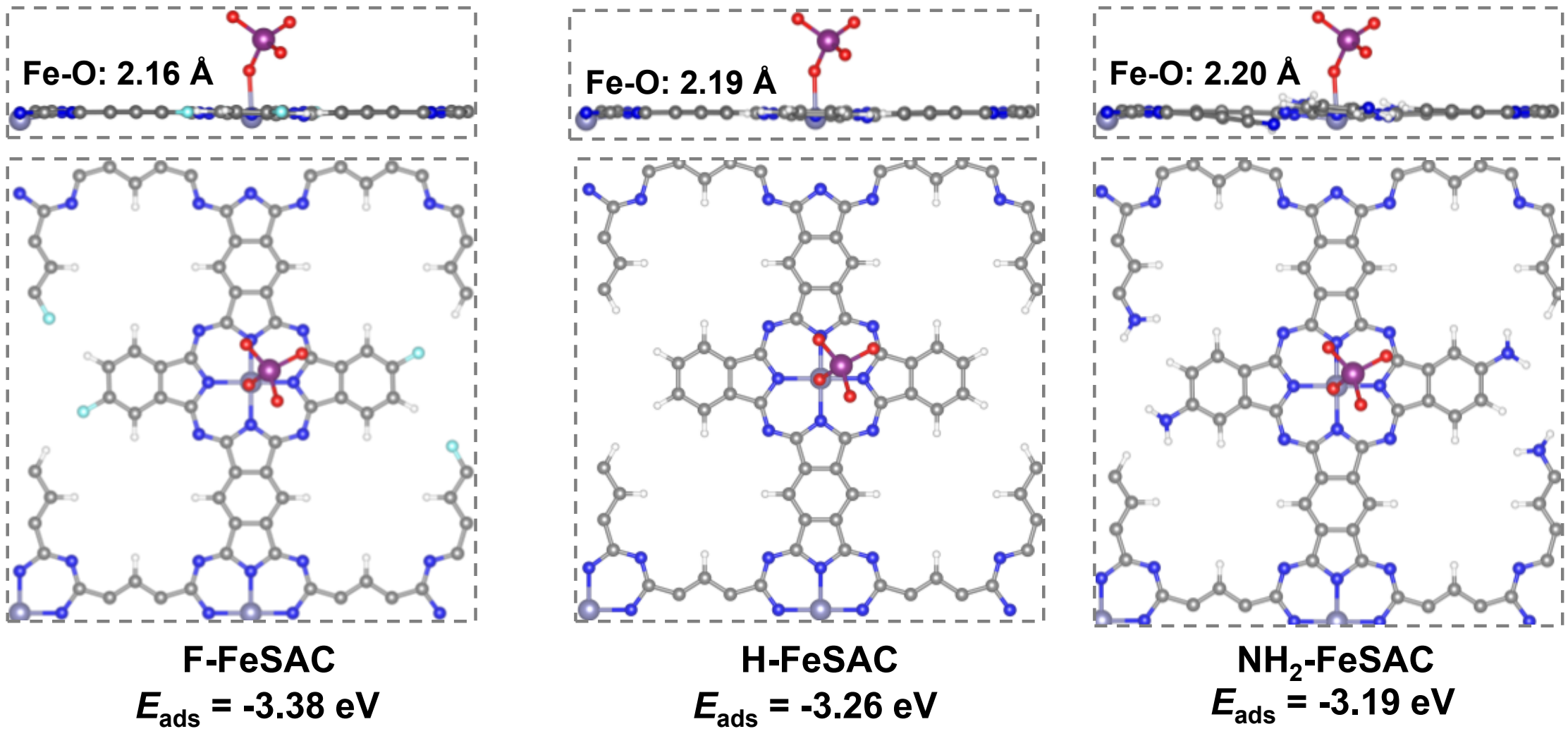

Supplementary Fig. 33 Optimized adsorption configurations of PI on F-FeSAC, H-FeSAC, and NH_2-FeSAC.

Action 4: We have added related statement in the Introduction section of the revised manuscript.

"Lai et al.[7] recently identified an ETP pathway in the functionalized carbon nanotube (CNT)/PI system, in which CNTs were modified through oxidative treatment to introduce oxygen-containing functional groups, defect sites, and edge structures. Carbonyl/ketonic groups and double-vacancy defects were proposed to function as electron-shuttling motifs that mediate direct electron transfer from organic contaminants to PI. This mechanistic insight into ETP is pivotal, as it underscores the critical role of catalyst design in optimizing PI adsorption and interfacial redox potentials. However, in CNT-based systems, ETP can be influenced simultaneously by conductivity, graphitic domains, oxygen-containing groups, vacancy defects, edge sites, and other surface features. As a result, although CNT/PI studies establish carbon-surface-mediated ETP, precise regulation of ETP at a structurally defined catalytic site remains insufficiently explored."

5. Is the observed ETP mechanism unique to Fe? If the interaction with PI is significant, would other transition metals (e.g., Co or Ni) exhibit similar performance within this scaffold? A brief comparison or discussion on metal specificity would strengthen the manuscript.

Response 5:

We thank the reviewer for this important comment. We agree that the metal specificity of the observed ETP behavior should be clarified more carefully, particularly whether ETP is unique to Fe or can also occur over other transition-metal centers within

the same scaffold.

ETP is not exclusive to Fe

While Fe is the central active metal in our design, ETP-type PI activation is not necessarily exclusive to Fe. Previous studies have shown that other atomically dispersed transition-metal sites can also activate PI through electron-transfer-mediated non-radical pathways. For example, Long et al.[57] reported that atomically dispersed Co-N sites on graphene can activate PI through N-rGO-CoSA/PI metastable complexes, which act as dominant oxidizing species in the non-radical oxidation process. Inspired by this precedent and following the reviewer's suggestion, we further examined whether other transition metals could exhibit similar ETP behavior within our scaffold.

Fe remains the most active center within the same scaffold

To address this question directly, we prepared Co-, Ni-, and Cu-based analogues within the same conjugated scaffold and evaluated their catalytic performance under identical reaction conditions. Even within this matched structural platform, the Fe catalyst consistently delivered the highest PI activation efficiency and pollutant removal performance, whereas the Co-, Ni-, and Cu-containing counterparts showed distinctly lower activities (**Fig. R22**). This result indicates a clear metal-dependent activity trend within the same scaffold[21].

ETP can occur over different metal centers, but with metal-dependent efficiency.

The mechanistic evidence further supports this conclusion. Radical-quenching experiments and premixing tests for F-CoSAC, F-NiSAC, and F-CuSAC ruled out dominant contributions from radical pathways, high-valent metal-mediated oxidation, and singlet oxygen oxidation **(Fig. R23)**. The galvanic oxidation process (GOP) experiments further confirmed that pollutant oxidation in these systems proceeds predominantly through electron transfer (**Fig. R24**). These results indicate that ETP is not unique to Fe in the present scaffold. Instead, the key difference among the four metal centers lies in how efficiently each catalyst-PI complex drives interfacial electron transfer.

The DFT results provide a molecular-level explanation for this metal dependence. Differential charge-density analysis reveals pronounced interfacial electron redistribution in all four catalyst-PI complexes **(Fig. R25)**. In every case, electron depletion and accumulation are concentrated around the metal-PI interface and extend into the coordinated PI moiety, indicating electronic coupling between the active site and PI. However, the magnitude of charge transfer varies substantially among the metal

centers, following the order F-FeSAC (0.843 e) > F-CuSAC (0.822 e) > F-NiSAC (0.795 e) > F-CoSAC (0.784 e). These results indicate that all four metals can electronically activate PI, but the strength of interfacial charge transfer is clearly metal-dependent.

A similar trend is observed in the PI adsorption energetics. All four metal centers form thermodynamically stable catalyst-PI complexes, with adsorption energies of −3.378, −3.056, −2.900, and −2.827 eV for F-FeSAC/PI, F-CuSAC/PI, F-NiSAC/PI, and F-CoSAC/PI, respectively **(Fig. R26)**. Notably, Fe provides the strongest stabilization of the catalyst-PI complex among the examined metals. Together with the largest interfacial charge transfer observed for F-FeSAC, this result identifies Fe as the most favorable metal center for promoting PI activation and ETP-driven oxidation within this scaffold.

Taken together, these results show that ETP is not unique to Fe in the present scaffold. Rather, Fe exhibits the most favorable metal specificity in terms of catalytic efficiency because it affords the strongest catalyst-PI stabilization and the most pronounced interfacial electronic coupling. Thus, the metal specificity of Fe is reflected not in the exclusive occurrence of ETP, but in its superior ability to promote PI activation and electron-transfer-driven pollutant oxidation within this scaffold.

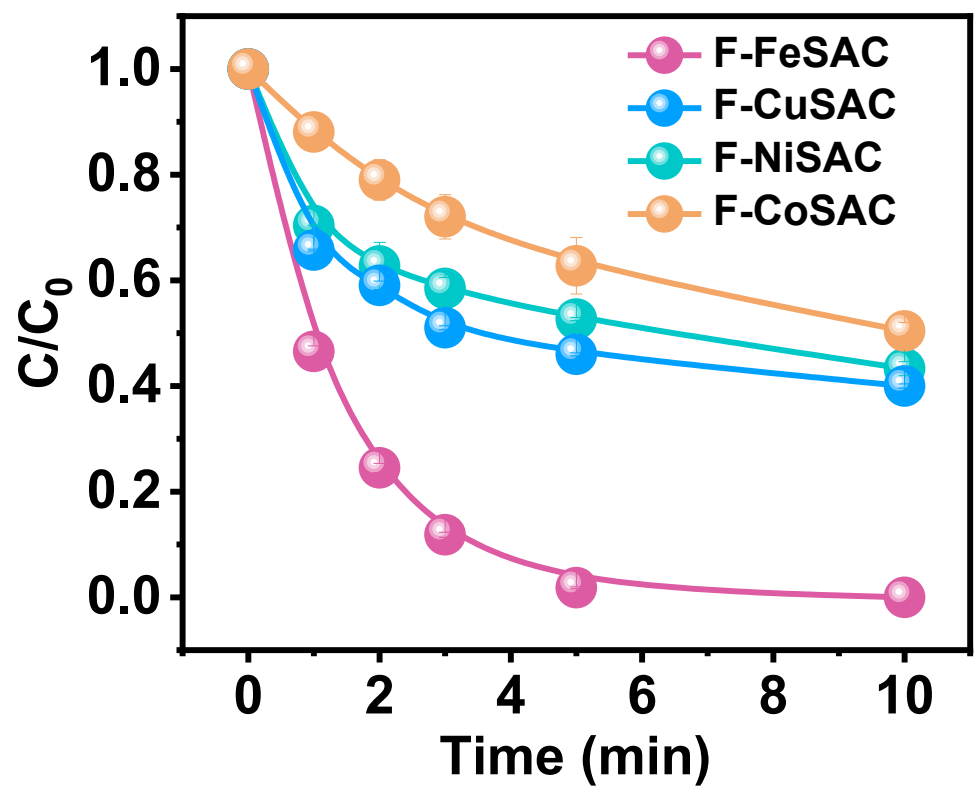

Fig. R22 Effects of different metal catalytic systems on the degradation of 4-CP. Routine conditions: [4-CP] = 80 μM, [catalyst] = 50 mg/L, [PI] = 0.20 mM, temperature = 25 °C, without pH adjustment.

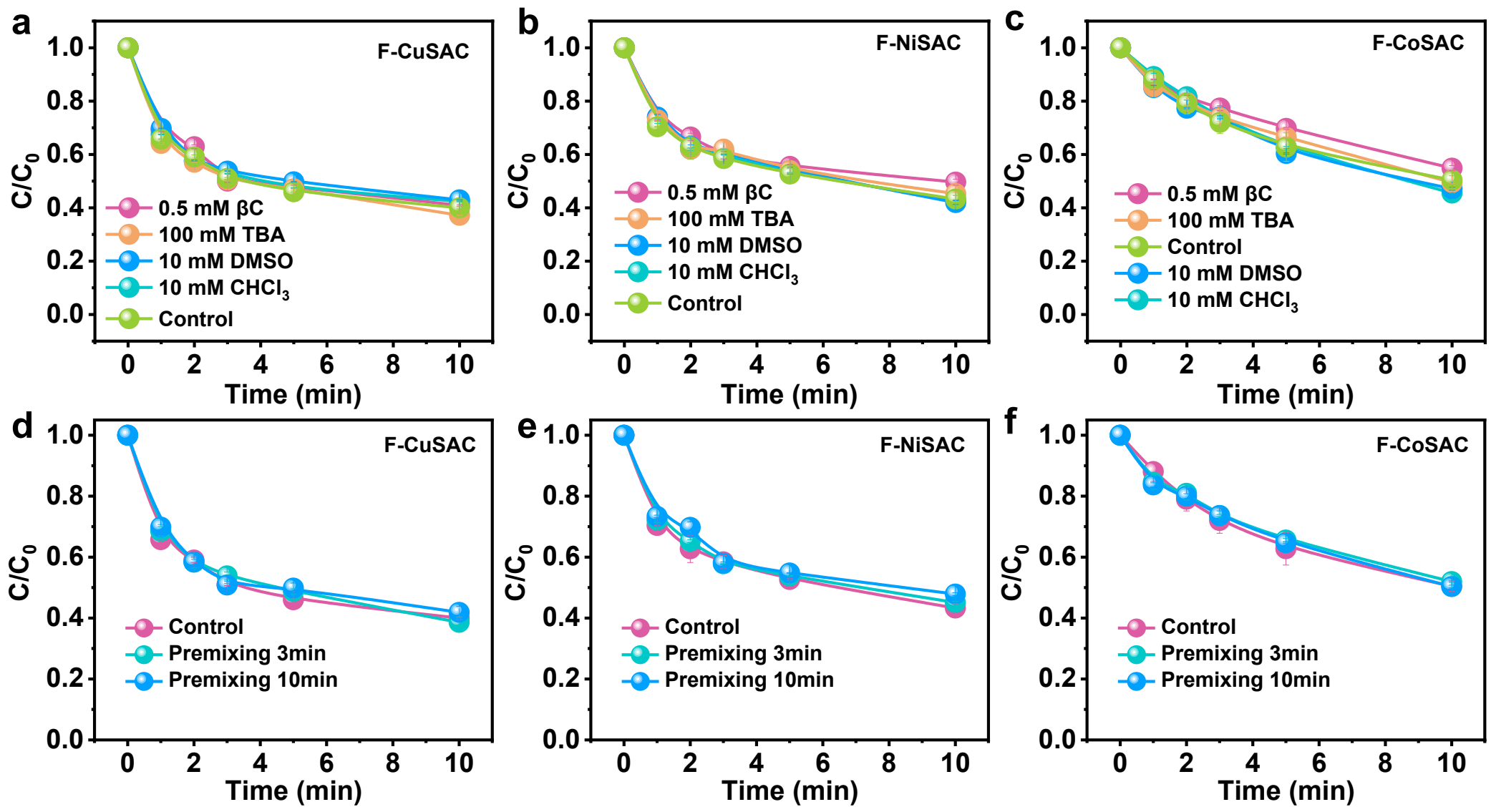

Fig. R23 Degradation of 4-CP in (a) F-CuSAC/PI, (b) F-NiSAC/PI, and (c) F-CoSAC/PI system under different quenchers. Effect of premixing in the (d) F-CuSAC/PI, (e) F-NiSAC/PI, and (f) F-CoSAC/PI system on 4-CP removal. Routine conditions: [4-CP] = 80 μM, [catalyst] = 50 mg/L, [PI] = 0.20 mM, temperature = 25 °C, without pH adjustment.

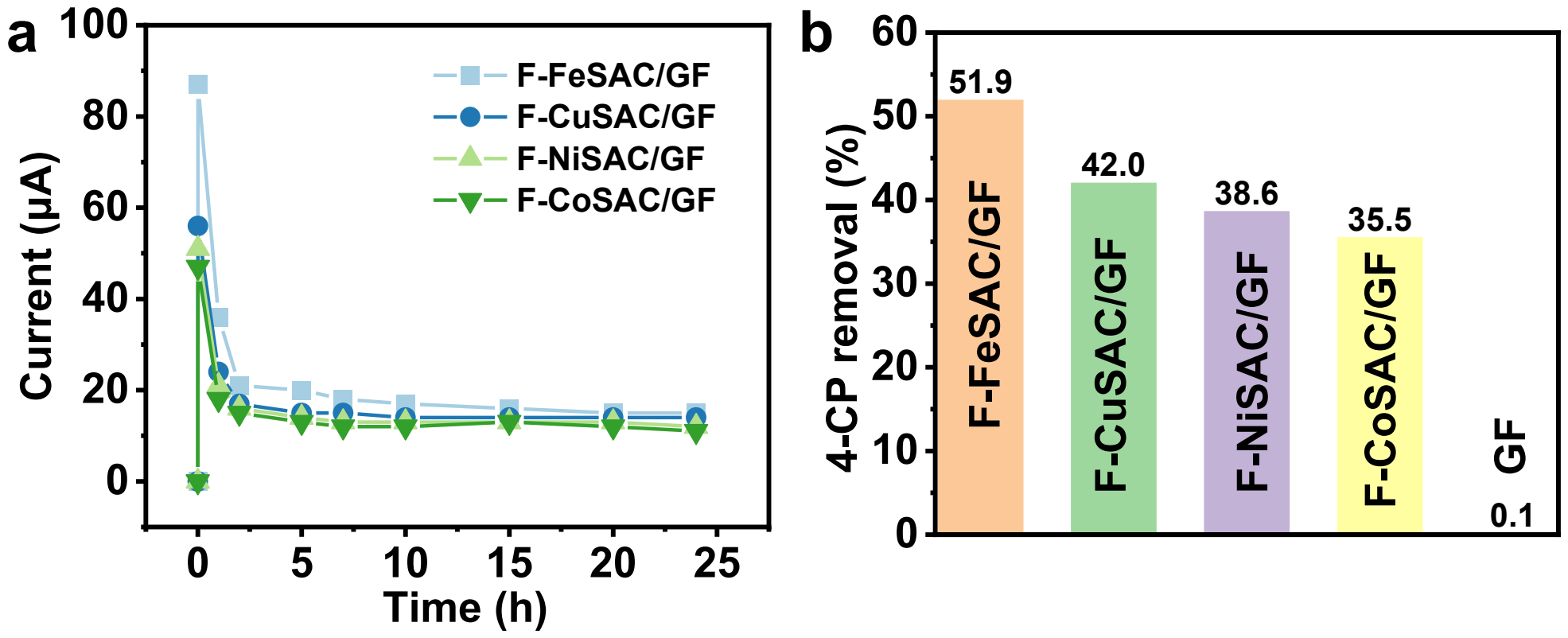

Fig. R24 Measurement of (a) current-time curves and (b) removal rates of 4-CP in F-CuSAC/GF, F-NiSAC/GF, and F-CoSAC/GF GOP systems.

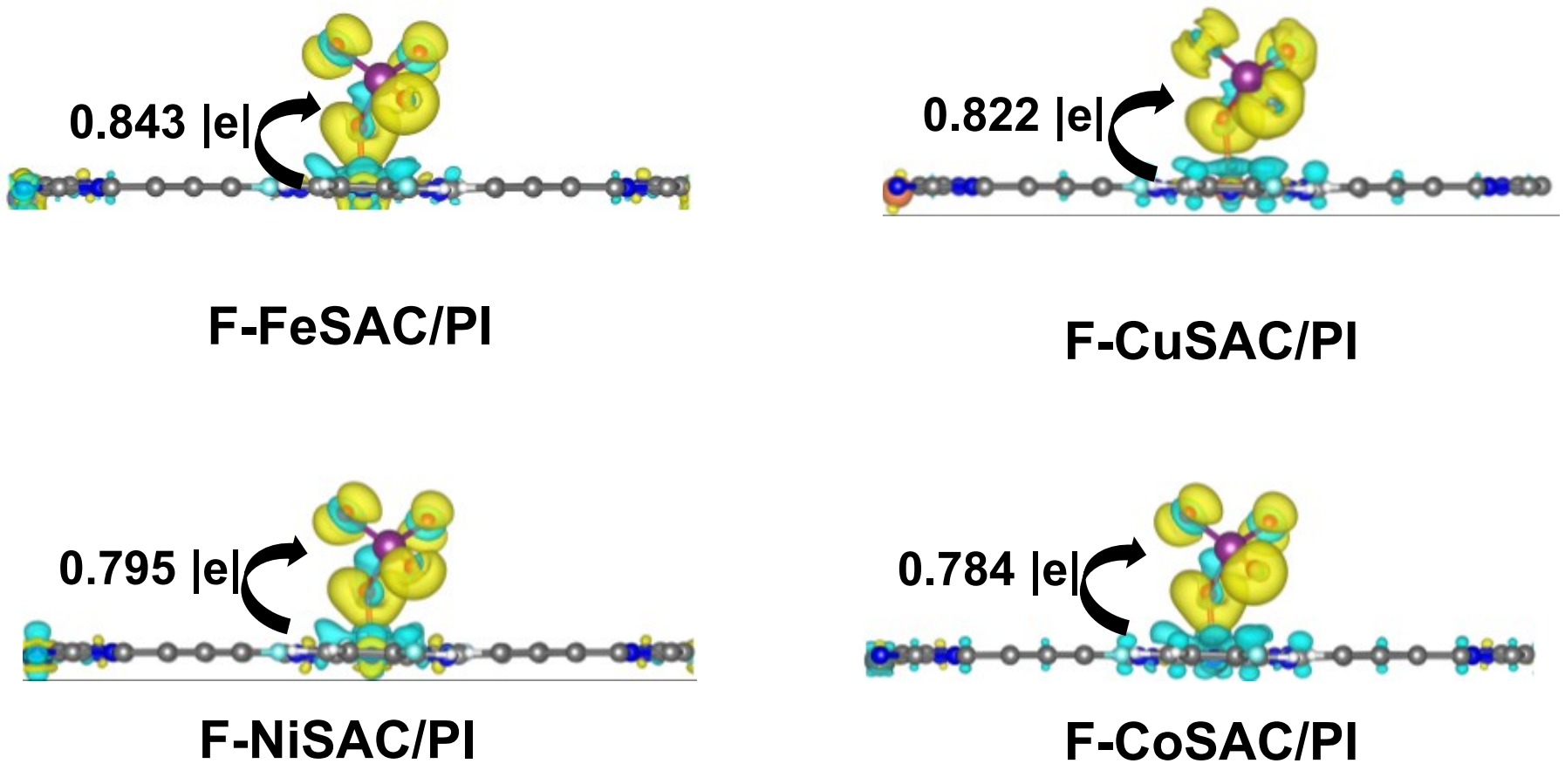

Fig. R25 Differential charge density analysis of catalyst-PI complexes over Fe-, Co-, Ni-, and Cu-based analogues within the same scaffold.

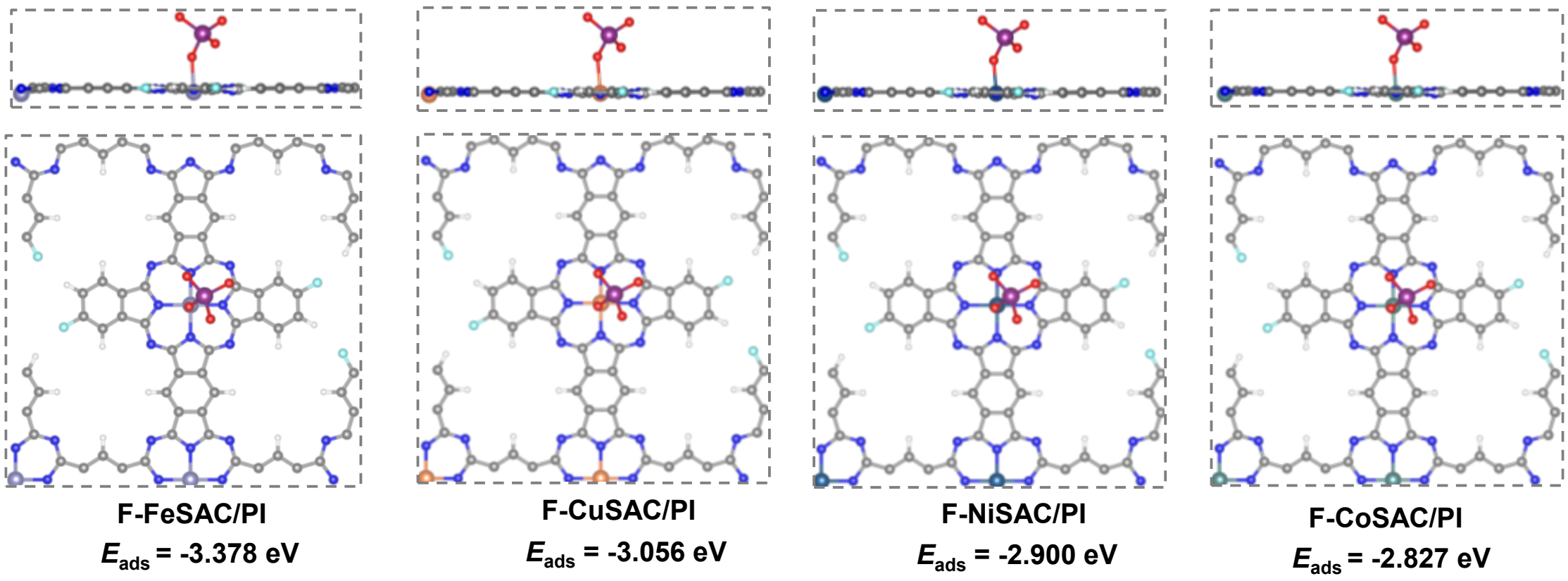

Fig. R26 Optimized adsorption configurations of PI on Fe-, Co-, Ni-, and Cu-based analogues within the same scaffold.

6. The manuscript attributes 4-CP removal to a polymerization-mediated ETP. The LC-MS identification of chlorinated oligomeric species on the used catalyst is consistent with this. However, to substantiate the claim that polymerization is the dominant pathway, a clearer mechanistic description of how ETP leads to oligomer formation would be necessary.

Response 6:

We thank the reviewer for this insightful comment. We agree that a clearer mechanistic explanation is needed to show how the ETP pathway leads to oligomer formation.

In our view, the key role of ETP is not simply to oxidize 4-CP, but to selectively convert 4-CP into coupling-active intermediates. Specifically, 4-CP first undergoes one-electron transfer at the catalyst/oxidant interface to generate phenoxy radical or

phenoxonium-type intermediates. Previous studies on polymerization-oriented oxidation of phenolic pollutants have shown that such one-electron oxidation products are the key precursors for oligomer formation, rather than direct ring-cleavage products[58].

Once formed, these intermediates exhibit delocalized spin density and positive charge over the phenoxy oxygen and the aromatic ortho/para positions. This delocalization makes them highly susceptible to intermolecular coupling, so that neighboring 4-CP-derived intermediates can react through C–C and C–O bond formation to generate dimers, which can then undergo further coupling to produce trimers and higher oligomers.

In addition, because the ETP process occurs at the catalyst surface, these reactive intermediates are generated in a confined interfacial region rather than uniformly in the bulk solution. Such surface confinement favors the adsorption, stabilization, and local accumulation of the phenoxy/phenoxonium-type intermediates, thereby increasing the probability of intermolecular coupling over fragmentation. Recent work has similarly shown that interfacial electron transfer can promote the formation, stabilization, and aggregation of polymeric precursors from 4-CP[59].

Therefore, the mechanistic link between ETP and oligomer formation can be described as follows: ETP first initiates one-electron oxidation of 4-CP, producing phenoxy/phenoxonium-type intermediates; these intermediates then undergo intermolecular C–C/C–O coupling at the catalyst interface; repeated coupling finally leads to chlorinated dimers, trimers, and higher oligomeric products. Thus, polymerization is intrinsically linked to the ETP pathway, because the initial oxidation step directly generates the coupling-active intermediates that drive oligomer formation.

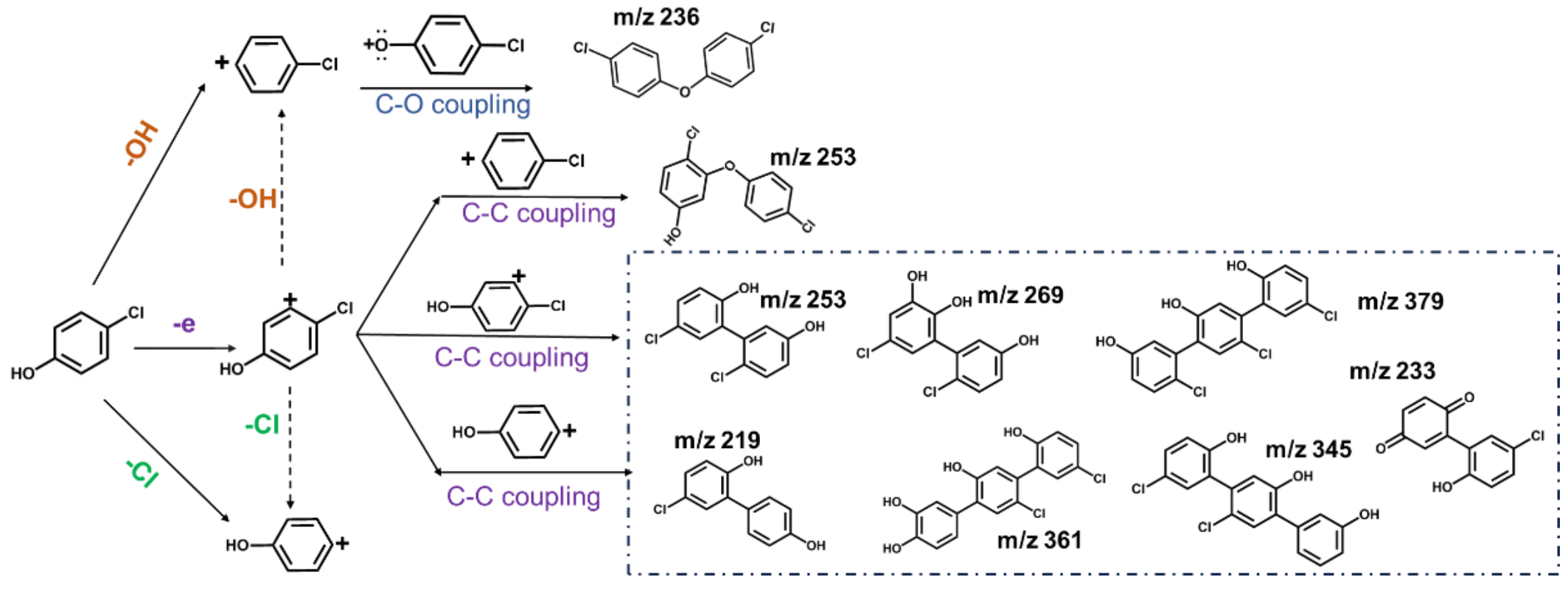

Fig. R8 Possible transformation pathways of 4-CP in F-FeSAC/PI.

Action 6: We have revised the corresponding section to better explain how the polymerization-mediated ETP pathway leads to oligomer formation.

"Characterization of the accumulated surface species provided mechanistic insight. Liquid chromatography-mass spectrometry (LC-MS) analysis of the solution following desorption identified chlorinated oligomers as the main surface-adsorbed products formed during 4-CP oxidation (Supplementary Figs. 34-35). Their formation supports a polymerization-mediated electron-transfer pathway. Specifically, 4-CP is first oxidized through interfacial electron transfer to generate phenoxy- or phenoxonium-type intermediates. These intermediates are resonance-stabilized over the oxygen atom and the ortho/para positions of the aromatic ring, making them prone to intermolecular C–C and C–O coupling. Repeated coupling of these surface-generated intermediates then leads to chlorinated dimers, trimers, and higher oligomeric products. Owing to their low solubility and strong interfacial affinity, these oligomeric products preferentially accumulate on the catalyst surface, leading to progressive activity attenuation during cycling."

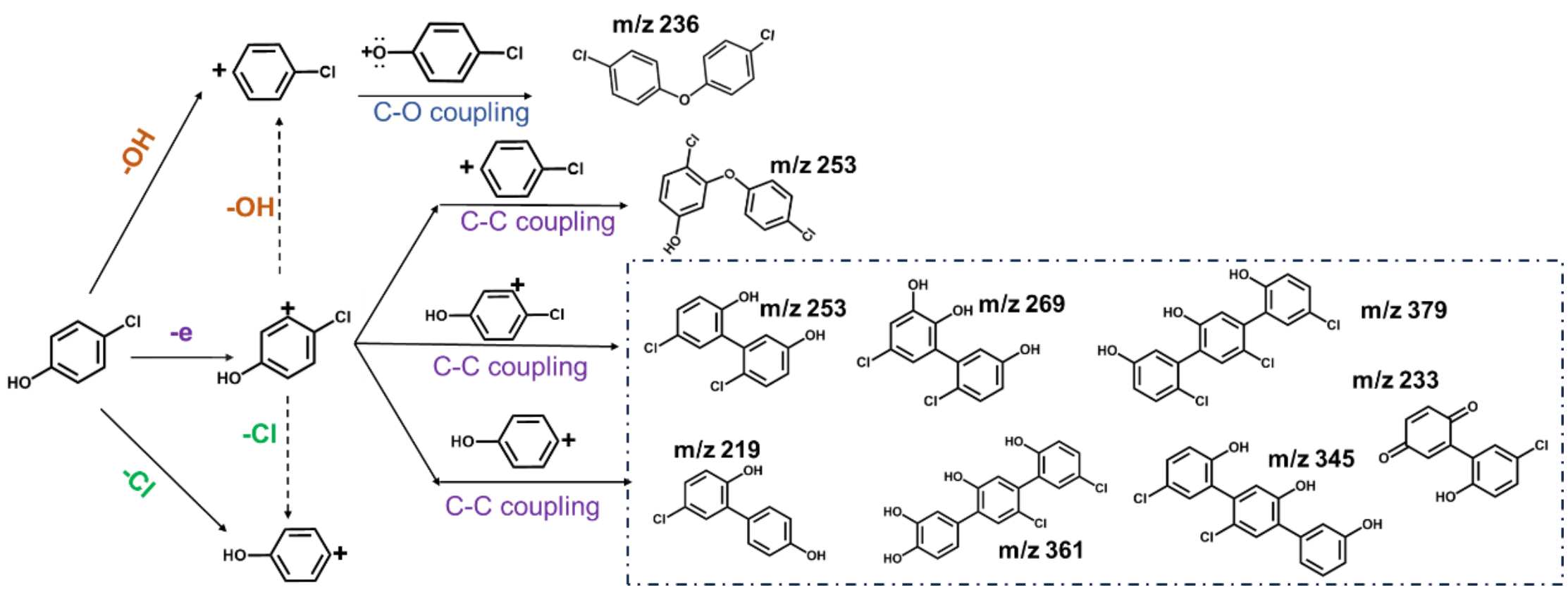

Supplementary Fig. 34 Possible transformation pathways of 4-CP in F-FeSAC/PI.

7. The claim of a non-radical mechanism relies on negative evidence. A direct comparison between IO3 radicals and IO3- ions would be more convincing than noting the absence of a radical peak. Additionally, please provide positive reference data to prove the detection is sensitive enough.

Response 7:

We sincerely thank the reviewer for this insightful and important comment. We appreciate the reviewer's suggestion to further substantiate the non-radical electron-transfer pathway with evidence beyond the absence of radical signals. Accordingly, we have strengthened the mechanistic analysis by integrating exclusion of alternative

oxidizing species with direct and supportive evidence for catalyst/PI-mediated interfacial electron transfer. We also added targeted experiments to clarify the distinct roles of stable IO_3^- and transient $IO_3^•$, along with a positive reference system to validate the sensitivity of $IO_3^•$ detection.

First, we used exclusion experiments to rule out major alternative oxidation pathways. Radical scavenging experiments, EPR measurements, NBT assays, β-carotene quenching, D_2O substitution, DPA probing, PMSO oxidation, and iodine-speciation analyses collectively show that •OH, $O_2^{•-}$, $^1O_2$, high-valent Fe-oxo species, and reactive iodine intermediates do not make dominant contributions to 4-CP oxidation in the R-FeSAC/PI system (**Fig. 3c; Supplementary Figs. 12-21**). These results narrow the plausible oxidation mechanism to a non-radical pathway and provide the basis for further evaluating the electron-transfer process.

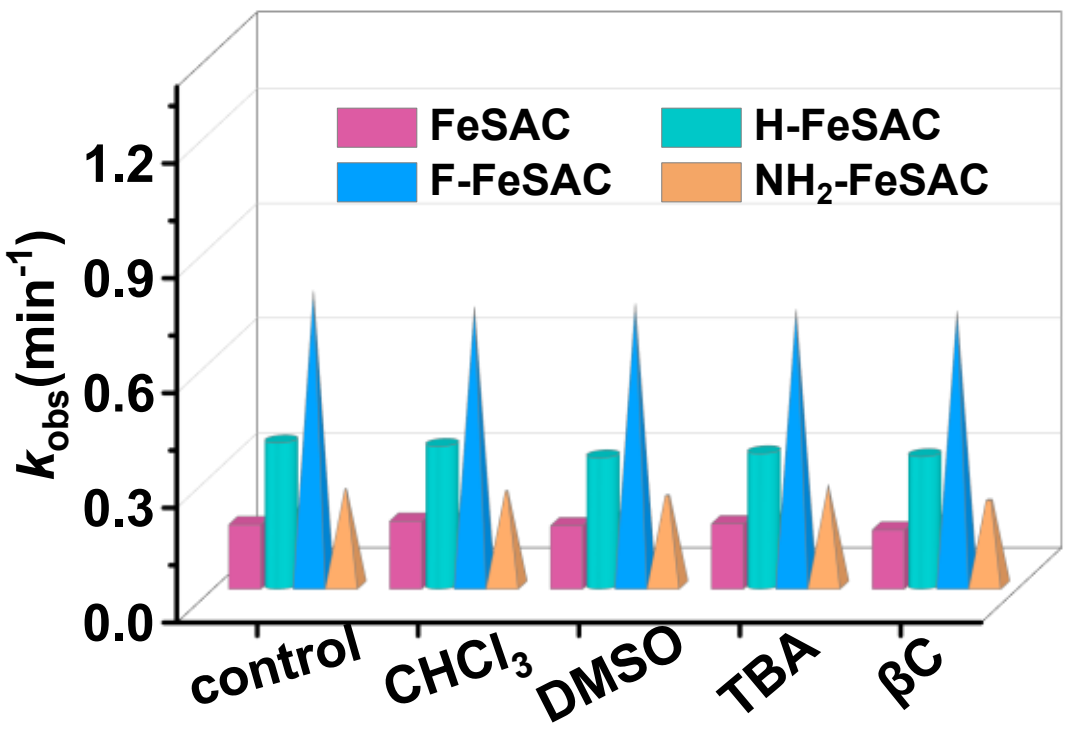

Fig. 3 (c) The k_{obs} values of FeSAC/PI and R-FeSAC/PI systems in the presence of various quenchers.

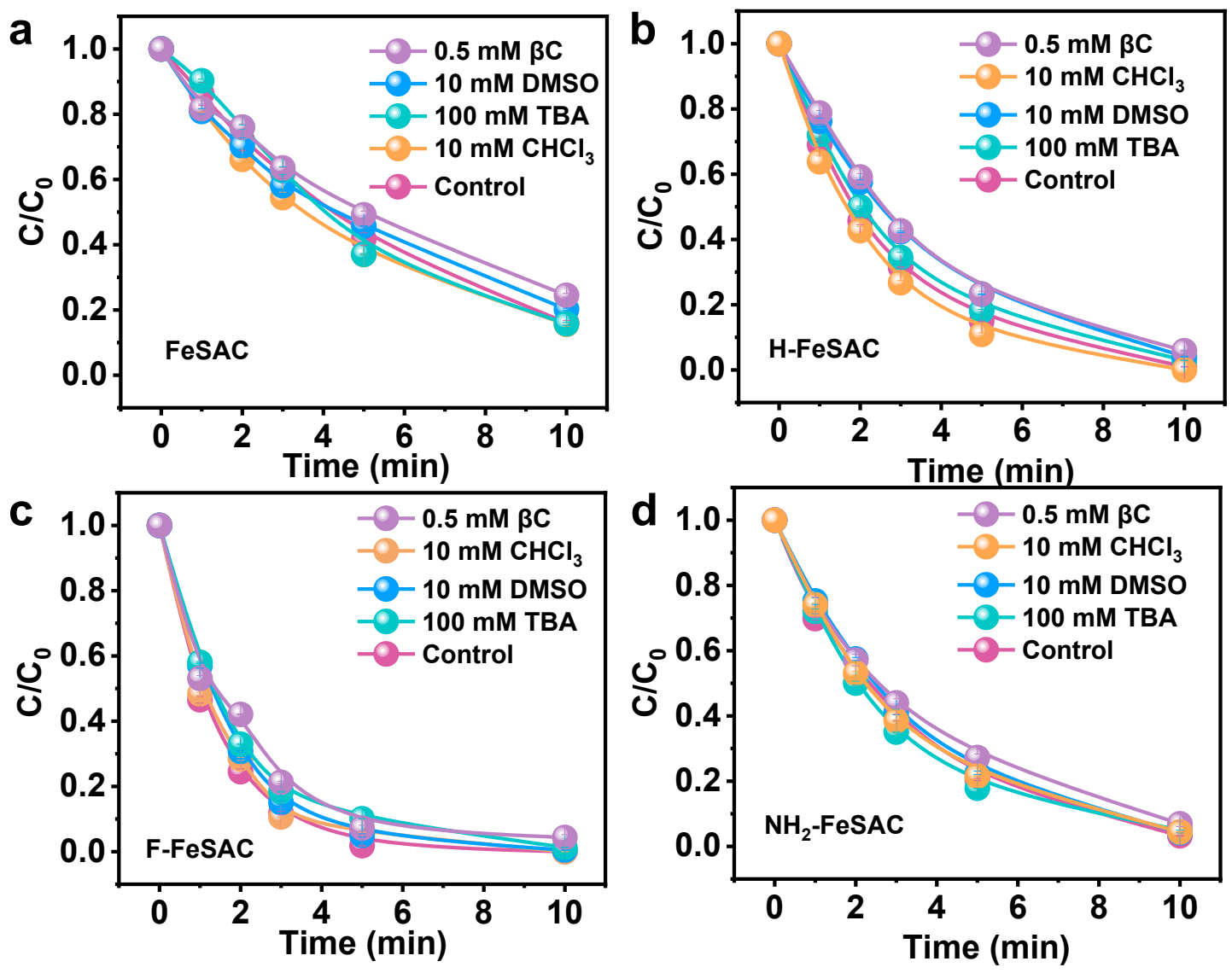

Supplementary Fig. 12 Degradation of 4-CP in (a) FeSAC/PI, (b) H-FeSAC/PI, (c) F-FeSAC/PI and (d) NH_2-FeSAC/PI system under different quenchers. Routine

conditions: [4-CP] = 80 μM, [catalyst] = 50 mg/L, [PI] = 0.20 mM, temperature = 25 °C, without pH adjustment.

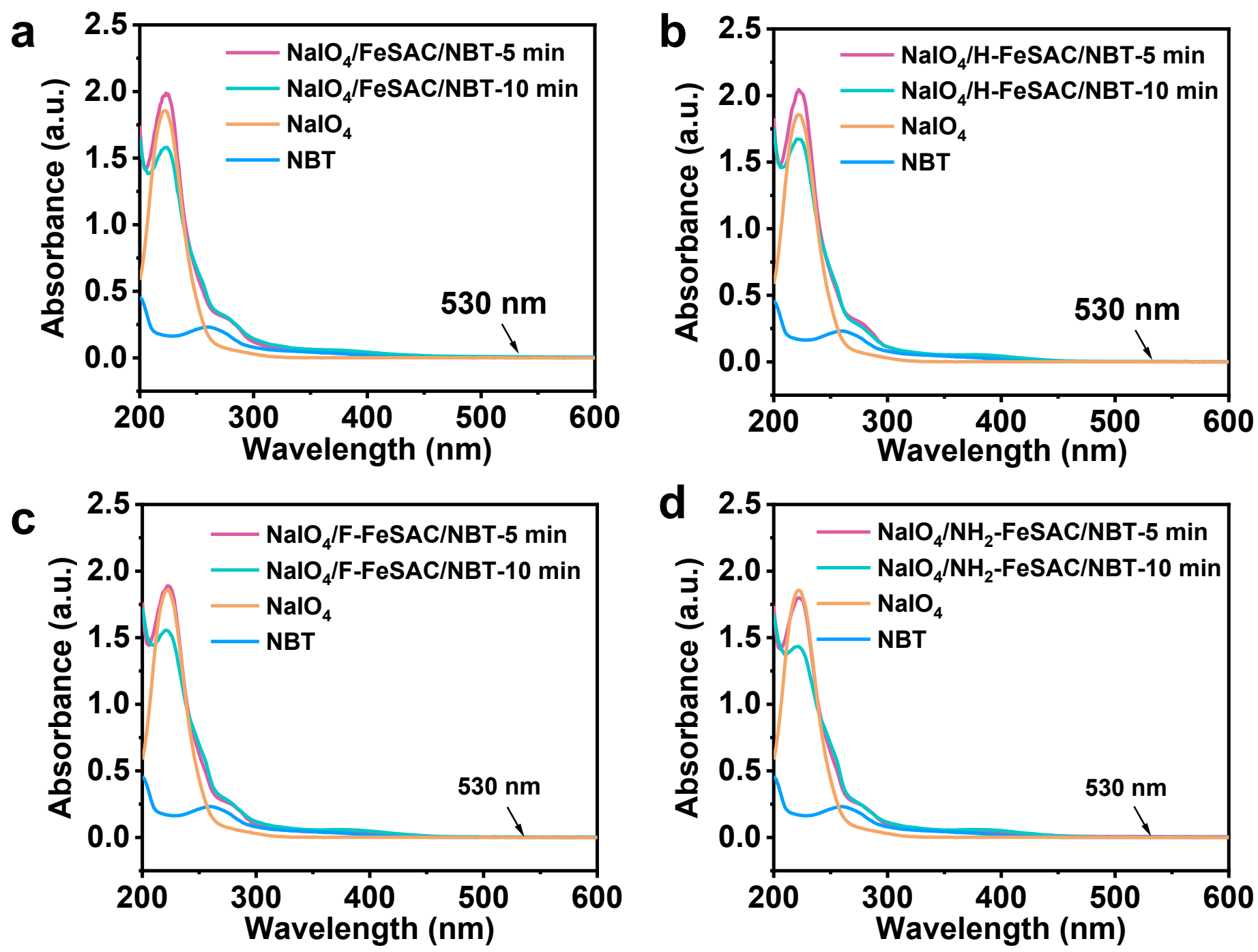

Supplementary Fig. 13 UV-vis absorption spectra of NBT in the (a) FeSAC/PI, (b) H-FeSAC/PI, (c) F-FeSAC/PI and (d) NH_2-FeSAC/PI system.

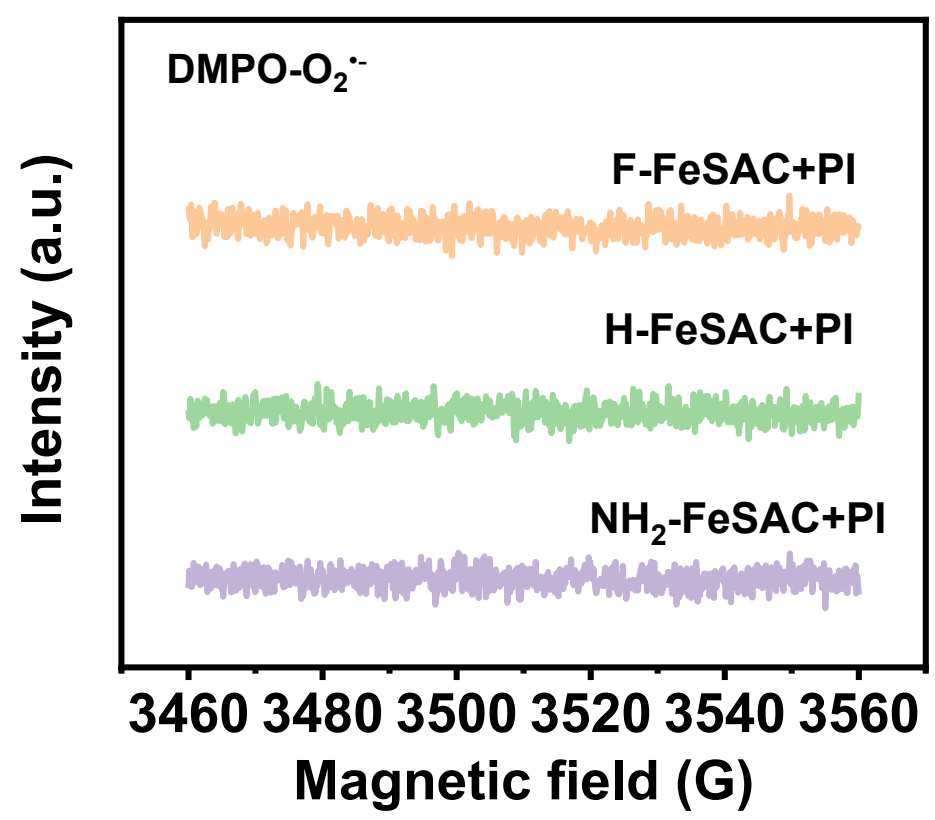

Supplementary Fig. 14 EPR spectra of $O_2^{\bullet-}$ trapped by DMPO in F-FeSAC/PI, H-FeSAC/PI, and NH_2-FeSAC/PI system.

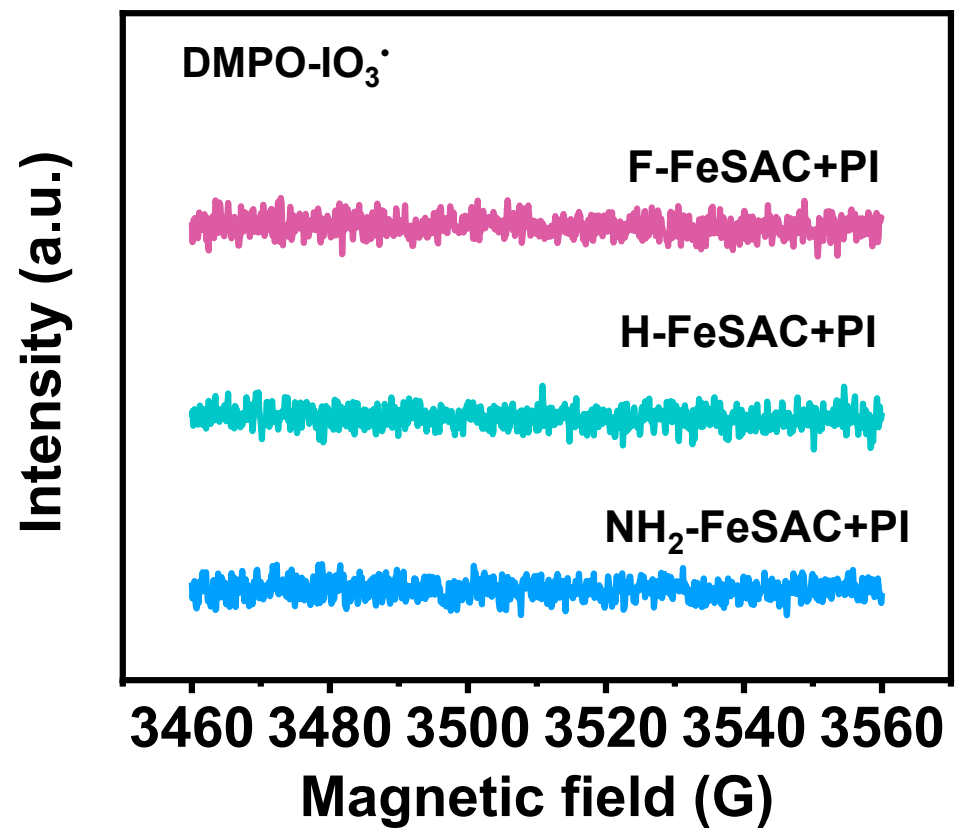

Supplementary Fig. 15 EPR spectra of $IO_3^{\bullet}$ trapped by DMPO in F-FeSAC/PI, H-FeSAC/PI, and NH_2-FeSAC/PI system.

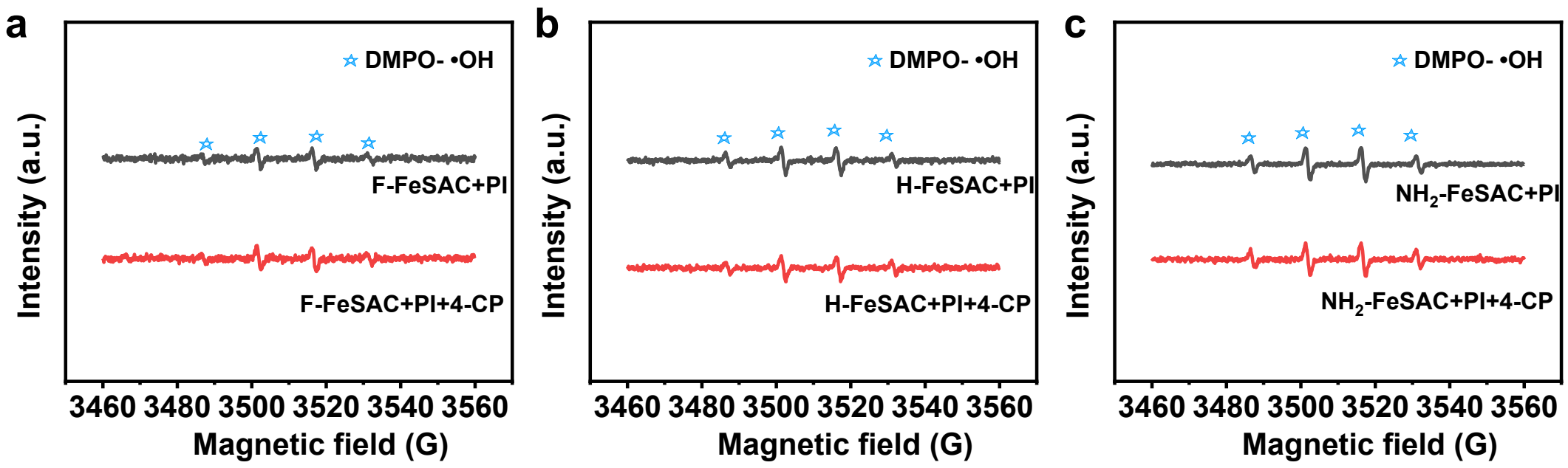

Supplementary Fig. 16 EPR spectra of •OH trapped by DMPO in (a) F-FeSAC/PI, (b) H-FeSAC/PI, and (c) NH_2-FeSAC/PI system.

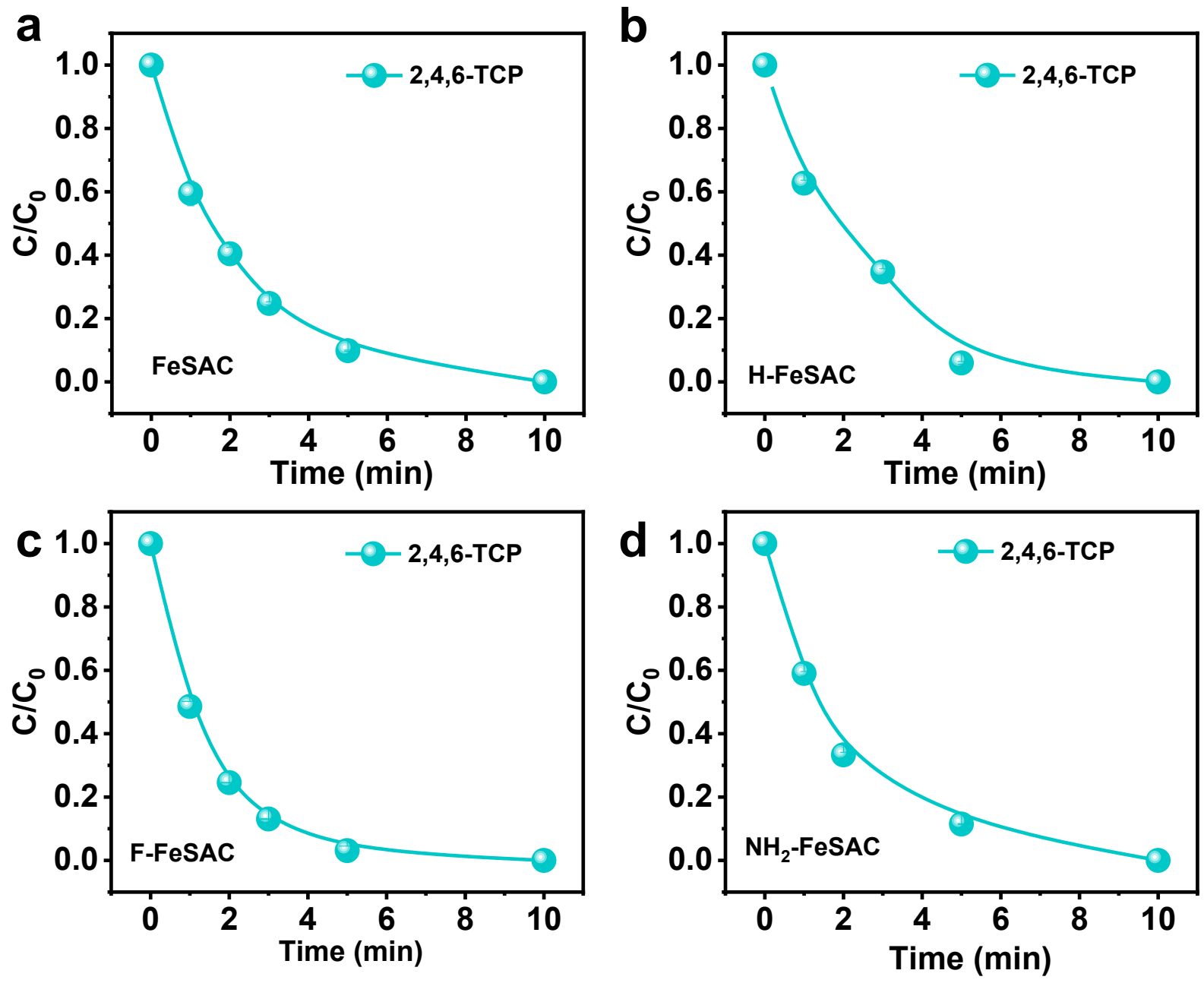

Supplementary Fig. 17 Degradation of 2,4,6-TCP in the (a) FeSAC/PI, (b) H-FeSAC/PI, (c) F-FeSAC/PI and (d) NH_2-FeSAC/PI system.

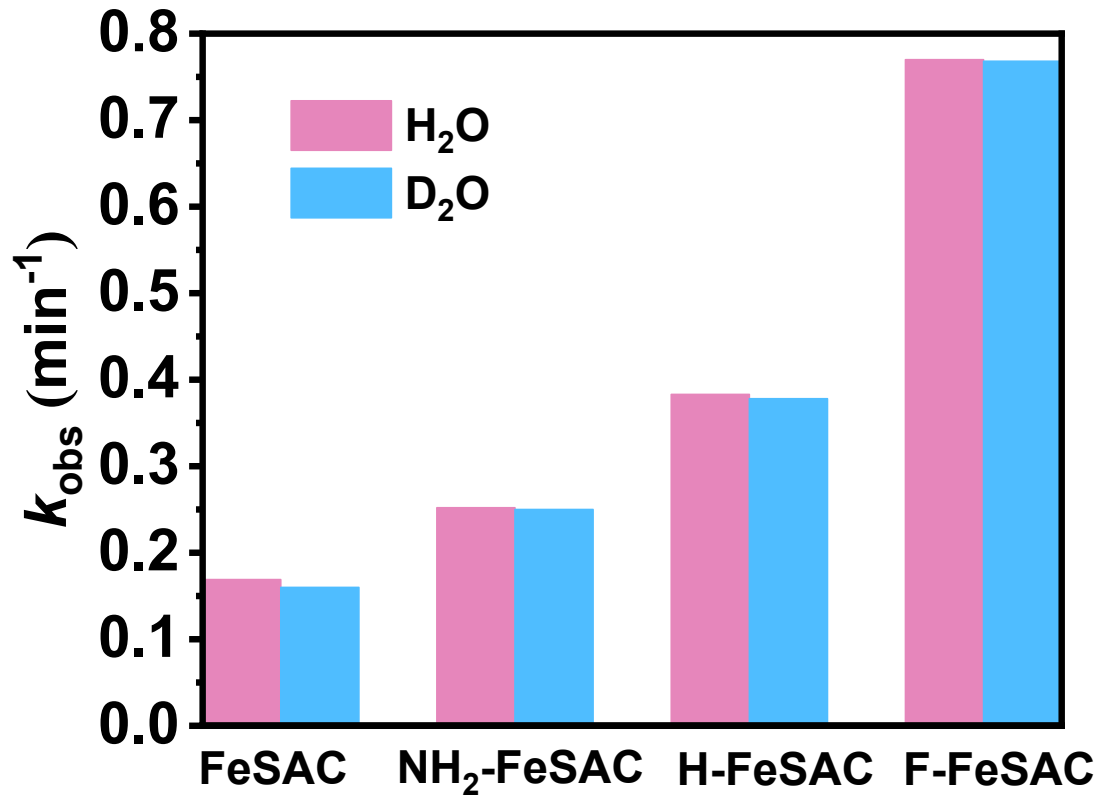

Supplementary Fig. 18 Effect of reaction solvents (H_2O and D_2O) on k_{obs} in different systems.

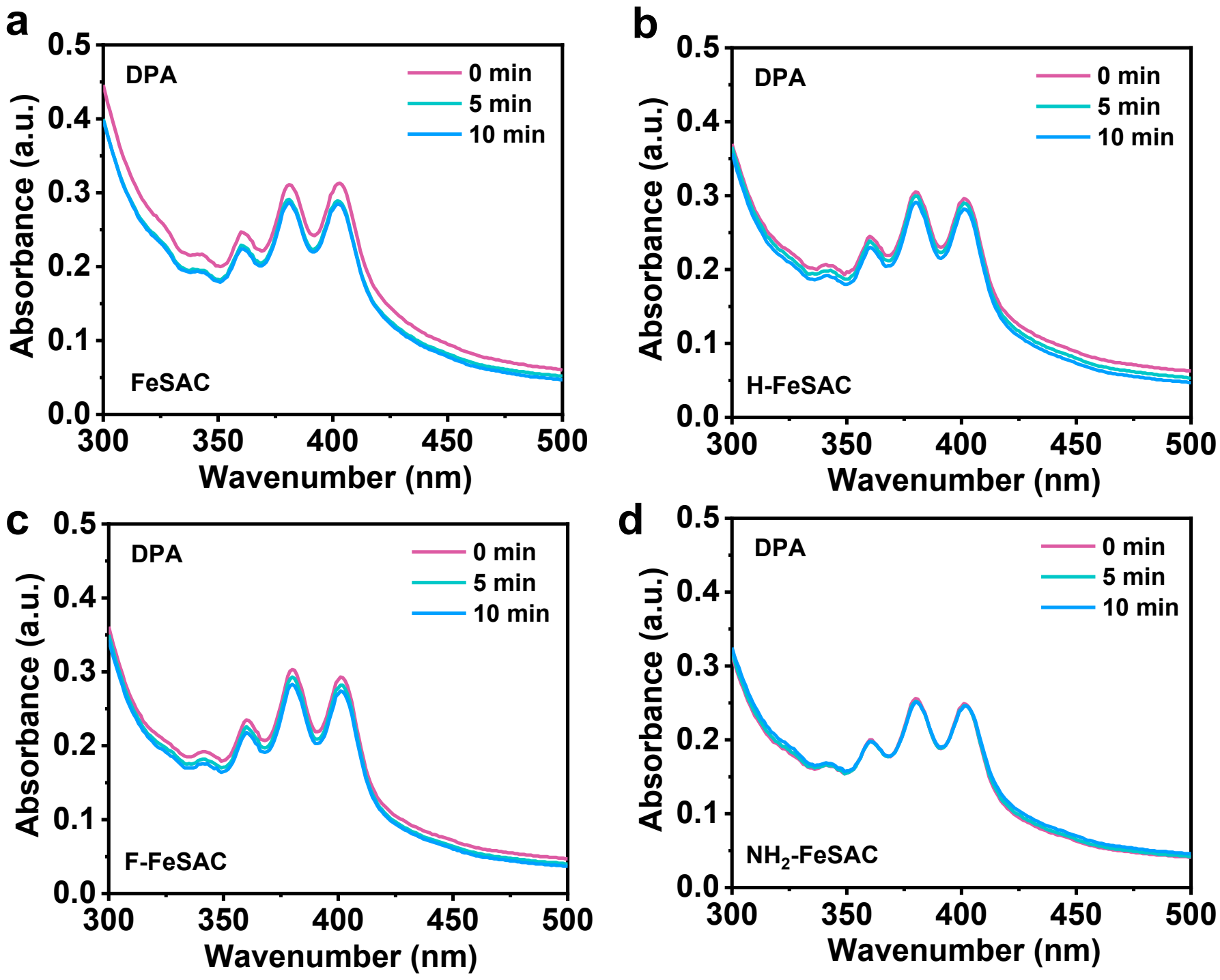

Supplementary Fig. 19 DPA signals in (a) FeSAC/PI, (b) H-FeSAC/PI, (c) F-FeSAC/PI and (d) NH_2-FeSAC/PI systems, Routine conditions: [4-CP] = 80 μM, [catalyst] = 50 mg/L, [PI] = 0.20 mM, temperature = 25 °C, without pH adjustment.

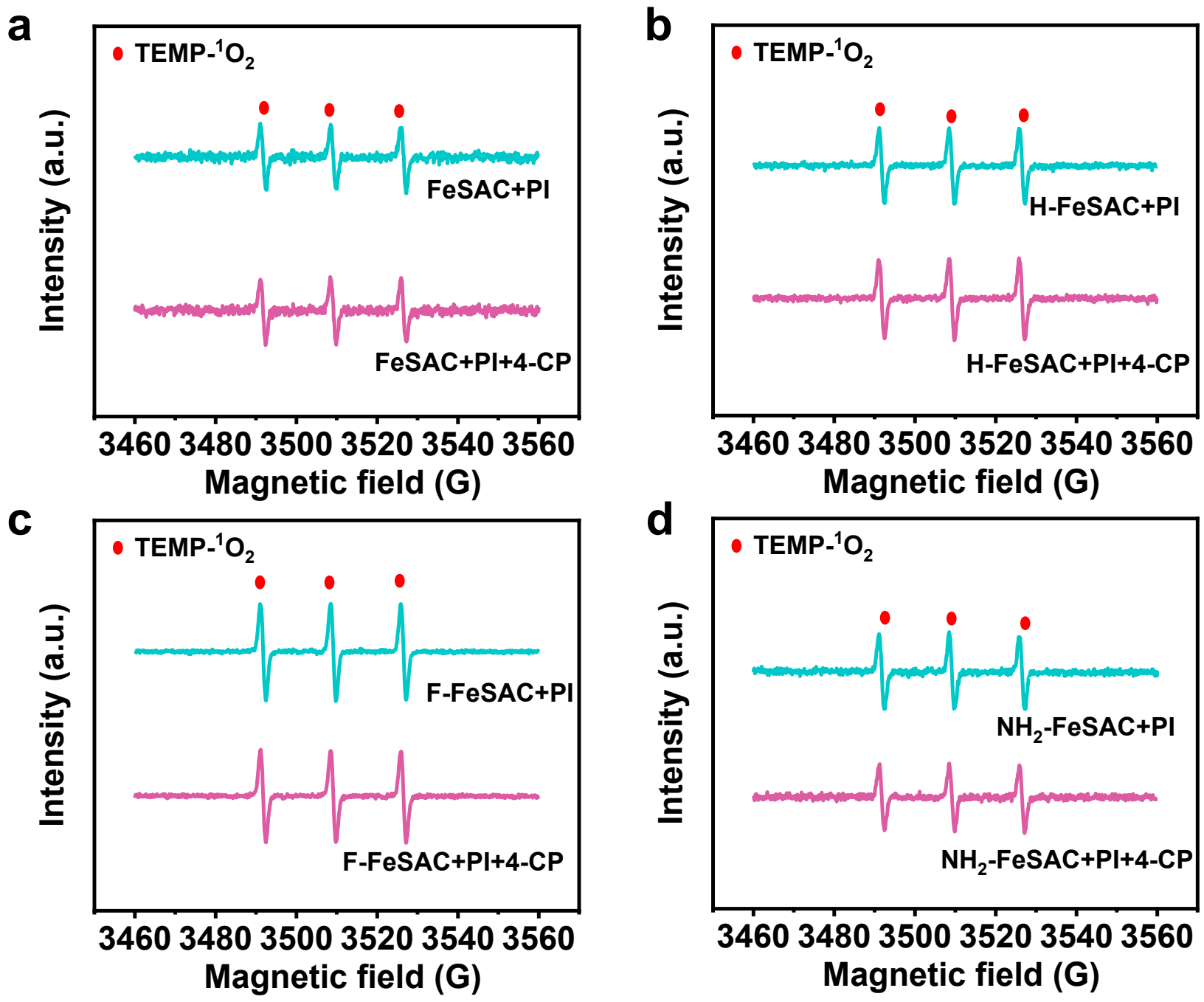

Supplementary Fig. 20 EPR spectra of $^1O_2$ trapped by TEMP in (a) FeSAC/PI, (b) H-FeSAC/PI, (c) F-FeSAC/PI and (d) NH_2-FeSAC/PI system.

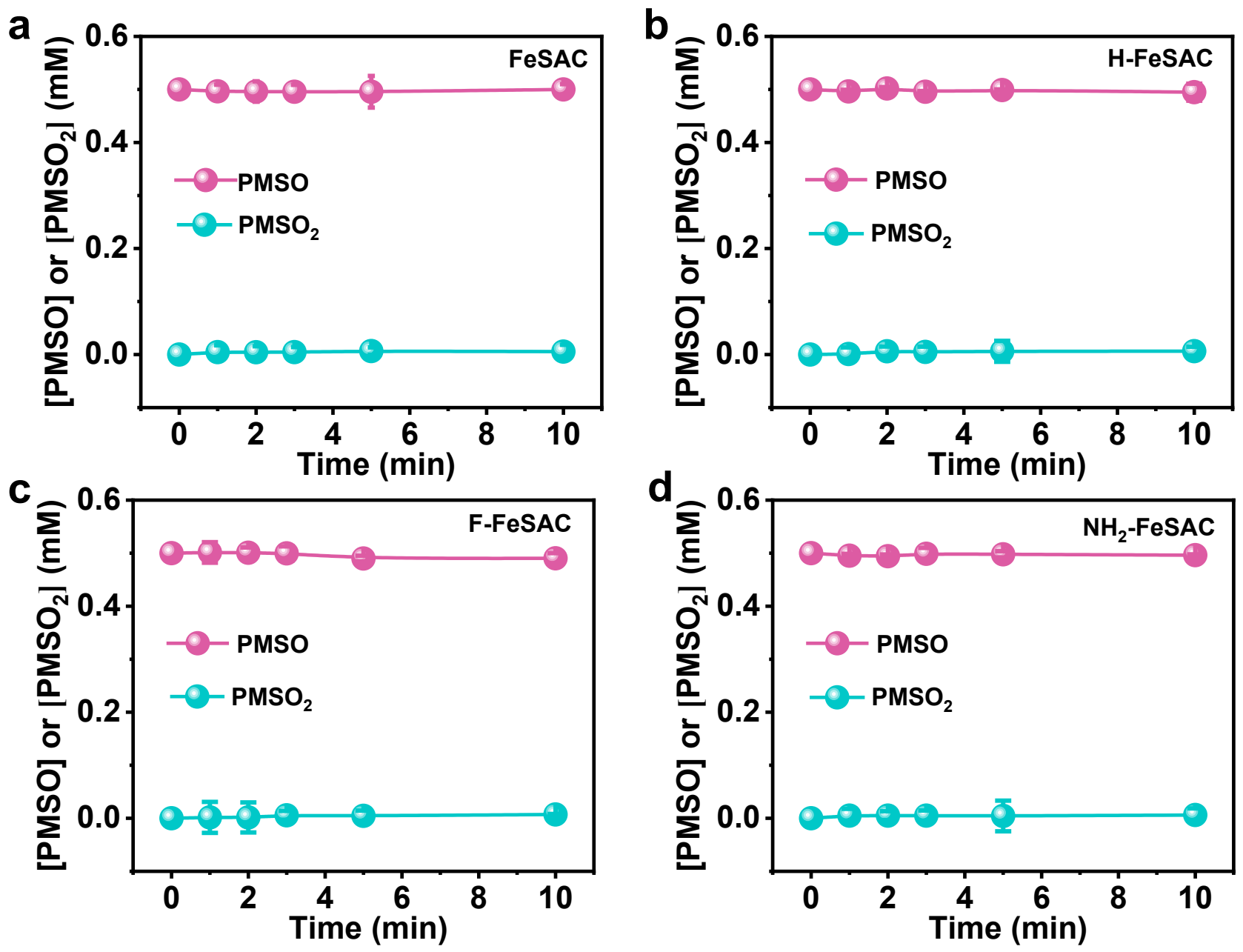

Supplementary Fig. 21 Concentration changes of PMSO and $PMSO_2$ in (a) FeSAC/PI, (b) H-FeSAC/PI, (c) F-FeSAC/PI and (d) NH_2-FeSAC/PI system.

Second, the premixing experiment provides additional support for a surface-mediated ETP pathway. When the catalysts and PI were premixed for different periods before the introduction of 4-CP, no discernible enhancement in 4-CP removal was

observed (**Fig. 3d; Supplementary Figs. 22a-c**). This result indicates that the oxidation process does not rely on the prior accumulation of freely diffusing reactive oxidizing species. Instead, pollutant oxidation is triggered when 4-CP is introduced into the catalyst/PI system, which is more consistent with a catalyst/PI interfacial process.

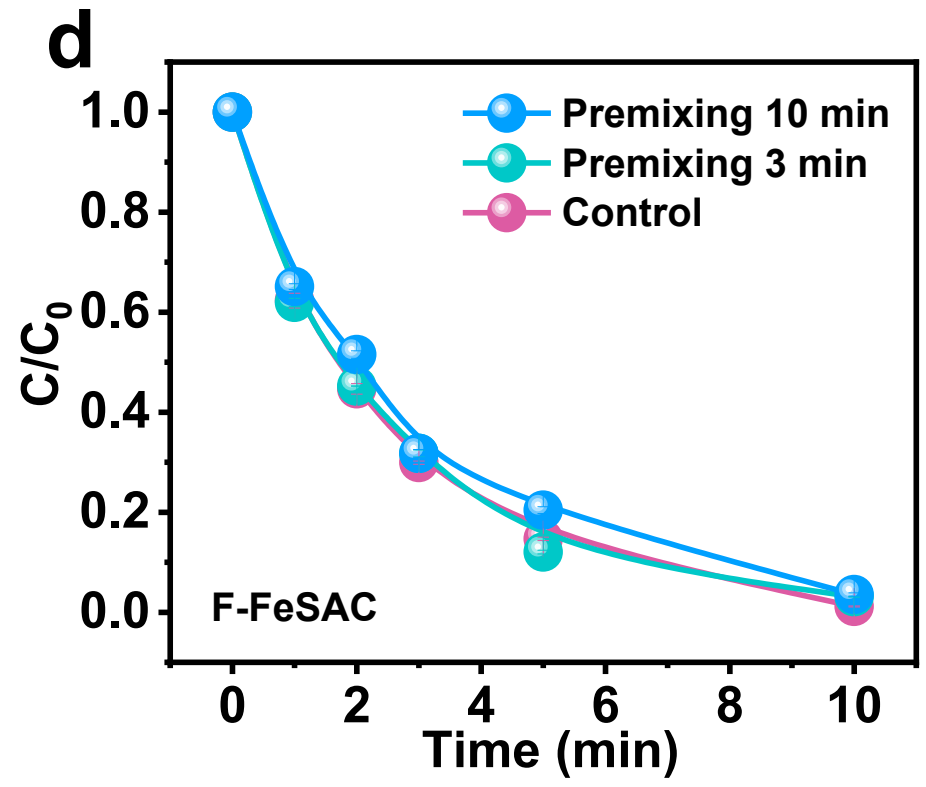

Fig. 3 (d) Effect of premixing on 4-CP removal in the F-FeSAC/PI system.

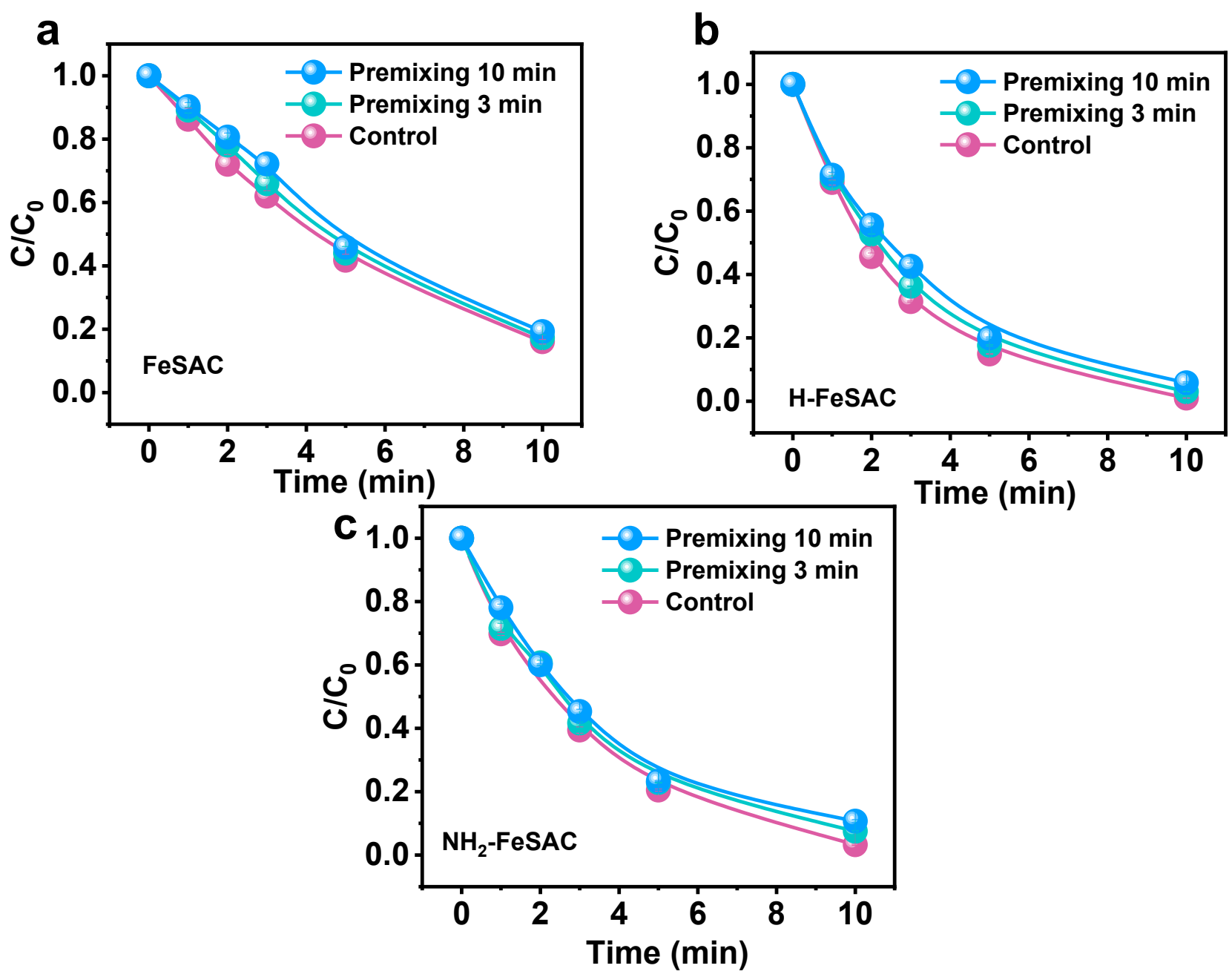

Supplementary Fig. 22 Effect of premixing in the (a) FeSAC/PI, (b) H-FeSAC/PI, (c) NH_2-FeSAC/PI system on 4-CP removal.

Third, oxidant consumption is tightly coupled with pollutant oxidation. In the absence of 4-CP, IO_4^- decomposition by R-FeSAC is limited, whereas the introduction of 4-CP immediately accelerates IO_4^- depletion (**Supplementary Fig. 23**). A strong linear correlation between 4-CP removal and IO_4^- consumption was observed, indicating that pollutant oxidation and oxidant reduction proceed synchronously (**Fig.**

3e). In addition, iodine-speciation analysis shows that IO_4^- is mainly converted to IO_3^- without detectable accumulation of HOI, I^-, or I_3^- (**Fig. 6a-c**). These results indicate that PI functions as the terminal electron-accepting oxidant in the reaction process rather than serving primarily as a source of freely diffusing reactive iodine species.

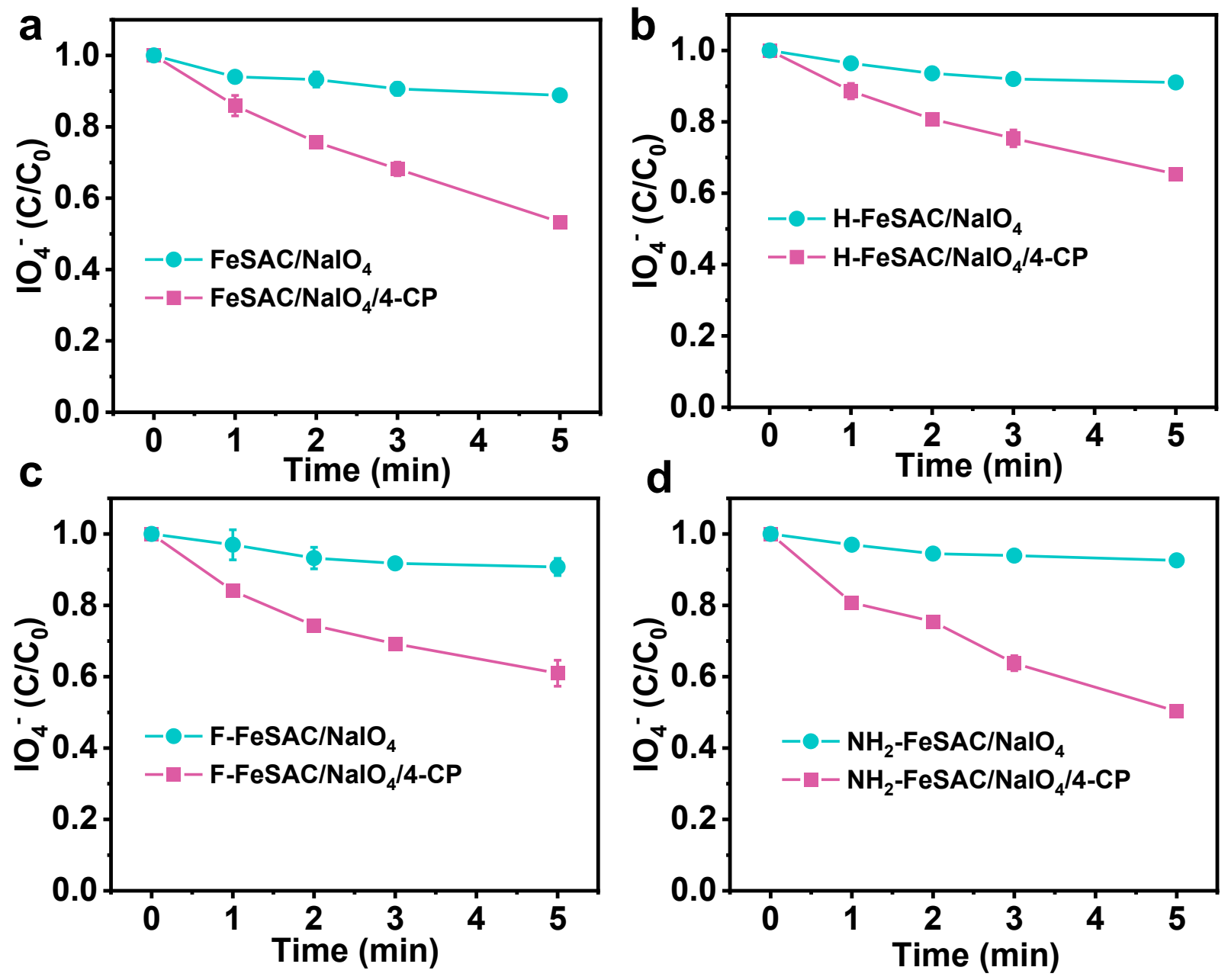

Supplementary Fig. 23 Decomposition rate of PI in (a) FeSAC/PI, (b) H-FeSAC/PI, (c) F-FeSAC/PI and (d) NH_2-FeSAC/PI system.

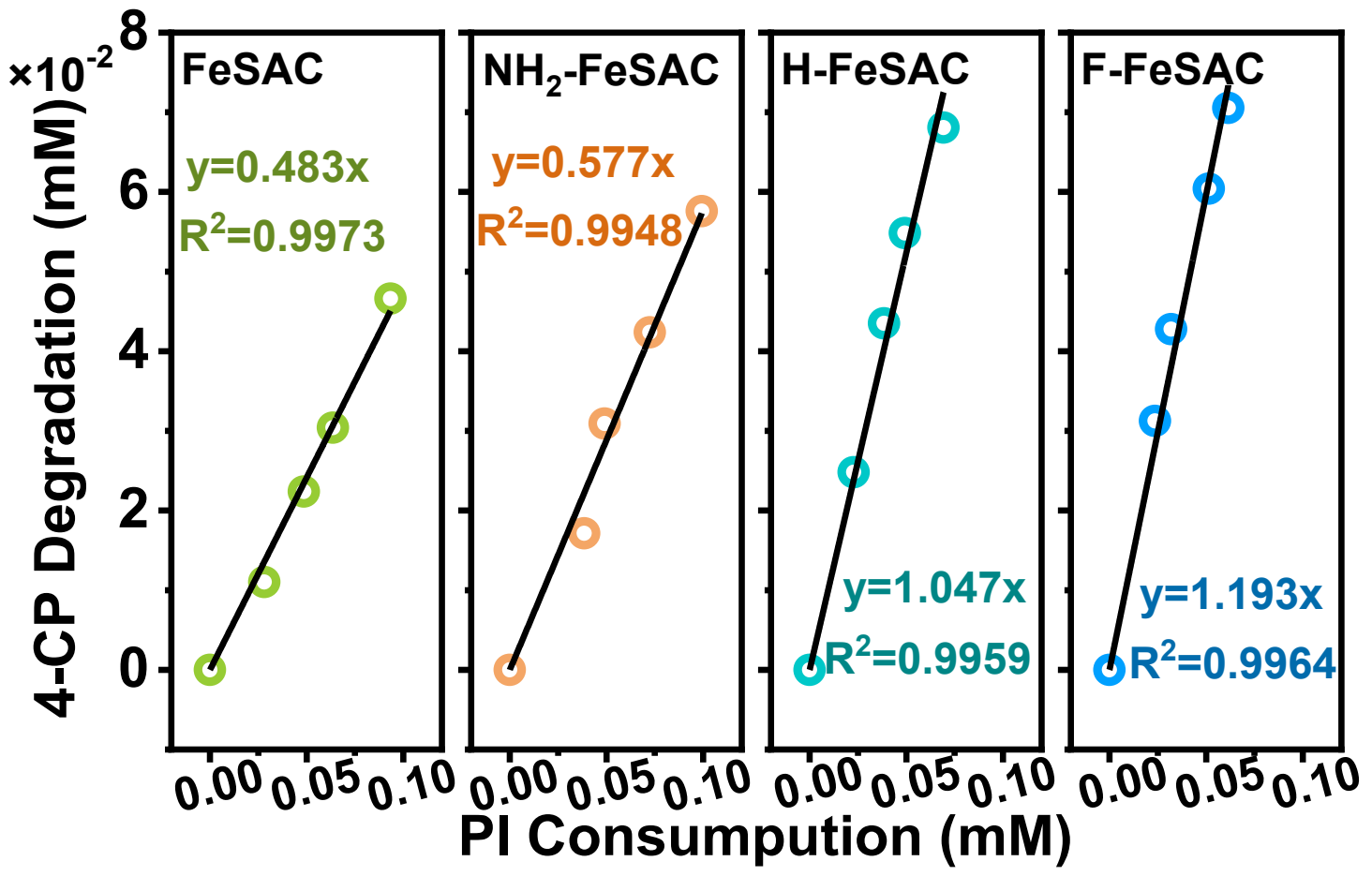

Fig. 3 (e) Correlation between 4-CP degradation and PI consumption in FeSAC/PI, NH_2-FeSAC/PI, H-FeSAC/PI and F-FeSAC/PI systems.

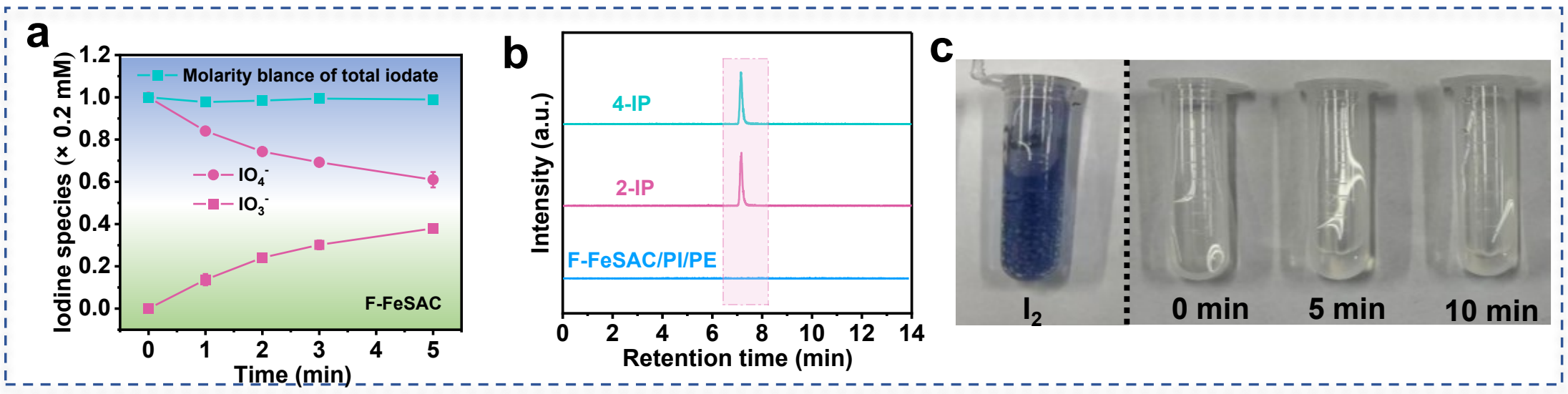

Fig. 6 (a) Concentration changes of IO_4^- and IO_3^- in the F-FeSAC/PI/4-CP system. (b) Detection of HOI generated in the F-FeSAC/PI system using phenol as a trapping agent. (c) Detection of I_2 and I_3^- in the F-FeSAC/PI system.

Fourth, in situ Raman spectroscopy supports the formation and consumption of surface catalyst/PI complexes. Upon PI addition to F-FeSAC, a new PI-related vibrational feature appears at approximately 830 cm^{-1}, indicating specific adsorption or coordination of PI on the catalyst surface. After 4-CP introduction, the corresponding PI-related Raman signal decreases, suggesting that the surface-associated catalyst/PI species is consumed during pollutant oxidation (**Fig. 3f**). This behavior is consistent with a surface-mediated electron-transfer process.

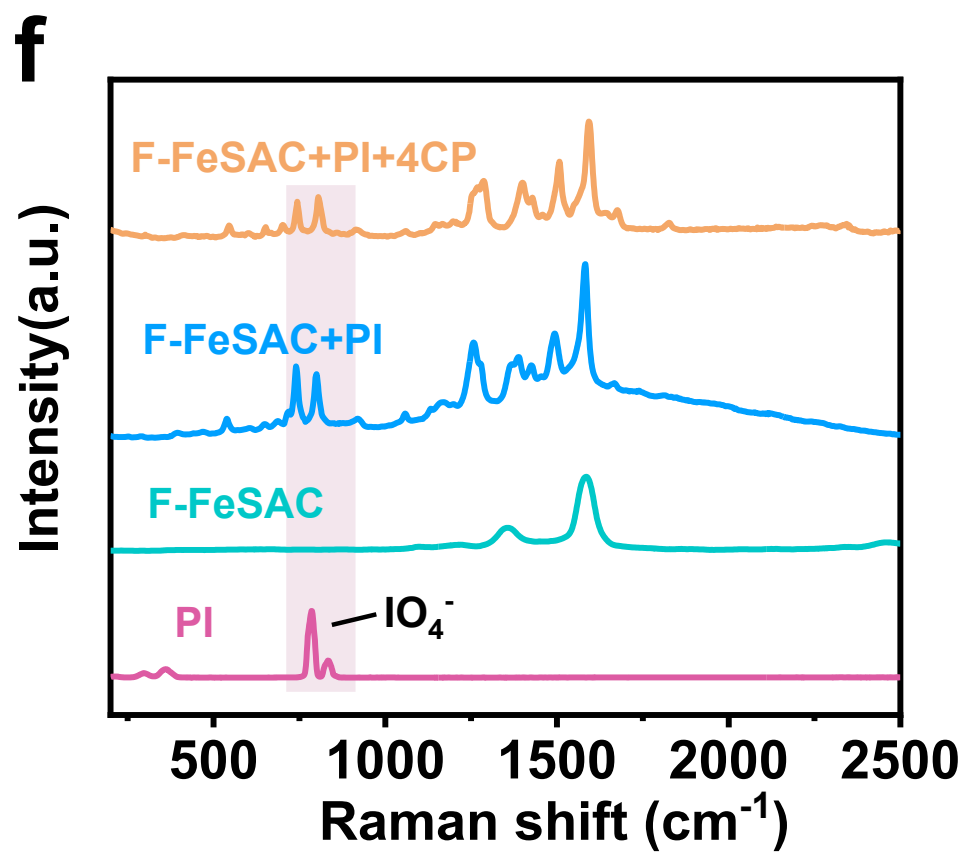

Fig. 3 (f) In situ Raman spectroscopy of different systems.

Fifth, electrochemical measurements further support the proposed ETP mechanism. The OCP, chronoamperometry, LSV, and EIS results collectively indicate the formation of an electroactive catalyst/PI interface and enhanced interfacial charge transfer after pollutant addition (**Fig. 3g; Supplementary Figs.24-26**). Specifically, PI addition induces a clear electrochemical response associated with catalyst/PI interfacial complex formation, while subsequent 4-CP addition triggers additional current responses related to pollutant-involved charge transfer. Among the R-FeSAC catalysts, F-FeSAC shows the strongest electrochemical response and the lowest charge-transfer resistance,

consistent with its highest catalytic activity.

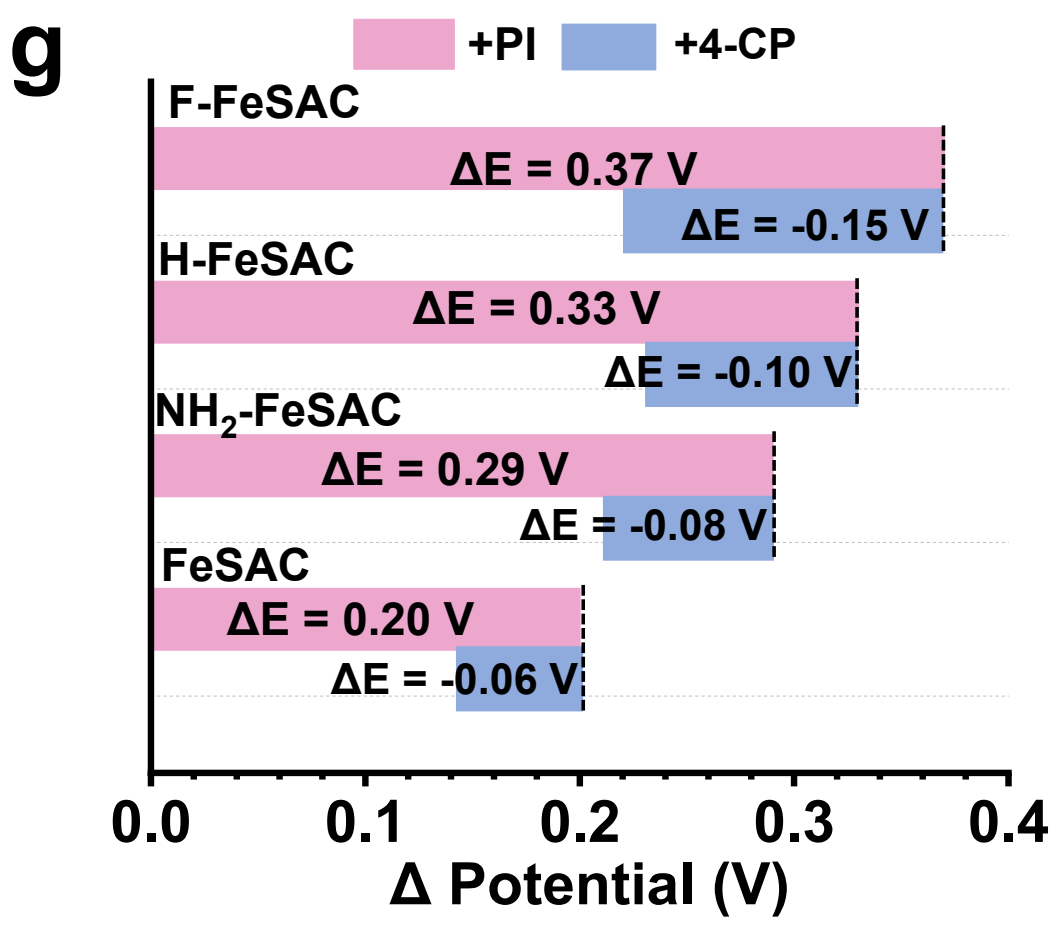

Fig. 3 (g) OCP changes of different systems with the addition of PI and 4-CP.

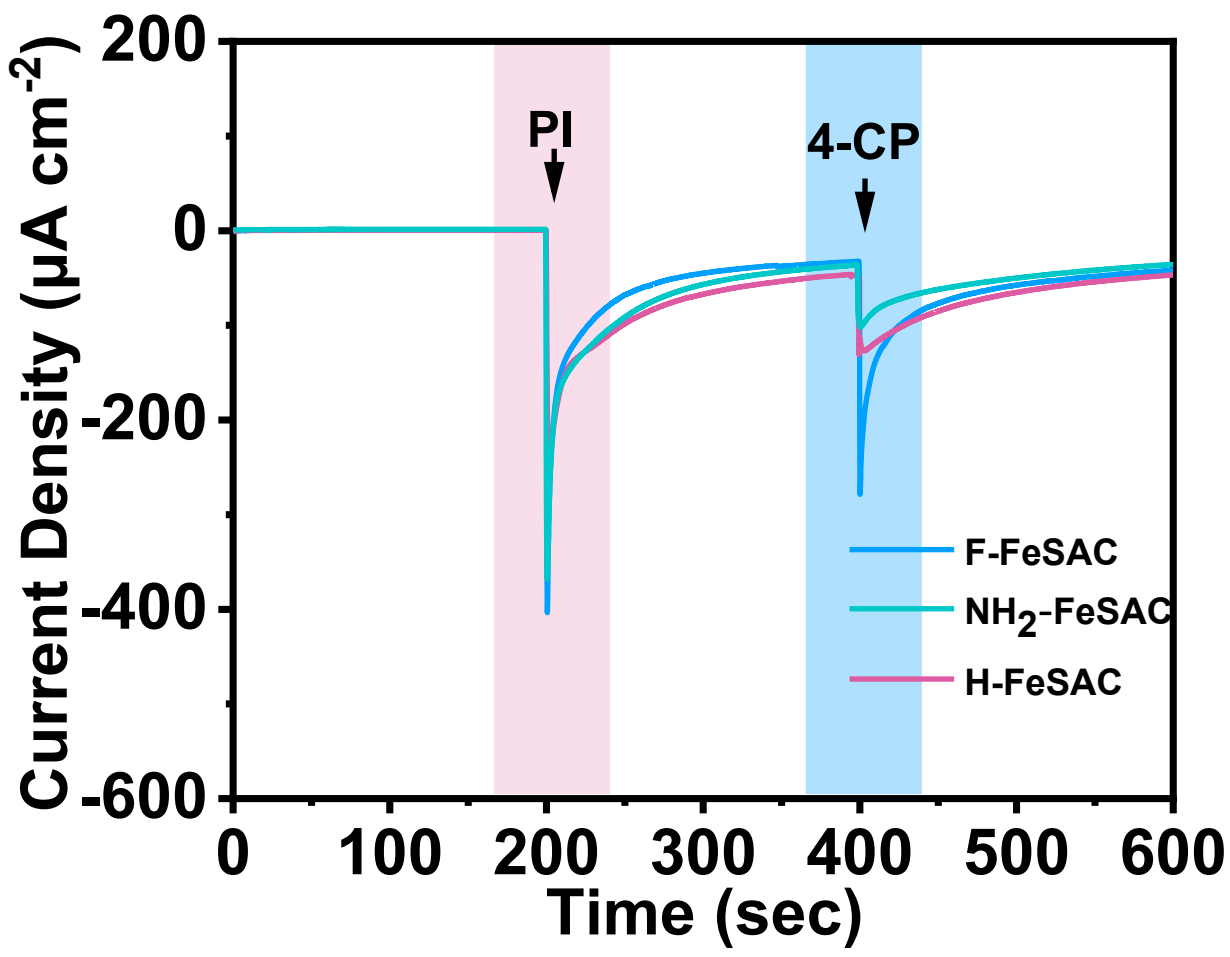

Supplementary Fig. 24 i-t curve of F-FeSAC, H-FeSAC and NH_2-FeSAC after adding PI and 4-CP successively.

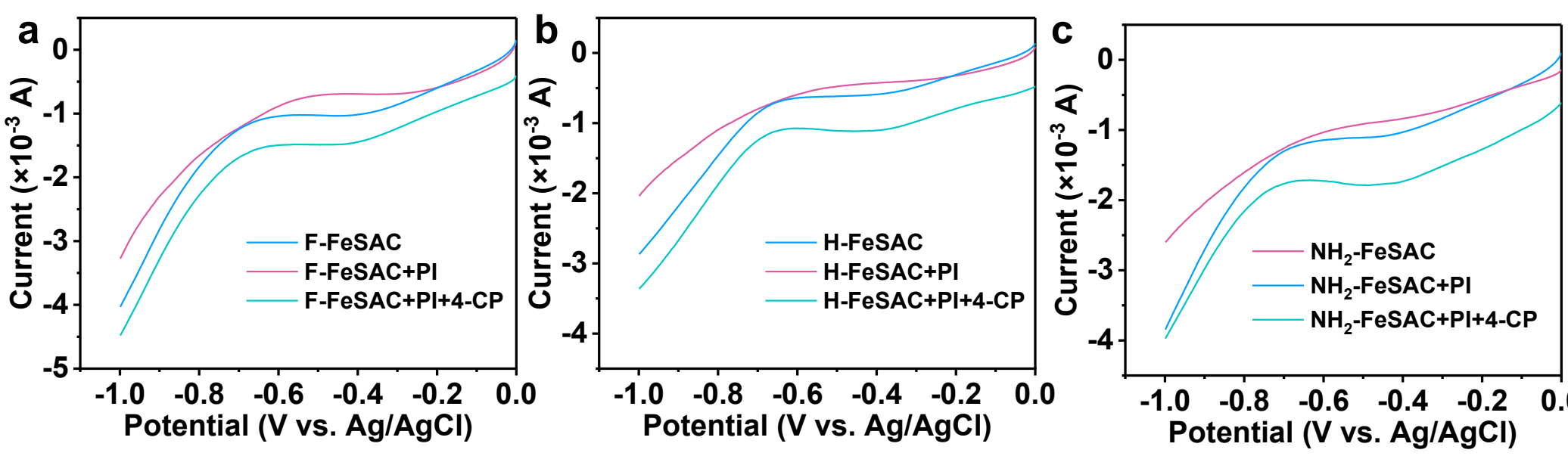

Supplementary Fig. 25 Linear voltammetry curves (LSV) curve of (a) F-FeSAC, (b) H-FeSAC and (c) NH_2-FeSAC after adding PI and 4-CP successively.

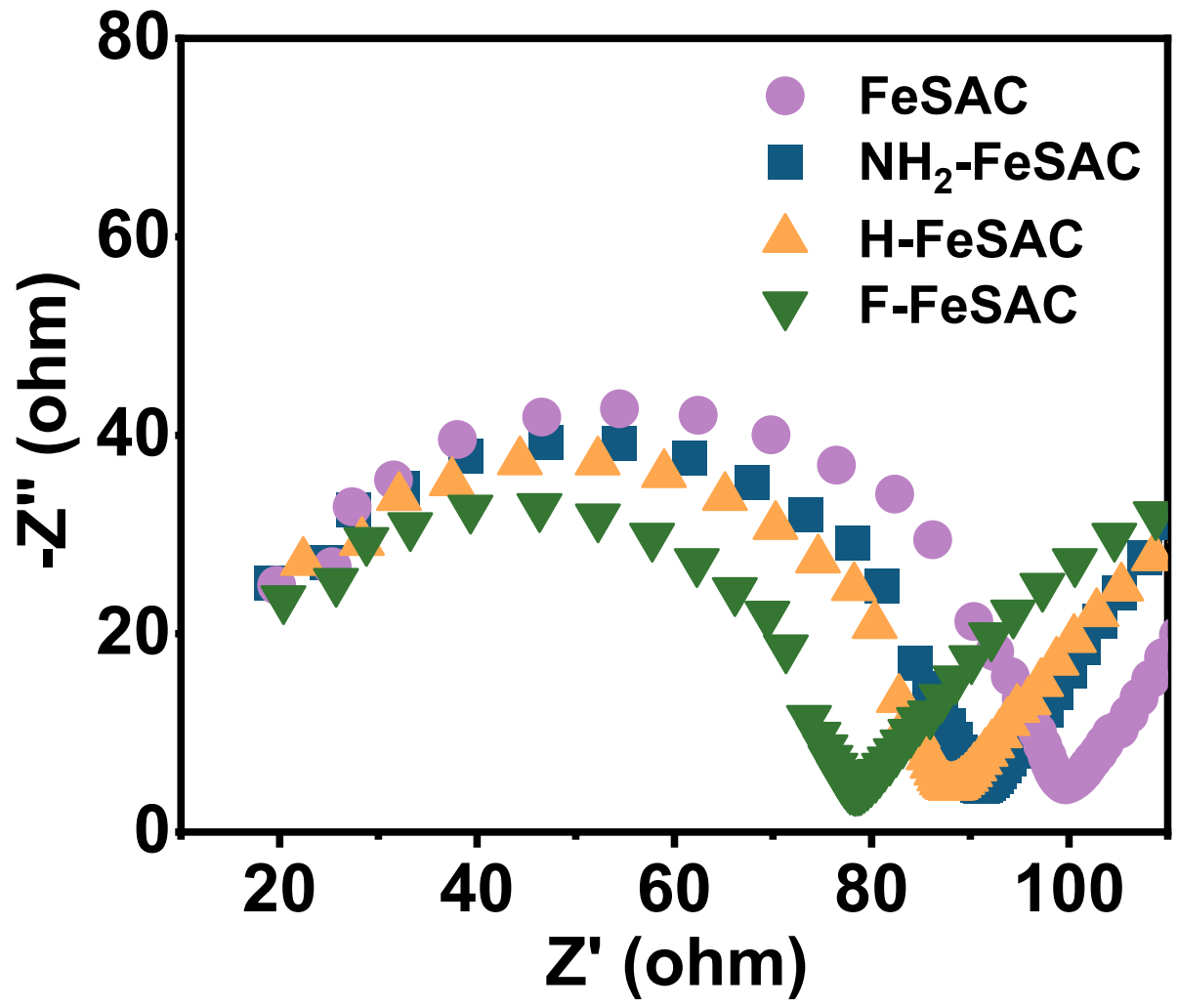

Supplementary Fig. 26 EIS analyses spectra of FeSAC, F-FeSAC, H-FeSAC and NH_2-FeSAC.

Sixth, the galvanic oxidation process (GOP) experiment provides direct mechanistic support for electron-transfer-mediated oxidation. In the dual-chamber reactor separated by a proton exchange membrane, PI and 4-CP are physically isolated and can communicate only through the external circuit. Under these conditions, the F-FeSAC-based system still generates a clear current response and achieves significant 4-CP oxidation, whereas the unmodified control system shows negligible activity (**Fig. 3h–j; Supplementary Figs. 27–28**). This spatially separated configuration excludes direct molecular contact between PI and 4-CP and strongly supports an electron-transfer-mediated oxidation pathway.

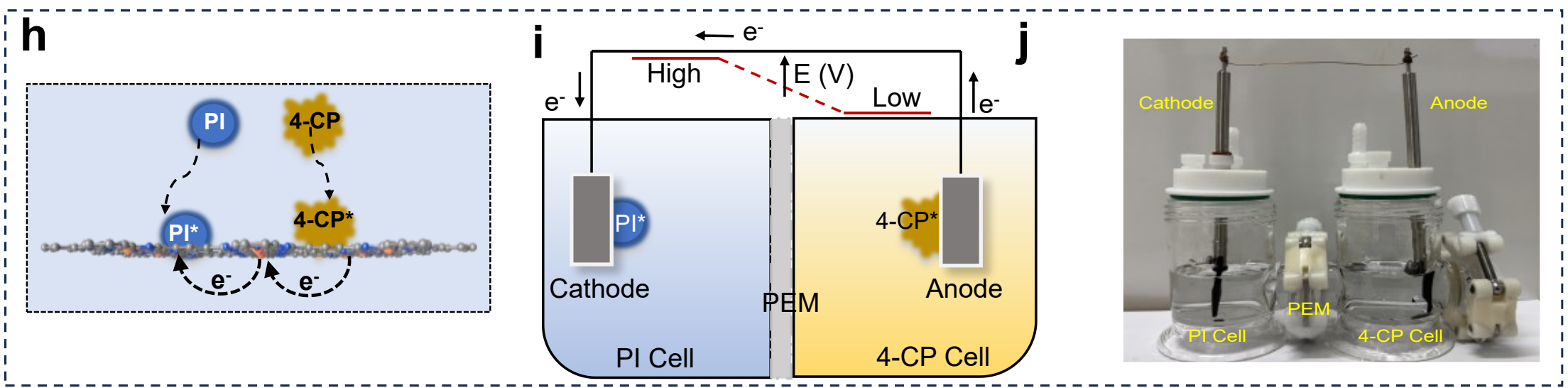

Fig. 3 (h) Schematic illustration of the electron transfer process. (i) Schematic diagram of the concept design and (j) photograph of the double-chamber reactor developed for R-FeSAC/GF performance evaluation.

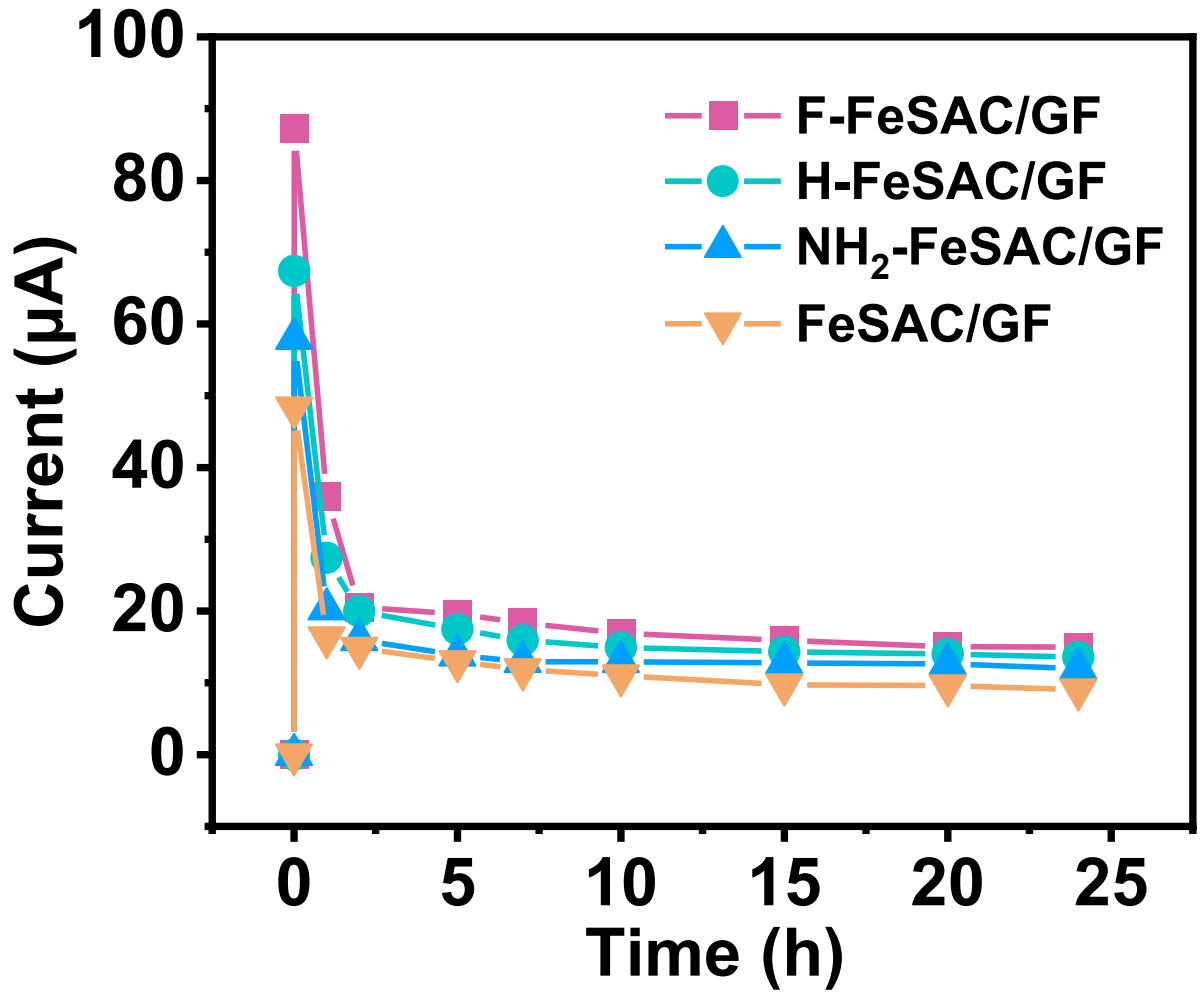

Supplementary Fig. 27 Measurement of current-time curves on the FeSAC/GF, F-FeSAC/GF, H-FeSAC/GF and NH_2-FeSAC/GF electrode.

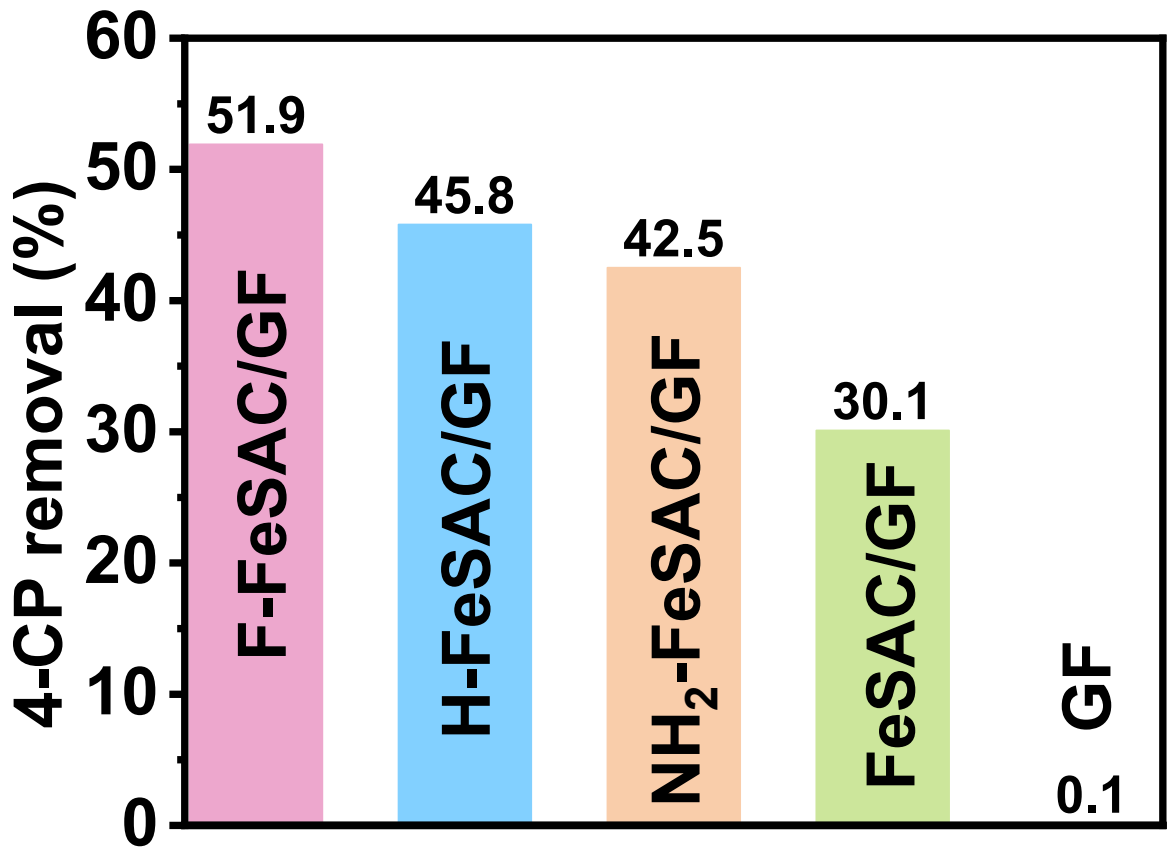

Supplementary Fig. 28 Removal rates of 4-CP in different GOP systems.

Seventh, the pollutant-dependent reactivity is also consistent with an ETP mechanism. The degradation rates of representative pollutants correlate with their electron-donating ability, as reflected by their half-wave potentials. Electron-rich pollutants are oxidized much more rapidly than electron-poor substrates such as nitrobenzene and benzoic acid (**Fig. 3k**). This selectivity is inconsistent with radical oxidation but is consistent with an electron-transfer-controlled process.

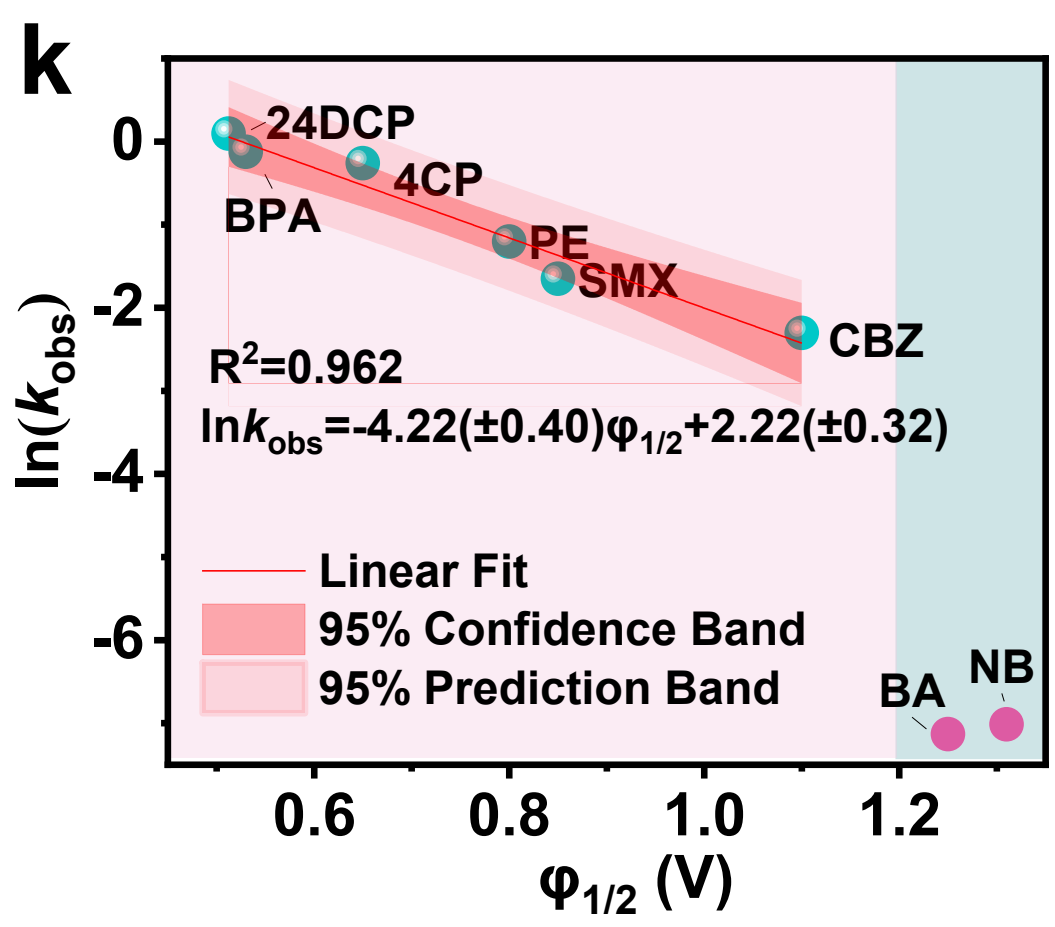

Fig. 3 (k) Correlation between the $\varphi_{1/2}$ values of eight contaminants and their ln(k_{obs}).

Finally, DFT calculations further support directional interfacial electron transfer (**Fig. 4a–h**). HOMO/LUMO analysis, PDOS, charge-density-difference analysis, adsorption-energy calculations, Fe–O bond-length analysis, and charge-transfer results collectively show that F substitution promotes stronger PI adsorption, more pronounced interfacial charge redistribution, and a more favorable electronic alignment between the catalyst/PI complex and organic pollutants. These theoretical results are consistent with the experimentally observed activity trend and electrochemical behavior.

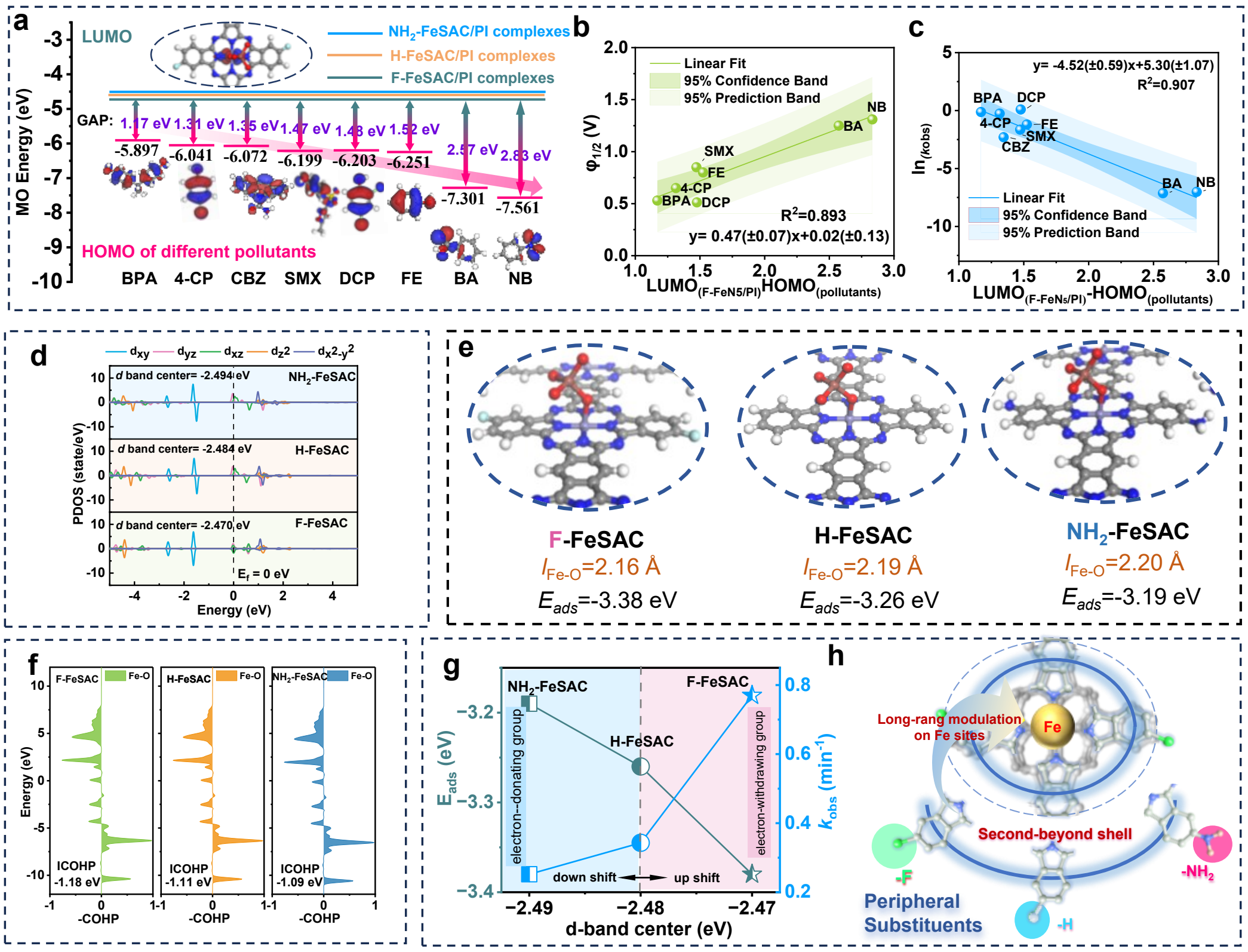

Fig. 4 | The mechanistic of substituents beyond the second coordination sphere in

regulating Fe active sites. (a) Energy gap between $LUMO_{(R\text{-}FeSAC/PI)}$ and $HOMO_{pollutants}$. (b) Linear correlation between $LUMO_{(R\text{-}FeSAC/PI)}$-$HOMO_{pollutants}$ and $\varphi_{1/2}$ values of pollutants. (c) Linear correlation between $LUMO_{(R\text{-}FeSAC/PI)}$-$HOMO_{pollutants}$ and $\ln(k_{obs})$. (d) Projected Density of States of the Fe 3d orbitals in F-FeSAC, H-FeSAC, and NH_2-FeSAC. (e) Optimized adsorption configurations of PI at Fe site on the F-FeSAC, H-FeSAC, and NH_2-FeSAC. (f) Crystal orbital Hamilton population (COHP) analysis of Fe-O when PI adsorbs on Fe. (g) Correlations among k_{obs}, E_{ads}, and *d*-band center for F-FeSAC, H-FeSAC, and NH_2-FeSAC. (h) Schematic illustration of long-range electronic modulation at Fe sites induced by substituent groups beyond the secondary coordination sphere.

Following the reviewer's specific suggestion, we further added a comparison between IO_3^- and $IO_3^•$ to clarify the roles of stable iodate ions and transient iodate radicals in the reaction network.

Exclusion of IO_3^- as an active oxidant

To determine whether IO_3^-, the final reduction product of periodate, could directly contribute to 4-CP oxidation, we performed comparative degradation experiments using IO_3^- in place of IO_4^-. As shown in the **Fig. R27**, both the IO_3^-/ 4-CP and F-FeSAC/IO_3^-/4-CP systems exhibited negligible 4-CP removal, whereas rapid degradation was observed in the F-FeSAC/IO_4^-system. These results clearly demonstrate that IO_3^- is not an effective oxidant under the present conditions and therefore cannot account for the observed 4-CP oxidation. Thus, IO_3^- is more appropriately assigned as a stable reduction product of PI rather than an active oxidizing species in this system.

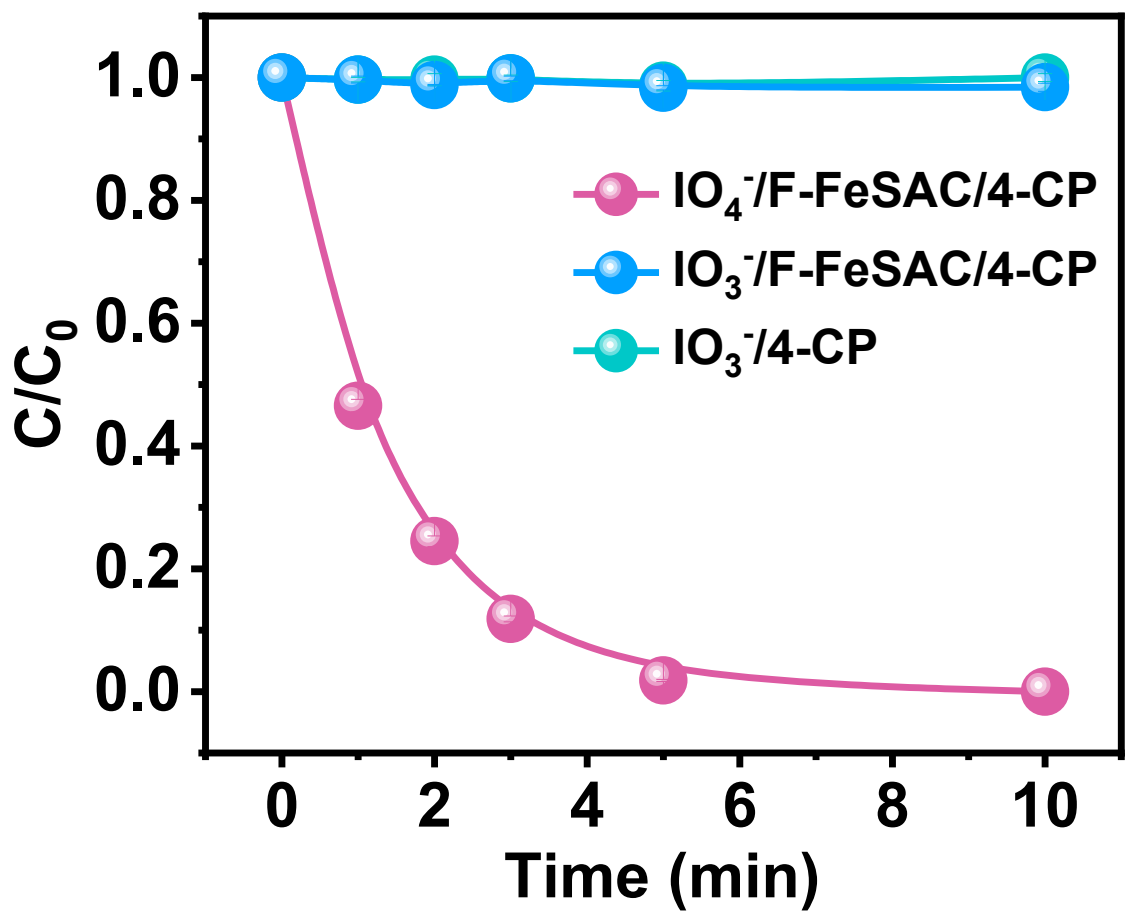

Fig. R27 Effects of different systems on the degradation of 4-CP.

Evaluation of $IO_3^\bullet$ involvement in the catalytic system

We then examined whether $IO_3^\bullet$ was generated in the R-FeSAC/IO_4^- systems using DMPO-based EPR spectroscopy. As shown in the **Fig. R17**, no discernible DMPO-$IO_3^\bullet$ signal was detected in the F-FeSAC/IO_4^-, H-FeSAC/IO_4^-, or NH_2-FeSAC/IO_4^- systems. This result suggests that $IO_3^\bullet$ is not the dominant radical species driving 4-CP oxidation in the catalytic systems.

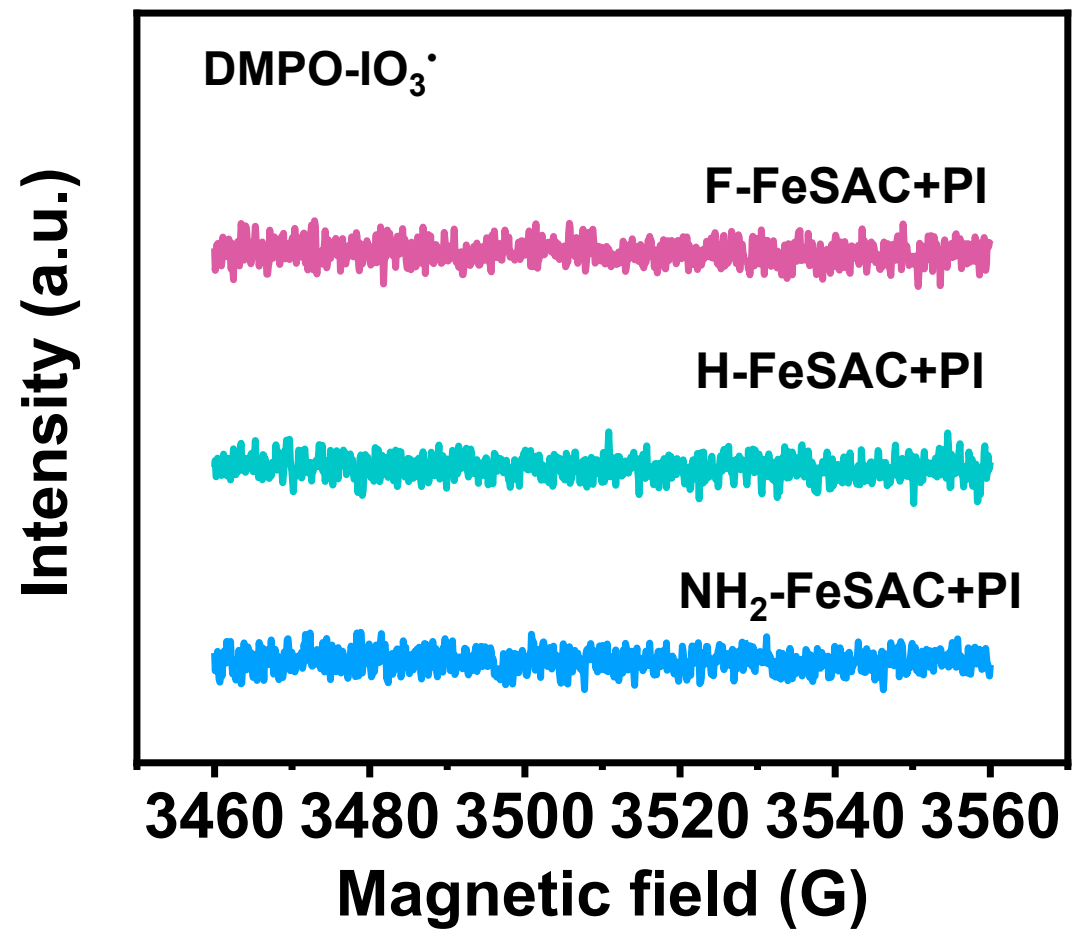

Fig. R17 EPR spectra of $IO_3^\bullet$ trapped by DMPO in F-FeSAC/PI, H-FeSAC/PI, and NH_2-FeSAC/PI system.

Validation of EPR detection sensitivity (positive reference)

To verify that the absence of DMPO–$IO_3^\bullet$ signals was not caused by insufficient detection sensitivity, we performed a positive reference experiment using the UV/IO_4^-/DMPO system[60]. This system has been reported to generate detectable $IO_3^\bullet$ signals with characteristic hyperfine splitting features. As shown in **Fig. R28**, a clear DMPO–$IO_3^\bullet$ signal was observed under UV irradiation, whereas no such signal was detected in the dark control. Notably, 4-CP addition markedly weakened the $IO_3^\bullet$ signal, confirming that our EPR analysis is sufficiently sensitive to detect $IO_3^\bullet$ and monitor its reaction with 4-CP.

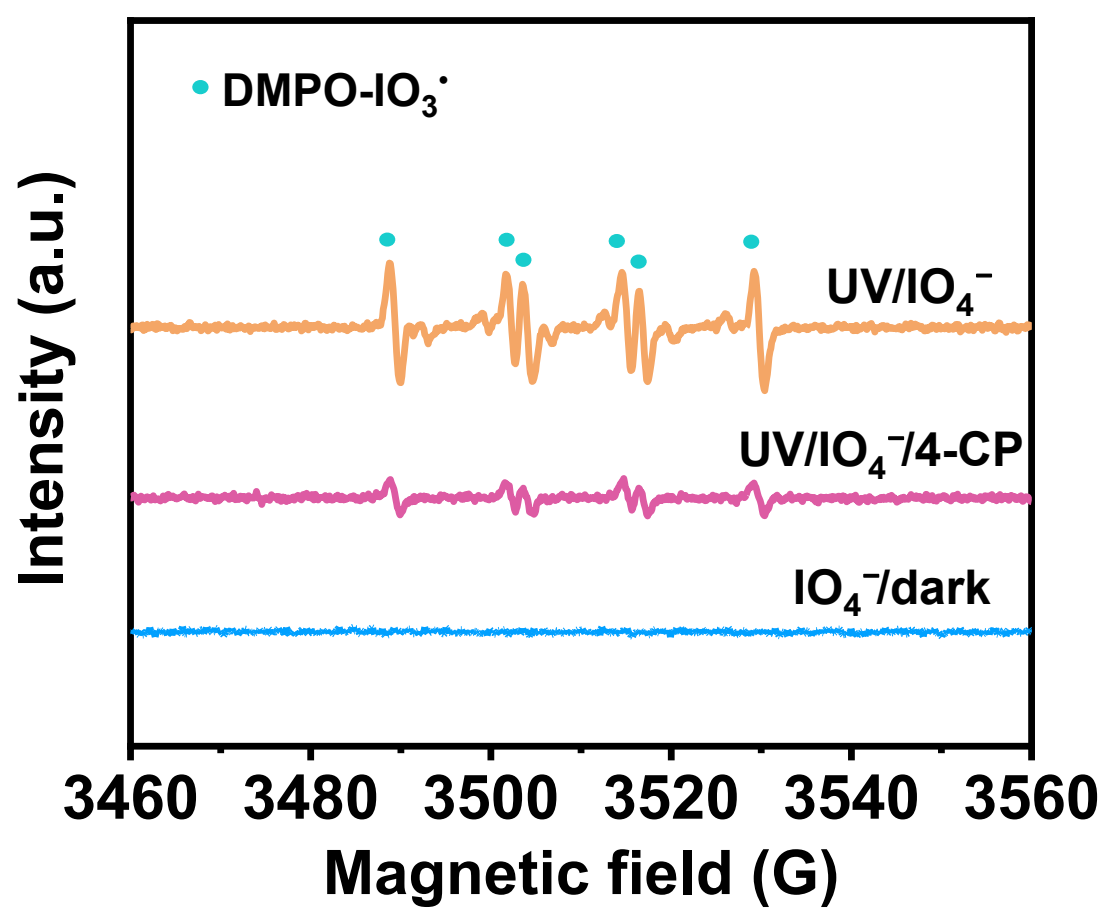

Fig. R28 EPR spectra of $IO_3^{\bullet}$ trapped by DMPO in different systems.

Taken together, the IO_3^- control experiments show that stable iodate ions are not direct oxidants for 4-CP, while the UV/IO_4^- positive reference confirms that $IO_3^{\bullet}$ can be detected under our EPR conditions when it is generated. In contrast, no detectable $IO_3^{\bullet}$ signal was observed in the R-FeSAC/IO_4^- catalytic systems. Therefore, the rapid degradation of 4-CP in the F-FeSAC/IO_4^- system cannot be attributed to either IO_3^- oxidation or $IO_3^{\bullet}$-mediated radical oxidation. More importantly, this conclusion is consistent with the broader mechanistic evidence, including the premixing experiments, synchronous PI consumption and 4-CP removal, in situ Raman spectroscopy, electrochemical measurements, GOP experiments, pollutant-dependent reactivity, and DFT calculations. Collectively, these results support that 4-CP oxidation proceeds predominantly through a catalyst/PI-mediated non-radical electron-transfer pathway.

8. Regarding the PEM analysis, did the authors verify that no species crossover occurred after the measurement?

Response 8:

We thank the reviewer for raising this important point. We agree that verifying species crossover is essential for interpreting the PEM-separated GOP experiment. To address this concern, we performed additional post-reaction analyses of the solutions collected from both chambers. The concentrations of IO_4^- and IO_3^- were measured to evaluate the possible crossover of inorganic iodine species. In parallel, LC-MS analysis was conducted to determine whether 4-CP or its transformation products migrated from the pollutant chamber to the oxidant chamber.

The results showed that IO_4^- and IO_3^- were predominantly retained in the PI chamber, with negligible signals detected in the 4-CP chamber (**Fig. R29**). Meanwhile, LC-MS analysis did not detect 4-CP or its related transformation products in the PI chamber

(**Fig. R30**). These results confirm that crossover of both iodine species and organic compounds was negligible under the tested conditions.

Therefore, the 4-CP oxidation observed in the GOP experiment cannot be ascribed to direct contact between 4-CP and PI or to inter-chamber mixing. Instead, these results support the proposed electron-transfer process mediated through the external circuit.

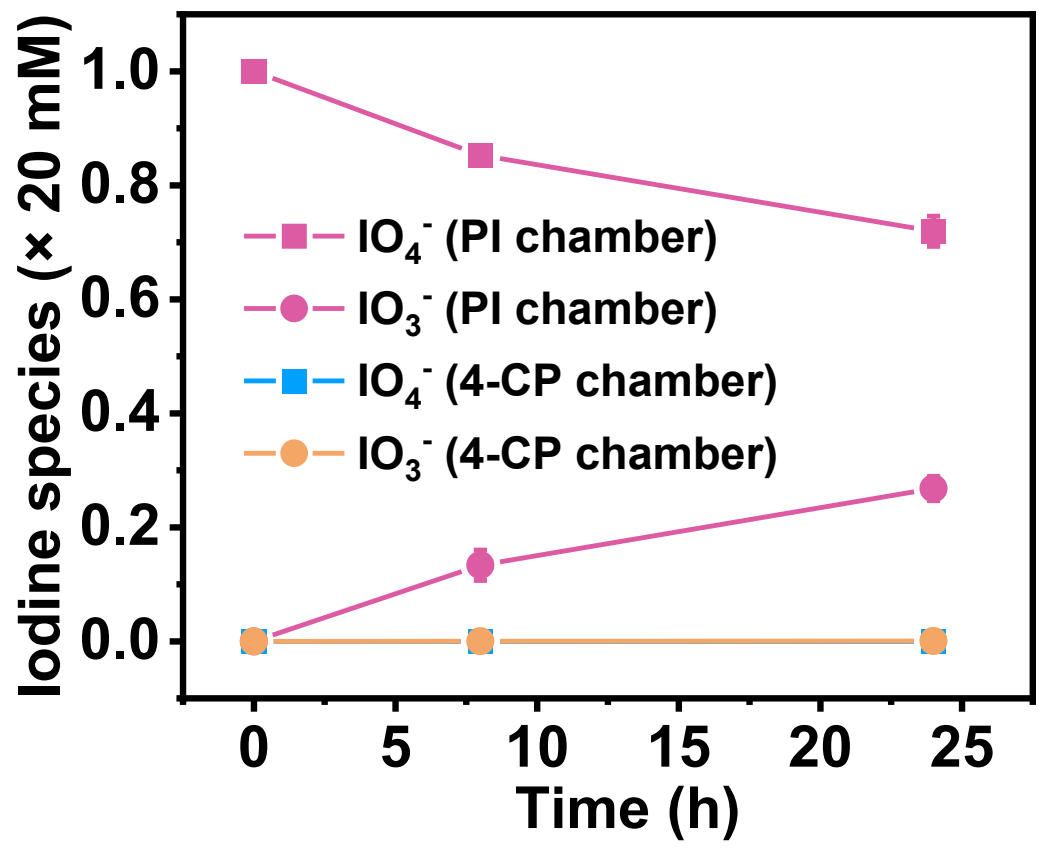

Fig. R29 Concentration changes of IO_4^- and IO_3^- in the F-FeSAC/PI/4-CP GOP system.

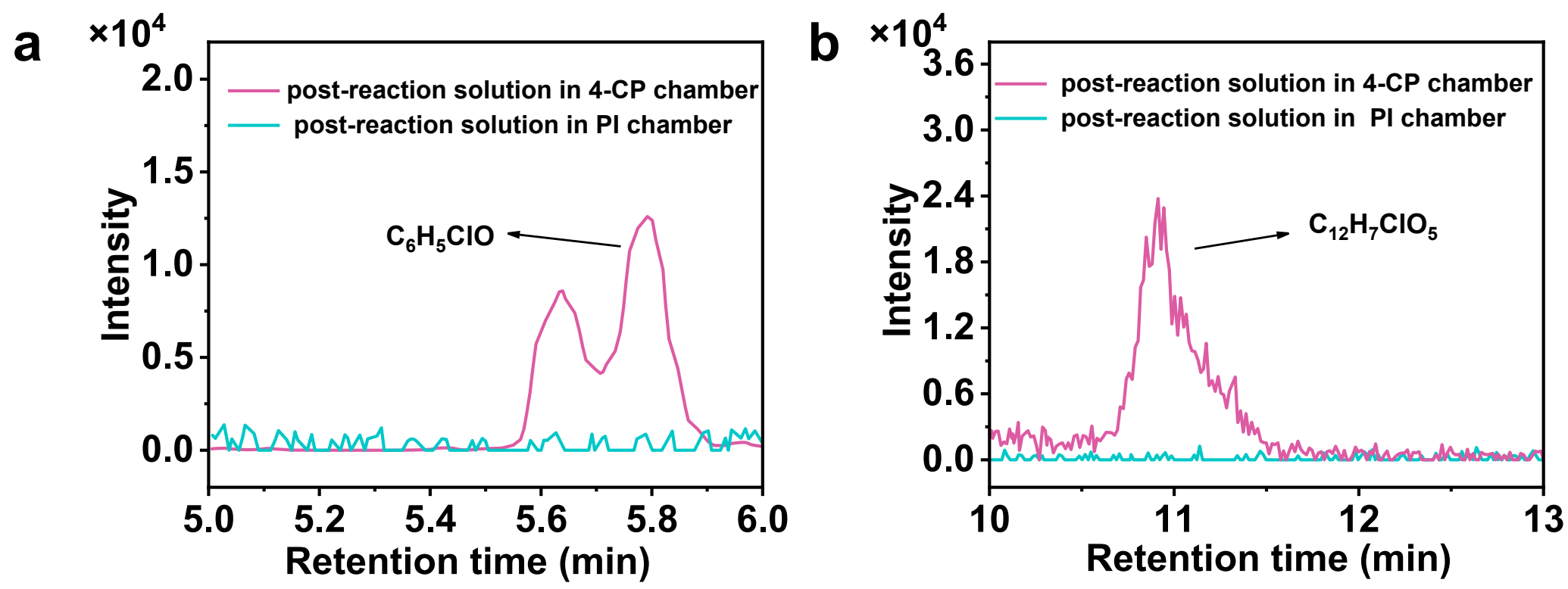

Fig. R30 Extracted ion chromatogram (EIC) comparison of 4-CP and its transformation products in the PI and 4-CP chambers.

9. What was the scientific basis for selecting 3 min and 10 min intervals for 4-CP addition in the premixing experiments?

Response 9:

We thank the reviewer for highlighting the need to clarify the basis for selecting the 3 min and 10 min premixing intervals before 4-CP addition.

These two intervals were selected based on the degradation timescale of 4-CP in the present R-FeSAC/PI system. In the standard reaction without premixing, 4-CP degradation occurs rapidly within the first several minutes and is largely completed

within 10 min. Therefore, 3 min was chosen to represent a short R-FeSAC/PI contact period within the initial rapid-reaction stage. By contrast, 10 min was chosen to as an extended premixing period that covers the main reaction window of 4-CP degradation under the same reaction conditions.

As shown in Fig. 3d and Supplementary Fig. 22, the degradation profiles obtained after 3 min and 10 min premixing were close to that of the control without premixing. This observation indicates that, within the reaction-relevant timescale selected above, extending R-FeSAC/PI pre-contact from 3 min to 10 min has little influence on the subsequent 4-CP degradation.

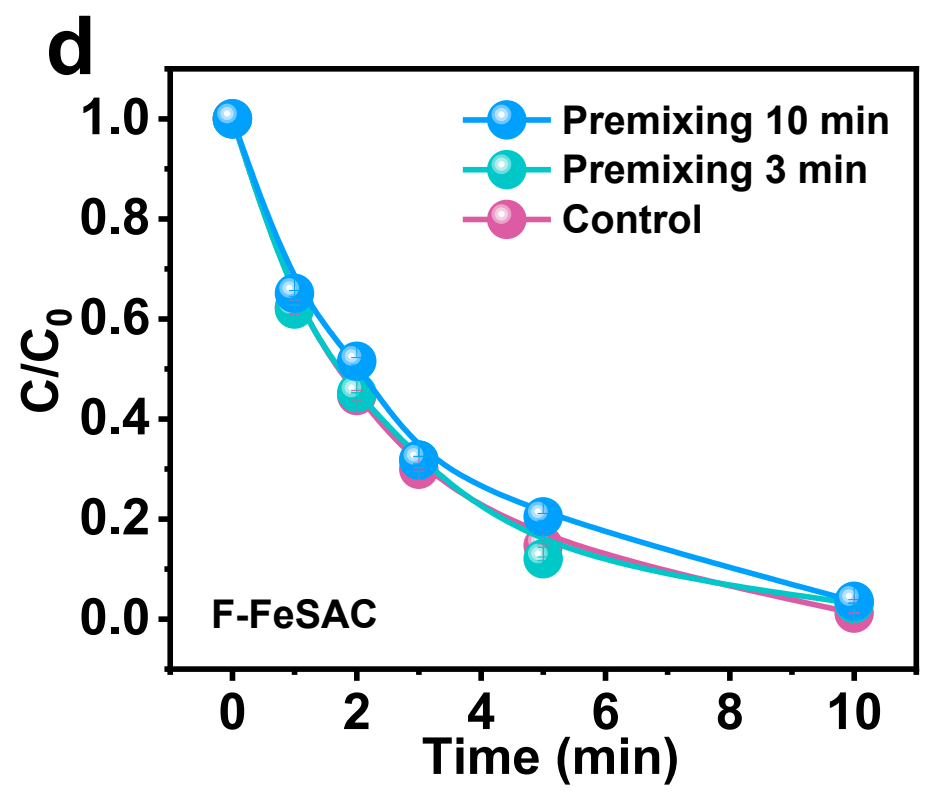

Fig. 3 (d) Effect of premixing on 4-CP removal in the F-FeSAC/PI system.

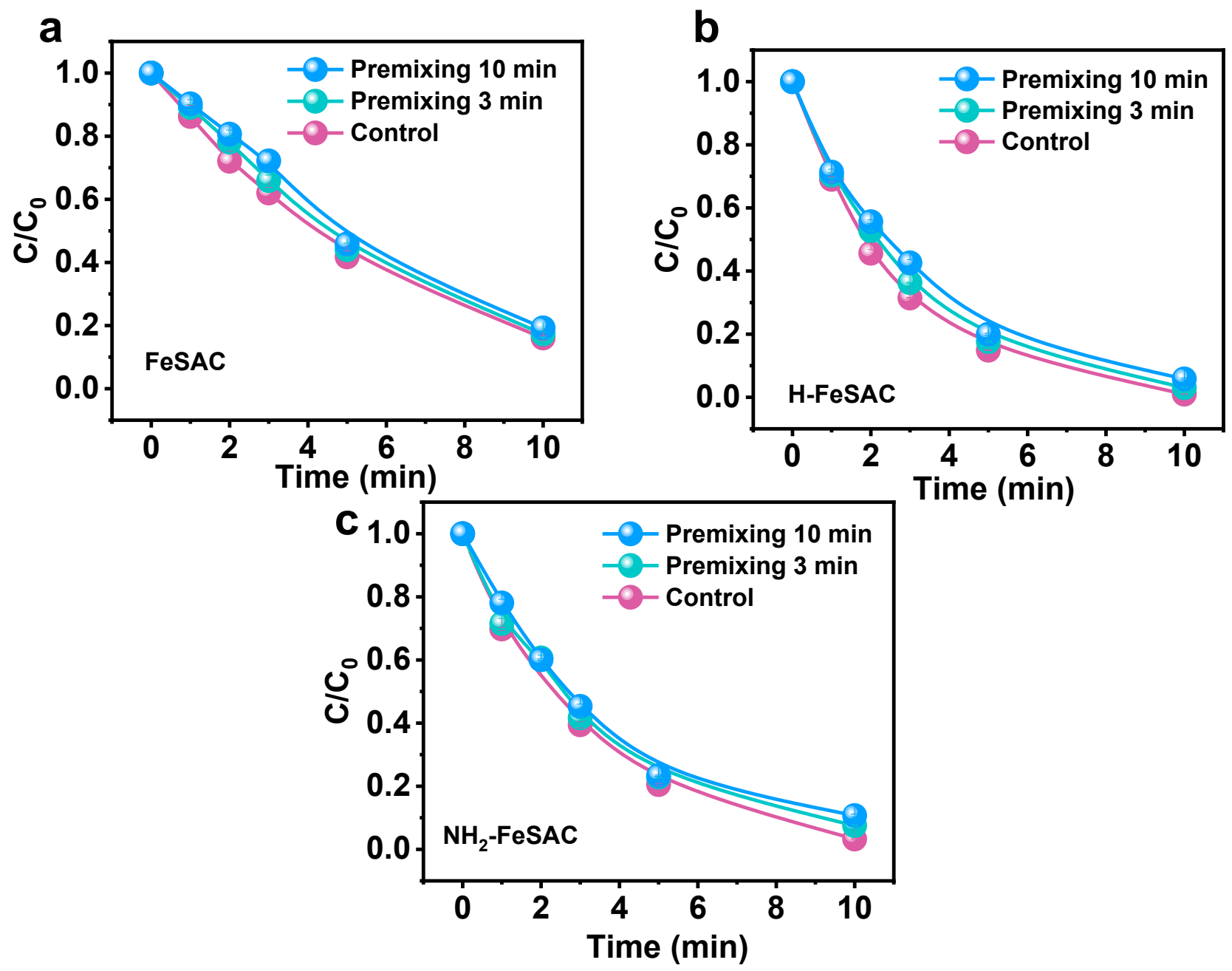

Supplementary Fig. 22 Effect of premixing in the (a) FeSAC/PI, (b) H-FeSAC/PI, (c) NH_2-FeSAC/PI system on 4-CP removal.

10. For the zebrafish toxicity assay (Fig. 6d), please report quantitative survival rates and include statistical analysis. The current presentation with representative images alone is insufficient for drawing conclusions about biocompatibility.

Response 10:

We thank the reviewer for this important comment. We agree that representative zebrafish images alone are insufficient to support a conclusion regarding biocompatibility. Therefore, we have reanalyzed the zebrafish toxicity assay and added quantitative survival-rate data together with statistical analysis.

The survival rate was calculated as the number of surviving zebrafish divided by the total number of exposed zebrafish in each replicate. The data are now presented as mean ± SD from three independent experiments. Statistical significance was evaluated using one-way ANOVA followed by Tukey's post hoc test.

The untreated 4-CP solution caused clear survival inhibition, with survival rates decreasing from 58.9 ± 8.4% at 24 h to 12.2 ± 8.4% at 120 h. In contrast, zebrafish exposed to the treated solution maintained much higher survival rates of 93.3 ± 3.3%, 87.8 ± 8.4%, 85.6 ± 5.1%, and 83.3 ± 3.3% at 24, 48, 96, and 120 h, respectively (Table R4 and Fig. R31). These values were not significantly different from those of the blank group at the corresponding time points, but were significantly higher than those of the untreated 4-CP group.

Table R4. Zebrafish survival rates after exposure to blank, 4-CP solution (Control), and post-reaction 4-CP solutions (F-FeSAC/PI).

Comparison	24 h	48 h	96 h	120 h
Blank	95.6±1.9%	92.2±1.9%	91.1±1.9%	90.0±3.3%
4-CP solution (Control)	58.9±8.4%	33.3±6.7%	15.6±5.1%	12.2±8.4%
post-reaction 4-CP solution (F-FeSAC/PI)	93.3±13.3%	87.8±8.4%	85.6±5.1%	83.3±3.3%

Data are presented as mean ± SD from three independent experiments, n=30.

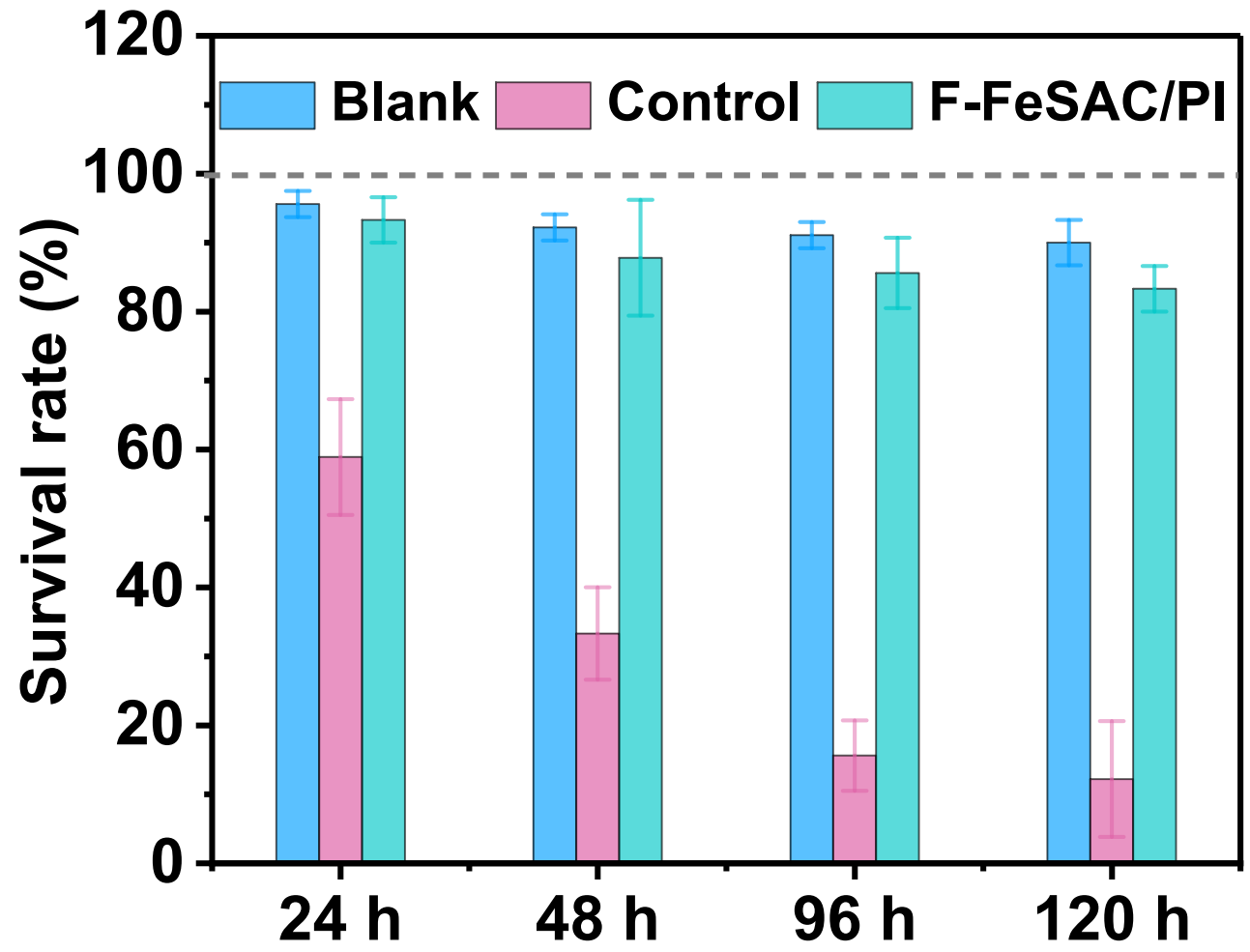

Fig. R31 Quantitative survival rates of zebrafish exposed to the control, untreated 4-CP solution, and treated solution for 24, 48, 96, and 120 h. Survival rates were calculated as the number of surviving zebrafish divided by the total number of exposed zebrafish in each replicate. Data are presented as mean ± SD from three independent experiments, n=30. Statistical significance was determined by one-way ANOVA followed by Tukey's post hoc test. ns, not significant; ***$p < 0.001$; ****$p < 0.0001$.

Table R5. Statistical comparison of zebrafish survival rates between the post-reaction 4-CP solution, blank, and untreated 4-CP solution.

Comparison	24 h	48 h	96 h	120 h
post-reaction 4-CP solution (F-FeSAC/PI) vs Blank	ns	ns	ns	ns
post-reaction 4-CP solution (F-FeSAC/PI) vs 4-CP solution (Control)	***	****	****	****

*Note: Statistical significance was determined by one-way ANOVA followed by Tukey's post hoc test. ns, not significant; ***$p < 0.001$; ****$p < 0.0001$.*

These quantitative results demonstrate that F-FeSAC/PI treatment substantially reduced the acute toxicity of the 4-CP-containing solution under the tested exposure conditions.

Action 10: Quantitative zebrafish survival-rate data have been added to Supplementary Fig. 47 and Table 6. Statistical analysis was performed using one-way ANOVA followed by Tukey's post hoc test. The figure caption was revised to include the survival-rate calculation, replicate number, data presentation format, and significance notation. The manuscript text was also revised to avoid relying solely on representative images and to support the toxicity assessment with quantitative survival data.

*"Quantitative survival analysis showed that the untreated 4-CP solution caused clear survival inhibition during 120 h of exposure, whereas zebrafish exposed to the F-FeSAC/PI-treated solution maintained survival rates comparable to those of the blank group and significantly higher than those of the untreated 4-CP group (**Supplementary Fig. 47 and Table 6**). Representative images further showed no obvious morphological abnormalities after exposure to the treated solution (Fig. 6d). These results indicate that F-FeSAC/PI treatment substantially attenuated the acute toxicity of the 4-CP-containing solution."*

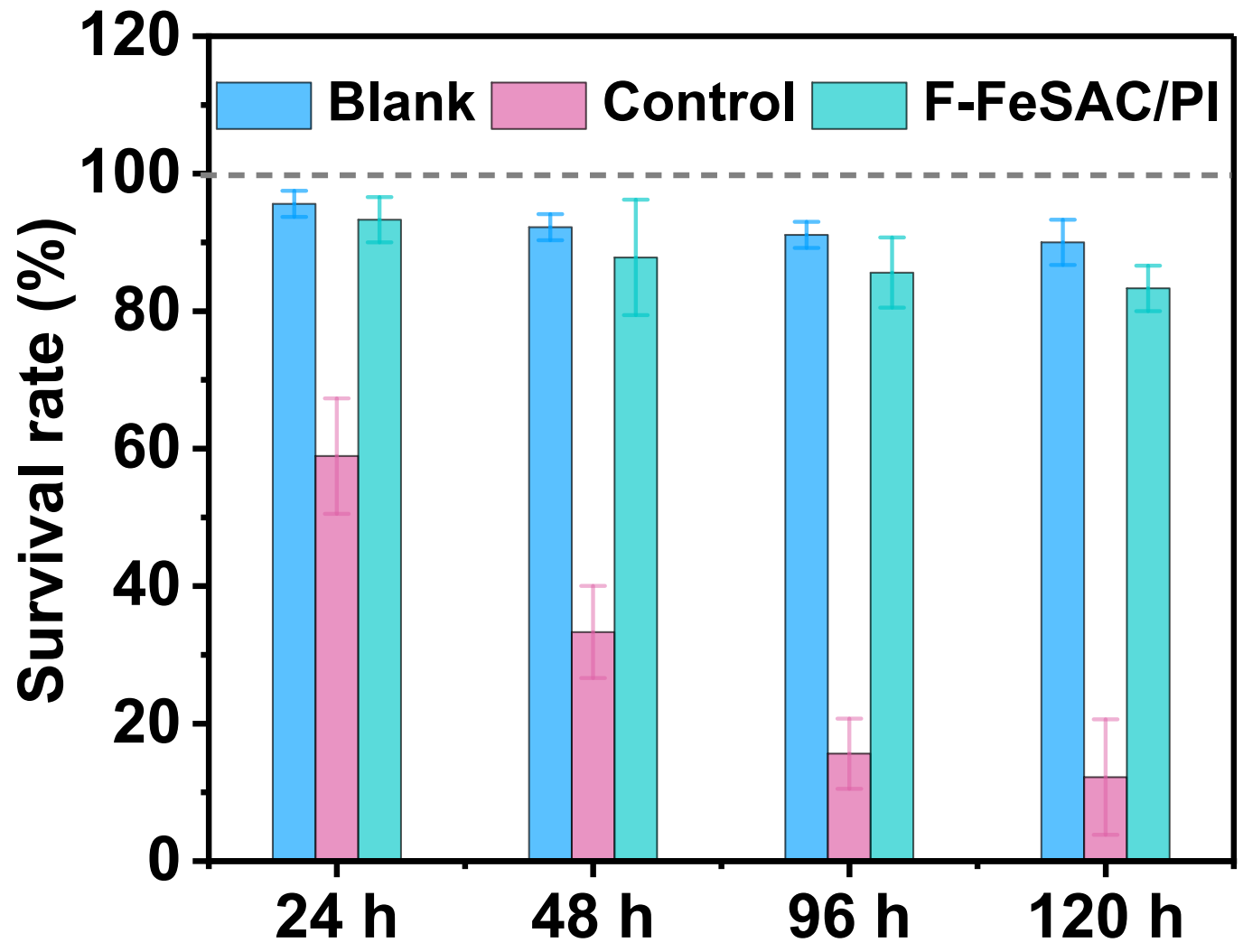

Supplementary Fig. 47 Quantitative survival rates of zebrafish exposed to the control, untreated 4-CP solution, and treated solution for 24, 48, 96, and 120 h. Survival rates were calculated as the number of surviving zebrafish divided by the total number of exposed zebrafish in each replicate. Data are presented as mean ± SD from three independent experiments, n=30. Statistical significance was determined by one-way ANOVA followed by Tukey's post hoc test. ns, not significant; ***$p < 0.001$; ****$p < 0.0001$.

Supplementary Table 6. Statistical comparison of zebrafish survival rates between the post-reaction 4-CP solution, blank, and untreated 4-CP solution

Comparison	24 h	48 h	96 h	120 h
post-reaction 4-CP solution (F-FeSAC/PI) vs Blank	ns	ns	ns	ns
post-reaction 4-CP solution(F-FeSAC/PI) vs 4-CP solution (Control)	***	****	****	****

*Note: Data are presented as mean ± SD from three independent experiments, n=30. Statistical significance was determined by one-way ANOVA followed by Tukey's post hoc test. ns, not significant; ***p < 0.001; ****p < 0.0001.*

11. The correlation between ln(kobs) and φ1/2 (Fig. 3k) excludes BA and NB based on a φ1/2 < 1.2 V threshold (line 387), yet the two pollutants are included in the linear regressions in Fig. 4b,c. This inconsistency raises questions whether this categorization has a mechanistic basis or is introduced as a post-hoc threshold to optimize linearity.

Response 11:

We sincerely thank the reviewer for raising this important point. We agree that the original manuscript did not explain clearly enough why BA and NB were handled differently in Fig. 3k and Fig. 4b,c.

BA and NB were treated differently because the two analyses were designed to answer different mechanistic questions. In Fig. 3k, $\varphi_{1/2}$ was used to examine whether the degradation rates of responsive pollutants follow their intrinsic redox properties. This analysis is meaningful only when the pollutants undergo measurable and comparable oxidation in the F-FeSAC/PI system. BA and NB showed much lower reactivity under the same conditions and therefore were not used to define this local $\varphi_{1/2}$-dependent kinetic trend. This treatment clarifies the applicable range of the $\varphi_{1/2}$ analysis and was not based on selective removal of data points to improve the regression.

Fig. 4b,c examine the same reactivity series from a broader electronic-structure perspective. Here, $LUMO_{(R\text{-}FeSAC/PI)} - HOMO_{pollutant}$ was used to quantify the frontier-orbital energy offset between the activated R-FeSAC/PI complex and each pollutant molecule. Compared with $\varphi_{1/2}$, which describes the redox property of the isolated pollutant, this orbital-energy descriptor more directly reflects the energetic accessibility of pollutant-to-complex electron transfer. BA and NB were therefore retained in Fig. 4b,c as weakly reactive boundary cases within the complete pollutant set. Their

inclusion tests whether the frontier-orbital analysis can account for both readily degraded and poorly reactive pollutants.

In this context, Fig. 3k and Fig. 4b,c should be interpreted as complementary analyses rather than as applications of the same selection rule. Fig. 3k focuses on a local relationship between $\varphi_{1/2}$ and degradation kinetics for responsive pollutants, whereas Fig. 4b,c evaluate whether the complete reactivity trend can be described by the frontier-orbital energy offset between pollutants and the activated R-FeSAC/PI complex. BA and NB were therefore excluded from the former analysis but retained in the latter as weakly reactive boundary cases. Importantly, the correlations in Fig. 4b,c remained strong after BA and NB were included, with R^2 values of 0.893 and 0.907, respectively. This result indicates that the orbital-energy analysis remains valid for the complete pollutant set and was not dependent on removing poorly reactive substrates.

Action 11: To avoid further ambiguity, we have revised the manuscript text to clarify that the $\varphi_{1/2}$ threshold was not used as a post-hoc exclusion criterion. Instead, $\varphi_{1/2}$ and $LUMO_{(R\text{-}FeSAC/PI)}-HOMO_{pollutant}$ are now explicitly described as two related but distinct electronic descriptors: the former reflects the pollutant redox property within a kinetically responsive subset, whereas the latter captures the broader orbital-energy matching between pollutants and the activated R-FeSAC/PI complex. The revised version is provided below.

"Conversely, BA and NB showed elevated $\varphi_{1/2}$ values, pronounced departure from the linear trend, and markedly lower $\ln(k_{obs})$ values. This behavior is consistent with the presence of electron-withdrawing –COOH and –NO_2 groups, which decrease the electron density of the aromatic ring and render oxidation less favorable (Supplementary Fig. 29). These results indicate that pollutant oxidation potential is closely associated with degradation kinetics in the F-FeSAC/PI system, particularly for contaminants that readily undergo electron-transfer-mediated oxidation."

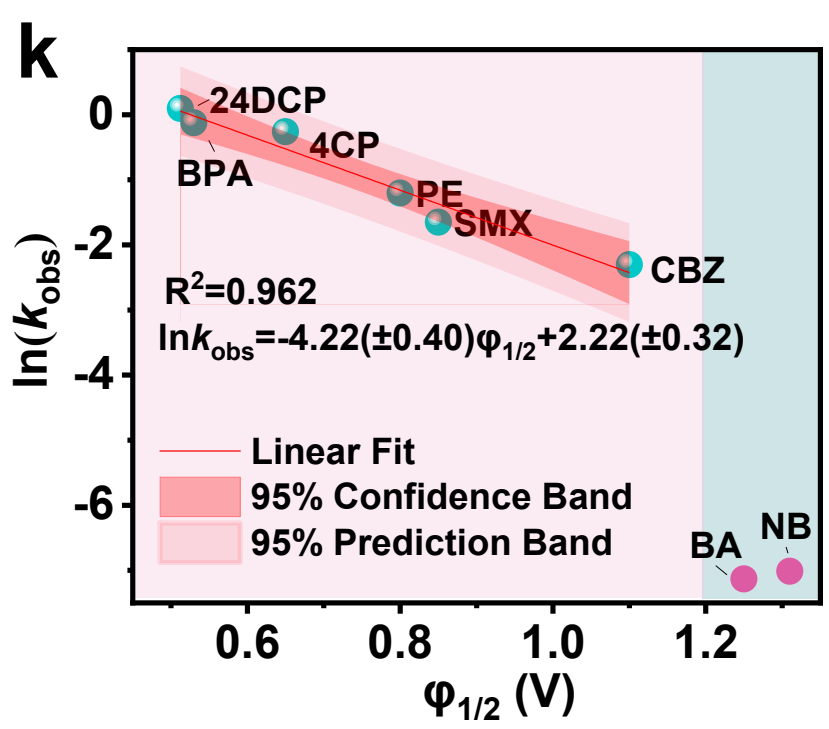

Fig. 3 (k) Correlation between the $\varphi_{1/2}$ values of eight contaminants and their $\ln(k_{obs})$.

"The narrower energy gap inherent to the F-FeSAC/PI system implies a more favorable electron transfer pathway. Experimental validation corroborates this theoretical prediction, revealing a linear relationship between the magnitude of this energy gap and the observed lnk_{obs} (Fig. 4c). Complementing the $\varphi_{1/2}$-based trend in Fig. 3k, the frontier-orbital descriptor captures the reactivity differences across the full pollutant series, including BA and NB. This broader correlation further links pollutant degradation kinetics to the frontier-orbital energy alignment between F-FeSAC/PI and the pollutant."

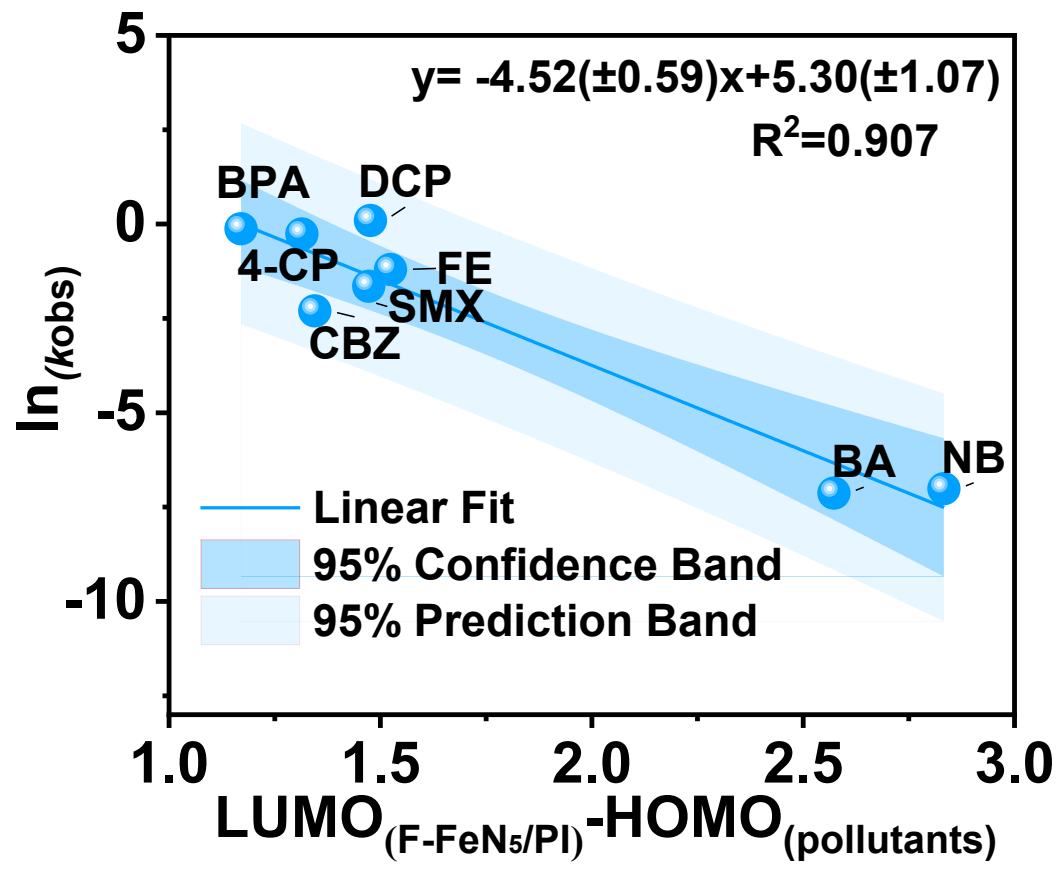

Fig. 4 (c) Linear correlation between LUMO$_{(R\text{-}FeSAC/PI)}$-HOMO$_{pollutants}$ and ln(k_{obs}).

12. In the discussion of the pilot-scale ETP reactor (lines 571-583), the authors refer to "the PMS electron acceptor", whereas the rest of the section and Fig. 5m describe a PI-based system. The authors should clarify whether PMS was indeed used in the pilot-scale experiment.

Response 12:

We thank the reviewer for carefully identifying this inconsistency. We apologize for the inaccurate wording in this section. The pilot-scale ETP experiment was conducted using PI as the electron acceptor, not PMS. We have corrected this phrase to "PI electron acceptor" in the revised manuscript.

Action 12: The incorrect phrase "PMS electron acceptor" has been revised to "PI electron acceptor" in the discussion of the pilot-scale ETP reactor.

*"Concurrently, the PI electron acceptor was consumed in the dedicated chambers, with only ~40% remaining (**Fig. 5m**)."*

m

Fig. 5 (m) 4-CP removal efficiency and PI consumption in the amplifying ETP reactor.

13. Minor comments:

- Typo, 0.8 in Figure 3g should be corrected to 0.08.
- The bar graph in Figure 3c is currently difficult to interpret and compare.
- In Fig. 3k, the R2 value (0.962) differs from the R2 = 0.90 stated in the main text (line 388). The values should be corrected.
- Including specific values such as g-values for EPR and directly stating kobs values would significantly improve the clarity and impact for the readership.

Response 13:

We thank the reviewer for these helpful comments. We have carefully checked the figures and the corresponding text.

Action 13: Fig. 3g, Fig. 3c, Fig. 3k, and the corresponding text in the revised manuscript have been corrected and refined accordingly.

1) First, the value "0.8" in **Fig. 3g** was inaccurate and has been corrected to "0.08".

g

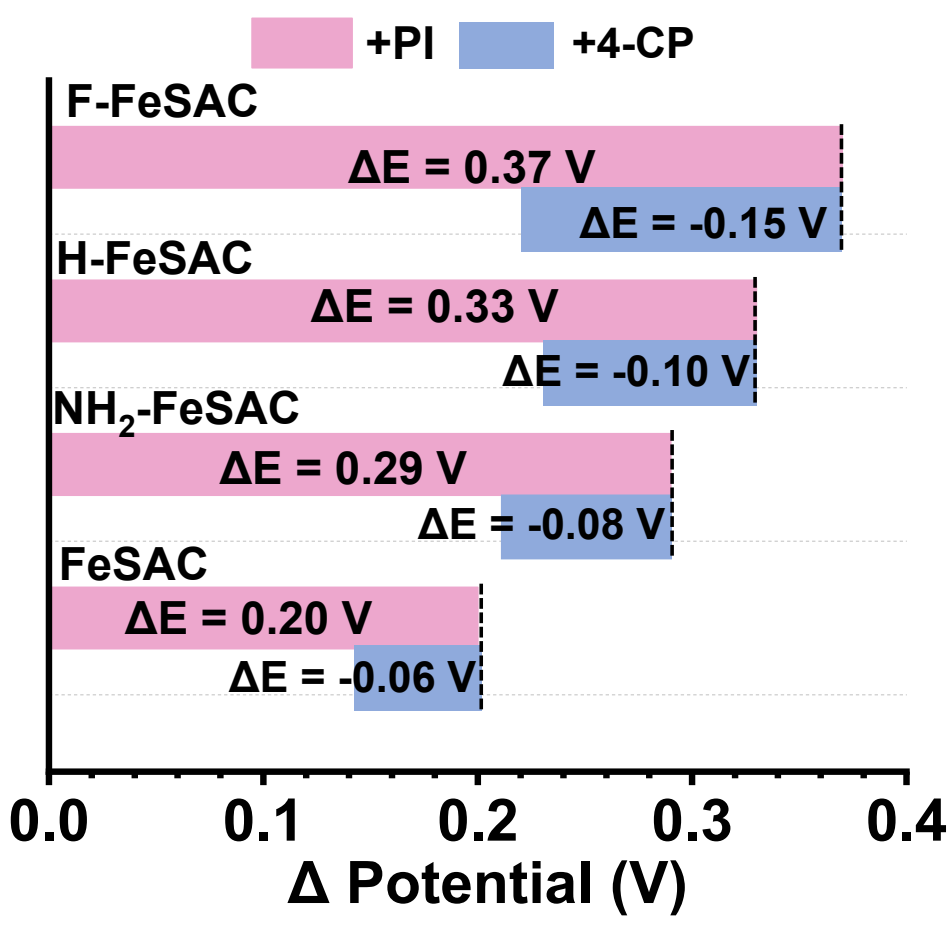

Fig. 3 (g) OCP changes of different systems with the addition of PI and 4-CP.

2) Second, we have revised the bar graph in **Fig. 3c** to improve readability and facilitate direct comparison among different reaction conditions.

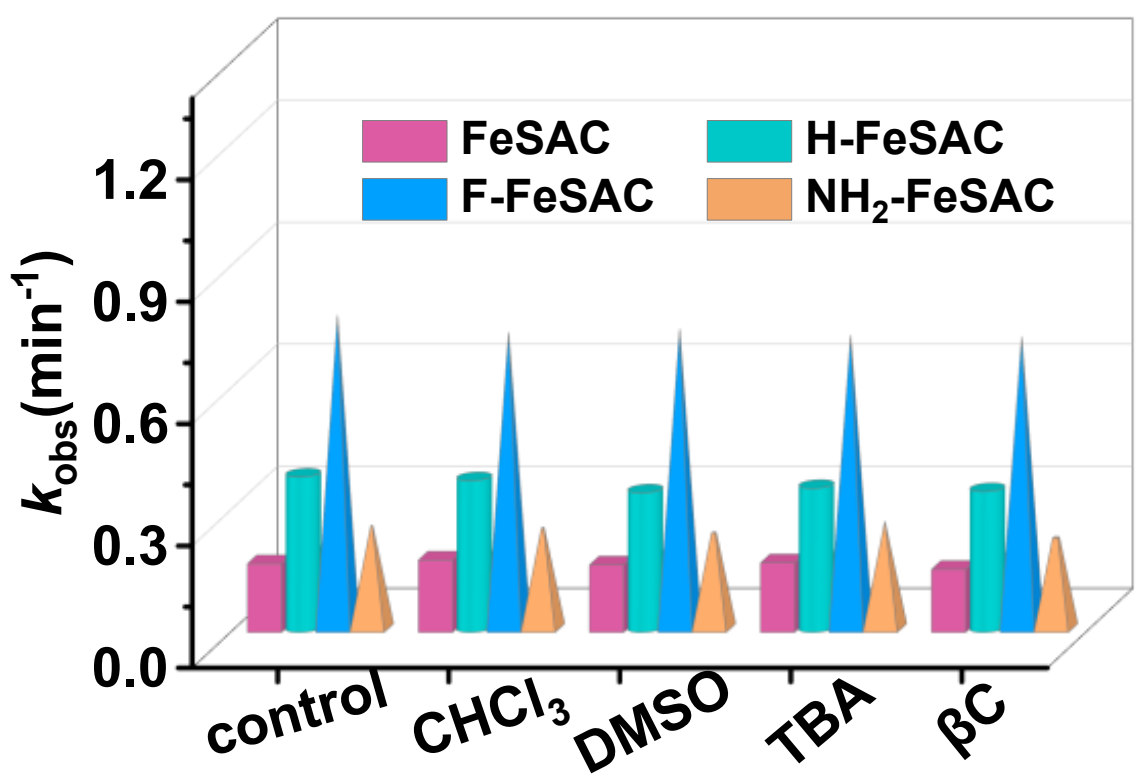

Fig. 3 (c) The k_{obs} values of FeSAC/PI and R-FeSAC/PI systems in the presence of various quenchers.

3) Third, we corrected the inconsistent R^2 value in the main text. The R^2 value is now consistently reported as 0.962, in agreement with **Fig. 3k**.

*"In the F-FeSAC system, contaminants with $\varphi_{1/2}$ < 1.2 V (2,4-DCP, PE, CBZ, BPA, 4-CP, SMX) demonstrated a strong linear correlation between lnk_{obs} and $\varphi_{1/2}$ (R^2 = 0.962, **Fig. 3k**)."*

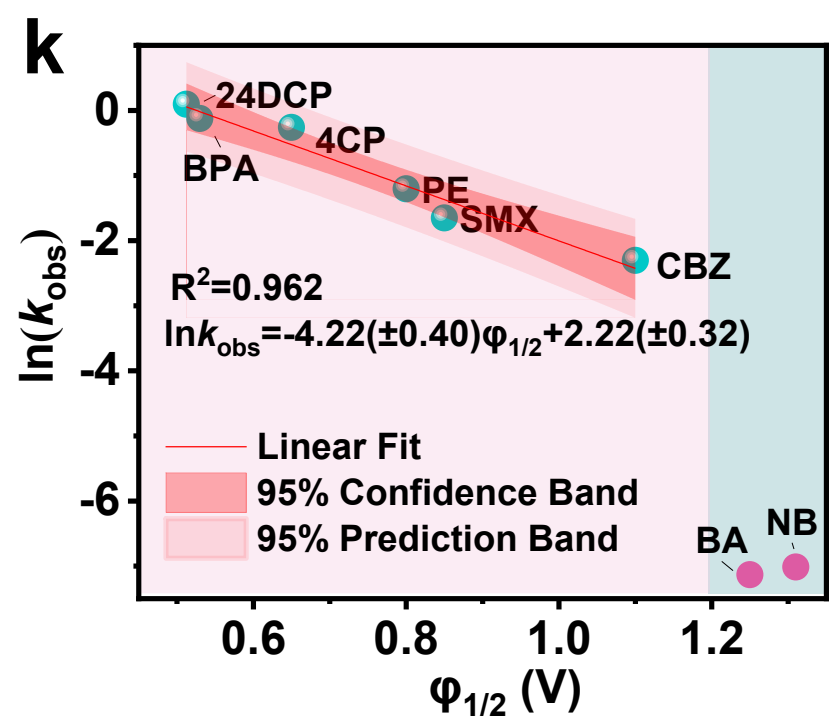

Fig. 3 (k) Correlation between the $\varphi_{1/2}$ values of eight contaminants and their ln(k_{obs}).

4) Finally, we have added the relevant g-values for the EPR signals and directly stated the k_{obs} values in the revised manuscript to improve clarity for readers.

"As shown in Supplementary Fig. 14 and 15, no detectable DMPO-$O_2^{\bullet-}$ (α_N = 14.3 G, α_H^{β} = 11.4 G, α_H^{γ} = 1.2 G) or DMPO-$IO_3^{\bullet}$ (α_H = 12.9 G, α_N = 14.2 G) signals were observed, excluding $O_2^{\bullet-}$ and $IO_3^{\bullet}$ as the dominant radical species in these systems. Although weak DMPO-•OH signals (α_N = α_H = 14.9 G) were detected, their spectral profiles and intensities remained essentially unchanged after 4-CP addition, suggesting that •OH was not directly involved in 4-CP oxidation (Supplementary Fig. 16)."

"This conclusion was corroborated by EPR spectral analysis (Supplementary Figs. 16a-d), which revealed no detectable variation in TEMP-$^1O_2$ signals (α_N = 16 G) in the

catalyst/PI systems, regardless of the presence or absence of 4-CP."

"As illustrated in Fig. 3c and Supplementary Figs. 11a-d, tert-butanol (TBA) and chloroform ($CHCl_3$) were used to probe the possible contributions of •OH and $O_2^{\bullet-}$, respectively. Their addition caused only minor changes in reaction kinetics, with representative k_{obs} values for F-FeSAC/PI remaining comparable to that of the control system (0.72 min^{-1} for TBA, 0.73 min^{-1} for $CHCl_3$, and 0.77 min^{-1} for the control)."

"βC caused negligible kinetic inhibition, with the representative k_{obs} of F-FeSAC/PI changing from 0.77 to 0.72 min^{-1} (Fig. 3c and Supplementary Figs. 11a-d), while D_2O substitution did not accelerate the reaction kinetics (0.77 versus 0.76 min^{-1}; Supplementary Fig. 18)."

References

1. Du J, Xiao G, Xi Y, Zhu X, Su F, Kim SH. Periodate activation with manganese oxides for sulfanilamide degradation. *Water Res.* **169**, 115278 (2020).
2. Tang Q*, et al.* Electron transfer mediated activation of periodate by contaminants to generate $^1O_2$ by charge-confined single-atom catalyst. *Nat. Commun.* **15**, 9549 (2024).
3. Chen L*, et al.* Solar-light-activated periodate for degradation and detoxification of highly toxic 6PPD-quinone at environmental levels. *Nat. Water* **2**, 453-463 (2024).
4. Zong Y*, et al.* Enhanced oxidation of organic contaminants by Iron(II)-activated periodate: The significance of high-valent iron-oxo species. *Environ. Sci. Technol.* **55**, 7634-7642 (2021).
5. Luo M*, et al.* Electrochemical activation of periodate with graphite electrodes for water decontamination: Excellent applicability and selective oxidation mechanism. *Water Res.* **240**, 120128 (2023).
6. Yu Y, Dong H, Chen T, Sun Y, Guan X. Unraveling the intrinsic mechanism behind the selective oxidation of sulfonamide antibiotics in the Mn(II)/periodate process: The overlooked surface-mediated electron transfer process. *Water Res.* **244**, 120507 (2023).
7. Peng J*, et al.* Removal of phenols by highly active periodate on carbon nanotubes: A mechanistic investigation. *Environ. Sci. Technol.* **57**, 10804-10815 (2023).
8. Hamdaoui O, Merouani S. Improvement of sonochemical degradation of Brilliant blue R in water using periodate ions: Implication of iodine radicals in the oxidation process. *Ultrason. Sonochem* **37**, 344-350 (2017).
9. Yang T*, et al.* Enhanced hydroxyl radical generation for micropollutant degradation in the In_2O_3/Vis-LED process through the addition of periodate. *Water Res.* **243**, 120401 (2023).
10. Bokare AD, Choi W. Singlet-oxygen generation in alkaline periodate solution. *Environ. Sci. Technol.* **49**, 14392-14400 (2015).
11. Niu L, Lin J, Chen W, Zhang Q, Yu X, Feng M. Ferrate(VI)/periodate system: Synergistic and rapid oxidation of micropollutants via periodate/iodate-modulated Fe(IV)/Fe(V) intermediates. *Environ. Sci. Technol.* **57**, 7051-7062 (2023).
12. Choi Y*, et al.* Activation of periodate by freezing for the degradation of aqueous organic pollutants. *Environ. Sci. Technol.* **52**, 5378-5385 (2018).
13. Lee H*, et al.* Oxidizing capacity of periodate activated with iron-based bimetallic nanoparticles. *Environ. Sci. Technol.* **48**, 8086-8093 (2014).

14. Yao Y, Zhang L, Qiu Y, Li Z, Ma Z, Wang S. Phase-activity relationship of MnO_2 nanomaterials in periodate oxidation for organic pollutant degradation. *Water Res.* **264**, 122224 (2024).
15. Ling C, *et al.* Sulfide-modified zero-valent iron activated periodate for sulfadiazine removal: Performance and dominant routine of reactive species production. *Water Res.* **220**, 118676 (2022).
16. Chen Y, Ge X, Li J, Zhang ZB, Li Z. Anchored atomic Ru-O_4 architecture enables ultra-effective Fe(VI) activation via avoiding Fe(VI) self-decay for water purification. *Nat. Commun.* **16**, 7509 (2025).
17. Wu Z, Xiong Z, Huang B, Yao G, Zhan S, Lai B. Long-range interactions driving neighboring Fe–N_4 sites in Fenton-like reactions for sustainable water decontamination. *Nat. Commun.* **15**, 7775 (2024).
18. Lin Y, *et al.* Coordination engineering of heterogeneous high-valent Fe(IV)-oxo for safe removal of pollutants via powerful Fenton-like reactions. *Nat. Commun.* **15**, 10032 (2024).
19. Liu T, *et al.* Water decontamination via nonradical process by nanoconfined Fenton-like catalysts. *Nat. Commun.* **14**, 2881 (2023).
20. Shi L J, *et al.* Dual-substrate synergistic catalysis for highly efficient water purification. *Nat. Water* **3**, 345-353 (2025).
21. Guo J, *et al.* Fenton-like activity and pathway modulation via single-atom sites and pollutants comediates the electron transfer process. *Pro. Natl. Acad. Sci.* **121**, e2313387121 (2024).
22. Shang Y, Xu X, Gao B, Wang S, Duan X. Single-atom catalysis in advanced oxidation processes for environmental remediation. *Chem. Soc. Rev.* **50**, 5281-5322 (2021).
23. Zhu Z-S, *et al.* Multidimensional engineering of single-atom cobalt catalysts for ultrafast Fenton-like reactions. *Nat. Water* **3**, 211-221 (2025).
24. Yang H, *et al.* Manganese vacancy-confined single-atom Ag in cryptomelane nanorods for efficient Wacker oxidation of styrene derivatives. *Chem. Sci.* **12**, 6099-6106 (2021).
25. Song W, *et al.* Review of carbon support coordination environments for single metal atom electrocatalysts (SACS). *Adv. Mater.* **36**, 2301477 (2024).
26. Sun T, *et al.* Design of local atomic environments in single-atom electrocatalysts for renewable energy conversions. *Adv. Mater.* **33**, 2003075 (2021).
27. Stripp ST, *et al.* Second and outer coordination sphere effects in nitrogenase,

hydrogenase, formate dehydrogenase, and CO dehydrogenase. *Chem. Rev.* **122**, 11900-11973 (2022).

28. Kong S*, et al.* Delocalization state-induced selective bond breaking for efficient methanol electrosynthesis from CO_2. *Nat. Catal.* **6**, 6-15 (2023).
29. Cui X, Gao L, Lu C-H, Ma R, Yang Y, Lin Z. Rational coordination regulation in carbon-based single-metal-atom catalysts for electrocatalytic oxygen reduction reaction. *Nano Conver.* **9**, 34 (2022).
30. Li Q*, et al.* Modulation of the second-beyond coordination structure in single-atom electrocatalysts for confirmed promotion of ammonia synthesis. *J. Am. Chem. Soc.* **147**, 1884-1892 (2025).
31. Zhu P*, et al.* Tailoring the selectivity and activity of oxygen reduction by regulating the coordination environments of carbon-supported atomically dispersed metal sites. *J. Mater. Chem. A* **10**, 17948-17967 (2022).
32. Jin S*, et al.* Remote tuning of single-atom $Fe-N_5$ sites via high-coordination defects for enhanced Fenton-like water decontamination. *Nat. Commun.* **16**, 10455 (2025).
33. Dou J, Tang Y, Lu Z, He G, Xu J, He Y. Neglected but efficient electron utilization driven by biochar-coactivated phenols and peroxydisulfate: Polyphenol accumulation rather than mineralization. *Environ. Sci. Technol.* **57**, 5703-5713 (2023).
34. Zhou T*, et al.* Reaction pathway tuning between electron transfer-mediated degradation and polymerization in $Fe-MoS_2$-based persulfate activation system. *Environ. Sci. Technol.* **60**, 4406-4417 (2026).
35. Xie W*, et al.* Selective polymerization of chlorophenols in hypersaline wastewater via surface-complex-mediated electron transfer over Ce-doped CuO. *Water Res.* **284**, 124023 (2025).
36. Liu H-Z*, et al.* Tailoring d-band center of high-valent metal-oxo species for pollutant removal via complete polymerization. *Nat. Commun.* **15**, 2327 (2024).
37. Zuo X, Xin C, Postigo C. High-resolution mass spectrometry to advance DBP research. *Curr. Opin. Environ. Sci. Health* **48**, 100682 (2025).
38. Yang Y, Peng Ye, Chang Q, Dan C, Guo W, Wang Y. Selective identification of organic iodine compounds using liquid chromatography-high resolution mass spectrometry. *Anal. Chem.* **88**, 1275-1280 (2016).
39. Zonja B, Delgado A, Pérez S, Barceló D. LC-HRMS suspect screening for detection-based prioritization of iodinated contrast media photodegradates in surface waters. *Environ. Sci. Technol.* **49**, 3464-3472 (2015).

40. Wu B*, et al.* Axially engineered single atoms in enzyme-mimic-binding pocket steering dehalogenation-polymerization pathways toward water pollutant upcycling. *Nat. Commun.* **17**, 2405 (2026).
41. Kumar A*, et al.* Advances in coordination engineering of M-N-C single atom catalysts for superior oxygen reduction performance. *Coord. Chem. Rev.* **549**, 217244 (2026).
42. Van Stappen C*, et al.* Designing artificial metalloenzymes by tuning of the environment beyond the primary coordination sphere. *Chem. Rev.* **122**, 11974-12045 (2022).
43. Zhong H*, et al.* Selective introduction of pentagon defects into Co-N_4 sites for boosting Fenton-like activity. *Adv. Funct. Mater.* **35**, 2501208 (2025).
44. Yang P*, et al.* Regulating the local electronic structure of copper single atoms with unsaturated B,O-coordination for selective $^1O_2$ generation. *ACS Catal.* **13**, 12414-12424 (2023).
45. Zhang L*, et al.* Constructing hollow multishelled microreactors with a nanoconfined microenvironment for ofloxacin degradation through peroxymonosulfate activation: Evolution of high-valence cobalt-oxo species. *Environ. Sci. Technol.* **57**, 16141-16151 (2023).
46. Zhu C*, et al.* Heterogeneous Fe-Co dual-atom catalyst outdistances the homogeneous counterpart for peroxymonosulfate-assisted water decontamination: New surface collision oxidation path and diatomic synergy. *Water Res.* **241**, 120164 (2023).
47. Shi Z*, et al.* Fe confined in Prophyrin-based porous organic polymer as an efficient periodate activator for nonradical pathway removal of contaminants. *Sep. Purif. Technol.* **317**, 123868 (2023).
48. Cheng C, Ren W, Miao F, Chen X, Chen X, Zhang H. Generation of Fe^{IV}=O and its contribution to Fenton-like reactions on a single-atom iron−N−C catalyst. *Angew. Chem.* **135**, e202218510 (2023).
49. Zhang L-S*, et al.* Carbon Nitride Supported High-Loading Fe single-atom catalyst for activation of peroxymonosulfate to generate $^1O_2$ with 100 % selectivity. *Angew. Chem. Int. Ed.* **60**, 21751-21755 (2021).
50. Xie M*, et al.* Single-atom Co-N_5 catalytic sites on carbon nanotubes as peroxymonosulfate activator for sulfamerazine degradation via enhanced electron transfer pathway. *Sep. Purif. Technol.* **304**, 122398 (2023).
51. Wang Z*, et al.* Cobalt single atoms anchored on oxygen-doped tubular carbon

nitride for efficient peroxymonosulfate activation: Simultaneous coordination structure and morphology modulation. *Angew. Chem.* **134**, e202202338 (2022).
52. Liu C, Li J, He X, Yue J, Chen M, Chen JP. The "4 + 1" strategy fabrication of iron single-atom catalysts with selective high-valent iron-oxo species generation. *Pro. Natl. Acad. Sci.* **121**, e2322283121 (2024).
53. Wu Q-Y, Yang Z-W, Wang Z-W, Wang W-L. Oxygen doping of cobalt-single-atom coordination enhances peroxymonosulfate activation and high-valent cobalt-oxo species formation. *Pro. Natl. Acad. Sci.* **120**, e2219923120 (2023).
54. Wu Z*, et al.* Facilely Tuning the First-Shell Coordination Microenvironment in Iron Single-Atom for Fenton-like chemistry toward highly efficient wastewater purification. *Environ. Sci. Technol.* **57**, 14046-14057 (2023).
55. Yin K*, et al.* High-loading of well dispersed single-atom catalysts derived from Fe-rich marine algae for boosting Fenton-like reaction: Role identification of iron center and catalytic mechanisms. *Appl. Catal. B* **336**, 122951 (2023).
56. Cui L, Gong Y, Zhao S, Wu Y, Wang A, Chen Z. Homogenous oxidizing oligomerization coupled with coagulation for water purification. *Water Res.* **257**, 121684 (2024).
57. Long Y, Dai J, Zhao S, Su Y, Wang Z, Zhang Z. Atomically dispersed cobalt sites on graphene as efficient periodate activators for selective organic pollutant degradation. *Environ. Sci. Technol.* **55**, 5357-5370 (2021).
58. Zhang P, Yang Y, Duan X, Wang S. Oxidative polymerization versus degradation of organic pollutants in heterogeneous catalytic persulfate chemistry. *Water Res.* **255**, 121485 (2024).
59. Liu B*, et al.* Defect-induced electric field effects direct Fenton-like oxidation pathways towards polymerization for sustainable water treatment. *Nat. Commun.* **16**, 10963 (2025).
60. Chen L, Duan J, Du P, Sun W, Lai B, Liu W. Accurate identification of radicals by in-situ electron paramagnetic resonance in ultraviolet-based homogenous advanced oxidation processes. *Water Res.* **221**, 118747 (2022).

Responses to Reviewer's Comments

The revised manuscript entitled "**Enzyme-Mimicking Peripheral Substituents beyond the Second Coordination Sphere Tune Single-Atom Catalysts for Periodate Activation**" (Manuscript ID: NCOMMS-26-004517B)

Reviewer #1 (Remarks to the Author):

The authors have revised the manuscript according to my comments, and the paper can be accepted for publication

Response: Thank you for your positive feedback. We appreciate your approval and are glad the revisions meet the publication requirements.

Reviewer #2 (Remarks to the Author):

After revision, the authors have addressed all my concerns, I agree to accept this manuscript under current version.

Response: We are grateful for the reviewer's positive assessment and acceptance recommendation.

Reviewer #3 (Remarks to the Author):

Although the authors addressed some of my previous concerns, I have further questions that should be addressed.

1. The differential treatment of BA and NB across the two analyses is acknowledged. However, the 1.2 V threshold underlying Fig. 3k is not independently substantiated and appears to have been defined a posteriori by the data points it segregates. Whereas Fig. 4a-c incorporate BA and NB into the $\varphi_{1/2}$ and frontier-orbital correlations, Fig. 3k excludes these same points. The adoption of inconsistent inclusion criteria across a single contaminant set raises the concern that the classification was introduced to maximize the coefficient of determination rather than on a defensible mechanistic basis. The authors are requested to substantiate the 1.2 V threshold on physicochemical grounds.

Response 1:

We thank the reviewer for this careful and important criticism, which prompted us to re-examine the relevant analysis and improve the clarity and rigor of the manuscript.

Clarification of the 1.2 V boundary

Prompted by the reviewer's comment, we carefully re-examined the relevant discussion and recognize that using 1.2 V as a rigid dividing boundary is inappropriate because the transition between electron-transfer-accessible and electron-transfer-unfavorable pollutants is system-dependent rather than universally fixed. It depends on the catalyst/oxidant system, electrochemical measurement protocol, and pollutant set[1, 2, 3]. Therefore, we have revised the manuscript to avoid defining 1.2 V as a strict physicochemical threshold. These revisions do not alter the main conclusion that electron-rich pollutants are more favorable for ETP-mediated oxidation, whereas electron-deficient pollutants exhibit limited electron-transfer accessibility.

Reason for removing the $\varphi_{1/2}$-ln(k_{obs}) linear interpretation

We have also removed the strict $\varphi_{1/2}$-ln(k_{obs}) linear-fit interpretation. This revision is based on the mechanistic meaning and inherent limitation of $\varphi_{1/2}$ as a single electrochemical descriptor. The $\varphi_{1/2}$ mainly reflects the relative thermodynamic accessibility of the initial pollutant oxidation step. However, according to electron-transfer theory, the rate of electron transfer is not determined solely by the thermodynamic driving force associated with donor/acceptor redox potentials, but also by reorganization energy and electronic coupling between the donor and acceptor[4, 5]. Although the heterogeneous F-FeSAC/PI system is more complex than an idealized molecular electron-transfer reaction, this principle provides a clear physicochemical rationale for why $\varphi_{1/2}$ alone should not be expected to linearly predict ln(k_{obs}) across structurally diverse pollutants.

In our interfacial ETP system, the apparent degradation rate further depends on several coupled processes, including pollutant enrichment near the catalytic interface, formation of the F-FeSAC/PI complex, electronic coupling between the pollutant and the F-FeSAC/PI complex, molecular accessibility, charge-transfer reorganization, and subsequent chemical transformation after the initial electron-transfer event. Thus, forcing a strict global linear relationship between $\varphi_{1/2}$ and ln(k_{obs}) would overstate the predictive capability and mechanistic meaning of a single electrochemical descriptor.

Revision in Fig. 3k

In the revised Fig. 3k and corresponding discussion, BA and NB are retained within the same contaminant set and discussed as high-$\varphi_{1/2}$, electron-deficient pollutants with limited electron-transfer accessibility. Their low degradation rates are therefore interpreted as a mechanistically meaningful observation that electron-deficient pollutants are unfavorable for donating electrons to the F-FeSAC/PI complex, rather than as data points to be removed from a fitted line. It should be noted that these

revisions, including the removal of both the 1.2 V boundary and the linear interpretation, do not alter the central conclusion that electron-rich pollutants are more favorable for ETP-mediated oxidation, whereas electron-deficient pollutants exhibit limited electron-transfer accessibility.

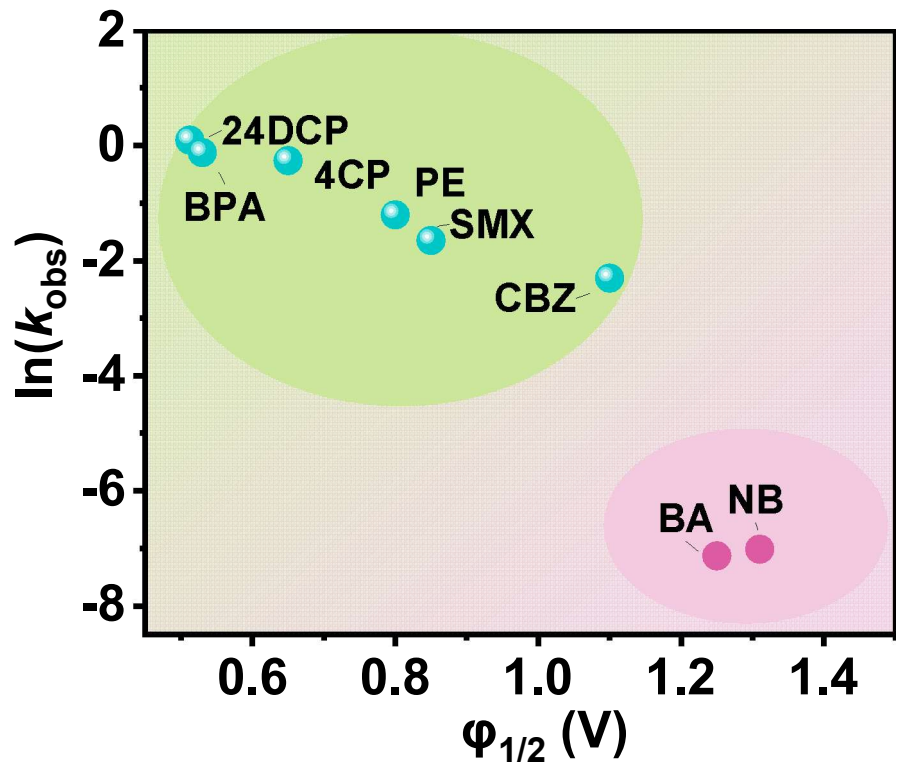

Revised Fig. 3 (k) Distribution of ln(k_{obs}) as a function of $\varphi_{1/2}$ for the eight contaminants.

Relationship with the frontier-orbital analysis in Fig. 4a-c

We have further revised the discussion of Fig. 4a-c to ensure consistency with the $\varphi_{1/2}$ analysis. BA and NB remain included in the frontier-orbital analysis, but this analysis is no longer described as evidence that all pollutants obey a strict ETP-controlled kinetic relationship. Instead, the frontier-orbital energy alignment between the F-FeSAC/PI complex and the pollutant is used as a complementary molecular-level descriptor of electron-transfer accessibility. Pollutants with higher HOMO levels and smaller $LUMO_{(F\text{-}FeSAC/PI)}$–$HOMO_{(pollutant)}$ gaps are more favorable for electron donation to the F-FeSAC/PI complex, whereas BA and NB show unfavorable orbital alignment and correspondingly low reactivity.

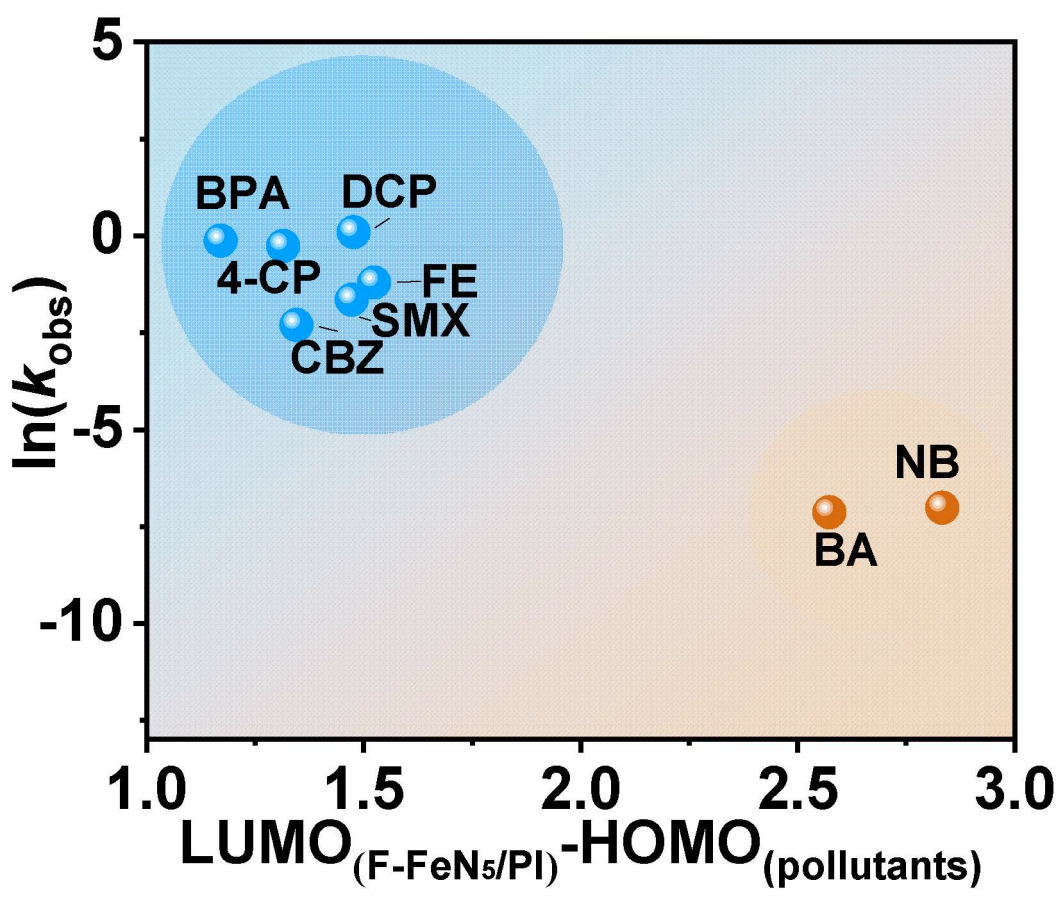

Revised Fig. 4 (b) Relationship between $LUMO_{(R\text{-}FeSAC/PI)}$-$HOMO_{(pollutants)}$ and ln(k_{obs}).

Taken together, the revised analysis no longer relies on a 1.2 V threshold or on selective exclusion of BA and NB to improve the coefficient of determination. Both $\varphi_{1/2}$ and frontier-orbital energy alignment are now used consistently to describe the accessibility of different pollutants toward interfacial electron transfer. These revisions do not change the central mechanistic conclusion. Instead, they provide a more consistent physicochemical interpretation: electron-rich pollutants are more favorable for ETP-mediated oxidation, whereas electron-deficient pollutants such as BA and NB show limited reactivity because of their unfavorable electron-donating ability.

Action 1: To make this distinction clearer, we have revised the manuscript as follows:

Revised text in the Abstract:

"The feasibility of the ETP pathway is further supported by consistent relationships among pollutant half-wave potentials ($\varphi_{1/2}$), the $LUMO_{(catalyst/PI)}$–$HOMO_{(pollutant)}$ energy gap, and degradation reactivity, indicating that pollutant electron-donating ability strongly influences reaction selectivity."

Revised text in the Introduction:

"Theoretical calculations further show that efficient ETP is favored when the pollutant HOMO is energetically well aligned with the LUMO of the catalyst/PI complex."

Revised text in the "Relationship between Degradation Performances and Properties of Pollutants" section:

*"To systematically evaluate the catalytic performance of the F-FeSAC/PI system, degradation experiments were conducted using eight representative contaminants: 4-CP, bisphenol A (BPA), 2,4-dichlorophenol (2,4-DCP), phenol (PE), carbamazepine (CBZ), sulfamethoxazole (SMX), nitrobenzene (NB), and benzoic acid (BA). To assess whether substrate redox accessibility contributes to the observed contaminant-dependent reactivity[6], the $\varphi_{1/2}$ of these eight contaminants were measured by cyclic voltammetry and used as experimental descriptors of their electron-donating propensity[7, 8]. In the F-FeSAC/PI system, pollutants with relatively lower $\varphi_{1/2}$ values, including 2,4-DCP, PE, CBZ, BPA, 4-CP, and SMX, generally exhibited higher degradation rates, whereas BA and NB showed higher $\varphi_{1/2}$ values and much lower $\ln(k_{obs})$ values (**Fig. 3k**). This behavior is consistent with the presence of electron-withdrawing –COOH and $–NO_2$ groups, which decrease the electron density of the aromatic ring and render oxidation less favorable (Supplementary Fig. 29). These*

results indicate that pollutant oxidation potential is closely associated with interfacial electron-transfer accessibility in the F-FeSAC/PI system, with electron-rich substrates being more favorable for ETP-mediated oxidation than electron-deficient substrates."

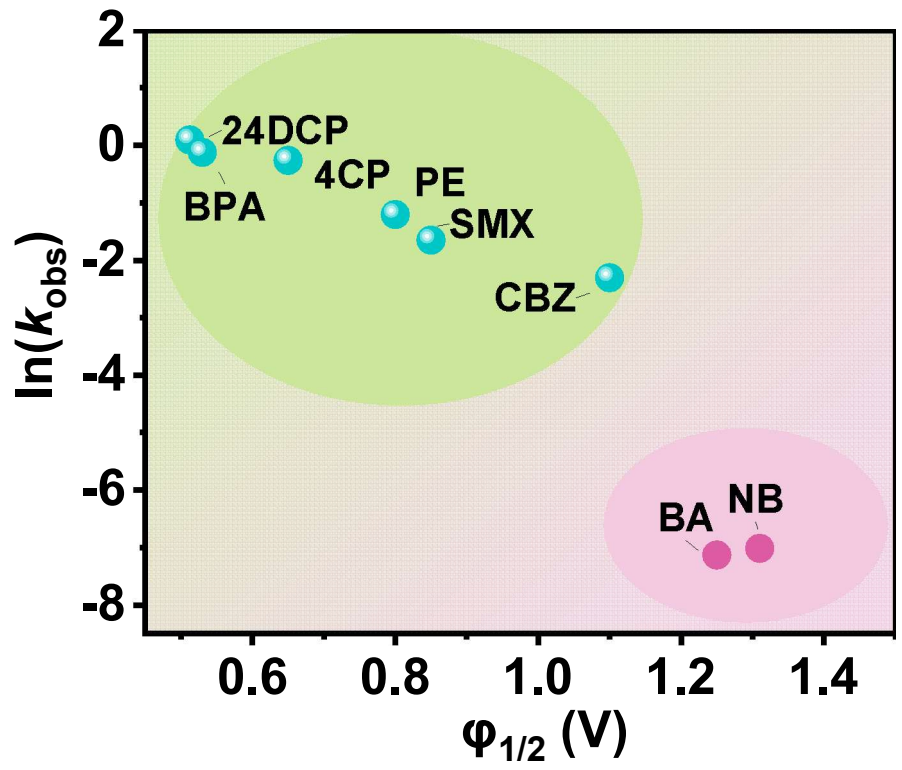

Revised Fig. 3(k) Distribution of ln(k_{obs}) as a function of $\varphi_{1/2}$ for the eight contaminants.

Revised text in the "Mechanistic Insights into the Electron Transfer Pathway in R-FeSAC/PI Composites" section:

*"To further clarify the substrate-dependent ETP reactivity, the LUMO$_{(R\text{-}FeSAC/PI)}$–HOMO$_{(pollutant)}$ energy gaps were compared with the observed ln(k_{obs}) values for the same eight contaminants (**Fig. 4b**). Pollutants with smaller energy gaps generally showed higher degradation rates, indicating that closer frontier-orbital alignment facilitates electron donation from the pollutant to the R-FeSAC/PI complex[9]. In contrast, BA and NB were located in the large-gap/low-reactivity region, consistent with their high $\varphi_{1/2}$ values and weak electron-donating character. Overall, higher pollutant HOMO levels and smaller catalyst/PI-pollutant energy gaps favor interfacial electron transfer, while electron-deficient pollutants with unfavorable orbital alignment show limited ETP accessibility."*

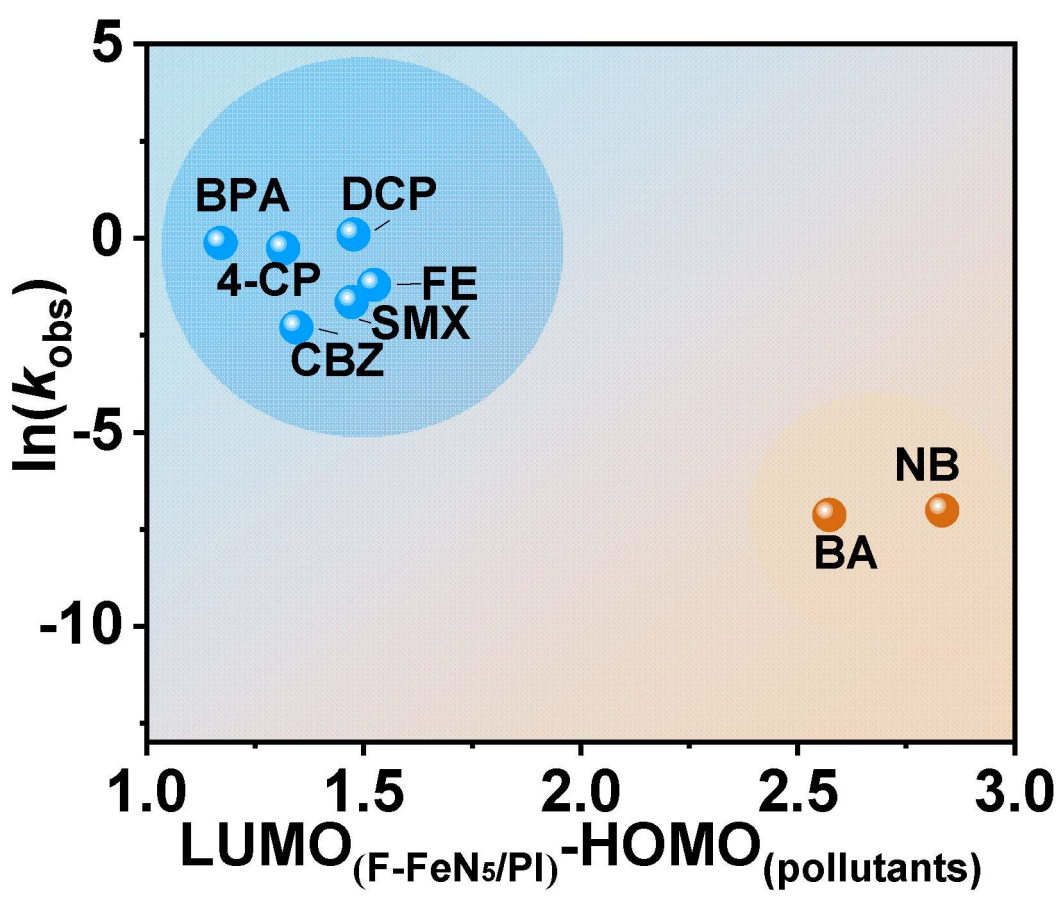

Revised Fig. 4 (b) Relationship between LUMO$_{(R\text{-}FeSAC/PI)}$-HOMO$_{pollutants}$ and ln(k_{obs}).

Revised text in the Discussion:

"The consistent trends among pollutant $\varphi_{1/2}$, $LUMO_{(catalyst/PI)}$–$HOMO_{(pollutant)}$ energy alignment, and degradation reactivity indicate that pollutant electron-donating ability and frontier-orbital matching jointly govern electron-transfer accessibility and reaction selectivity."

2. In Fig. 4b, CBZ appears to be excluded from the dataset despite its inclusion in Fig. 4a and 4c. Clarification as to whether this exclusion is intentional is requested.

Response 2:

We thank the reviewer for raising this concern. The original Fig. 4b compared the experimentally measured half-wave potential, $\varphi_{1/2}$, with the theoretically calculated frontier-orbital energy gap, $LUMO_{(F\text{-}FeSAC/PI)}$–$HOMO_{(pollutant)}$. Both descriptors are related to the electron-donating ability and oxidation accessibility of the pollutants, but they are not equivalent. The $\varphi_{1/2}$ value is influenced by electrochemical measurement conditions and interfacial electrode processes, whereas the calculated orbital energy gap depends on the molecular model, electronic-structure method, and assumed molecular state.

As discussed in our response to Comment 1, we have revised the manuscript to avoid using strict linear relationships or R^2-based arguments to support the $\varphi_{1/2}$ and frontier-orbital analyses. In this revised framework, $\varphi_{1/2}$ and the calculated $LUMO_{(F\text{-}FeSAC/PI)}$–$HOMO_{(pollutant)}$ gap are treated as complementary descriptors rather than as two quantities that should be interpreted through a direct quantitative descriptor-to-descriptor correlation. Therefore, retaining the original Fig. 4b could overemphasize the relationship between two non-equivalent descriptors. To avoid this potential overinterpretation and to maintain consistency with the revised mechanistic discussion, we have removed the original Fig. 4b from the revised manuscript.

After removal of the original auxiliary descriptor-to-descriptor comparison, the remaining Fig. 4 analyses focus on the relationship between frontier-orbital alignment and pollutant degradation kinetics. In these retained analyses, all tested pollutants are included together. The revised figure set therefore uses a consistent contaminant series for the mechanistic comparisons that remain in the manuscript. **The removal of the original Fig. 4b does not affect the central mechanistic conclusion.** The retained analyses continue to support that electron-rich pollutants are more accessible to ETP-mediated oxidation, whereas electron-deficient pollutants exhibit limited electron-

transfer accessibility.

The corresponding figure, caption, and text have been revised accordingly, and the subsequent panels have been renumbered to maintain consistency throughout the revised manuscript.

3. The difference in the Fe d-band center among the F-, H-, and NH_2-FeSAC variants is on the order of 0.02 eV. While a difference of this magnitude is consistent with the modification of solely peripheral substituents upon an otherwise invariant Fe-N-C framework, it resides within the characteristic uncertainty of DFT and is therefore of limited reliability as a quantitative descriptor. Consequently, the deterministic phrasing invoked in the abstract and discussion ("optimizes" or "upshifts" the Fe d-band center) is arguably overstated. The authors are requested either to establish the robustness of this ordering or to attenuate the causal language accordingly.

Response 3:

We thank the reviewer for this important and constructive comment. The central conclusion of this work does not rely solely on the approximately 0.02 eV differences in the calculated Fe *d*-band centers. More pronounced substituent-dependent differences are observed in the PI-adsorbed states. Specifically, F-FeSAC exhibits the strongest PI adsorption, the shortest Fe–O bond length, the most negative ICOHP value, the weakest occupied antibonding contribution near the Fermi level, and the strongest Fe–O orbital hybridization. These descriptors are more directly related to PI binding and activation than the *d*-band center. Nevertheless, we agree with the reviewer that describing such small differences in the Fe *d*-band center as “optimizes” or “upshifts” the Fe *d*-band center may overstate their quantitative significance. We have therefore moderated the relevant language throughout the revised manuscript.

It is also important to note that these theoretical trends are consistent with the experimental results. F-FeSAC exhibits the highest degradation rate constant, the strongest electrochemical response upon PI addition, the most pronounced OCP change after pollutant addition, the lowest charge-transfer resistance, and the highest current response in the galvanic oxidation system. Taken together, the computational and experimental results indicate that the enhanced activity of F-FeSAC arises from the combined effects of stronger PI adsorption, and Fe–O bonding, favorable interfacial charge redistribution, and improved electron-transfer accessibility.

Action 3: We have revised the relevant statements in the Abstract, Introduction, Results section "Mechanistic Insights into the Electron Transfer Pathway in R-FeSAC/PI Composites," and Discussion to avoid overinterpreting the small Fe *d*-band-center difference. The specific revisions are listed below.

Revised text in the Abstract:

"Among them, F-FeSAC exhibits superior pollutant degradation performance over its H- and NH_2-substituted counterparts, owing to long-range electronic regulation that reconfigures the Fe-site microenvironment and strengthens PI adsorption and activation."

"Fluorine substitution electronically modulates the Fe site through an electron-withdrawing effect, leading to reinforced Fe–O interaction with PI and accelerated direct electron-transfer kinetics."

Revised text in the Introduction:

"Mechanistic studies reveal that this enhancement originates from long-range electronic effects, whereby electron-withdrawing peripheral groups reconfigure the Fe-site electronic microenvironment, strengthen PI binding, and facilitate an efficient ETP between pollutants and the catalyst/PI complex."

Revised text in the "Mechanistic Insights into the Electron Transfer Pathway in R-FeSAC/PI Composites" section:

"The calculated Fe d-band centers of F-FeSAC, H-FeSAC, and NH_2-FeSAC are −2.470, −2.484, and −2.494 eV, respectively (Fig. 4c). Although the differences are small, this trend is consistent with a subtle perturbation of the Fe 3d electronic structure induced by peripheral substitution, which is further reflected in the orbital-resolved DOS near the Fermi level. In particular, the dz^2 and d_{xz}/d_{yz} components, which are closely related to the subsequent Fe–O interfacial interaction with adsorbed PI, show clear substituent-dependent differences. Compared with H-FeSAC and NH_2-FeSAC, F-FeSAC exhibits more pronounced Fe 3d contributions in the energy region relevant to PI coordination, particularly for orbitals capable of coupling with the O-centered states of PI. This orbital arrangement provides an electronic basis for the stronger Fe–O interaction formed upon PI adsorption."

"The adsorption energy (E_{ads}) follows the same trend, with values of –3.38 eV for F-FeSAC, –3.26 eV for H-FeSAC, and –3.19 eV for NH_2-FeSAC (Fig. 4d and

Supplementary Fig. 33), supporting stronger PI binding on F-FeSAC.

Overall, these results demonstrate that beyond-second-coordination-shell substituents remotely regulate the electronic structure of Fe single-atom sites and thereby govern PI adsorption and activation (Fig. 4f-g). Specifically, the electron-withdrawing F substituent reconfigures the Fe-site electronic microenvironment, reshapes the frontier 3d orbital distribution near the Fermi level, and strengthens Fe–O orbital hybridization. Consequently, F-FeSAC exhibits the shortest Fe–O bond, the most negative ICOHP value, the weakest occupied antibonding contribution, and the strongest PI adsorption among the three catalysts. This electronically and structurally reinforced Fe–O interaction facilitates catalyst/PI intermediate formation and promotes interfacial electron transfer, ultimately accounting for the superior PI activation and pollutant oxidation performance of F-FeSAC."*

Revised text in the Discussion:

"Crucially, electron-withdrawing fluorine substitution enhances the catalytic performance of F-FeSAC compared with H- and NH_2-substituted analogues by remotely reconfiguring the Fe-site electronic microenvironment without altering the immediate Fe–N_4 coordination shell. This remote regulation strengthens PI adsorption and enables a direct ETP for selective pollutant removal."

4. In Fig. 4e, the reported adsorption energies and Fe-O bond lengths deviate substantially from those presented in the original manuscript (F-FeSAC/PI: Fe-O 1.64 to 2.16 Å; Eads -2.02 to -3.38 eV, with similar shifts for H- and NH_2-FeSAC). Shifts of this magnitude imply a completely different optimized adsorption geometry, yet the molecular structures in Fig. 4e remain unchanged between versions. Please explain the origin of these modified values and provide the actual optimized structures that correspond to the reported Eads and bond distances.

Response 4:

We thank the reviewer for raising this point. During the previous round of revision, we re-optimized the adsorption geometries and recalculated the adsorption energies using the similar computational settings described in the Methods section. The revised adsorption energies and Fe-O bond lengths reported in Fig. 4e were obtained from these newly optimized structures. The difference between the previously reported and revised values originates from a renewed geometry optimization, which identified more stable relaxed adsorption configurations than those used in the previous version. The

numerical values now reported in Fig. 4d correspond to these fully optimized structures.

However, when preparing the revised figure, we inadvertently updated the numerical values but did not replace the old structural images with the corresponding newly optimized geometries. This caused the mismatch between the reported values and the displayed structures. We sincerely apologize for this oversight.

We have now corrected the previous Fig. 4e, which is now Fig. 4d in the revised manuscript, by replacing the previous structural images with the actual optimized structures corresponding to the reported adsorption energies and Fe-O bond lengths. For F-FeSAC/PI, the updated optimized structure gives a Fe-O bond length of 2.16 Å and an adsorption energy of −3.38 eV. The optimized structures for H-FeSAC/PI and NH_2-FeSAC/PI have also been updated accordingly. To ensure transparency and reproducibility, we have added the optimized atomic coordinates to the Supplementary Data. All structures were fully relaxed until the convergence criteria were satisfied.

This correction only updates the structural presentation in the previous Fig. 4e, now Fig. 4d in the revised manuscript, and does not affect the conclusions, which are based on the recalculated adsorption energies and the corresponding optimized geometries.

We appreciate the reviewer's careful examination, which helped us improve the accuracy and clarity of the revised manuscript.

Action 4: We have updated the figure, namely the previous Fig. 4e, which is now Fig. 4d in the revised manuscript. The previous structural images have been replaced with the corresponding newly optimized structures.

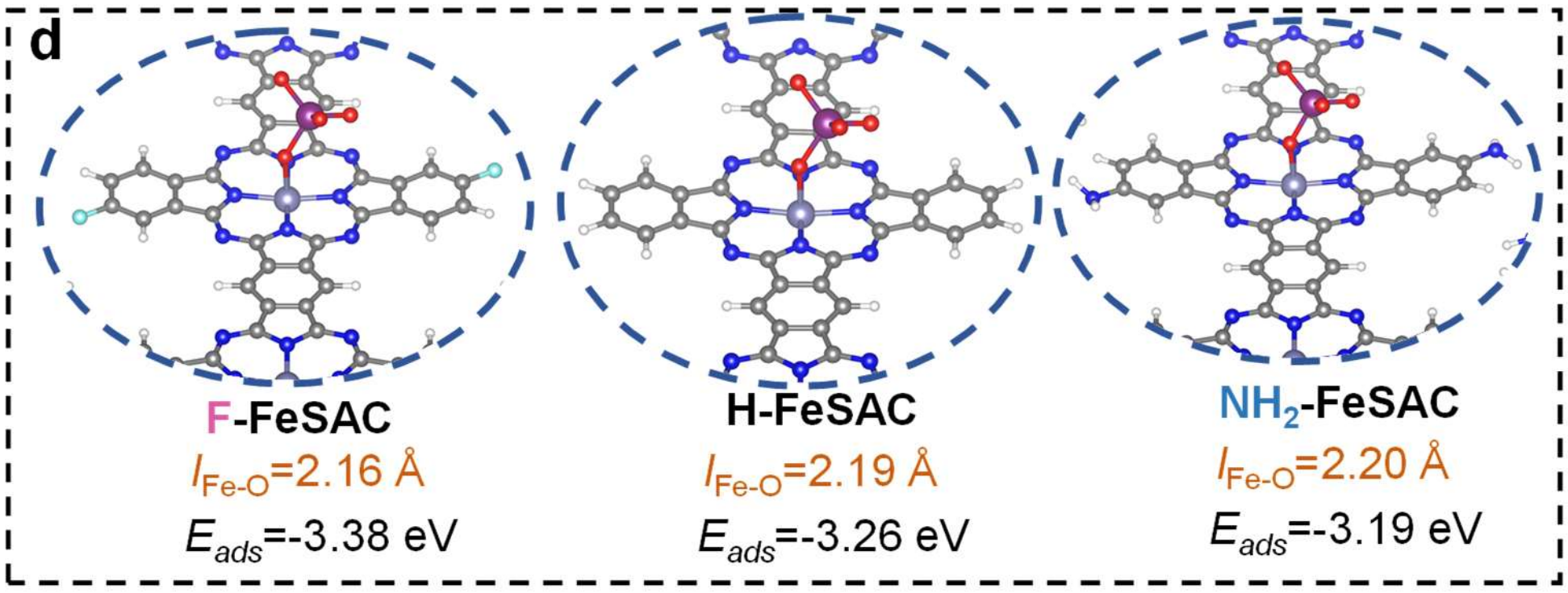

Fig. 4 (d) Optimized adsorption configurations of PI at the Fe site on the F-FeSAC, H-FeSAC, and NH_2-FeSAC.

References

1. Zhang P, *et al.* Origins of selective oxidation in carbon-based nonradical oxidation processes toward organic pollutants: Quantitative structure-activity relationships (QSARs). *Environ. Sci. Technol.* **58**, 4781-4791 (2024).
2. Yang M, *et al.* Unveiling the origins of selective oxidation in single-atom catalysis via Co–N_4–C intensified radical and nonradical pathways. *Environ. Sci. Technol.* **56**, 11635-11645 (2022).
3. Pan J, *et al.* Insights into the enhanced oxidation of organic micropollutants by single-atom Cu catalyst activated peroxydisulfate: Valence-dominated nonradical pathway. *Appl. Catal. B* **351**, 123997 (2024).
4. Kuss-Petermann M, Wenger OS. Unusual distance dependences of electron transfer rates. *Phys. Chem. Chem. Phys.* **18**, 18657-18664 (2016).
5. Marcus RA, Sutin N. Electron transfers in chemistry and biology. *Biochim. Biophys. Acta Bioenerg.* **811**, 265-322 (1985).
6. Yu Z, *et al.* Decoupled oxidation process enabled by atomically dispersed copper electrodes for in-situ chemical water treatment. *Nat. Commun.* **15**, 1186 (2024).
7. Cheng C, Ren W, Miao F, Chen X, Chen X, Zhang H. Generation of FeIV=O and its contribution to Fenton-like reactions on a single-atom iron−N−C catalyst. *Angew. Chem. Int. Ed.* **62**, e202218510 (2023).
8. Ren W, *et al.* Activation of peroxydisulfate on carbon nanotubes: Electron-transfer mechanism. *Environ. Sci. Technol.* **53**, 14595-14603 (2019).
9. Yin K, *et al.* Pore modulation of single atomic Fe sites for ultrafast Fenton-like chemistry with amplified electron migration oxidation. *Water Res.* **268**, 122545 (2025).

Responses to Reviewer's Comments

The revised manuscript entitled "**Enzyme-Mimicking Peripheral Substituents beyond the Second Coordination Sphere Tune Single-Atom Catalysts for Periodate Activation**" (Manuscript ID: NCOMMS-26-004517C)

Reviewer #3 (Remarks to the Author):

The authors address well my comments, so I would like to recommend this manuscript to publish in Nat. Commun.

Response: We are grateful for the reviewer's positive assessment and acceptance recommendation.
