# Supplementary material for: Enzyme-mimicking peripheral substituents beyond the second coordination sphere tune single-atom catalysts for periodate activation
Source: Nat Commun. 2026 Sep 17;17:10926. doi: 10.1038/s41467-026-77805-5 (PMC13680503; doi:10.1038/s41467-026-77805-5)
Supplement: Supplementary file 3 [file 41467_2026_77805_MOESM3_ESM.pdf]

Description of Additional Supplementary Files

Title: Supplementary Data 1

Description: Supplementary Data 1 provides the atomic coordinates of the catalyst's electronic structure as required, with figure legends including 'Serial number,' 'Element,' 'Element sequence number,' 'X-axis spatial position,' 'Y-axis spatial position,' and 'Z-axis spatial position.'
