# Supplementary material for: Enzyme-mimicking peripheral substituents beyond the second coordination sphere tune single-atom catalysts for periodate activation
Source: Nat Commun. 2026 Sep 17;17:10926. doi: 10.1038/s41467-026-77805-5 (PMC13680503; doi:10.1038/s41467-026-77805-5)
Supplement: Supplementary file 4 [file 41467_2026_77805_MOESM4_ESM.pdf]

DFT-Optimized F-FeSAC/PI Atomic Coordinates

Serial number	element	Element sequence number	x-axis space position	y-axis space position	z-axis space position
1	N	N1	0.52166	0.61273	0.20082
2	N	N2	0.42915	0.52087	0.20177
3	N	N3	0.61344	0.52106	0.19812
4	N	N4	0.52123	0.42898	0.20059
5	N	N5	0.40902	0.63311	0.20324
6	N	N6	0.63401	0.63332	0.19539
7	N	N7	0.4087	0.40861	0.20202
8	N	N8	0.63375	0.40876	0.2013
9	N	N9	0.02094	0.11152	0.20321
10	N	N10	0.92992	0.02078	0.20156
11	N	N11	0.11186	0.0209	0.20163
12	N	N12	0.02081	0.93017	0.2032
13	N	N13	0.90865	0.13313	0.20238
14	N	N14	0.13316	0.13329	0.20213
15	N	N15	0.90858	0.90842	0.20199
16	N	N16	0.13309	0.90854	0.20248
17	N	N17	0.52097	0.11136	0.20348
18	N	N18	0.4299	0.02083	0.2019
19	N	N19	0.61195	0.0209	0.20167
20	N	N20	0.52095	0.93035	0.20345
21	N	N21	0.40861	0.13311	0.20254
22	N	N22	0.63335	0.13314	0.20264
23	N	N23	0.40861	0.90854	0.20308
24	N	N24	0.63332	0.90865	0.20188
25	C	C1	0.98734	0.71356	0.20261
26	C	C2	0.82747	0.55329	0.20346
27	C	C3	0.05332	0.71538	0.20306
28	C	C4	0.82914	0.48746	0.20504
29	C	C5	0.21326	0.55436	0.20521
30	C	C6	0.21503	0.48848	0.20479
31	C	C7	0.95369	0.76977	0.20258
32	C	C8	0.7725	0.58853	0.20092
33	C	C9	0.08851	0.77038	0.20332
34	C	C10	0.7728	0.45386	0.20424
35	C	C11	0.98816	0.32631	0.20326
36	C	C12	0.05414	0.32814	0.20282
37	C	C13	0.46884	0.65026	0.20275
38	C	C14	0.39141	0.57346	0.2031

39	C	C15	0.57429	0.65049	0.19875
40	C	C16	0.39144	0.46832	0.2024
41	C	C17	0.48805	0.71578	0.20332
42	C	C18	0.32578	0.55403	0.2043
43	C	C19	0.55484	0.71592	0.20119
44	C	C20	0.3258	0.48787	0.20388
45	C	C21	0.65121	0.57366	0.19716
46	C	C22	0.46853	0.3914	0.20197
47	C	C23	0.65119	0.46845	0.20028
48	C	C24	0.57393	0.39144	0.20171
49	C	C25	0.71681	0.55402	0.19945
50	C	C26	0.71677	0.48793	0.20137
51	C	C27	0.45269	0.77089	0.20479
52	C	C28	0.26959	0.58799	0.20516
53	C	C29	0.58984	0.77125	0.20186
54	C	C30	0.27006	0.4533	0.2044
55	C	C31	0.48777	0.32591	0.20358
56	C	C32	0.55461	0.3259	0.20354
57	C	C33	0.96833	0.15003	0.20283
58	C	C34	0.89152	0.07332	0.20276
59	C	C35	0.07348	0.1503	0.20263
60	C	C36	0.8915	0.96824	0.20261
61	C	C37	0.98776	0.21553	0.20314
62	C	C38	0.8262	0.05418	0.2046
63	C	C39	0.05389	0.21566	0.20292
64	C	C40	0.82617	0.9874	0.20452
65	C	C41	0.15027	0.07346	0.20271
66	C	C42	0.96825	0.89141	0.20252
67	C	C43	0.15025	0.96835	0.20287
68	C	C44	0.0734	0.89164	0.20285
69	C	C45	0.21561	0.05425	0.20465
70	C	C46	0.2156	0.98748	0.20477
71	C	C47	0.95303	0.27126	0.20347
72	C	C48	0.77095	0.08945	0.20508
73	C	C49	0.08786	0.27198	0.20282
74	C	C50	0.7709	0.95217	0.20492
75	C	C51	0.98776	0.82604	0.20275
76	C	C52	0.05389	0.82615	0.20304
77	C	C53	0.46837	0.15003	0.20331
78	C	C54	0.3915	0.07339	0.20278
79	C	C55	0.57356	0.15002	0.20335
80	C	C56	0.3915	0.96826	0.20309
81	C	C57	0.48765	0.21533	0.20415
82	C	C58	0.32615	0.0542	0.20459

83	C	C59	0.55433	0.21531	0.20418
84	C	C60	0.32614	0.98745	0.20476
85	C	C61	0.65039	0.07343	0.20274
86	C	C62	0.46838	0.89163	0.20357
87	C	C63	0.65036	0.96835	0.20245
88	C	C64	0.57356	0.89174	0.20267
89	C	C65	0.71573	0.05421	0.20445
90	C	C66	0.71571	0.98748	0.20431
91	C	C67	0.45251	0.27072	0.20427
92	C	C68	0.2709	0.08945	0.20505
93	C	C69	0.58965	0.27059	0.20428
94	C	C70	0.27088	0.95223	0.20532
95	C	C71	0.48774	0.82633	0.20405
96	C	C72	0.55442	0.82644	0.20309
97	H	H1	0.77308	0.63976	0.20053
98	H	H2	0.77239	0.40259	0.20583
99	H	H3	0.13974	0.76965	0.20365
100	H	H4	0.90241	0.76994	0.20236
101	H	H5	0.26986	0.63928	0.20555
102	H	H6	0.26944	0.40206	0.20428
103	H	H7	0.64109	0.77135	0.20122
104	H	H8	0.40145	0.77061	0.20599
105	H	H9	0.77087	0.1407	0.20495
106	H	H10	0.77077	0.90092	0.2048
107	H	H11	0.13914	0.27186	0.20255
108	H	H12	0.90179	0.27187	0.2037
109	H	H13	0.27096	0.1407	0.20477
110	H	H14	0.27094	0.90098	0.20519
111	H	H15	0.64091	0.27051	0.20441
112	H	H16	0.40125	0.27083	0.20436
113	H	H17	0.16781	0.57812	0.20545
114	H	H18	0.87452	0.46366	0.20703
115	H	H19	0.0778	0.37365	0.20254
116	H	H20	0.96362	0.66808	0.20238
117	F	F1	0.15876	0.45717	0.20458
118	F	F2	0.88378	0.58455	0.20448
119	F	F3	0.08458	0.6593	0.20335
120	F	F4	0.95697	0.38241	0.20357
121	Fe	Fe1	0.52131	0.52091	0.19085
122	Fe	Fe2	0.02088	0.02084	0.18585
123	Fe	Fe3	0.52091	0.02085	0.18592
124	I	I1	0.56109	0.54666	0.37053
125	O	O1	0.51702	0.51573	0.29868
126	O	O2	0.63152	0.58311	0.34011

127	O	O3	0.51104	0.60301	0.4101
128	O	O4	0.57817	0.48028	0.4222

DFT-Optimized H-FeSAC/PI Atomic Coordinates

Serial number	element	Element sequence number	x-axis space position	y-axis space position	z-axis space position
1	N	N1	0.52165	0.6128	0.1979
2	N	N2	0.42933	0.52103	0.20138
3	N	N3	0.61355	0.52128	0.19537
4	N	N4	0.52147	0.42935	0.20052
5	N	N5	0.40904	0.63324	0.20193
6	N	N6	0.63403	0.6335	0.19242
7	N	N7	0.40894	0.40879	0.20232
8	N	N8	0.63397	0.40904	0.20012
9	N	N9	0.02088	0.11173	0.20263
10	N	N10	0.93001	0.02065	0.20246
11	N	N11	0.11187	0.02106	0.2025
12	N	N12	0.02097	0.92996	0.20264
13	N	N13	0.90859	0.13303	0.20234
14	N	N14	0.13306	0.1335	0.20236
15	N	N15	0.90876	0.90826	0.20228
16	N	N16	0.13324	0.90866	0.20241
17	N	N17	0.52108	0.11181	0.20251
18	N	N18	0.43014	0.02107	0.2028
19	N	N19	0.61182	0.02102	0.20256
20	N	N20	0.52097	0.93029	0.20283
21	N	N21	0.40878	0.13341	0.20243
22	N	N22	0.63341	0.1333	0.20257
23	N	N23	0.40869	0.90873	0.20331
24	N	N24	0.63326	0.90873	0.20173
25	C	C1	0.98819	0.71368	0.20145
26	C	C2	0.82893	0.5543	0.20359
27	C	C3	0.05446	0.71381	0.20152
28	C	C4	0.82885	0.48812	0.20539
29	C	C5	0.21368	0.55385	0.20531
30	C	C6	0.21377	0.48763	0.20519
31	C	C7	0.95397	0.76966	0.20222
32	C	C8	0.77296	0.58842	0.19919
33	C	C9	0.08846	0.76992	0.20237
34	C	C10	0.77276	0.45405	0.20319
35	C	C11	0.98681	0.32777	0.20162
36	C	C12	0.05309	0.32806	0.20162
37	C	C13	0.46887	0.65033	0.20077
38	C	C14	0.39154	0.5736	0.20233

39	C	C15	0.5743	0.65056	0.196
40	C	C16	0.39154	0.46846	0.20238
41	C	C17	0.48808	0.71579	0.20179
42	C	C18	0.32587	0.55409	0.20393
43	C	C19	0.55483	0.71594	0.1992
44	C	C20	0.32592	0.48791	0.20388
45	C	C21	0.65142	0.5739	0.1941
46	C	C22	0.46878	0.39173	0.20227
47	C	C23	0.6513	0.46872	0.198
48	C	C24	0.57416	0.39178	0.20144
49	C	C25	0.71701	0.55432	0.19662
50	C	C26	0.71693	0.48824	0.19898
51	C	C27	0.4527	0.77088	0.20409
52	C	C28	0.26972	0.58806	0.20481
53	C	C29	0.58984	0.77125	0.20018
54	C	C30	0.26995	0.45366	0.20461
55	C	C31	0.48805	0.3263	0.20399
56	C	C32	0.55485	0.32629	0.20377
57	C	C33	0.96824	0.15017	0.20247
58	C	C34	0.89158	0.07321	0.20313
59	C	C35	0.07334	0.15047	0.20247
60	C	C36	0.89159	0.96804	0.20312
61	C	C37	0.98749	0.21567	0.20275
62	C	C38	0.82626	0.05403	0.20475
63	C	C39	0.05362	0.21591	0.20274
64	C	C40	0.82622	0.98724	0.20477
65	C	C41	0.15025	0.07371	0.20315
66	C	C42	0.96847	0.89127	0.20241
67	C	C43	0.1503	0.96848	0.20319
68	C	C44	0.07358	0.89147	0.20249
69	C	C45	0.21563	0.05452	0.20479
70	C	C46	0.21565	0.98772	0.20484
71	C	C47	0.953	0.27156	0.20239
72	C	C48	0.77103	0.08936	0.20514
73	C	C49	0.08749	0.27219	0.20239
74	C	C50	0.77083	0.95215	0.20517
75	C	C51	0.98805	0.82582	0.20264
76	C	C52	0.05419	0.82595	0.20271
77	C	C53	0.46853	0.15042	0.20279
78	C	C54	0.39171	0.07369	0.20321
79	C	C55	0.57367	0.15036	0.20287
80	C	C56	0.39169	0.96848	0.20367
81	C	C57	0.48782	0.21575	0.20397
82	C	C58	0.32632	0.05449	0.20482

83	C	C59	0.55453	0.2157	0.20402
84	C	C60	0.32631	0.98772	0.20503
85	C	C61	0.65035	0.07356	0.2032
86	C	C62	0.46842	0.89166	0.20335
87	C	C63	0.65024	0.96842	0.203
88	C	C64	0.57353	0.89173	0.20206
89	C	C65	0.71569	0.05426	0.20475
90	C	C66	0.71562	0.98751	0.20472
91	C	C67	0.45273	0.27115	0.20437
92	C	C68	0.27099	0.08968	0.20516
93	C	C69	0.58988	0.27095	0.20428
94	C	C70	0.27099	0.95253	0.20538
95	C	C71	0.48772	0.82631	0.20355
96	C	C72	0.55441	0.82641	0.20216
97	H	H1	0.77245	0.6397	0.19846
98	H	H2	0.77206	0.40278	0.20512
99	H	H3	0.13976	0.77048	0.20241
100	H	H4	0.90267	0.76999	0.20216
101	H	H5	0.27009	0.63937	0.20482
102	H	H6	0.27061	0.40236	0.20439
103	H	H7	0.64108	0.77131	0.19905
104	H	H8	0.40146	0.77052	0.20568
105	H	H9	0.77115	0.14061	0.20493
106	H	H10	0.77065	0.90088	0.20508
107	H	H11	0.13878	0.27201	0.2023
108	H	H12	0.9017	0.27079	0.20233
109	H	H13	0.271	0.14094	0.20495
110	H	H14	0.27097	0.90127	0.20528
111	H	H15	0.64114	0.27089	0.20419
112	H	H16	0.40147	0.27141	0.20419
113	H	H17	0.16879	0.57879	0.20573
114	H	H18	0.16897	0.46253	0.20546
115	H	H19	0.87373	0.57929	0.20618
116	H	H20	0.87357	0.4631	0.20916
117	H	H21	0.07957	0.66902	0.20077
118	H	H22	0.07787	0.37303	0.20085
119	H	H23	0.96157	0.37248	0.20083
120	H	H24	0.96327	0.66879	0.20066
121	Fe	Fe1	0.52135	0.52104	0.18883
122	Fe	Fe2	0.02094	0.02084	0.18632
123	Fe	Fe3	0.52097	0.02101	0.18596
124	I	I1	0.56559	0.54899	0.36887
125	O	O1	0.51911	0.51883	0.29834
126	O	O2	0.63515	0.58562	0.33645

127	O	O3	0.51734	0.60511	0.41125
128	O	O4	0.58471	0.48254	0.41965

DFT-Optimized NH_2-FeSAC/PI Atomic Coordinates

Serial number	element	Element sequence number	x-axis space position	y-axis space position	z-axis space position
1	N	N1	0.52028	0.6121	0.19745
2	N	N2	0.42905	0.51997	0.19756
3	N	N3	0.61263	0.52082	0.19743
4	N	N4	0.52188	0.42849	0.19781
5	N	N5	0.40754	0.63203	0.20012
6	N	N6	0.63265	0.63314	0.1937
7	N	N7	0.40963	0.40741	0.19984
8	N	N8	0.63427	0.40869	0.20343
9	N	N9	0.02078	0.11253	0.20442
10	N	N10	0.93001	0.02124	0.20206
11	N	N11	0.11241	0.02308	0.20142
12	N	N12	0.0216	0.93205	0.20339
13	N	N13	0.90823	0.13336	0.20285
14	N	N14	0.13307	0.13539	0.20105
15	N	N15	0.90947	0.90881	0.2025
16	N	N16	0.13405	0.91094	0.20204
17	N	N17	0.52168	0.11119	0.20385
18	N	N18	0.43038	0.02102	0.20335
19	N	N19	0.61201	0.02007	0.20303
20	N	N20	0.52072	0.92973	0.20338
21	N	N21	0.40949	0.13344	0.20289
22	N	N22	0.63396	0.13237	0.20389
23	N	N23	0.4083	0.90881	0.20383
24	N	N24	0.63302	0.90773	0.20177
25	N	N25	0.15844	0.44733	0.21898
26	N	N26	0.88428	0.58963	0.21254
27	N	N27	0.09213	0.66018	0.20621
28	N	N28	0.95096	0.38374	0.17498
29	C	C1	0.99262	0.71575	0.20808
30	C	C2	0.82767	0.55677	0.21026
31	C	C3	0.05939	0.71649	0.20674
32	C	C4	0.82764	0.49047	0.21787
33	C	C5	0.2133	0.54741	0.2099
34	C	C6	0.21468	0.48087	0.21375
35	C	C7	0.95729	0.77055	0.20639
36	C	C8	0.77071	0.5896	0.20312
37	C	C9	0.09167	0.77399	0.20403
38	C	C10	0.77215	0.45598	0.21573

39	C	C11	0.98354	0.32754	0.18251
40	C	C12	0.05042	0.32802	0.18155
41	C	C13	0.46729	0.64956	0.19982
42	C	C14	0.39042	0.57218	0.19967
43	C	C15	0.57279	0.64999	0.19627
44	C	C16	0.39214	0.46702	0.20004
45	C	C17	0.48652	0.71514	0.20143
46	C	C18	0.32535	0.55161	0.20293
47	C	C19	0.5533	0.71525	0.19924
48	C	C20	0.32646	0.48546	0.20403
49	C	C21	0.65006	0.57363	0.1965
50	C	C22	0.46947	0.39064	0.19934
51	C	C23	0.65099	0.46862	0.20263
52	C	C24	0.57466	0.39111	0.20111
53	C	C25	0.71572	0.55463	0.20153
54	C	C26	0.71589	0.4887	0.2066
55	C	C27	0.45158	0.77056	0.20394
56	C	C28	0.26783	0.58328	0.20523
57	C	C29	0.58862	0.77036	0.20035
58	C	C30	0.27241	0.44923	0.2097
59	C	C31	0.48886	0.32542	0.20206
60	C	C32	0.55563	0.32554	0.20308
61	C	C33	0.96785	0.15055	0.2027
62	C	C34	0.89135	0.07348	0.20366
63	C	C35	0.07312	0.1519	0.20139
64	C	C36	0.89193	0.96845	0.20298
65	C	C37	0.98665	0.21612	0.19866
66	C	C38	0.82617	0.05393	0.20557
67	C	C39	0.0528	0.21663	0.19763
68	C	C40	0.82645	0.98715	0.20498
69	C	C41	0.1507	0.07575	0.2025
70	C	C42	0.96937	0.89247	0.20302
71	C	C43	0.15088	0.97075	0.20272
72	C	C44	0.07443	0.89403	0.20246
73	C	C45	0.21619	0.05653	0.2048
74	C	C46	0.21605	0.98974	0.20498
75	C	C47	0.95113	0.27068	0.19228
76	C	C48	0.77073	0.08879	0.20642
77	C	C49	0.08531	0.27335	0.18949
78	C	C50	0.77131	0.9516	0.20514
79	C	C51	0.98969	0.82773	0.20366
80	C	C52	0.05578	0.82849	0.20294
81	C	C53	0.46933	0.14999	0.20347
82	C	C54	0.39223	0.07381	0.20375

83	C	C55	0.57433	0.14965	0.20416
84	C	C56	0.39166	0.96868	0.20429
85	C	C57	0.48873	0.21515	0.20407
86	C	C58	0.32682	0.05516	0.20541
87	C	C59	0.55541	0.21504	0.20472
88	C	C60	0.32646	0.98842	0.20573
89	C	C61	0.65054	0.07252	0.20416
90	C	C62	0.46795	0.89135	0.20377
91	C	C63	0.65035	0.96737	0.20315
92	C	C64	0.57314	0.89098	0.2023
93	C	C65	0.71573	0.05324	0.20569
94	C	C66	0.7158	0.9865	0.20504
95	C	C67	0.45353	0.27038	0.20315
96	C	C68	0.27191	0.09106	0.20541
97	C	C69	0.59075	0.27029	0.20486
98	C	C70	0.27087	0.95385	0.20594
99	C	C71	0.4869	0.82588	0.20366
100	C	C72	0.55361	0.82573	0.20227
101	H	H1	0.76946	0.64079	0.19922
102	H	H2	0.77214	0.40498	0.22186
103	H	H3	0.14296	0.77619	0.20344
104	H	H4	0.906	0.76941	0.20787
105	H	H5	0.26605	0.63454	0.20307
106	H	H6	0.27531	0.39799	0.2116
107	H	H7	0.63986	0.77005	0.19933
108	H	H8	0.40034	0.77065	0.20558
109	H	H9	0.77026	0.14005	0.20673
110	H	H10	0.77161	0.90033	0.20446
111	H	H11	0.1366	0.27407	0.18751
112	H	H12	0.89981	0.26876	0.19273
113	H	H13	0.27274	0.14232	0.20498
114	H	H14	0.27019	0.9026	0.20586
115	H	H15	0.642	0.27026	0.20565
116	H	H16	0.40229	0.27052	0.20259
117	H	H17	0.16735	0.57014	0.20987
118	H	H18	0.87244	0.46662	0.22673
119	H	H19	0.07458	0.37265	0.17266
120	H	H20	0.96934	0.67024	0.21212
121	H	H21	0.16306	0.40298	0.23755
122	H	H22	0.12274	0.4707	0.24203
123	H	H23	0.92303	0.5645	0.19938
124	H	H24	0.88312	0.63273	0.19053
125	H	H25	0.06948	0.62243	0.22599
126	H	H26	0.13814	0.66263	0.21971

127	H	H27	0.90506	0.38001	0.16119
128	H	H28	0.97395	0.41819	0.14955
129	Fe	Fe1	0.52098	0.52033	0.18706
130	Fe	Fe2	0.02114	0.0223	0.18602
131	Fe	Fe3	0.52118	0.0205	0.18703
132	I	I1	0.55538	0.54613	0.36954
133	O	O1	0.51394	0.51469	0.29637
134	O	O2	0.62613	0.5834	0.34105
135	O	O3	0.50356	0.60171	0.40784
136	O	O4	0.57209	0.48033	0.42224
