# Supplementary material for: Enzyme-mimicking peripheral substituents beyond the second coordination sphere tune single-atom catalysts for periodate activation
Source: Nat Commun. 2026 Sep 17;17:10926. doi: 10.1038/s41467-026-77805-5 (PMC13680503; doi:10.1038/s41467-026-77805-5)
Supplement: Supplementary file 5 [file 41467_2026_77805_MOESM5_ESM.pdf]

nature portfolio

Corresponding author(s): Tao Zeng
Last updated by author(s): Aug 10, 2026

Reporting Summary

Nature Portfolio wishes to improve the reproducibility of the work that we publish. This form provides structure for consistency and transparency in reporting. For further information on Nature Portfolio policies, see our Editorial Policies and the Editorial Policy Checklist.

Statistics

For all statistical analyses, confirm that the following items are present in the figure legend, table legend, main text, or Methods section.

n/a	Confirmed	
☐	☒	The exact sample size (*n*) for each experimental group/condition, given as a discrete number and unit of measurement
☐	☒	A statement on whether measurements were taken from distinct samples or whether the same sample was measured repeatedly
☐	☒	The statistical test(s) used AND whether they are one- or two-sided<br>*Only common tests should be described solely by name; describe more complex techniques in the Methods section.*
☒	☐	A description of all covariates tested
☒	☐	A description of any assumptions or corrections, such as tests of normality and adjustment for multiple comparisons
☐	☒	A full description of the statistical parameters including central tendency (e.g. means) or other basic estimates (e.g. regression coefficient) AND variation (e.g. standard deviation) or associated estimates of uncertainty (e.g. confidence intervals)
☐	☒	For null hypothesis testing, the test statistic (e.g. *F*, *t*, *r*) with confidence intervals, effect sizes, degrees of freedom and *P* value noted<br>*Give P values as exact values whenever suitable.*
☒	☐	For Bayesian analysis, information on the choice of priors and Markov chain Monte Carlo settings
☒	☐	For hierarchical and complex designs, identification of the appropriate level for tests and full reporting of outcomes
☒	☐	Estimates of effect sizes (e.g. Cohen's *d*, Pearson's *r*), indicating how they were calculated

Our web collection on statistics for biologists contains articles on many of the points above.

Software and code

Policy information about availability of computer code

Data collection	Vienna Ab-initio Simulation Package (VASP) for DFT and all other data are obtained by direct experimentation. No custom code was used.
Data analysis	VESTA 3.5.8, Origin 2021, XPSPEAK41; Jade9; CytExpert software, version 2.5

For manuscripts utilizing custom algorithms or software that are central to the research but not yet described in published literature, software must be made available to editors and reviewers. We strongly encourage code deposition in a community repository (e.g. GitHub). See the Nature Portfolio guidelines for submitting code & software for further information.

Data

Policy information about availability of data

All manuscripts must include a data availability statement. This statement should provide the following information, where applicable:

- Accession codes, unique identifiers, or web links for publicly available datasets
- A description of any restrictions on data availability
- For clinical datasets or third party data, please ensure that the statement adheres to our policy

The data generated in this study are provided within the article and the Supplementary Information file. Additional data are available from the corresponding authors upon request. Source data are provided with this paper.

Research involving human participants, their data, or biological material

Policy information about studies with human participants or human data. See also policy information about sex, gender (identity/presentation), and sexual orientation and race, ethnicity and racism.

Reporting on sex and gender	N/A
Reporting on race, ethnicity, or other socially relevant groupings	N/A
Population characteristics	N/A
Recruitment	N/A
Ethics oversight	N/A

Note that full information on the approval of the study protocol must also be provided in the manuscript.

Field-specific reporting

Please select the one below that is the best fit for your research. If you are not sure, read the appropriate sections before making your selection.

☐ Life sciences ☐ Behavioural & social sciences ☒ Ecological, evolutionary & environmental sciences

For a reference copy of the document with all sections, see nature.com/documents/nr-reporting-summary-flat.pdf

Ecological, evolutionary & environmental sciences study design

All studies must disclose on these points even when the disclosure is negative.

Study description	Single-atom catalysts (SACs) have attracted extensive interest as enzyme-mimetic active centers; however, faithfully reproducing the long-range regulation exerted by distal binding pockets in natural metalloenzymes remains a formidable challenge. To investigate how such underexplored long-range effects influence SA center for advanced oxidation reactions, we precisely synthesized a series of conjugated organic SACs with tunable peripheral substituents beyond the second coordination sphere (denoted as R-FeSAC, where R = F, H, NH2). These catalysts reveal that functional groups in second-beyond coordination shells can remotely regulate periodate (PI) activation and advanced oxidation performance. Among them, F-FeSAC exhibits superior pollutant degradation performance over its H- and NH2-substituted counterparts, owing to long-range electronic modulation that optimizes the Fe d-band configuration. Mechanistic studies combining in situ Raman spectroscopy, galvanic oxidation assays, and electrochemical analysis reveal that R-FeSAC activates PI to generate high-potential metastable intermediates, thereby facilitating a direct electron-transfer pathway (ETP) for selective pollutant removal via polymerization, rather than radical or singlet-oxygen pathways. Fluorine substitution optimizes the Fe d-band center through electron withdrawal, and accelerates direct electron-transfer kinetics. The feasibility of the ETP pathway is further supported by a strong correlation between pollutant half-wave potentials($\phi_{1/2}$) and the LUMO (catalyst/PI)–HOMO (pollutant) energy gap, reflecting reaction selectivity dictated by pollutant electronics. Notably, the F-functionalized membrane exhibits outstanding durability under continuous-flow operation, and a pilot-scale ETP reactor achieves efficient degradation of real wastewater without iodate leakage. This work establishes peripheral functionalization beyond the second coordination sphere as an enzyme-inspired design principle for PI-based advanced oxidation and provides a sustainable, polymerization-mediated paradigm for advanced water purification.
Research sample	The research samples were synthetic Fe single-atom catalysts, including FeSAC and R-FeSACs with peripheral substituents beyond the second coordination sphere (R = F, H, NH2). They were selected to evaluate how long-range substituent effects regulate Fe–N4sites for periodate activation and electron-transfer-mediated pollutant oxidation. 4-Chlorophenol was used as the main model pollutant, while additional contaminants and real wastewater samples from pharmaceutical manufacturing and fine chemical industries were used to assess broader applicability.
Sampling strategy	No statistical sample-size calculation was performed. Sample sizes were chosen based on standard practice for catalyst synthesis, catalytic degradation tests, and mechanistic experiments. Each degradation experiment was performed in triplicate, which was sufficient to assess reproducibility and compare catalytic performance among different systems. Error bars represent the average of three parallel experiments.
Data collection	All experimental data were collected and recorded by the authors of the manuscript using the specific apparatuses and procedures described in the Methods section.
Timing and spatial scale	Data were collected during the experimental period of this study. No seasonal or periodic sampling design was involved. Samples were taken at predetermined time intervals to monitor degradation kinetics and oxidant consumption. The data were mainly generated at laboratory scale, with additional bench/pilot-scale continuous-flow and enlarged reactor tests.
Data exclusions	No data were excluded from the analysis.

Reproducibility	Reproducibility was verified by repeating key catalytic degradation experiments in triplicate and by confirming consistent trends across catalyst batches, characterization results, and mechanistic tests. Continuous-flow and wastewater-treatment experiments were also repeated under the stated operating conditions. All attempts to repeat the reported experiments were successful.
Randomization	Samples were assigned to predefined experimental groups according to catalyst type, pollutant substrate, control condition, or treatment condition. Random allocation was not applicable to the main catalyst and degradation experiments. Key variables were controlled by using fixed catalyst dosage, PI concentration, pollutant concentration, temperature, and pH.
Blinding	Blinding was not used because the study was based on predefined catalyst systems and instrumental measurements rather than subjective outcome assessment. Blinding was therefore not relevant to data acquisition or analysis.

Did the study involve field work? ☐ Yes ☒ No

Reporting for specific materials, systems and methods

We require information from authors about some types of materials, experimental systems and methods used in many studies. Here, indicate whether each material, system or method listed is relevant to your study. If you are not sure if a list item applies to your research, read the appropriate section before selecting a response.

Materials & experimental systems

n/a	Involved in the study
☒	☐ Antibodies
☒	☐ Eukaryotic cell lines
☒	☐ Palaeontology and archaeology
☐	☒ Animals and other organisms
☒	☐ Clinical data
☒	☐ Dual use research of concern
☒	☐ Plants

Methods

n/a	Involved in the study
☒	☐ ChIP-seq
☐	☒ Flow cytometry
☒	☐ MRI-based neuroimaging

Animals and other research organisms

Policy information about studies involving animals; ARRIVE guidelines recommended for reporting animal research, and Sex and Gender in Research

Laboratory animals	This study involved adult zebrafish (about 4 months).
Wild animals	The study did not involve wild animals.
Reporting on sex	Sex was considered only in the breeding setup, which included one female and two male adult zebrafish, but not in the embryo exposure design or analysis because sex cannot be reliably determined at the eight-cell stage.
Field-collected samples	The study did not involve field-collected samples.
Ethics oversight	The study was approved by the Animal Experimentation Ethics Committee of Zhejiang University of Technology (approval no. 231005016; 15 March 2023) and conducted in accordance with the national guideline "Laboratory Animal-Guideline for Ethical Review of Animal Welfare (GB/T 35892-2018).

Note that full information on the approval of the study protocol must also be provided in the manuscript.

Plants

Seed stocks	N/A
Novel plant genotypes	N/A
Authentication	N/A

Flow Cytometry

Plots

Confirm that:

☐ The axis labels state the marker and fluorochrome used (e.g. CD4-FITC).

☒ The axis scales are clearly visible. Include numbers along axes only for bottom left plot of group (a 'group' is an analysis of identical markers).

☒ All plots are contour plots with outliers or pseudocolor plots.

☐ A numerical value for number of cells or percentage (with statistics) is provided.

Methodology

Sample preparation	The E. coli strain was obtained from the American Type Culture Collection (ATCC), and beef extract was purchased from Beijing Coolabor Biotechnology Co., Ltd. Briefly, 10 µL of E. coli was inoculated into 10 mL of nutrient medium (3 g/L beef extract, 10 g/L tryptone, 5 g/L NaCl, pH 7.2) and incubated on a rotary shaker at 30 °C for 10 h. Cells were harvested by centrifugation at 402 × g for 5 min and washed three times with phosphate-buffered saline (PBS, 0.5 M, pH 7.4). Next, 1 mL of the prepared bacterial suspension was added to 10 mL of the reaction solution collected at different time points (0 and 40 min). After incubation for 9 h, the mixtures were centrifuged at 402 × g for 5 min, washed three times with PBS. and adjusted to approximately 1 × 106 cellsmL-1. SYTO 9 and propidium iodide (PI) were added at final concentrations of 3.34 and 20µM, respectively. After incubation for 15min at room temperature in the dark, the samples were analyzed directly without further washing.
Instrument	CytoFLEX LX flow cytometer (Beckman Coulter)
Software	CytExpert software, version 2.5, was used for data acquisition and analysis.
Cell population abundance	No cell sorting was performed; therefore, post-sort cell population abundance and sample purity were not applicable.
Gating strategy	The PBS negative control was used to identify background noise and define the boundary of Gate 1. Single-stained SYTO 9 and PI controls were used for fluorescence compensation between the FL1 and FL3 channels. Heat-killed bacteria prepared at 70°C for 30min served as the PI-positive reference. The same gating criteria were applied to the PBS control and water samples to directly compare bacterial population abundance and viability.

☒ Tick this box to confirm that a figure exemplifying the gating strategy is provided in the Supplementary Information.
